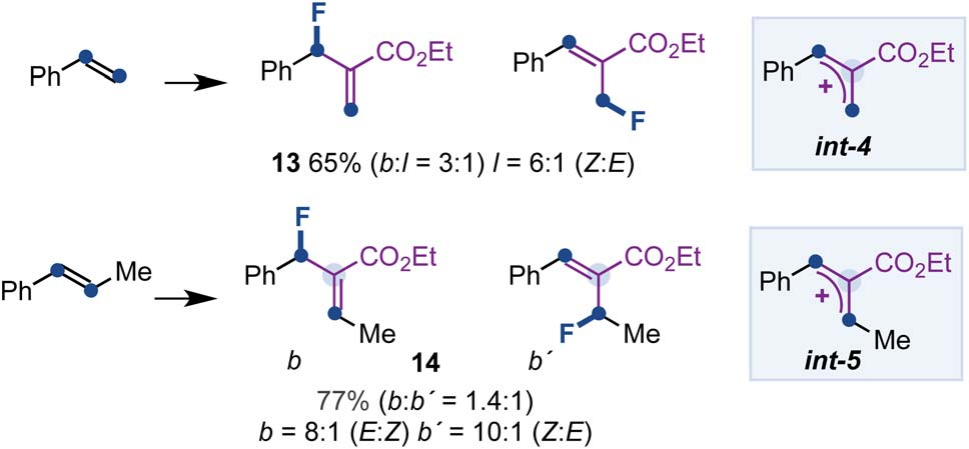# Catalytic alkene skeletal modification for the construction of fluorinated tertiary stereocenters†

**DOI:** 10.1039/d2sc00968d

**Published:** 2022-03-15

**Authors:** Liyin Jiang, Pau Sarró, Wei Jie Teo, Jordi Llop, Marcos G. Suero

**Affiliations:** Institute of Chemical Research of Catalonia (ICIQ), Barcelona Institute of Science and Technology. Av. Països Catalans, 16 43007 Tarragona Spain mgsuero@iciq.es; Departament de Química Analítica I Química Orgànica, Universitat Rovira I Virgili, C. Marcel·lí Domingo, 1 43007 Tarragona Spain; CIC BiomaGUNE, Basque Research and Technology Alliance 20014 San Sebastián Guipuzcoa Spain

## Abstract

Herein we describe the first construction of fluorinated tertiary stereocenters based on an alkene C(sp^2^)–C(sp^2^) bond cleavage. The new process, that takes advantage of a Rh-catalyzed carbyne transfer, relies on a branched-selective fluorination of tertiary allyl cations and is distinguished by a wide scope including natural products and drug molecule derivatives as well as adaptability to radiofluorination.

## Abstract 2

We report a previously unknown disconnection approach to valuable fluorinated tertiary stereocenters based on the skeletal modification of 1,1-disubstituted alkenes by a Rh-catalyzed carbyne transfer.

## Graphical abstract

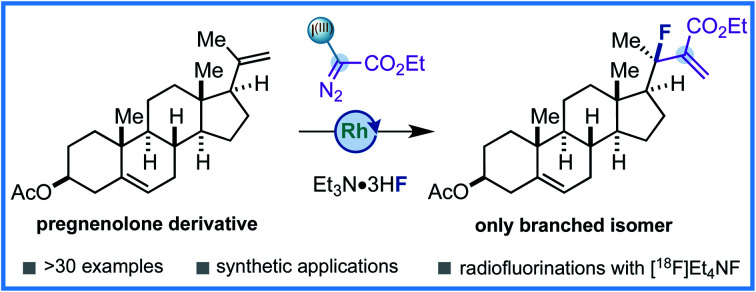

## Introduction

**Fig. 1:**
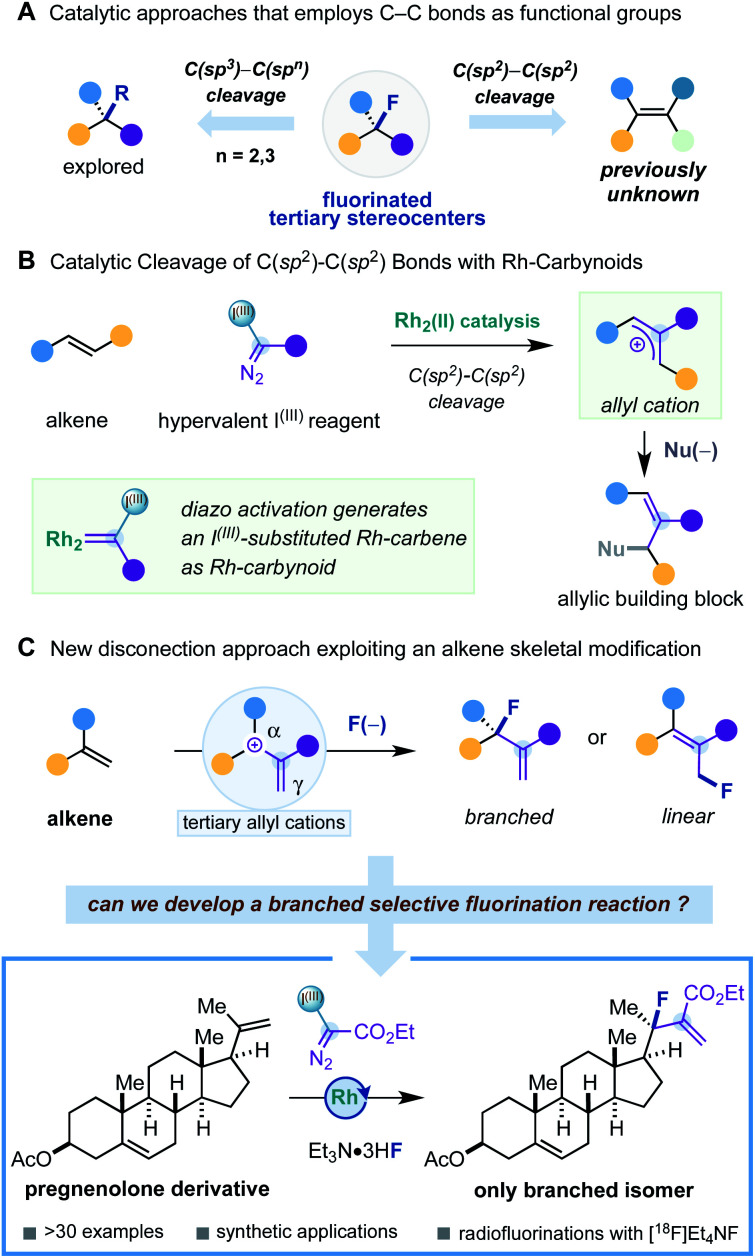
Fluorinated tertiary stereocenters.

The growing number of approved fluorinated small-molecule pharmaceuticals is a testimony of the tremendous research efforts in synthetic organofluorine chemistry^1^ and their application in fluorine-based drug design.^2^ The most prevalent chemotypes found in fluoro-pharmaceuticals feature monofluorinated moieties (Ar–F, Het–F, and alkyl–CH_2_F) and trifluoromethyl groups (Ar–CF_3_, Het–CF_3_, and alkyl–CF_3_).^2*d*^ However, the appearance of fluorinated tertiary stereocenters is very rare (Fig. 1A), despite this fluorinated motif being an ideal bioisostere of tertiary stereocenters – a prevalent motif in drug molecules – and being found in fludrocortisone – the first approved fluorine-containing drug. The main reason for the lack of fluorinated tertiary stereocenters in drug molecules can be attributed to a less developed area of research and difficulties of adopting the known synthetic methods for drug molecule design.^1*d*^ In this sense, it is of high contemporary interest to develop new synthetic concepts to such fluorinated motifs based on unconventional disconnection approaches that can expand and complement known synthetic protocols.

Catalytic methodologies based on (i) fluorination of alkenes, enol(ates), phenols and C–H bonds with electrophilic fluorinating reagents^3^ or in (ii) transformations of fluorine-containing starting materials,^4^ can be considered the most developed approaches for the regio- and enantioselective construction of fluorinated tertiary stereocenters. However, they respectively employ electrophilic fluorinating reagents – which are derived from fluorine gas – and use pre-functionalized fluorinated starting materials that could need multistep synthetic sequences. On the other hand, catalytic fluorination of alkenes, allylic electrophiles, and C–H bonds with nucleophilic fluoride sources represents in general an efficient option considering the availability of fluoride sources.^5,6^

Despite the variety of catalytic strategies developed, it is remarkable to observe that methodologies that employ C–C bonds as functional groups are scarce.^7^ Such processes rely on the skeletal modification of an organic molecule, offering new disconnection approaches.^8^ Examples of this class of fluorinations that reach fluorinated tertiary stereocenters are limited to the catalytic cleavage of C(sp^3^)–C(sp^2^) bonds of redox-active esters,^9^ carboxylic acids,^10^ and C(sp^3^)–C(sp^3^) bonds in cyclopropanes.^11^ However, to the best of our knowledge, synthesis of tertiary fluorinated stereocenters through catalytic alkene C(sp^2^)–C(sp^2^) bond cleavage is previously unknown (Fig. 1A).

As part of a research programme focused on the development of a carbyne transfer platform in organic synthesis, we reported a catalytic strategy that generates Rh-carbynoids as I^(III)^-substituted Rh-carbenes by selective diazo activation of bespoke hypervalent iodine reagents with a rhodium paddlewheel catalyst (Fig. 1B).^12,13^ We found that Rh-carbynoids provoked the skeletal modification of alkenes by formally inserting a cationic monovalent carbon unit (:^+^C–R) between both sp^2^-hybridized carbons. This constructive C(sp^2^)–C(sp^2^) bond cleavage process generated synthetically useful cation intermediates that converted to valuable chiral racemic allylic building blocks with a broad range of heteroatomic and carbon nucleophiles.

Recently, we wondered whether we could exploit our alkene skeletal modification platform for the catalytic conversion of 1,1-disubstituted alkenes into fluorinated tertiary stereocenters (Fig. 1C). We hypothesized that the fluoride nucleophilic attack would proceed with high branched selectivity considering that both the charge and the highest LUMO coefficient of the allyl cation may be centered at the α position, due to the double substitution with two stabilizing groups (alkyl and aromatic groups).^14,15^ However, we recognized that constructing a sterically demanding tertiary allylic fluoride could bring some problems associated with (i) parasitic proton eliminations promoted by fluoride and (ii) generation of undesirable branched/linear mixtures due to lack of regiocontrol in the nucleophilic fluoride attack.^16^

The successful development of such nucleophilic branched-selective fluorination of tertiary allyl cations would unlock a novel access to fluorinated tertiary stereocenters using readily or commercially available 1,1-disubstituted alkenes and nucleophilic fluoride sources. In addition, our strategy would represent also a novel approach to a class of allylic fluorides difficult to obtain with traditional bimolecular nucleophilic substitutions or transition metal-catalyzed platforms.^1*a*,*f*,17^

Herein, we would like to present the successful execution of this goal for a previously unknown disconnection approach to valuable fluorinated tertiary stereocenters based on the skeletal modification of 1,1-disubstituted alkenes. The synthetic protocol is amenable to a broad range of 1,1-disubstituted alkenes and permits the installation of a fluorinated tertiary stereocenter in natural products and drug molecule derivatives. Notably, the fluorination of the allyl cation intermediates occurred with excellent branched selectivity. Follow-up alkene transformations of the products and adaptability to radiofluorination were demonstrated.

## Results and discussion

The initial experiments were performed using α-methylstyrene (1a, 2 equiv.), hypervalent iodine reagent 2a (1 equiv.), tetrabutylammonium fluoride trihydrate Bu_4_NF·3H_2_O (3 equiv.) and the Du Bois catalyst Rh_2_(esp)_2_ (1 mol%)^18^ in dichloromethane (Table 1). We were pleased to find that branched allylic fluoride (±)-3a was formed in a promising 36% isolated yield with excellent branched/linear selectivity (*b* : *l* = >20 : 1). In addition to (±)-3a, alcohol (±)-4 and diene 5 were also formed as subproducts of the reaction in low yields. The formation of (±)-3a was proposed to proceed *via* (i) catalytic generation of a Rh-carbynoid by diazo activation of 2a; (ii) stereoselective cyclopropanation (int-1) to deliver a transient cyclopropyl–I^(III)^ intermediate int-2; (iii) disrotatory ring-opening (the Ph ring rotates inwardly, and Me rotates outwardly) to give allyl cation int-3; and (iv) regioselective fluoride attack. Finally, alcohol (±)-4 could be formed by hydrolysis of (±)-3a ^19^ or by a branched-selective water attack to int-3 ^19,20^ and diene 5 by proton elimination.

**Table 1:** Discovery and optimization studies^*a,b*^

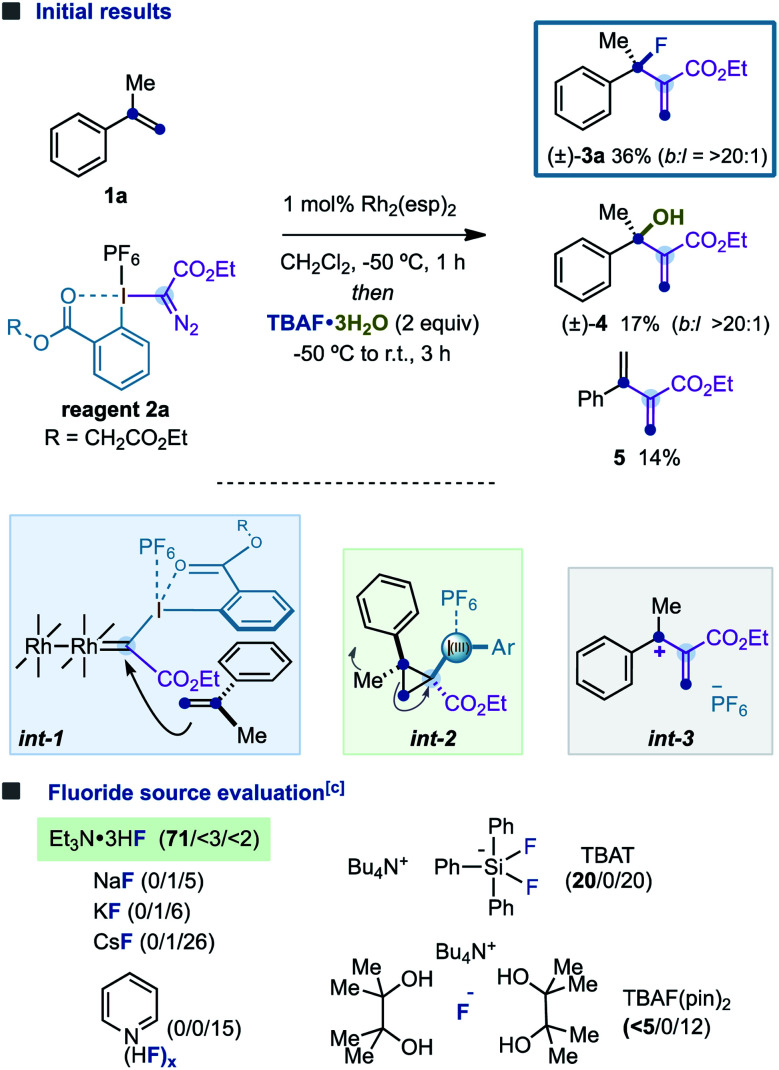

a Performed with 1a (0.2 mmol, 2 equiv.), 2a (0.1 mmol, 1 equiv.), Rh_2_(esp)_2_ (0.002 mmol, 1 mol%), and a fluoride source (3 equiv.) in CH_2_Cl_2_ (0.1 M).

b Yields are reported on the basis of ^1^H-NMR analysis using anisole as the internal standard; branched/linear ratio was determined by ^19^F NMR analysis.

c Yields in parentheses are of (±)-3a/(±)-4/5. esp = α,α,α′,α′-tetramethyl-1,3-benzenedipropanoate.

With these promising results, we were encouraged to optimize this novel fluorination reaction by evaluating a diverse variety of commercially available fluoride sources, hoping to improve the efficiency of the C–F bond-forming process while minimizing the parasitic proton elimination or water attack. We were pleased to find that Et_3_N·3HF led to (±)-3a in 71% isolated yield, and the formation of (±)-4 and 5 was certainly suppressed (<5% yield).^21^ However, while metallic fluorides MF (M = Na, K, Cs) and Olah's reagent [Py(HF)_*x*_] promoted the conversion to diene 5, tetrabutylammonium difluorotriphenylsilicate (TBAT) or the tetrabutylammonium fluoride bispinacol complex (TBAF(pin)_2_) provided (±)-3a in poor yields.^22,23^

With the optimized reaction conditions in hand, we next investigated the scope of this fluorination reaction by examining a broad range of α-substituted styrenes (Table 2). We were delighted to observe that substrates substituted in the *para* position of the aromatic ring with halogens [(±)-3b-e], trifluoromethyl [(±)-3f], ester [(±)-3g], trifluoromethoxy [(±)-3h], acyloxy [(±)-3i-j] and alkyne [(±)-3k] were well tolerated. However, methyl or methoxy substituents provided low levels of efficiency [(±)-3l] or no product [(±)-3m], as notable polymerizations were noticed with full starting material consumption. This observation might suggest that the corresponding allyl carbocation species are generated, before Et_3_N·3HF is added to the reaction, due to a significant acceleration in the ring-opening of cyclopropyl–I^(III)^ intermediates (int-2) caused by the electron-rich aromatic rings. We later hypothesized that these electron-donating groups may not provoke such significant acceleration in the ring-opening step when placed in a *meta* position. As predicted, the reactions carried out with *meta*-MeO- and *meta*-Me-substituted α-methylstyrenes provided (±)-3n-o with satisfactory yields and excellent branched/linear ratios. Moreover, *para*- and *meta*-disubstituted aromatic rings or naphthalene provided the desired products with high level of efficiencies [(±)-3p,q].

**Table 2:** Scope of tertiary allylic fluoride 3^*a,b*^

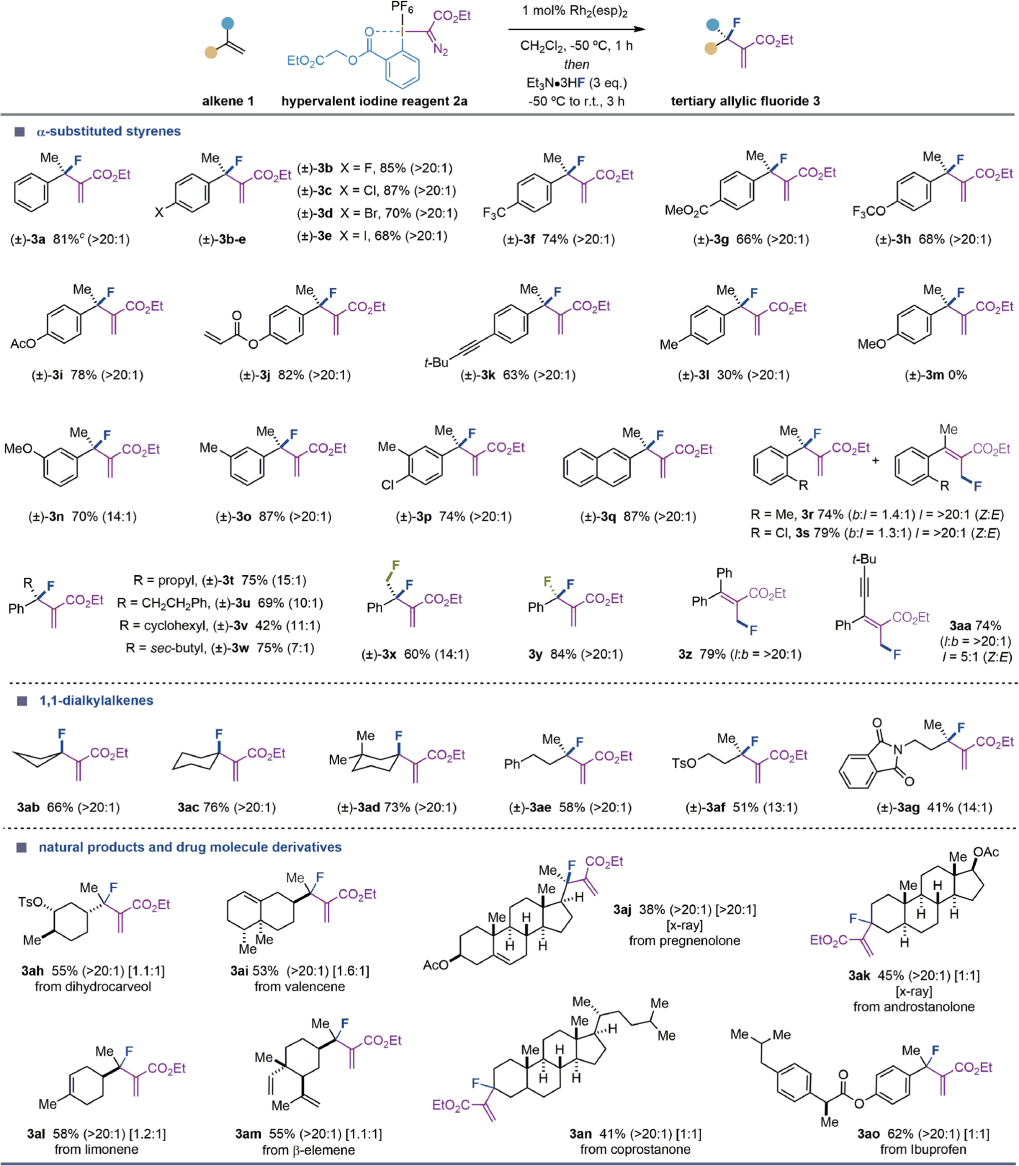

a Performed with 1 (0.4 mmol, 2 equiv.), 2a (0.2 mmol, 1 equiv.), Rh_2_(esp)_2_ (0.002 mmol, 1 mol%), and Et_3_N·3HF (3 equiv.) in CH_2_Cl_2_ (0.1 M). Yields are reported on the basis of the isolated pure product.

b Branched/linear ratio, indicated in parentheses, and the diastereoselectivity ratio, indicated in brackets, were determined by ^19^F NMR or ^1^H NMR analysis of the crude reaction mixture.

c Yield of the isolated product using 1.7 grams of 1a and 4.2 grams of 2a.

In contrast to the exquisite branched selectivity obtained for *para*- and *meta*-substituted α-methylstyrenes, a different situation was observed for *ortho*-substituted derivatives. Equimolecular mixtures of branched/linear fluorides were obtained (3r,s) when using substrates substituted with a methyl or a chlorine group. Although this is a clear limitation of our method, it underlines a potential subtle effect of the *ortho* substituent in preventing the aromatic ring from stabilizing the charge at the α position of the tertiary allyl cation.

After this, we decided to extend the scope by varying the substituent in the α position of the styrene (Table 2). We observed that styrenes substituted with alkyl groups such as propyl, phenethyl, cyclohexyl, or *iso*-butyl [(±)-3t-w] worked well; however, a general decrease in the branched/linear ratio was observed. Fluoromethyl and fluoro substituents were well tolerated and provided access to difluoromethyl and 1,2-difluoroethyl compounds (±)-3x,y with high efficiency. The latter results highlight an added-value of our methodology in accessing an interesting and useful subset of organofluorine compounds increasingly observed in newly approved drug molecules.^24^ Phenyl or alkyne groups did not provide the expected branched fluorides, and instead, the corresponding linear derivatives were obtained (3z,aa). These results indicate that these substituents may provoke a delocalization of the charge in the allylic π system; however, we do not have a clear explanation for the excellent linear selectivity observed. The latter examples led us to question the behavior of allyl carbocations doubly-substituted in the α position with alkyl groups, which are generated from 1,1-dialkyl substituted alkenes. We were delighted to find that exocyclic and acyclic aliphatic olefins were well tolerated and provide tertiary allylic fluorides (3ab-ag) with good to excellent branched/linear ratios.

Further demonstration of the potential of our methodology was validated in the fluorination of a selection of natural products and drug molecule derivatives (3ah-an) (Table 2). It is worth highlighting the excellent degree of chemoselectivity observed in substrates containing more than one alkene. The results indicate that highly substituted alkenes are less reactive; however, the excellent selectivity observed for β-elemene (3al) highlights that the initial alkene cyclopropanation to form a cyclopropyl–I^(III)^ intermediate is sensitive to steric effects.

We next aimed to transform the alkenyl-carboxylate moiety of (±)-3a into useful functionalities without compromising the integrity of the fluorinated tertiary stereocenter (Table 3). Hydrogenation (6), dihydroxylation (7), epoxidation (8) 1,3-cycloaddition reactions (9) and oxidation (10) provided a series of fluorinated derivatives that would be otherwise difficult to obtain by other means. To demonstrate the synthetic utility of our methodology in providing access to fluorinated analogues of medically relevant agents containing a tertiary stereocenter, we sought to synthesize (±)-F-flurbiprofen 12. Although this fluorinated analogue is known, it was synthesized using electrophilic reagents.^25^ Initially, we performed our fluorination reaction using a readily available styrene and obtained branched tertiary fluoride (±)-11 with high efficiency. Finally, oxidation with OsO_4_ transformed (±)-11 into the desired (±)-F-flurbiprofen 12 (Table 3).

**Table 3:** Synthetic applications

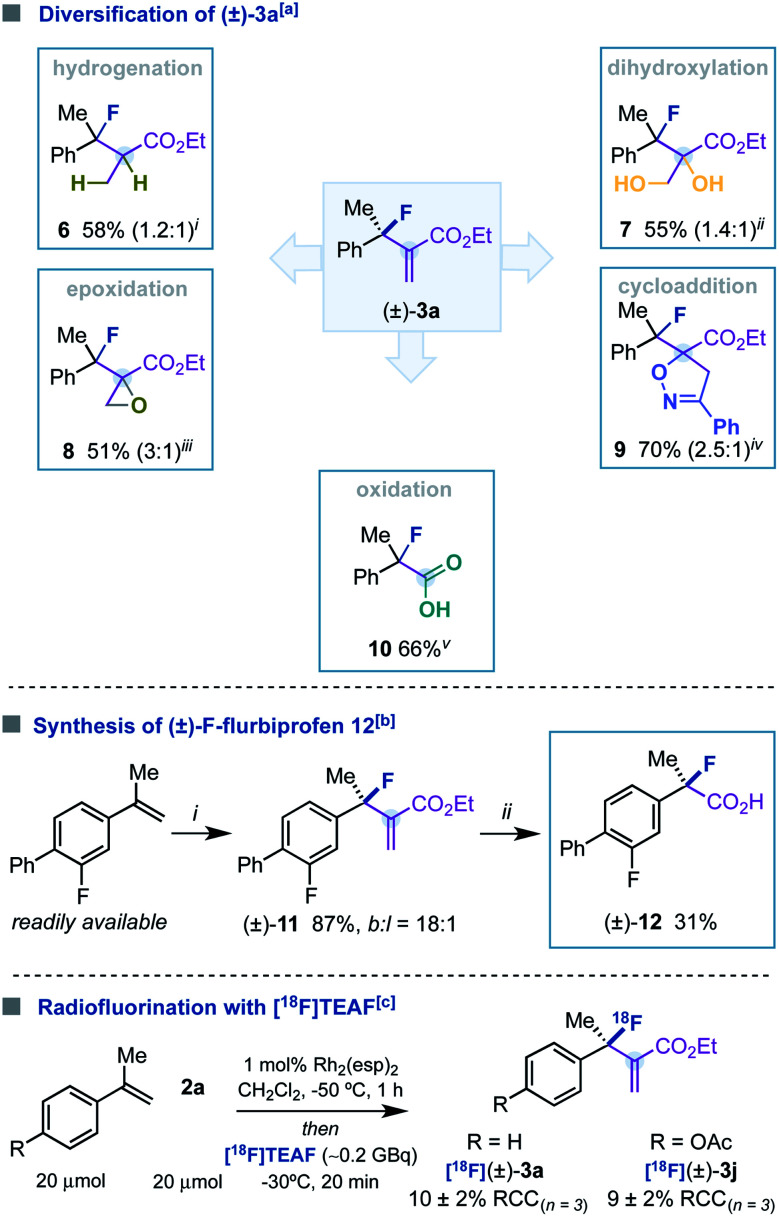

a Reaction conditions: (i) TsNHNH_2_, NaOEt, EtOH, 1 hour, and 80 °C; (ii) OsO_4_ (1 mol%), Oxone, DMF/H_2_O, 1 hour, and rt; (iii) *m*-CPBA, CH_2_Cl_2_, 14 hours, and reflux; (iv) chlorobenzaldoxime, Et_3_N, 5 hours, and 0 °C; (v) OsO_4_ (1 mol%), Oxone, DMF, 14 hours, and rt; then H_2_O_2_, 2M NaOH, and THF.

b (i) 2-fluoro-4-(prop-1-en-2-yl)-1,1′-biphenyl, Rh_2_(esp)_2_ (1 mol%), and Et_3_N·3HF; (ii) OsO_4_ (1 mol%), Oxone, DMF, 14 hours, and rt; then H_2_O_2_, 2M NaOH, and THF.

c 1a or 1j (20 μmol), 2a (20 μmol), Rh_2_(esp)_2_ (1 mol%), CH_2_Cl_2_, 1 hour, and −50 °C; *then* [^18^F]TEAF (∼0.2 GBq) in CH_2_Cl_2_ (100 μL), 20 min, and −50 °C → −30 °C. RCC was calculated with radio-HPLC with the number of replicates noted.

^18^Fluorine [^18^F] is emerging as one of the most prominent radionuclides in the application of positron emission tomography (PET), a fundamental technology in precision medicine for (pre)clinical imaging.^26^ The preparation of radioactive molecules containing a [^18^F] fluorinated tertiary stereocenter remains a critical challenge. These motifs cannot be synthesized by nucleophilic substitution of alkylsulfonates with [^18^F]KF-K_222_, and current synthetic methodologies are limited in scope.^9,27^ We wondered whether our fluorination reaction could be a suitable methodology for the synthesis of [^18^F] tertiary allylic fluorides. The initial experiments were performed with α-methylstyrene, reagent 2a, Rh_2_(esp)_2_, and [^18^F]KF-K_222_ (∼0.2 GBq). The latter [^18^F]-fluoride source was added at −50 °C, and the reaction mixture was warmed until −30 °C during 20 min. Unfortunately, the radiolabelled product [^18^F](±)-3a was not detected, probably due to the low solubility of [^18^F]KF-K_222_. However, when using [^18^F]tetraethylammonium fluoride [^18^F]TEAF (∼0.2 GBq),^28^ labelled product [^18^F](±)-3a was formed in 10% ± 2% (*n* = 3) of radiochemical conversion (RCC). In addition, we observed that the radiolabelling could also work with other substrates ([^18^F](±)-3j, 9% ± 2% (*n* = 3) RCC). However, attempts to improve the RCC were unsuccessful (Table 3).^29^

## Conclusions

In summary, we have developed a new synthetic methodology for the construction of fluorinated tertiary stereocenters from 1,1-disubstituted alkenes. The process relies on the generation of tertiary allyl cations, mediated by a catalytically-generated Rh-carbynoid, that undergoes nucleophilic fluorination with an excellent branched/selectivity ratio. Notable features of this process are the broad scope of 1,1-disubstituted alkenes, including natural products and drug molecule derivatives, applications in the synthesis of a fluorinated drug molecule – (±)-F-flurbiprofen – and its translation to radiofluorination with [^18^F]TEAF. The generality of our methodology and synthetic applications, based on a Rh-catalyzed carbyne transfer with alkenes, stands as a testament of its potential utility to expand the chemical space in fluorine-based drug design.

## Data availability

The data for this work, including optimization tables, general experimental procedures, characterization data for all new compounds and X-ray data are provided in the ESI.†

## Author contributions

L. J. discovered the fluorination reaction. L. J., P. S. & W. J. T. performed the experiments, and all authors contributed to the analysis and interpretation of the data. P. S. carried out the radiofluorination under the supervision and guidance of J. L. M. G. S. directed the project and wrote the manuscript with contributions from all authors.

## Conflicts of interest

There are no conflicts to declare.

## Supplementary Material

SC-013-D2SC00968D-s001

SC-013-D2SC00968D-s002
