# Supplementary material for: Catalytic alkene skeletal modification for the construction of fluorinated tertiary stereocenters
Source: Chem Sci. 2022 Mar 15;13(15):4327–33. doi: 10.1039/d2sc00968d (PMC9006967; doi:10.1039/d2sc00968d)
Supplement: SC-013-D2SC00968D-s001 [file SC-013-D2SC00968D-s001.pdf]

Electronic Supplementary Material (ESI) for Chemical Science.
This journal is © The Royal Society of Chemistry 2022

Catalytic alkene skeletal modification for the construction of fluorinated tertiary stereocenters

Liyin Jiang,[a] Pau Sarro,[a,b] Wei Jie Teo,[a] Jordi Llop,[c] and Marcos G. Suero*[a]

[a] Institute of Chemical Research of Catalonia (ICIQ), The Barcelona Institute of Science and Technology, Països Catalans 16, 43007 Tarragona (Spain)

[b] Departament de Química Analítica i Química Orgànica, Universitat Rovira i Virgili, C. Marcel·lí Domingo, 1, 43007 Tarragona (Spain)

[c] CIC biomaGUNE, Basque Research and Technology Alliance, Paseo Miramon 182, 20014, San Sebastian (Spain)

Electronic Supplementary Information

Table of Contents

1. General information. 3

2. Synthesis of alkenes 1...4

3. Synthesis of hypervalent iodine reagents 2..10

4. Optimization studies. 10

5. Synthesis of tertiary allylic fluorides 3...12

6. Derivatizations of (±)-3a. 35

7. Synthesis of F-flurbiprofen 12. 40

8. Radiofluorination of styrenes 1a and 1i. 41

9. Fluorination of styrene and β-methylstyrene. 44

10. References. 46

11. NMR Spectra. 50

1. General information.

All reagents were directly used as purchased without any further purification. Ethyl diazoacetate, (contains ≥13 wt. % dichloromethane) was purchased from Sigma-Aldrich (Ref. E22201). Anhydrous solvents were dried by passing them through an activated alumina column on a PureSolvTM solvent purification system (Innovative Technologies, Inc., MA). Analytical thin layer chromatography (TLC) was carried out using aluminum sheets with 0.2 mm of silica gel (Merck GF234). Visualization of the developed chromatogram was performed by irradiation with UV light. Flash column chromatography was performed on silica gel (Aldrich, 230-400 mesh) or neutral silica gel (Material Harvest Ltd., 230-400 mesh). Preparative thin layer chromatography (PLC) was carried out using glass plate with 0.5 mm of silica gel 60. Organic solutions were concentrated under *vacuum* on a Büchi rotatory evaporator. Unless otherwise stated, reactions were carried out under argon atmosphere. NMR spectra were recorded at 298 K on Bruker Avance 300, Bruker Avance 400 Ultrashield and Bruker Avance 500 Ultrashield apparatuses. Chemical shifts (δ) are quoted in ppm relative to residual solvent signals, $CDCl_3$ referenced at δ 7.26 and 77.16 ppm, CD_3CN referenced at δ 1.94 and 1.39, 118.69 ppm respectively, Acetone-D_6 referenced at 2.05 and 29.8 ppm, CD_2Cl_2 referenced at δ 5.32 and 53.84 ppm. Coupling constants (*J*) are quoted in hertz (Hz). Multiplicity is reported with the following abbreviations: s = singlet, brs = broad singlet, d = doublet, t = triplet, q = quartet, dt = doublet of triplets, td = triplet of doublets, tt = triplet of triplets, tq = triplet of quartets, qd = quartet of doublets, qt = quartet of triplets, ddd = doublet of doublets of doublets, sp = septet, m = multiplet, app = apparent. Melting points were measured using open glass capillaries in a Büchi B540 apparatus. Infrared spectra were recorded on a Bruker Tensor 27. Mass spectra were recorded on a Waters LCT Premier spectrometer. Specific optical rotation measurements were carried out on a Jasco P-1030 model polarimeter equipped with a PMT detector using the Sodium line at 589 nm using a 100 mm pathlength cell and are reported as: $[\alpha]_D^T$ (concentration in 10 mg/1 mL, solvent).

2. Synthesis of alkenes 1.

1a, 1b, 1f, 1l, 1ab,1ac, 1ad, 1ae, 1ah, 1am, 1al were commercially available and used directly as received. **1c**[1], **1d**[1], **1e**[2], **1g**[3], **1h**[4], **1i**[2], **1n**[3], **1o**[3], **1p**[5], **1q**[2], **1r**[3], **1s**[1], **1t**[6], **1u**[6], **1v**[7], **1w**[8], **1z**[1] were prepared according to the general procedure A[5] from the corresponding commercial ketone compounds. **1x**[9], **1y**[10], **1af**[11], **1ag**[11] were synthesized by following reported protocols.

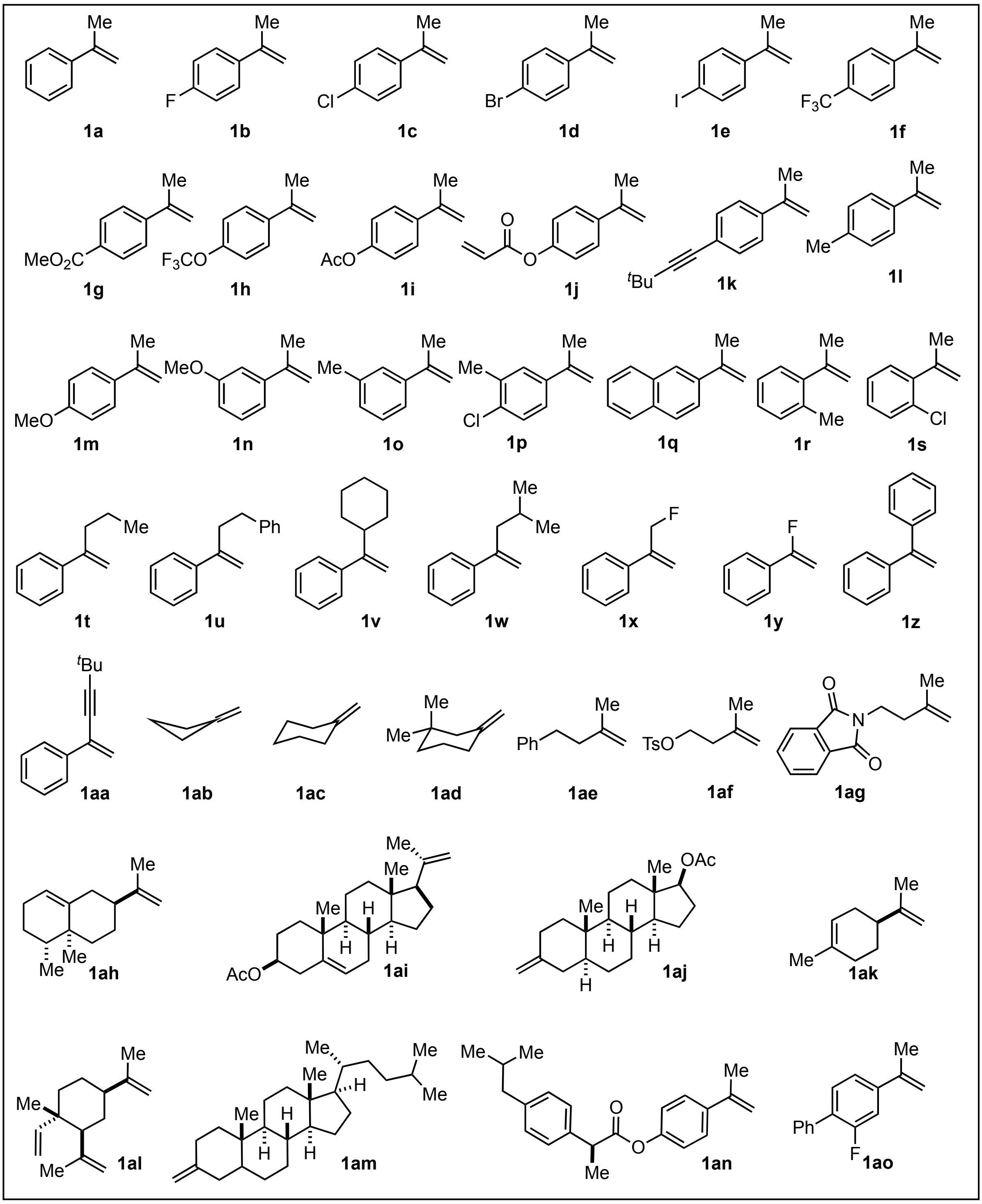

General procedure A:[1,2] To an oven-dried flask was charged methyltriphenylphosphonium bromide (5.3 g, 15 mmol), potassium *tert*-butoxide (1.9 g, 16 mmol) and THF (15 mL). The reaction mixture was stirred at room temperature for 1 hour. After this, the mixture was cooled to 0 °C and the corresponding ketone (12 mmol, 1.0 equiv.) was added dropwise. The reaction mixture was allowed to reach room temperature and stirred overnight. Then, the reaction was quenched with a saturated aqueous solution of NH_4Cl (40 mL) and extracted with diethyl ether (3 x 20 mL). The combined organic layers were washed with brine and dried over Na_2SO_4. Removal of solvent under *vacuum* and purification by flash column chromatography with hexane/ethyl acetate mixtures afforded the desired alkenes **1**.

4-(Prop-1-en-2-yl)phenyl acrylate (1j)

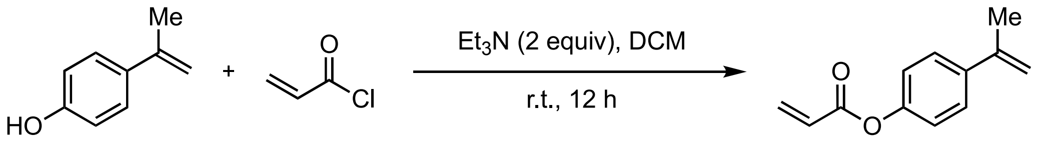

1j

To an oven-dried flask was charged 4-(prop-1-en-2-yl)phenol (550 mg, 4.1 mmol), Et_3N (0.6 mL, 8.2 mmol) and dichloromethane (10 mL). The mixture was cooled at 0 °C and acryloyl chloride (0.65 mL, 8.2 mmol) was added dropwise. After this, the reaction mixture was warmed to room temperature and stirred for 12 hours. The reaction was quenched with a saturated aqueous solution of $NaHCO_3$ (25 mL) and extracted with ethyl acetate (3 x 15 mL). The combined organic layers were washed with brine and dried over Na_2SO_4. Removal of solvent under *vacuum* and purification by flash column chromatography (hexane/ethyl acetate = 10/1) provided product **1j** as colorless oil (520 mg, 68% yield).

^{1}H NMR (300 MHz, $CDCl_3$) δ 7.49 (d, *J* = 8.7 Hz, 2H), 7.16 – 7.06 (m, 2H), 6.62 (dd, *J* = 17.3, 1.4 Hz, 1H), 6.33 (dd, *J* = 17.3, 10.4 Hz, 1H), 6.02 (dd, *J* = 10.4, 1.3 Hz, 1H), 5.37 – 5.34 (m, 1H), 5.13 – 5.05 (m, 1H), 2.15 (dd, *J* = 1.5, 0.8 Hz, 3H); **^{13}C NMR** (75 MHz, $CDCl_3$) δ 164.7, 150.0, 142.6, 139.2, 132.7, 128.1, 126.7, 121.3, 112.8, 22.0.

HRMS (ESI): calculated for $C_{12}H_{12}O_2Na^+$ $[M+Na]^+$ m/z: 211.0730, found: 211.0728.

1-(3,3-Dimethylbut-1-yn-1-yl)-4-(prop-1-en-2-yl)benzene (1k)

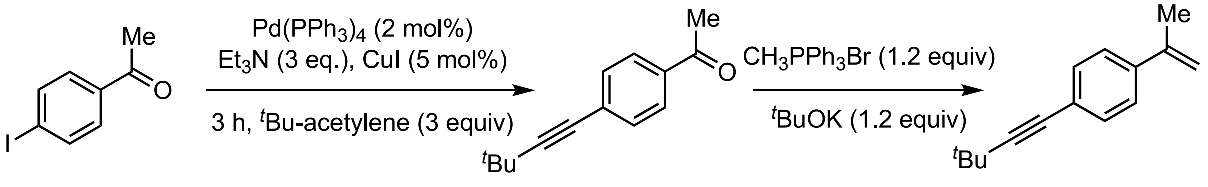

Compound **1k** was prepared according to the general procedure A using ketone **A** (1.5 g, 7.5 mmol, *prepared following a reported protocol*)[12] and obtained as a colorless oil (1.4 g, 93% yield).

^{1}H NMR (300 MHz, $CDCl_3$) δ 7.44 – 7.31 (m, 4H), 5.41 – 5.38 (m, 1H), 5.12 – 5.09 (m, 1H), 2.14 (dd, J = 1.5, 0.8 Hz, 3H), 1.34 (s, 9H); **^{13}C NMR** (75 MHz, $CDCl_3$) δ 142.8, 140.2, 131.5, 125.3, 123.2, 112.8, 99.2, 79.1, 31.2, 28.1, 21.8.

HRMS (APCI): calculated for $C_{15}H_{19}$ $[M]^+$ m/z: 199.1841, found: 199.1843.

(5,5-Dimethylhex-1-en-3-yn-2-yl)benzene (1aa)

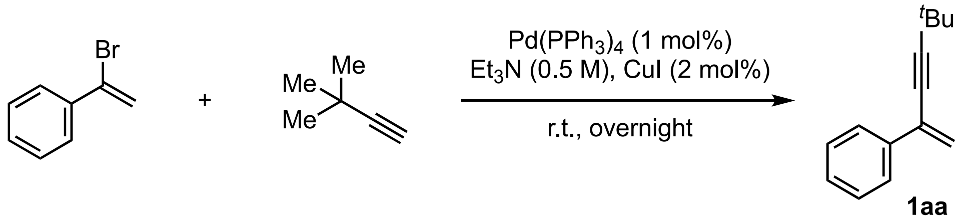

To a solution of (1-bromovinyl)benzene (1.8 g, 10 mmol) in triethylamine (20 mL, 0.5M) were added $Pd(PPh_3)_4$ (108 mg, 0.1 mmol, 1 mol%) and CuI (38 mg, 0.2 mmol). After this, 3,3-dimethylbut-1-yne (0.52 g, 25 mmol, 2.5 equiv.) was added and the reaction mixture was stirred overnight. The reaction was quenched with a saturated aqueous solution of NH_4Cl (40 mL) and extracted with diethyl ether (3 x 20 mL). The combined organic layers were washed with brine and dried over Na_2SO_4. Removal of solvent under *vacuum* and purification by flash column chromatography (hexane) provided product **1aa** as colorless oil (1.5 g, 83% yield).

^{1}H NMR (500 MHz, $CDCl_3$) δ 7.73 – 7.57 (m, 2H), 7.39 – 7.32 (m, 2H), 7.32 – 7.27 (m, 1H), 5.83 (d, J = 1.2 Hz, 1H), 5.57 (d, J = 1.2 Hz, 1H), 1.33 (s, 9H); **^{13}C NMR** (126 MHz, $CDCl_3$) δ 138.1, 131.0, 128.4, 128.2, 126.2, 119.2, 100.3, 78.4, 31.2, 28.2.

HRMS (APCI): calculated for $C_{14}H_{17}^+$ $[M+H]^+$ m/z: 185.1325, found: 185.1321.

(3*S*,8*S*,9*S*,10*R*,13*S*,14*S*,17*R*)-10,13-Dimethyl-17-(prop-1-en-2-yl)-2,3,4,7,8,9,10,11,12,13,14,15,16,17-tetradecahydro-1H-cyclopenta[a]phenanthren-3-yl acetate (1ai)

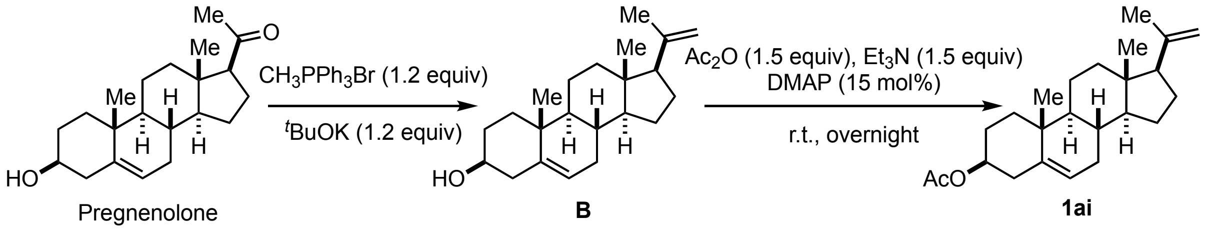

To an oven-dried flask was charged compound **B** (2.7 g, 8.7 mmol, *prepared following the general procedure A*),[13] *N,N*-dimethyl-4-aminopyridine (160 mg, 1.3 mmol), Et_3N (1.8 mL, 13 mmol) and dichloromethane (25 mL). The reaction was cooled to 0 °C and acetic anhydride (1.3 mL, 13 mmol) was added dropwise. The reaction mixture was allowed to reach room temperature and stirred overnight. After this, the reaction was quenched with a saturated aqueous solution of $NaHCO_3$ (20 mL) and extracted with ethyl acetate (3 x 25 mL). The combined organic layers were washed with brine and dried over Na_2SO_4. Removal of solvent under *vacuum* and purification by flash column chromatography (hexane/ethyl acetate = 10/1) provided product **1ai** as colorless oil (1.8 g, 58% yield).

^{1}H NMR (400 MHz, $CDCl_3$) δ 5.39 – 5.36 (m, 1H), 4.94 – 4.82 (m, 1H), 4.73 – 4.67 (m, 1H), 4.66 – 4.52 (m, 1H), 2.43 – 2.24 (m, 2H), 2.09 – 1.93 (m, 5H), 1.91 – 1.81 (m, 3H), 1.80 – 1.72 (m, 4H), 1.72 – 1.62 (m, 2H), 1.62 – 1.51 (m, 3H), 1.50 – 1.38 (m, 2H), 1.29 – 1.05 (m, 4H), 1.02 (s, 3H), 1.01 – 0.92 (m, 1H), 0.58 (s, 3H); **^{13}C NMR** (101 MHz, $CDCl_3$) δ 170.6, 145.7, 139.8, 122.6, 110.8, 74.1, 57.4, 56.6, 50.3, 43.2, 38.8, 38.2, 37.2, 36.8, 32.3, 31.9, 27.9, 25.5, 24.8, 24.4, 21.6, 21.2, 19.5, 12.8.

HRMS (ESI): calculated for $C_{24}H_{36}O_2Na^+$ $[M+Na]^+$ m/z: 379.2608, found: 379.2603.

Spectra were consistent with the previously reported.[14]

(5*S*,8*R*,10*S*,13*S*,14*S*,17*S*)-10,13-Dimethyl-3-methylenehexadecahydro-1H-cyclopenta[a]phenanthren-17-yl acetate (1aj)

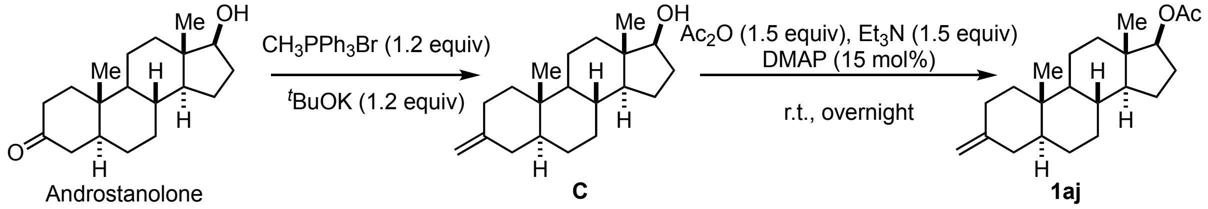

To a 50 mL oven dried flask was charged **C** (0.92 g, 3.1 mmol, *prepared following the general procedure A*),[15] *N,N*-dimethylpyridin-4-amine (38 mg, 0.31mmol), Et_3N (0.6 mL, 4.3 mmol)

and dichloromethane (25 mL). The reaction was cooled to 0 °C and acetic anhydride (0.45 mL, 4.3 mmol) was added dropwise. The reaction mixture was allowed to reach room temperature and stirred overnight. Then, the reaction was quenched with a saturated aqueous solution of $NaHCO_3$ (20 mL) and extracted with ethyl acetate (3 x 25 mL). The combined organic layers were washed with brine and dried over Na_2SO_4. Removal of solvent under *vacuum* and purification by flash column chromatography (hexane/ethyl acetate = 10/1) provided **1aj** as white solid (780 mg, 71% yield).

^{1}H NMR (400 MHz, $CDCl_3$) δ 4.62 – 4.50 (m, 3H), 2.24 – 2.06 (m, 3H), 2.03 (s, 3H), 2.02 – 1.86 (m, 2H), 1.81 – 1.74 (m, 2H), 1.68 – 1.35 (m, 5H), 1.35 – 1.20 (m, 4H), 1.09 – 0.99 (m, 2H), 0.99 – 0.92 (m, 2H), 0.92 – 0.80 (m, 4H), 0.78 (d, *J* = 0.6 Hz, 3H), 0.66 (ddd, *J* = 12.2, 10.5, 4.2 Hz, 1H); **^{13}C NMR** (101 MHz, $CDCl_3$) δ 171.4, 150.0, 106.3, 83.0, 54.5, 50.9, 48.2, 42.7, 40.0, 38.0, 37.1, 36.2, 35.4, 31.6, 31.1, 28.8, 27.7, 23.7, 21.3, 20.7, 12.3, 11.9.

HRMS (ESI): calculated for $C_{22}H_{34}O_2Na^+$ $[M+Na]^+$ m/z: 353.2451, found: 353.2461.

(8*R*,10*S*,13*R*,17*R*)-10,13-Dimethyl-3-methylene-17-((*R*)-6-methylheptan-2-yl)hexadecahydro-1H-cyclopenta[a]phenanthrene (1am)

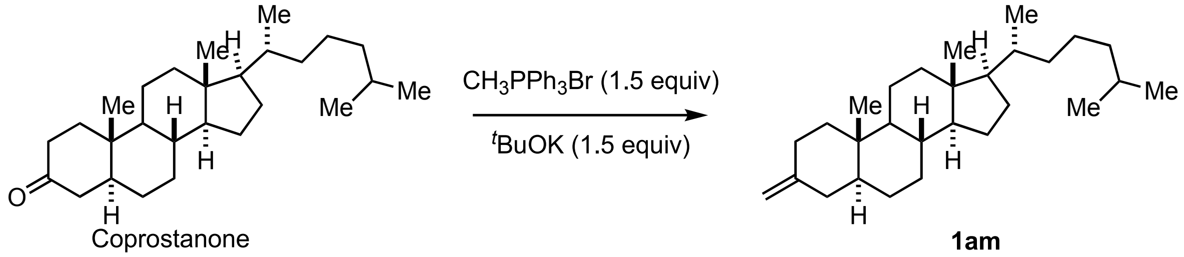

Compound **1am** was prepared according to the general procedure A using Coprostanone (600 mg, 1.6 mmol) and obtained as white solid (560 mg, 93% yield).

^{1}H NMR (400 MHz, $CDCl_3$) δ 4.60 – 4.51 (m, 2H), 2.19 – 2.14 (m, 2H), 2.07 – 1.93 (m, 2H), 1.91 – 1.86 (m, 1H), 1.86 – 1.74 (m, 2H), 1.65 – 1.45 (m, 3H), 1.60 – 1.55 (m, 1H), 1.53 – 1.45 (m, 2H), 1.45 – 1.20 (m, 8H), 1.19 – 1.06 (m, 6H), 1.06 – 0.93 (m, 4H), 0.90 (d, *J* = 6.5 Hz, 3H), 0.87 (d, *J* = 1.8 Hz, 3H), 0.86 (d, *J* = 1.8 Hz, 6H), 0.66 (s, 3H); **^{13}C NMR** (101 MHz, $CDCl_3$) δ 150.4, 106.1, 56.7, 56.5, 54.6, 48.3, 42.7, 40.2, 40.0, 39.7, 38.1, 36.3, 36.2, 36.0, 35.7, 32.2, 31.2, 29.1, 28.4, 28.2, 24.4, 24.0, 23.0, 22.7, 21.3, 18.8, 12.2, 11.9.

HRMS (APCI): calculated for $C_{28}H_{49}^+$ $[M+H]^+$ m/z: 385.3829, found: 385.3832.

4-(Prop-1-en-2-yl)phenyl (*S*)-2-(4-isobutylphenyl)propanoate (1an)

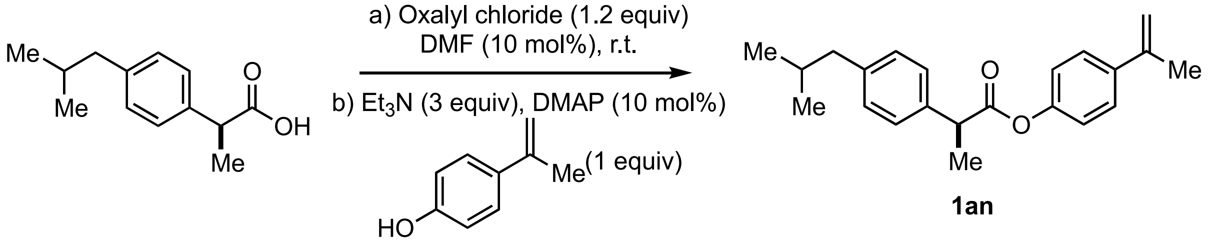

To an oven-dried flask was charged ibuprofen (1.5 g, 7.3 mmol), DMF (55 μl, 10 mol%) and dichloromethane (15 mL). Then, the reaction mixture was cooled to 0 °C and oxalyl chloride (0.7 mL, 8.0 mmol) was added dropwise. The reaction mixture was allowed to reach room temperature and stirred overnight. After this, the solvent was removed under *vacuum* and the crude was re-dissolved in dichloromethane (5 mL). Then, this solution was added dropwise to a second oven-dried flask charged with 4-(prop-1-en-2-yl)phenol (800 mg, 6.0 mmol), *N*,*N*-dimethylpyridin-4-amine (73 mg, 10 mol%), Et_3N (2.5 mL, 18.0 mmol) and dichloromethane (15 mL). The resulting reaction mixture was stirred overnight at room temperature and then quenched with a saturated aqueous solution of $NaHCO_3$ (20 mL) and extracted with CH_2Cl_2 (3 x 15 mL). The combined organic layers were washed with brine and dried over Na_2SO_4. Removal of solvent and purification by flash column chromatography (hexane/ethyl acetate = 10/1) provided **1an** as colorless oil (1.2 g, 54% yield).

^{1}H NMR (400 MHz, $CDCl_3$) δ 7.48 – 7.37 (m, 2H), 7.35 – 7.27 (m, 2H), 7.17 – 7.11 (m, 2H), 7.01 – 6.90 (m, 2H), 5.34 – 5.28 (m, 1H), 5.09 – 5.02 (m, 1H), 3.93 (q, *J* = 7.1 Hz, 1H) 2.47 (d, *J* = 7.3 Hz, 2H), 2.12 (s, 3H), 1.87 (sp, *J* = 6.8 Hz, 1H), 1.61 (d, *J* = 7.2 Hz, 3H), 0.91 (d, *J* = 6.8 Hz, 6H); **^{13}C NMR** (101 MHz, $CDCl_3$) δ 173.4, 150.4, 142.6, 141.0, 139.0, 137.4, 129.7, 127.4, 126.6, 121.2, 112.7, 77.5, 77.2, 76.8, 45.4, 45.2, 30.3, 22.6, 22.0, 18.7.

HRMS (ESI): calculated for $C_{22}H_{27}O_2{}^+$ $[M+H]^+$ m/z: 323.2006, found: 323.1997.

2-Fluoro-4-(prop-1-en-2-yl)-1,1'-biphenyl (1ao)

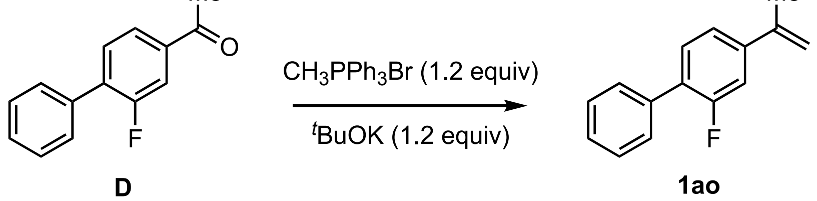

Compound **1ao** was prepared according to the general procedure A using ketone **D** (2.1 g, 9.7 mmols, *prepared following a reported protocol*)[16] and obtained as a white solid (1.8 g, 87% yield).

^{1}H NMR (400 MHz, $CDCl_3$) δ 7.61 – 7.54 (m, 2H), 7.50 – 7.42 (m, 2H), 7.42 – 7.38 (m, 1H), 7.38 – 7.31 (m, 2H), 7.30 – 7.22 (m, 1H), 5.46 (q, J = 1.0 Hz, 1H), 5.19 – 5.14 (m, 1H), 2.18 (s, 3H); **^{13}C NMR** (101 MHz, $CDCl_3$) δ 159.8 (d, 248.1 Hz), 142.6 (d, J = 7.7 Hz), 141.9 (d, J = 2.1 Hz), 135.8 (d, J = 1.4 Hz), 130.5 (d, J = 4.1 Hz), 129.1 (d, J = 6.0 Hz), 128.6, 128.0 (d, J = 13.8 Hz), 127.8, 121.5 (d, J = 3.2 Hz), 113.6, 113.3 (d, J = 23.8 Hz), 21.8; **^{19}F NMR** (376 MHz, $CDCl_3$) δ -118.56 (dd, J = 12.5, 8.2 Hz).

HRMS (APCI): calculated for $C_{15}H_{14}F$ $[M+H]^+$ m/z: 213.1074, found: 213.1081.

3. Synthesis of hypervalent iodine reagents 2.

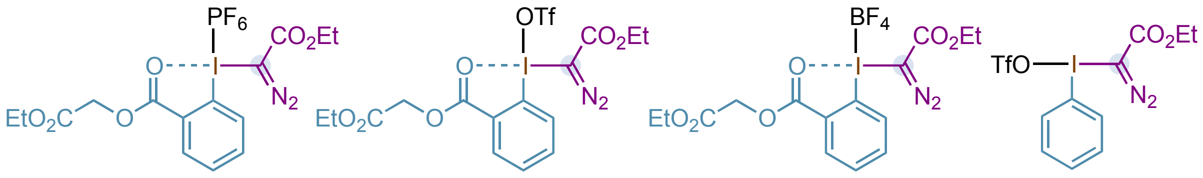

2a **2a-OTf** **2a-BF$_4$** **2b**

The hypervalent iodine reagents **2** indicated above are known compounds and were prepared following reported protocols.[17, 18] **2a** was prepared by the following modification of a reported protocol:[17] a solution of **2a-OTf** (3.0 g, 5.1 mmol) in dichloromethane (25 mL, 0.2 M) was added to a 100 mL separation funnel and then washed with a saturated aqueous solution of KPF_6 (3 x 25 mL). The combined organic layers were dried over Na_2SO_4 and solvent was removed under *vacuum* to give **2a** as yellow solid (2.7 g, 89 % yield). Spectra were consistent with the previously reported.[17]

(*Note: if the product contains impurities, it is recrystallized with dichloromethane/diethyl ether = 1/5*).

4. Optimization studies.

General procedure B: To a 10 mL oven-dried tube was added the corresponding Rh catalyst (0.001 mmol, 1 mol%). The tube was sealed before being evacuated and backfilled with argon three times. α-methylstyrene **1a** (24 mg, 27 µL, 0.2 mmol) and degassed dichloromethane (0.5 mL) were added and the resulting mixture was cooled at -50 ºC. Then, a solution of reagent **2** (0.1 mmol, 1.0 equiv.) in degassed dichloromethane (1 mL) was added dropwise during 1 h using a syringe pump. After this, the reaction mixture was stirred at -50 ºC for 30 minutes. Next, fluoride nucleophile was added and the resulting reaction mixture was allowed to warm to room temperature during 3 hours. The resulting reaction mixture was analyzed by ^{1}H-NMR and ^{19}F-NMR using anisole (10.8 mg, 0.1 mmol, 1.0 equiv.) as internal standard.

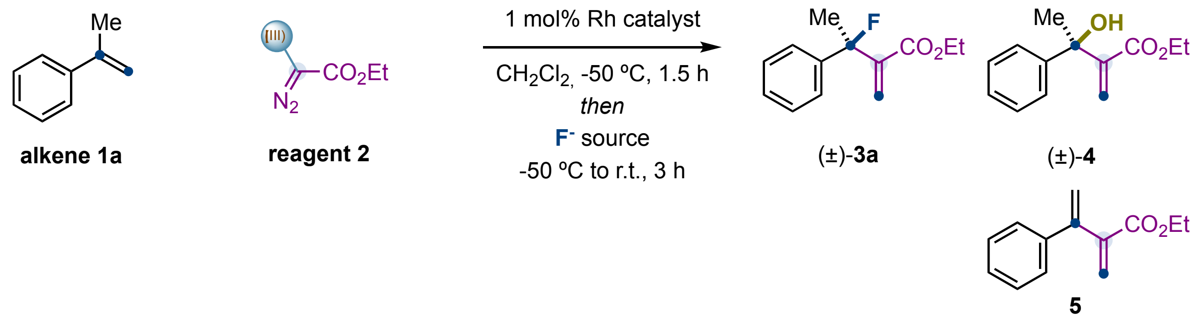

Entry	Catalyst	Reagent 2	F^- source (equiv.)	(±)-**3**[a]	*b*:*l*[b]	(±)-**4**:**5**[a]
1	$Rh_2(OAc)_4$	**2a**	$Et_3N\cdot 3HF$ (3)	47%	> 20:1	3%:nd
2	$Rh_2(Adc)_4$	**2a**	$Et_3N\cdot 3HF$ (3)	65%	> 20:1	2%:3%
3	$Rh_2(TPA)_4$	**2a**	$Et_3N\cdot 3HF$ (3)	29%	> 20:1	2%:2%
4	$Rh_2(esp)_2$	**2a-OTf**	$Et_3N\cdot 3HF$ (3)	65%	> 20:1	3%:7%
5	$Rh_2(esp)_2$	**2a-BF$_4$**	$Et_3N\cdot 3HF$ (3)	68%	> 20:1	4%:3%
6	$Rh_2(esp)_2$	**2a**	$Et_3N\cdot 3HF$ (3)	73%	> 20:1	<3%:<2%
7	$Rh_2(esp)_2$	**2b**	$Et_3N\cdot 3HF$ (3)	45%	> 20:1	2%:8%
8	$Rh_2(esp)_2$	**2a**	$Et_3N\cdot 3HF$ (1)	10%	> 20:1	45%:8%
9	$Rh_2(esp)_2$	**2a**	$Et_3N\cdot 3HF$ (3)[c]	nd	-	66%:4%
10	$Rh_2(esp)_2$	**2a**	TBAF·H_2O (3)	28%	> 20:1	5%:29%
11	$Rh_2(esp)_2$	**2a**	TBAF·$(pin)_2$ (3)	< 5%	-	nd:12%
13	$Rh_2(esp)_2$	**2a**	TBAT (3)	20%	> 20:1	nd:20%
14	$Rh_2(esp)_2$	**2a**	Py(HF)x (3)	nd	-	nd:15%
15	$Rh_2(esp)_2$	**2a**	NaF (3)	nd	-	1%:5%
16	$Rh_2(esp)_2$	**2a**	KF (3)	nd	-	1%:6%
17	$Rh_2(esp)_2$	**2a**	CsF (3)	nd	-	1%:26%

[a]Yields are reported on the basis of ^{1}H-NMR analysis using anisole as internal standard. [b]Branched/linear ratio was determined by ^{19}F-NMR analysis of crude reaction mixture. [c]Water (10 equiv.) was added together with the fluoride source. nd refers to not detected. TBAF refers to tetrabutylammonium fluoride. TBAT refers to tetrabutylammonium difluorotriphenylsilicate. Py(HF)x refers to Olah's reagent.

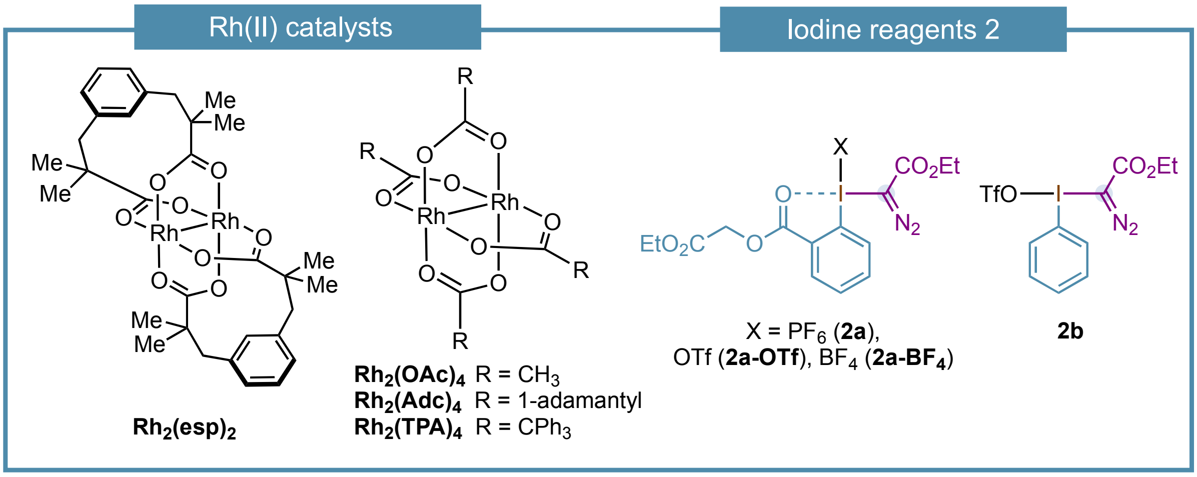

Ethyl 3-hydroxy-2-methylene-3-phenylbutanoate ((±)-4)

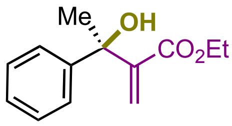

Prepared according to general procedure C using α-methylstyrene **1a** (52 µL, 0.4 mmol, 0.2 equiv.), reagent **2a** (120 mg, 0.2 mmol) and H_2O (20 equiv, 72 mg, 72 µL) instead of TBAF·3H_2O as nucleophile (74% NMR yield and ratio of *branched*:*linear* isomers > 20:1 by ^{1}H-NMR from the crude reaction mixture using trimethoxybenzene as internal standard). The desired product was not separable from the reaction byproduct 2-ethoxy-2-oxoethyl 2-iodobenzoate and was characterized together with it.[19]

^{1}H NMR (400 MHz, $CDCl_3$) δ 7.35 – 7.28 (m, 2H), 7.26 – 7.21 (m, 1H), 7.22 – 7.09 (m, 2H), 6.40 (s, 1H), 5.96 (s, 1H), 4.20 – 4.03 (m, 2H), 1.67 (s, 3H), 1.19 (td, *J* = 7.1, 0.5 Hz, 3H); **^{13}C NMR** (101 MHz, $CDCl_3$) 167.5, 147.0, 144.7, 128.3, 127.0, 124.9, 124.7, 94.6, 61.2, 29.4, 14.1.

Spectra are consistent with previously reported.[20]

Ethyl 2-methylene-3-phenylbut-3-enoate (5)

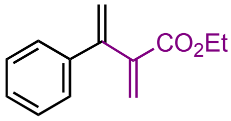

^{1}H NMR (400 MHz, $CDCl_3$) δ 7.35 – 7.27 (m, 5H), 6.31 (d, *J* = 1.7 Hz, 1H), 5.81 (d, *J* = 1.7 Hz, 1H), 5.50 (d, *J* = 1.4 Hz, 1H), 5.40 (d, *J* = 1.4 Hz, 1H), 4.10 (q, *J* = 7.1 Hz, 2H), 1.09 (t, *J* = 7.1 Hz, 3H); **^{13}C NMR** (101 MHz, $CDCl_3$) δ 166.7, 146.4, 142.5, 140.0, 128.4, 127.9, 127.6, 126.7, 116.4, 61.0, 14.0.

HRMS (ESI) calculated for $C_{13}H_{14}NaO_2^+$ $[M+Na]^+$ m/z: 225.0886, found: 225.0884.

^{1}H-^{1}H COSY, ^{1}H-^{13}C HSQC, ^{1}H-^{13}C HMBC spectra were measured.

5. Synthesis of tertiary allylic fluorides 3.

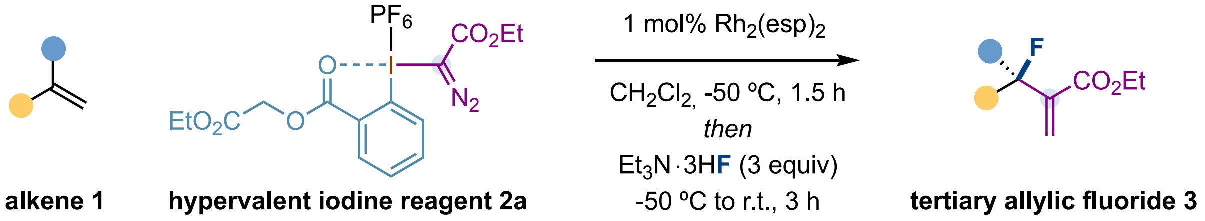

General procedure C: To a 10 mL oven-dried tube was added $Rh_2(esp)_2$ (2.0 mg, 0.002 mmol, 1 mol%). The tube was sealed before being evacuated and backfilled with argon three times. The corresponding alkene **1** (0.4 mmol, 2.0 equiv.) and degassed dichloromethane (1.0 mL) were added and the resulting mixture was cooled at -50 ºC. Then, a solution of reagent **2a** (0.2 mmol, 1.0 equiv.) in degassed dichloromethane (2.0 mL) was added dropwise during 1 h using

a syringe pump. After addition, the reaction mixture was stirred for 30 min at -50 ºC. Next, Et_3N•3HF (3.0 equiv., 0.1 mL, 96 mg) was added, and the resulting reaction mixture was allowed to warm to room temperature during 3 hours. The resulting reaction mixture was filtered through a short plug of silica gel and washed with dichloromethane. Solvent was removed under *vacuum* and the resulting crude residue was analyzed by ^{19}F-NMR spectroscopy for the determination of the *branched:linear* ratio. Purification by flash chromatography on silica gel (hexane & ethyl acetate mixtures) provided the corresponding tertiary allylic fluoride compounds **3**.

Ethyl 3-fluoro-2-methylene-3-phenylbutanoate ((±)-3a)

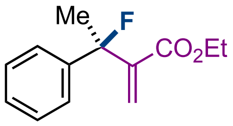

Prepared according to general procedure C using α-methylstyrene **1a** (52 µL, 0.4 mmol, 0.2 equiv.), reagent **2a** (120 mg, 0.2 mmol) and Et_3N•3HF (96 mg, 0.6 mmol). Ratio of *branched*:*linear* isomers was determined to be > 20:1 from the crude reaction mixture using ^{19}F-NMR spectroscopy. Purification by flash chromatography on silica gel (hexane/ethyl acetate = 40/1) provided the title compound as colorless oil (32 mg, 73% yield).

^{1}H NMR (500 MHz, $CDCl_3$) δ 7.46 – 7.42 (m, 2H), 7.38 – 7.32 (m, 2H), 7.32 – 7.27 (m, 1H), 6.38 (dd, *J* = 4.3, 1.0 Hz, 1H), 6.12 (d, *J* = 1.0 Hz, 1H), 4.14 – 4.00 (m, 2H), 2.02 (d, *J* = 23.5 Hz, 3H), 1.14 (t, *J* = 7.1 Hz, 3H); **^{13}C NMR** (126 MHz, $CDCl_3$) δ 164.9 (d, *J* = 6.3 Hz), 144.0 (d, *J* = 25.2 Hz), 142.0 (d, *J* = 22.8 Hz), 128.2, 128.1 (d, *J* = 2.5Hz), 125.7 (d, *J* = 6.3 Hz), 124.8 (d, = 11.3 Hz), 95.9 (d, *J* = 173.9 Hz), 60.8, 25.8, (d, *J* = 23.9 Hz), 14.0; **^{19}F NMR** (376 MHz, $CDCl_3$) δ -132.0 (qd, *J* = 23.5, 4.3 Hz).

HRMS (ESI): calculated for $C_{13}H_{15}O_2FNa^+$ $[M+Na]^+$ m/z: 245.0948, found: 245.0948.

Note: we have observed that this particular product is unstable in solution.

Gram-scale reaction: To a 100 mL oven-dried round bottom flask was added $Rh_2(esp)_2$ (50.0 mg, 0.07 mmol, 1.0 mol%). The tube was sealed before being evacuated and backfilled with argon three times. α-methyl styrene **1a** (14.2 mmol, 1.67 g, 2.0 equiv.) and degassed dichloromethane (10 mL) were added and the resulting mixture was cooled at -50 ºC. Then, a solution of reagent **2a** (4.2 g, 7.1 mmol, 1.0 equiv.) in degassed dichloromethane (50 mL) was added dropwise during 1 h using a syringe pump. After addition, the reaction mixture was stirred for 30 min at -50 ºC. Next, Et_3N•3HF (3.4 g, 3.0 equiv.) was added and the resulting reaction mixture was allowed to warm to room temperature during 3 hours. The resulting

reaction mixture was quenched with aqueous solution of NH_3 (10%, 50 mL) and extracted with dichloromethane (3 x 30 mL). The combined organic layers were washed with brine and dried over Na_2SO_4. Removal of solvent under *vacuum* and purification by flash column chromatography provided (±)-**3a** as colorless oil (1.3 g, 82% yield, *b*:*l* > 20:1).

Ethyl 3-fluoro-3-(4-fluorophenyl)-2-methylenebutanoate ((±)-3b)

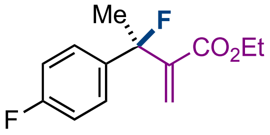

Prepared according to general procedure C using alkene **1b** (52 mg, 0.4 mmol), reagent **2a** (120 mg, 0.2 mmol) and Et_3N•3HF (96 mg, 0.6 mmol). Ratio of *branched*:*linear* isomers was determined to be > 20:1 from the crude reaction mixture using ^{19}F-NMR spectroscopy. Purification by flash chromatography on silica gel (hexane/ethyl acetate = 40/1) provided the title compound as colorless oil (41 mg, 85% yield).

^{1}H NMR (500 MHz, $CDCl_3$) δ 7.43 – 7.40 (m, 2H), 7.03 – 7.01 (m, 2H), 6.38 (dd, *J* = 4.5, 1.0 Hz, 1H), 6.13 (d, *J* = 1.0 Hz, 1H), 4.11 – 4.01 (m, 2H), 2.01 (d, *J* = 23.6 Hz, 3H), 1.16 (t, *J* = 7.1 Hz, 3H); **^{13}C NMR** (126 MHz, $CDCl_3$) δ 164.8 (d, *J* = 6.6 Hz), 162.6 (dd, *J* = 247.4, 2.7 Hz), 143.8 (d, *J* = 24.4 Hz), 137.9 (dd, *J* = 22.2, 3.2 Hz), 127.7 (dd, *J* = 8.3, 6.2 Hz), 124.8 (d, *J* = 12.0 Hz), 115.1 (d, *J* = 21.6 Hz), 95.5 (d, *J* = 174.2 Hz), 60.9, 25.8 (d, *J* = 24.8 Hz), 14.1; **^{19}F NMR** (376 MHz, $CDCl_3$) δ -114.5 (m, 1H), -130.4 (m, 1H).

HRMS (ESI): calculated for $C_{13}H_{14}O_2F_2Na^+$ $[M+Na]^+$ m/z: 263.0854, found: 263.0849.

Ethyl 3-(4-chlorophenyl)-3-fluoro-2-methylenebutanoate ((±)-3c)

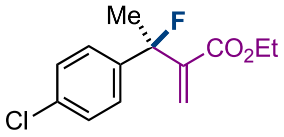

Prepared according to general procedure C using alkene **1c** (60 mg, 0.4 mmol), reagent **2a** (120 mg, 0.2 mmol) and Et_3N•3HF (96 mg, 0.6 mmol). Ratio of *branched*:*linear* isomers was determined to be > 20:1 from the crude reaction mixture using ^{19}F-NMR spectroscopy. Purification by flash chromatography on silica gel (hexane/ethyl acetate = 20/1) provided the title compound as colorless oil (45 mg, 87% yield).

^{1}H NMR (400 MHz, $CDCl_3$) δ 7.41 – 7.35 (m, 2H), 7.35 – 7.28 (m, 2H), 6.39 (dd, *J* = 4.5, 0.9 Hz, 1H), 6.14 (d, *J* = 0.9 Hz, 1H), 4.07 (qd, *J* = 7.1, 3.2 Hz, 2H), 1.99 (d, *J* = 23.5 Hz, 3H), 1.17 (t, *J* = 7.1 Hz, 3H); **^{13}C NMR** (101 MHz, $CDCl_3$) δ 164.6 (d, *J* = 6.4 Hz), 143.5 (d, *J* = 24.2 Hz), 140.6 (d, *J* = 22.3 Hz), 134.0 (d, *J* = 2.8 Hz), 128.3, 127.2 (d, *J* = 6.6 Hz), 125.0 (d, *J* = 12.0 Hz), 95.4 (d, *J* = 174.8 Hz), 60.9, 25.7 (d, *J* = 24.7 Hz), 14.0; **^{19}F NMR** (376 MHz, $CDCl_3$) δ -132.3 (qd, *J* = 23.5, 4.5 Hz).

HRMS (ESI): calculated for $C_{13}H_{14}ClFO_2Na^+$ $[M+Na]^+$ m/z: 279.0559, found: 279.0558.

Ethyl 3-(4-bromophenyl)-3-fluoro-2-methylenebutanoate ((±)-3d)

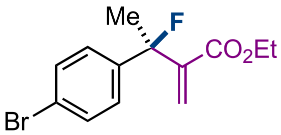

Prepared according to general procedure C using alkene **1d** (80 mg, 0.4 mmol), reagent **2a** (120 mg, 0.2 mmol) and Et_3N•3HF (96 mg, 0.6 mmol). Ratio of *branched*:*linear* isomers was determined to be > 20:1 from the crude reaction mixture using ^{19}F-NMR spectroscopy. Purification by flash chromatography on silica gel (hexane/ethyl acetate = 50/1) provided the title compound as colorless oil (43 mg, 70% yield).

^{1}H NMR (400 MHz, $CDCl_3$) δ 7.52 – 7.40 (m, 2H), 7.38 – 7.28 (m, 2H), 6.39 (dd, *J* = 4.4, 0.9 Hz, 1H), 6.13 (d, *J* = 0.9 Hz, 1H), 4.07 (qd, *J* = 7.1, 3.1 Hz, 2H), 1.98 (d, *J* = 23.5 Hz, 3H), 1.17 (t, *J* = 7.1 Hz, 3H); **^{13}C NMR** (101 MHz, $CDCl_3$) δ 164.7 (d, *J* = 6.8 Hz), 143.4 (d, *J* = 21.1 Hz), 141.2 (d, *J* = 22.1 Hz), 131.3, 127.5 (d, *J* = 6.6 Hz), 125.1 (d, *J* = 11.8 Hz), 122.3 (d, *J* = 2.9 Hz), 95.5 (d, *J* = 174.8 Hz), 61.0, 25.7 (d, *J* = 24.6 Hz), 14.1; **^{19}F NMR** (376 MHz, $CDCl_3$) δ -132.7 (qd, *J* = 23.5, 4.4 Hz).

HRMS (ESI): calculated for $C_{13}H_{14}BrO_2FNa^+$ $[M+Na]^+$ m/z: 323.0053, found: 323.0050.

Ethyl 3-fluoro-3-(4-iodophenyl)-2-methylenebutanoate ((±)-3e)

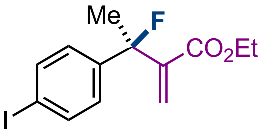

Prepared according to general procedure C using alkene **1e** (96 mg, 0.4 mmol), reagent **2a** (120 mg, 0.2 mmol) and Et_3N•3HF (96 mg, 0.6 mmol). Ratio of *branched*:*linear* isomers was determined to be > 20:1 from the crude reaction mixture using ^{19}F-NMR spectroscopy. Purification by flash chromatography on silica gel (hexane/ethyl acetate = 40/1) provided the title compound as colorless oil (49 mg, 68% yield).

^{1}H NMR (400 MHz, $CDCl_3$ δ 7.70 – 7.63 (m, 2H), 7.21 – 7.14 (m, 2H), 6.39 (dd, *J* = 4.4, 0.9 Hz, 1H), 6.13 (d, *J* = 0.9 Hz, 1H), 4.07 (qd, *J* = 7.1, 3.0 Hz, 2H), 1.97 (d, J = 23.6 Hz, 3H), 1.17 (t, *J* = 7.1 Hz, 3H); **^{13}C NMR** (101 MHz, $CDCl_3$) δ 164.6 (d, *J* = 6.4 Hz), 143.4 (d, *J* = 24.2 Hz), 141.9 (d, *J* = 22.1 Hz), 137.3, 127.7 (d, *J* = 6.6 Hz), 125.1 (d, *J* = 11.8 Hz), 95.6 (d, *J* = 174.9 Hz), 94.0 (d, *J* = 3.2 Hz), 61.0, 25.7 (d, *J* = 24.7 Hz), 14.1; **^{19}F NMR** (376 MHz, $CDCl_3$) δ -133.2 (qd, *J* = 23.6, 4.5 Hz).

HRMS (ESI): calculated for $C_{13}H_{14}O_2FINa^+$ $[M+Na]^+$ m/z: 370.9915, found: 370.9918.

Ethyl 3-fluoro-2-methylene-3-(4-(trifluoromethyl)phenyl)butanoate ((±)-3f)

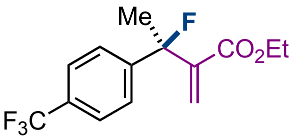

Prepared according to general procedure C using alkene **1f** (76 mg, 0.4 mmol), reagent **2a** (120 mg, 0.2 mmol) and Et_3N•3HF (96 mg, 0.6 mmol). Ratio of *branched*:*linear* isomers was determined to be > 20:1

from the crude reaction mixture using ^{19}F-NMR spectroscopy. Purification by flash chromatography on silica gel (hexane/ethyl acetate = 30/1) provided the title compound as colorless oil (43 mg, 74% yield).

^{1}H NMR (400 MHz, $CDCl_3$) δ 7.62 – 7.59 (m, 2H), 7.56 – 7.54 (m, 2H), 6.43 (d, *J* = 4.2 Hz, 1H), 6.17 (s, 1H), 4.13 – 4.01 (m, 2H), 2.01 (d, *J* = 23.6 Hz, 3H), 1.16 (t, *J* = 7.1 Hz, 3H); **^{13}C NMR** (126 MHz, $CDCl_3$) δ 164.6 (d, *J* = 6.1 Hz), 146.2 (d, *J* = 20.4 Hz), 143.2 (d, *J* = 23.8 Hz), 130.3 (qd *J* = 32.6, 2.2 Hz), 126.0 (d, *J* = 7.0 Hz), 125.5 (d, *J* = 11.6 Hz), 125.2 (q, *J* = 3.8 Hz), 124.2 (q, *J* = 271.9 Hz), 95.5 (d, 175.5 Hz), 61.0, 26.0 (d, *J* = 24.8 Hz), 14.0; **^{19}F NMR** (376 MHz, $CDCl_3$) δ -62.7 (s, 3F), -134.7 (qd, *J* = 23.5, 4.2 Hz, 1F).

HRMS (ESI): calculated for $C_{14}H_{14}F_4O_2Na^+$ $[M+Na]^+$ m/z: 313.0822, found: 313.0825.

Methyl 4-(3-(ethoxycarbonyl)-2-fluorobut-3-en-2-yl)benzoate ((±)-3g)

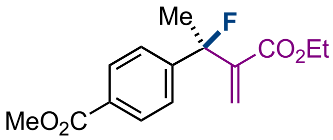

Prepared according to general procedure C using alkene **1g** (70 mg, 0.4 mmol), reagent **2a** (120 mg, 0.2 mmol) and Et_3N•3HF (96 mg, 0.6 mmol). Ratio of *branched*:*linear* isomers was determined to be > 20:1 from the crude reaction mixture using ^{19}F-NMR spectroscopy. Purification by flash chromatography on silica gel (hexane/ethyl acetate = 10/1) provided the title compound as colorless oil (40 mg, 66% yield).

^{1}H NMR (400 MHz, $CDCl_3$) δ 8.02 – 8.00 (m, 2H), 7.51 – 7.49 (m, 2H), 6.42 (dd, *J* = 4.1, 0.8 Hz, 1H), 6.16 – 6.14 (m, 1H), 4.09 – 4.03 (m, 2H), 3.91 (s, 3H), 2.00 (d, *J* = 23.5 Hz, 3H), 1.14 (t, *J* = 7.1 Hz, 3H); **^{13}C NMR** (101 MHz, $CDCl_3$) δ 166.9, 164.6 (d, *J* = 5.8 Hz), 147.1 (d, *J* = 21.8 Hz), 143.3 (d, *J* = 23.9 Hz), 129.8 (d, *J* = 2.2 Hz), 129.5, 125.6 (d, *J* = 6.8 Hz), 125.5 (d, *J* = 11.4 Hz), 95.6 (d, *J* = 175.2 Hz), 61.0, 52.3, 26.0 (d, *J* = 24.8 Hz), 14.0; **^{19}F NMR** (376 MHz, $CDCl_3$) δ -134.7 (qd, *J* = 23.5, 4.1 Hz).

HRMS (ESI): calculated for $C_{15}H_{17}O_4FNa^+$ $[M+Na]^+$ m/z: 303.1003, found: 303.1015.

Ethyl 3-fluoro-2-methylene-3-(4-(trifluoromethoxy)phenyl)butanoate ((±)-3h)

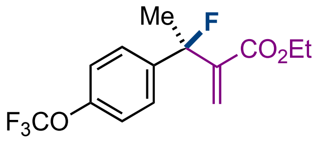

Prepared according to general procedure C using alkene **1h** (80 mg, 0.4 mmol), reagent **2a** (120 mg, 0.2 mmol) and Et_3N•3HF (96 mg, 0.6 mmol). Ratio of *branched*:*linear* isomers was determined to be > 20:1 from the crude reaction mixture using ^{19}F-NMR spectroscopy. Purification by flash chromatography on silica gel (hexane/ethyl acetate = 20/1) provided the title compound as colorless oil (41 mg, 68% yield).

^{1}**H NMR** (400 MHz, $CDCl_3$) δ 7.53 – 7.43 (m, 2H), 7.21 – 7.16 (m, 2H), 6.45 – 6.38 (m, 1H), 6.21 – 6.10 (m, 1H), 4.15 – 4.01 (m, 2H), 2.01 (d, *J* = 23.6 Hz, 3H), 1.15 (td, *J* = 7.1, 0.5 Hz, 3H); ^{13}**C NMR** (101 MHz, $CDCl_3$) δ 164.7 (d, *J* = 6.5 Hz), 149.0 (m), 143.5 (d *J* = 24.2 Hz), 140.8 (d, *J* = 22.3 Hz), 127.4 (d, *J* = 6.7 Hz), 125.1 (d, *J* = 11.9 Hz), 120.6 (q, *J* = 257.7 Hz), 120.5, 95.4 (d, *J* = 174.2 Hz), 61.0, 25.9 (d, *J* = 24.6 Hz), 14.0; ^{19}**F NMR** (376 MHz, $CDCl_3$) δ -58.9 (s, 3F), -132.2 (qd, *J* = 23.6, 4.5 Hz, 1F).

HRMS (ESI): calculated for $C_{14}H_{14}O_2F_4Na^+$ $[M+Na]^+$ m/z: 329.0770, found: 329.0771.

Ethyl 3-(4-acetoxyphenyl)-3-fluoro-2-methylenebutanoate ((±)-3i)

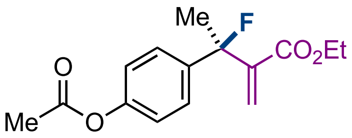

Prepared according to general procedure C using alkene **1i** (74 mg, 0.4 mmol), reagent **2a** (120 mg, 0.2 mmol) and Et_3N•3HF (96 mg, 0.6 mmol). Ratio of *branched*:*linear* isomers was determined to be > 20:1 from the crude reaction mixture using ^{19}F-NMR spectroscopy. Purification by flash chromatography on silica gel (hexane/ethyl acetate = 10/1) provided the title compound as colorless oil (44 mg, 78% yield).

^{1}**H NMR** (400 MHz, $CDCl_3$) δ 7.50 – 7.37 (m, 2H), 7.10 – 7.03 (m, 2H), 6.38 (dd, *J* = 4.4, 1.0 Hz, 1H), 6.12 (d, *J* = 1.0 Hz, 1H), 4.07 (qd, *J* = 7.1, 3.6 Hz, 2H), 2.29 (s, 3H), 2.01 (d, *J* = 23.6 Hz, 3H), 1.16 (t, *J* = 7.1 Hz, 3H); ^{13}**C NMR** (101 MHz, $CDCl_3$) δ 169.4, 164.8 (d, *J* = 6.5 Hz), 150.4 (d, *J* = 2.6 Hz), 143.7 (d, *J* = 24.4 Hz), 139.6 (d, *J* = 22.2 Hz), 127.0 (d, *J* = 6.7 Hz), 124.9 (d, *J* = 12.0 Hz), 121.2, 95.6 (d, *J* = 174.7), 60.1, 25.9 (d, *J* = 24.8 Hz), 21.3, 14.0; ^{19}**F NMR** (376 MHz, $CDCl_3$) δ -131.8 (qd, *J* = 23.6, 4.5 Hz).

HRMS (ESI): calculated for $C_{15}H_{17}OF_4Na^+$ $[M+Na]^+$ m/z: 303.1003, found: 303.0998.

Ethyl 3-(4-(acryloyloxy)phenyl)-3-fluoro-2-methylenebutanoate ((±)-3j)

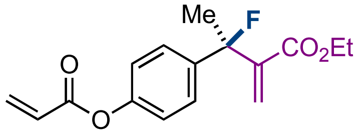

Prepared according to general procedure C using alkene **1j** (76 mg, 0.4 mmol), reagent **2a** (120 mg, 0.2 mmol) and Et3N•3HF (96 mg, 0.6 mmol). Ratio of *branched*:*linear* isomers was determined to be > 20:1 from the crude reaction mixture using ^{19}F-NMR spectroscopy. Purification by flash chromatography on silica gel (hexane/ethyl acetate = 20/1) provided the title compound as colorless oil (48 mg, 82% yield).

^{1}**H NMR** (400 MHz, $CDCl_3$) δ 7.46 (dd, *J* = 8.8, 1.1 Hz, 2H), 7.15 – 7.08 (m, 2H), 6.60 (dd, *J* = 17.3, 1.3 Hz, 1H), 6.38 (dd, *J* = 4.4, 1.0 Hz, 1H), 6.31 (dd, *J* = 17.3, 10.4 Hz, 1H), 6.13 (d, *J* = 1.0 Hz, 1H), 6.01 (dd, *J* = 10.4, 1.3 Hz, 1H), 4.14 – 4.00 (m, 2H), 2.01 (d, *J* = 23.6 Hz, 3H), 1.16 (t, *J* = 7.1 Hz, 3H); ^{13}**C NMR** (101 MHz, $CDCl_3$) δ 164.8 (d, *J* = 6.6 Hz), 164.5,

150.3 (d, J = 2.6 Hz), 143.7 (d, J = 24.3 Hz), 139.7 (d, J = 22.3 Hz), 132.8, 128.0, 127.0 (d, J = 6.6 Hz), 125.0 (d, J = 11.9 Hz), 121.2, 95.6 (d, J = 169.1 Hz), 60.9, 25.9 (d, J = 24.7 Hz), 14.0; **^{19}F NMR** (376 MHz, $CDCl_3$) δ -131.4 (qd, J = 23.6, 4.4 Hz).

HRMS (ESI): calculated for $C_{16}H_{17}O_4FNa^+$ $[M+Na]^+$ m/z: 315.1003, found: 315.0998.

Ethyl 3-(4-(3,3-dimethylbut-1-yn-1-yl)phenyl)-3-fluoro-2-methylenebutanoate ((±)-3k)

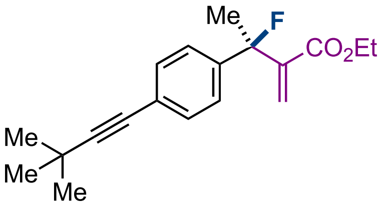

Prepared according to general procedure C using alkene **1k** (80 mg, 0.4 mmol), reagent **2a** (120 mg, 0.2 mmol) and Et_3N•3HF (90 mg, 0.6 mmol). Ratio of *branched*:*linear* isomers was determined to be > 20:1 from the crude reaction mixture using ^{19}F-NMR spectroscopy. Purification by flash chromatography on silica gel (hexane/ethyl acetate = 30/1) provided the title compound as colorless oil (38 mg, 63% yield).

^{1}H NMR (400 MHz, $CDCl_3$) δ 7.40 – 7.30 (m, 4H), 6.37 (dd, J = 4.3, 1.0 Hz, 1H), 6.10 (d, J = 1.0 Hz, 1H), 4.11 – 3.99 (m, 2H), 1.99 (d, J = 23.5 Hz, 3H), 1.31 (s, 9H), 1.15 (t, J = 7.1 Hz, 3H); **^{13}C NMR** (101 MHz, $CDCl_3$) δ 164.8 (d, J = 6.3 Hz), 143.7 (d, J = 24.3 Hz), 141.1 (d, J = 21.7 Hz), 131.4, 125.5 (d, J = 6.6 Hz), 124.9 (d, J = 11.7 Hz), 124.0 (d, J = 2.4 Hz), 99.1, 95.7 (d, J = 174.5 Hz), 78.8 (d, J = 1.4 Hz), 60.9, 31.1, 28.1, 25.7 (d, J = 24.8 Hz), 14.1; **^{19}F NMR** (376 MHz, $CDCl_3$) δ -132.6 (qd, J = 23.5, 4.3 Hz).

HRMS (ESI): calculated for $C_{19}H_{23}O_2FNa^+$ $[M+Na]^+$ m/z: 325.1574, found: 325.1564.

Ethyl -3-fluoro-2-methylene-3-(p-tolyl)butanoate ((±)-3l)

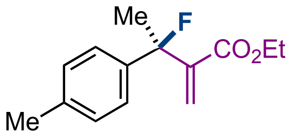

Prepared according to general procedure C using alkene **1l** (53 mg, 0.4 mmol), reagent **2a** (120 mg, 0.2 mmol) and Et_3N•3HF (96 mg, 0.6 mmol). Ratio of *branched*:*linear* isomers was determined to be > 20:1 from the crude reaction mixture using ^{19}F-NMR spectroscopy. Purification by flash chromatography on silica gel (hexane/ethyl acetate = 30/1) provided the title compound as colorless oil (15 mg, 30% yield).

^{1}H NMR (400 MHz, $CDCl_3$) δ 7.32 (dd, J = 8.3, 1.3 Hz, 2H), 7.18 – 7.12 (m, 2H), 6.36 (dd, J = 4.4, 1.1 Hz, 1H), 6.11 (d, J = 1.1 Hz, 1H), 4.12 – 4.03 (m, 2H), 2.33 (s, 3H), 2.00 (d, J = 23.5 Hz, 3H), 1.36 (t, J = 7.3 Hz, 3H); **^{13}C NMR** (101 MHz, $CDCl_3$) δ 164.8 (d, J = 7.0 Hz), 144.0 (d, J = 24.8 Hz), 143.9 (d, J = 24.8 Hz), 137.8 (d, J = 2.4 Hz), 128.7, 125.5 (d, J = 6.2 Hz), 124.4 (d, J = 11.8 Hz), 95.8 (d, J = 173.8 Hz), 60.7, 25.6 (d, J = 24.7 Hz), 21.1, 13.9 (*peaks at 46.8, 29.7 and 8.6 are from inseparable impurities*); **^{19}F NMR** (376 MHz, $CDCl_3$) δ -130.2 (q, J = 23.5 Hz).

HRMS (ESI): calculated for $C_{14}H_{17}O_2FNa^+$ $[M+Na]^+$ m/z: 259.1105, found: 259.1106.

Ethyl 3-fluoro-3-(3-methoxyphenyl)-2-methylenebutanoate ((±)-3n)

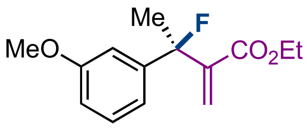

Prepared according to modified general procedure C using alkene **1n** (60 mg, 0.4 mmol), reagent **2a** (120 mg, 0.2 mmol) and Et_3N•3HF (96 mg, 0.6 mmol). The addition of **2a** was carried out at -70 °C instead of -50 °C. Ratio of *branched*:*linear* isomers was determined to be 15:1 from the crude reaction mixture using ^{19}F-NMR spectroscopy. Purification by flash chromatography on silica gel (hexane/ethyl acetate = 20/1) provided the title compound as colorless oil (35 mg, 70% yield).

^{1}H NMR (400 MHz, $CDCl_3$) δ 7.29 – 7.23 (m, 1H), 7.04 – 6.97 (m, 2H), 6.85 – 6.82 (m, 1H), 6.38 (dd, *J* = 4.1, 1.0 Hz, 1H), 6.10 (dd, *J* = 1.0, 0.5 Hz, 1H), 4.12 – 4.04 (m, 2H), 3.80 (s, 3H), 2.00 (d, *J* = 23.5 Hz, 3H), 1.16 (t, *J* = 7.1 Hz, 3H); **^{13}C NMR** (101 MHz, $CDCl_3$) δ 164.9 (d, *J* = 6.0 Hz), 159.5, 143.8 (d, *J* = 24.2 Hz), 143.7 (d, *J* = 21.8 Hz), 129.2, 124.9 (d, *J* = 11.6 Hz), 118.0 (d, *J* = 6.5 Hz), 113.4 (d, *J* = 2.1 Hz), 111.7 (d, *J* = 7.1 Hz), 95.8 (d, *J* = 174.7 Hz), 60.8, 55.4, 26.0 (d, *J* = 24.9 Hz), 14.0; **^{19}F NMR** (376 MHz, $CDCl_3$) δ -132.4 (qd, *J* = 23.6, 4.1 Hz).

HRMS (ESI): calculated for $C_{14}H_{17}O_3FNa^+$ $[M+Na]^+$ m/z: 275.1054, found: 275.1050.

Ethyl 3-fluoro-2-methylene-3-(m-tolyl)butanoate ((±)-3o)

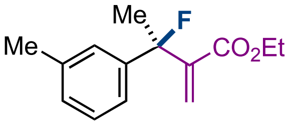

Prepared according to general procedure C using alkene **1o** (54 mg, 0.4 mmol), reagent **2a** (120 mg, 0.2 mmol) and Et_3N•3HF (96 mg, 0.6 mmol). Ratio of *branched*:*linear* isomers was determined to be > 20:1 from the crude reaction mixture using ^{19}F-NMR spectroscopy. Purification by flash chromatography on silica gel (hexane/ethyl acetate = 30/1) provided the title compound as colorless oil (47 mg, 87% yield).

^{1}H NMR (400 MHz, $CDCl_3$) δ 7.24 – 7.22 (m, 3H), 7.12 – 7.09 (m, 1H), 6.37 (dd, *J* = 4.4, 1.1 Hz, 1H), 6.11 (d, *J* = 1.1 Hz, 1H), 4.13 – 4.01 (m, 2H), 2.35 (s, 3H), 2.00 (d, *J* = 23.6 Hz, 3H), 1.16 (t, *J* = 7.2 Hz, 3H); **^{13}C NMR** (101 MHz, $CDCl_3$) δ 164.9 (d, *J* = 6.3 Hz), 144.0 (d, *J* = 24.3 Hz), 141.9 (d *J* = 21.6 Hz), 137.8, 128.9 (d, *J* = 2.4 Hz), 128.1, 126.3 (d, *J* = 6.5 Hz), 124.8 (d, *J* = 11.8 Hz), 122.8 (d, *J* = 6.5 Hz), 95.9 (d, *J* = 173.9 Hz), 60.8, 25.9 (d, *J* = 24.9 Hz), 21.7, 14.0; **^{19}F NMR** (376 MHz, $CDCl_3$) δ -131.6 (qd, *J* = 23.6, 4.4 Hz).

HRMS (ESI): calculated for $C_{14}H_{17}O_2FNa^+$ $[M+Na]^+$ m/z: 259.1105, found: 295.1104.

Ethyl 3-(4-acetoxyphenyl)-3-fluoro-2-methylenebutanoate ((±)-3p)

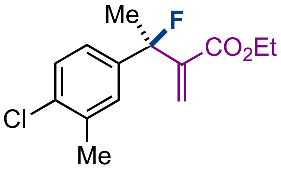

Prepared according to general procedure C using alkene **1p** (66 mg, 0.4 mmol), reagent **2a** (120 mg, 0.2 mmol) and Et_3N•3HF (96 mg, 0.6 mmol). Ratio of *branched*:*linear* isomers was determined to be > 20:1 from the crude reaction mixture using ^{19}F-NMR spectroscopy. Purification by flash chromatography on silica gel (hexane/ethyl acetate = 30/1) provided the title compound as colorless oil (40 mg, 74% yield).

^{1}H NMR (400 MHz, CD_2Cl_2) δ 7.32 – 7.30 (m, 2H), 7.21 – 7.18 (m, 1H), 6.38 (dd, *J* = 4.3, 0.9 Hz, 1H), 6.10 (d, *J* = 0.9 Hz, 1H), 4.10 – 4.01 (m, 2H), 2.37 (s, 3H), 1.96 (d, *J* = 23.6 Hz, 3H), 1.16 (t, *J* = 7.1 Hz, 3H); **^{13}C NMR** (101 MHz, CD_2Cl_2) δ 164.9 (d, *J* = 6.3 Hz), 143.9 (d, *J* = 24.0 Hz), 141.1 (d, *J* = 21.9 Hz), 136.1, 134.3 (d, *J* = 2.8 Hz), 128.9, 128.6 (d, *J* = 6.5 Hz), 125.1 (d, *J* = 11.7 Hz), 124.9 (d, *J* = 6.3 Hz), 95.8 (d, *J* = 174.0 Hz), 61.2, 25.9 (d, *J* = 24.7 Hz), 20.3, 14.1; **^{19}F NMR** (376 MHz, $CDCl_3$) δ -131.9 (qd, *J* = 23.6, 4.4 Hz).

HRMS (ESI): calculated for $C_{14}H_{16}O_2FClNa^+$ $[M+Na]^+$ m/z: 293.0715, found: 293.0704.

Ethyl 3-fluoro-2-methylene-3-(naphthalen-2-yl)butanoate ((±)-3q)

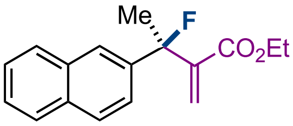

Prepared according to general procedure C using alkene **1q** (68 mg, 0.4 mmol), reagent **2a** (120 mg, 0.2 mmol) and Et_3N•3HF (96 mg, 0.6 mmol). Ratio of *branched*:*linear* isomers was determined to be > 20:1 from the crude reaction mixture using ^{19}F-NMR spectroscopy. Purification by flash chromatography on silica gel (hexane/ethyl acetate = 30/1) provided the title compound as colorless oil (41 mg, 76% yield).

^{1}H NMR (400 MHz, $CDCl_3$) δ 7.92 (d, *J* = 2.0 Hz, 1H), 7.89 – 7.78 (m, 3H), 7.54 (dd, *J* = 8.7, 1.9 Hz, 1H), 7.52 – 7.44 (m, 2H), 6.43 (dd, *J* = 4.3, 1.1 Hz, 1H), 6.19 (d, *J* = 1.1 Hz, 1H), 4.04 (qd, *J* = 7.1, 4.0 Hz, 2H), 2.13 (d, *J* = 23.5 Hz, 3H), 1.12 (t, *J* = 7.1 Hz, 3H); **^{13}C NMR** (101 MHz, $CDCl_3$) δ 164.9 (d, *J* = 6.3 Hz), 143.9 (d, *J* = 24.2 Hz), 139.4 (d, *J* = 21.6 Hz), 133.1 (d, *J* = 1.7 Hz), 133.0, 128.5, 127.9, 127.7, 126.4 126.3, 125.0 (d, *J* = 11.8 Hz), 124.9 (d, *J* = 7.3 Hz), 123.7 (d, *J* = 6.1 Hz), 96.1 (d, *J* = 174.1 Hz), 60.9, 26.0 (d, *J* = 24.8 Hz), 14.0; **^{19}F NMR** (376 MHz, $CDCl_3$) δ -132.0 (qd, *J* = 23.5, 4.3 Hz).

HRMS (ESI): calculated for $C_{17}H_{17}O_2FNa^+$ $[M+Na]^+$ m/z: 295.1105, found: 295.1113.

Note: Under the optimized reaction conditions we have performed experiments with Rh_2(*S*-NTTL)$_4$, Rh_2(*R*-PTAD)$_4$ and Rh_2(*R*-DOSP)$_4$. Unfortunately, only Rh_2(*R*-DOSP)$_4$ provided **3q** with 20% yield and with 0% of enantiomeric excess.

The separation of the racemic mixture **3q** was performed on an Agilent 1260 Infinity II SFC system equipped with Daicel Chiralpak IG column (100 x 3 mm, particle size 3 μm. 1% IPA in supercritical CO_2, 1.0 mL/min). The chromatogram is indicated below and the retention times for racemic **3q** are 3.84 min, 4.99 min.

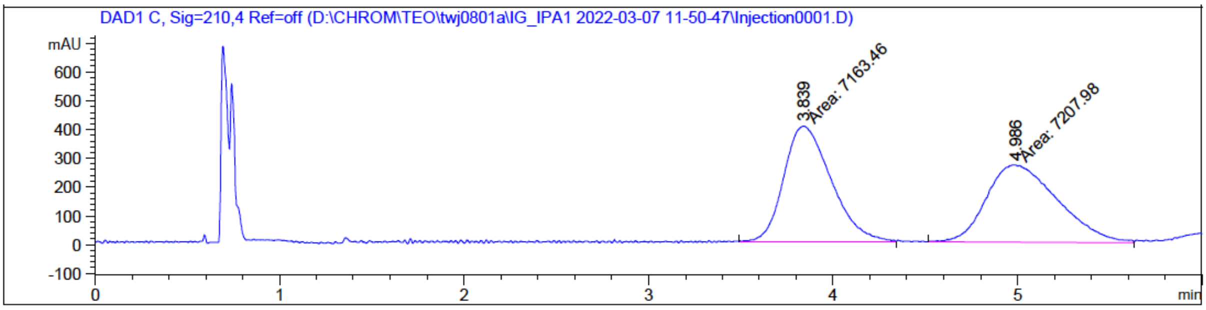

Signal 3: DAD1 C, Sig=210,4 Ref=off

Peak #	RetTime [min]	Type	Width [min]	Area [mAU*s]	Height [mAU]	Area %
1	3.839	MM	0.2973	7163.45605	401.60138	49.8451
2	4.986	MM	0.4468	7207.97510	268.86902	50.1549

Analysis of a sample of **3q** synthesized with Rh_2(R-DOSP)$_4$ using SFC on the same conditions determined a 0% of enantiomeric excess (ee). The chromatogram is indicated below and the retention times are 3.75 min, 4.94 min.

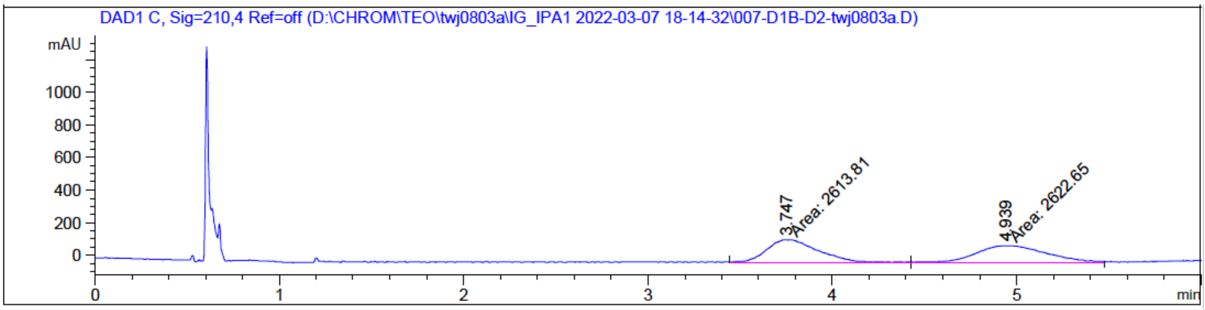

Signal 3: DAD1 C, Sig=210,4 Ref=off

Peak #	RetTime [min]	Type	Width [min]	Area [mAU*s]	Height [mAU]	Area %
1	3.747	MM	0.3137	2613.81250	138.85881	49.9156
2	4.939	MM	0.4276	2622.65308	102.22952	50.0844

Ethyl 3-fluoro-2-methylene-3-(o-tolyl)butanoate ((±)3r) and Ethyl (*Z*)-2-(fluoromethyl)-3-(o-tolyl)but-2-enoate (3r')

Prepared according to general procedure C using alkene **1r** (54 mg, 0.4 mmol), reagent **2a** (120 mg, 0.2 mmol) and Et_3N•3HF (96 mg, 0.6 mmol). Ratio of *branched*:*linear* isomers was determined to be 1:1.4 and ratio of *Z*:*E* isomers from the *linear* isomer was determined to be >

20:1 from the crude reaction mixture using ^{19}F-NMR spectroscopy. Purification by flash chromatography on silica gel (hexane/ethyl acetate = 40/1) provided a mixture of the title compounds as colorless oil (38 mg, 77% yield). Separation of **(±)-3r** and **3r'** was achieved using a PLC plate.

(±)-3r

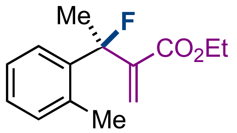

^{1}H NMR (500 MHz, $CDCl_3$) δ 7.47 – 7.42 (m, 1H), 7.24 – 7.16 (m, 2H), 7.15 – 7.09 (m, 1H), 6.34 (dd, *J* = 3.2, 1.0 Hz, 1H), 5.99 – 5.97 (m, 1H), 4.13 – 3.97 (m, 2H), 2.39 – 2.33 (m, 3H), 2.06 (d, *J* = 23.9 Hz, 3H), 1.13 (t, *J* = 7.1 Hz, 3H); **^{13}C NMR** (126 MHz, $CDCl_3$) δ 165.2 (d, *J* = 5.6 Hz), 143.8 (d, *J* = 23.2 Hz), 138.9 (d, *J* = 20.4 Hz), 136.8 (d, *J* = 1.5 Hz), 132.2 (d, *J* = 1.6 Hz), 128.4 (d, *J* = 1.8 Hz), 127.4 (d, *J* = 7.4 Hz), 125.4, 125.0 (d, *J* = 9.5 Hz), 97.0 (d, *J* = 174.2 Hz), 60.8, 27.5 (d, *J* = 25.6 Hz), 21.2 (d, *J* = 6.1 Hz), 14.0; **^{19}F NMR** (376 MHz, $CDCl_3$) δ -136.5 (q, *J* = 23.9 Hz).

HRMS (ESI): calculated for $C_{14}H_{17}O_2FNa^+$ $[M+Na]^+$ m/z: 259.1105, found: 259.1096.

3r'

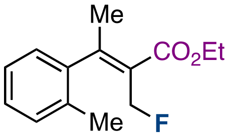

^{1}H NMR (500 MHz, $CDCl_3$) δ 7.25 – 7.16 (m, 3H), 7.00 – 6.98 (m, 1H), 4.88 – 4.77 (m, 1H), 4.77 – 4.67 (m, 1H), 4.33 (q, *J* = 7.1 Hz, 2H), 2.38 – 2.32 (m, 3H), 2.21 (s, 3H), 1.37 (t, *J* = 7.1 Hz, 3H); **^{13}C NMR** (126 MHz, $CDCl_3$) δ 167.2, 156.2 (d, *J* = 8.5 Hz), 141.4 (d, *J* = 2.6 Hz), 133.7 (d, *J* = 2.6 Hz), 130.5, 128.0, 126.8 (d, *J* = 2.5 Hz), 126.2, 126.1, 80.6 (d, *J* = 162.3 Hz), 60.9, 23.3 (d, *J* = 2.4 Hz), 19.3, 14.4; **^{19}F NMR** (376 MHz, $CDCl_3$) δ -208.2 (tq, *J* = 47.1, 6.2 Hz); ^{1}H-^{13}C HSQC, ^{1}H-^{13}C HMBC, ^{1}H-^{1}H NOESY and ^{1}H-^{19}F HOESY spectra were measured.

HRMS (ESI): calculated for $C_{14}H_{17}O_2FNa^+$ $[M+Na]^+$ m/z: 259.1105, found: 259.1098.

Ethyl 3-(2-chlorophenyl)-3-fluoro-2-methylenebutanoate ((±)-3s) and ethyl (*Z*)-3-(2-chlorophenyl)-2-(fluoromethyl)but-2-enoate (3s')

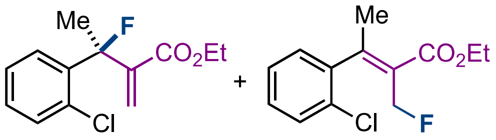

Prepared according to general procedure C using alkene **1s** (60 mg, 0.4 mmol), reagent **2a** (120 mg, 0.2 mmol) and Et_3N•3HF (96 mg, 0.6 mmol). Ratio of *branched*:*linear* isomers was determined to be 1:1.3 and ratio of *Z*:*E* isomers from the *linear* isomer was determined to be > 20:1 from the crude reaction mixture using ^{19}F-NMR spectroscopy. Purification by flash chromatography on silica gel (hexane/ethyl acetate = 40/1) provided the title compound as colorless oil (40 mg, 79% yield). **^{1}H NMR** (400 MHz, $CDCl_3$) δ 7.69 (dd, *J* = 7.9, 1.6 Hz, 1H*), 7.44 – 7.39 (m, 1H), 7.32 – 7.27 (m, 2H+1H*), 7.26 – 7.23 (m, 2H*), 7.18 – 7.13 (m, 1H), 6.53 (s, 1H*), 6.12 (dd, *J* = 3.0, 0.6 Hz, 1H*), 4.99 (dd, *J* =

10.2, 0.8 Hz, 1H), 4.93 (dd, J = 10.1, 0.8 Hz, 1H), 4.40 – 4.28 (m, 2H), 4.06 (q, J = 7.1 Hz, 2H*), 2.38 (d, J = 6.3 Hz, 3H), 2.04 (d, J = 23.7 Hz, 3H*), 1.37 (t, J = 7.1 Hz, 3H), 1.12 (t, J = 7.1 Hz, 3H*); **^{13}C NMR** (101 MHz, $CDCl_3$) δ 166.9, 165.0* (d, J = 3.2 Hz), 153.3 (d, J = 8.4 Hz), 141.4 (d, J = 21.6 Hz), 140.4, 140.3*, 139.7* (d, J = 22.1 Hz), 131.3, 131.3*, 131.1*, 129.9, 129.2* (d, J = 1.5 Hz), 128.8 (d, J = 2.3 Hz), 128.2* (d, J = 2.3 Hz), 128.1 (d, J = 2.2 Hz), 127.1, 127.1* (d, J = 16.6 Hz), 126.7* (d, J = 1.8 Hz), 95.1* (d, J = 175.2 Hz), 80.4 (d, J = 163.7 Hz), 61.1, 60.8*, 25.9* (d, J = 25.4 Hz), 22.7 (d, J = 2.4 Hz), 14.4, 14.0*; **^{19}F NMR** (376 MHz, $CDCl_3$) δ -208.0 (tq, J = 47.1, 6.2 Hz), -135.0* (qd, J = 23.7, 3.2 Hz); * *indicates the signals of the branched isomer*; ^{1}H-^{13}C HSQC, ^{1}H-^{13}C HMBC, ^{1}H-^{1}H NOE and ^{1}H-^{19}F HOESY spectra were measured.

HRMS (ESI): calculated for $C_{13}H_{14}ClFO_2Na^+$ [M+Na]$^+$ m/z: 279.0559, found: 279.0554.

Ethyl 3-fluoro-2-methylene-3-phenylhexanoate ((±)-3t)

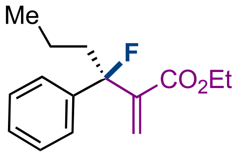

Prepared according to general procedure C using alkene **1t** (60 mg, 0.4 mmol), reagent **2a** (120 mg, 0.2 mmol) and Et_3N•3HF (96 mg, 0.6 mmol). Ratio of *branched*:*linear* isomers was determined to be 15:1 from the crude reaction mixture using ^{19}F-NMR spectroscopy. Purification by flash chromatography on silica gel (hexane/ethyl acetate = 40/1) provided the title compound as colorless oil (38 mg, 75% yield).

^{1}H NMR (400 MHz, $CDCl_3$) δ 7.45 – 7.42 (m, 2H), 7.38 – 7.20 (m, 2H), 7.30 – 7.24 (m, 1H), 6.36 (dd, J = 4.9, 1.1 Hz, 1H), 6.10 (d, J = 1.1 Hz, 1H), 4.14 – 4.02 (m, 2H), 2.62 – 2.41 (m, 1H), 2.35 – 2.17 (m, 1H), 1.51 – 1.28 (m, 2H), 1.16 (t, J = 7.1 Hz, 3H), 1.02 – 0.92 (m, 3H); **^{13}C NMR** (101 MHz, $CDCl_3$) δ 165.0 (d, J = 7.7 Hz), 143.0 (d, J = 25.2 Hz), 142.0 (d, J = 22.2 Hz), 128.1, 127.9 (d, J = 2.1 Hz), 125.9 (d, J = 7.4 Hz), 125.0 (d, J = 12.9 Hz), 98.6 (d, J = 179.3 Hz), 60.8, 39.2 (d, J = 22.6 Hz), 17.0 (d, J = 3.7 Hz), 14.4, 14.0; **^{19}F NMR** (376 MHz, $CDCl_3$) δ -142.7 (ddd, J = 34.9, 22.5, 5.0 Hz).

HRMS (ESI): calculated for $C_{15}H_{19}O_2FNa^+$ [M+Na]$^+$ m/z: 273.1261, found: 273.1270.

Ethyl 3-fluoro-2-methylene-3,5-diphenylpentanoate ((±)-3u)

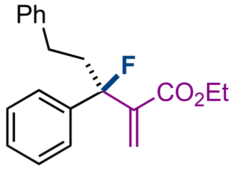

Prepared according to general procedure C using alkene **1u** (83 mg, 0.4 mmol), reagent **2a** (120 mg, 0.2 mmol) and Et_3N•3HF (96, 0.6 mmol). Ratio of *branched*:*linear* isomers was determined to be 10:1 from the crude reaction mixture using ^{19}F-NMR spectroscopy. Purification by flash

chromatography on silica gel (hexane/ethyl acetate = 40/1) provided the title compound as colorless oil (43 mg, 69% yield).

^{1}H NMR (400 MHz, $CDCl_3$) δ 7.50 – 7.48 (m, 2H), 7.38 – 7.33 (m, 2H), 7.32 – 7.27 (m, 3H), 7.21 – 7.18 (m, 3H), 6.42 (dd, *J* = 1H), 6.18 (d, *J* = 1H), 4.16 – 4.03 (m, 2H), 2.99 – 2.83 (m, 1H), 2.75 – 2.52 (m, 3H), 1.19 (t, *J* = 7.1 Hz, 3H); **^{13}C NMR** (101 MHz, $CDCl_3$) δ 164.9 (d, *J* = 7.6 Hz), 142.4 (d, *J* = 24.8 Hz), 141.9, 141.2 (d, *J* = 22.1 Hz), 128.6, 128.6, 128.2, 128.1 (d, *J* = 2.0 Hz), 126.1, 125.9 (d, *J* = 7.5 Hz), 125.4 (d, *J* = 13.1 Hz), 97.7 (d, *J* = 178.7 Hz), 60.9, 39.2 (d, *J* = 22.3 Hz), 30.1 (d, *J* = 4.0 Hz), 14.1; **^{19}F NMR** (376 MHz, $CDCl_3$) δ -144.9 (ddd, *J* = 30.1, 19.4, 5.1 Hz).

HRMS (ESI): calculated for $C_{20}H_{21}O_2FNa^+$ $[M+Na]^+$ m/z: 335.1418, found: 335.1414.

Ethyl 2-(cyclohexylfluoro(phenyl)methyl)acrylate ((±)-3v)

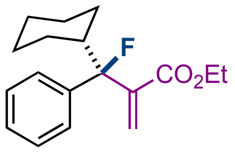

Prepared according to general procedure C using alkene **1v** (75 mg, 0.4 mmol), reagent **2a** (120 mg, 0.2 mmol) and Et_3N•3HF (96 mg, 0.6 mmol). Ratio of *branched*:*linear* isomers was determined to be 11:1 from the crude reaction mixture using ^{19}F-NMR spectroscopy. Purification by flash chromatography on silica gel (hexane/ethyl acetate = 40/1) provided the title compound as colorless oil (24 mg, 42% yield).

^{1}H NMR (500 MHz, $CDCl_3$) δ 7.54 – 7.43 (m, 2H), 7.38 – 7.28 (m, 2H), 7.26 – 7.21 (m, 1H), 6.21 (dd, *J* = 6.4, 1.2 Hz, 1H), 6.10 – 6.05 (m, 1H), 4.16 – 4.05 (m, 2H), 2.82 – 2.70 (m, 1H), 1.86 – 1.77 (m, 1H), 1.76 – 1.61 (m, 3H), 1.55 – 1.48 (m, 1H), 1.38 – 1.24 (m, 3H), 1.21 (t, *J* = 7.1 Hz, 3H), 1.19 – 1.05 (m, 2H); **^{13}C NMR** (126 MHz, $CDCl_3$) δ 165.2 (d, *J* = 8.8 Hz), 143.0 (d, *J* = 25.9 Hz), 141.0 (d, *J* = 23.2 Hz), 128.0 (d, *J* = 1.2 Hz), 127.4 (d, *J* = 1.3 Hz), 125.9 (d, *J* = 9.9 Hz), 124.1 (d, *J* = 15.4 Hz), 100.3 (d, *J* = 183.6 Hz), 60.8, 42.1 (d, *J* = 20.6 Hz), 27.3 (d, *J* = 2.0 Hz), 26.7, 26.7, 26.6, 26.5, 14.1; **^{19}F NMR** (376 MHz, $CDCl_3$) δ -162.7 (dd, *J* = 32.8, 6.6 Hz).

HRMS (ESI): calculated for $C_{18}H_{23}O_2FNa^+$ $[M+Na]^+$ m/z: 313.1574, found: 313.1586.

Ethyl 3-fluoro-6-methyl-2-methylene-3-phenylheptanoate ((±)-3w)

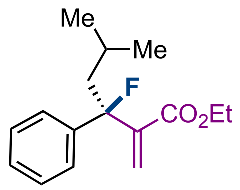

Prepared according to general procedure C using alkene **1w** (68 mg, 0.4 mmol), reagent **2a** (120 mg, 0.2 mmol) and Et_3N•3HF (96 mg, 0.6 mmol). Ratio of *branched*:*linear* isomers was determined to be 7:1 from the crude reaction mixture using ^{19}F-NMR spectroscopy. Purification by flash

chromatography on silica gel (hexane/ethyl acetate = 40/1) provided the title compound as colorless oil (34 mg, 67% yield).

^{1}H NMR (400 MHz, $CDCl_3$) δ 7.49 – 7.41 (m, 2H), 7.38 – 7.22 (m, 3H), 6.37 (dd, *J* = 5.1, 1.2 Hz, 1H), 6.13 (d, *J* = 1.2 Hz, 1H), 4.13 – 4.02 (m, 2H), 2.54 (ddd, *J* = 34.7, 14.7, 5.3 Hz, 1H), 2.22 – 2.08 (m, 1H), 1.83 – 1.70 (m, 1H), 1.18 (t, *J* = 7.1 Hz, 3H), 0.99 – 0.90 (m, 6H); **^{13}C NMR** (101 MHz, $CDCl_3$) δ 165.1 (d, *J* = 7.7 Hz), 143.0 (d, *J* = 25.2 Hz), 142.0 (d, *J* = 22.2 Hz), 128.1, 127.9 (d, *J* = 2.1 Hz), 125.9 (d, *J* = 7.4 Hz), 125.0 (d, *J* = 12.9 Hz), 98.6 (d, *J* = 179.2 Hz), 60.8, 45.2 (d, *J* = 21.1 Hz), 24.6, 24.4 (d, *J* = 1.6 Hz), 24.0 (d, *J* = 2.5 Hz), 14.1; **^{19}F NMR** (376 MHz, $CDCl_3$) δ -142.4 (ddd, *J* = 31.0, 21.1, 4.9 Hz).

HRMS (ESI): calculated for $C_{16}H_{21}O_2FNa^+$ $[M+Na]^+$ m/z: 287.1418, found: 287.1408.

Ethyl 3,4-difluoro-2-methylene-3-phenylbutanoate ((±)-3x)

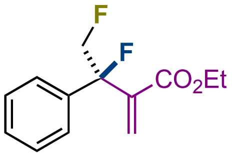

Prepared according to general procedure C using alkene **1x** (54 mg, 0.4 mmol), reagent **2a** (120 mg, 0.2 mmol) and Et_3N•3HF (96 mg, 0.6 mmol). Ratio of *branched*:*linear* isomers was determined to be 14:1 from the crude reaction mixture using ^{19}F-NMR spectroscopy. Purification by flash chromatography on silica gel (hexane/ethyl acetate = 40/1) provided the title compound as colorless oil (29 mg, 60% yield).

^{1}H NMR (400 MHz, $CDCl_3$) δ 7.45 – 7.40 (m, 2H), 7.40 – 7.32 (m, 3H), 6.55 (dd, *J* = 4.5, 0.7 Hz, 1H), 6.25 (d, *J* = 0.8 Hz, 1H), 5.35 – 5.13 (m, 1H), 5.12 – 4.94 (m, 1H), 4.19 – 4.07 (m, 2H), 1.21 (t, *J* = 7.1 Hz, 3H); **^{13}C NMR** (101 MHz, $CDCl_3$) δ 164.7 (d, *J* = 6.7 Hz), 138.9 (dd, *J* = 24.4, 3.2 Hz), 137.3 (dd, *J* = 11.0, 4.0 Hz), 129.0 (d, *J* = 2.2 Hz), 128.5, 128.3 (d, *J* = 12.9 Hz), 125.9 (d, *J* = 7.1 Hz), 96.5 (dd, *J* = 181.4, 18.7 Hz), 84.7 (dd, *J* = 181.9, 23.7 Hz), 61.2, 14.1; **^{19}F NMR** (376 MHz, $CDCl_3$) δ -150.2 (m, 1F), -226.3 (td, *J* = 47.1, 15.6 Hz, 1F).

HRMS (ESI): calculated for $C_{13}H_{14}O_2F_2Na^+$ $[M+Na]^+$ m/z: 263.0854, found: 263.0855.

Ethyl 2-(difluoro(phenyl)methyl)acrylate (3y)

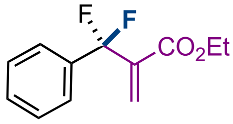

Prepared according to general procedure C using alkene **1y** (48 mg, 0.4 mmol), reagent **2a** (120 mg, 0.2 mmol) and Et_3N•3HF (96 mg, 0.6 mmol). Ratio of *branched*:*linear* isomers was determined to be > 20:1 from the crude reaction mixture using ^{19}F-NMR spectroscopy. Purification by flash chromatography on silica gel (hexane/ethyl acetate = 30/1) provided the title compound as colorless oil (37 mg, 84% yield).

^{1}H NMR (400 MHz, $CDCl_3$) δ 7.57 – 7.48 (m, 2H), 7.41 (dd, *J* = 5.3, 1.9 Hz, 3H), 6.63 (q, *J* = 0.8 Hz, 1H), 6.36 (q, *J* = 0.8 Hz, 1H), 4.13 (q, *J* = 7.1 Hz, 2H), 1.17 (t, *J* = 7.1 Hz, 3H); **^{13}C NMR** (101 MHz, $CDCl_3$) δ 163.1 (t, *J* = 2.6 Hz), 137.7 (t, *J* = 28.1 Hz), 136.2 (t, *J* = 27.0 Hz), 130.1 (t, *J* = 0.4 Hz), 129.9 (t, *J* = 7.6 Hz), 128.3, 125.7 (t, *J* = 5.8 Hz), 118.4 (t, *J* = 243.7 Hz), 61.3, 14.0; **^{19}F NMR** (376 MHz, $CDCl_3$) δ -93.0 (s, 2F).

HRMS (ESI): calculated for $C_{12}H_{12}O_2F_2Na^+$ $[M+Na]^+$ m/z: 249.0698, found: 249.0689.

Ethyl 2-(fluoromethyl)-3,3-diphenylacrylate (3z)

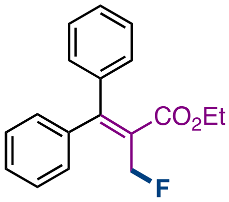

Prepared according to general procedure C using alkene **1z** (74 mg, 0.4 mmol), reagent **2a** (120 mg, 0.2 mmol) and $Et_3N{\bullet}3HF$ (96 mg, 0.6 mmol). The addition of **2a** was carried out at -70 ºC instead of -50 ºC. Ratio of *branched*:*linear* isomers was determined to be 1:15 from the crude reaction mixture using ^{19}F-NMR spectroscopy. Purification by flash chromatography on silica gel (hexane/ethyl acetate = 30/1) provided the title compound as yellowish oil (46 mg, 79% yield).

^{1}H NMR (400 MHz, $CDCl_3$) δ 7.37 (dd, *J* = 5.1, 1.8 Hz, 3H), 7.35 – 7.30 (m, 4H), 7.29 – 7.26 (m, 1H), 7.17 – 7.11 (m, 2H), 5.11 (d, *J* = 47.7 Hz, 2H), 3.99 (q, *J* = 7.1 Hz, 2H), 0.89 (t, *J* = 7.1 Hz, 3H); **^{13}C NMR** (101 MHz, $CDCl_3$) δ 169.1, 155.5 (d, *J* = 8.7 Hz), 141.6 (d, *J* = 2.2 Hz), 139.6 (d, *J* = 2.8 Hz), 129.9 (d, J = 2.8 Hz) 129.2, 128.9 (d, *J* = 2.7 Hz), 128.6, 128.4, 128.2, 127.3, 82.0 (d, *J* = 163.0 Hz), 61.1, 13.6; **^{19}F NMR** (376 MHz, $CDCl_3$) δ -203.8 (t, *J* = 47.7 Hz).

HRMS (ESI): calculated for $C_{18}H_{17}O_2F_2Na^+$ $[M+Na]^+$ m/z: 307.1105, found: 307.1106.

(*E*)-Ethyl-2-(fluoromethyl)-6,6-dimethyl-3-phenylhept-2-en-4-ynoate (3aa)

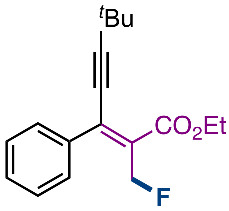

Prepared according to general procedure C using Enynes **1aa** (74 mg, 0.4 mmol), reagent **2a** (120 mg, 0.2 mmol) and $Et_3N{\bullet}3HF$ (96 mg, 0.6 mmol). Ratio of *linear*:*branched* isomers was determined to be > 20:1 and ratio of *Z*:*E* isomers in the *linear* isomer was determined to be 1:5 from the crude reaction mixture using ^{19}F-NMR spectroscopy. Purification by flash chromatography on silica gel (hexane/ethyl acetate = 30/1) provided the title compound as yellowish oil (46 mg, 82%).

^{1}H NMR (500 MHz, $CDCl_3$) δ 7.45 – 7.38 (m, 5H), 5.03 (d, *J* = 47.3 Hz, 2H), 4.36 (q, *J* = 7.1 Hz, 2H), 1.41 (t, *J* = 7.1 Hz, 3H), 1.28 (s, 9H); **^{13}C NMR** (101 MHz, $CDCl_3$) δ 166.8 (d, *J* = 1.0 Hz), 138.0 (d, *J* = 2.9 Hz), 137.8 (d, *J* = 8.9 Hz), 129.1, 128.9, 128.9, 128.3, 112.3 (d, *J* =

4.1 Hz), 79.7 (d, J = 163.4 Hz), 79.2 (d, J = 4.7 Hz), 61.2, 30.6, 30.5, 14.3; **^{19}F NMR** (376 MHz, $CDCl_3$) δ -203.0 (t, J = 47.3 Hz).

HRMS (ESI): calculated for $C_{18}H_{21}O_2FNa^+$ $[M+Na]^+$ m/z: 311.1423, found: 311.1421.

Ethyl 2-(1-fluorocyclobutyl)acrylate (3ab)

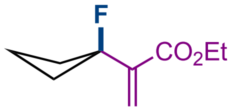

Prepared according to general procedure C using alkene **1ab** (30 mg, 0.4 mmol), reagent **2a** (120 mg, 0.2 mmol) and Et_3N•3HF (96 mg, 0.6 mmol). Ratio of *branched*:*linear* isomers was determined to be > 20:1 from the crude reaction mixture using ^{19}F-NMR spectroscopy. Purification by flash chromatography on silica gel (hexane/ethyl acetate = 50/1) provided the title compound as colorless oil (26 mg, 66% yield).

^{1}H NMR (400 MHz, CDCl3) δ 6.32 (d, J = 0.9 Hz, 1H), 5.86 (dd, J = 3.4, 0.9 Hz, 1H), 4.25 (q, J = 7.1 Hz, 2H), 2.61 – 2.41 (m, 4H), 2.06 – 1.95 (m, 1H), 1.73 – 1.61 (m, 1H), 1.32 (t, J = 7.1 Hz, 3H); **^{13}C NMR** (101 MHz, $CDCl_3$) δ 165.4 (d, J = 2.3 Hz), 140.5 (d, J = 22.7 Hz), 125.4 (d, J = 8.7 Hz), 96.7 (d, J = 204.1 Hz), 60.9, 33.6 (d, J = 23.2 Hz), 14.3, 13.0 (d, J = 7.4 Hz); **^{19}F NMR** (376 MHz, $CDCl_3$) δ -131.6 (m).

HRMS (ESI): calculated for $C_9H_{13}O_2FNa^+$ $[M+Na]^+$ m/z: 195.0792, found: 195.0788.

Ethyl-2-(1-fluorocyclohexyl) acrylate (3ac)

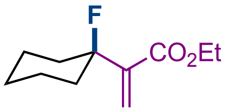

Prepared according to general procedure C using alkene **1ac** (39 mg, 0.4 mmol), reagent **2a** (120 mg, 0.2 mmol) and Et_3N•3HF (96 mg, 0.6 mmol). Ratio of *branched*:*linear* isomers was determined to be > 20:1 from the crude reaction mixture using ^{19}F-NMR spectroscopy. Purification by flash chromatography on silica gel (hexane/ethyl acetate = 40/1) provided the title compound as colorless oil (35 mg, 88% yield).

^{1}H NMR (400 MHz, $CDCl_3$) δ 6.18 (dd, J = 6.0, 1.3 Hz, 1H), 5.93 (d, J = 1.3 Hz, 1H), 4.20 (q, J = 7.1 Hz, 2H), 2.08 (dddd, J = 44.8, 14.0, 12.6, 5.8 Hz, 2H), 1.79 – 1.61 (m, 7H), 1.31 (t, J = 7.1 Hz, 3H), 1.27 – 1.24 (m, 1H); **^{13}C NMR** (101 MHz, $CDCl_3$) δ 165.7 (d, J = 8.4 Hz), 144.8 (d, J = 21.8 Hz), 123.9 (d, J = 14.9 Hz), 95.8 (d, J = 175.6 Hz), 60.8, 34.3 (d, J = 22.6 Hz), 24.7, 21.7 (d, J = 1.7 Hz), 14.3; **^{19}F NMR** (376 MHz, $CDCl_3$) δ -155.6 (m).

HRMS (ESI): calculated for $C_{11}H_{17}O_2FNa^+$ $[M+Na]^+$ m/z: 223.1092, found: 223.1100.

Ethyl 2-(1-fluoro-3,3-dimethylcyclohexyl)acrylate ((±)-3ad)

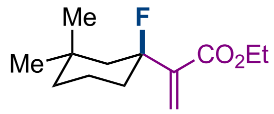

Prepared according to general procedure C using alkene **1ad** (39 mg, 0.4 mmol), reagent **2a** (120 mg, 0.2 mmol) and Et3N•3HF (96 mg, 0.6 mmol). Ratio of *branched*:*linear* isomers was determined to be > 20:1 from the crude reaction mixture using ^{19}F-NMR spectroscopy. Purification by flash chromatography on silica gel (hexane/ethyl acetate = 40/1) provided the title compound as colorless oil (35 mg, 73% yield).

^{1}H NMR (400 MHz, CDCl3) δ 6.20 (dd, *J* = 6.4, 1.3 Hz, 1H), 5.96 (dd, *J* = 1.3, 0.6 Hz, 1H), 4.28 – 4.17 (m, 2H), 2.17 – 1.89 (m, 2H), 1.87 – 1.71 (m, 2H), 1.68 – 1.51 (m, 3H), 1.51 – 1.43 (m, 1H), 1.33 (t, *J* = 7.1 Hz, 3H), 1.08 (d, *J* = 2.2 Hz, 3H), 0.95 (s, 3H); **^{13}C NMR** (101 MHz, $CDCl_3$) δ 165.8 (d, *J* = 6.8 Hz), 144.8 (d, *J* = 18.0 Hz), 124.1 (d, *J* = 12.5 Hz), 97.1 (d, *J* = 143.2 Hz), 60.8, 45.7 (d, *J* = 16.9 Hz), 38.0, 34.2, 34.0 (d, *J* = 18.6), 30.7 (d, *J* = 1.3 Hz), 26.7 (d, *J* = 5.1 Hz), 18.3, 14.2; **^{19}F NMR** (376 MHz, $CDCl_3$) δ -155.6 (m).

HRMS (ESI): calculated for $C_{13}H_{21}O_2FNa^+$ $[M+Na]^+$ m/z: 251.1418, found: 251.1420.

Ethyl 3-fluoro-3-methyl 2-methylene-5-phenylpentanoate ((±)-3ae)

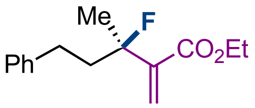

Prepared according to general procedure C using alkene **1ae** (59 mg, 0.4 mmol), reagent **2a** (120 mg, 0.2 mmol) and Et_3N•3HF (96 mg, 0.6 mmol). Ratio of *branched*:*linear* isomers was determined to be > 20:1 from the crude reaction mixture using ^{19}F-NMR spectroscopy. Purification by flash chromatography on silica gel (hexane/ethyl acetate = 30/1) provided the title compound as colorless oil (29 mg, 58% yield).

^{1}H NMR (400 MHz, $CDCl_3$) δ 7.32 – 7.22 (m, 2H), 7.20 – 7.16 (m, 3H), 6.33 (dd, *J* = 6.4, 1.4 Hz, 1H), 6.04 – 6.02 (m, 1H), 4.23 (q, *J* = 7.1 Hz, 2H), 2.79 – 2.63 (m, 1H), 2.57 – 2.47 (m, 1H), 2.47 – 2.31 (m, 1H), 2.26 – 2.08 (m, 1H), 1.64 (d, *J* = 23.2 Hz, 3H), 1.33 (t, *J* = 7.1 Hz, 3H); **^{13}C NMR** (101 MHz, $CDCl_3$) δ 165.3 (d, *J* = 9.8 Hz), 142.8 (d, *J* = 22.3 Hz), 142.0, 128.5, 128.5, 125.9, 125.0 (d, *J* = 15.1 Hz), 96.9 (d, *J* = 176.5 Hz), 60.9, 40.8 (d, *J* = 22.4 Hz), 30.2 (d, *J* = 3.3 Hz), 26.3 (d, *J* = 24.3 Hz), 14.3; **^{19}F NMR** (376 MHz, $CDCl_3$) δ -144.9 (m).

HRMS (ESI): calculated for $C_{15}H_{19}O_2FNa^+$ $[M+Na]^+$ m/z: 273.1261, found: 273.1265.

Ethyl 3-fluoro-3-mEthyl 2-methylene-5-(tosyloxy)pentanoate ((±)-3af)

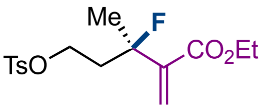

Prepared according to general procedure C using alkene **1af** (96 mg, 0.4 mmol), reagent **2a** (120 mg, 0.2 mmol) and Et_3N•3HF (96 mg, 0.6 mmol). Ratio of *branched*:*linear* isomers was determined to be 14:1

from the crude reaction mixture using ^{19}F-NMR spectroscopy. Purification by flash chromatography on silica gel (hexane/ethyl acetate = 15/1) provided the title compound as colorless oil (35 mg, 51% yield).

^{1}H NMR (400 MHz, $CDCl_3$) δ 7.77 – 7.74 (m, 2H), 7.35 – 7.26 (m, 2H), 6.22 (dd, *J* = 6.6, 1.2 Hz, 1H), 5.86 – 5.84 (m, 1H), 4.19 (q, *J* = 7.1 Hz, 2H), 4.13 – 3.96 (m, 2H), 2.71 – 2.46 (m, 1H), 2.45 (s, 3H), 2.17 – 2.12 (m, 1H), 1.58 (d, *J* = 23.4 Hz, 3H), 1.31 (t, *J* = 7.1 Hz, 3H); **^{13}C NMR** (101 MHz, $CDCl_3$) δ 164.8 (d, *J* = 9.6 Hz), 144.8, 141.7 (d, *J* = 21.8), 133.1, 129.8, 128.1, 125.2, (d, *J* = 15.1Hz), 95.2 (d, *J* = 177.0 Hz), 66.0 (d, *J* = 3.8 Hz), 61.2, 37.7 (d = 21.9 Hz), 26.6 (d, *J* = 23.9 Hz), 21.8, 14.2; **^{19}F NMR** (376 MHz, $CDCl_3$) δ -144.7 (m).

HRMS (ESI): calculated for $C_{16}H_{21}O_5SFNa^+$ $[M+Na]^+$ m/z: 367.0986, found: 367.0987.

Ethyl 5-(1,3-dioxoisoindolin-2-yl)-3-fluoro-3-methyl-2-methylenepentanoate ((±)-3ag)

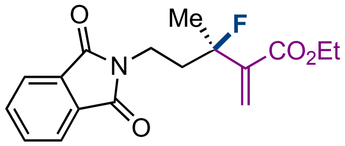

Prepared according to general procedure C using alkene **1ag** (86 mg, 0.4 mmol), reagent **2a** (120 mg, 0.2 mmol) and Et_3N•3HF (96 mg, 0.6 mmol). Ratio of *branched*:*linear* isomers was determined to be 14:1 from the crude reaction mixture using ^{19}F-NMR spectroscopy. Purification by flash chromatography on silica gel (hexane/ethyl acetate = 5/1) provided the title compound as colorless oil (27 mg, 41% yield).

^{1}H NMR (400 MHz, $CDCl_3$) δ 7.82 (dd, *J* = 5.4, 3.0 Hz, 2H), 7.73 – 7.65 (m, 2H), 6.32 (dd, *J* = 6.5, 1.2 Hz, 1H), 6.04 – 6.02 (m, 1H), 4.22 – 4.09 (m, 2H), 3.81 – 3.63 (m, 2H), 2.63 – 2.47 (m, 1H), 2.33 – 2.22 (m, 1H), 1.64 (d, *J* = 23.3 Hz, 3H), 1.27 (t, *J* = 7.1 Hz, 3H); **^{13}C NMR** (101 MHz, $CDCl_3$) δ 168.2, 165.0 (d, *J* = 9.7 Hz), 141.7 (d, *J* = 23.1 Hz), 134.0, 132.3, 125.8 (d, *J* = 15.2 Hz), 123.3, 96.0 (d, *J* = 176.8 Hz), 61.0, 36.9 (d, *J* = 21.7 Hz), 33.5 (d, *J* = 4.3 Hz), 26.4 (d, *J* = 23.9 Hz), 14.2; **^{19}F NMR** (376 MHz, CDC13) δ -146.3 (m).

HRMS (ESI): calculated for $C_{17}H_{18}FNO_4Na^+$ $[M+Na]^+$ m/z: 342.1112, found: 342.1125.

Ethyl 3-((2*R*,8*R*,8a*S*)-8,8a-dimethyl-1,2,3,4,6,7,8,8a-octahydronaphthalen-2-yl)-3-fluoro-2-methylenebutanoate (3ah)

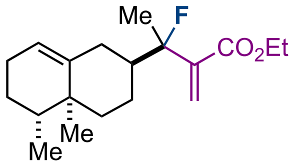

Prepared according to the modified general procedure C using valencene **1ah** (82 mg, 0.4 mmol), reagent **2a** (120 mg, 0.2 mmol) and Et_3N•3HF (96 mg, 0.6 mmol). The addition of **2a** was carried out at -70 ºC instead of -50 ºC. Ratio of *branched*:*linear* isomers was determined to be > 20:1 and the ratio of *diastereoisomers* was determined to be 1.6:1 from the crude reaction mixture using ^{19}F-NMR spectroscopy. Purification by flash chromatography on

silica gel (hexane/ethyl acetate = 30/1) provided the title compound as colorless oil (35 mg, 53% yield).

^{1}H NMR (500 MHz, $CDCl_3$) δ 6.25 – 6.21 (m, 1H), 5.94 – 5.86 (m, 1H), 5.32 – 5.29 (m, 1H), 4.29 – 4.14 (m, 2H), 2.36 – 2.16 (m, 2H), 2.15 – 1.88 (m, 4H), 1.64 – 1.51 (m, 5H), 1.45 – 1.36 (m, 3H), 1.35 – 1.18 (m, 4H), 1.13 – 0.95 (m, 1H), 0.95 – 0.82 (m, 5H); **^{13}C NMR** (101 MHz, $CDCl_3$) δ 165.6* (d, *J* = 9.7 Hz) 165.6 (d, *J* = 9.6 Hz), 147.8* (d, *J* = 22.6 Hz), 143.5 (d, *J* = 22.8 Hz), 142.9, 142.9*, 124.5 (d, *J* = 15.3 Hz), 124.2* (d, *J* = 15.3 Hz), 120.2*, 120.2, 98.7 (d, *J* = 179.6 Hz), 98.6* (d, *J* = 180.0 Hz), 60.9*, 60.8, 41.2, 41.0*, 40.2, 40.2*, 39.1* (d, *J* = 24.8 Hz), 38.9 (d, *J* = 21.3 Hz), 39.0*, 37.7, 37.6*, 32.5, 28.7 (d, *J* = 2.7 Hz), 27.4* (d, *J* = 3.8 Hz), 27.4, 27.3*, 26.0, 26.0*, 23.9* (d, *J* = 25.0 Hz), 23.6 (d, *J* = 24.6 Hz), 18.6, 18.3*, 15.9, 15.8*, 14.3*, 14.3; **^{19}F NMR** (376 MHz, $CDCl_3$) δ -156.6 (m), -157.6 (m)*; * *indicates the signals of the minor diastereoisomer.*

HRMS (ESI): calculated for $C_{19}H_{29}FO_2Na^+$ [M+Na]$^+$ m/z: 331.2044, found: 331.2032.

Ethyl 3-((3*S*,8*S*,9*S*,10*R*,13*S*,14*S*,17*S*)-3-acetoxy-10,13-dimethyl-2,3,4,7,8,9,10,11,12,13,14,15,16,17-tetradecahydro-1H-cyclopenta[a]phenanthren-17-yl)-3-fluoro-2-methylenebutanoate (3ai)

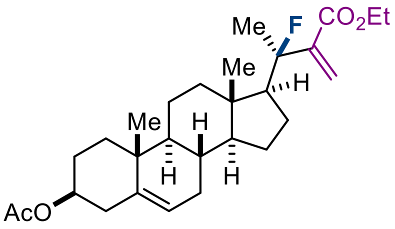

Prepared according to general procedure C using alkene **1ai** (142 mg, 0.4 mmol), reagent **2a** (120 mg, 0.2 mmol) and Et_3N•3HF (96 mg, 0.6 mmol). Ratio of *branched:linear* isomers was determined to be > 20:1 from the crude reaction mixture using ^{19}F-NMR spectroscopy. Purification by flash chromatography on silica gel (hexane/ethyl acetate = 30/1) provided the title compound as colorless solid (35 mg, 38% yield).

^{1}H NMR (500 MHz, $CDCl_3$) δ 6.14 (dd, *J* = 7.4, 1.5 Hz, 1H), 5.85 (t, *J* = 1.4 Hz, 1H), 5.36 (dt, *J* = 3.9, 1.6 Hz, 1H), 4.65 – 4.56 (m, 1H), 4.22 – 4.14 (m, 2H), 2.34 – 2.29 (m, 2H), 2.18 – 2.06 (m, 2H), 2.02 (s, 3H), 1.98 – 1.92 (m, 1H), 1.86 (dt, *J* = 12.7, 3.1 Hz, 2H), 1.72 (s, 3H), 1.66 – 1.58 (m, 2H), 1.58 – 1.47 (m, 5H), 1.41 – 1.33 (m, 2H), 1.30 (t, *J* = 7.1 Hz, 3H), 1.17 – 1.05 (m, 3H), 1.02 (s, 3H), 0.99 – 0.93 (m, 1H), 0.87 (d, *J* = 4.2 Hz, 3H); **^{13}C NMR** (126 MHz, $CDCl_3$) δ 170.6, 165.6 (d, *J* = 9.7 Hz), 144.7 (d, *J* = 22.3 Hz), 139.8, 123.3 (d, *J* = 16.7 Hz), 122.6, 98.2 (d, *J* = 182.9 Hz), 74.1, 60.8, 57.1, 54.1 (d, *J* = 20.1 Hz), 50.1, 42.7, 39.9, 38.2, 37.1, 36.7, 31.8, 31.5, 27.9, 25.4 (d, *J* = 25.0 Hz), 23.6, 23.0 (d, *J* = 4.2 Hz), 21.6, 21.0, 19.4, 14.2, 13.2 (d, *J* = 6.0 Hz); **^{19}F NMR** (471 MHz, $CDCl_3$) δ -153.4 (m).

HRMS (ESI): calculated for $C_{28}H_{41}O_4FNa^+$ [M+Na]$^+$ m/z: 483.2881, found: 483.2877.

[α]$^{25}_{D}$ = -64.0 (*c* = 0.11, $CHCl_3$).

m.p. 111-113 ºC.

The crystal structure of the title compound has been deposited at the Cambridge Crystallographic Data Centre, CCDC No. 2092729.

Ethyl 2-((3*R,5*S*,8*R*,9*S*,10*S*,13*S*,14*S*,17*S*)-17-acetoxy-3-fluoro-10,13-imethylhexadecahydro-1H-cyclopenta[a]phenanthren-3-yl)acrylate (3aj)**

Prepared according to general procedure C using alkene **1aj** (132 mg, 0.4 mmol), reagent **2a** (120 mg, 0.2 mmol) and Et_3N•3HF (90 mg, 0.6 mmol). Ratio of *branched*:*linear* isomers was determined to be > 20:1 and the ratio of *diastereoisomers* was determined to be 1:1 from the crude reaction mixture using ^{19}F-NMR spectroscopy. Purification by flash chromatography on silica gel (hexane/ethyl acetate = 30/1) provided the title compound as colorless oil (39 mg, 45% yield).

(3*R*)-3aj

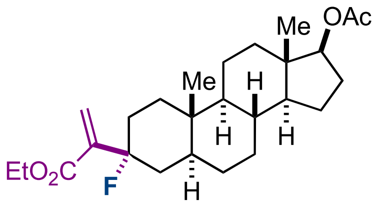

^{1}H NMR (500 MHz, $CDCl_3$) δ 6.30 – 6.20 (m, 1H), 5.98 (s, 1H), 4.59 (t, *J* = 8.5 Hz, 1H), 4.21 (q, *J* = 6.9 Hz, 2H), 2.40 – 2.20 (m, 1H), 2.20 – 2.07 (m, 2H), 2.03 (s, 3H), 1.78 – 1.61 (m, 4H), 1.58 – 1.53 (m, 2H), 1.52 – 1.38 (m, 3H), 1.38 – 1.11 (m, 10H), 1.10 – 1.01 (m, 1H), 0.90 (d, *J* = 6.2 Hz, 4H), 0.79 (d, *J* = 5.8 Hz, 4H); **^{13}C NMR** (126 MHz, $CDCl_3$), 171.2, 165.7 (d, *J* = 8.9 Hz), 144.2 (d, *J* = 21.8 Hz), 124.4 (d, *J* = 15.4 Hz), 96.3 (d, *J* = 176.7 Hz), 83.0, 60.9, 53.9, 50.9, 42.8, 41.0, 37.5 (d, *J* = 22.4 Hz), 37.1, 35.5, 33.9, 31.6, 30.6 (d, *J* = 23.2 Hz), 28.2, 27.7, 23.6, 21.3, 20.6, 14.3, 12.3, 11.4, 11.4; **^{19}F NMR** (376 MHz, $CDCl_3$) δ -150.7 (m); ^{1}H-^{13}C HSQC, ^{1}H-^{13}C HMBC, ^{1}H-^{19}F HOESY spectra were measured.

HRMS (ESI): calculated for $C_{26}H_{39}O_4FNa^+$ [M+Na]$^+$ m/z: 457.2725, found: 457.2730.

[α]$^{25}_{D}$ = +3.65 (*c* = 0.10, $CHCl_3$).

m.p. 118 – 120 ºC.

The crystal structure of title compound has been deposited at the Cambridge Crystallographic Data Centre, CCDC No. 2092730.

(3*S*)-3aj

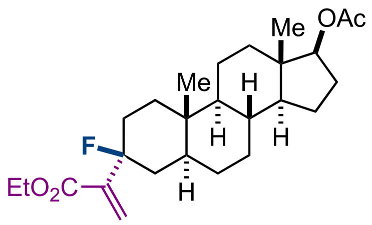

^{1}H NMR (400 MHz, $CDCl_3$) δ 6.31 (d, *J* = 1.1 Hz, 1H), 5.88 (d, *J* = 4.5 Hz, 1H), 4.57 (dd, *J* = 9.2, 7.8 Hz, 1H), 4.24 (q, *J* = 7.1 Hz, 2H), 2.40 – 2.29 (m, 1H), 2.25 – 2.18 (m, 1H), 2.17 – 2.09 (m, 1H), 2.03 (s, 3H), 1.98 – 1.89 (m, 1H), 1.84 – 1.58 (m, 6H), 1.51 – 1.44 (m, 2H), 1.44 – 1.37 (m, 1H), 1.31 (t, *J* = 7.1 Hz, 5H), 1.27 – 1.22 (m, 2H), 1.16 – 0.92 (m, 5H), 0.90 (s, 3H), 0.78 – 0.76 (m, 3H); **^{13}C NMR** (101 MHz, $CDCl_3$) δ 171.4, 166.4, 141.3 (d, *J* = 22.0 Hz), 127.1 (d, *J* = 7.2 Hz), 95.2 (d, *J* = 170.1 Hz), 82.9, 61.0, 54.5 (d, *J* = 2.4 Hz), 50.9, 43.8 (d, *J* = 8.7 Hz), 42.8, 37.2 (d, *J* = 20.6 Hz), 37.0, 36.9, 36.1 (d, *J* = 1.6 Hz), 35.3, 31.6, 31.1 (d, *J* = 21.4 Hz), 28.4, 27.7, 23.6, 21.3, 20.9, 14.3, 12.4, 12.3; **^{19}F NMR** (376 MHz, $CDCl_3$) δ -122.8 (m); .

HRMS (ESI): calculated for $C_{26}H_{39}O_4FNa^+$ [M+Na]$^+$ m/z: 457.2725, found: 457.2730.

[α]$^{25}_D$ = +13.44 (c = 0.185, $CHCl_3$).

Ethyl 3-fluoro-3-(4-methylcyclohex-3-en-1-yl)-2-methylenebutanoate (3ak)

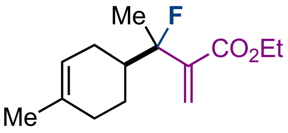

Prepared according to general procedure C using alkene **1ak** (55 mg, 0.4 mmol), reagent **2a** (120 mg, 0.2 mmol) and Et_3N•3HF (90 mg, 0.6 mmol). Ratio of *branched*:*linear* isomers was determined to be > 20:1 and the ratio of *diastereoisomers* was determined to be 1.1:1 from the crude reaction mixture using ^{19}F-NMR spectroscopy. Purification by flash chromatography on silica gel (hexane/ethyl acetate = 30/1) provided the title compound as colorless oil (31 mg, 58% yield). **^{1}H NMR** (400 MHz, $CDCl_3$) δ 6.28 – 6.23 (m, 1H), 5.91 (dt, *J* = 2.7, 1.1 Hz, 1H), 5.44 – 5.27 (m, 1H), 4.23 – 4.16 (m, 2H), 2.26 – 1.80 (m, 5H), 1.64 – 1.61 (m, 3H), 1.60 – 1.54 (m, 3H), 1.48 – 1.32 (m, 1H), 1.32 – 1.28 (m, 4H); **^{13}C NMR** (101 MHz, $CDCl_3$) δ 165.6 (d, *J* = 9.4 Hz), 165.4 (d, *J* = 9.6 Hz), 143.5 (d, *J* = 22.4 Hz), 143.3 (d, *J* = 22.5 Hz), 134.0, 133.8, 124.7 (d, *J* = 15.5 Hz), 124.4 (d, 15.7 Hz), 120.6, 120.4, 98.5 (d, *J* = 179.6Hz), 98.1 (d, *J* = 180.3 Hz), 60.9, 39.4 (d, *J* = 21.7 Hz), 39.3 (d, *J* = 21.5 Hz), 30.9, 30.8, 26.7 (d, *J* = 2.7 Hz), 25.5 (d, *J* = 3.8 Hz), 23.9, 23.6, 23.6, 23.4, 23.4, 23.4, 22.7, 22.6, 14.3, 14.3; **^{19}F NMR** (376 MHz, $CDCl_3$) δ -156.9 (m), -158.2 (m).

HRMS (ESI): calculated for $C_{14}H_{21}O_2FNa^+$ [M+Na]$^+$ m/z: 263.1418, found: 263.1414.

Ethyl 3-fluoro-3-((1*R*,3*S*,4*S*)-4-methyl-3-(prop-1-en-2-yl)-4-vinylcyclohexyl)-2-methylenebutanoate (3al)

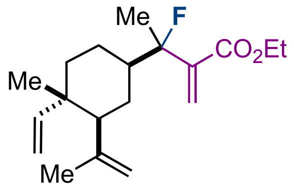

Prepared according to the modified general procedure C using β-elemene **1al** (82 mg, 0.4 mmol), reagent **2a** (120 mg, 0.2 mmol) and Et_3N•3HF (96 mg, 0.6 mmol). Ratio of *branched*:*linear* isomers was determined to be > 20:1 and the ratio of *diastereoisomers* was determined to be 1.3:1 from the crude reaction mixture using ^{19}F-NMR spectroscopy. Purification by flash chromatography on silica gel (hexane/ethyl acetate = 40/1) provided the title compound as colorless oil (37 mg, 57% yield).

^{1}H NMR (400 MHz, $CDCl_3$) δ 6.27 – 6.25 (m, 1H), 5.93 – 5.91 (m, 1H), 5.83 – 5.72 (m, 1H), 4.91 – 4.89 (m, 1H), 4.87 – 4.86 (m, 1H), 4.84 – 4.78 (m, 1H), 4.53 – 4.55 (m, 1H), 4.26 – 4.17 (m, 2H), 2.12 – 2.02 (m, 1H), 2.00 – 1.89 (m, 1H), 1.74 – 1.72 (m, 2H), 1.68 – 1.64 (m, 2H), 1.61 (d, *J* = 1.4 Hz, 2H), 1.57 – 1.51 (m, 2H), 1.49 – 1.36 (m, 3H), 1.34 – 1.28 (m, 4H), 0.97 (s, 3H); **^{13}C NMR** (101 MHz, $CDCl_3$) δ 165.5 (d, *J* = 9.8 Hz), 165.4 (d, *J* = 9.8 Hz), 150.3, 150.2, 148.0, 147.9, 124.7 (d, *J* = 15.7 Hz), 124.5 (d, *J* = 15.6 Hz), 112.3, 112.2, 110.1 (2C), 98.5 (2C) (d, *J* = 180.0 Hz), 60.9 (2C), 52.7, 52.5, 43.2 (d, *J* = 21.3 Hz), 43.9 (d, *J* = 21.3 Hz), 39.7, 39.7, 39.7, 39.7, 28.1, 28.1, 27.1, 27.0, 24.9, 24.9, 23.8, 23.6, 22.4 (d, *J* = 3.1 Hz), 21.2 (d, *J* = 3.6 Hz), 16.5, 16.5, 14.3, 14.3; **^{19}F[^{1}H] NMR** (376 MHz, $CDCl_3$) δ -156.4, -157.5; **^{19}F NMR** (376 MHz, $CDCl_3$) δ -156.5 (m, 2F); ^{1}H-^{13}C HSQC and ^{1}H-^{13}C HMBC spectra were measured.

HRMS (ESI): calculated for $C_{19}H_{29}O_2FNa^+$ $[M+Na]^+$ m/z: 331.2044, found: 331.2043.

Ethyl 2-((3*R,8*R*,10*S*,13*R*,17*R*)-3-fluoro-10,13-dimethyl-17-((*R*)-5-methylhexan-2-yl)hexadecahydro-1H-cyclopenta[a]phenanthren-3-yl)acrylate (3am)**

Prepared according to general procedure C using alkene **1am** (153 mg, 0.4 mmol), reagent **2b** (120 mg, 0.2 mmol) and Et_3N•3HF (96 mg, 0.6 mmol). Due to the poor solubility of starting alkene, 10 mL of CH_2Cl_2 was employed to dissolve rhodium catalyst and alkene. Ratio of *branched*:*linear* isomers was determined to be > 20:1 and the ratio of *diastereoisomers* was determined to be 1:1 from the crude reaction mixture using ^{19}F-NMR spectroscopy. Purification by flash chromatography on silica gel (hexane/ethyl acetate = 30/1) provided the title compounds as white solid (37 mg, 41% yield).

(3*R*)-3am

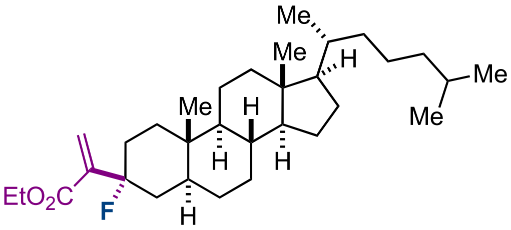

^{1}H NMR (400 MHz, $CDCl_3$) δ 6.21 (dd, *J* = 6.5, 1.5 Hz, 1H), 5.97 (d, *J* = 1.4 Hz, 1H), 4.21 (q, *J* = 7.2 Hz, 2H), 2.37 – 2.21 (m, 1H), 2.19 – 2.04 (m, 1H), 1.97 (dt, *J* = 12.7, 3.5 Hz, 1H), 1.84 – 1.77 (m, 1H), 1.68 – 1.58 (m, 3H), 1.54 – 1.48 (m, 3H), 1.39 – 1.30 (m, 10H), 1.26 – 1.20 (m, 4H), 1.15 – 1.09 (m, 6H), 1.04 – 0.97 (m, 3H), 0.92 – 0.85 (m, 13H), 0.66 (s, 3H); **^{13}C NMR** (126 MHz, $CDCl_3$) δ 165.8 (d, *J* = 8.9 Hz), 144.3 (d, *J* = 22.0 Hz), 124.3 (d, *J* = 15.4 Hz), 96.4 (d, *J* = 176.4 Hz), 60.9, 56.7, 56.4, 53.9, 42.8, 41.0, 40.2, 39.7, 37.6 (d, *J* = 22.5 Hz), 36.3, 36.0, 35.7, 35.7, 33.9, 32.1, 30.7 (d, *J* = 23.6 Hz), 28.5, 28.4, 28.2, 24.4, 24.0, 23.0, 22.7, 21.1, 18.8, 14.3, 12.2, 11.4; **^{19}F NMR** (376MHz, $CDCl_3$) δ -150.6 (ttd, *J* = 44.4, 12.3, 6.3 Hz).

HRMS (ESI): calculated for $C_{32}H_{53}O_2FNa^+$ $[M+Na]^+$ m/z: 511.3922, found: 511.3916.

[α]$^{25}_{D}$ = +23.5 (*c* = 0.175, $CHCl_3$).

(3*S*)-3am

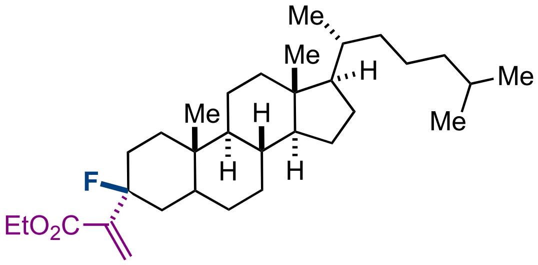

^{1}H NMR (400 MHz, $CDCl_3$) δ 6.31 (d, *J* = 1.1 Hz, 1H), 5.89 (d, *J* = 4.5 Hz, 1H), 4.28 – 4.21 (m, 2H), 2.33 (d, *J* = 13.7 Hz, 1H), 2.21 – 2.16 (m, 2H), 1.96 (d, *J* = 9.4 Hz, 1H), 1.90 – 1.62 (m, 5H), 1.58 – 1.52 (m, 2H), 1.51 – 1.23 (m, 15H), 1.15 – 0.96 (m, 10H), 0.91 – 0.82 (m, 13H); **^{13}C NMR** (101 MHz, $CDCl_3$), δ 166.4, 141.4 (d, *J* = 21.8 Hz), 127.1 (d, *J* = 7.6 Hz), 95.4 (d, *J* = 169.8 Hz), 61.0, 56.6, 56.4, 54.5 (d, *J* = 2.3 Hz), 43.8 (d, *J* = 8.6 Hz), 42.8, 40.1, 39.7, 37.2 (d, *J* = 20.5 Hz), 36.9 (d, *J* = 10.0 Hz), 36.3, 36.0 (d, *J* = 1.6 Hz), 35.9, 35.5, 32.1, 31.2 (d, *J* = 21.6 Hz), 28.6, 28.4, 28.2, 24.3, 24.0, 23.0, 22.7, 21.4, 18.8, 14.3, 12.4, 12.2; **^{19}F NMR** (471MHz, $CDCl_3$) δ -122.6 (m).

HRMS (ESI): calculated for $C_{32}H_{53}O_2FNa^+$ $[M+Na]^+$ m/z: 511.3922, found: 511.3916.

[α]$^{25}_{D}$ = +12.5 (*c* = 0.15, $CHCl_3$).

Ethyl 3-fluoro-3-(4-(((*S*)-2-(4-isobutylphenyl)propanoyl)oxy)phenyl)-2-methylenebutanoate (3an)

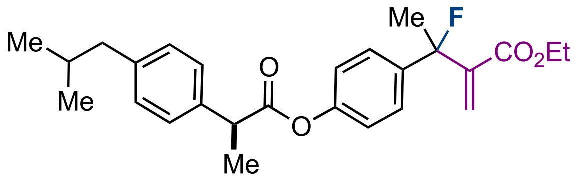

Prepared according to general procedure C using alkene **1an** (129 mg, 0.4 mmol), reagent **2a** (120 mg, 0.2 mmol) and Et_3N•3HF (96 mg, 0.6 mmol). Ratio of *branched*:*linear* isomers was determined to be > 20:1 and the ratio of *diastereoisomers* was determined to be 1:1 from the crude reaction mixture using ^{19}F-NMR spectroscopy. Purification by flash chromatography on silica gel (hexane/ethyl acetate = 30/1) provided the title compound as colorless oil (53 mg, 62% yield).

^{1}H NMR (400 MHz, $CDCl_3$), δ 7.43 – 7.37 (m, 2H), 7.33 – 7.27 (m, 2H), 7.15 – 7.12 (m, 2H), 7.01 – 6.95 (m, 2H), 6.36 (dd, *J* = 4.4, 1.0 Hz, 1H), 6.10 (d, *J* = 1.0 Hz, 1H), 4.10 – 4.01 (m, 2H), 3.92 (q, *J* = 7.1 Hz, 1H), 2.47 (d, *J* = 7.2 Hz, 2H), 2.02 (s, 3H), 1.86 (dt, *J* = 13.5, 6.8 Hz, 1H), 1.60 (d, *J* = 7.1 Hz, 3H), 1.15 (t, *J* = 7.1 Hz, 3H), 0.92 (s, 3H), 0.90 (s, 3H); **^{13}C NMR** (101 MHz, $CDCl_3$) δ 173.2, 164.8 (d, *J* = 6.4 Hz), 150.7 (d, *J* = 2.6 Hz), 143.7 (d, *J* = 24.3 Hz), 141.0, 139.5 (d, *J* = 22.1 Hz), 137.3, 129.6, 127.3, 126.9 (d, *J* = 6.6 Hz), 124.9 (d, *J* = 11.8 Hz), 121.0, 95.6 (d, *J* = 174.6 Hz), 60.9, 45.4, 45.2, 30.3, 25.9 (d, *J* = 24.8 Hz), 22.5, 18.6, 14.3; **^{19}F[^{1}H] NMR** (376 MHz, CD_2Cl_2) δ -131.4, -131.4; **^{19}F NMR** (376 MHz, $CDCl_3$) δ -131.8 (m, 2F).

HRMS (ESI): calculated for $C_{26}H_{31}O_4FNa^+$ $[M+Na]^+$ m/z: 449.2099, found: 449.2089.

6. Derivatizations of (±)-3a.

Ethyl 3-fluoro-2-methyl-3-phenylbutanoate (6)[21]

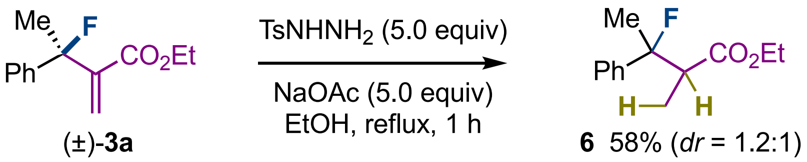

To an oven-dried reaction tube was added **3a** (88 mg, 0.4 mmol), $TsNHNH_2$ (360 mg, 2.0 mmol), NaOAc (160 mg, 2.0 mmol) and anhydrous EtOH (5 mL). The resulting reaction mixture was heated to reflux during 1 hour. After this, the reaction was quenched with water and (30 mL) extracted with CH_2Cl_2 (3 x 10 mL). The combined organic layers were washed with brine, dried over Na_2SO_4 and the solvent was removed under *vacuum*. Ratio of *diastereoisomers* was determined to be 1.2:1 from the crude reaction mixture using ^{19}F-NMR

spectroscopy. Purification by flash chromatography on silica gel (hexane/ethyl acetate = 40/1) provided the title compound as colorless oil (50 mg, 58% yield).

^{1}H NMR (400 MHz, $CDCl_3$) δ 7.43 – 7.26 (m, 5H), 4.15 – 3.97 (m, 2H), 3.04 – 2.90 (m, 1H), 1.80 – 1.73 (m, 3H), 1.18 – 1.10 (m, 6H); **^{13}C NMR** (101 MHz, $CDCl_3$), 173.05 (d, J = 6.1 Hz), 173.0* (d, J = 3.5 Hz), 143.4 (d, J = 21.9 Hz), 142.9* (d, J = 22.1 Hz), 128.3 (d, J = 1.7 Hz), 128.2* (d, J = 0.9 Hz), 127.8 (d, J = 1.5 Hz), 127.7* (d, J = 1.1 Hz), 125.0* (d, J = 9.2 Hz), 124.5 (d, J = 9.7 Hz), 97.4* (d, J = 179.0 Hz), 96.9 (d, J = 181.5), 60.6, 60.6*, 50.6*, 50.3, 24.4 (d, J = 24.5 Hz), 23.5* (d, J = 24.5 Hz), 14.2, 14.1*, 12.5* (d, J = 6.5 Hz), 12.3 (d, J = 4.3 Hz); **^{19}F NMR** (376 MHz, $CDCl_3$) δ -146.9* (qd, J = 23.2, 15.6 Hz), -153.6 (m); * *indicates the signals of the minor diastereoisomer*

HRMS (ESI): calculated for $C_{13}H_{17}O_2FNa^+$ $[M+Na]^+$ m/z: 247.1105, found: 247.1116.

Note: The product contains some impurities that were unable to remove using flash chromatographic column and preparative TLC.

Ethyl 3-fluoro-2-hydroxy-2-(hydroxymethyl)-3-phenylbutanoate (7)[22]

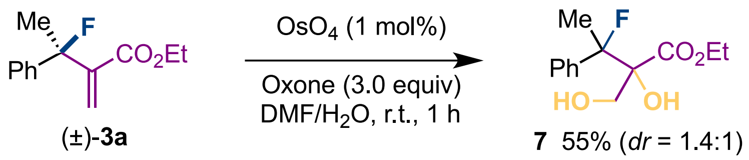

To an oven-dried reaction tube was added **3a** (80 mg, 0.4 mmol), DMF (2.0 mL), H_2O (2.0 mL) and OsO_4 (1 mg, 1.0 mol%). After 5 minutes, Oxone (180 mg, 1.2 mmol) was added in one portion and the reaction was stirred during 1 hour at room temperature. The reaction mixture was quenched with a saturated aqueous solution of $Na_2S_2O_3$ (10 mL) and stirred for 1 hour. After this, the reaction mixture was extracted with ethyl acetate (3 x 15 mL), washed with brine, dried over Na_2SO_4 and the solvent was removed under *vacuum*. Ratio of *diastereoisomers* was determined to be 1.4:1 from the crude reaction mixture using ^{19}F-NMR spectroscopy. Purification by flash chromatography on silica gel (hexane/ethyl acetate = 2/1) provided the title compound as colorless oil (56 mg, 70% yield).

^{1}H NMR (400 MHz, $CDCl_3$) δ 7.39 – 7.29 (m, 5H), δ 7.39 – 7.29 (m, 5H*), 4.29 (q, J = 7.1 Hz, 2H*), 4.18 (dd, J = 11.4, 2.8 Hz, 1H), 4.13 – 4.03 (m, 1H*), 4.11 – 4.05 (m, 2H), 3.93 (dd, J = 11.4, 0.9 Hz, 1H), 3.55 (dd, J = 11.4, 0.8 Hz, 1H*), 1.79 (d, J = 23.7 Hz, 3H), 1.74 (d, J = 23.8 Hz, 3H*), 1.29 (t, J = 7.1 Hz, 3H*), 1.20 (d, J = 7.1 Hz, 3H); **^{13}C NMR** (101 MHz, $CDCl_3$) δ 173.0 (2C), 140.8 (d, J = 22.2 Hz), 140.3* (d, J = 22.0 Hz), 128.2 (d, J = 0.9 Hz), 128.2* (d, J = 0.9 Hz), 128.1 (d, J = 1.8 Hz), 128.0* (d, J = 1.8 Hz), 125.6* (d, J = 10.7 Hz),

125.0 (d, J = 10.5 Hz), 97.4 (d, J = 184.4 Hz), 97.3* (d, J = 183.7 Hz), 81.9* (d, J = 26.0 Hz), 81.6 (d, J = 27.4 Hz), 63.8* (d, J = 3.3 Hz), 63.6 (d, J = 5.4 Hz), 63.1*, 62.9 23.2* (d, J = 23.4 Hz), 22.9 (d, J = 23.0 Hz), 14.1*, 14.1; **^{19}F NMR** (101 MHz, $CDCl_3$) δ -155.2* (qd, J = 24.0, 3.7 Hz), -156.7 (qd, J = 23.7, 3.1 Hz); * *indicates the signals of the minor diastereoisomer.*
HRMS (ESI): calculated for $C_{13}H_{17}O_4FNa^+$ $[M+Na]^+$ m/z: 279.1003, found: 279.0996.

Ethyl 2-(1-fluoro-1-phenylethyl)oxirane-2-carboxylate (8)[23]

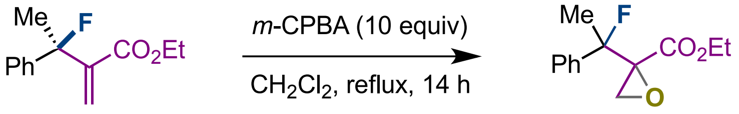

(±)-**3a** **8** 51% (*dr* = 3:1)

To an oven-dried reaction tube was added **3a** (44 mg, 0.2 mmol), CH_2Cl_2 (4.0 mL) and *meta*-chloroperoxybenzoic acid (*m*-CPBA) (254 mg, 2.0 mmol, 10.0 equiv.). The reaction mixture was heated to reflux during 14 hours. After this, the reaction was quenched with a saturated aqueous solution of $Na_2S_2O_3$ (10 mL) and stirred for 1 hour. The organic layer was separated, and the aqueous solution was extracted with CH_2Cl_2 (3 x 10 mL) washed with brine, dried over Na_2SO_4 and the solvent was removed under *vacuum*. Ratio of *diastereoisomers* was determined to be 3:1 from the crude reaction mixture using ^{19}F-NMR spectroscopy. Purification by flash chromatography on silica gel (hexane/ethyl acetate = 20/1) provided the title compound as colorless oil (71 mg, 51% yield).
^{1}H NMR (400 MHz, $CDCl_3$,) δ 7.48 – 7.45 (m, 2H), 7.40-7.30 (m, 3H), 4.13 – 3.98 (m, 2H), 3.28 – 3.06 (m, 2H), 1.93 – 1.82 (m, 3H), 1.15 – 1.03 (m, 3H). **^{13}C NMR** (101 MHz, $CDCl_3$) δ 167.5 (d, J = 3.8 Hz, 2C), 140.8 (d, J = 22.1 Hz), 140.4* (d, J = 22.5 Hz), 128.8*, 128.5 (d, J = 1.7 Hz), 128.4* (d, J = 1.4 Hz), 128.3, 128.3* (d, J = 4.7 Hz), 125.5 (d, J = 7.6 Hz), 94.6 (d, J = 180.4 Hz), 94.3* (d, J = 179.5 Hz), 61.9*, 61.8, 60.8* (d, J = 28.0 Hz), 60.7 (d, J = 29.2 Hz), 49.7* (d, J = 5.7 Hz), 49.2 (d, J = 6.6 Hz), 24.0* (d, J = 24.5 Hz), 22.5 (d, J = 24.5 Hz), 13.9*, 13.8; **^{19}F NMR** (376 MHz, $CDCl_3$) δ -147.8 (q, J = 23.2 Hz), -150.8* (q, J = 23.7 Hz); * *indicates the signals of the minor diastereoisomer.*
HRMS (ESI): calculated for $C_{13}H_{15}O_3FNa^+$ $[M+Na]^+$ m/z: 261.0897, found: 261.0891.

Ethyl 5-(1-fluoro-1-phenylethyl)-3-phenyl-4,5-dihydroisoxazole-5-carboxylate (9)[24]

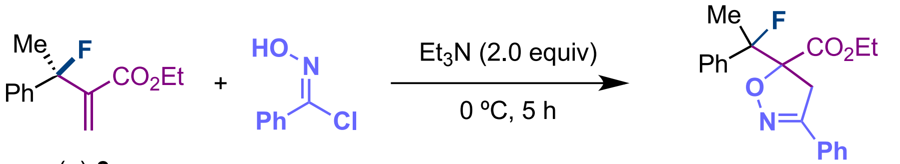

To a 10 mL oven-dried reaction tube equipped with a stirring bar was added chlorobenzaldoxime (96 mg, 0.6 mmol) and CH_2Cl_2 (6.0 mL). The reaction mixture was cooled at 0°C and triethylamine (90 µL, 0.6 mmol) was added. After stirring for 10 min, tertiary allylic fluoride **3a** (66 mg, 0.3 mmol) was added dropwise. The mixture was stirred at 0 °C for 5 h. Then the reaction was quenched with water (5 mL) and the organic layer was separated. The aqueous layer was extracted with CH_2Cl_2 (3 x 10 mL), the combined organic layers were dried over Na_2SO_4 and the solvent was removed under *vacuum*. Ratio of *diastereoisomers* was determined to be 2.5:1 from the crude reaction mixture using ^{19}F-NMR spectroscopy. Purification by flash chromatography on silica gel (hexane/ethyl acetate = 10/1) provided the title compound as colorless oil (71 mg, 70% yield).

^{1}H NMR (400 MHz, $CDCl_3$) δ 7.69 – 7.67 (m, 2H), 7.50 – 7.26 (m, 8H), 7.50 – 7.26 (m, 10H*). 4.37 – 4.22 (m, 2H*), 4.09 – 3.96 (m, 2H), 3.87 – 3.81 (m, 2H), 3.78 – 3.77 (m, 1H), 3.81 (d, *J* = 1.5 Hz, 1H), 3.77 (dd, *J* = 4.6, 1.2 Hz, 1H*), 3.49 (d, *J* = 17.6 Hz, 1H*),1.95 (d, *J* = 23.8 Hz, 3H*), 1.91 (d, *J* = 23.7 Hz, 3H), 1.31 (t, *J* = 7.1 Hz, 3H*), 1.06 (d, *J* = 7.1 Hz, 3H); **^{13}C NMR** (101 MHz, $CDCl_3$) δ 170.4* (d, *J* = 1.1 Hz), 169.7 (d, *J* = 3.0 Hz), 156.8*, 156.7, 140.0 (d, *J* = 20.0 Hz), 139.3* (d, *J* = 21.7 Hz), 130.7, 130.5*, 128.9, 128.8*, 128.7, 128.6* 128.5, 128.3 (d, *J* = 1.9 Hz), 128.2* (d, *J* = 2.0 Hz), 127.0, 126.8*, 125.8* (d, *J* = 10.4 Hz), 125.1 (d, *J* = 10.1 Hz), 97.5 (d, *J* = 186.9 Hz), 96.8* (d, *J* = 184.8 Hz), 93.0 (d, *J* = 26.4 Hz), 92.3* (d, *J* = 28.7 Hz), 62.6*, 62.3, 41.2*, 40.5 (d, *J* = 4.7 Hz), 23.7* (d, *J* = 22.5 Hz), 22.6 (d, *J* = 23.3 Hz), 14.2*, 13.8 (one aromatic *C is missing); **^{19}F NMR** (376 MHz, $CDCl_3$) δ -153.8* (q, *J* = 23.8 Hz), -154.9 (q, *J* = 23.7 Hz); ** indicates the signals of the minor diastereoisomer.*

HRMS (ESI): calculated for $C_{20}H_{20}NO_3FNa^+$ $[M+Na]^+$ m/z: 364.1319, found: 364.1303.

2-Fluoro-2-phenylpropanoic acid (10)[22]

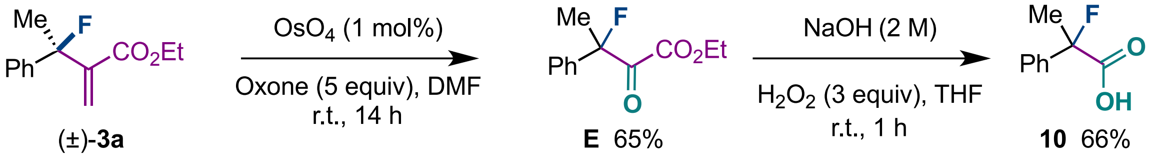

Synthesis of **E**: To an oven-dried reaction tube was added **3a** (80 mg, 0.4 mmol), DMF (2.0 mL) and OsO_4 (1 mg, 1.0 mol%). After 5 minutes, Oxone (300 mg, 2.0 mmol) was added in one portion and stirred for 14 hours, the reaction was quenched with saturated $Na_2S_2O_3$ aqueous solution (10 mL) and it was stirred for an additional hour. The reaction mixture was extracted with ethyl acetate (3 x 15 mL) and the combined organic layers were washed with brine, dried over Na_2SO_4 and the solvent was removed under *vacuum*. Purification by flash chromatography on silica gel (hexane/ethyl acetate = 30/1) provided **E** as colorless oil (52 mg, 65% yield).

^{1}H NMR (400 MHz, $CDCl_3$) δ 7.49 – 7.34 (m, 5H), 4.33 – 4.25 (m, 2H), 1.95 (d, *J* = 22.9 Hz, 3H), 1.29 (t, *J* = 7.2 Hz, 3H); **^{13}C NMR** (101 MHz, $CDCl_3$) δ 193.6 (d, *J* = 37.5 Hz), 162.2, 137.4 (d, *J* = 22.5 Hz), 129.0 (d, *J* = 1.2 Hz), 128.0 (d, *J* = 1.2 Hz), 124.8 (d, *J* = 8.6 Hz), 99.2 (d, *J* = 183.0 Hz), 62.6, 24.5 (d, *J* = 23.3 Hz), 14.0; **^{19}F NMR** (376 MHz, $CDCl_3$) δ -156.9 (q, *J* = 22.9 Hz).

HRMS (ESI): calculated for $C_{12}H_{13}O_3FNa^+$ $[M+Na]^+$ m/z: 247.0741, found: 247.0731.

Synthesis of **10**: To an oven-dried reaction tube equipped with stirring bar was added **E** (67 mg, 0.3 mmol), THF (2.0 mL) and NaOH (0.75 mL, 2M in water, 5.0 equiv.). The reaction mixture was cooled at 0 ºC and H_2O_2 (91 μL, 30% w/w, 3.0 equiv.) was added. Then, it was stirred for 1 hour at room temperature. After this time, the reaction was quenched with $Na_2S_2O_3$ saturated aqueous solution (1 mL), and it was stirred for an additional hour. The reaction mixture was then extracted with ethyl acetate (3 x 15 mL), the combined organic layers were washed with brine, dried over Na_2SO_4 and the solvent was removed under *vacuum*. Purification by flash chromatography on silica gel (hexane/ethyl acetate = 5/1 to 2/1) provided **10** as colorless oil (33 mg, 66% yield).

^{1}H NMR (400 MHz, $CDCl_3$) δ 9.42 (brs, 1H), 7.59 – 7.50 (m, 2H), 7.44 – 7.34 (m, 3H), 1.97 (d, *J* = 22.3 Hz, 3H); **^{13}C NMR** (101 MHz, $CDCl_3$) δ 176.6 (d, *J* = 28.1 Hz), 138.4 (d, *J* = 22.7 Hz), 129.1 (d, *J* = 1.5 Hz), 128.7 (d, *J* = 1.2 Hz), 124.8 (d, *J* = 8.5 Hz), 94.3 (d, *J* = 187.1 Hz), 24.6 (d, *J* = 23.7 Hz); **^{19}F NMR** (376 MHz, $CDCl_3$,) δ -151.3.

Spectra are consistent with previously reported.[25]

7. Synthesis of F-flurbiprofen 12.

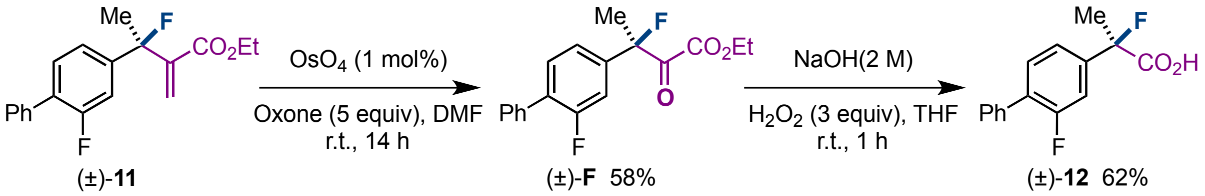

Ethyl 3-fluoro-3-(2-fluoro-[1,1'-biphenyl]-4-yl)-2-methylenebutanoate ((±)-11)

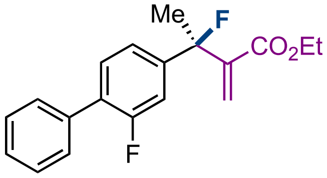

Prepared according to general procedure C using alkene **1ao** (85 mg, 0.4 mmol), reagent **2a** (120 mg, 0.2 mmol) and Et_3N•3HF (96 mg, 0.6 mmol). Ratio of *branched*:*linear* isomers was determined to be 18:1 from the crude reaction mixture using ^{19}F-NMR spectroscopy. Purification by flash chromatography on silica gel (hexane/ethyl acetate = 20/1) provided the title compound as colorless oil (55 mg, 87% yield).

^{1}H NMR (400 MHz, $CDCl_3$) δ 7.57 – 7.54 (m, 2H), 7.47 – 7.36 (m, 4H), 7.31 – 7.24 (m, 2H), 6.45 (dd, *J* = 4.4, 0.9 Hz, 1H), 6.19 (d, *J* = 0.9 Hz, 1H), 4.17 – 4.09 (m, 2H), 2.04 (d, *J* = 23.5 Hz, 3H), 1.21 (t, *J* = 7.1 Hz, 3H); **^{13}C NMR** (101 MHz, $CDCl_3$) δ 164.7 (d, *J* = 6.3 Hz), 159.5 (d, *J* = 248.9), 143.6 (dd, *J* = 16.5, 7.4 Hz), 143.3 (d, *J* = 24.3 Hz), 135.5, 130.5 (d, *J* = 3.8 Hz), 129.1 (d, *J* = 2.9 Hz), 128.7 (dd, *J* = 13.6, 2.2 Hz), 128.6, 127.9, 125.3 (d, *J* = 11.9 Hz), 121.6 (dd, *J* = 6.5, 3.5 Hz), 113.8 (dd, *J* = 24.9, 7.1 Hz), 95.3 (dd, *J* = 175.2, 1.7 Hz), 61.0, 25.8 (d, *J* = 24.6 Hz), 14.1; **^{19}F NMR** (376 MHz, $CDCl_3$) δ -117.9 (dd, *J* = 11.7, 8.1 Hz), -132.4 (qd, *J* = 23.5, 4.4 Hz).

HRMS (ESI): calculated for $C_{19}H_{18}O_2F_2Na^+$ $[M+Na]^+$ m/z: 339.1167, found: 339.1162.

Ethyl 3-fluoro-3-(2-fluoro-[1,1'-biphenyl]-4-yl)-2-oxobutanoate (±)-F

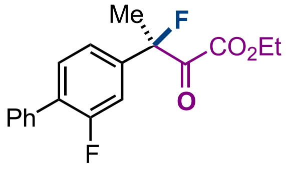

Synthesis of **(±)-F** was done following the previous protocol described for the synthesis of **E** using **(±)-11** (90 mg, 0.28 mmol), DMF (2.0 mL), OsO_4 (0.7 mg, 1.0 mol%) and Oxone (300 mg, 2.0 mmol). Purification by flash chromatography on silica gel (hexane/ethyl acetate = 20/1) provided the **(±)-F** as colorless oil (52 mg, 58% yield).

^{1}H NMR (400 MHz, $CDCl_3$) δ 7.56 – 7.53 (m, 2H), 7.50 – 7.43 (m, 3H), 7.41 – 7.37 (m, 1H), 7.33 – 7.26 (m, 2H), 4.38 – 4.30 (m, 2H), 1.97 (d, *J* = 22.8 Hz, 3H), 1.34 (t, *J* = 7.1 Hz, 3H); **^{13}C NMR** (101 MHz, $CDCl_3$) δ 193.2 (d, *J* = 37.8 Hz), 162.1, 159.8 (dd, J =250.2, 1.8 Hz), 138.7 (dd, *J* = 23.2, 7.7 Hz), 135.1 (d, *J* = 1.4 Hz), 131.2 (d, *J* = 3.7 Hz), 129.8 (d, *J* = 14.2 Hz), 129.1 (d, *J* = 2.9 Hz), 128.7, 128.2, 120.7 (dd, *J* = 8.4, 3.7 Hz), 113.0 (d, *J* = 25.9, 9.6

Hz), 98.7 (dd, J = 184.1, 1.7 Hz), 62.8, 24.6 (d, J = 23.2 Hz), 14.1; ^{19}**F NMR** (376 MHz, CDCl3) δ -116.5 (m), -157.1 (q, J = 22.8 Hz).

HRMS (ESI): calculated for $C_{18}H_{16}O_3F_2Na^+$ $[M+Na]^+$ m/z: 341.1222, found: 341.1225.

2-Fluoro-2-(2-fluoro-[1,1'-biphenyl]-4-yl)propanoic acid (±)-12

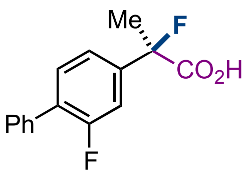

Synthesis of **(±)-12** was done following the previous protocol described for the synthesis of **10** using **(±)-F** (64 mg, 0.2 mmol), THF (2.0 mL), NaOH (0.8 mL, 2M in water, 4.0 equiv.) and H_2O_2 (102 µL, 30% w/w, 5.0 equiv.). Purification by flash chromatography on silica gel (dichloromethane/methanol = 20/1) provided the title compound as white solid (32 mg, 62 % yield); ^{1}**H NMR** (400 MHz, $CDCl_3$) δ 7.55 – 7.52 (m, 2H), 7.51 – 7.42 (m, 3H), 7.43 – 7.32 (m, 3H), 2.00 (d, J = 22.2 Hz, 3H); ^{13}**C NMR** (101 MHz, $CDCl_3$) δ 175.1 (d, J = 27.7 Hz), 159.7 (d, J = 249.6 Hz), 139.6 (dd, J = 23.2, 7.6 Hz), 135.1, 131.1 (d, J = 4.0 Hz), 129.9 (d, J = 13.4 Hz), 129.1 (d, J = 3.1 Hz), 128.7, 128.2, 120.8 (dd, J = 5.2 Hz, 3.8 Hz), 113.2 (dd, J = 25.7, 9.5 Hz), 93.9 (dd, J = 188.0, 1.7 Hz), 24.7 (d, J = 23.6 Hz); ^{19}**F NMR** (376 MHz, $CDCl_3$) δ -116.6 (m), -151.6 (q, J = 22.2 Hz).

Spectra are consistent with previously reported.[26]

8. Radiofluorination of styrenes 1a and 1i.

Procedure for preparation of a [^{18}F]TEAF solution in CH_2Cl_2:

[^{18}F]Fluoride was generated in an IBA Cyclone 18/9 cyclotron by irradiation of ^{18}O-enriched-water with high energy protons (18 MeV) via ^{18}O(p,n) ^{18}F reaction.

[^{18}F]Fluoride (0.5 – 1.0 GBq) was separated from ^{18}O-enriched-water using a Waters Plus QMA Plus Light ^{18}F separation cartridge (130 mg) and subsequently released with a solution of tetraethylammonium bicarbonate (9 mg/mL) in MeCN/H_2O, 4:1 (3 x 300 µL) into a 5 mL V-vial. The solution was dried with three cycles of azeotropic drying with MeCN (3 x 500 µL) under a flow of N_2 at 105 °C. The dried [^{18}F]TEAF residue was re-dissolved in anhydrous CH_2Cl_2 (250 – 500 µL) to obtain a concentration of radioactivity of 0.2 GBq/100 µL approximately.

Procedure for the ^{18}F-Fluorination of substrates:

To a 10 mL reaction tube was weighed the desired substrate (0.02 mmol). A freshly prepared solution of $Rh_2(esp)_2$ in dry degassed CH_2Cl_2 was added to it (200 μL, 1.0 M). The tube was sealed and evacuated and backfilled with nitrogen 3 times. The resulting mixture was cooled down to -50 ºC. Then, a solution of reagent **2a** (12.0 mg, 0.02 mmol, 1.0 equiv.) in degassed CH_2Cl_2 (1.0 mL) was added dropwise during 1 h using a syringe pump. After the addition, a solution of [^{18}F]TEAF in dry CH_2Cl_2 (0.2 GBq in 100 μL) was added and the reaction mixture was heated up to -30 ºC and stirred at this temperature for 20 min. After this time, an aliquot (300 μL) was taken for analysis by radio HPLC, CH_2Cl_2 was removed under a flow of N_2 and the residue was redissolved in dry MeCN (300 μL). Analysis was performed using an Agilent 1120 Compact LC system equipped with a variable wavelength UV detector and a radioactivity detector (Gabi, Raytest) interfaced using an analog-to-digital converter. Occasionally, an Agilent 1200 series HPLC system equipped with a variable wavelength UV detector and a radioactivity detector (Gabi, Raytest) controlle

HPLC conditions for small scale ^{18}F-Fluorination of substrates [^{18}F]3a and [^{18}F]3i:

Agilent 1120 Compact LC with a Teknokroma Mediterranea Sea C18 Column, 5 μm, 4.6 x 150 mm; flow rate: 1 mL/min; solvent A: Water with 0.1 % TFA, solvent B: MeCN with 0.1 % TFA., gradient: 0 – 1 min (A : B 80/20) isocratic, 1 – 10 min (A : B 80/20 to 20/80) linear increase, 10 – 15 min (A : B 20/80) isocratic, 15 – 18 min (A : B 20/80 to 80/20) linear decrease and 18 – 20 min (A : B = 80:20) isocratic.

Radio-HPLC of substrates [^{18}F]3a and [^{18}F]3i:

Crude Radio-HPLC traces of the crude mixture following the general procedure. The top chromatogram shows the UV trace for cold reference material (wavelength = 220 nm) and the bottom one the crude radio-HPLC trace. Radiochemical conversions (RCC) were calculated dividing the area of the peak from the desired product by the sum of areas of the rest of the peaks that appear in the radio-HPLC chromatogram.

[^{18}F] Ethyl 3-fluoro-2-methylene-3-phenylbutanoate ([^{18}F]3a)

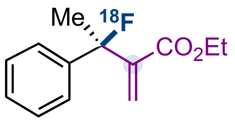

[^{18}F]3a
10 ±2 % RCY $_{(n\,=\,3)}$

Prepared according to the described procedure using prop-1-en-2-ylbenzene **1a** (2.4 mg 0.02 mmol) and reagent **2** (12.0 mg, 0.02 mmol, 1.0 equiv.). The radiochemical conversions (RCC) and the Radio-HPLC chromatograms are shown below:

Reaction	RCC
1	12
2	8
3	10
RCC mean ± Standard deviation	10 ± 2

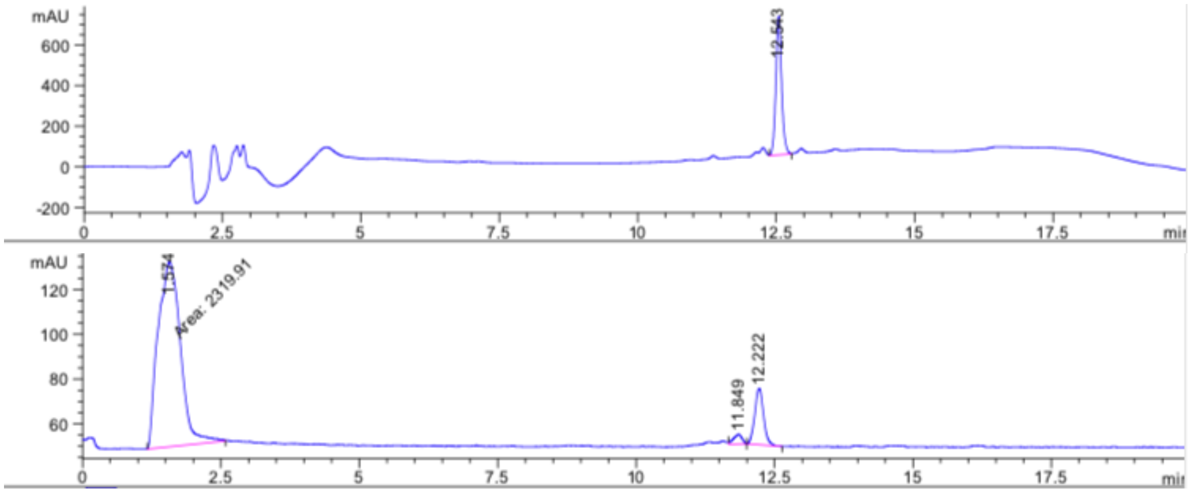

Figure 1: The top chromatogram shows the UV trace for cold reference material (wavelength = 220 nm) and the bottom one the crude radio-HPLC trace for ***[^{18}F]3a****.*

[^{18}F] Ethyl 3-(4-acetoxyphenyl)-3-fluoro-2-methylenebutanoate ([^{18}F]3i)

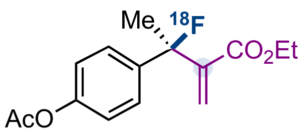

[^{18}F]3i
9 ±2 % RCY $_{(n\,=\,3)}$

Prepared according to the general procedure using 4-(prop-1-en-2-yl)phenyl acetate **1i** (3.5 mg, 0.02 mmol) and reagent **2** (12.0 mg, 0.02 mmol, 1.0 equiv.). The radiochemical conversions (RCC) and the Radio-HPLC chromatograms are shown below:

Reaction	RCC
1	10
2	7
3	9
RCC mean ± Standard deviation	9 ± 2

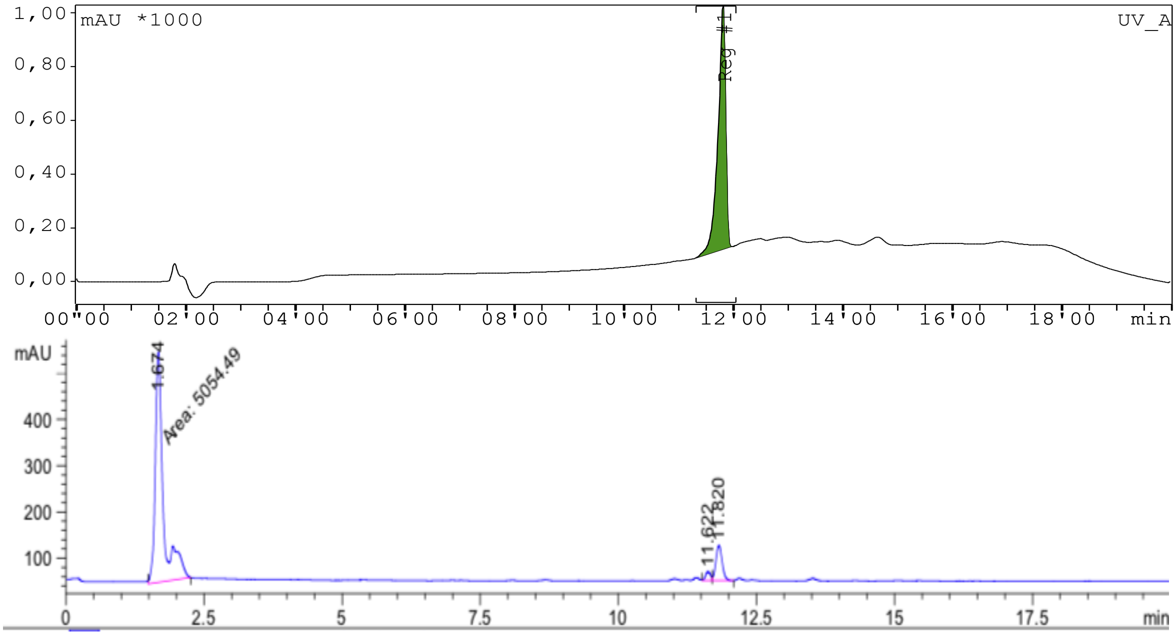

Figure 2: The top chromatogram shows the UV trace for cold reference material (wavelength = 220 nm) and the bottom one the crude radio-HPLC trace for ***[¹⁸F]3i****.*

9. Fluorination of styrene and β-methylstyrene.

Ethyl 2-(fluoro(phenyl)methyl)acrylate (13) and Ethyl (*Z*)-2-(fluoromethyl)-3-phenylacrylate (13')

Prepared according to general procedure C using styrene (42 mg, 0.4 mmol, 2.0 equiv.), reagent **2a** (120 mg, 0.2 mmol) and Et_3N•3HF (96 mg, 0.6 mmol). Ratio of *branched*:*linear* isomers was determined to be 3:1 and the *Z*:*E* ratio from the *linear* isomer was determined to be 6:1 from the crude reaction mixture using ^{19}F-NMR spectroscopy. Purification by flash chromatography on silica gel (hexane/ethyl acetate = 30/1) provided a mixture of the title compounds as colorless oil (41 mg, 65% yield). Further separation of two isomers was achieved with a PLC plate (hexane/ethyl acetate = 50/1).

Ethyl 2-(fluoro(phenyl)methyl)acrylate (13)

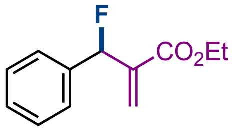

^{1}H NMR (400 MHz, $CDCl_3$) δ 7.45 – 7.28 (m, 5H), 6.45 (dt, *J* = 2.7, 1.0 Hz, 1H), 6.28 (dt, *J* = 46.0, 1.3 Hz, 1H), 6.01 (t, *J* = 1.3 Hz, 1H), 4.27 – 4.08 (m, 2H), 1.22 (t, *J* = 7.1 Hz, 3H); **^{13}C NMR** (101 MHz, $CDCl_3$) δ 164.9 (d, *J* = 6.3 Hz), 139.8 (d, *J* = 22.8 Hz), 137.6 (d, *J* = 20.4 Hz), 129.1 (d, *J* = 2.6 Hz), 128.6, 127.3 (d, *J* = 5.6 Hz), 125.8 (d, *J* = 8.9 Hz), 91.0 (d, *J* = 174.5 Hz), 61.1, 14.1; **^{19}F NMR** (376 MHz, $CDCl_3$) δ -171.0 (dd, *J* = 46.0, 2.7 Hz).

Spectra are consistent with previously reported.[27]

Ethyl (*Z*)-2-(fluoromethyl)-3-phenylacrylate (13')

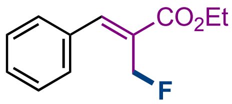

^{1}H NMR (400 MHz, $CDCl_3$) δ 8.07 (d, *J* = 2.0 Hz, 1H), 7.57 – 7.47 (m, 2H), 7.47 – 7.40 (m, 3H), 5.23 (d, *J* = 47.6 Hz, 2H), 4.34 (q, *J* = 7.1 Hz, 2H), 1.38 (t, *J* = 7.1 Hz, 3H); **^{13}C NMR** (101 MHz, $CDCl_3$) δ 166.9 (d, *J* = 1.3 Hz), 147.3 (d, *J* = 7.1 Hz), 139.2 (d, *J* = 10.7 Hz), 130.1, 129.9 (d, *J* = 3.8 Hz), 129.8, 128.2, 77.2 (d, *J* = 162.9 Hz), 61.5, 14.4; **^{19}F NMR** (376 MHz, $CDCl_3$) δ -205.1 (td, *J* = 47.6, 4.0 Hz); ^{1}H-^{13}C HSQC, ^{1}H-^{13}C HMBC and ^{1}H-^{1}H NOE spectra were measured.

HRMS (ESI): calculated for $C_{12}H_{13}O_2FNa^+$ $[M+Na]^+$ m/z: 231.0792, found: 231.0795.

Ethyl (*Z*)-2-benzylidene-3-fluorobutanoate (14-branched) and Ethyl (*E*)-2-(fluoro(phenyl)methyl)but-2-enoate (14-branched')

Prepared according to general procedure C using β-methyl-styrene (47 mg, 0.4 mmol, 2.0 equiv.), reagent **2a** (120 mg, 0.2 mmol) and Et_3N•3HF (96 mg, 0.6 mmol). Ratio of *branched:branched′* isomers was determined to be 1.4:1; the *E*:*Z* ratio from the *branched* isomer was determined to be 8:1 and the *Z*:*E* ratio from the *branched* isomer was determined to be 10:1 from the crude reaction mixture using ^{19}F-NMR spectroscopy. Purification by flash chromatography on silica gel (hexane/ethyl acetate = 40/1) provided a mixture of the title compounds as colorless oil (41 mg, 77% yield).

-14-branched

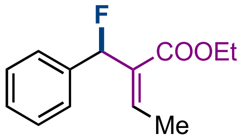

^{1}H NMR (400 MHz, $CDCl_3$) δ 7.39 – 7.29 (m, 5H), 6.37 (m, 1H), 6.35 – 6.20 (m, 1H), 4.17 (qd, *J* = 7.1, 0.2 Hz, 2H), 2.10 (dq, *J* = 1.8, 0.3 Hz, 3H), 1.21 (t, *J* = 7.1 Hz, 3H); **^{13}C NMR** (101 MHz, $CDCl_3$) δ 165.7 (d, *J* = 3.6 Hz), 139.8 (d, *J* = 9.6 Hz), 138.2 (d, *J* = 21.2 Hz), 132.1 (d, *J* = 20.4), 128.6 (d, *J* = 2.3

Hz), 128.4, 126.8 (d, J = 6.1 Hz), 92.1 (d, J = 174.1 Hz), 60.5, 15.6, 14.1; **^{19}F NMR** (376 MHz, $CDCl_3$) δ -169.0 (m); ^{1}H-^{13}C HSQC, ^{1}H-^{13}C HMBC and ^{1}H-^{1}H NOE spectra were measured.

HRMS (ESI): calculated for $C_{13}H_{15}O_2FNa^+$ $[M+Na]^+$ m/z: 245.0948, found: 245.0939.

-14-branched′

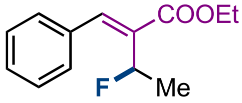

^{1}H NMR (400 MHz, $CDCl_3$) δ 7.83 (s, 1H), 7.44 – 7.30 (m, 5H), 5.67 (dq, J = 46.3, 6.6 Hz, 1H), 4.39 – 4.25 (m, 2H), 1.72 (dd, J = 22.6, 6.6 Hz, 3H), 1.38 (t, J = 7.1 Hz, 3H); **^{13}C NMR** (101 MHz, $CDCl_3$), δ 166.3 (d, J = 1.8 Hz), 142.9 (d, J = 6.2 Hz), 134.4 (d, J = 2.0 Hz), 131.9 (d, J = 18.8 Hz), 129.5 (d, J = 2.8 Hz), 129.2, 128.7, 85.6 (d, J = 167.3 Hz), 61.0, 20.2 (d, J = 25.4 Hz), 14.4; **^{19}F NMR** (376 MHz, $CDCl_3$) δ -168.5 (m); ^{1}H-^{13}C HSQC, ^{1}H-^{13}C HMBC and ^{1}H-^{1}H NOE spectra were measured.

HRMS (ESI): calculated for $C_{13}H_{15}O_2FNa^+$ $[M+Na]^+$ m/z: 245.0948, found: 245.0938.

10. References.

(1) Huang, J.; Hu, G.; An, S.; Chen, D.; Li, M.; Li, P. Synthesis of N-Alkylpyridin-4-Ones and Thiazolo[3,2- a]Pyridin-5-Ones through Pummerer-Type Reactions. *J. Org. Chem.* **2019**, *84* (15), 9758–9769. https://doi.org/10.1021/acs.joc.9b01672.

(2) Zhang, L.; Zuo, Z.; Wan, X.; Huang, Z. Cobalt-Catalyzed Enantioselective Hydroboration of 1,1-Disubstituted Aryl Alkenes. *J. Am. Chem. Soc.* **2014**, *136* (44), 15501–15504. https://doi.org/10.1021/ja5093908.

(3) Wen, Z. K.; Ge, X. M.; Zhao, Z. K.; Chao, J. Bin. Palladium/Acid Relay Catalyzed Tandem Heck Coupling/6-Endo Cyclization between Ortho-Halogenated Benzoates and Unactivated Terminal Alkenes for the Synthesis of 1-Isochromanones. *Adv. Synth. Catal.* **2019**, *361* (5), 983–988. https://doi.org/10.1002/adsc.201801507.

(4) Xing, Y.; Yu, R.; Fang, X. Synthesis of Tertiary Benzylic Nitriles via Nickel-Catalyzed Markovnikov Hydrocyanation of α-Substituted Styrenes. *Org. Lett.* **2020**, *22* (3), 1008–1012. https://doi.org/10.1021/acs.orglett.9b04554.

(5) Liu, W.; Lau, F.; Liu, K.; Wood, H. B.; Zhou, G.; Chen, Y.; Li, Y.; Akiyama, T. E.; Castriota, G.; Einstein, M.; Wang, C.; McCann, M. E.; Doebber, T. W.; Wu, M.; Chang, C. H.; McNamara, L.; McKeever, B.; Mosley, R. T.; Berger, J. P.; Meinke, P. T. Benzimidazolones: A New Class of Selective Peroxisome Proliferator- Activated

Receptor γ (PPARγ) Modulators. *J. Med. Chem.* **2011**, *54* (24), 8541–8554. https://doi.org/10.1021/jm201061j.

(6) Zhang, S.; Bedi, D.; Cheng, L.; Unruh, D. K.; Li, G.; Findlater, M. Cobalt(II)-Catalyzed Stereoselective Olefin Isomerization: Facile Access to Acyclic Trisubstituted Alkenes. *J. Am. Chem. Soc.* **2020**, *142* (19), 8910–8917. https://doi.org/10.1021/jacs.0c02101.

(7) Chatalova-Sazepin, C.; Wang, Q.; Sammis, G. M.; Zhu, J. Copper-Catalyzed Intermolecular Carboetherification of Unactivated Alkenes by Alkyl Nitriles and Alcohols. *Angew. Chemie* **2015**, *127* (18), 5533–5536. https://doi.org/10.1002/ange.201412357.

(8) Huang, C. Y.; Doyle, A. G. Electron-Deficient Olefin Ligands Enable Generation of Quaternary Carbons by Ni-Catalyzed Cross-Coupling. *J. Am. Chem. Soc.* **2015**, *137* (17), 5638–5641. https://doi.org/10.1021/jacs.5b02503.

(9) Baudoux, J.; Cahard, D. Electrophilic Fluorination with N-F Reagents. *Org. React.* **2008**, *69*, 1–326. https://doi.org/10.1002/0471264180.or069.02.

(10) Zhang, G.; McCorvy, J. D.; Shen, S.; Cheng, J.; Roth, B. L.; Kozikowski, A. P. Design of Fluorinated Cyclopropane Derivatives of 2-Phenylcyclopropylmethylamine Leading to Identification of a Selective Serotonin 2C (5-HT2C) Receptor Agonist without 5-HT2B Agonism. *Eur. J. Med. Chem.* **2019**, *182*. https://doi.org/10.1016/j.ejmech.2019.111626.

(11) Siu, J. C.; Parry, J. B.; Lin, S. Aminoxyl-Catalyzed Electrochemical Diazidation of Alkenes Mediated by a Metastable Charge-Transfer Complex. *J. Am. Chem. Soc.* **2019**, *141* (7), 2825–2831. https://doi.org/10.1021/jacs.8b13192.

(12) Ruiz, J.; Cutillas, N.; López, F.; López, G.; Bautista, D. A Copper- And Amine-Free Sonogashira Reaction of Aryl Halides Catalyzed by 1,3,5-Triaza-7-Phosphaadamantane Palladium Systems. *Organometallics* **2006**, *25* (24), 5768–5773. https://doi.org/10.1021/om060636k.

(13) La, D. S.; Salituro, F. G.; Martinez Botella, G.; Griffin, A. M.; Bai, Z.; Ackley, M. A.; Dai, J.; Doherty, J. J.; Harrison, B. L.; Hoffmann, E. C.; Kazdoba, T. M.; Lewis, M. C.; Quirk, M. C.; Robichaud, A. J. Neuroactive Steroid N-Methyl- d -Aspartate Receptor Positive Allosteric Modulators: Synthesis, SAR, and Pharmacological Activity. *J. Med. Chem.* **2019**, *62* (16), 7526–7542. https://doi.org/10.1021/acs.jmedchem.9b00591.

(14) Maity, S.; Manna, S.; Rana, S.; Naveen, T.; Mallick, A.; Maiti, D. Efficient and Stereoselective Nitration of Mono- and Disubstituted Olefins with AgNO2 and TEMPO. *J. Am. Chem. Soc.* **2013**, *135* (9), 3355–3358. https://doi.org/10.1021/ja311942e.

(15) Cook, C. E.; Corley, R. C.; Wall, M. E. Synthesis and Reactions of Oxiranes Obtained. *Steroids.* **1968**, *83* (7), 5–9. https://doi.org/10.1021/jo01271a038

(16) Byrd, J. C.; Bennett, C. E.; Vibhute, S. M.; Hertlein, E.; Elgamal, O. A.; Wilson, T. A. Methods and Compositions for Inhibition of Dihydroorotate Dehydrogenase. U.S. Patent WO 2021134042, July 01, 2021.

(17) Wang, Z.; Jiang, L.; Sarró, P.; Suero, M. G. Catalytic Cleavage of C(Sp2)-C(Sp2) Bonds with Rh-Carbynoids. *J. Am. Chem. Soc.* **2019**, *141* (39), 15509–15514. https://doi.org/10.1021/jacs.9b08632.

(18) Weiss, R.; Seubert, J.; Hampel, F. α-Aryliodonio Diazo Compounds: S_N Reactions at the α-C Atom as a Novel Reaction Type for Diazo Compounds. *Angew. Chem. Int. Ed.* **1994**, *33* (19), 1952–1953. https://doi.org/10.1002/anie.199419521.

(19) Dong, J. Y.; Wang, H.; Mao, S.; Wang, X.; Zhou, M. D.; Li, L. Visible Light-Induced [3+2] Cyclization Reactions of Hydrazones with Hypervalent Iodine Diazo Reagents for the Synthesis of 1-Amino-1,2,3-Triazoles. *Adv. Synth. Catal.* **2021**, *363* (8), 2133–2139. https://doi.org/10.1002/adsc.202001436.

(20) Ramachandran, P. V.; Rudd, M. T.; Burghardt, T. E.; Ram Reddy, M. V. Vinylalumination for the Synthesis of Functionalized Allyl Alcohols, Vinylepoxides, and α-Alkylidene-β-Hydroxy-γ-Lactones. *J. Org. Chem.* **2003**, *68* (24), 9310–9316. https://doi.org/10.1021/jo034954u.

(21) Tu, H. F.; Yang, P.; Lin, Z. H.; Zheng, C.; You, S. L. Time-Dependent Enantiodivergent Synthesis via Sequential Kinetic Resolution. *Nat. Chem.* **2020**, *12* (9), 838–844. https://doi.org/10.1038/s41557-020-0489-1.

(22) Travis, B. R.; Narayan, R. S.; Borhan, B. Osmium Tetroxide-Promoted Catalytic Oxidative Cleavage of Olefins: An Organometallic Ozonolysis. *J. Am. Chem. Soc.* **2002**, *124* (15), 3824–3825. https://doi.org/10.1021/ja017295g.

(23) Orrling, K. M.; Marzahn, M. R.; Gutiérrez-de-Terán, H.; Åqvist, J.; Dunn, B. M.; Larhed, M. α-Substituted Norstatines as the Transition-State Mimic in Inhibitors of Multiple Digestive Vacuole Malaria Aspartic Proteases. *Bioorganic Med. Chem.* **2009**, *17* (16), 5933–5949. https://doi.org/10.1016/j.bmc.2009.06.065

(24) Nishimine, T.; Taira, H.; Tokunaga, E.; Shiro, M.; Shibata, N. Enantioselective Trichloromethylation of MBH-Fluorides with Chloroform Based on Silicon-Assisted C-F Activation and Carbanion Exchange Induced by a Ruppert-Prakash Reagent. *Angew. Chemie - Int. Ed.* **2016**, *55* (1), 359–363. https://doi.org/10.1002/anie.201508574.

(25) Tengeiji, A.; Shiina, I. A New Method for Production of Chiral 2-Aryl-2-Fluoropropanoic Acids Using an Effective Kinetic Resolution of Racemic 2-Aryl-2-Fluoropropanoic Acids. *Molecules* **2012**, *17* (6), 7356–7378. https://doi.org/10.3390/molecules17067356.

(26) Schiefer, I. T.; Abdul-Hay, S.; Wang, H.; Vanni, M.; Qin, Z.; Thatcher, G. R. J. Inhibition of Amyloidogenesis by Nonsteroidal Anti-Inflammatory Drugs and Their Hybrid Nitrates. *J. Med. Chem.* **2011**, *54* (7), 2293–2306. https://doi.org/10.1021/jm101450p.

(27) Zi, Y.; Lange, M.; Vilotijevic, I. Enantioselective Lewis Base Catalyzed Phosphonyldifluoromethylation of Allylic Fluorides Using a: C-Silyl Latent Pronucleophile. *Chem. Commun.* **2020**, *56* (42), 5689–5692. https://doi.org/10.1039/d0cc01815e.

11. NMR Spectra.

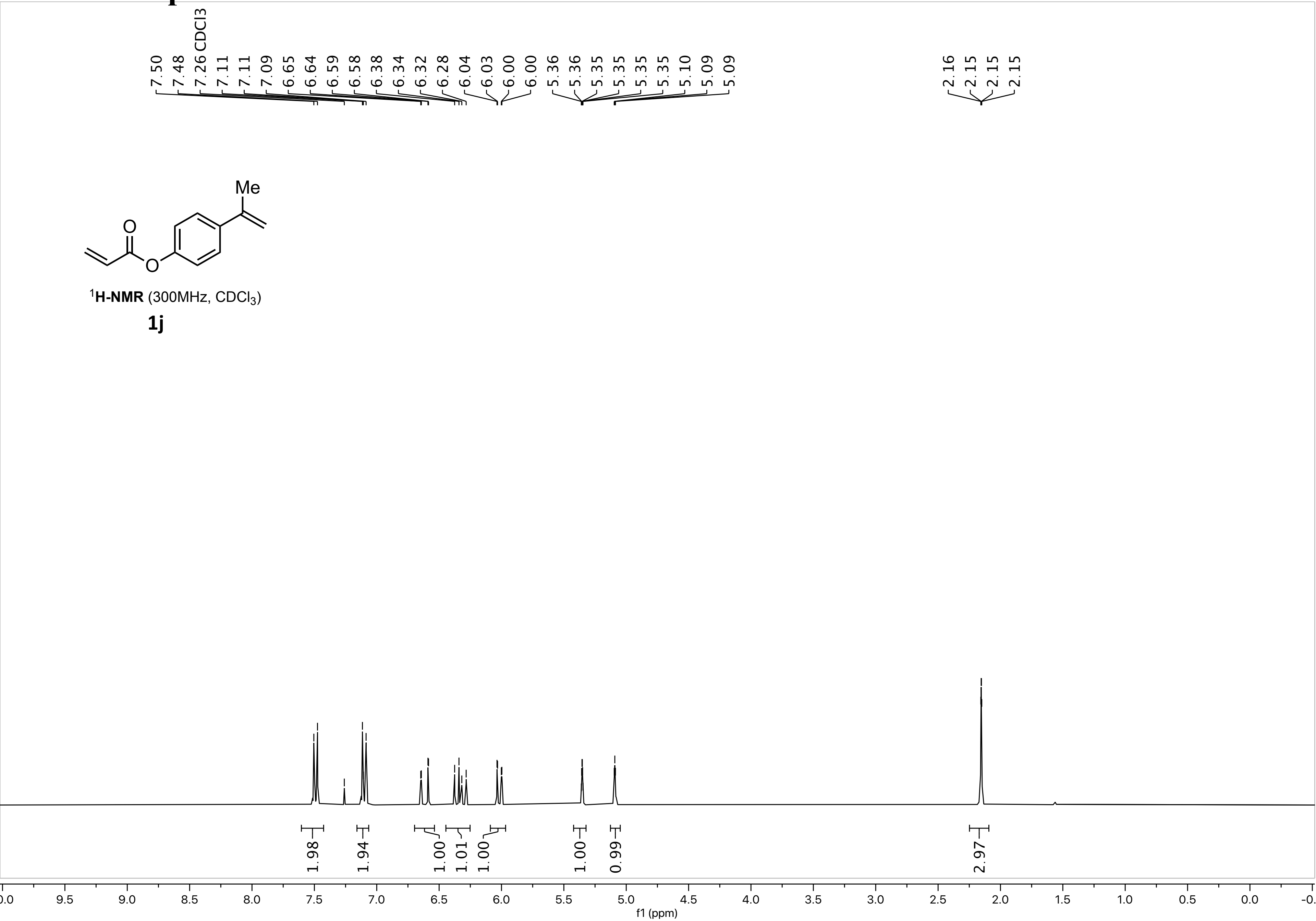

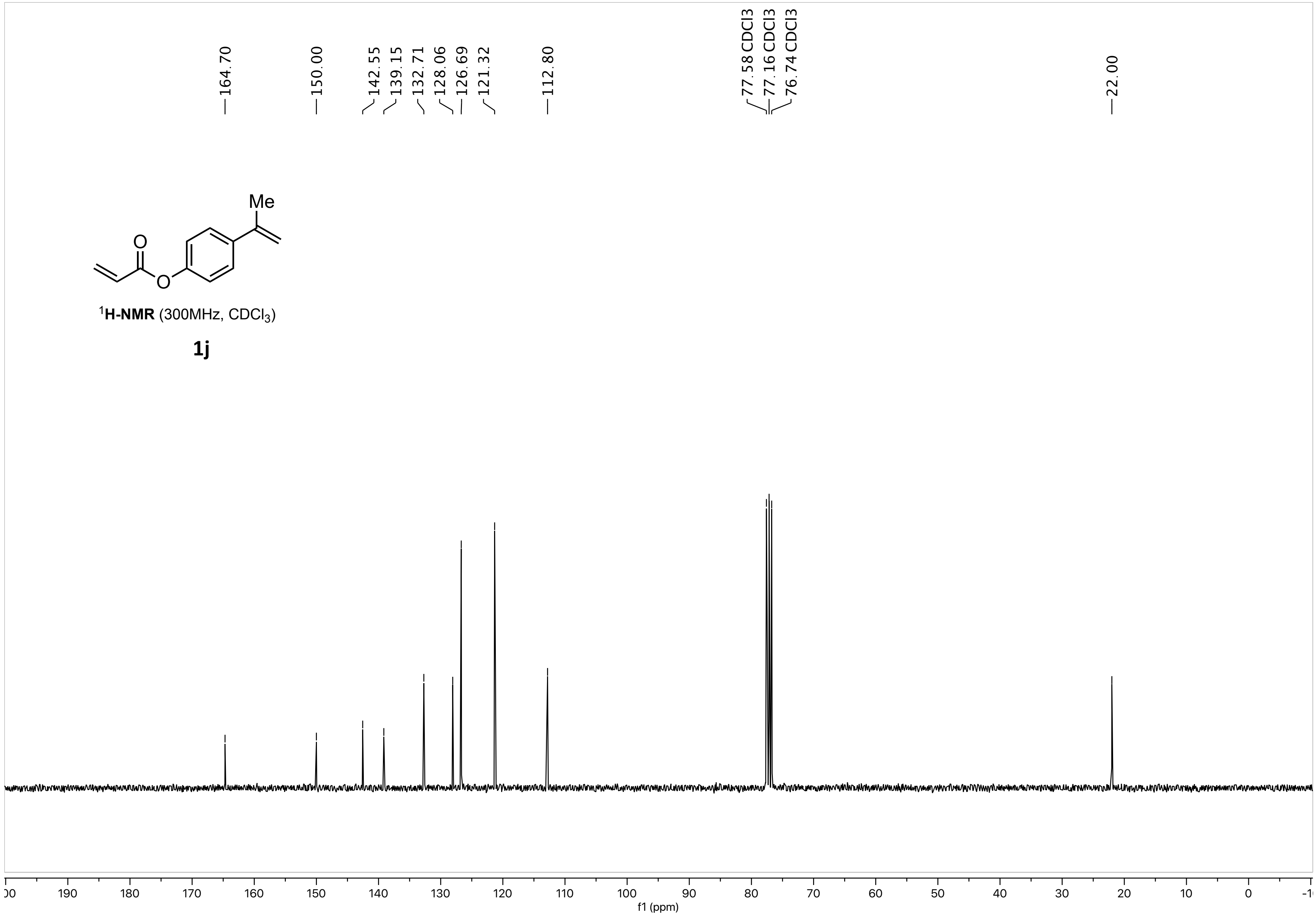

164.70
150.00
142.55
139.15
132.71
128.06
126.69
121.32
112.80
77.58 CDCl3
77.16 CDCl3
76.74 CDCl3
22.00
Me
O
O
1H-NMR (300MHz, CDCl3)
1j
f1 (ppm)

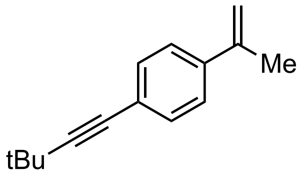

[1]**H-NMR** (300MHz, $CDCl_3$)

1k

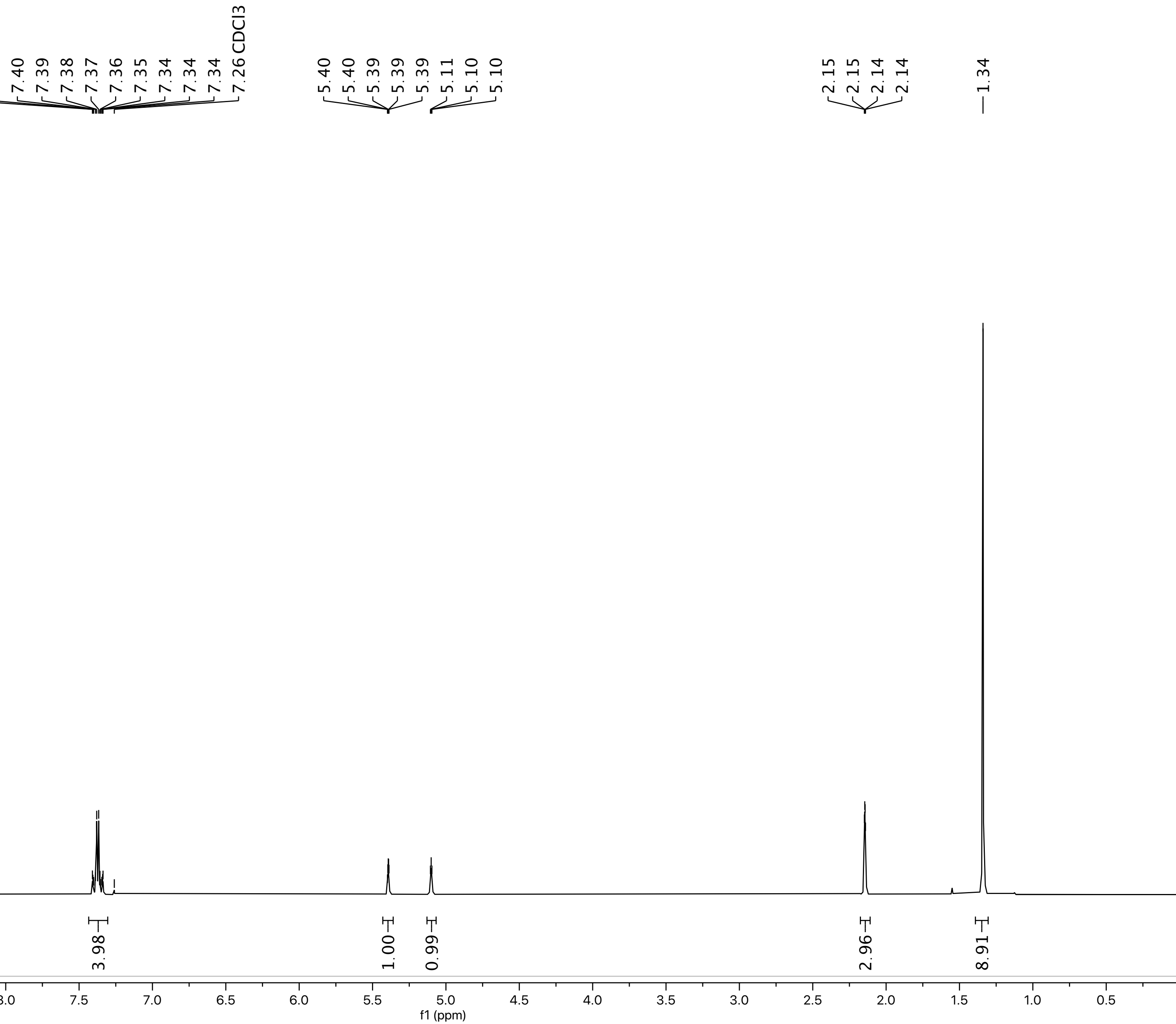

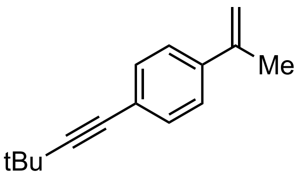

^{13}**C-NMR** (75MHz, $CDCl_3$)
1k

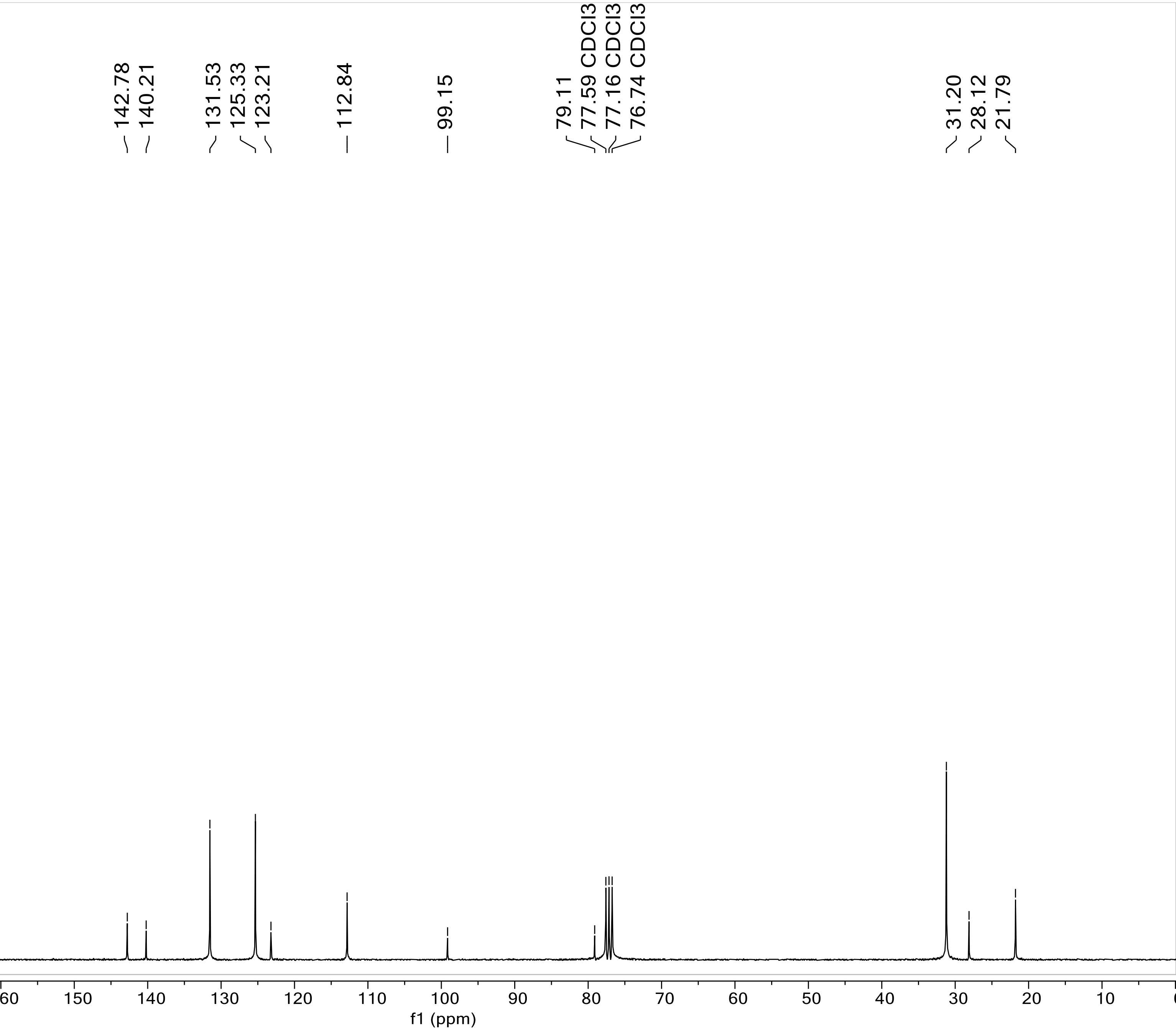

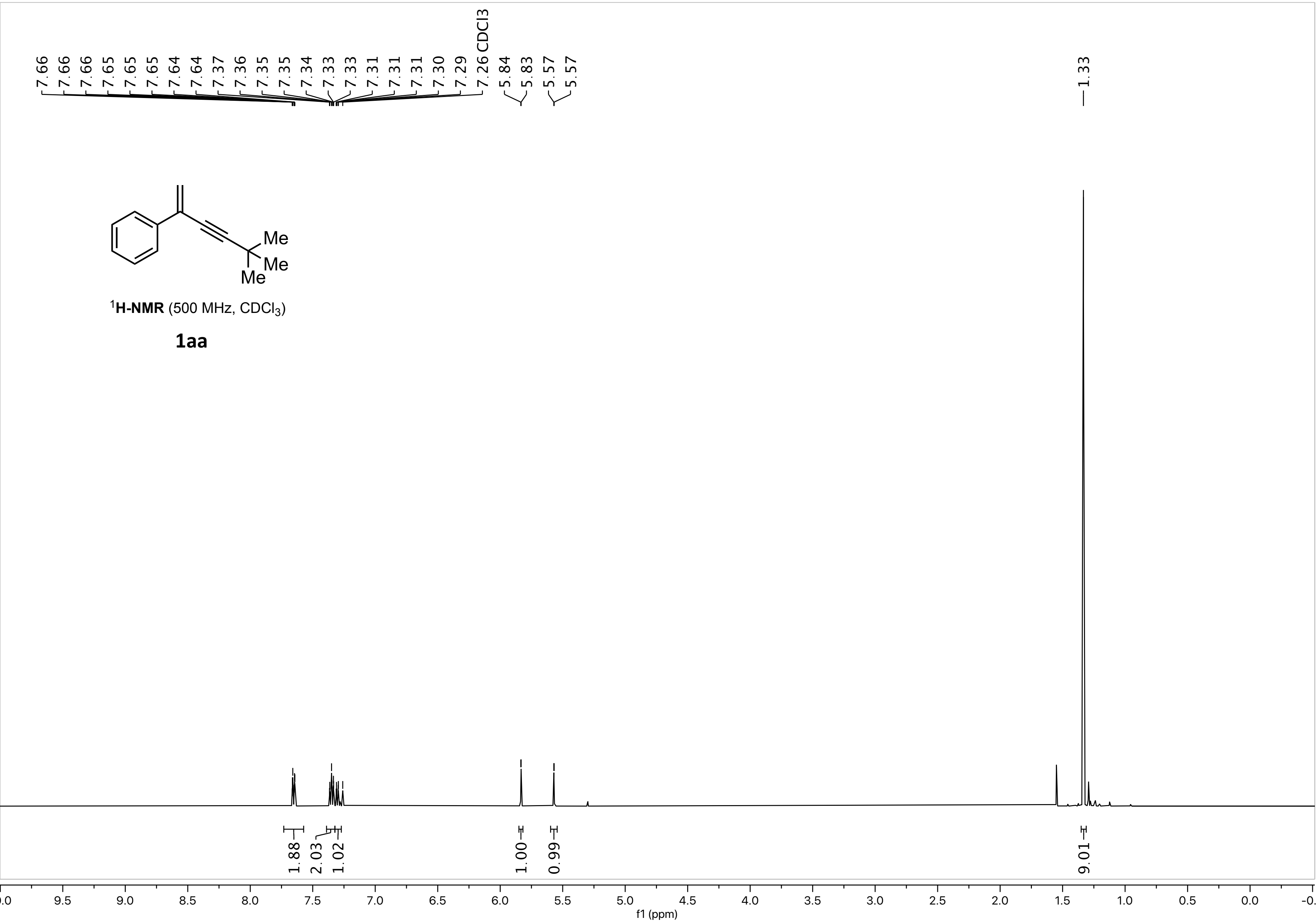

^{1}**H-NMR** (500 MHz, $CDCl_3$)

1aa

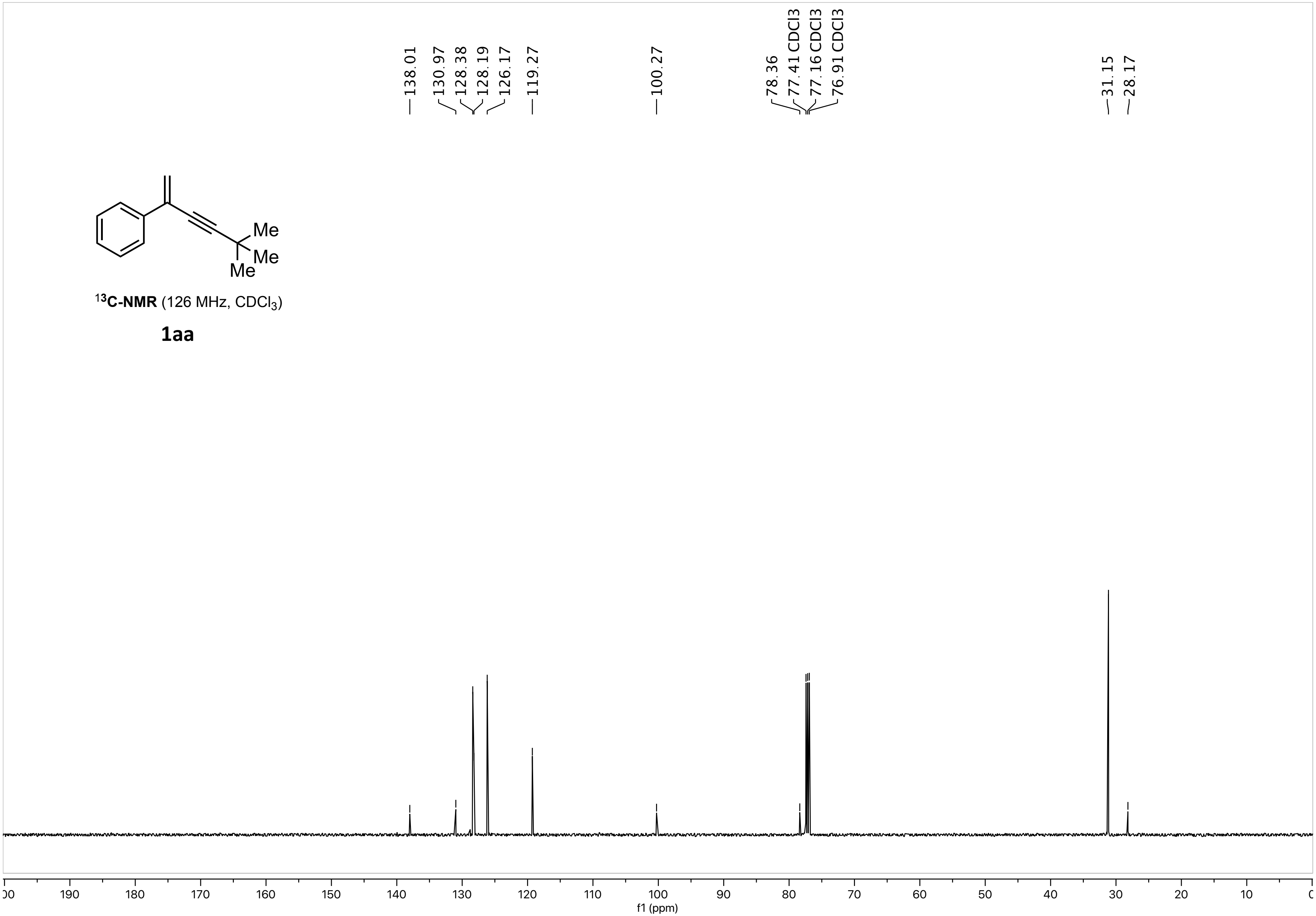
138.01
130.97
128.38
128.19
126.17
119.27
100.27
78.36
77.41 CDCl3
77.16 CDCl3
76.91 CDCl3
31.15
28.17
Me
Me
Me
13C-NMR (126 MHz, CDCl3)
1aa
190
180
170
160
150
140
130
120
110
100
90
80
70
60
50
40
30
20
10
f1 (ppm)

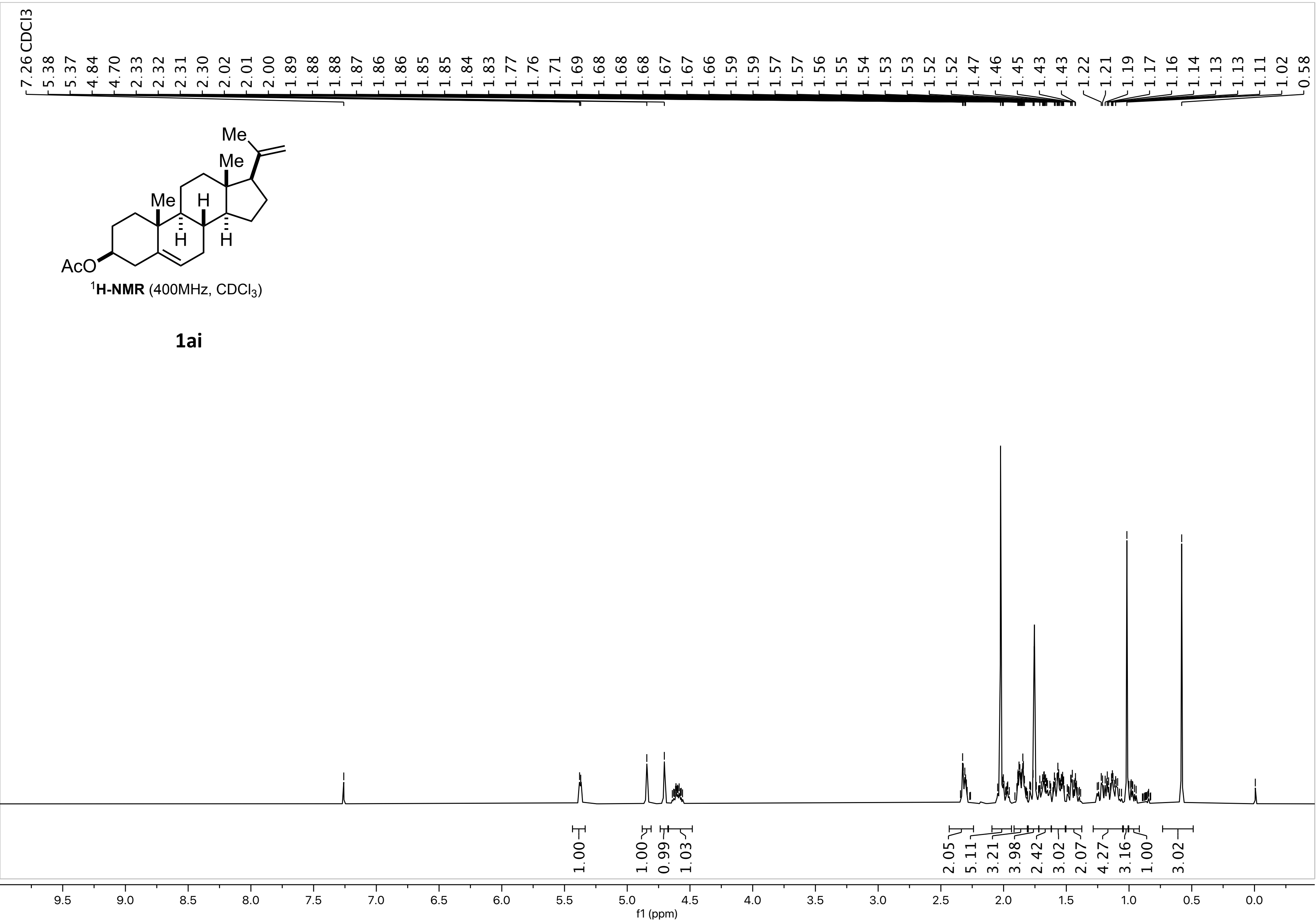
7.26 CDCl3
5.38
5.37
4.84
4.70
2.33
2.32
2.31
2.30
2.02
2.01
2.00
1.89
1.88
1.88
1.87
1.86
1.86
1.85
1.85
1.84
1.83
1.77
1.76
1.71
1.69
1.68
1.68
1.68
1.67
1.67
1.66
1.59
1.59
1.57
1.57
1.56
1.55
1.54
1.53
1.53
1.52
1.52
1.47
1.46
1.45
1.43
1.43
1.22
1.21
1.19
1.17
1.16
1.14
1.13
1.13
1.11
1.02
0.58
Me
Me
Me
H
H
H
AcO
1H-NMR (400MHz, CDCl3)
1ai
1.00
1.00
0.99
1.03
2.05
5.11
3.21
3.98
2.42
3.02
2.07
4.27
3.16
1.00
3.02
9.5
9.0
8.5
8.0
7.5
7.0
6.5
6.0
5.5
5.0
4.5
4.0
3.5
3.0
2.5
2.0
1.5
1.0
0.5
0.0
f1 (ppm)

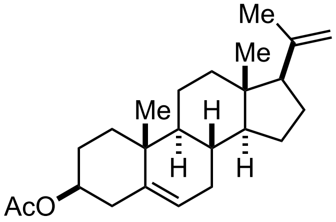

^{13}**C-NMR** (101MHz, $CDCl_3$)

1ai

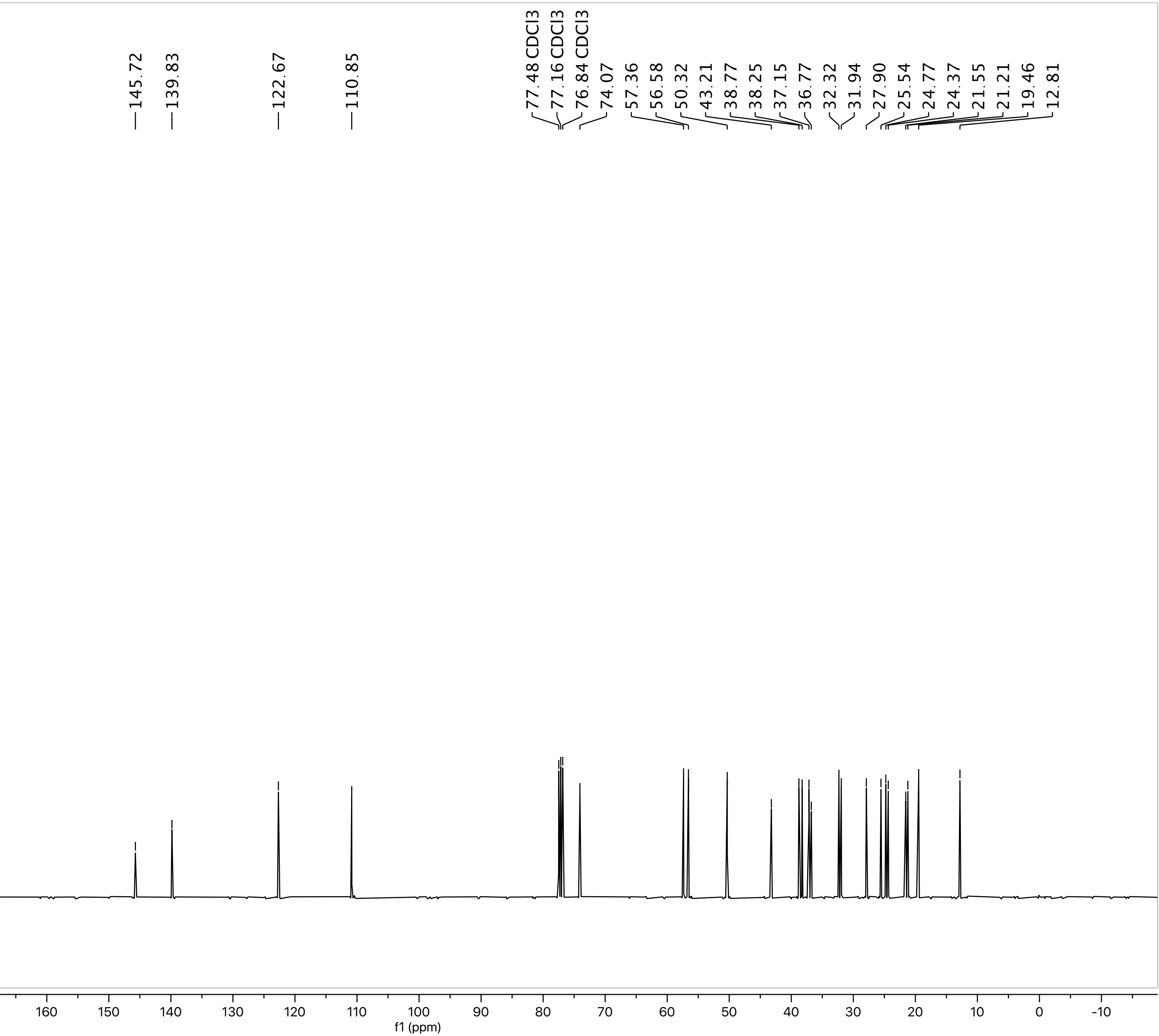

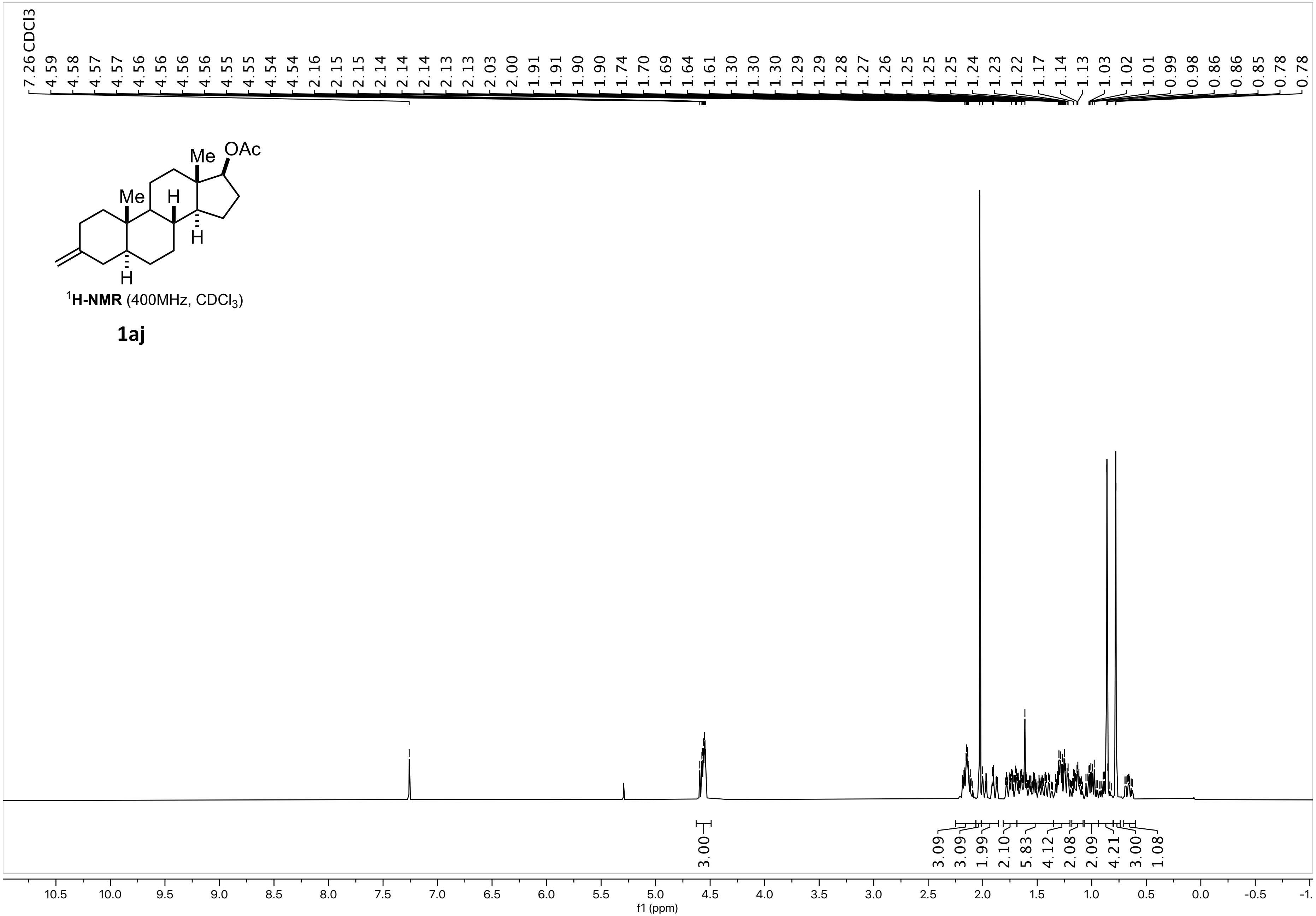

^{1}**H-NMR** (400MHz, $CDCl_3$)

1aj

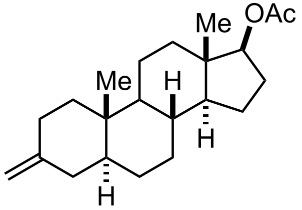

^{13}**C-NMR** (101MHz, $CDCl_3$)

1aj

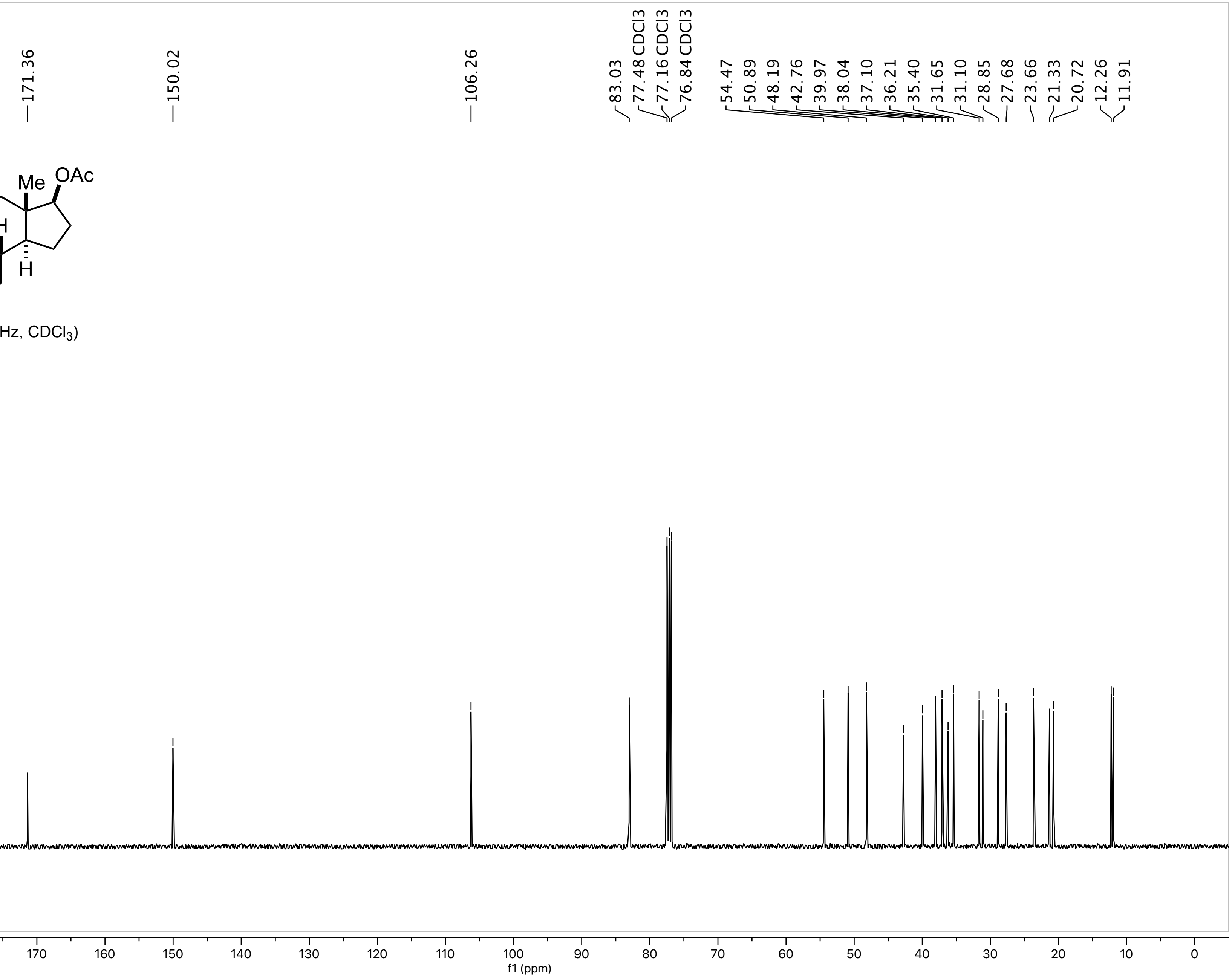

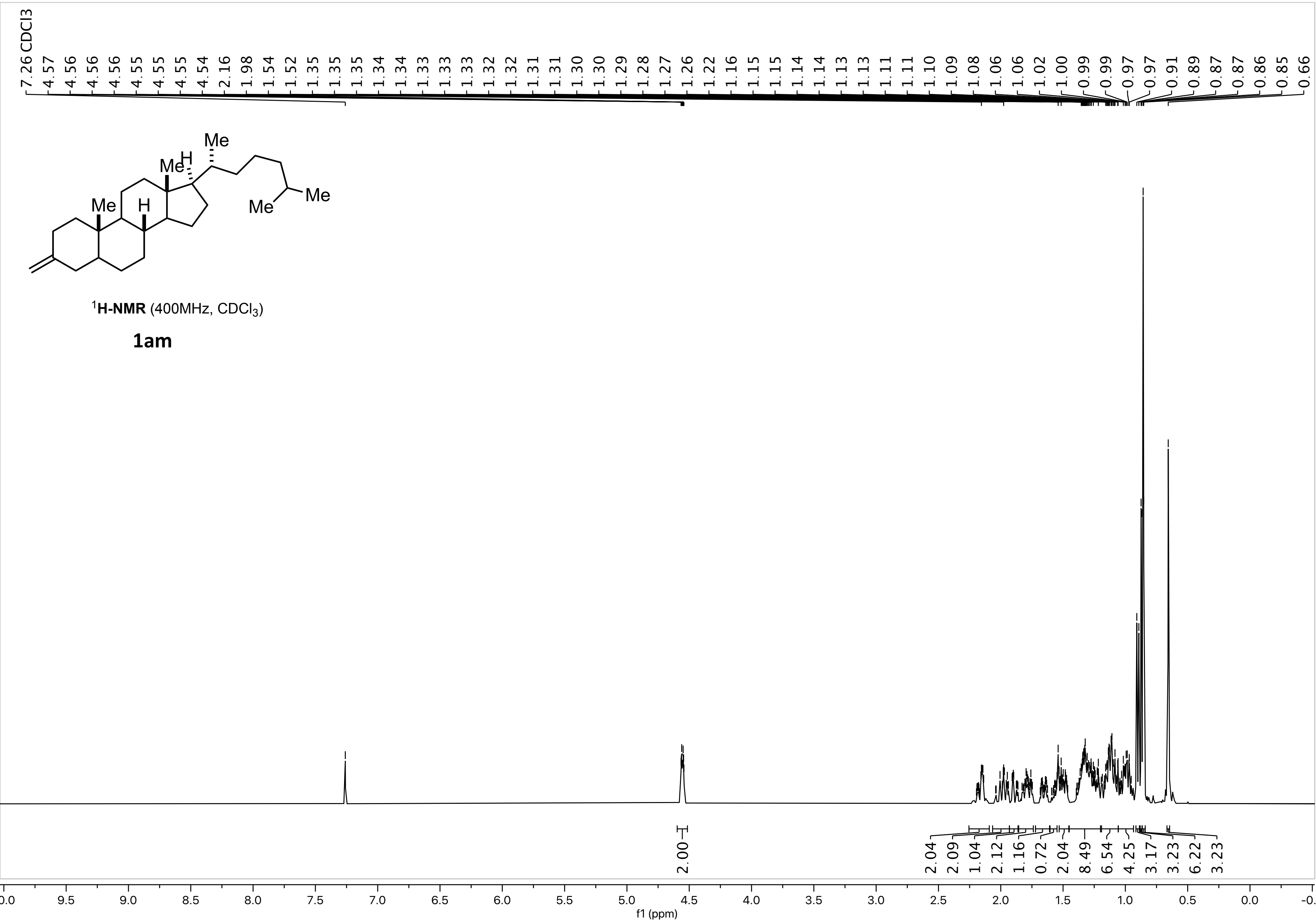
7.26 CDCl3
4.57
4.56
4.56
4.56
4.55
4.55
4.55
4.54
2.16
1.98
1.54
1.52
1.35
1.35
1.35
1.34
1.34
1.33
1.33
1.33
1.32
1.32
1.31
1.31
1.30
1.30
1.29
1.28
1.27
1.26
1.22
1.16
1.15
1.15
1.14
1.14
1.13
1.13
1.11
1.11
1.10
1.09
1.08
1.06
1.02
1.00
0.99
0.99
0.97
0.97
0.91
0.89
0.87
0.87
0.86
0.85
0.66
Me
Me
H
Me
H
Me
Me
^{1}H-NMR (400MHz, CDCl$_3$)
1am
2.00
2.04
2.09
1.04
2.12
1.16
0.72
2.04
8.49
6.54
4.25
3.17
3.23
6.22
3.23
f1 (ppm)

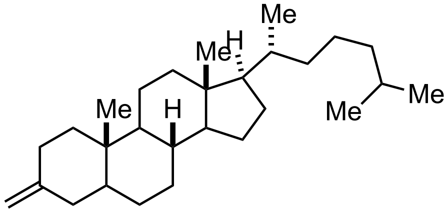

^{13}**C-NMR** (101MHz, $CDCl_3$)

1am

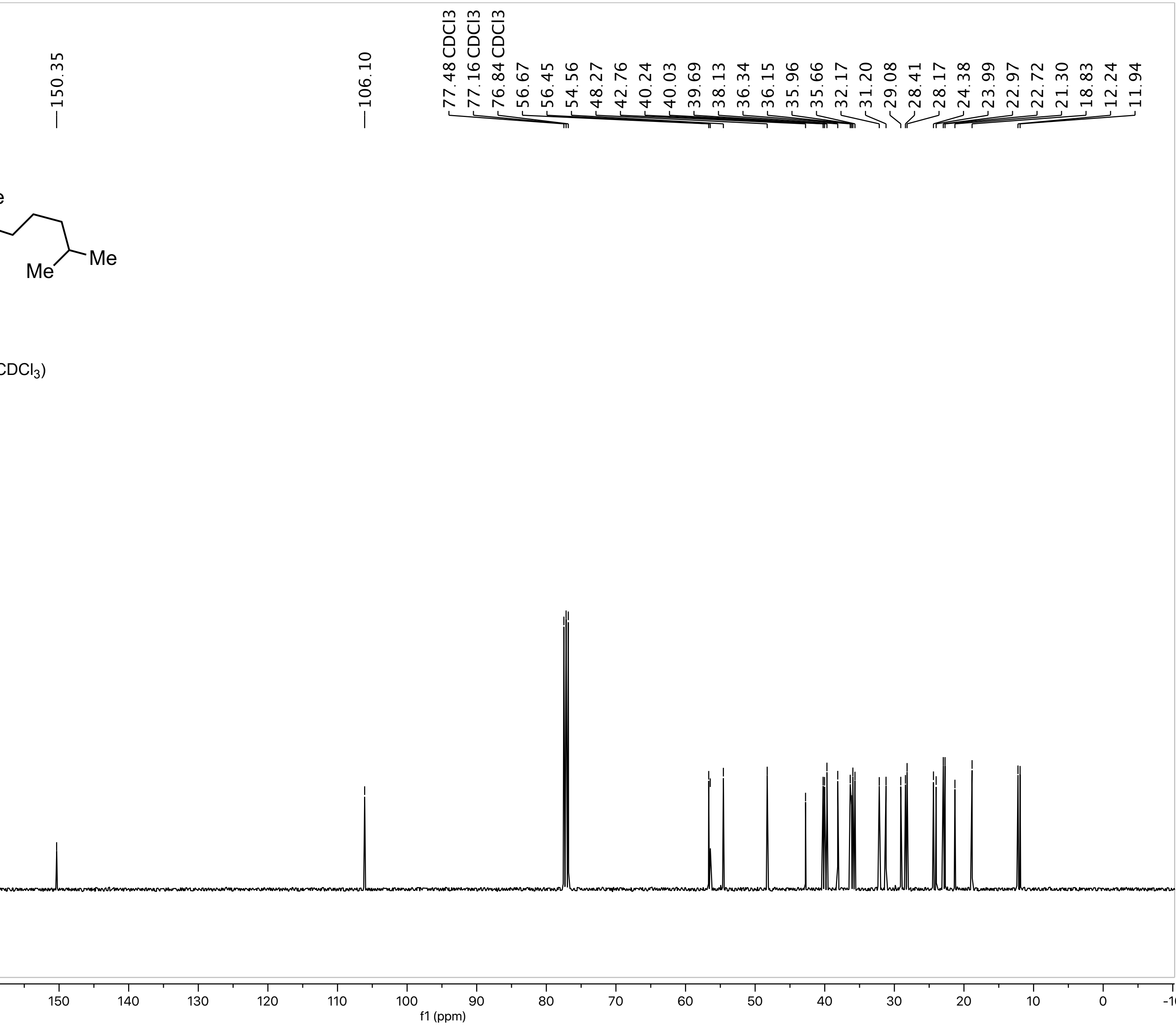

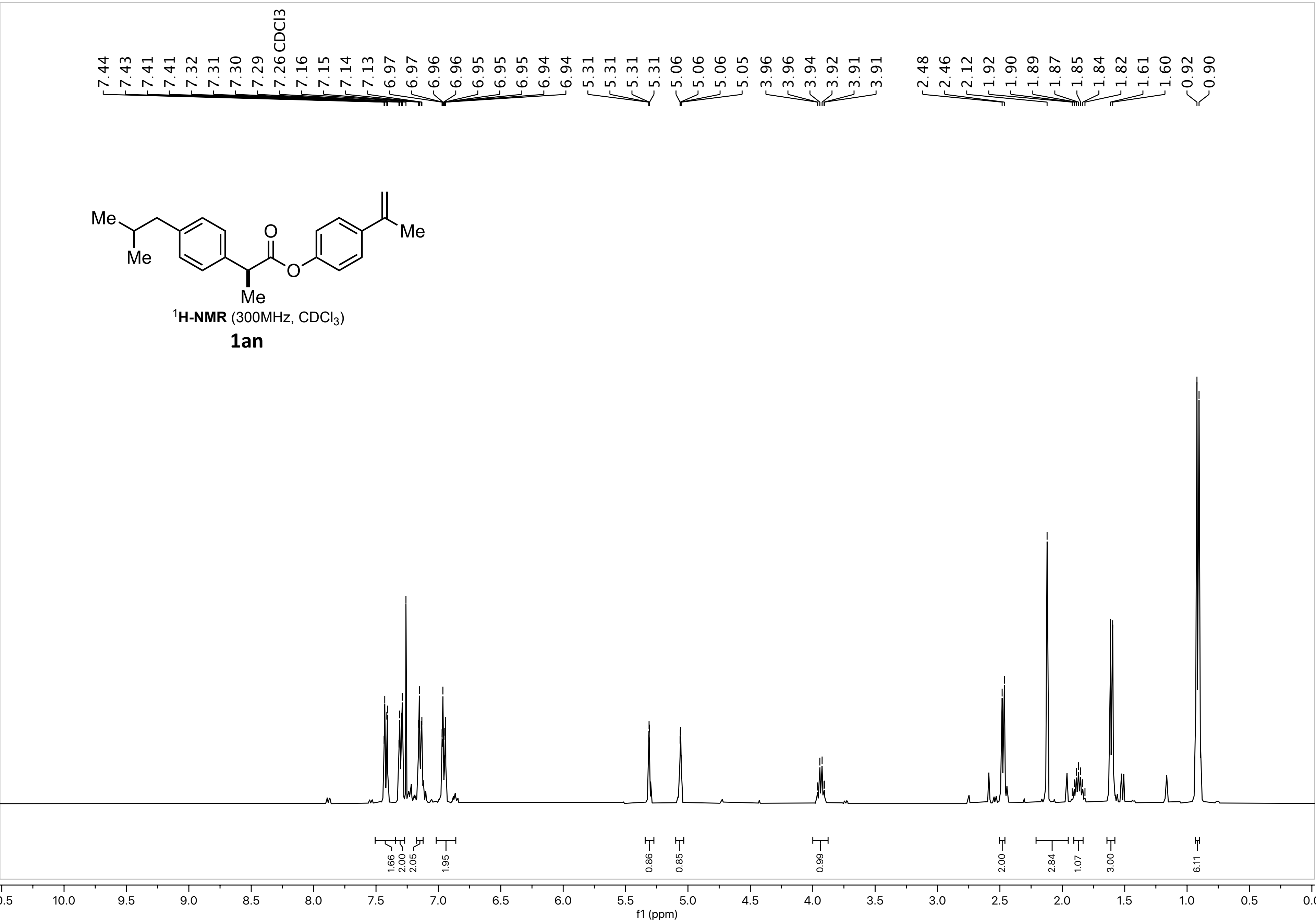

^{1}**H-NMR** (300MHz, $CDCl_3$)

1an

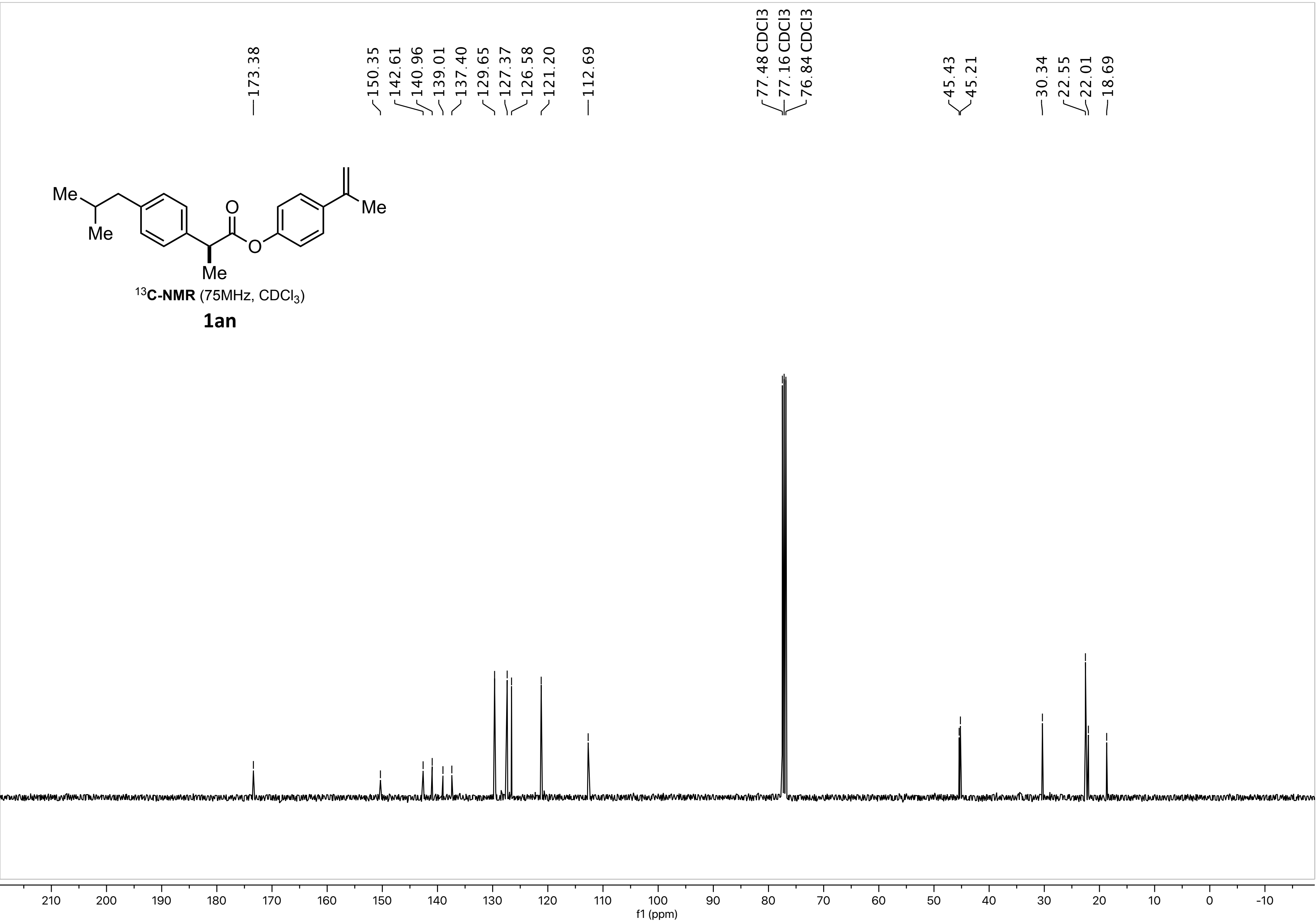

^{13}**C-NMR** (75MHz, $CDCl_3$)

1an

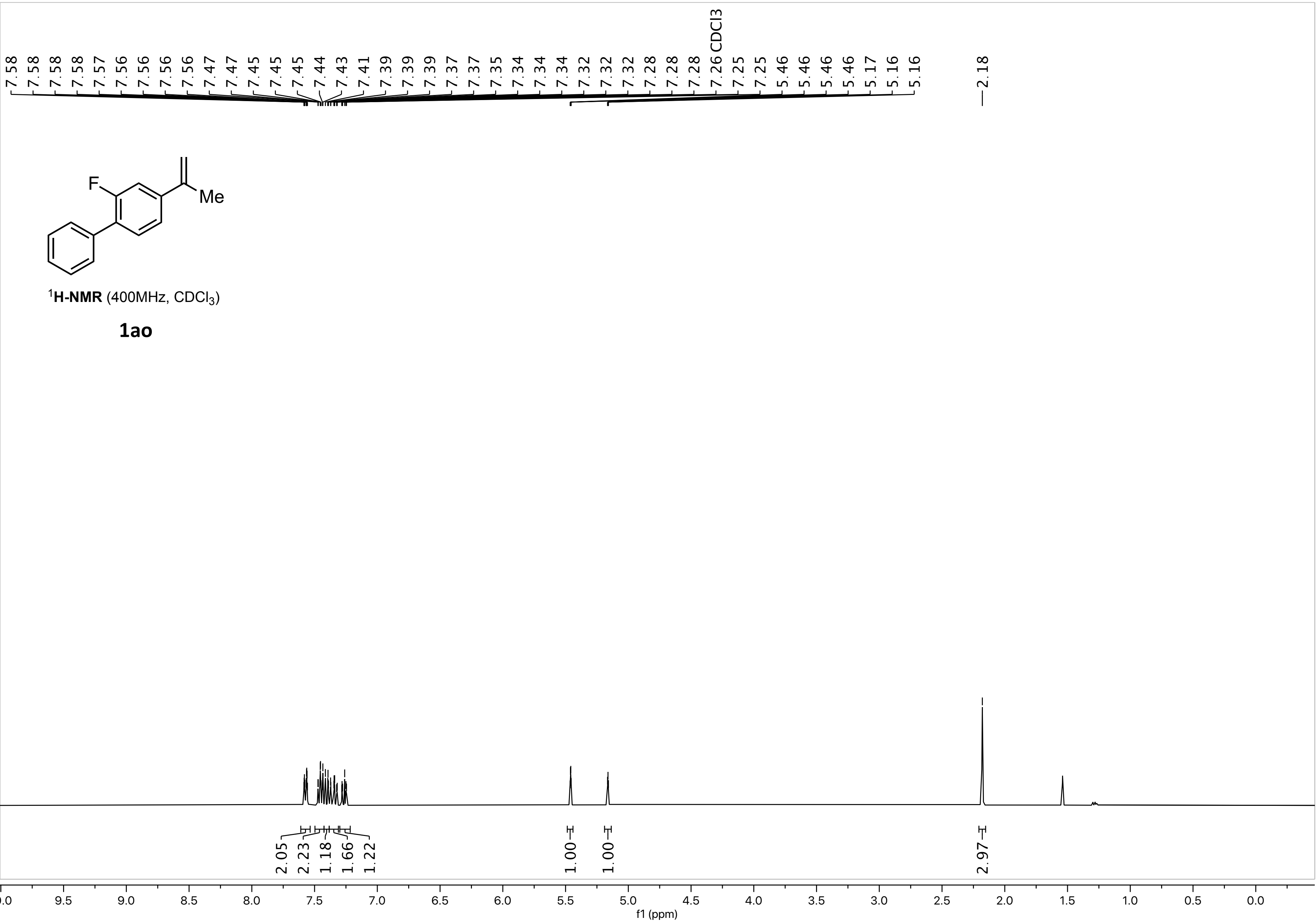
7.58 7.58 7.58 7.58 7.57 7.56 7.56 7.56 7.56 7.47 7.47 7.45 7.45 7.45 7.44 7.43 7.41 7.39 7.39 7.39 7.37 7.37 7.35 7.34 7.34 7.34 7.32 7.32 7.32 7.28 7.28 7.28 7.26 CDCl3 7.25 7.25 5.46 5.46 5.46 5.46 5.17 5.16 5.16
2.18
F
Me
1H-NMR (400MHz, CDCl3)
1ao
2.05 2.23 1.18 1.66 1.22
1.00
1.00
2.97
9.5 9.0 8.5 8.0 7.5 7.0 6.5 6.0 5.5 5.0 4.5 4.0 3.5 3.0 2.5 2.0 1.5 1.0 0.5 0.0
f1 (ppm)

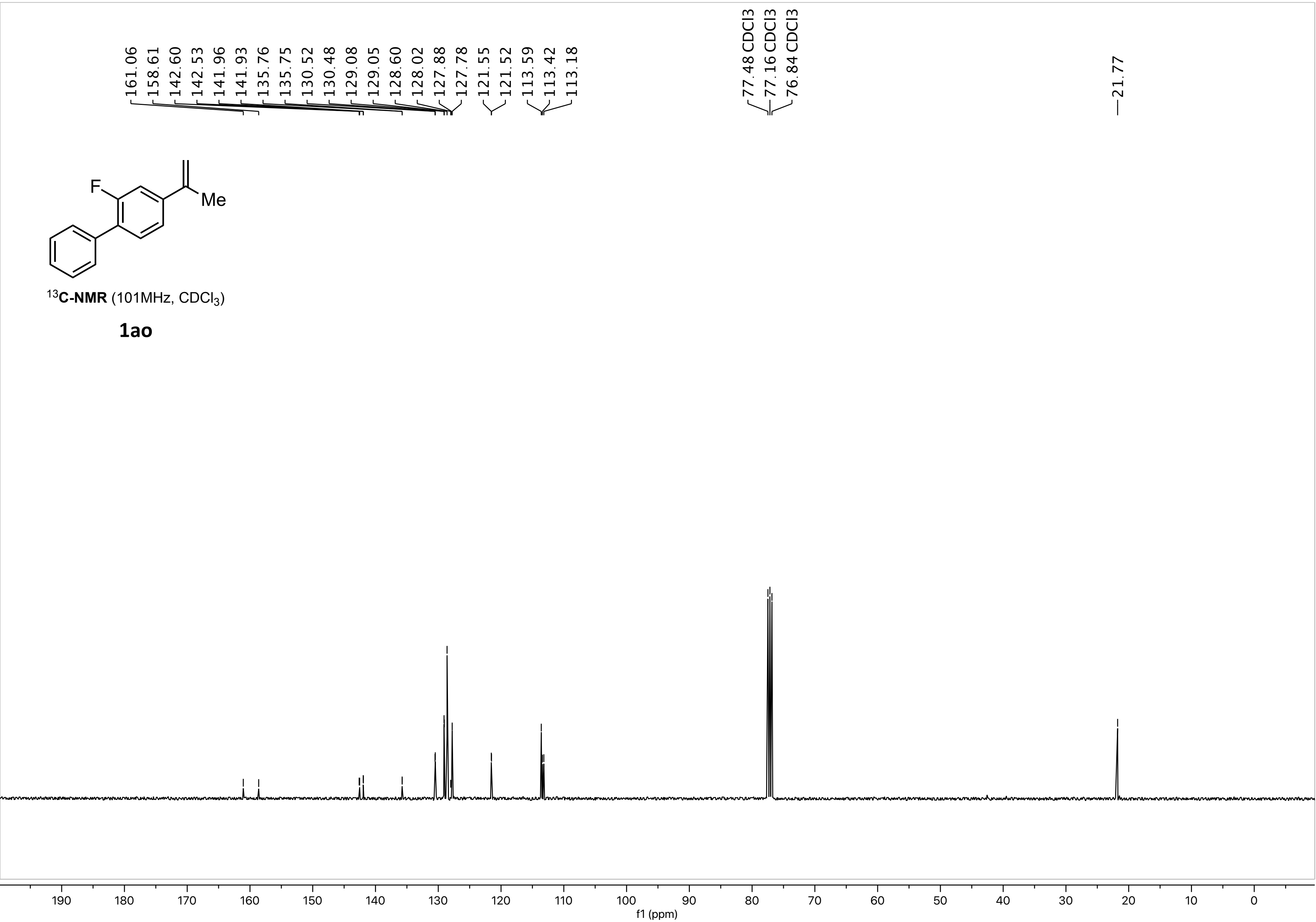

^{13}**C-NMR** (101MHz, $CDCl_3$)

1ao

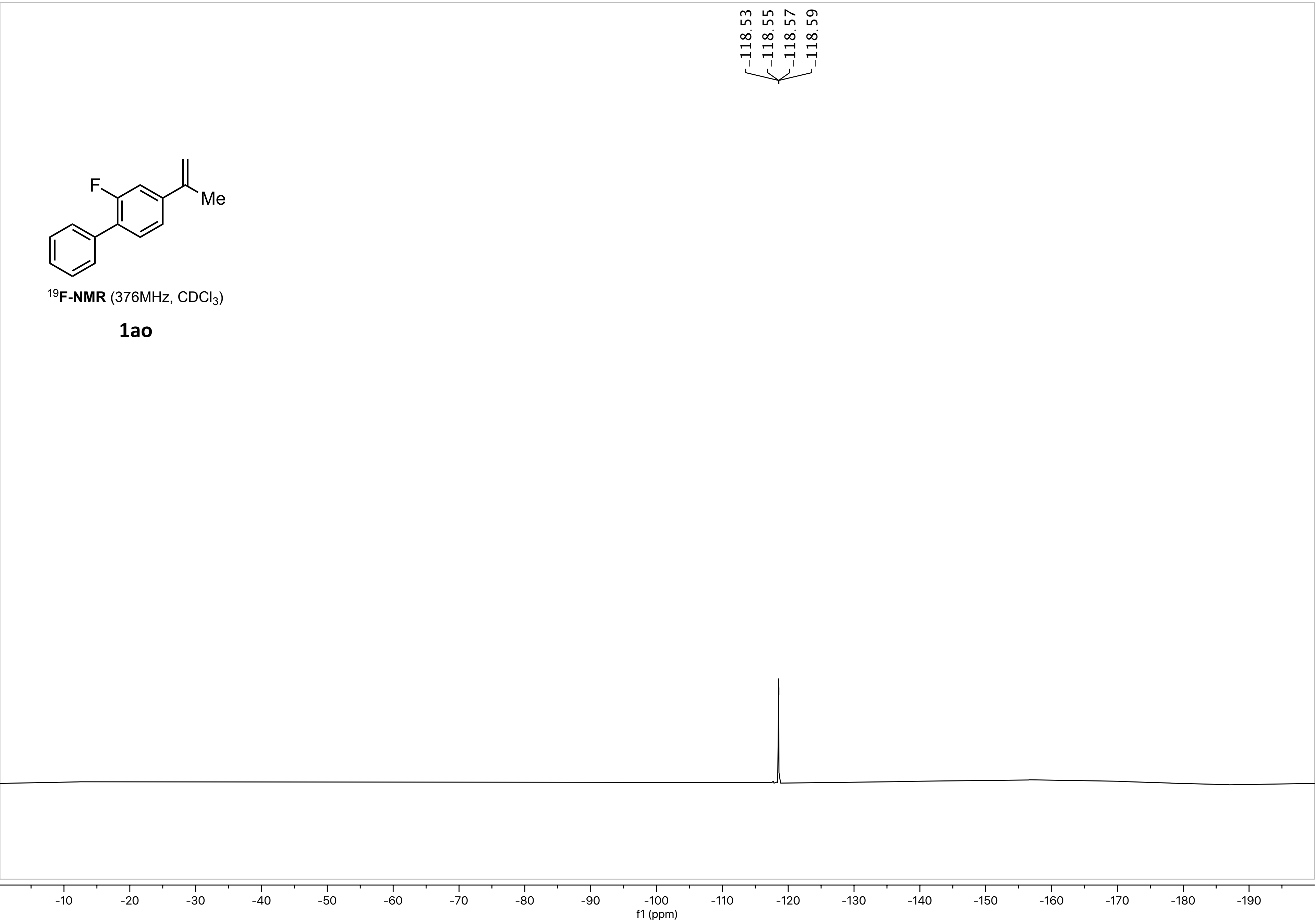

^{19}**F-NMR** (376MHz, $CDCl_3$)

1ao

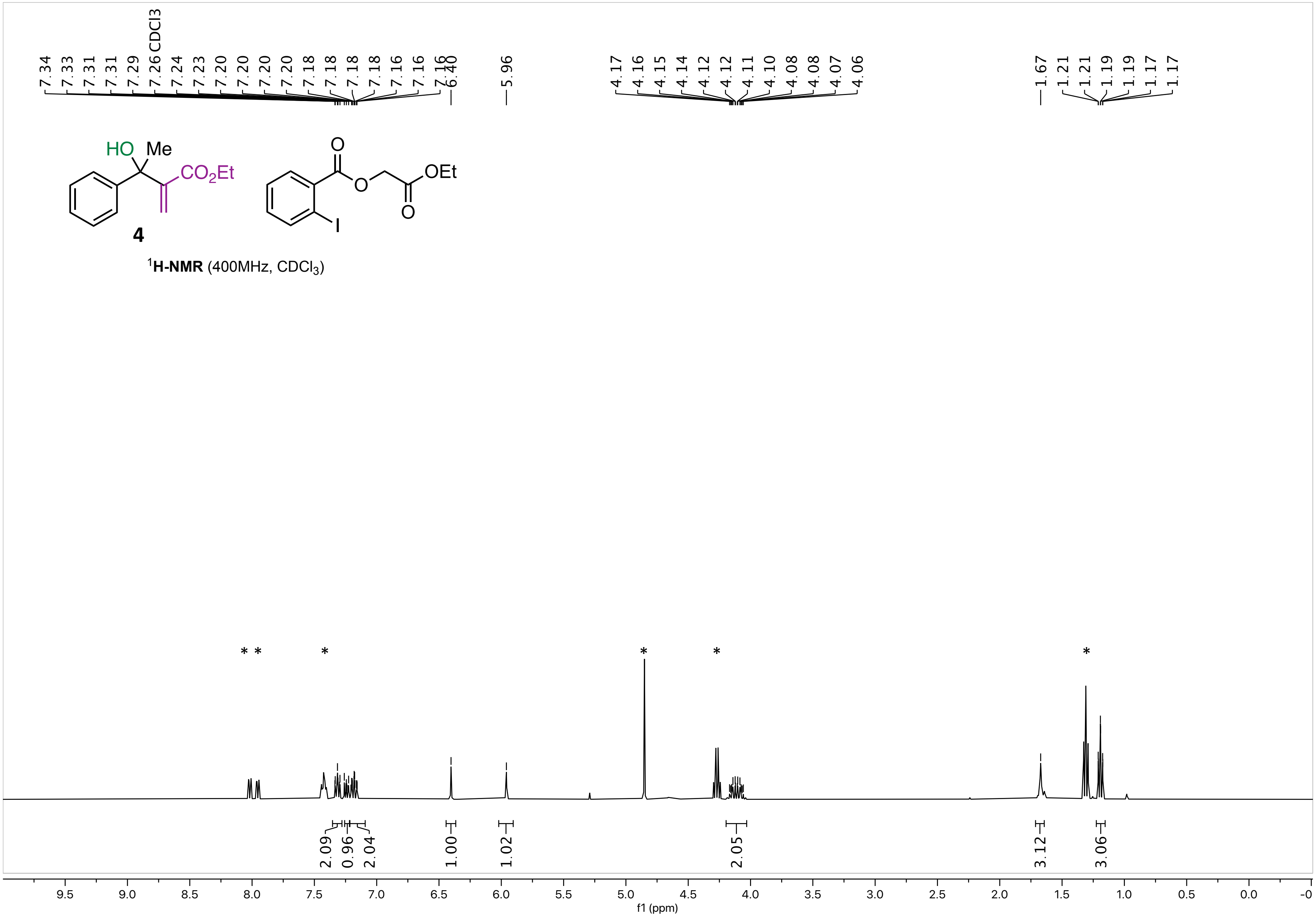
7.34 7.33 7.31 7.31 7.29 7.26 CDCl3 7.24 7.23 7.20 7.20 7.20 7.20 7.18 7.18 7.18 7.18 7.16 7.16 7.16 6.40
5.96
4.17 4.16 4.15 4.14 4.12 4.12 4.11 4.10 4.08 4.08 4.07 4.06
1.67 1.21 1.21 1.19 1.19 1.17 1.17
HO
Me
CO2Et
4
O
O
OEt
O
I
1H-NMR (400MHz, CDCl3)
2.09
0.96
2.04
1.00
1.02
2.05
3.12
3.06
9.5 9.0 8.5 8.0 7.5 7.0 6.5 6.0 5.5 5.0 4.5 4.0 3.5 3.0 2.5 2.0 1.5 1.0 0.5 0.0
f1 (ppm)

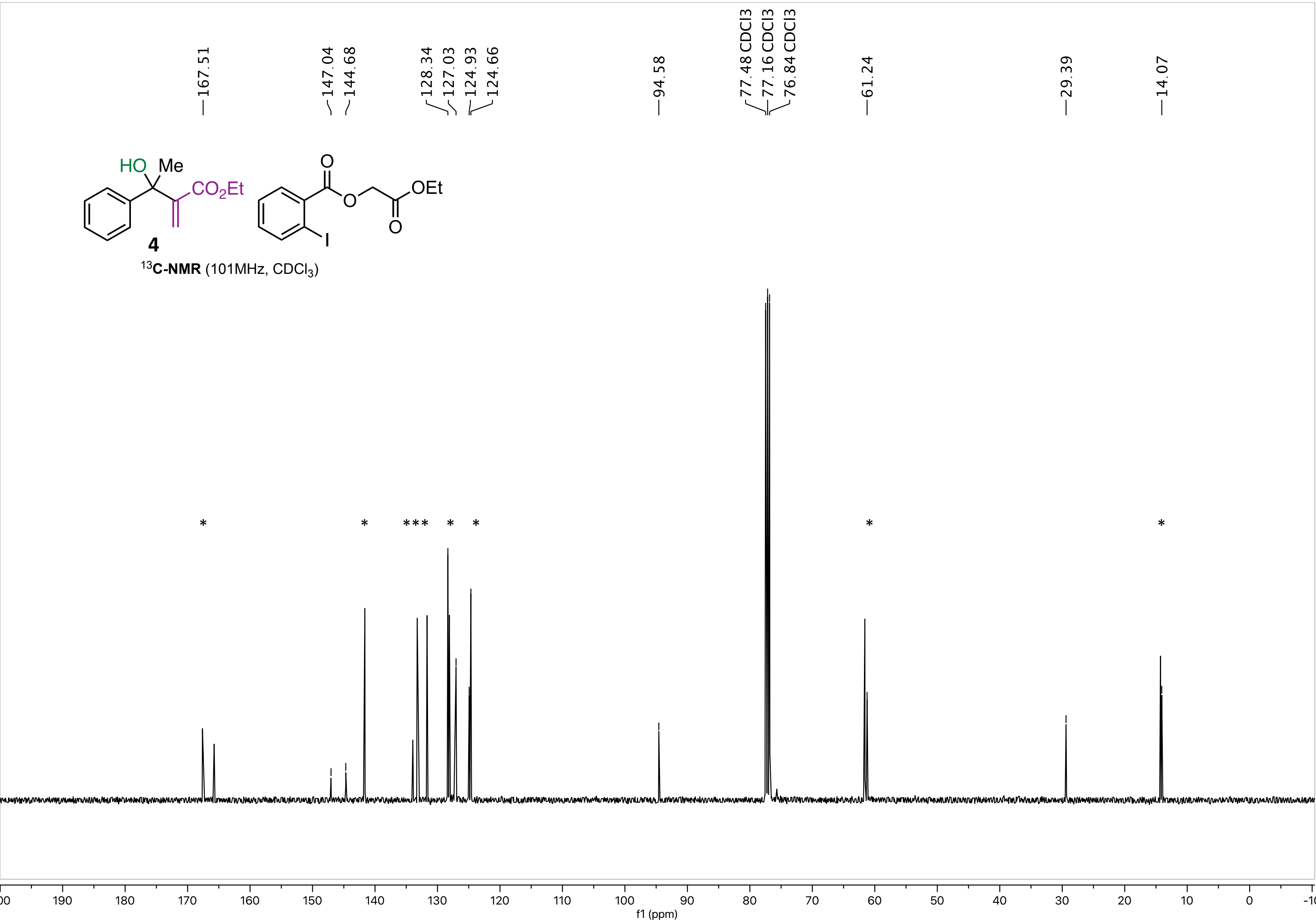

167.51
147.04
144.68
128.34
127.03
124.93
124.66
94.58
77.48 CDCl3
77.16 CDCl3
76.84 CDCl3
61.24
29.39
14.07
HO Me
CO2Et
4
O
O
OEt
O
I
13C-NMR (101MHz, CDCl3)
f1 (ppm)

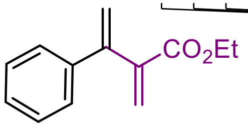

^{1}**H-NMR** (400MHz, $CDCl_3$)

5

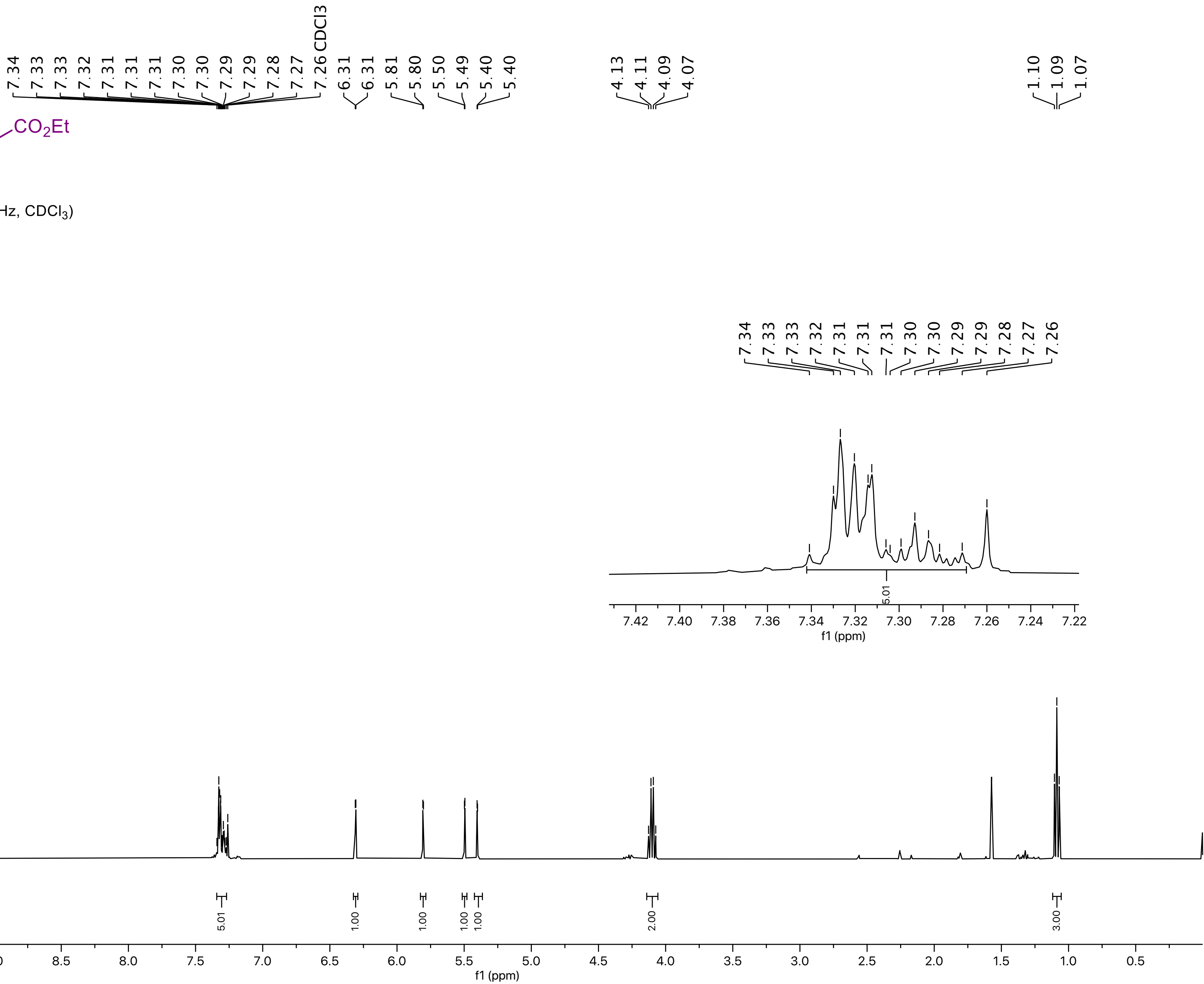

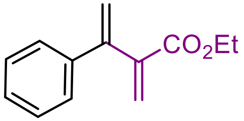

^{13}**C-NMR** (101MHz, $CDCl_3$)

5

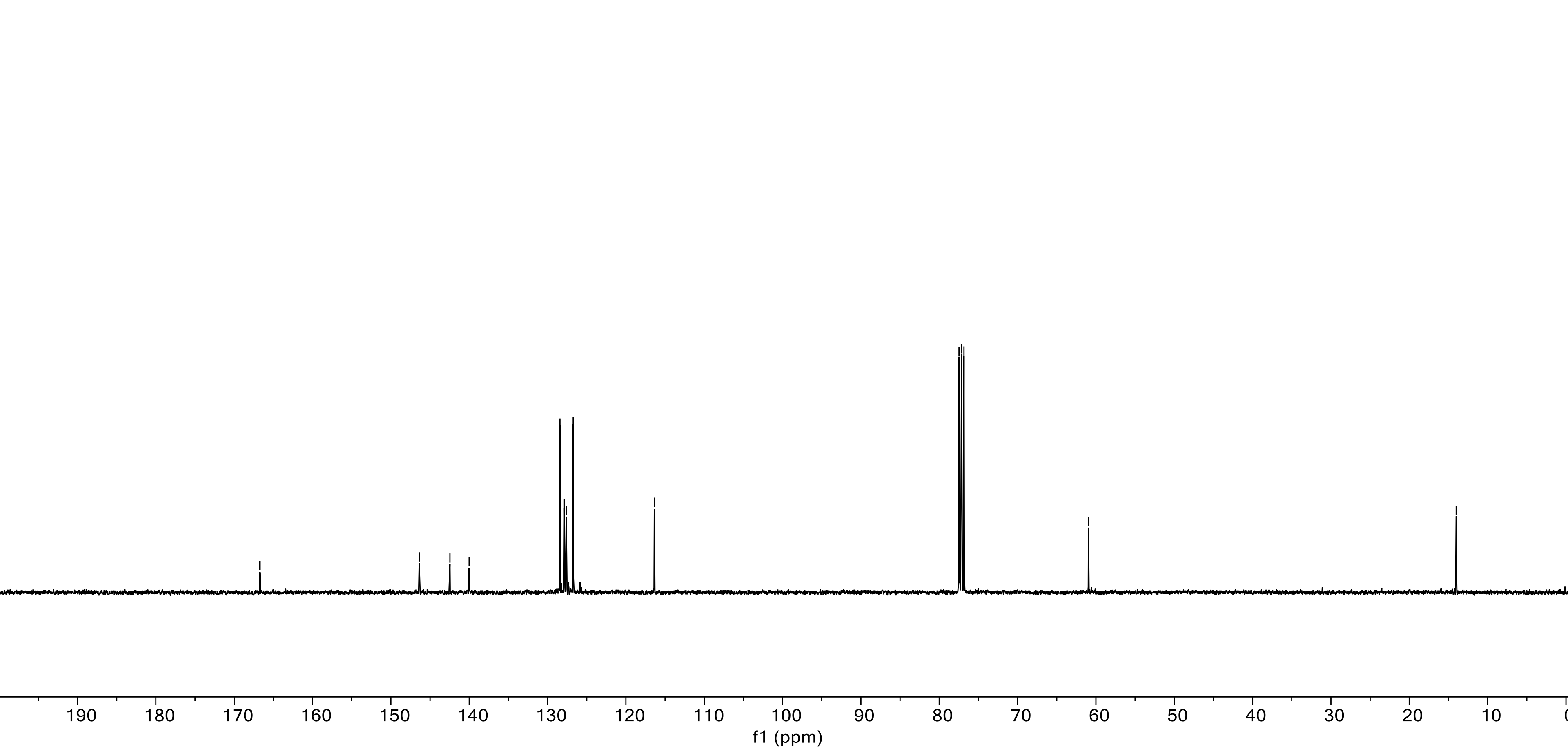

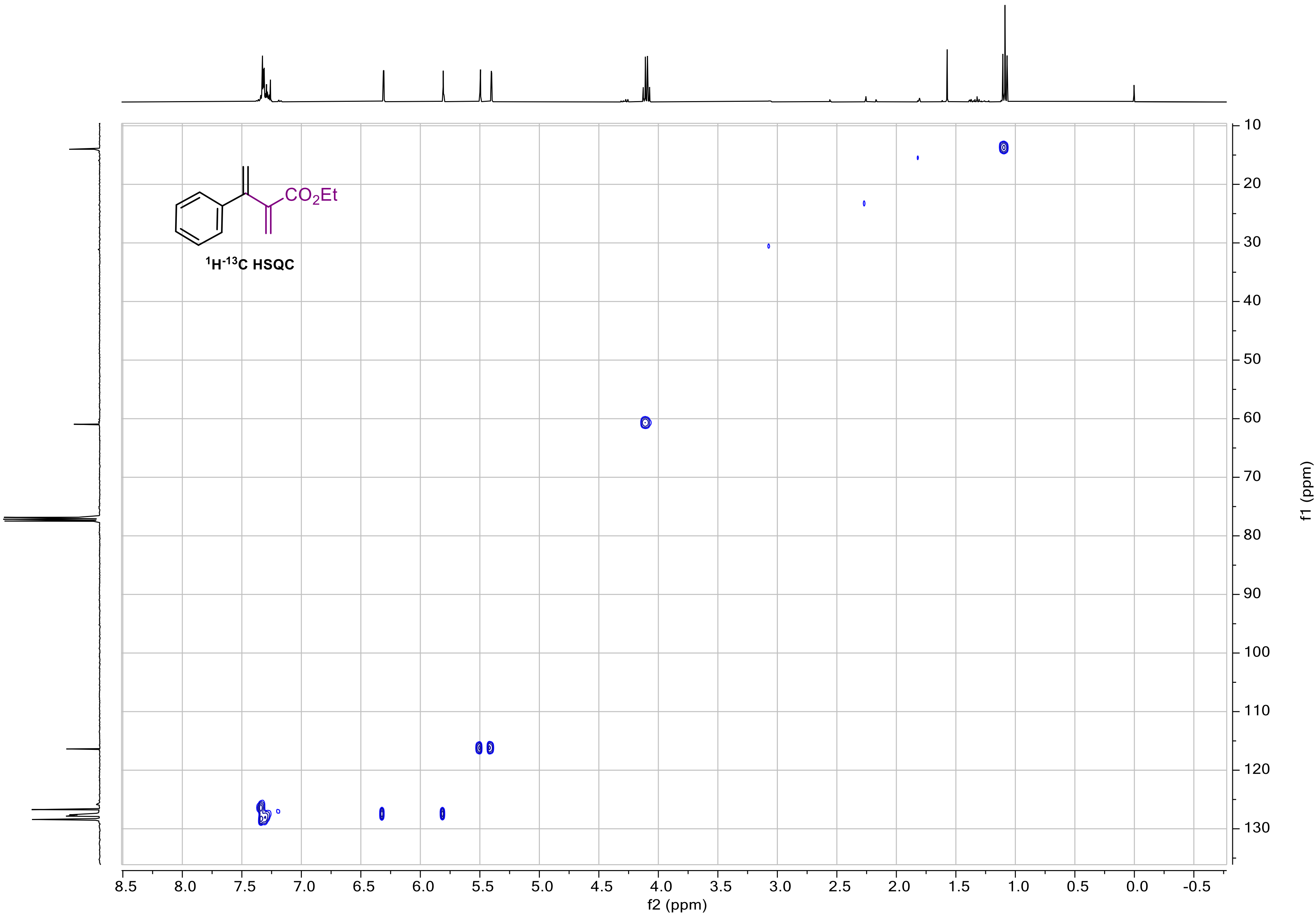

CO2Et
1H-13C HSQC
8.5
8.0
7.5
7.0
6.5
6.0
5.5
5.0
4.5
4.0
3.5
3.0
2.5
2.0
1.5
1.0
0.5
0.0
-0.5
f2 (ppm)
10
20
30
40
50
60
70
80
90
100
110
120
130
f1 (ppm)

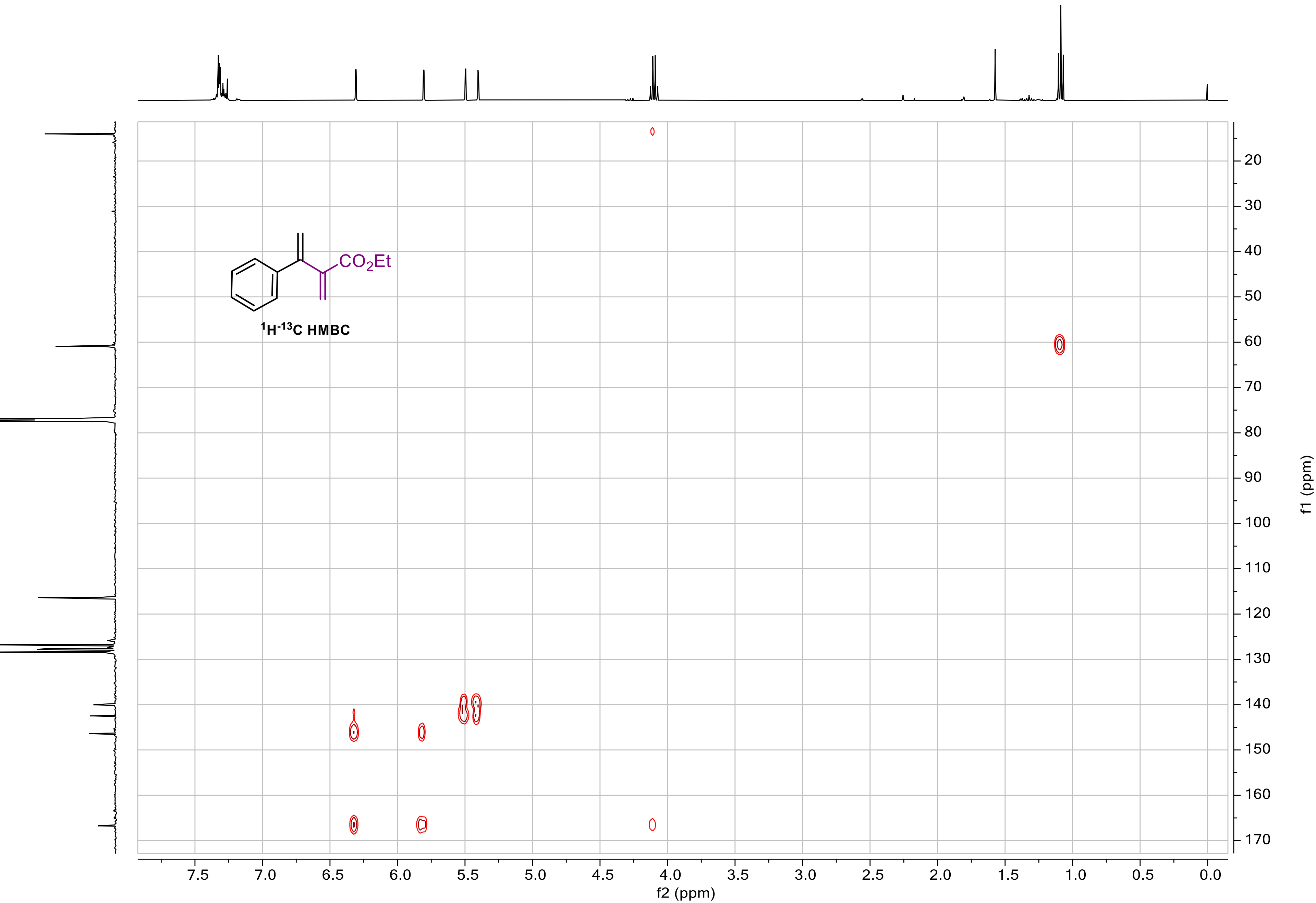

CO2Et
1H-13C HMBC
f2 (ppm)
f1 (ppm)

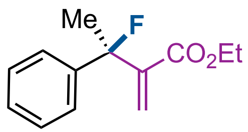

^{1}**H-NMR** (500MHz, $CDCl_3$)

3a

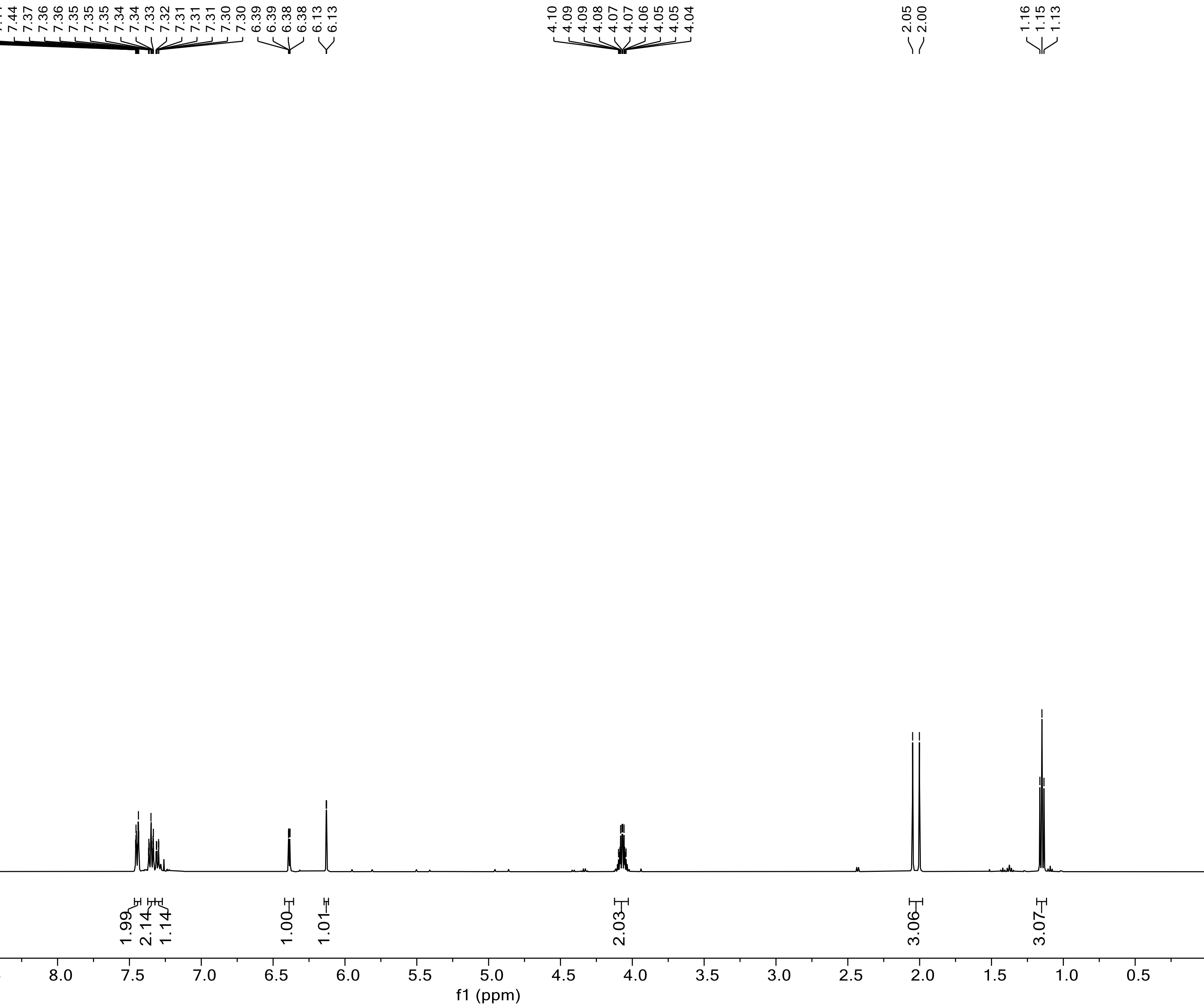

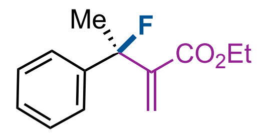

^{13}**C-NMR** (126MHz, $CDCl_3$)

3a

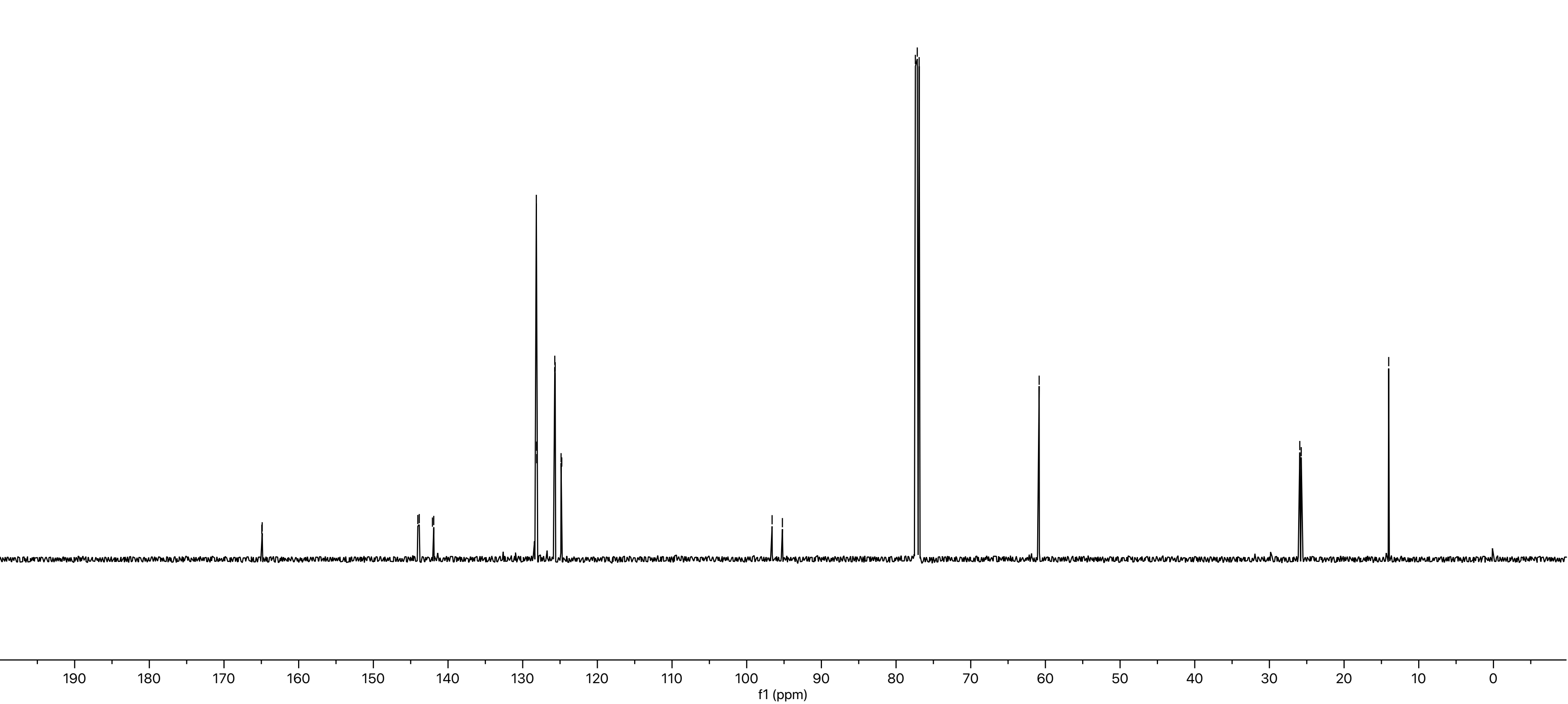

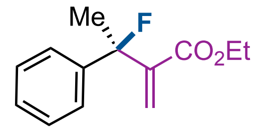

^{19}**F-NMR** (376MHz, $CDCl_3$)

3a

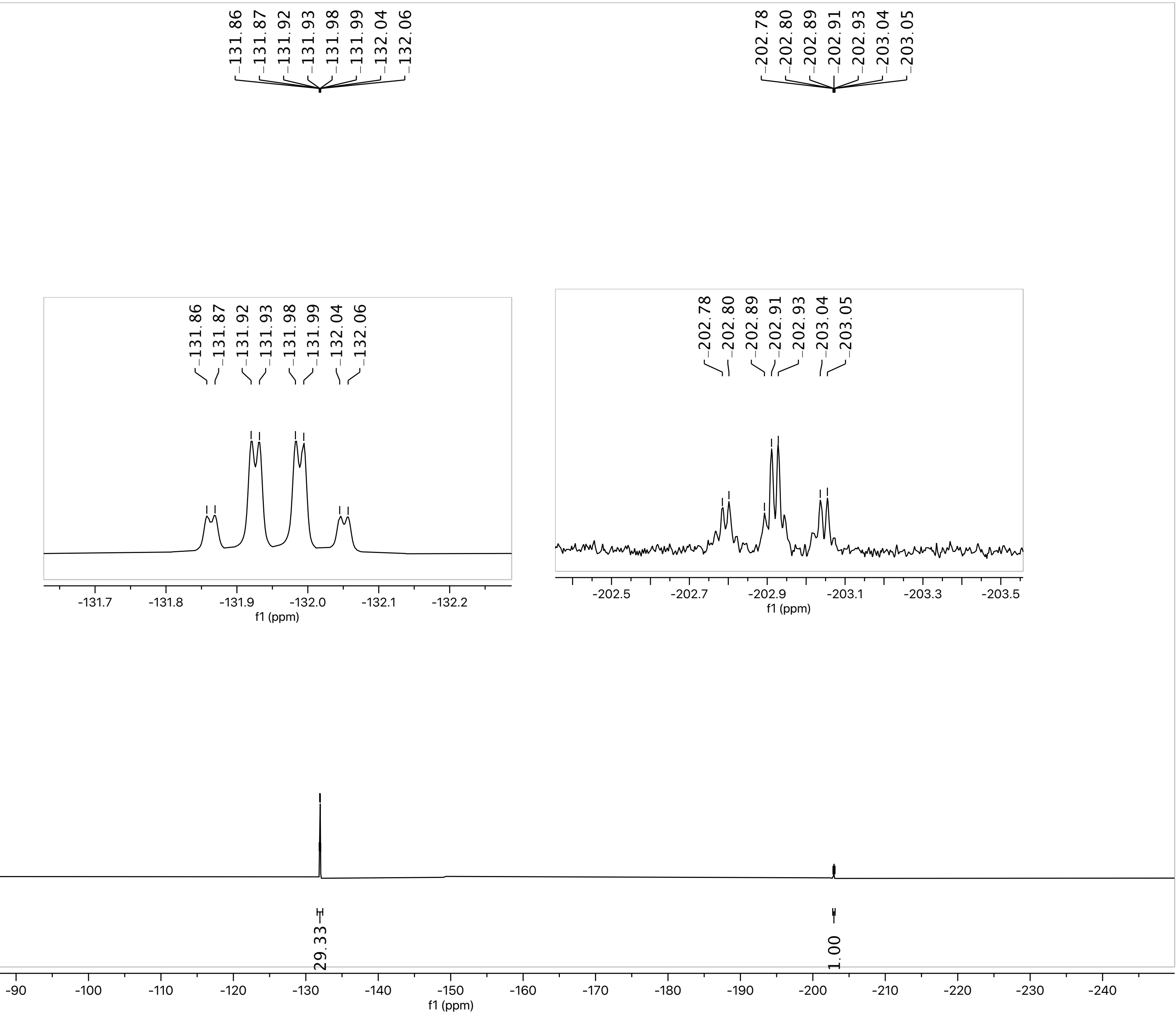

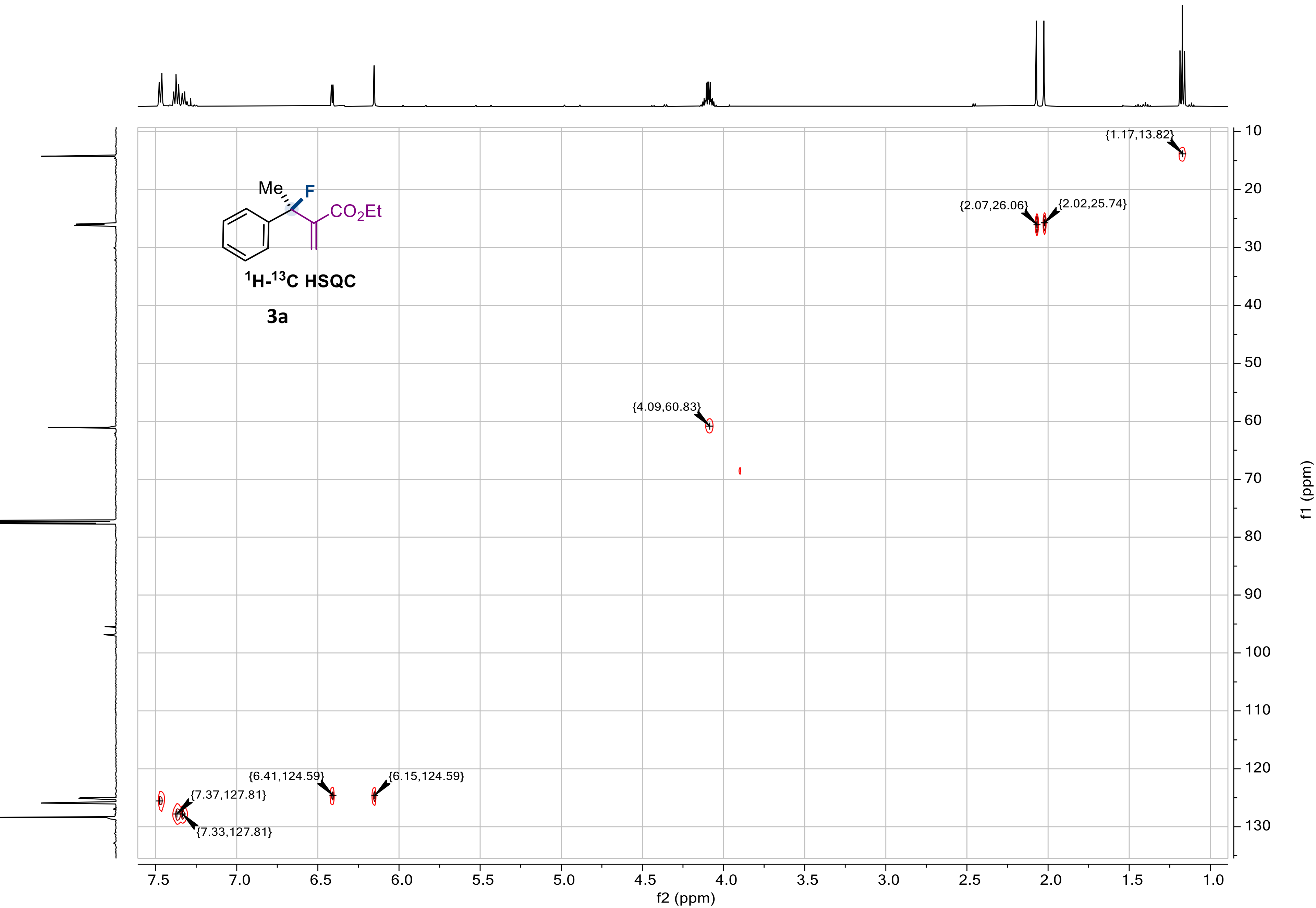
Me
F
CO2Et
1H-13C HSQC
3a
{1.17,13.82}
{2.07,26.06}
{2.02,25.74}
{4.09,60.83}
{6.41,124.59}
{6.15,124.59}
{7.37,127.81}
{7.33,127.81}
f2 (ppm)
f1 (ppm)

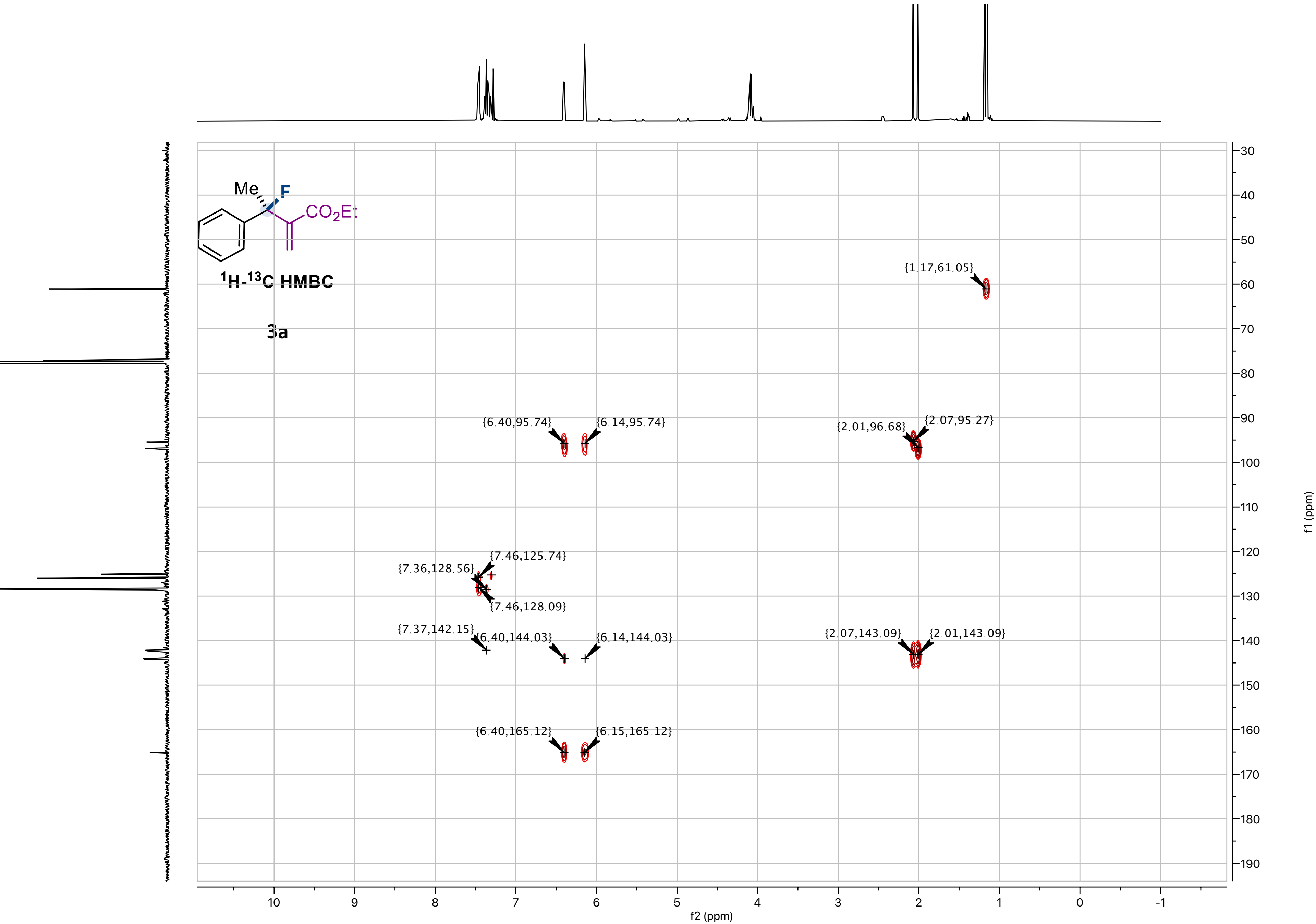
Me
F
CO2Et
1H-13C HMBC
3a
{1.17,61.05}
{6.40,95.74}
{6.14,95.74}
{2.01,96.68}
{2.07,95.27}
{7.46,125.74}
{7.36,128.56}
{7.46,128.09}
{7.37,142.15}
{6.40,144.03}
{6.14,144.03}
{2.07,143.09}
{2.01,143.09}
{6.40,165.12}
{6.15,165.12}
f2 (ppm)
f1 (ppm)

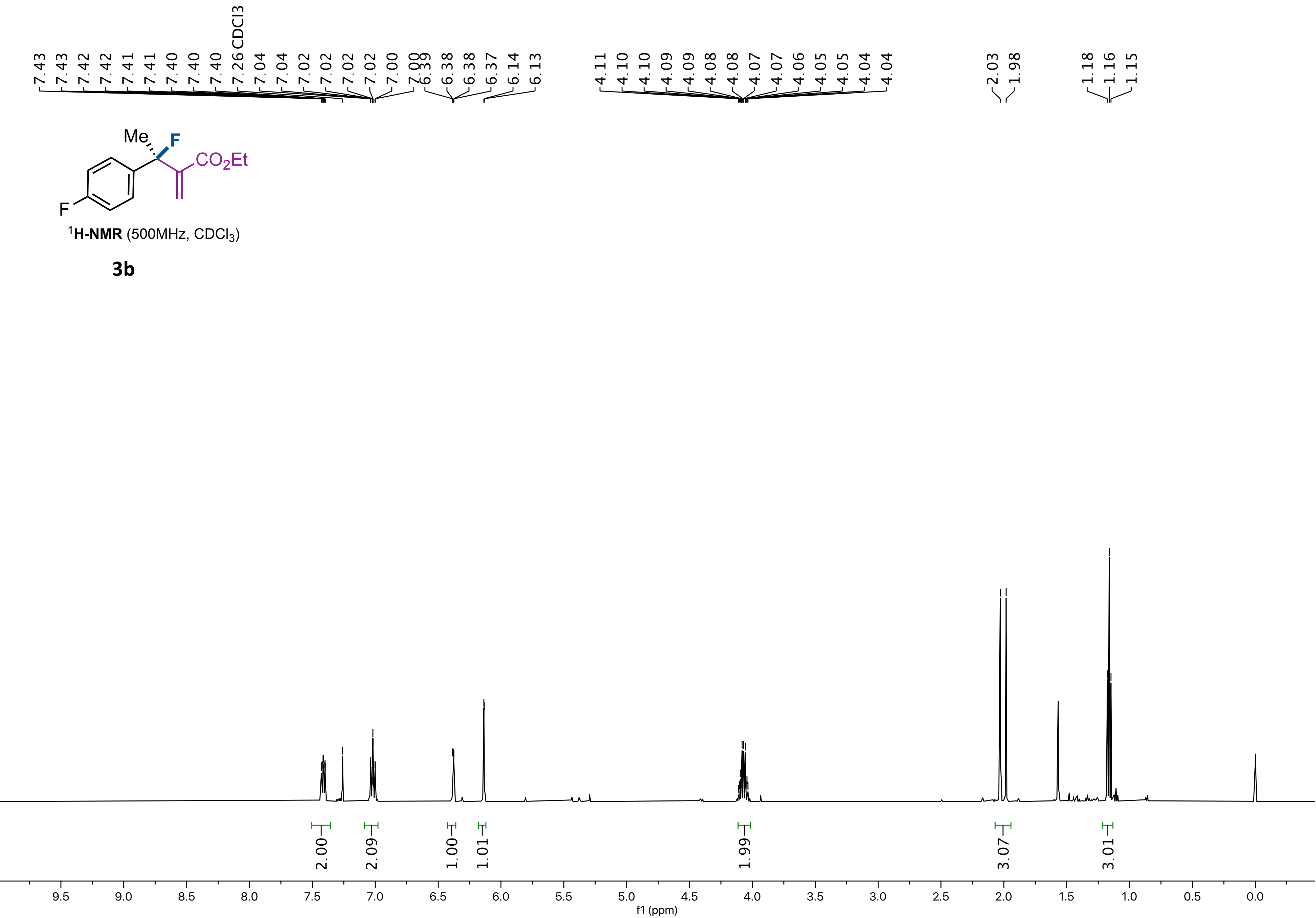

[1]**H-NMR** (500MHz, $CDCl_3$)

3b

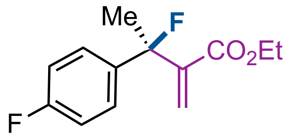

^{13}**C-NMR** (126MHz, $CDCl_3$)

3b

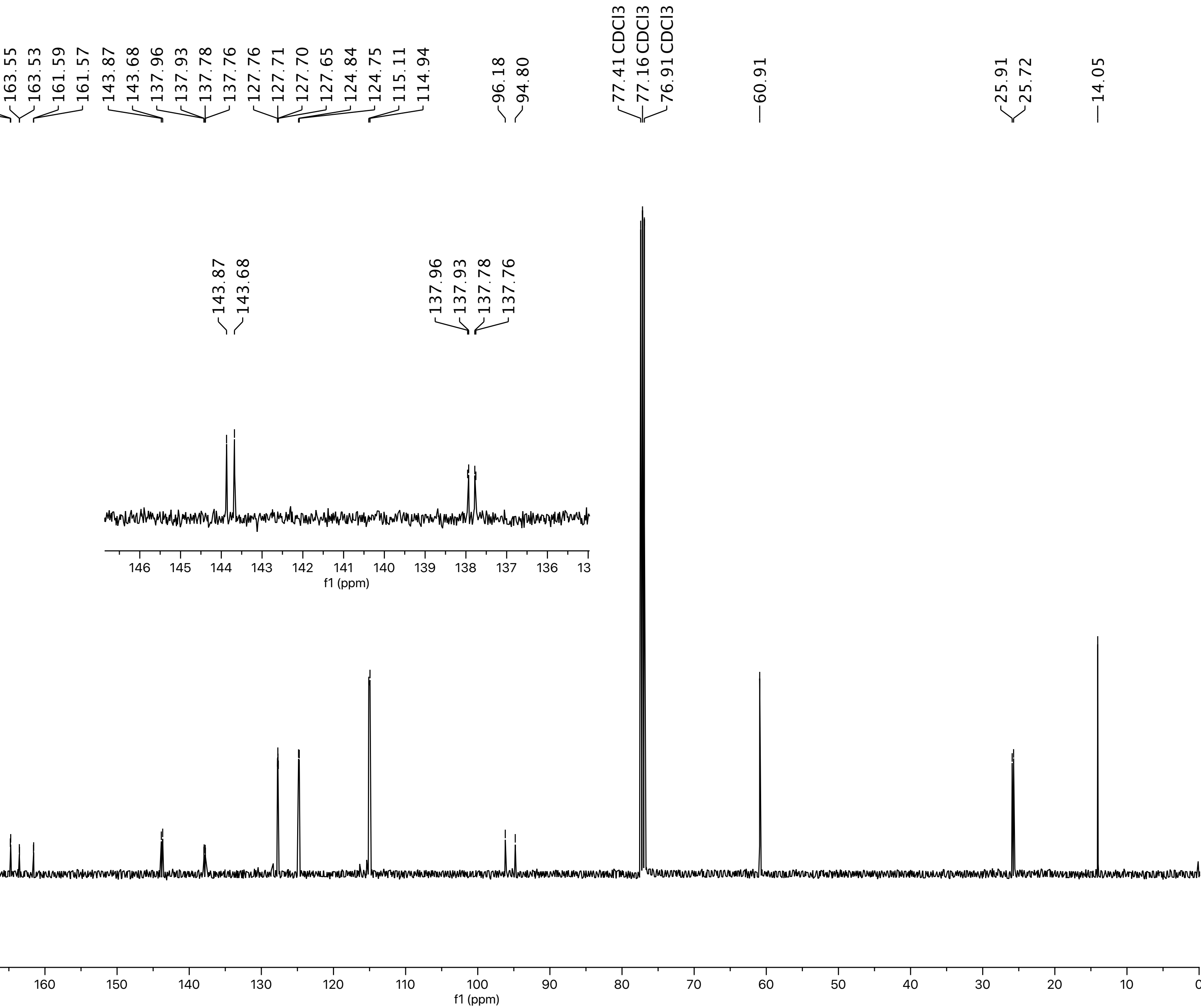

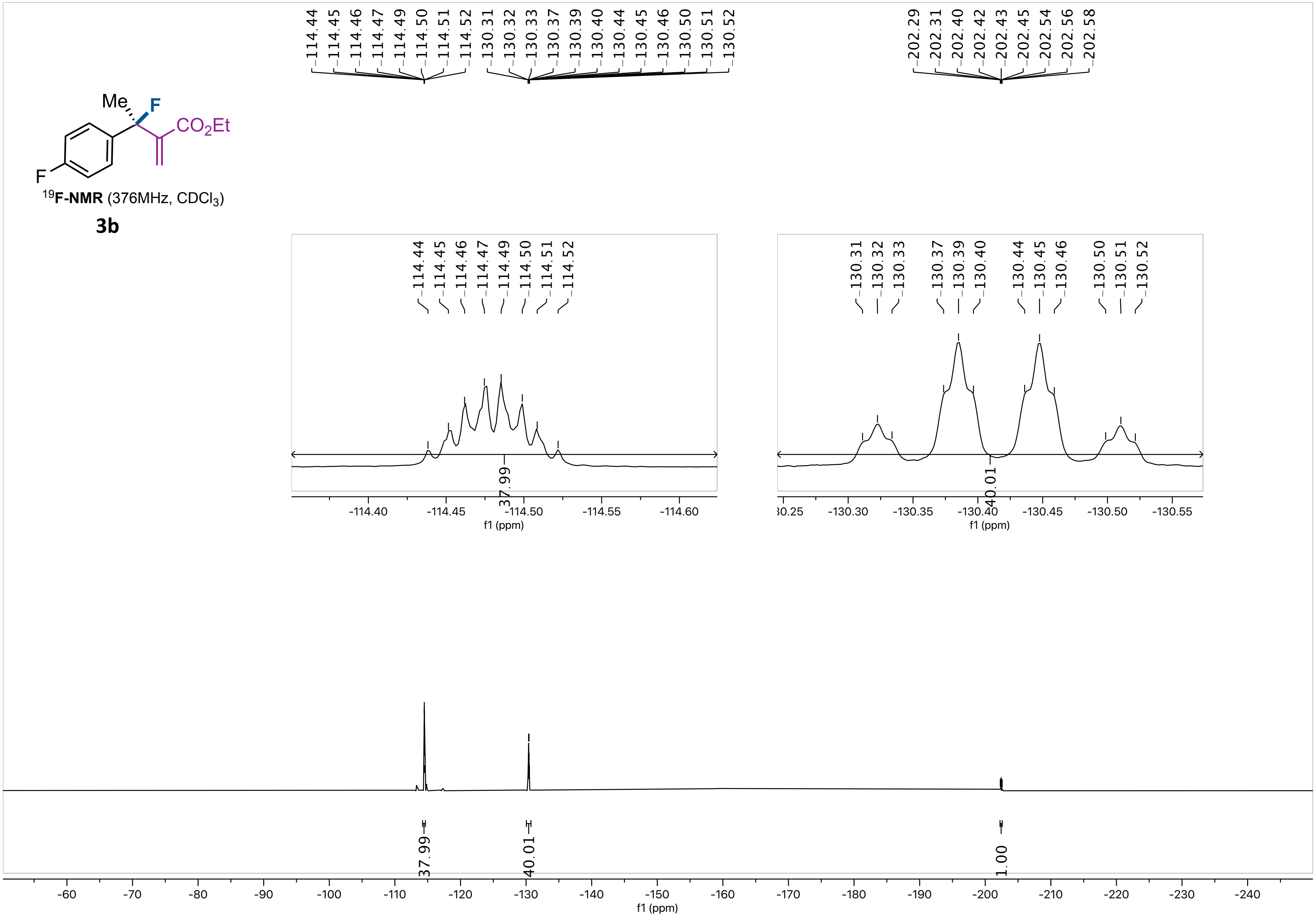
Me
F
CO2Et
F
19F-NMR (376MHz, CDCl3)
3b
-114.44
-114.45
-114.46
-114.47
-114.49
-114.50
-114.51
-114.52
-130.31
-130.32
-130.33
-130.37
-130.39
-130.40
-130.44
-130.45
-130.46
-130.50
-130.51
-130.52
-202.29
-202.31
-202.40
-202.42
-202.43
-202.45
-202.54
-202.56
-202.58
37.99
40.01
1.00
f1 (ppm)

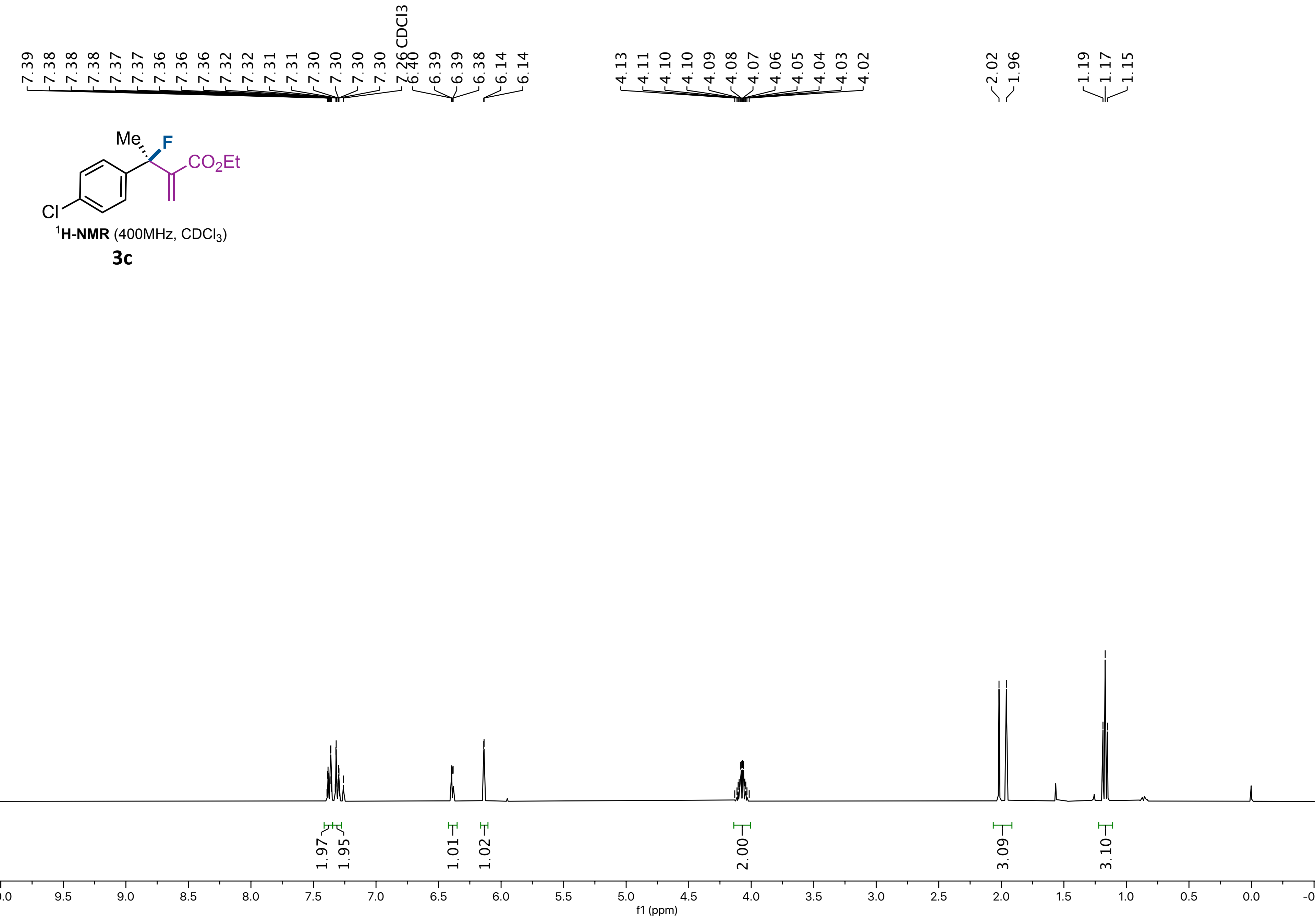

7.39
7.38
7.38
7.38
7.37
7.37
7.36
7.36
7.36
7.32
7.32
7.31
7.31
7.30
7.30
7.30
7.30
7.26 CDCl3
6.40
6.39
6.39
6.38
6.14
6.14
4.13
4.11
4.10
4.10
4.09
4.08
4.07
4.06
4.05
4.04
4.03
4.02
2.02
1.96
1.19
1.17
1.15
Me
F
CO2Et
Cl
1H-NMR (400MHz, CDCl3)
3c
1.97
1.95
1.01
1.02
2.00
3.09
3.10
f1 (ppm)

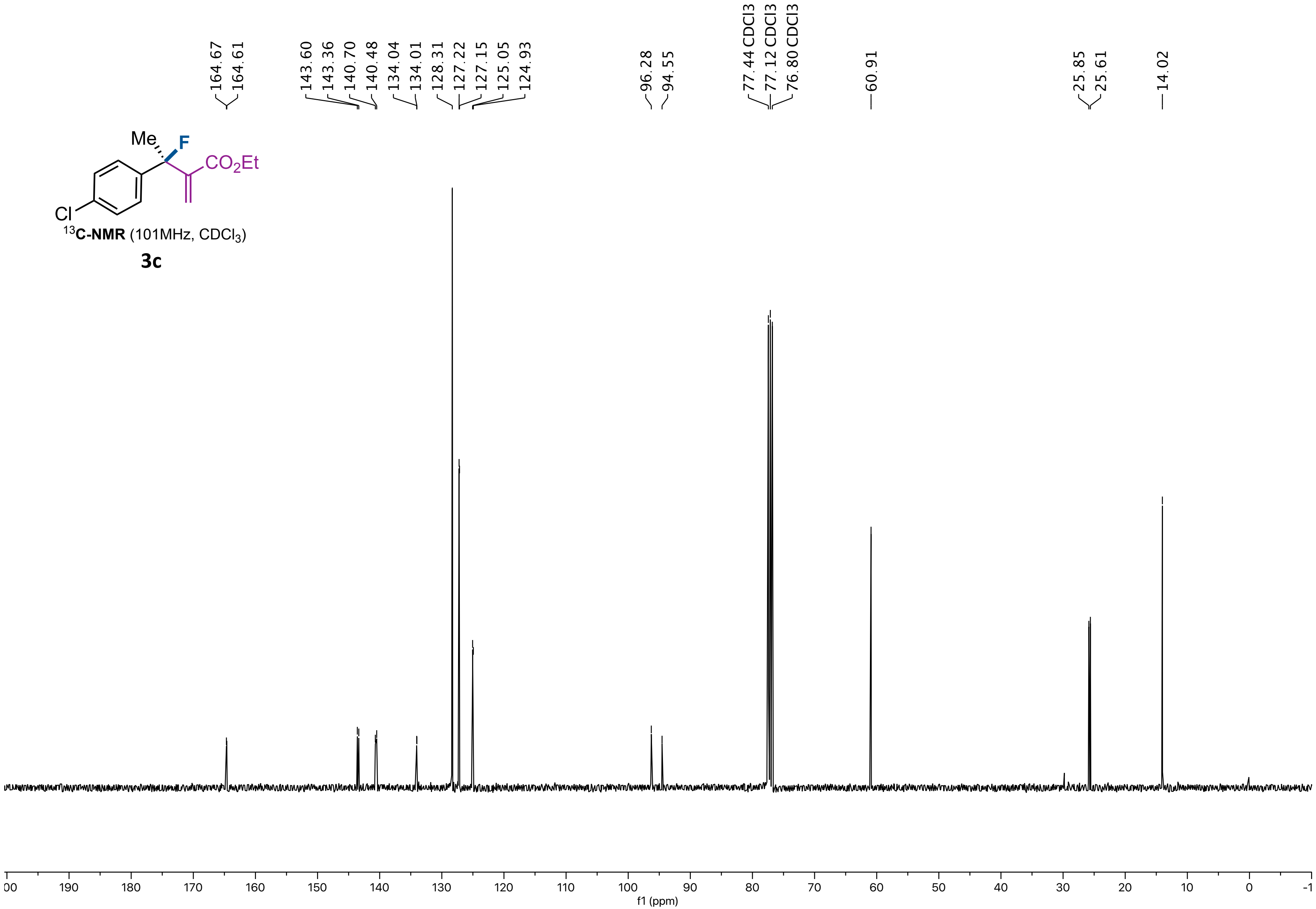

^{13}**C-NMR** (101MHz, $CDCl_3$)

3c

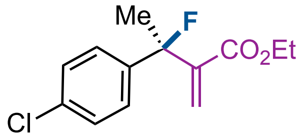

^{19}**F-NMR** (376MHz, $CDCl_3$)

3c

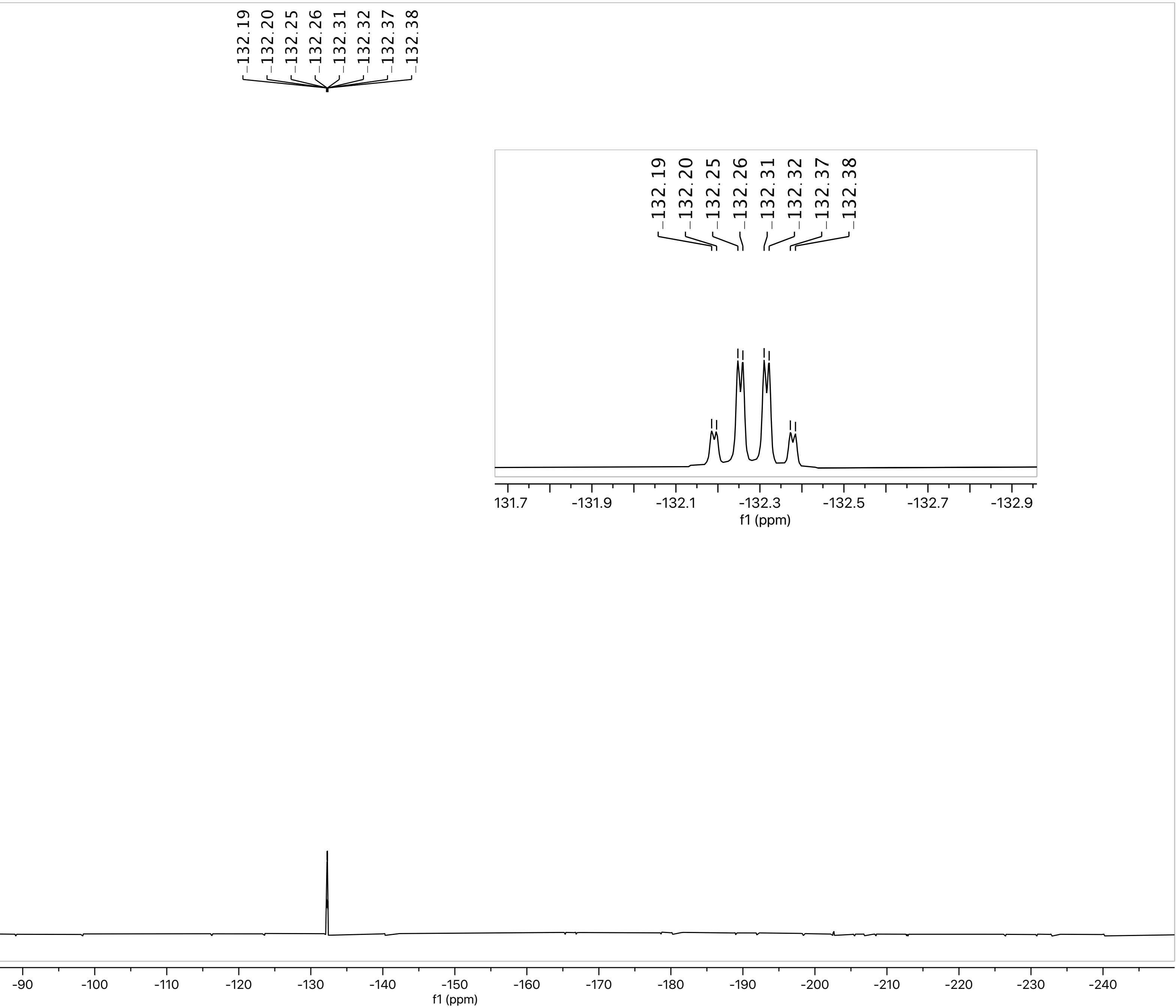

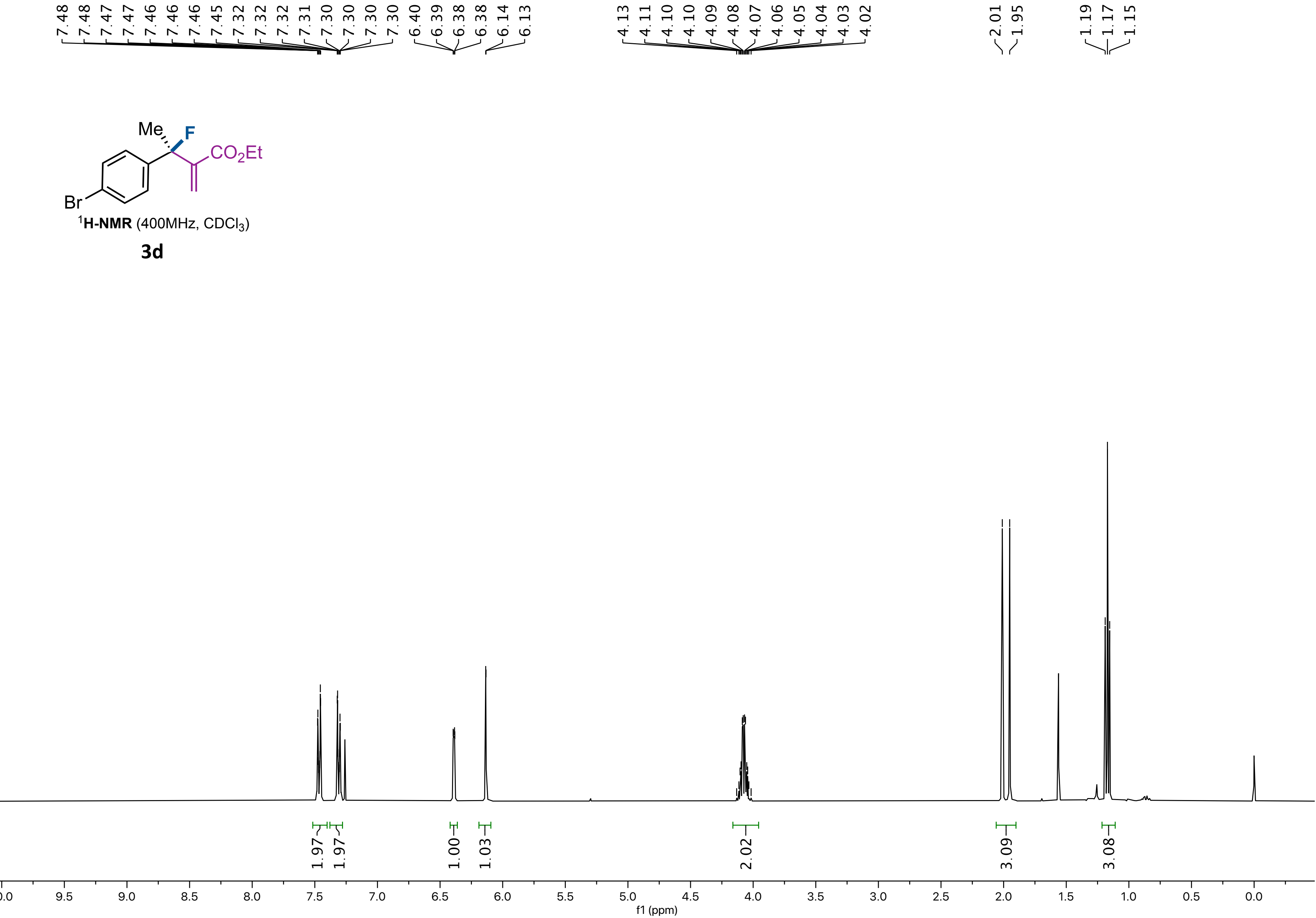

^{1}**H-NMR** (400MHz, $CDCl_3$)

3d

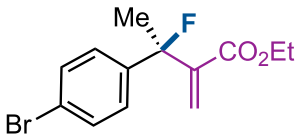

[19]**C-NMR** (101MHz, $CDCl_3$)

3d

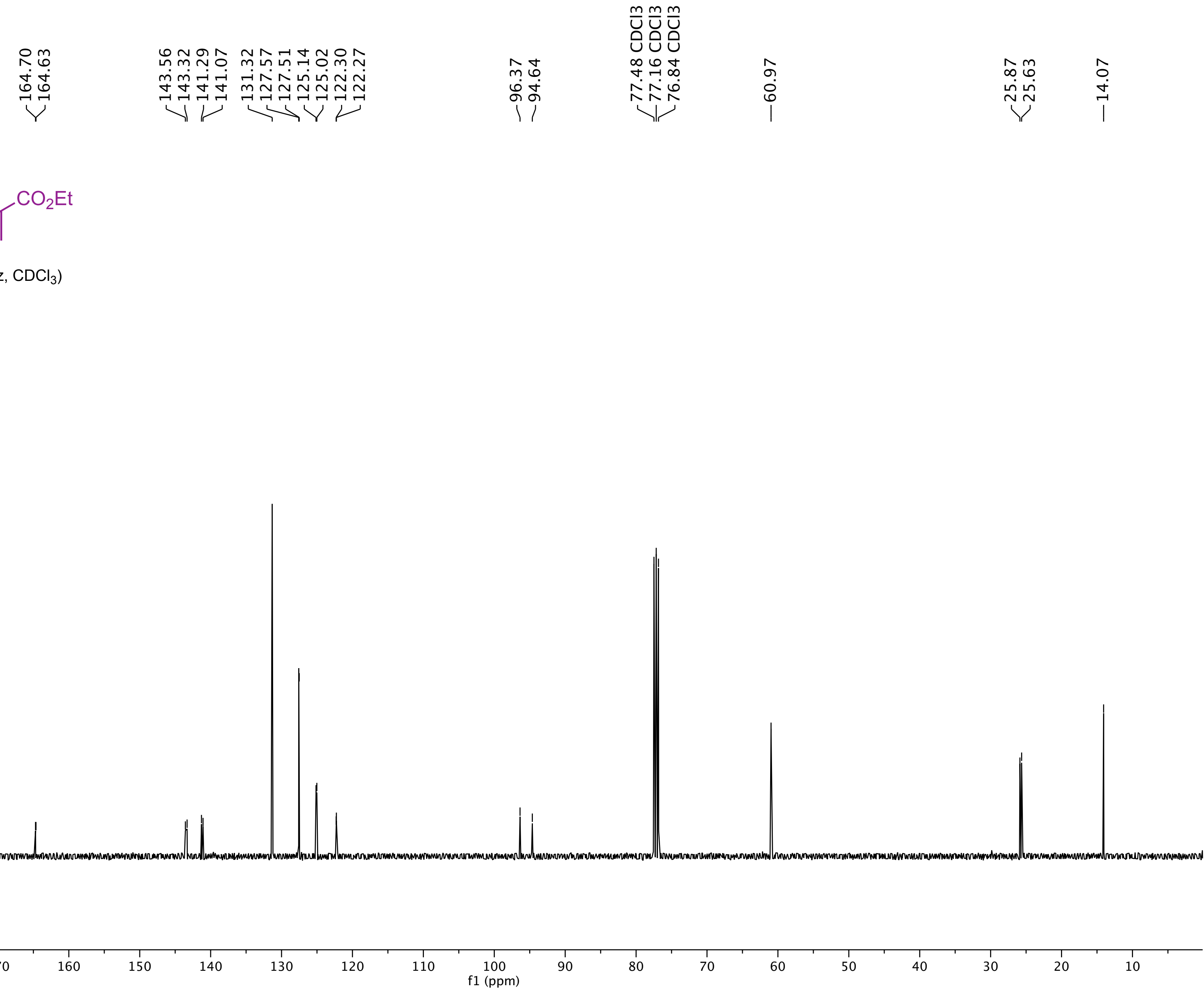

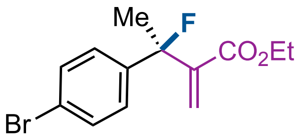

^{19}**F-NMR** (376MHz, $CDCl_3$)

3d

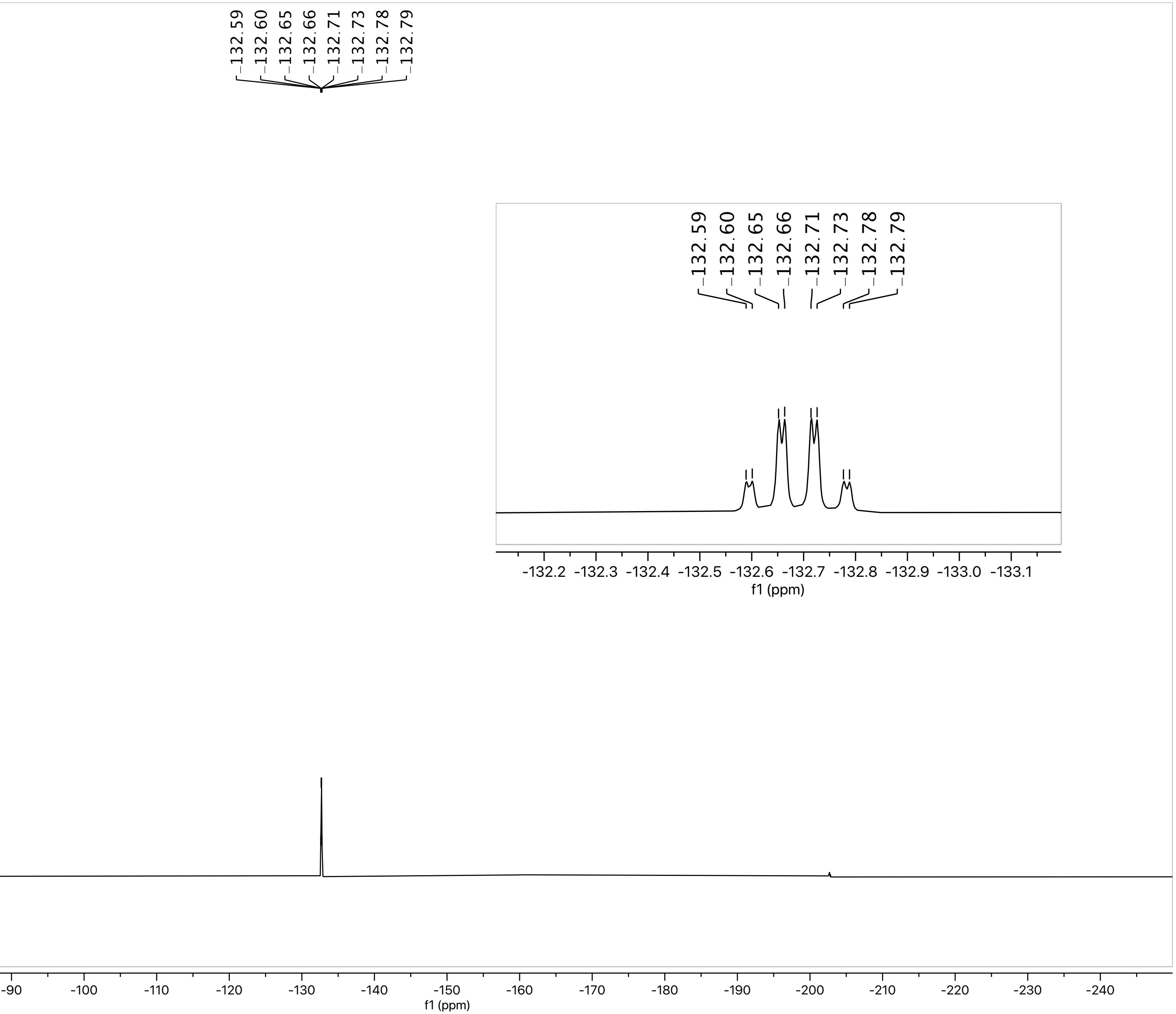

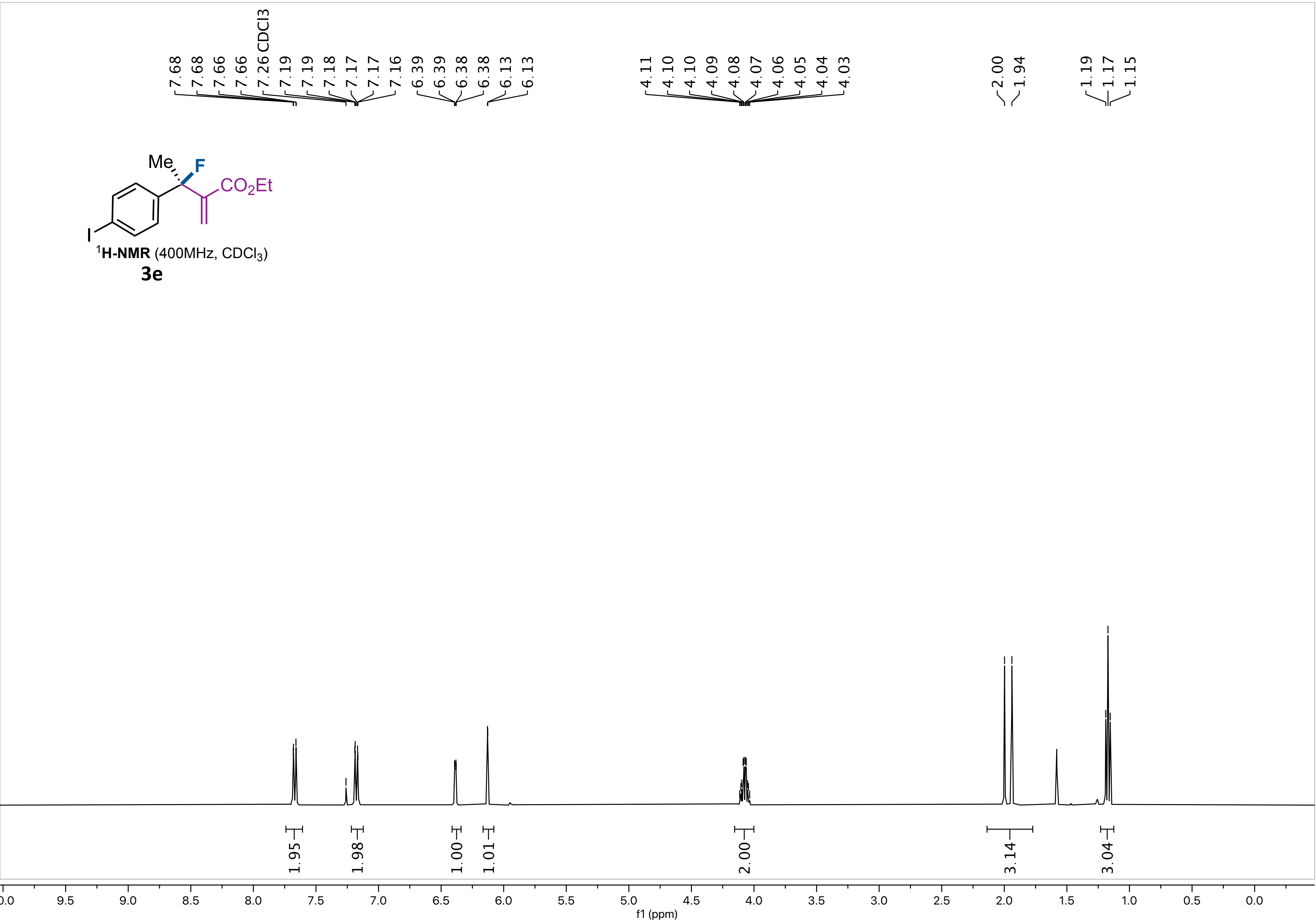

^{1}**H-NMR** (400MHz, $CDCl_3$)

3e

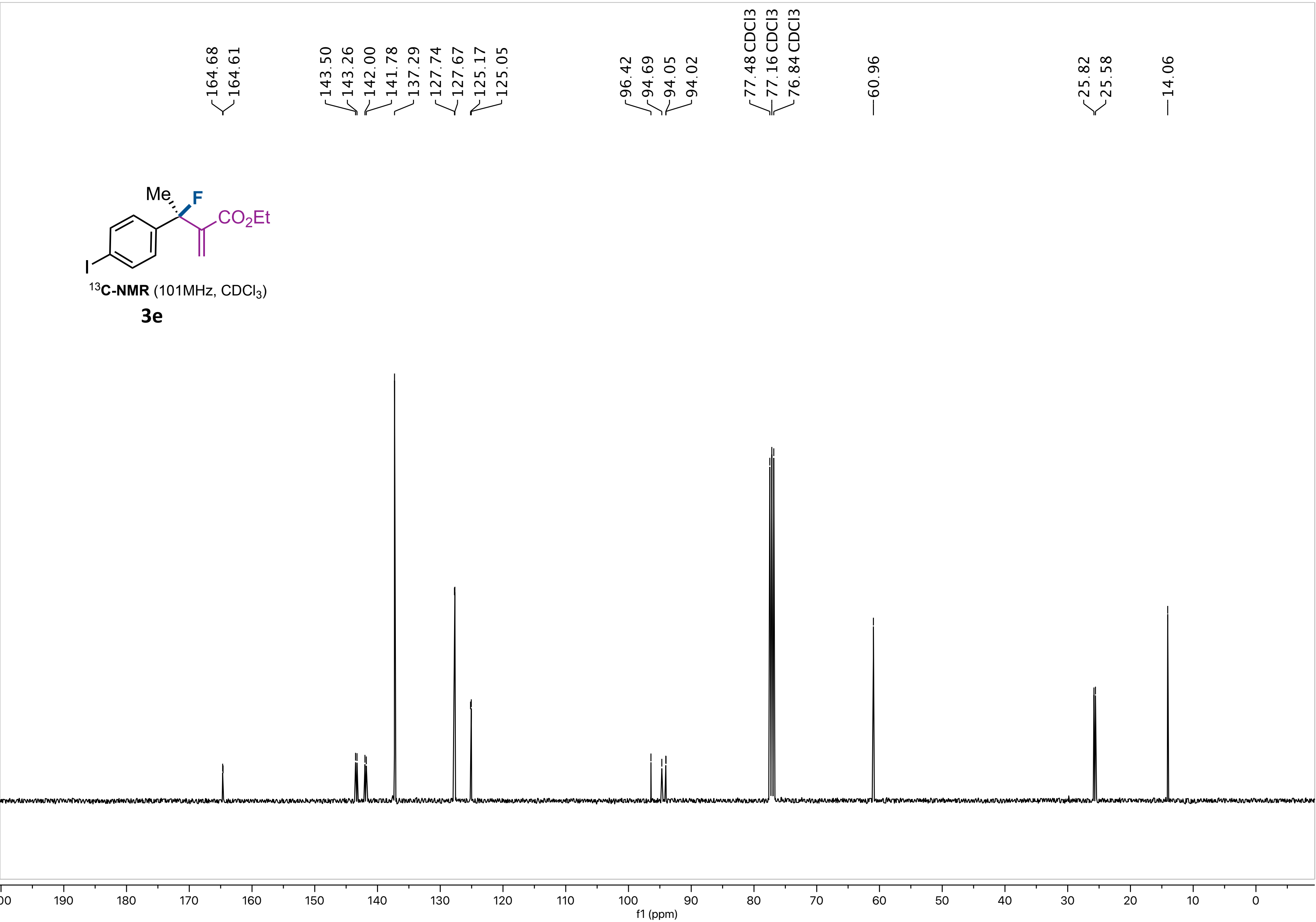

^{13}**C-NMR** (101MHz, $CDCl_3$)

3e

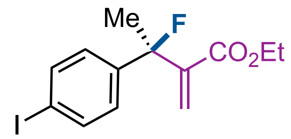

^{19}**F-NMR** (376MHz, $CDCl_3$)

3e

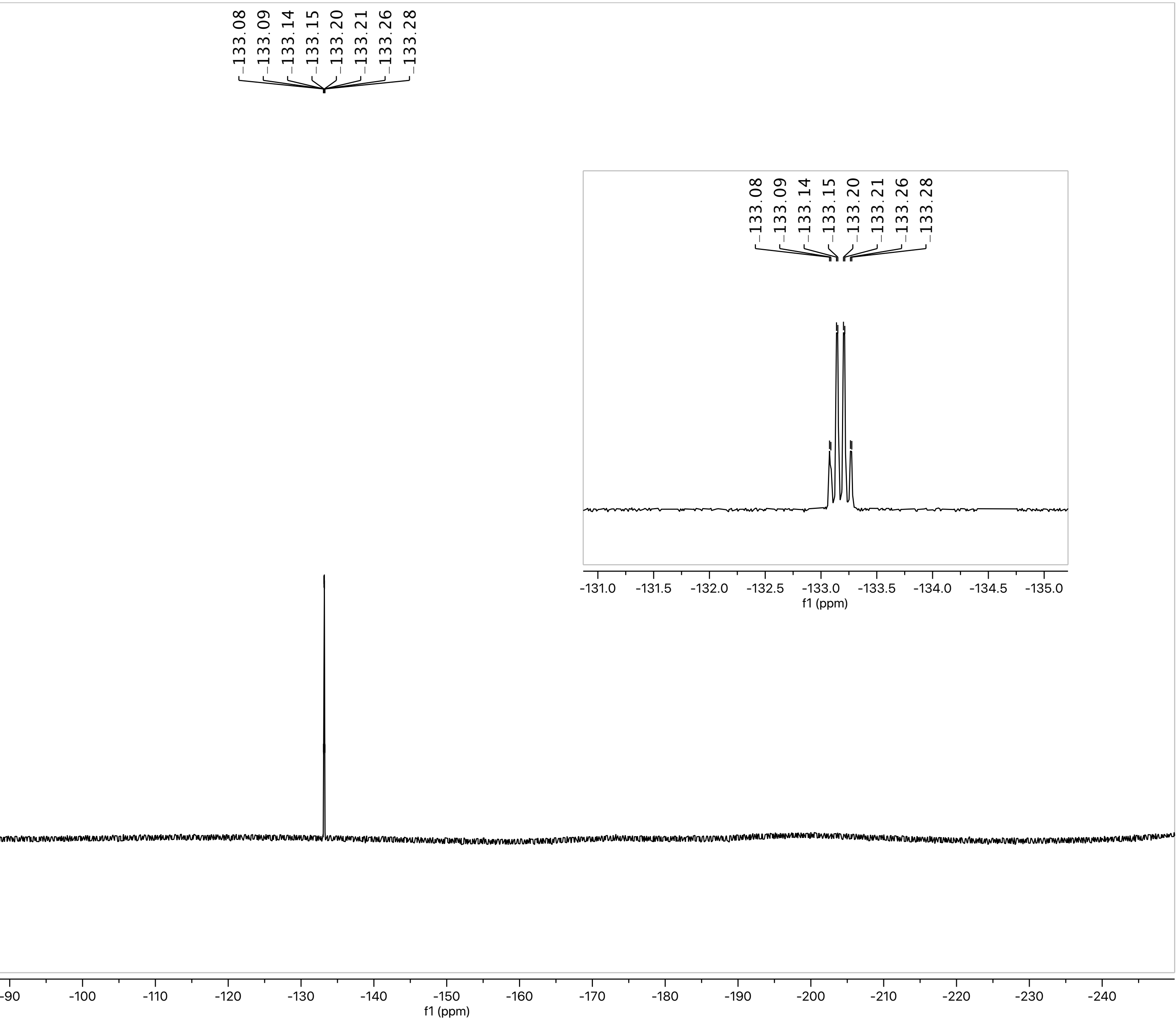

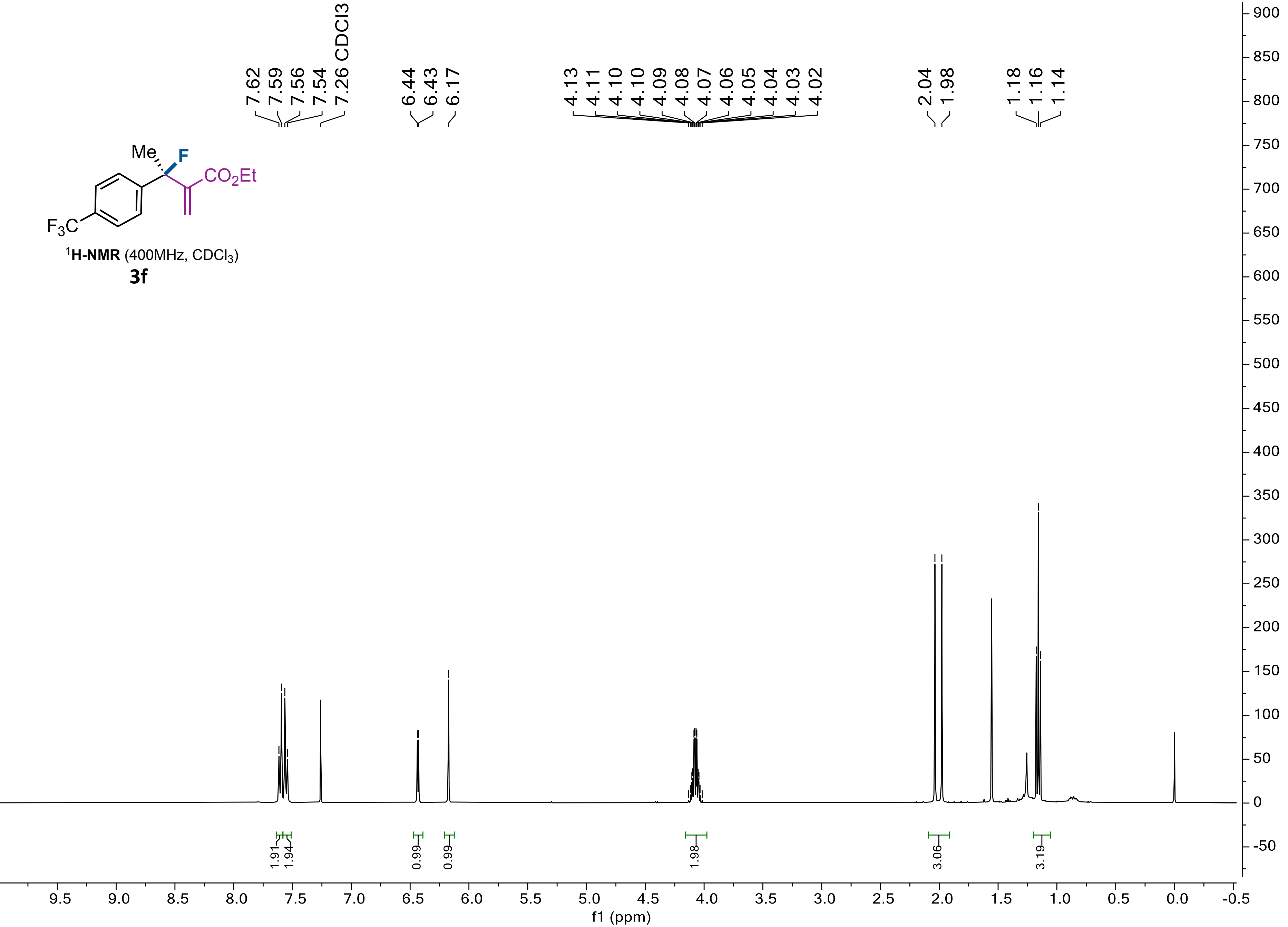

^{1}**H-NMR** (400MHz, $CDCl_3$)

3f

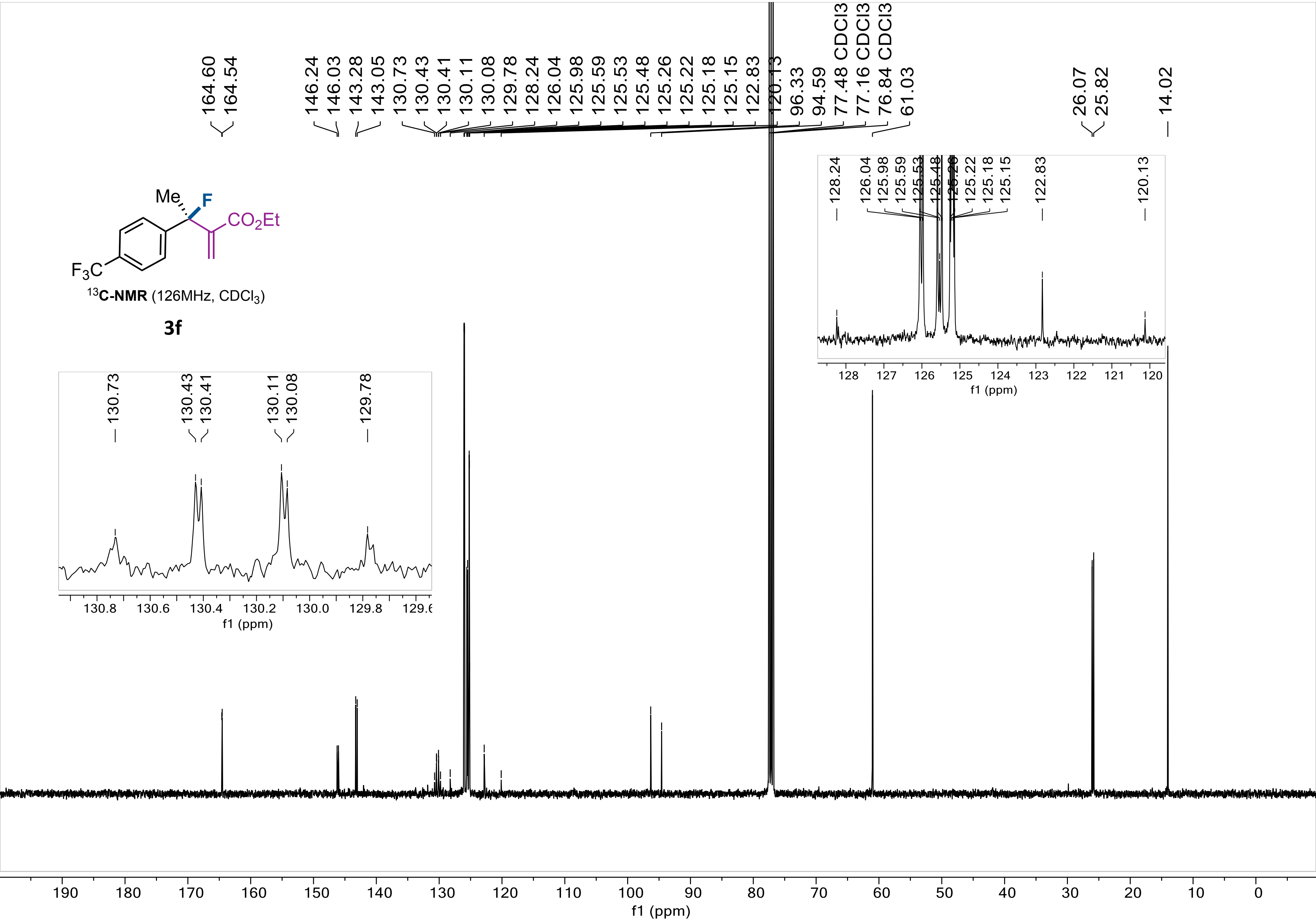
164.60
164.54
146.24
146.03
143.28
143.05
130.73
130.43
130.41
130.11
130.08
129.78
128.24
126.04
125.98
125.59
125.53
125.48
125.26
125.22
125.18
125.15
122.83
120.13
96.33
94.59
77.48 CDCl3
77.16 CDCl3
76.84 CDCl3
61.03
26.07
25.82
14.02
Me
F
CO_2Et
F_3C
^{13}C-NMR (126MHz, $CDCl_3$)
3f
f1 (ppm)

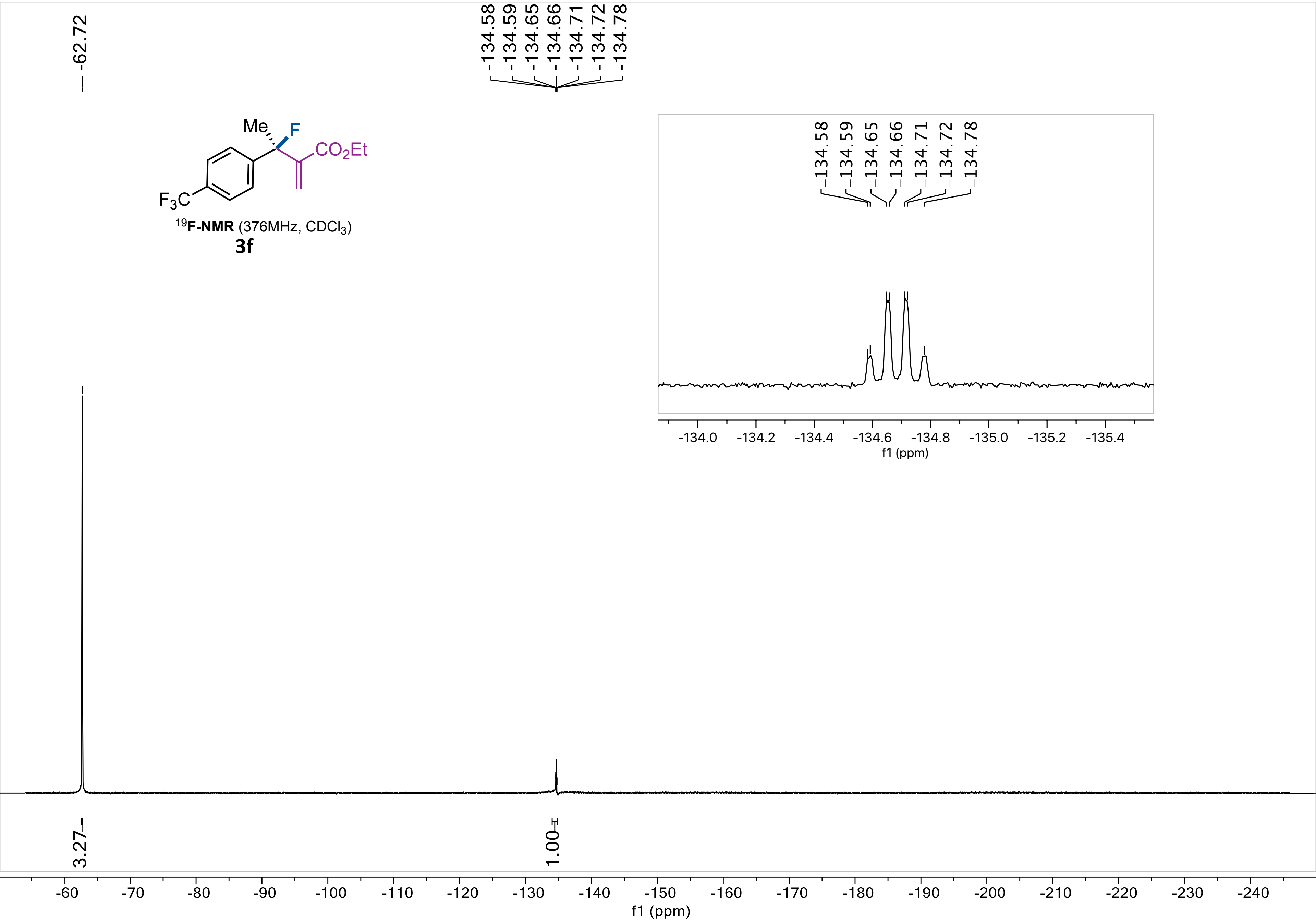
-62.72
-134.58
-134.59
-134.65
-134.66
-134.71
-134.72
-134.78
Me
F
CO_2Et
F_3C
^{19}F-NMR (376MHz, $CDCl_3$)
3f
-134.0
-134.2
-134.4
-134.6
-134.8
-135.0
-135.2
-135.4
f1 (ppm)
3.27
1.00
-60
-70
-80
-90
-100
-110
-120
-130
-140
-150
-160
-170
-180
-190
-200
-210
-220
-230
-240
f1 (ppm)

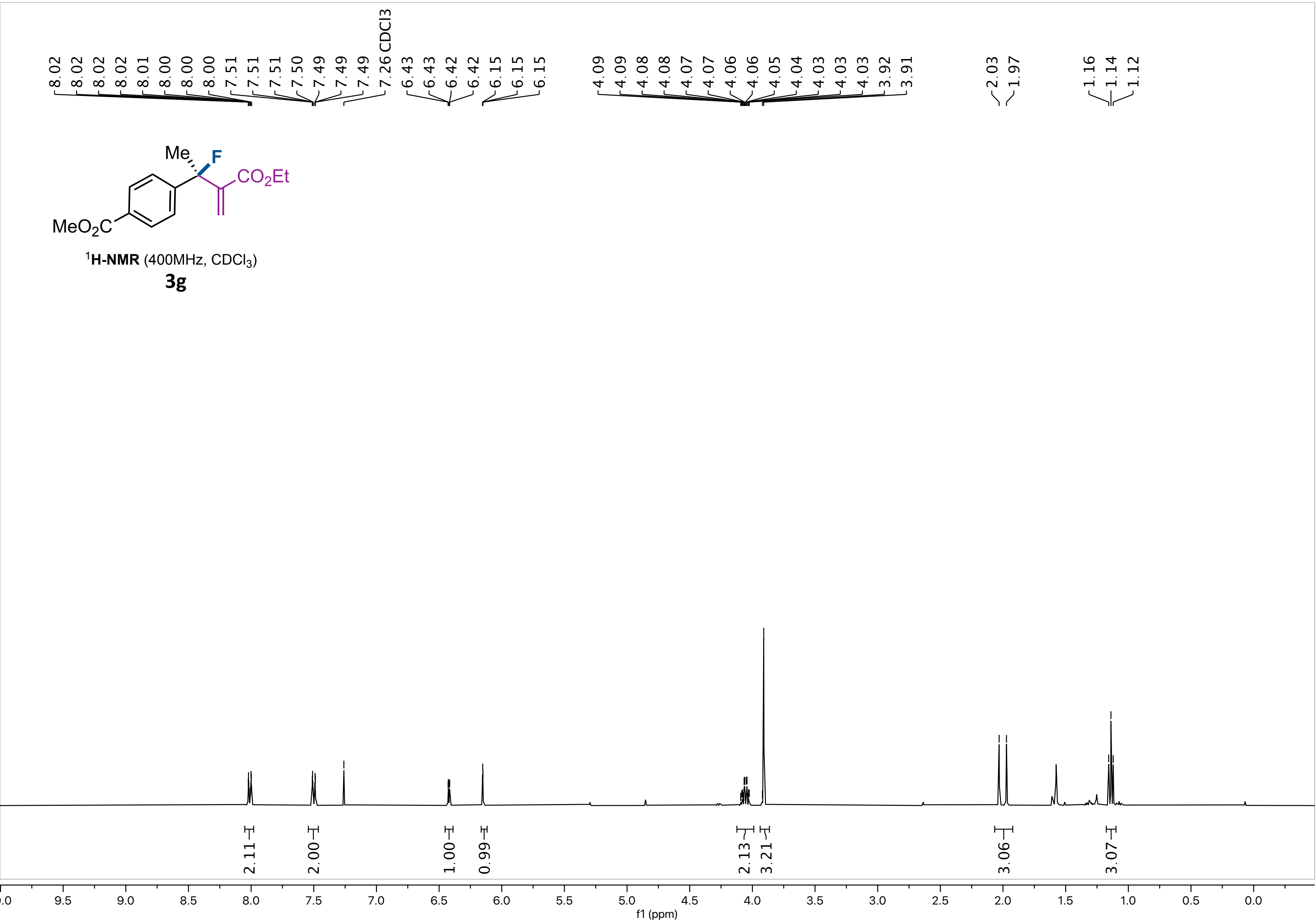
8.02 8.02 8.02 8.02 8.01 8.00 8.00 8.00 7.51 7.51 7.51 7.50 7.49 7.49 7.49 7.26 CDCl3 6.43 6.43 6.42 6.42 6.15 6.15 6.15
4.09 4.09 4.08 4.08 4.07 4.07 4.06 4.06 4.05 4.04 4.03 4.03 4.03 3.92 3.91
2.03 1.97
1.16 1.14 1.12
Me
F
CO2Et
MeO2C
1H-NMR (400MHz, CDCl3)
3g
2.11 2.00 1.00 0.99 2.13 3.21 3.06 3.07
9.5 9.0 8.5 8.0 7.5 7.0 6.5 6.0 5.5 5.0 4.5 4.0 3.5 3.0 2.5 2.0 1.5 1.0 0.5 0.0
f1 (ppm)

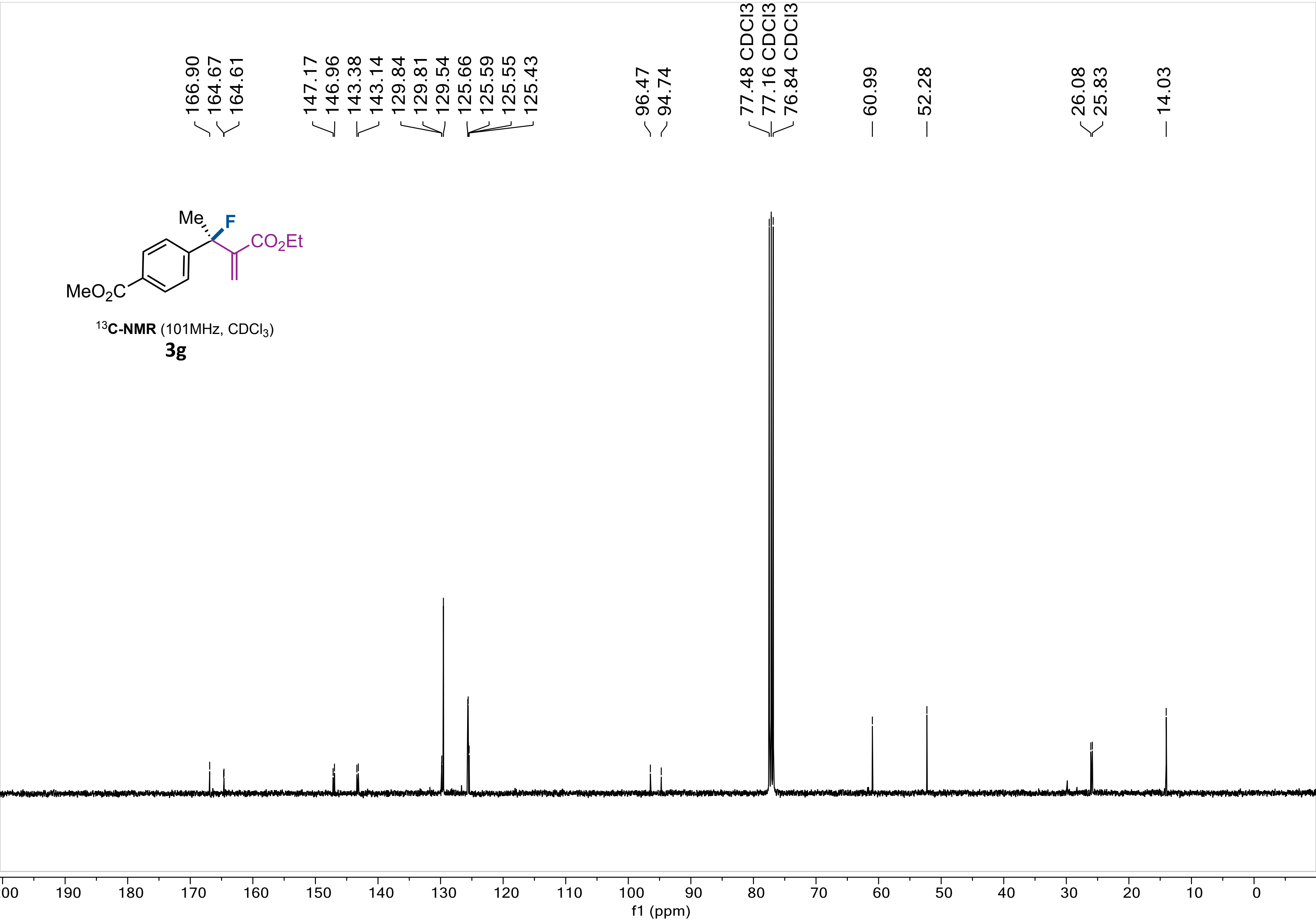
166.90
164.67
164.61
147.17
146.96
143.38
143.14
129.84
129.81
129.54
125.66
125.59
125.55
125.43
96.47
94.74
77.48 CDCl3
77.16 CDCl3
76.84 CDCl3
60.99
52.28
26.08
25.83
14.03
Me
F
CO_2Et
MeO_2C
^{13}C-NMR (101MHz, $CDCl_3$)
3g
00
190
180
170
160
150
140
130
120
110
100
90
80
70
60
50
40
30
20
10
0
f1 (ppm)

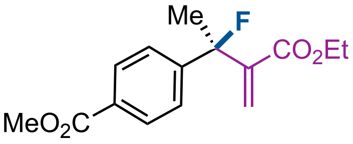

^{19}**F-NMR** (376MHz, $CDCl_3$)

3g

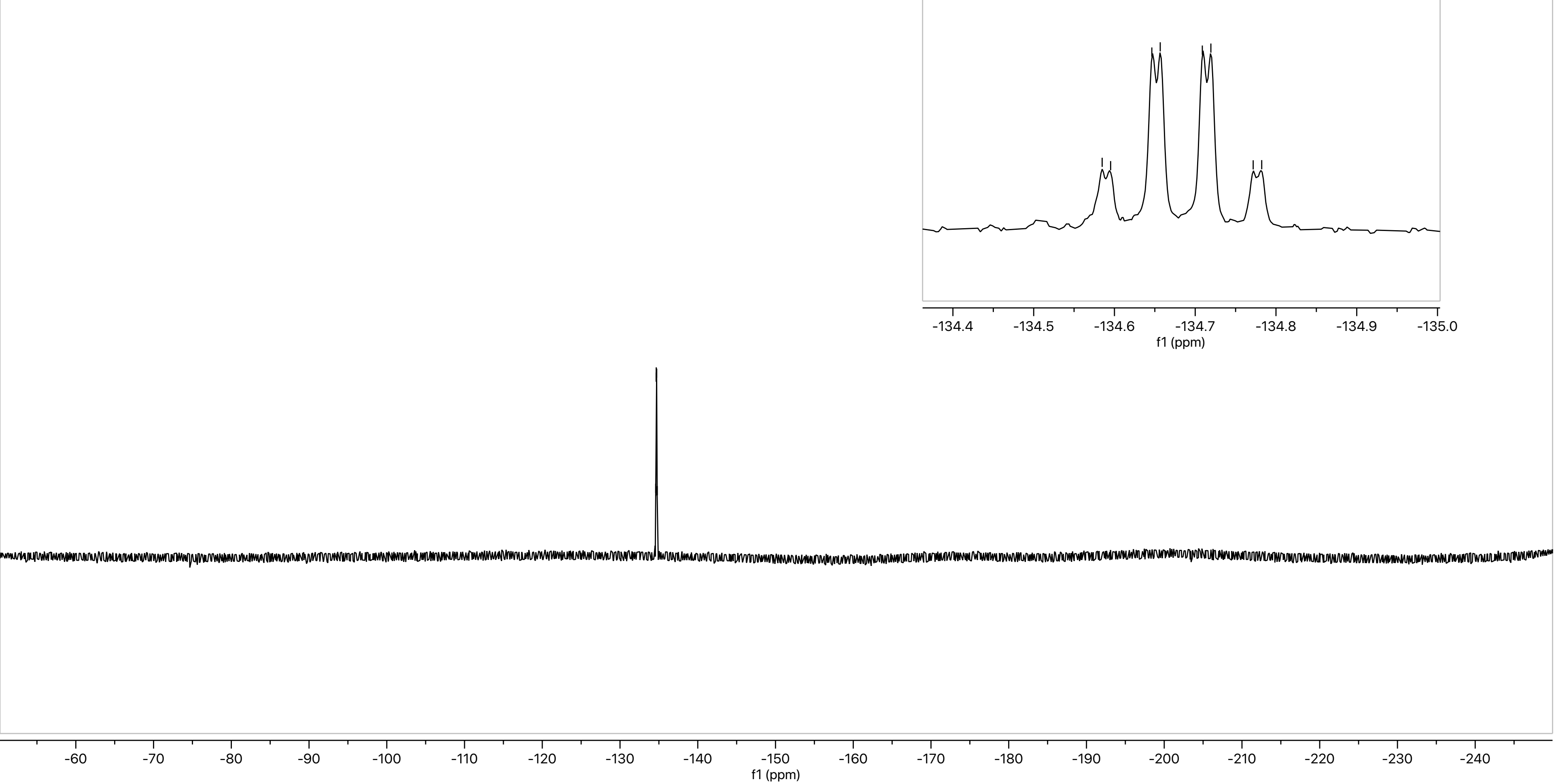

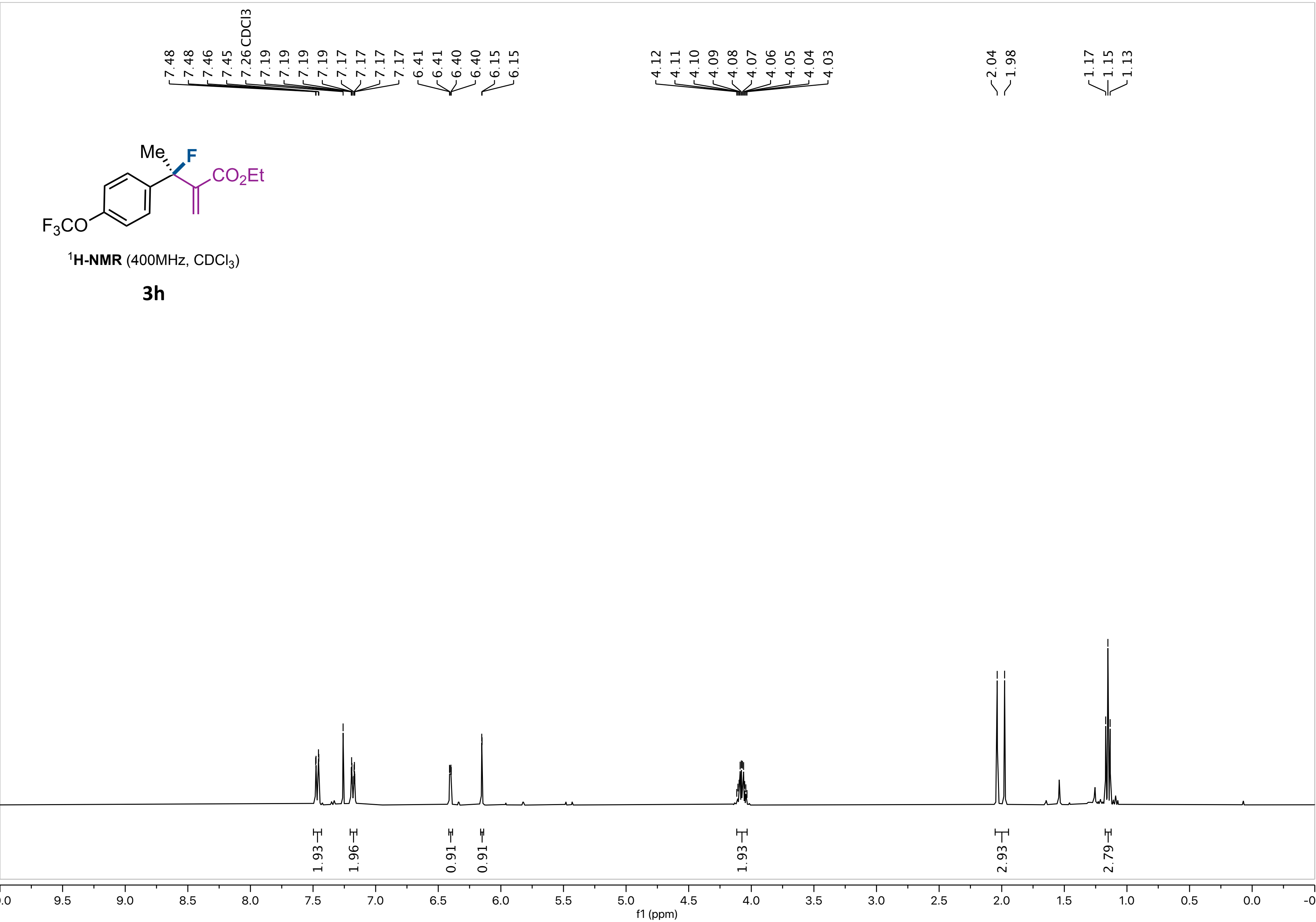
7.48
7.48
7.46
7.45
7.26 CDCl3
7.19
7.19
7.19
7.19
7.17
7.17
7.17
7.17
6.41
6.41
6.40
6.40
6.15
6.15
4.12
4.11
4.10
4.09
4.08
4.07
4.06
4.05
4.04
4.03
2.04
1.98
1.17
1.15
1.13
Me
F
CO2Et
F3CO
1H-NMR (400MHz, CDCl3)
3h
1.93
1.96
0.91
0.91
1.93
2.93
2.79
9.5
9.0
8.5
8.0
7.5
7.0
6.5
6.0
5.5
5.0
4.5
4.0
3.5
3.0
2.5
2.0
1.5
1.0
0.5
0.0
f1 (ppm)

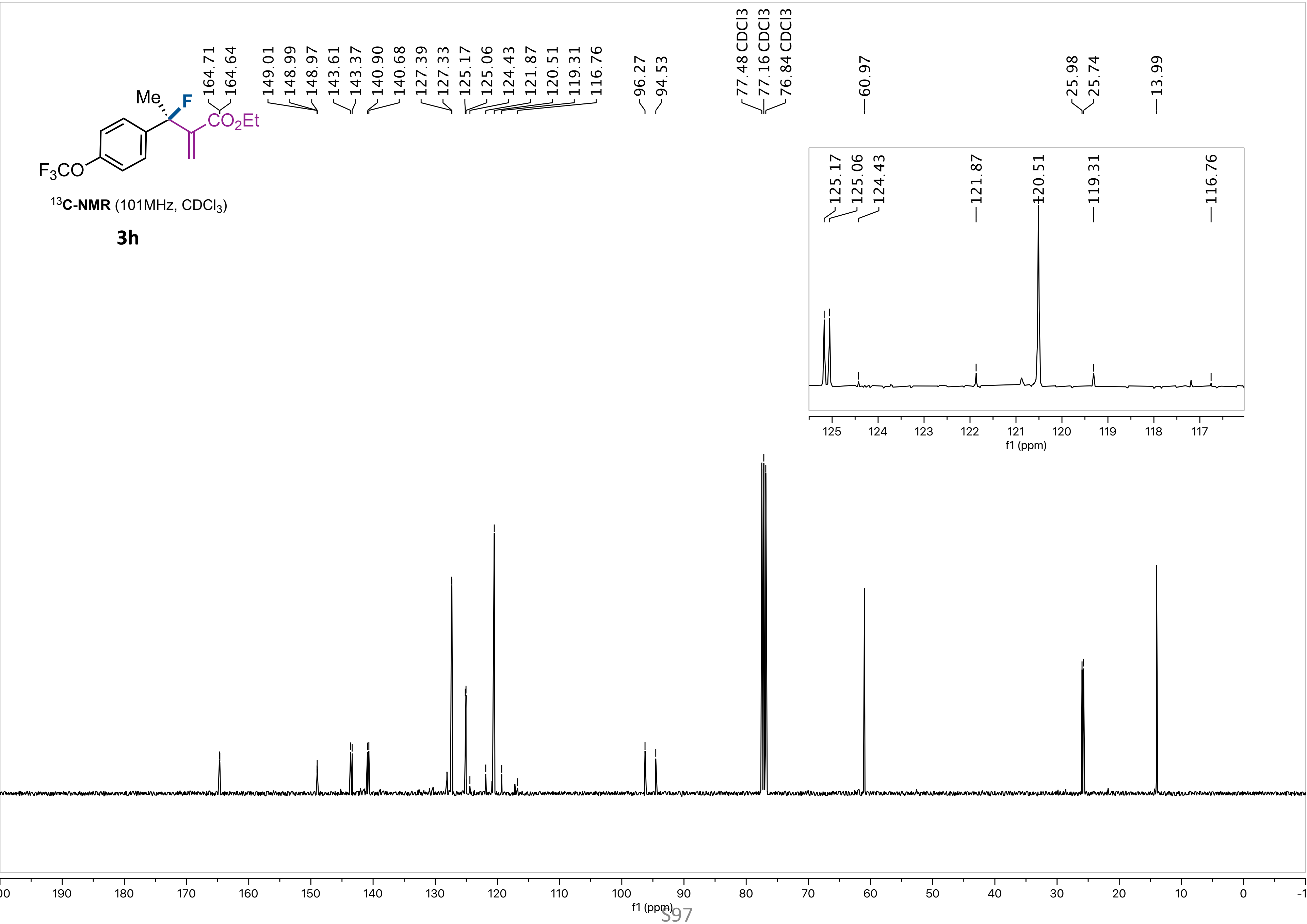
Me
F
CO2Et
F3CO
13C-NMR (101MHz, CDCl3)
3h
164.71
164.64
149.01
148.99
148.97
143.61
143.37
140.90
140.68
127.39
127.33
125.17
125.06
124.43
121.87
120.51
119.31
116.76
96.27
94.53
77.48 CDCl3
77.16 CDCl3
76.84 CDCl3
60.97
25.98
25.74
13.99
f1 (ppm)

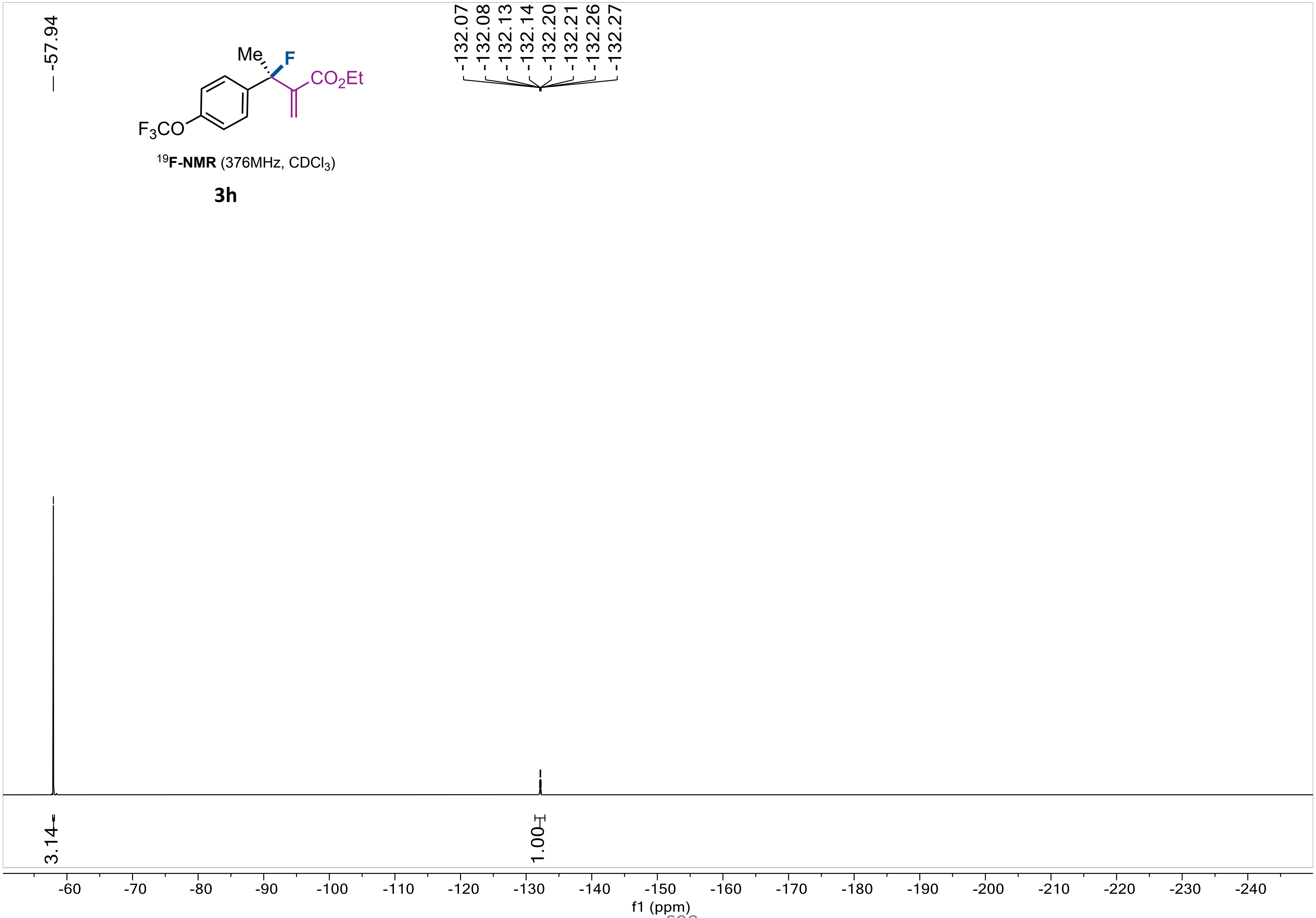
-57.94
-132.07
-132.08
-132.13
-132.14
-132.20
-132.21
-132.26
-132.27
Me
F
CO_2Et
F_3CO
^{19}F-NMR (376MHz, $CDCl_3$)
3h
3.14
1.00
-60
-70
-80
-90
-100
-110
-120
-130
-140
-150
-160
-170
-180
-190
-200
-210
-220
-230
-240
f1 (ppm)

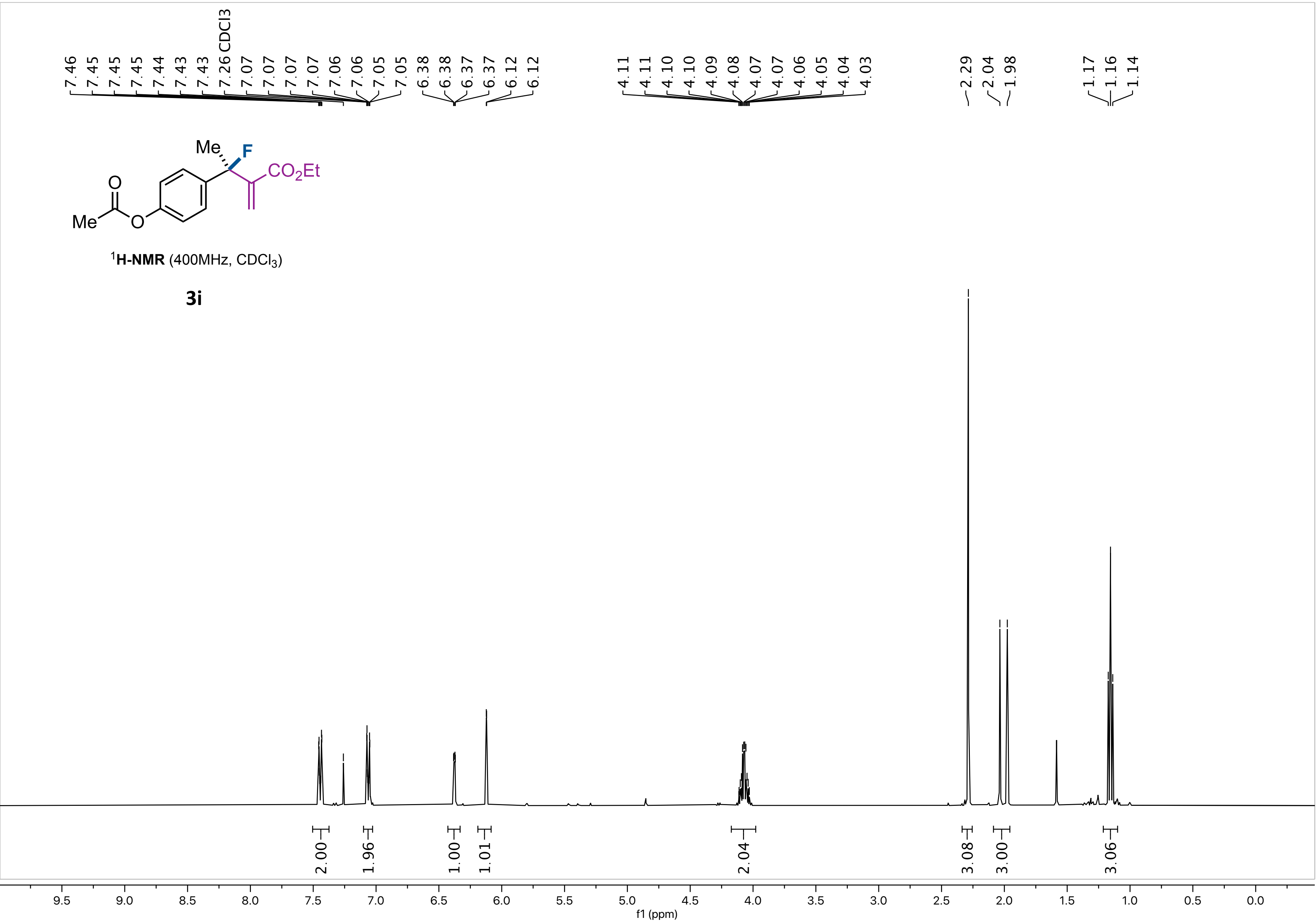
7.46
7.45
7.45
7.45
7.44
7.43
7.43
7.26 CDCl3
7.07
7.07
7.07
7.07
7.06
7.06
7.05
7.05
6.38
6.38
6.37
6.37
6.12
6.12
4.11
4.11
4.10
4.10
4.09
4.08
4.07
4.07
4.06
4.05
4.04
4.03
2.29
2.04
1.98
1.17
1.16
1.14
Me
F
CO_2Et
O
Me
O
^{1}H-NMR (400MHz, $CDCl_3$)
3i
2.00
1.96
1.00
1.01
2.04
3.08
3.00
3.06
9.5
9.0
8.5
8.0
7.5
7.0
6.5
6.0
5.5
5.0
4.5
4.0
3.5
3.0
2.5
2.0
1.5
1.0
0.5
0.0
f1 (ppm)

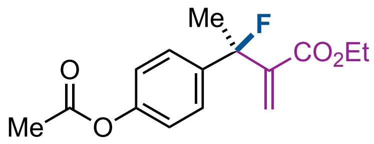

^{13}**C-NMR** (101MHz, $CDCl_3$)

3i

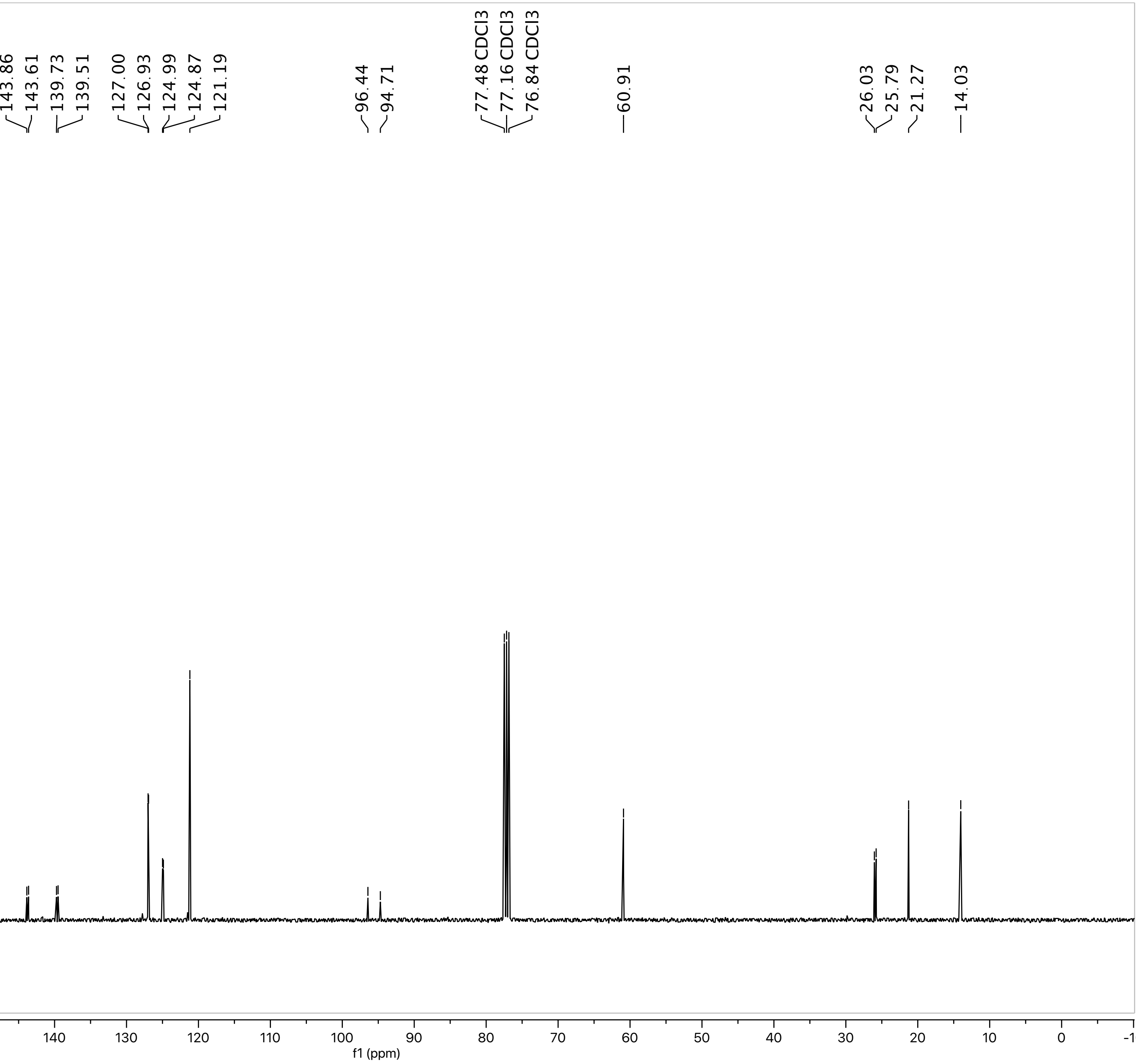

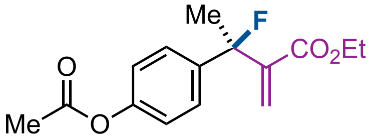

^{19}**F-NMR** (376MHz, $CDCl_3$)

3i

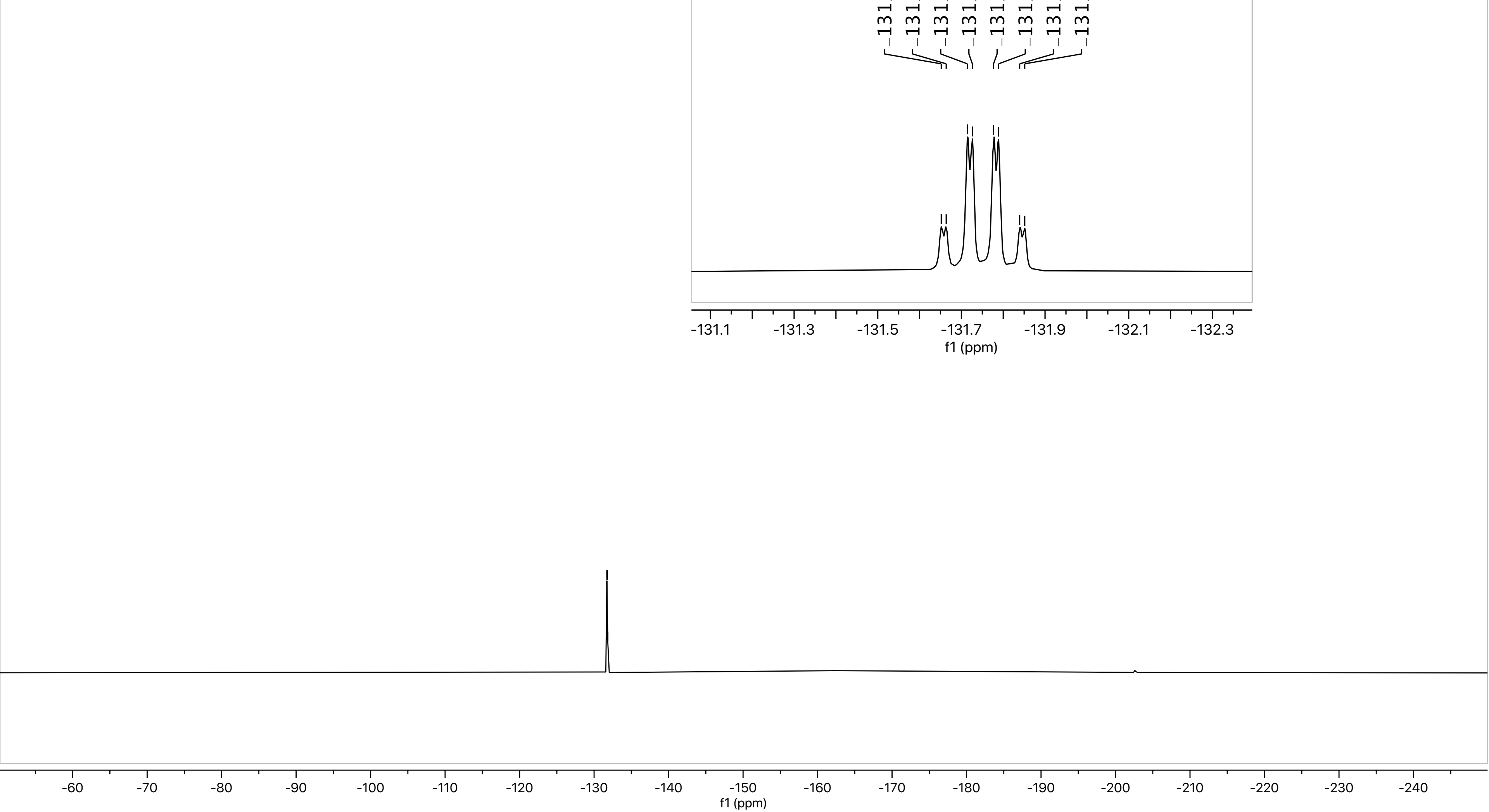

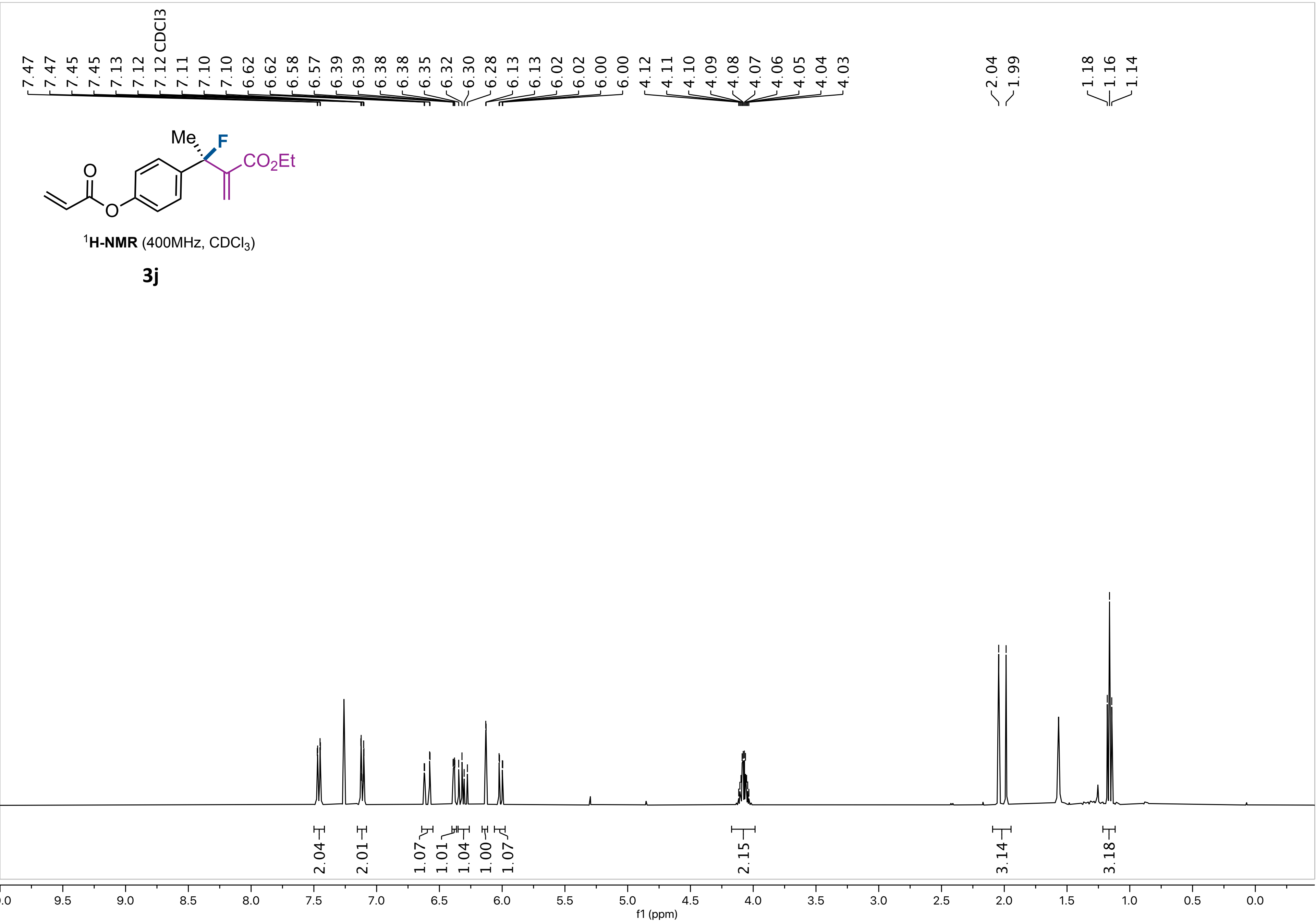

7.47 7.47 7.45 7.45 7.13 7.12 7.12 CDCl3 7.11 7.10 7.10 6.62 6.62 6.58 6.57 6.39 6.39 6.38 6.38 6.35 6.32 6.30 6.28 6.13 6.13 6.02 6.02 6.00 6.00 4.12 4.11 4.10 4.09 4.08 4.07 4.06 4.05 4.04 4.03 2.04 1.99 1.18 1.16 1.14
Me
F
CO_2Et
O
O
^{1}H-NMR (400MHz, $CDCl_3$)
3j
2.04 2.01 1.07 1.01 1.04 1.00 1.07 2.15 3.14 3.18
f1 (ppm)

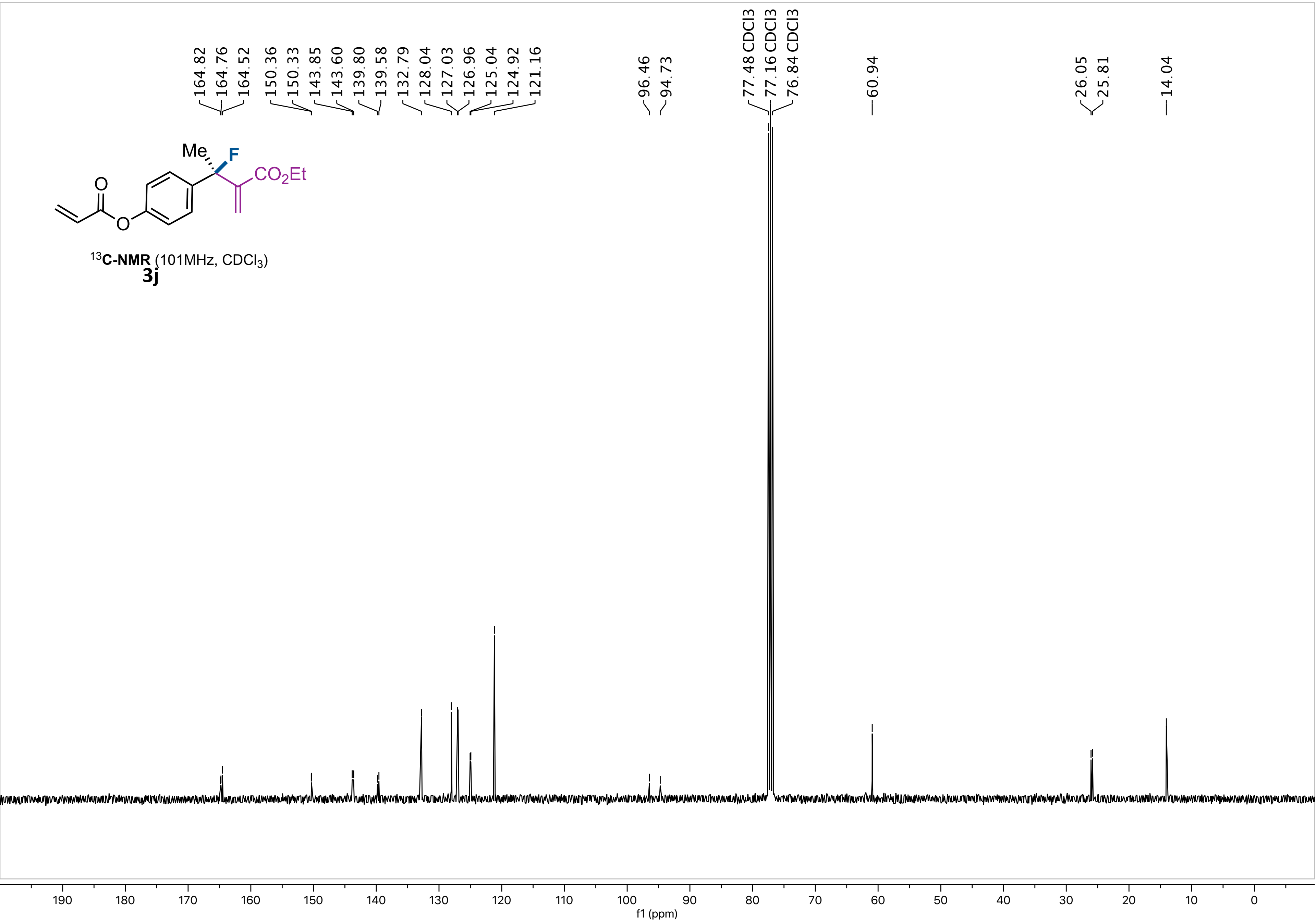
164.82
164.76
164.52
150.36
150.33
143.85
143.60
139.80
139.58
132.79
128.04
127.03
126.96
125.04
124.92
121.16
96.46
94.73
77.48 CDCl3
77.16 CDCl3
76.84 CDCl3
60.94
26.05
25.81
14.04
Me
F
CO2Et
O
O
13C-NMR (101MHz, CDCl3)
3j
190 180 170 160 150 140 130 120 110 100 90 80 70 60 50 40 30 20 10 0
f1 (ppm)

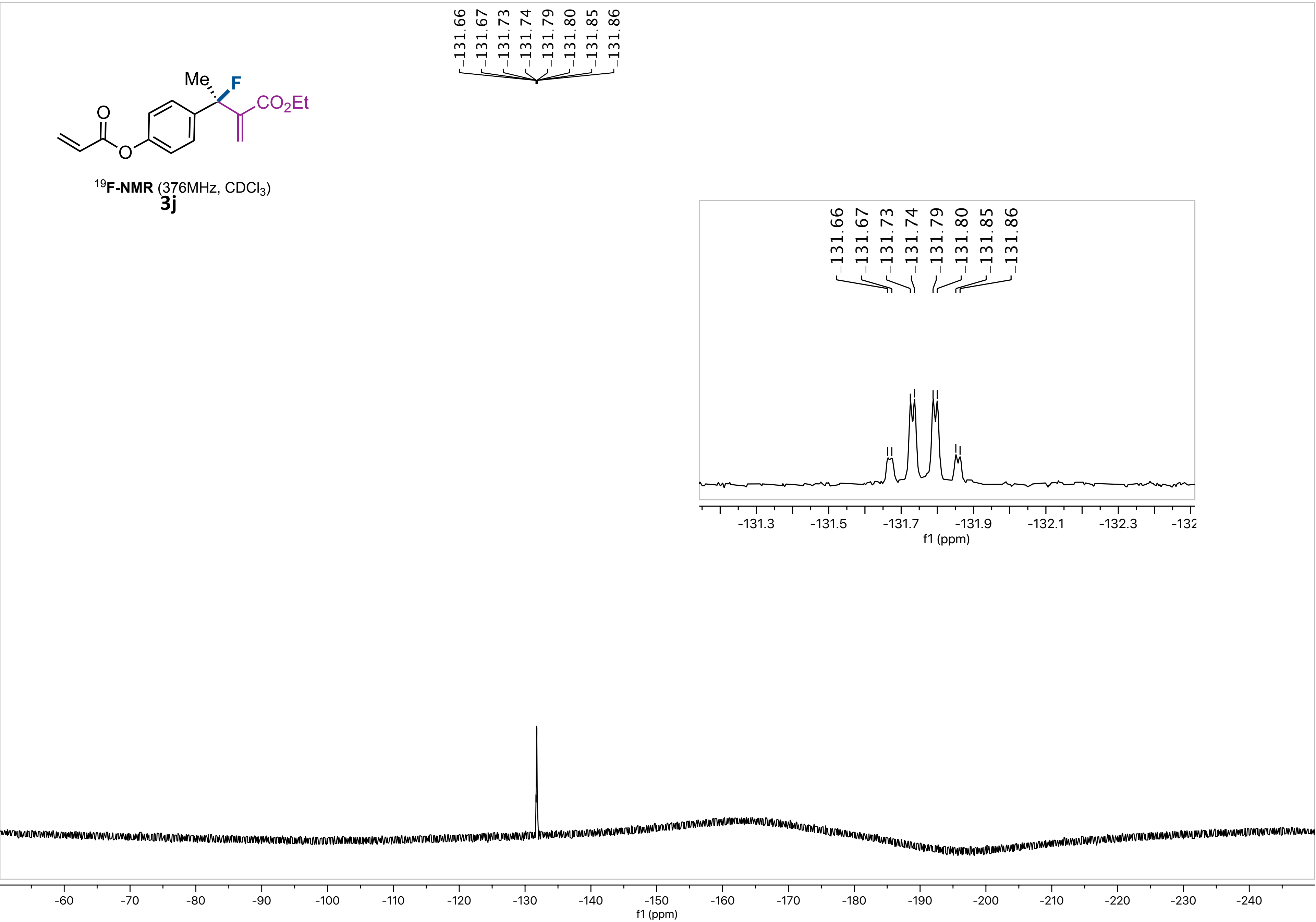

Me
F
CO_2Et
O
O
^{19}F-NMR (376MHz, $CDCl_3$)
3j
-131.66
-131.67
-131.73
-131.74
-131.79
-131.80
-131.85
-131.86
-131.3
-131.5
-131.7
-131.9
-132.1
-132.3
f1 (ppm)
-60
-70
-80
-90
-100
-110
-120
-130
-140
-150
-160
-170
-180
-190
-200
-210
-220
-230
-240
f1 (ppm)

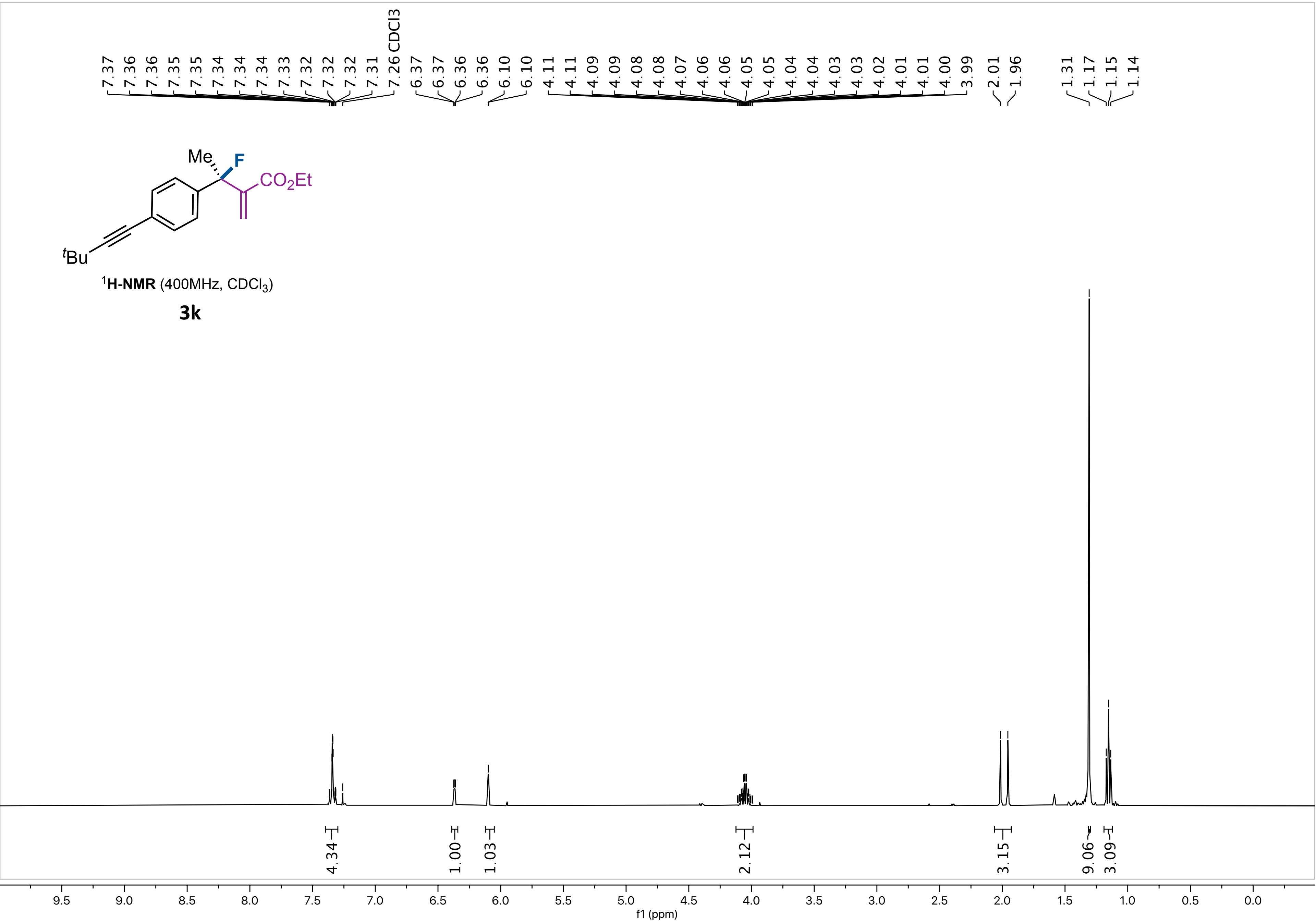
7.37 7.36 7.36 7.35 7.35 7.34 7.34 7.34 7.33 7.32 7.32 7.32 7.31 7.26 CDCl3 6.37 6.37 6.36 6.36 6.10 6.10 4.11 4.11 4.09 4.09 4.08 4.08 4.07 4.06 4.06 4.05 4.05 4.04 4.04 4.03 4.03 4.02 4.01 4.01 4.00 3.99 2.01 1.96 1.31 1.17 1.15 1.14
Me
F
CO_2Et
^{t}Bu
^{1}H-NMR (400MHz, $CDCl_3$)
3k
4.34
1.00
1.03
2.12
3.15
9.06
3.09
9.5 9.0 8.5 8.0 7.5 7.0 6.5 6.0 5.5 5.0 4.5 4.0 3.5 3.0 2.5 2.0 1.5 1.0 0.5 0.0
f1 (ppm)

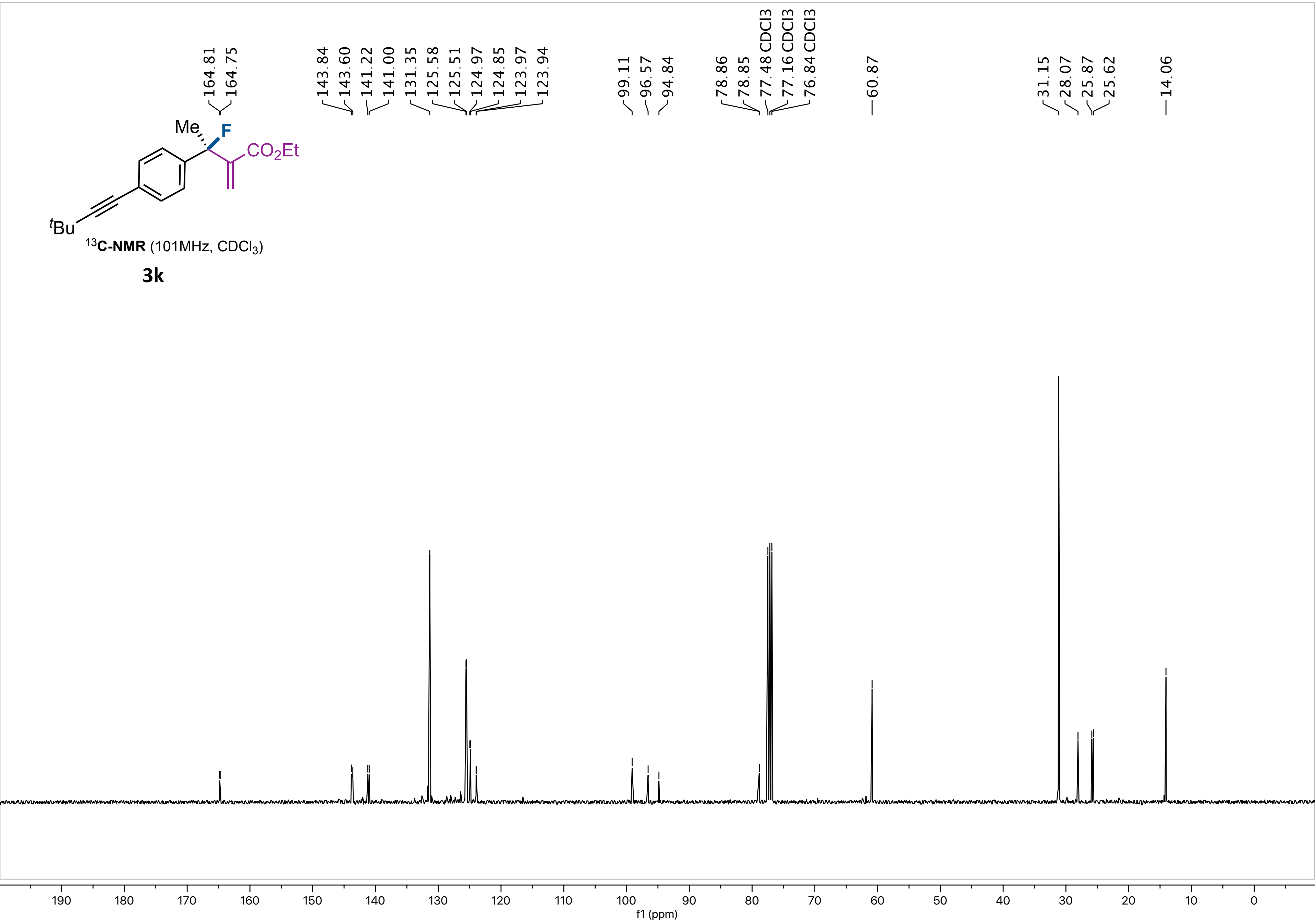

^{13}**C-NMR** (101MHz, $CDCl_3$)

3k

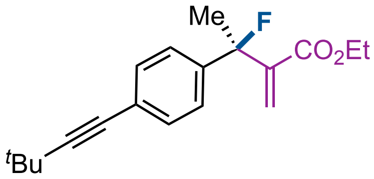

^{19}**F-NMR** (376MHz, $CDCl_3$)

3k

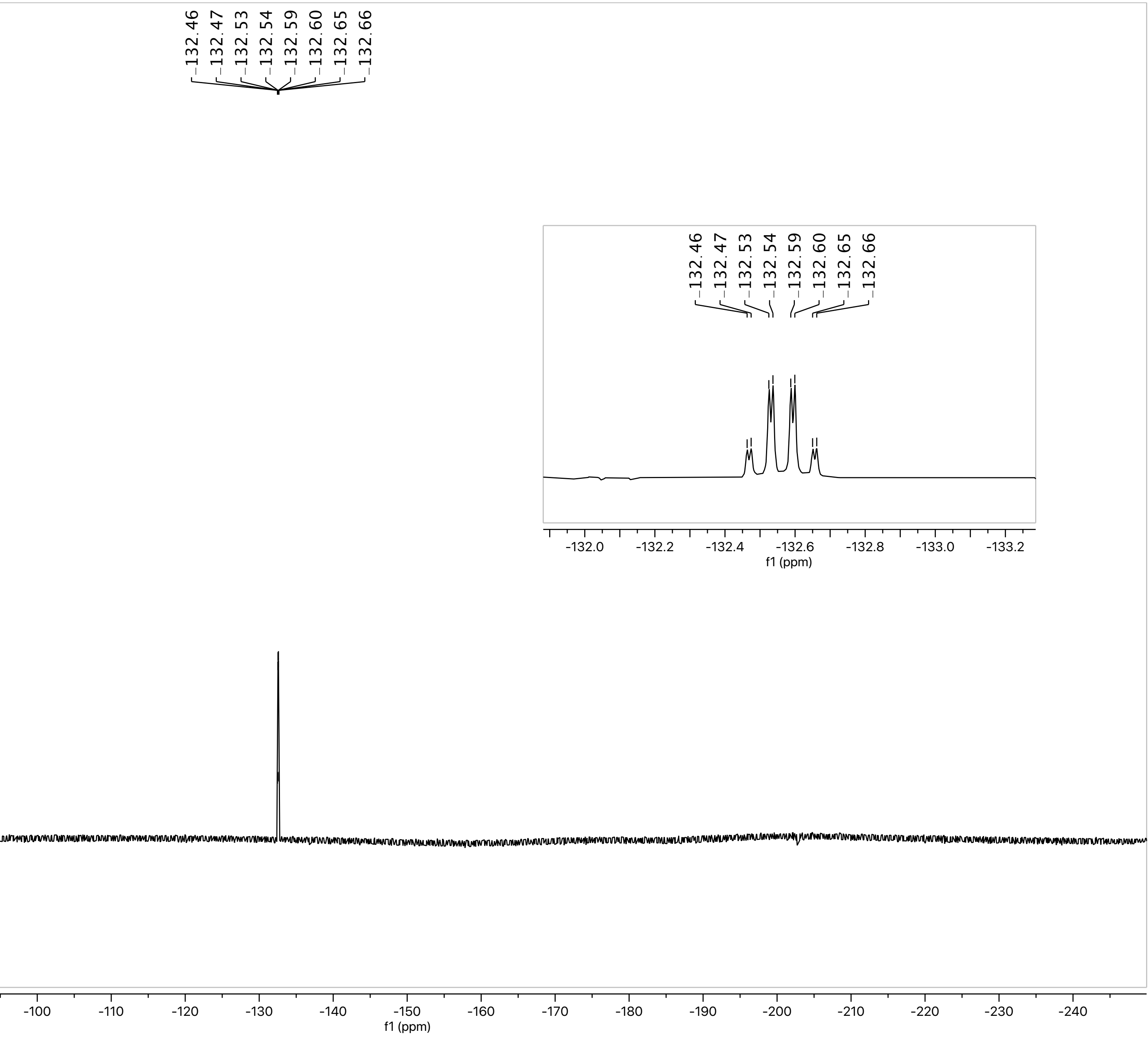

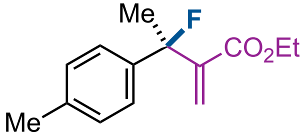

^{1}**H-NMR** (400MHz, $CDCl_3$)

3l

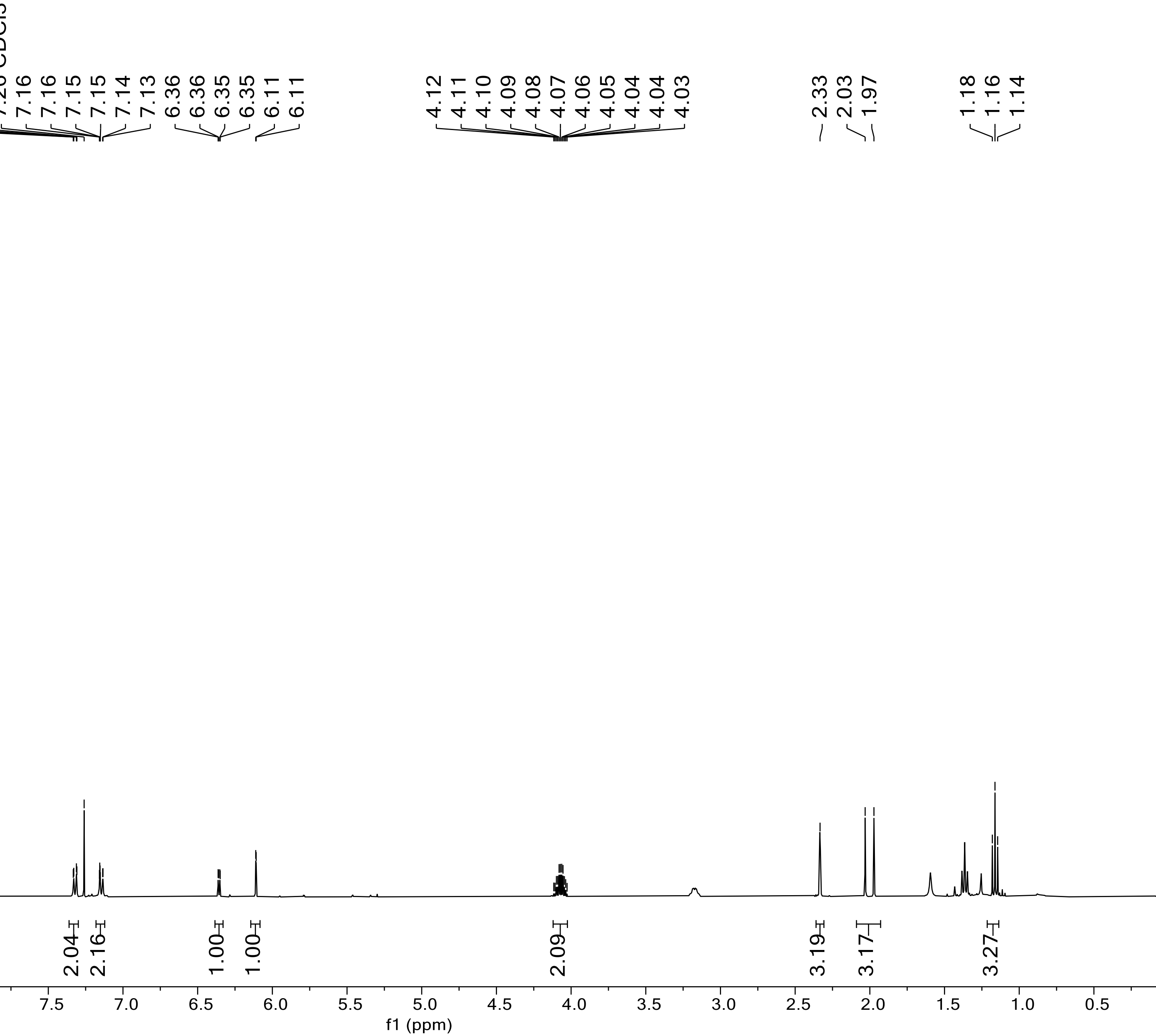

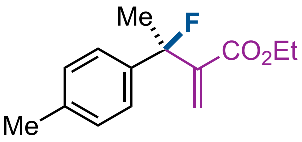

^{13}**C-NMR** (101MHz, $CDCl_3$)

3l

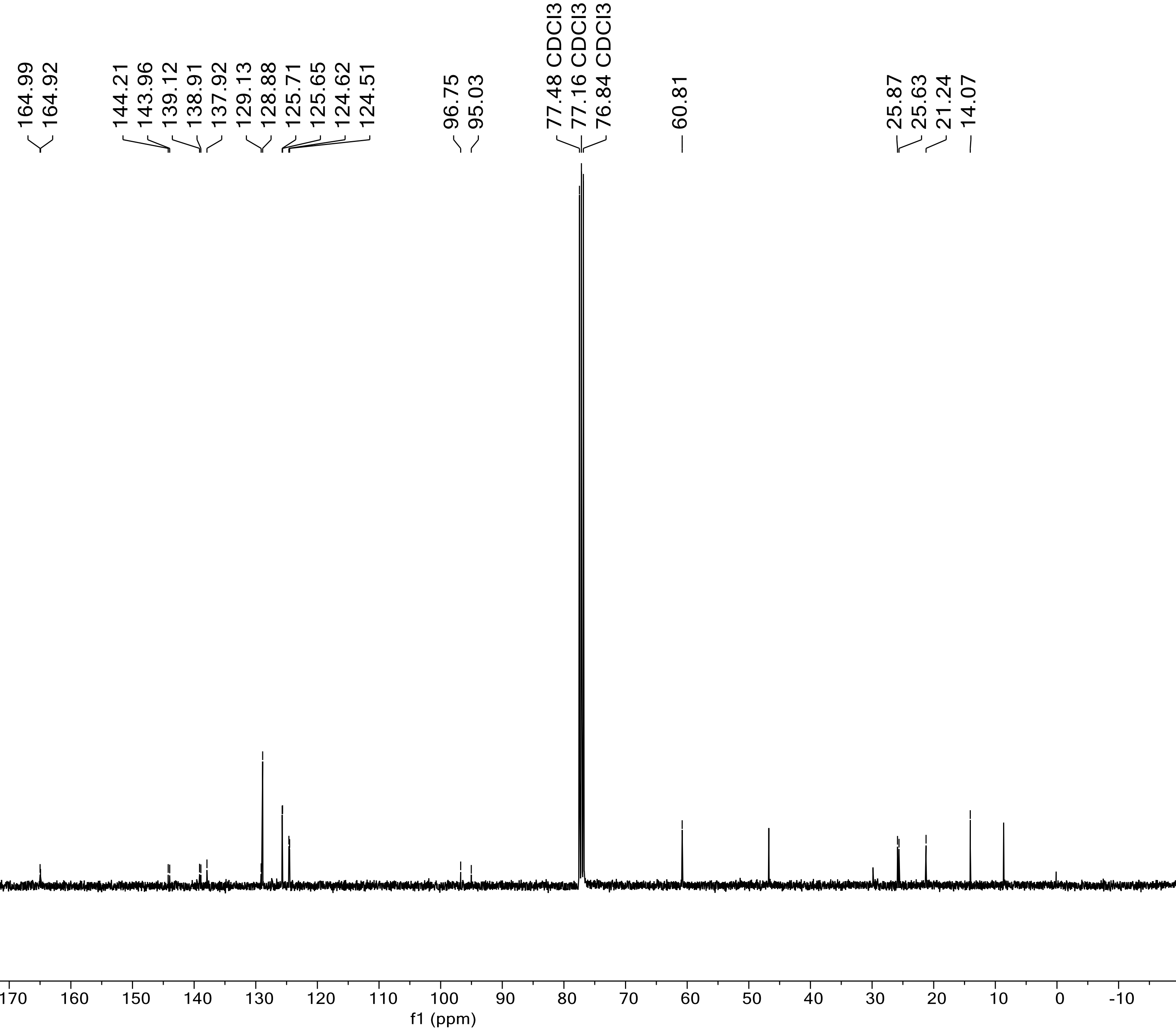

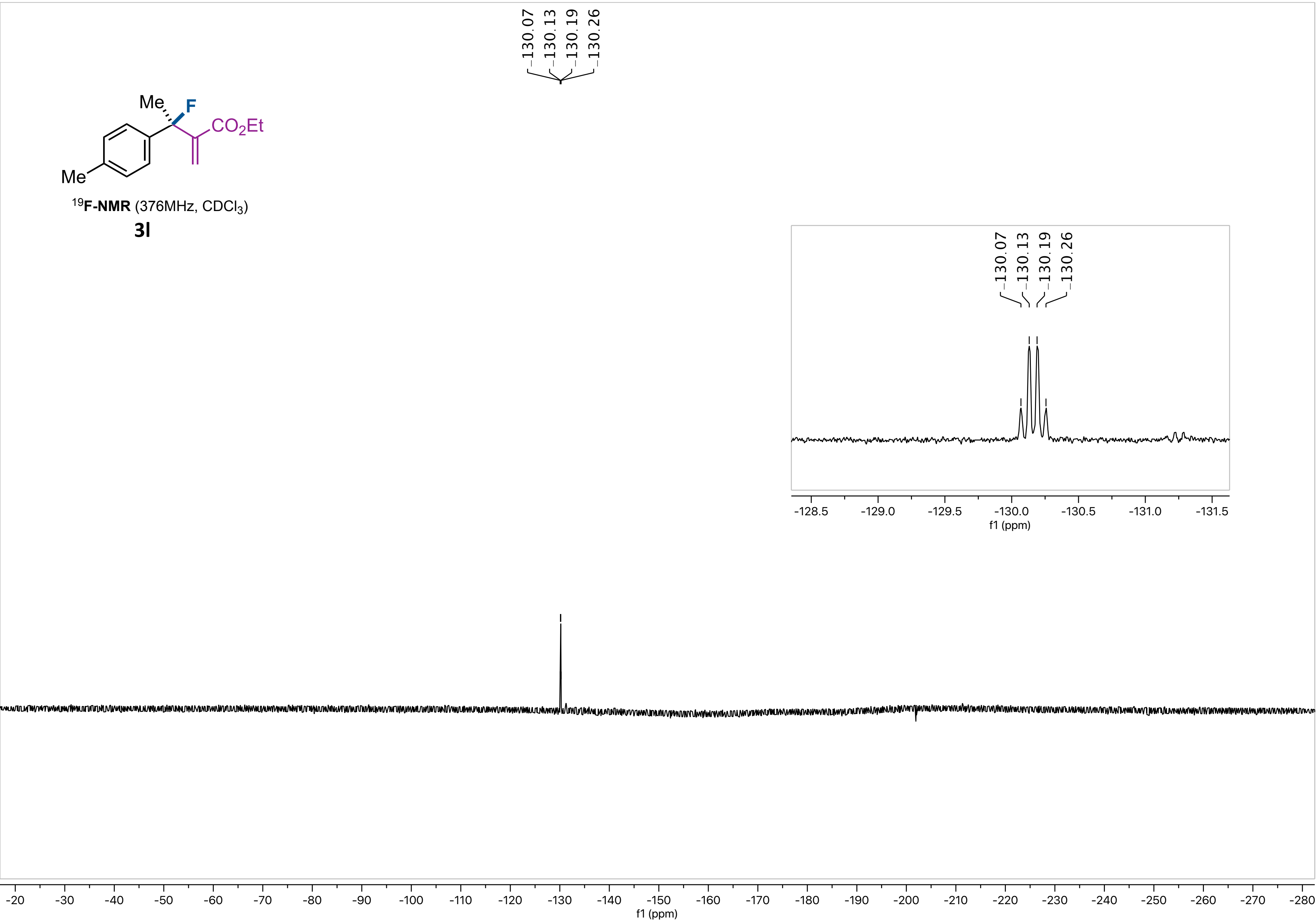
-130.07
-130.13
-130.19
-130.26
Me
F
CO2Et
Me
19F-NMR (376MHz, CDCl3)
3l
-128.5
-129.0
-129.5
-130.0
-130.5
-131.0
-131.5
f1 (ppm)
-20
-30
-40
-50
-60
-70
-80
-90
-100
-110
-120
-130
-140
-150
-160
-170
-180
-190
-200
-210
-220
-230
-240
-250
-260
-270
-280
f1 (ppm)

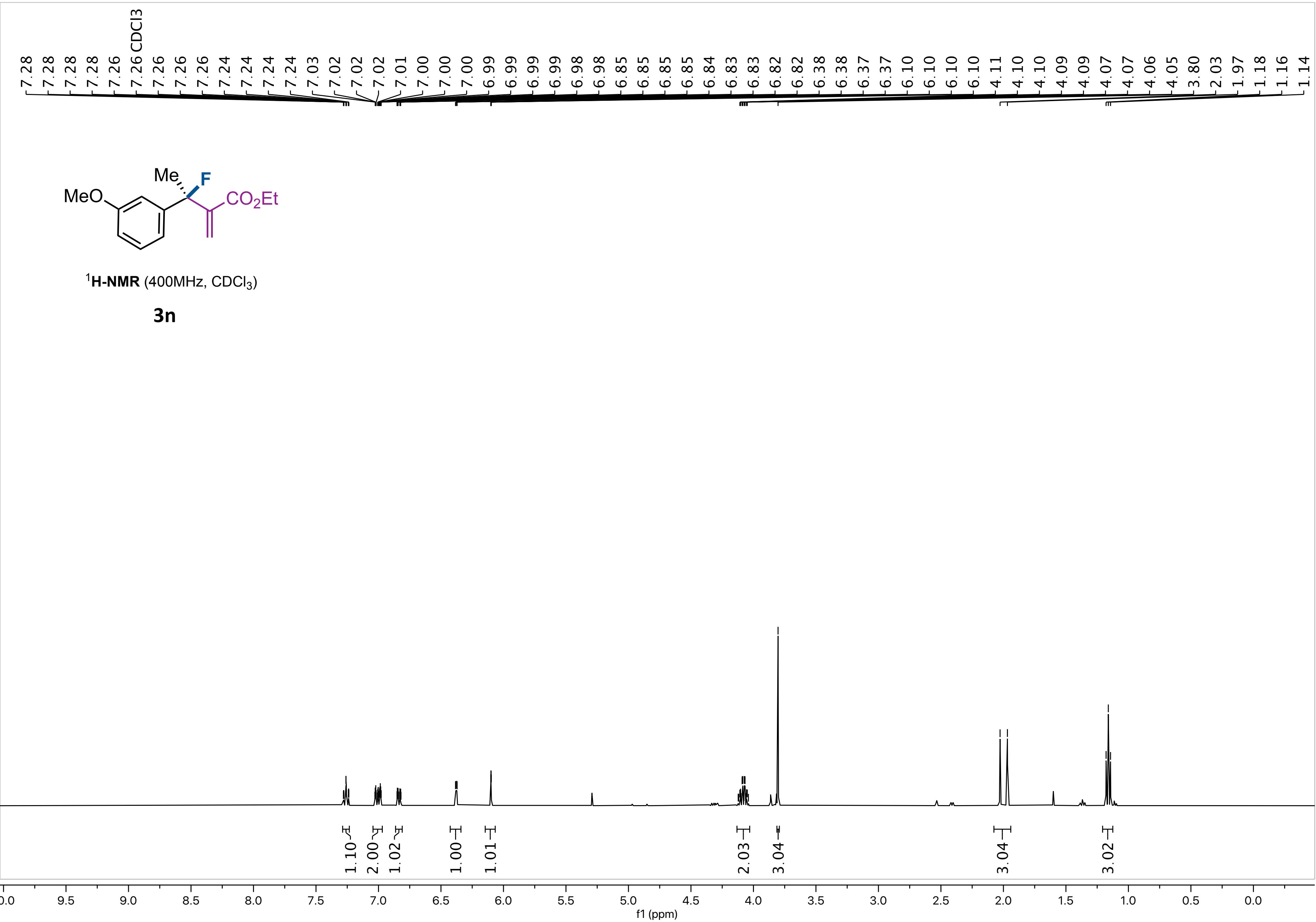
7.28 7.28 7.28 7.28 7.26 7.26 CDCl3 7.26 7.26 7.26 7.24 7.24 7.24 7.24 7.03 7.02 7.02 7.02 7.01 7.00 7.00 7.00 6.99 6.99 6.99 6.99 6.98 6.98 6.85 6.85 6.85 6.85 6.84 6.83 6.83 6.82 6.82 6.38 6.38 6.37 6.37 6.10 6.10 6.10 6.10 4.11 4.10 4.10 4.09 4.09 4.07 4.07 4.06 4.05 3.80 2.03 1.97 1.18 1.16 1.14
Me
F
MeO
CO2Et
1H-NMR (400MHz, CDCl3)
3n
1.10 2.00 1.02 1.00 1.01 2.03 3.04 3.04 3.02
10.0 9.5 9.0 8.5 8.0 7.5 7.0 6.5 6.0 5.5 5.0 4.5 4.0 3.5 3.0 2.5 2.0 1.5 1.0 0.5 0.0
f1 (ppm)

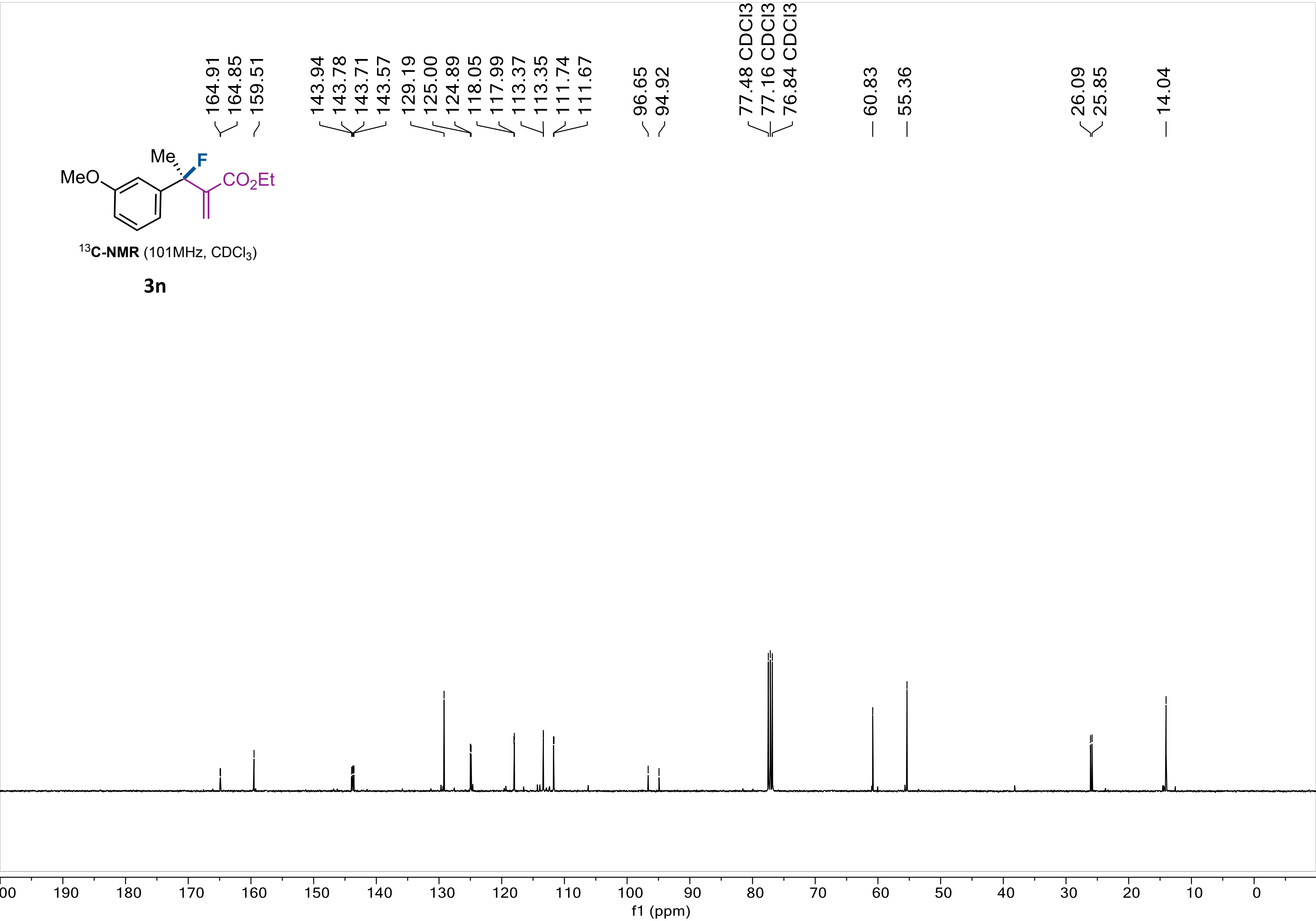
164.91
164.85
159.51
143.94
143.78
143.71
143.57
129.19
125.00
124.89
118.05
117.99
113.37
113.35
111.74
111.67
96.65
94.92
77.48 CDCl3
77.16 CDCl3
76.84 CDCl3
60.83
55.36
26.09
25.85
14.04
Me
F
MeO
CO_2Et
^{13}C-NMR (101MHz, $CDCl_3$)
3n
00 190 180 170 160 150 140 130 120 110 100 90 80 70 60 50 40 30 20 10 0
f1 (ppm)

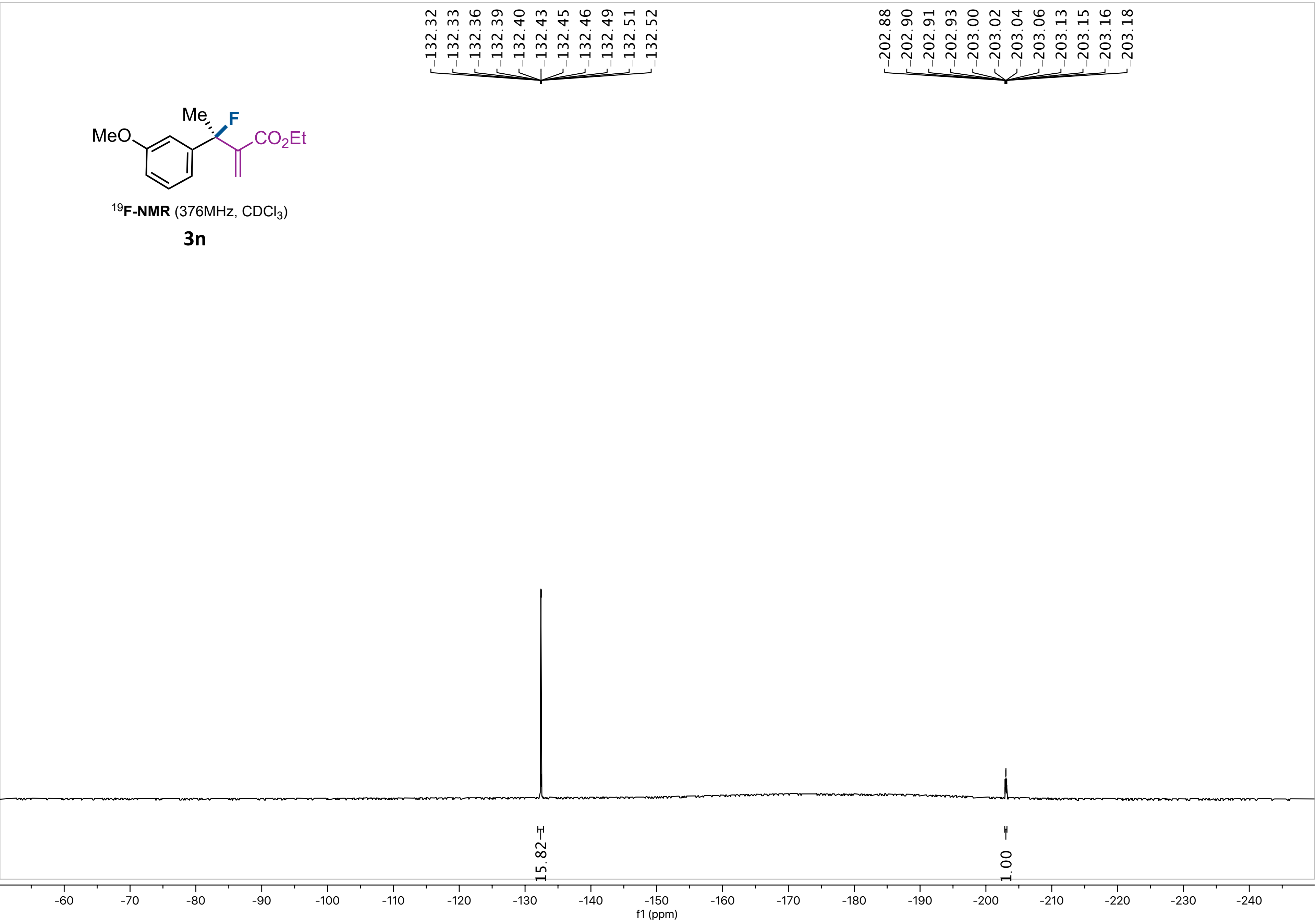
-132.32
-132.33
-132.36
-132.39
-132.40
-132.43
-132.45
-132.46
-132.49
-132.51
-132.52
-202.88
-202.90
-202.91
-202.93
-203.00
-203.02
-203.04
-203.06
-203.13
-203.15
-203.16
-203.18
Me
F
MeO
CO2Et
19F-NMR (376MHz, CDCl3)
3n
15.82
1.00
-60
-70
-80
-90
-100
-110
-120
-130
-140
-150
-160
-170
-180
-190
-200
-210
-220
-230
-240
f1 (ppm)

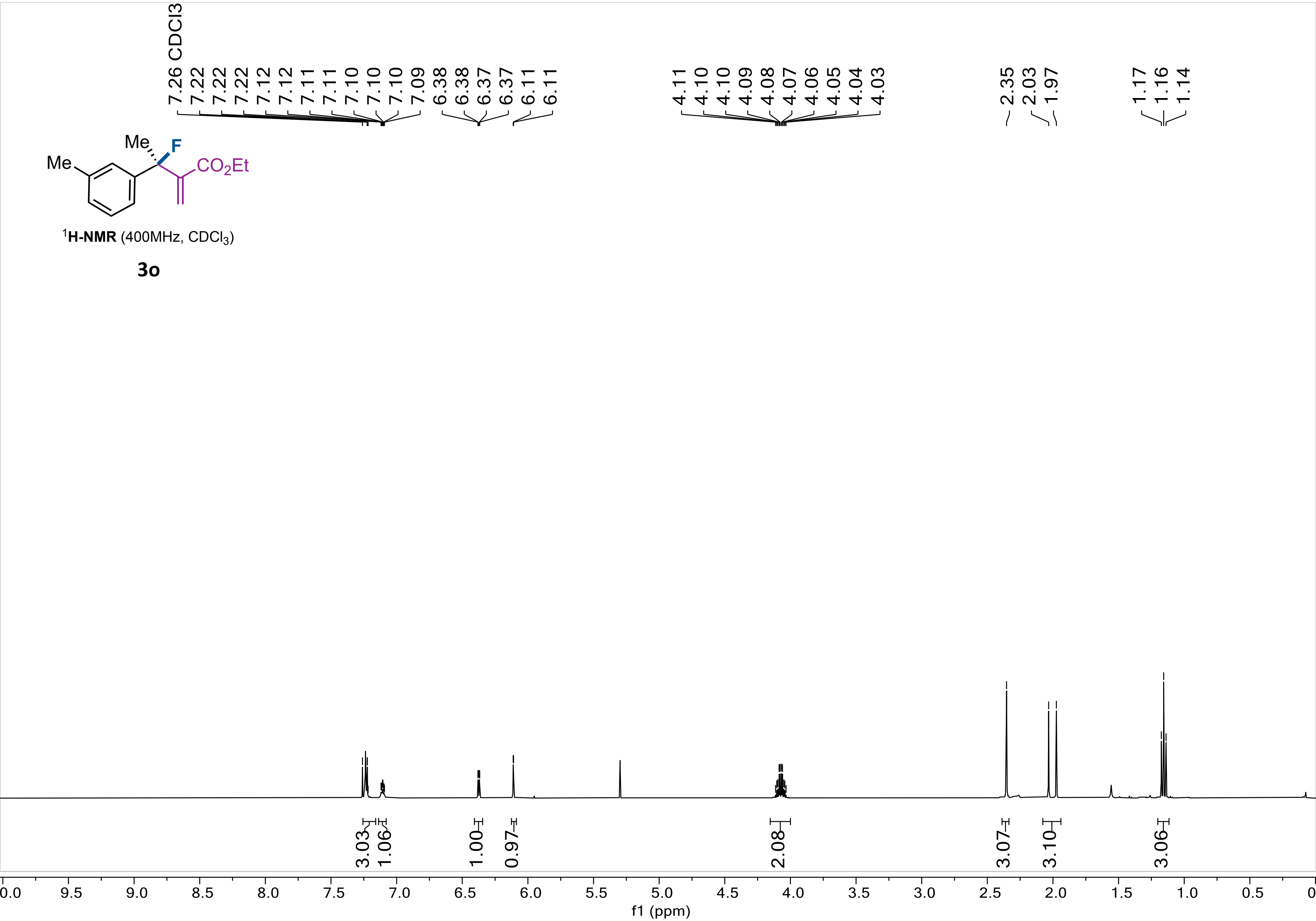

7.26 CDCl3
7.22
7.22
7.22
7.12
7.12
7.11
7.11
7.10
7.10
7.10
7.09
6.38
6.38
6.37
6.37
6.11
6.11
4.11
4.10
4.10
4.09
4.08
4.07
4.06
4.05
4.04
4.03
2.35
2.03
1.97
1.17
1.16
1.14
Me
F
Me
CO2Et
1H-NMR (400MHz, CDCl3)
3o
3.03
1.06
1.00
0.97
2.08
3.07
3.10
3.06
f1 (ppm)

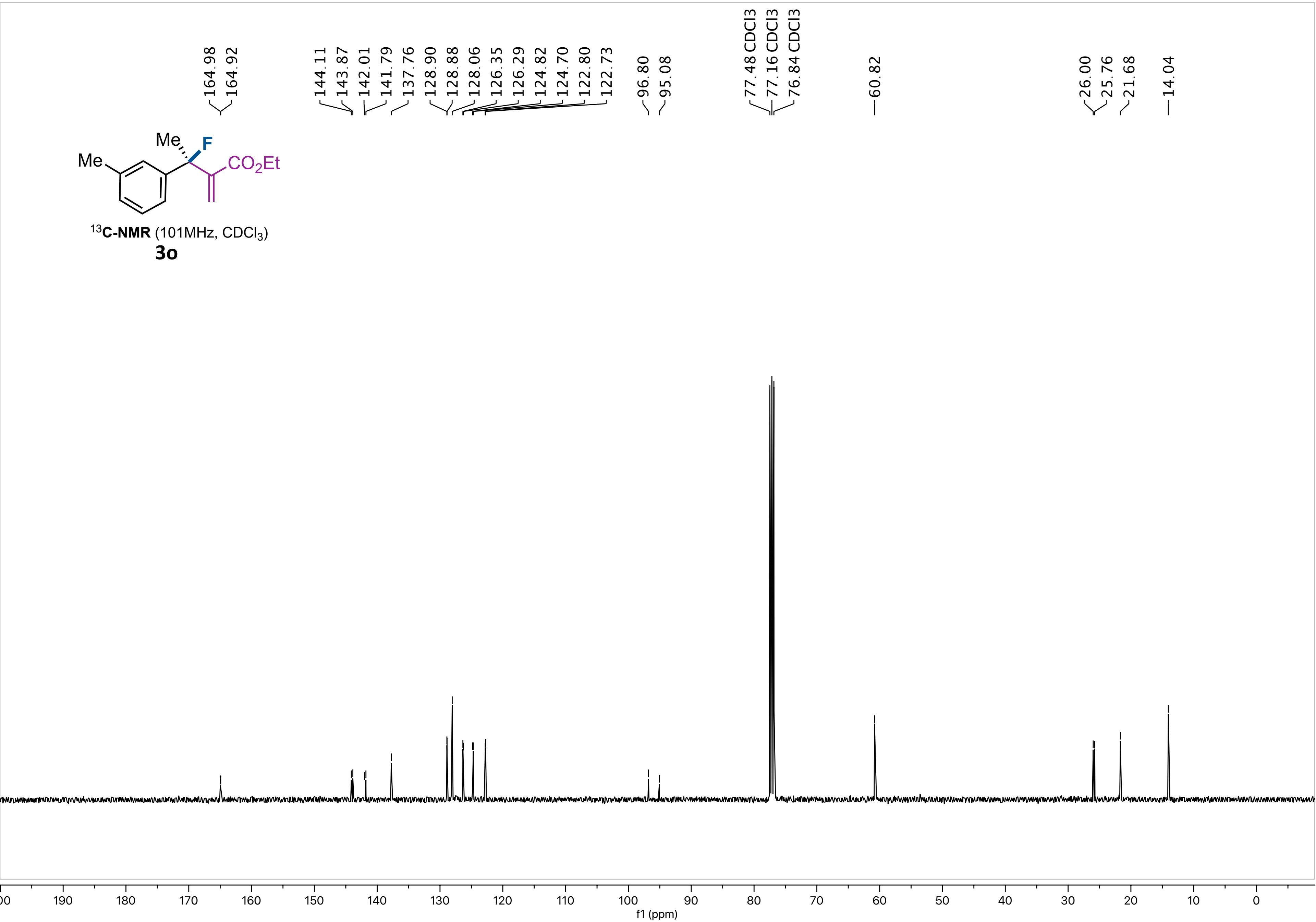
164.98
164.92
144.11
143.87
142.01
141.79
137.76
128.90
128.88
128.06
126.35
126.29
124.82
124.70
122.80
122.73
96.80
95.08
77.48 CDCl3
77.16 CDCl3
76.84 CDCl3
60.82
26.00
25.76
21.68
14.04
Me
F
Me
CO2Et
13C-NMR (101MHz, CDCl3)
3o
f1 (ppm)

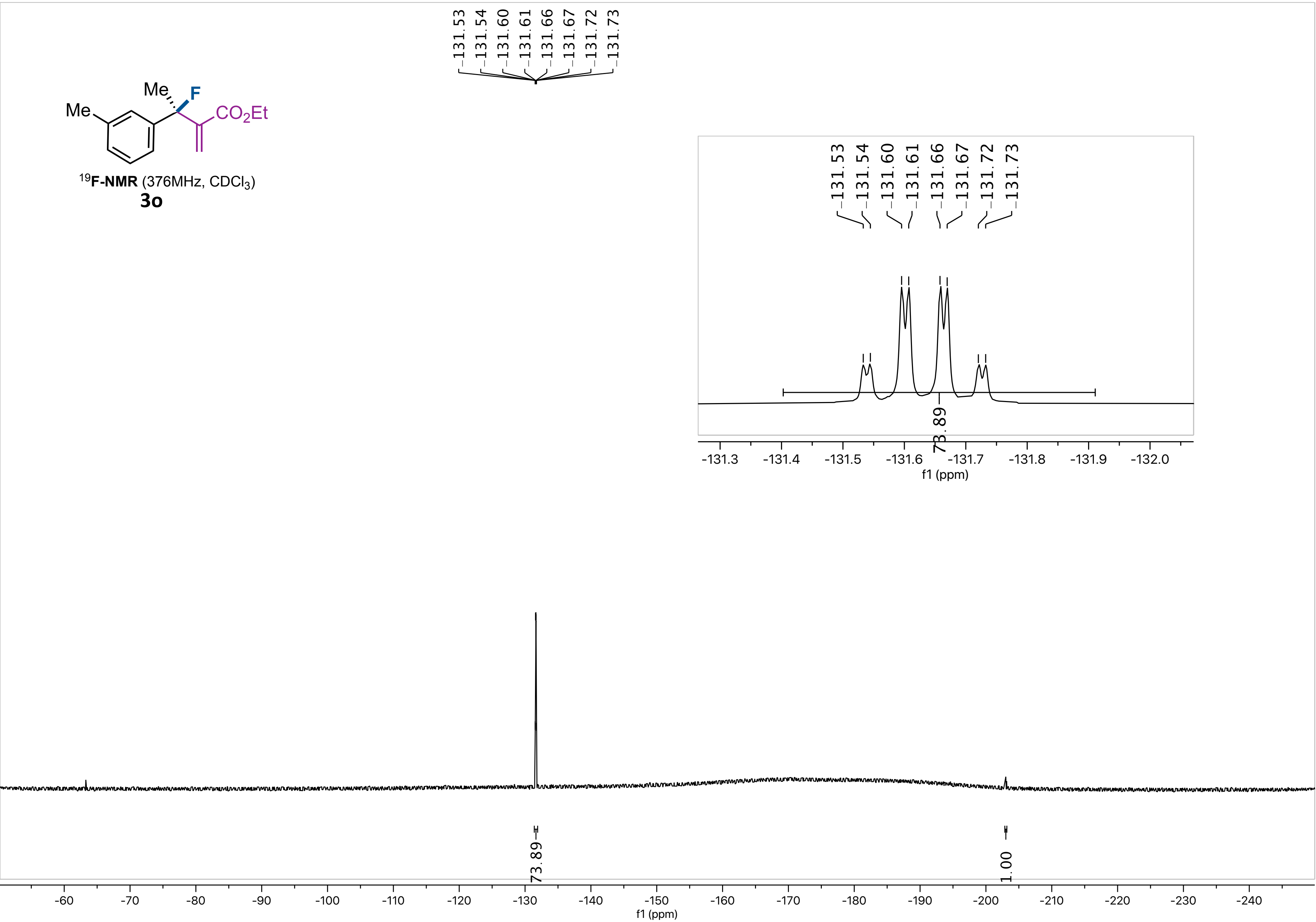

^{19}**F-NMR** (376MHz, $CDCl_3$)

3o

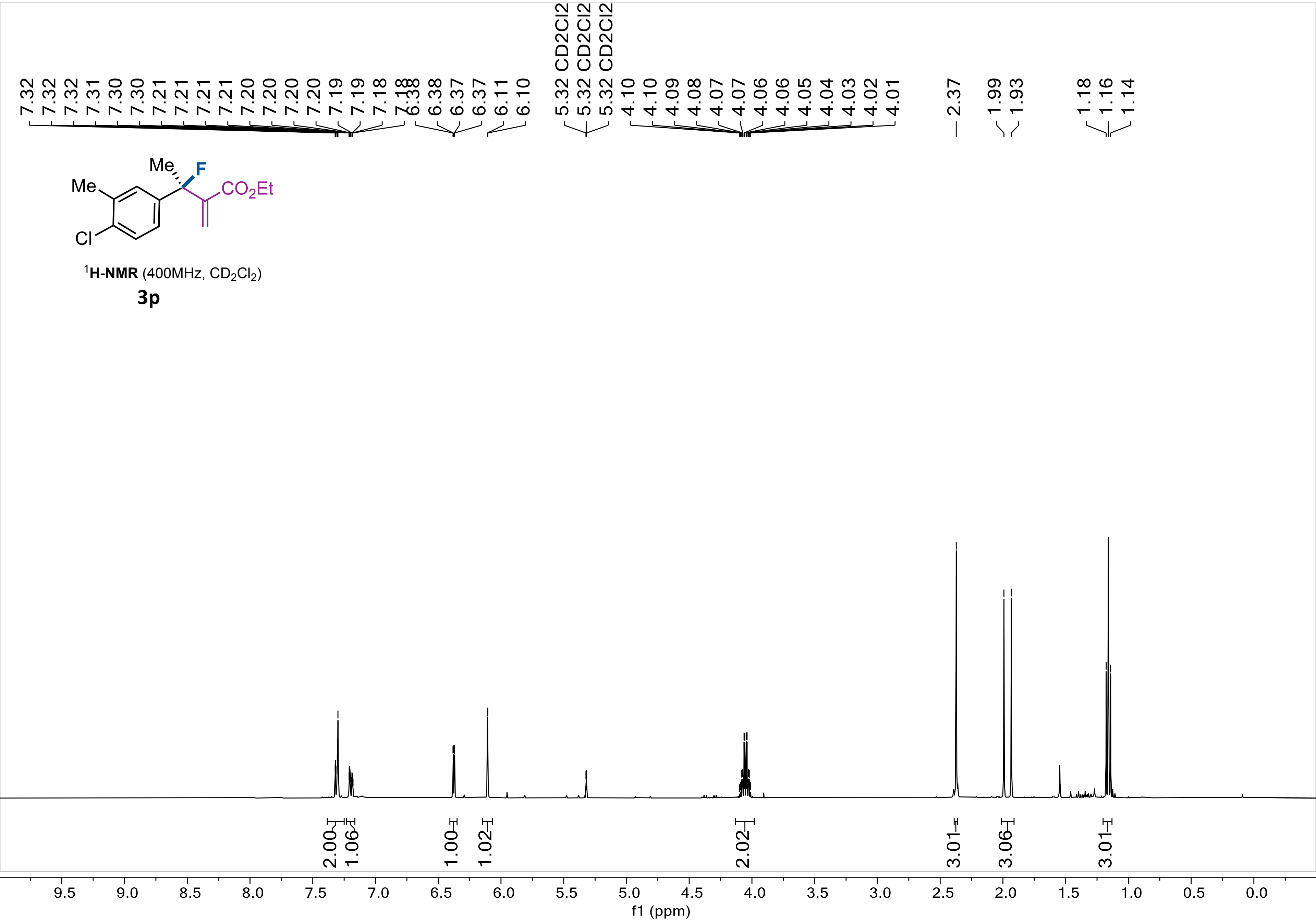
7.32 7.32 7.32 7.31 7.30 7.30 7.21 7.21 7.21 7.21 7.20 7.20 7.20 7.20 7.19 7.19 7.18 7.18 6.38 6.38 6.37 6.37 6.11 6.10
5.32 CD2Cl2 5.32 CD2Cl2 5.32 CD2Cl2
4.10 4.10 4.09 4.08 4.07 4.07 4.06 4.06 4.05 4.04 4.03 4.02 4.01
2.37
1.99 1.93
1.18 1.16 1.14
Me
F
Me
CO2Et
Cl
1H-NMR (400MHz, CD2Cl2)
3p
2.00 1.06 1.00 1.02 2.02 3.01 3.06 3.01
9.5 9.0 8.5 8.0 7.5 7.0 6.5 6.0 5.5 5.0 4.5 4.0 3.5 3.0 2.5 2.0 1.5 1.0 0.5 0.0
f1 (ppm)

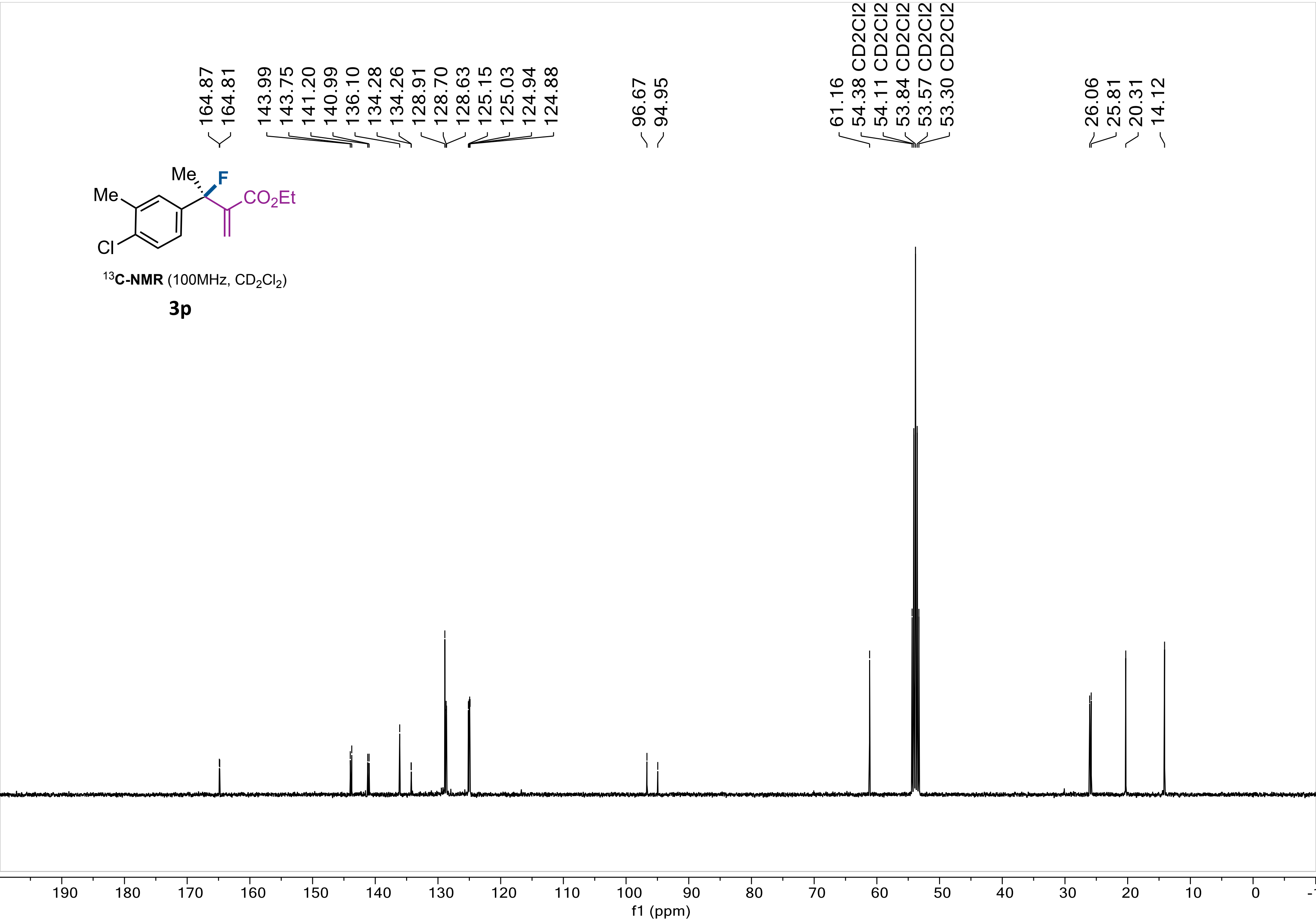

^{13}**C-NMR** (100MHz, CD_2Cl_2)

3p

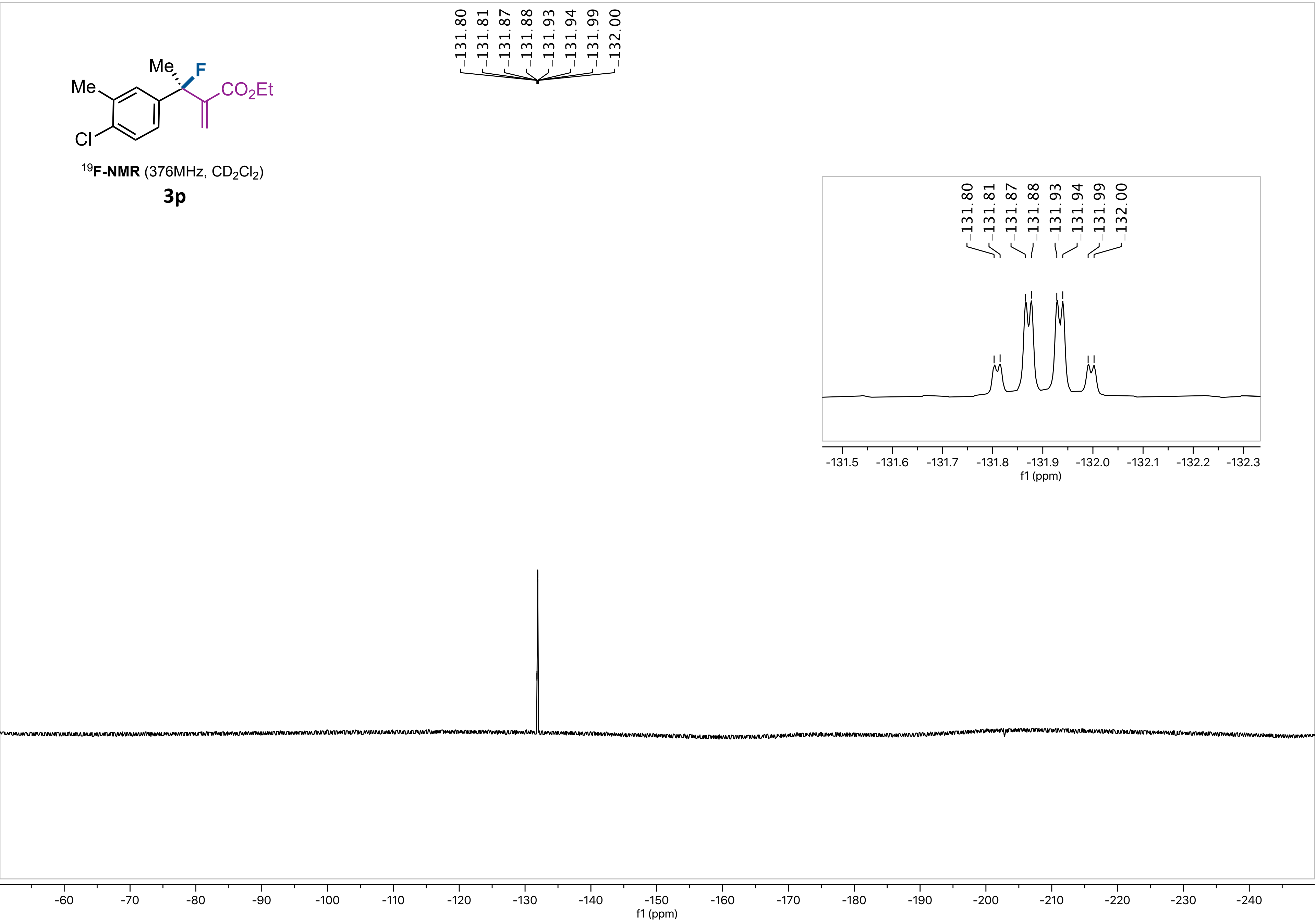

[19]**F-NMR** (376MHz, CD_2Cl_2)

3p

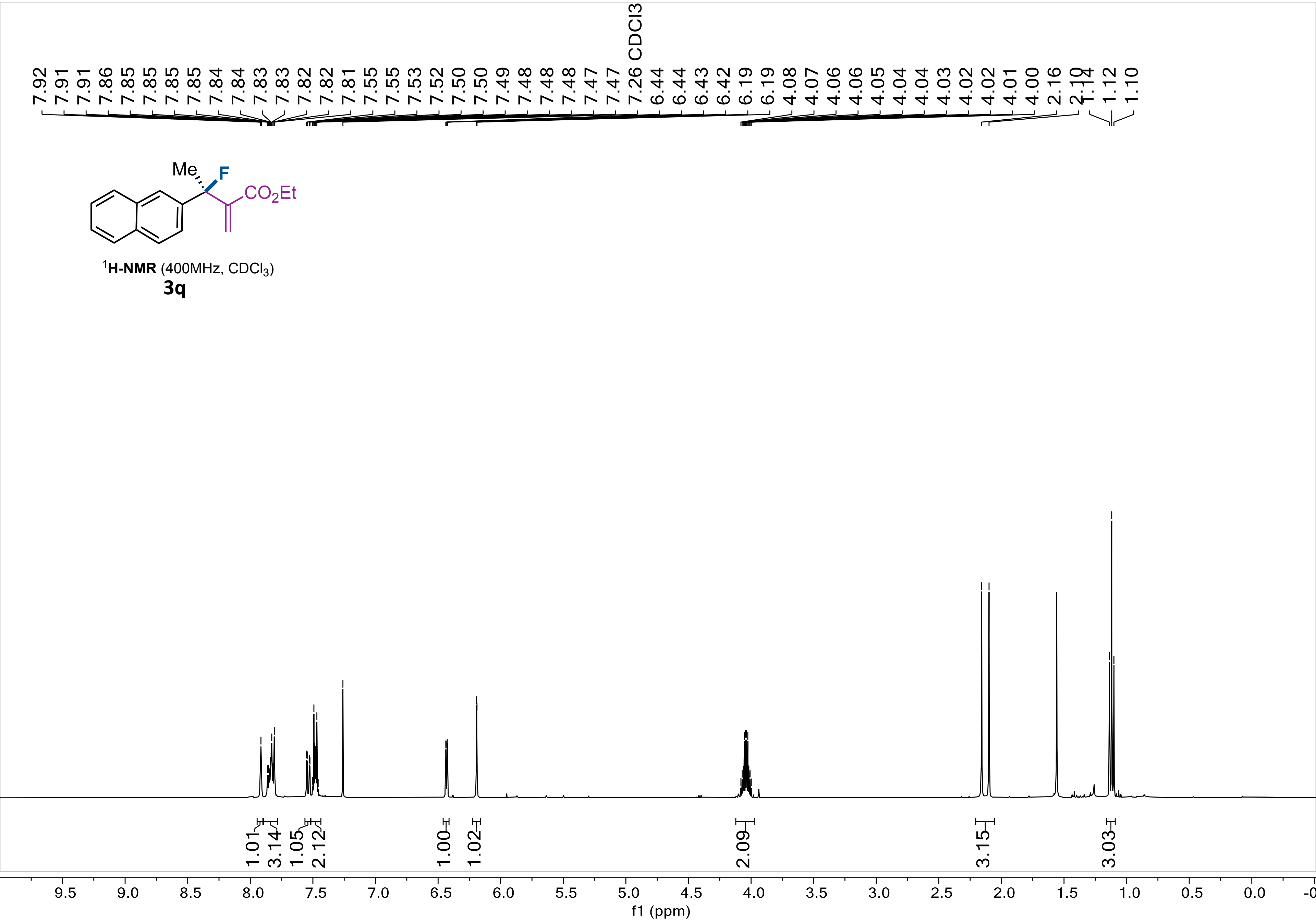

7.92 7.91 7.91 7.86 7.85 7.85 7.85 7.85 7.84 7.84 7.83 7.83 7.82 7.82 7.81 7.55 7.55 7.53 7.52 7.50 7.50 7.49 7.48 7.48 7.48 7.47 7.47 7.26 CDCl3 6.44 6.44 6.43 6.42 6.19 6.19 4.08 4.07 4.06 4.06 4.05 4.04 4.04 4.03 4.02 4.02 4.01 4.00 2.16 2.10 1.14 1.12 1.10
Me
F
CO2Et
1H-NMR (400MHz, CDCl3)
3q
1.01 3.14 1.05 2.12 1.00 1.02 2.09 3.15 3.03
9.5 9.0 8.5 8.0 7.5 7.0 6.5 6.0 5.5 5.0 4.5 4.0 3.5 3.0 2.5 2.0 1.5 1.0 0.5 0.0
f1 (ppm)

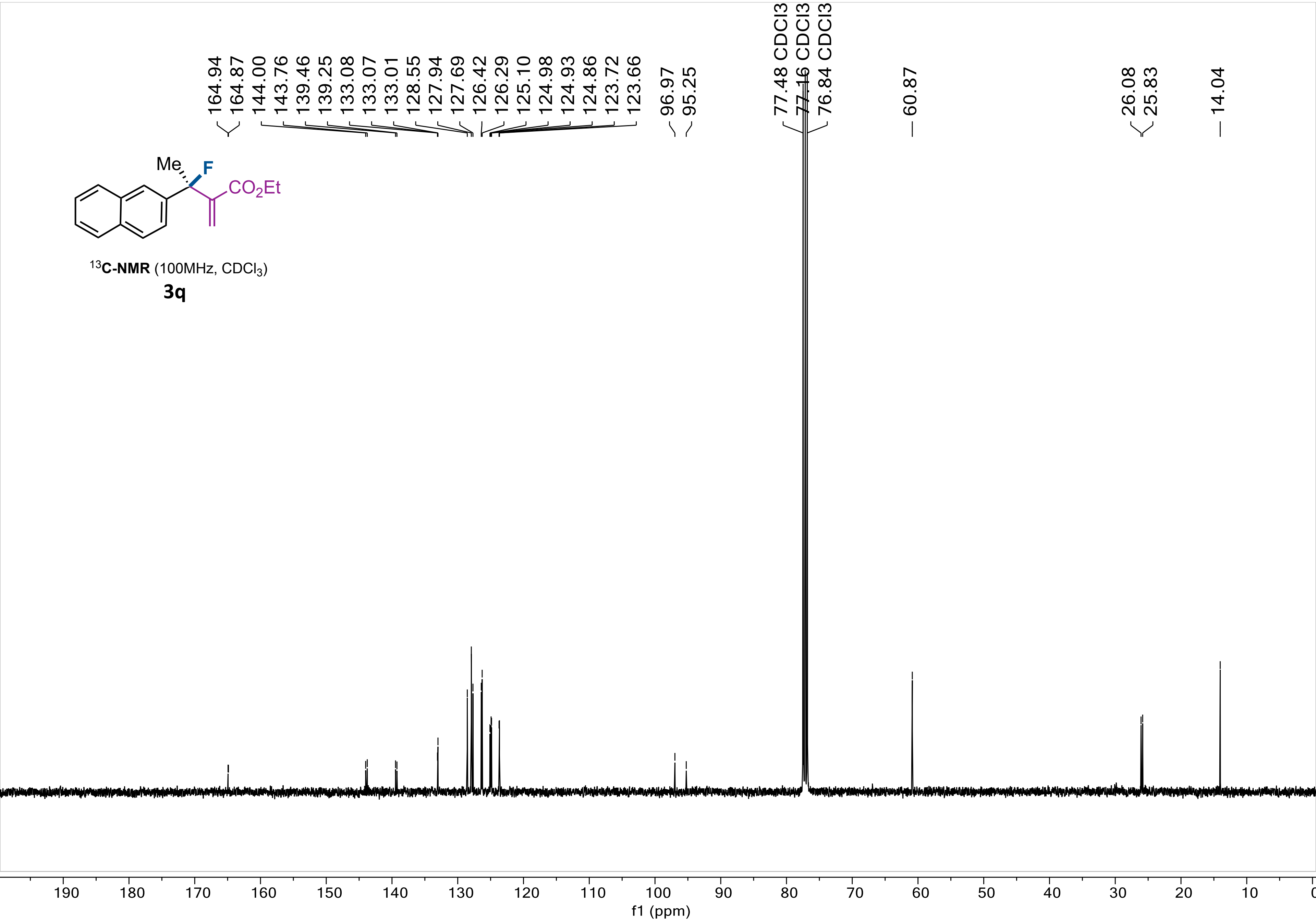

^{13}**C-NMR** (100MHz, $CDCl_3$)

3q

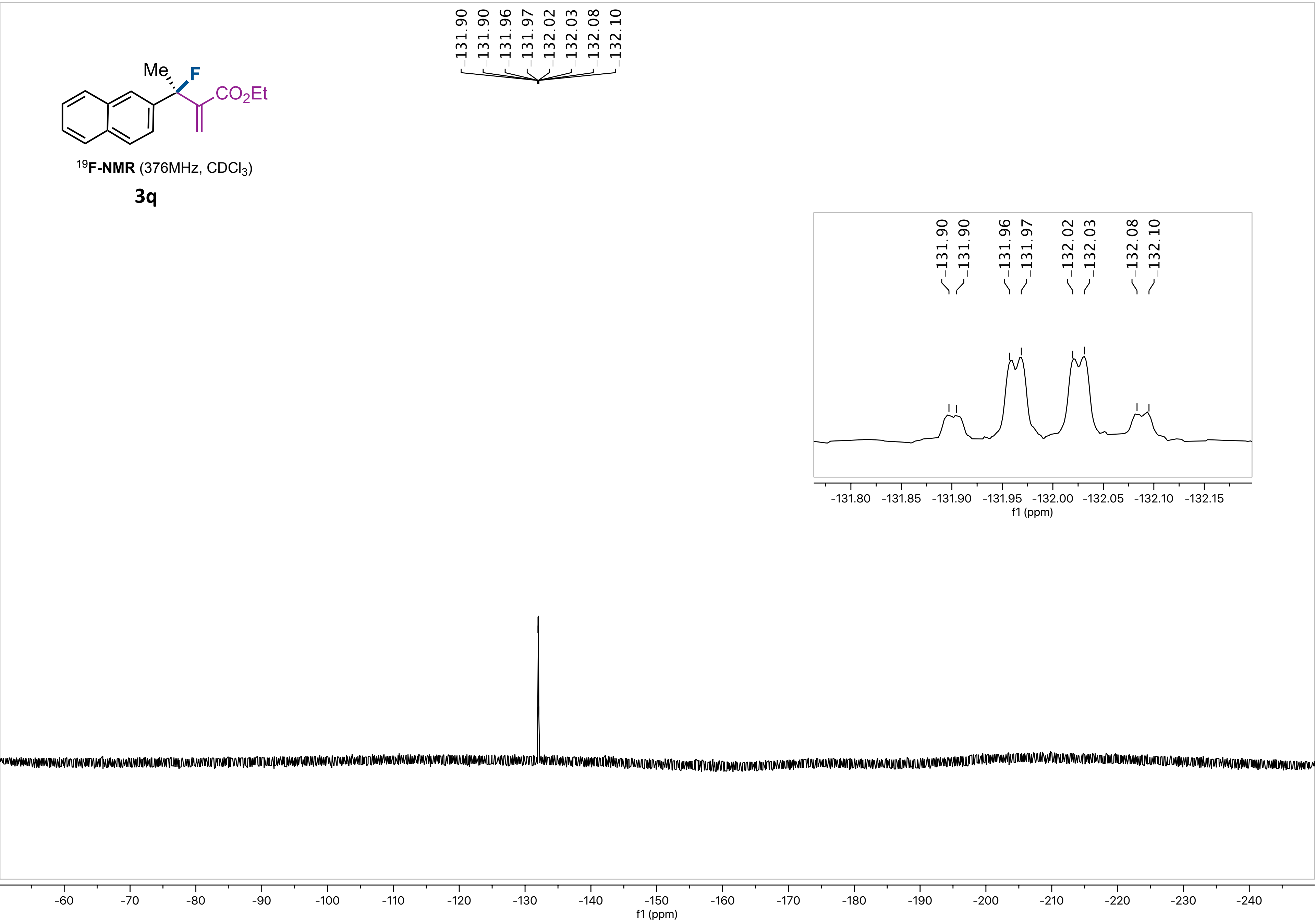

^{19}**F-NMR** (376MHz, $CDCl_3$)

3q

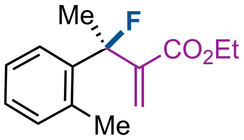

[1]**H-NMR** (400MHz, $CDCl_3$)

3r

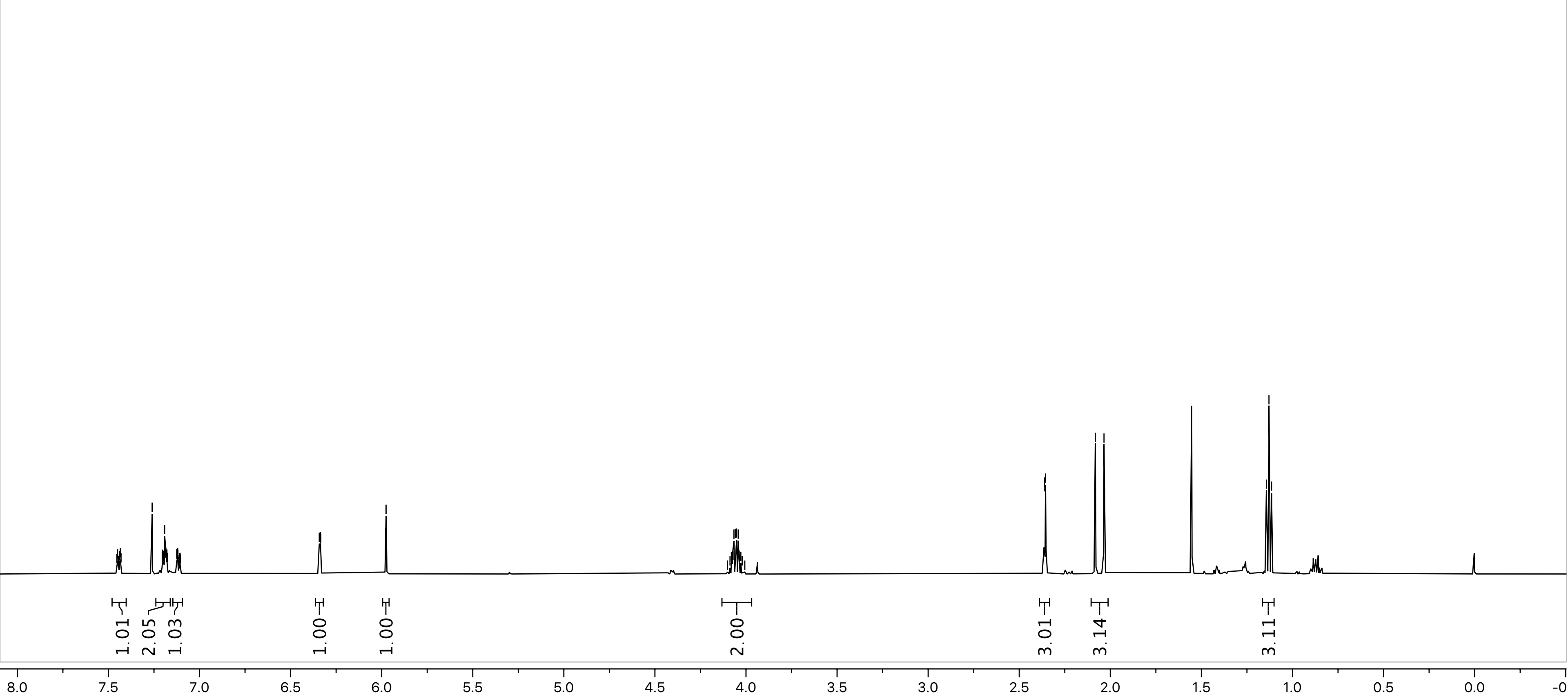

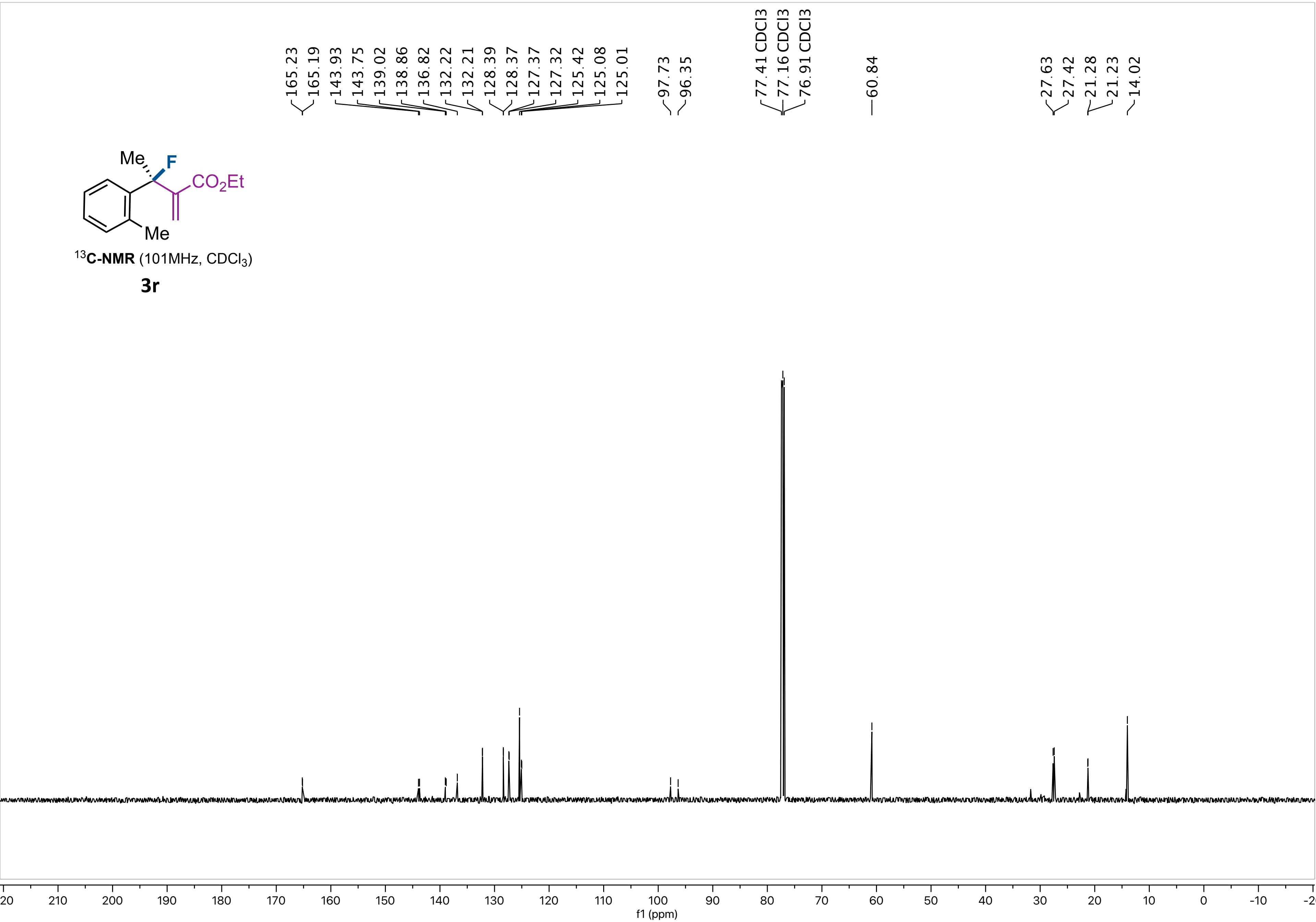

^{13}**C-NMR** (101MHz, $CDCl_3$)

3r

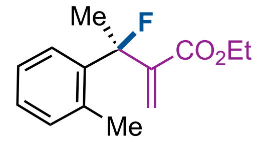

^{19}**F-NMR** (376MHz, $CDCl_3$)

3r

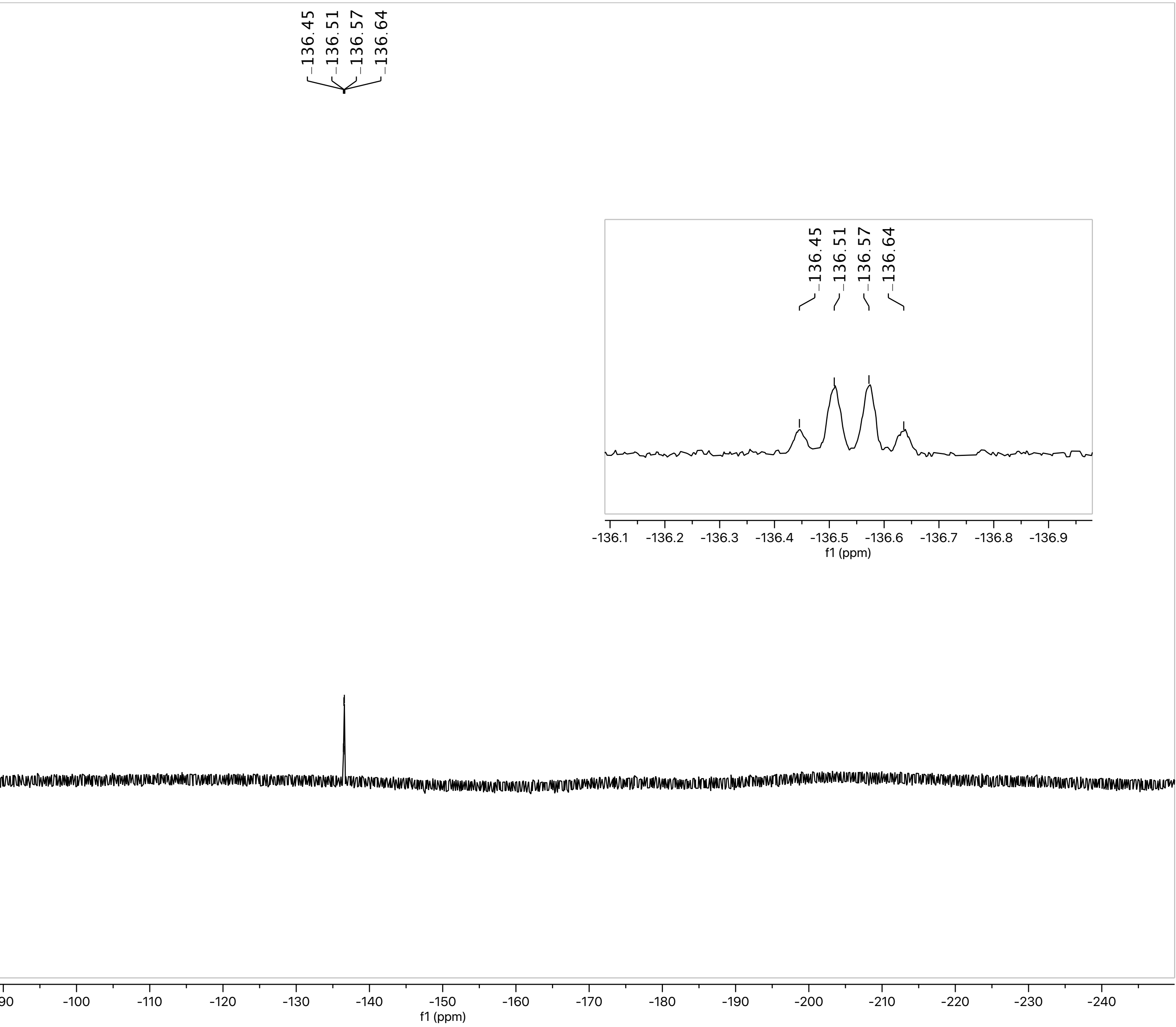

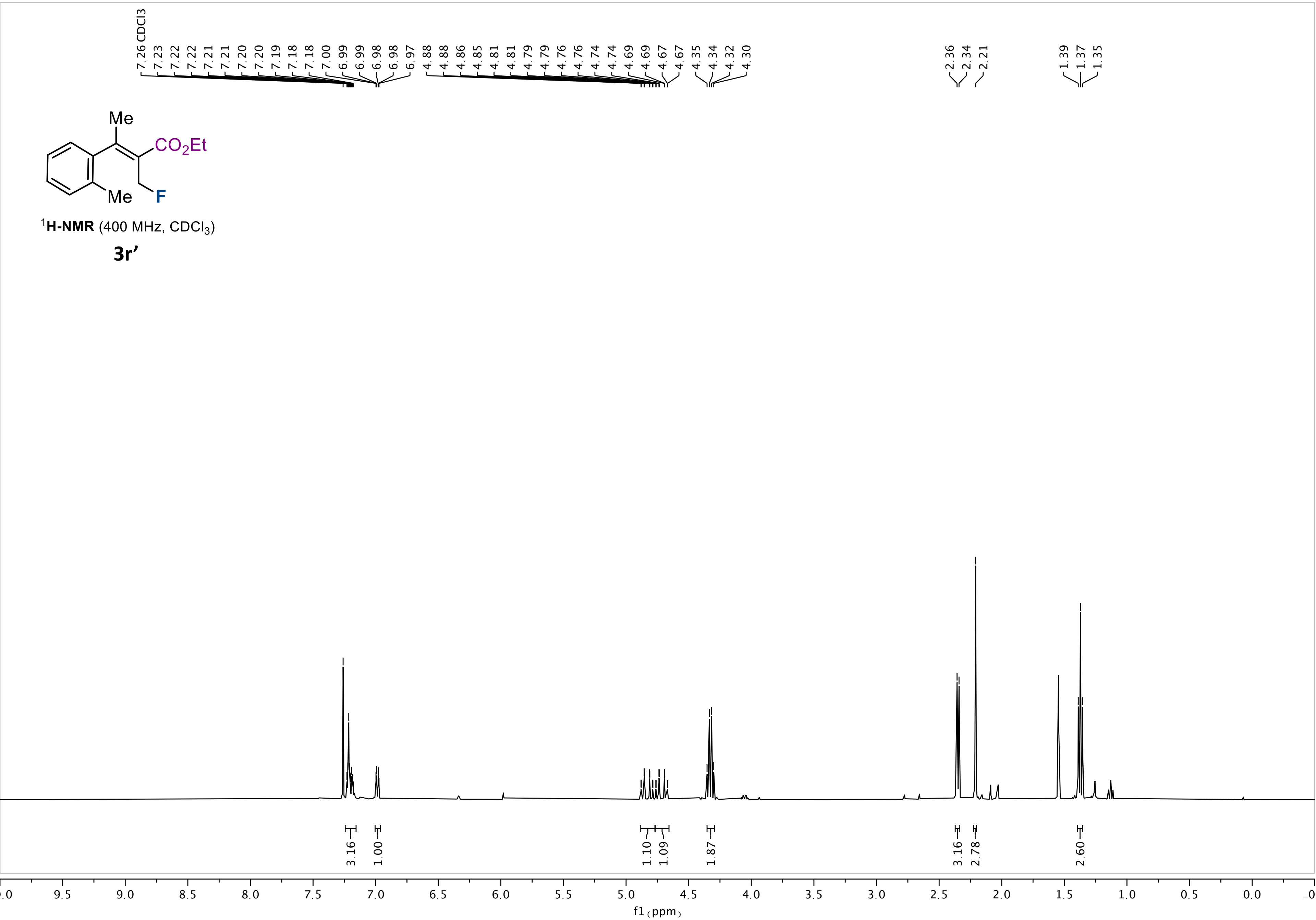
Me
CO_2Et
Me
F
^{1}H-NMR (400 MHz, $CDCl_3$)
3r'
7.26 CDCl3
7.23
7.22
7.22
7.21
7.21
7.20
7.20
7.19
7.18
7.18
7.00
6.99
6.99
6.98
6.98
6.97
4.88
4.88
4.86
4.85
4.81
4.81
4.79
4.79
4.76
4.76
4.74
4.74
4.69
4.69
4.67
4.67
4.35
4.34
4.32
4.30
2.36
2.34
2.21
1.39
1.37
1.35
3.16
1.00
1.10
1.09
1.87
3.16
2.78
2.60
f1 (ppm)

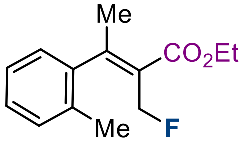

^{13}**C-NMR** (101 MHz, $CDCl_3$)

3r’

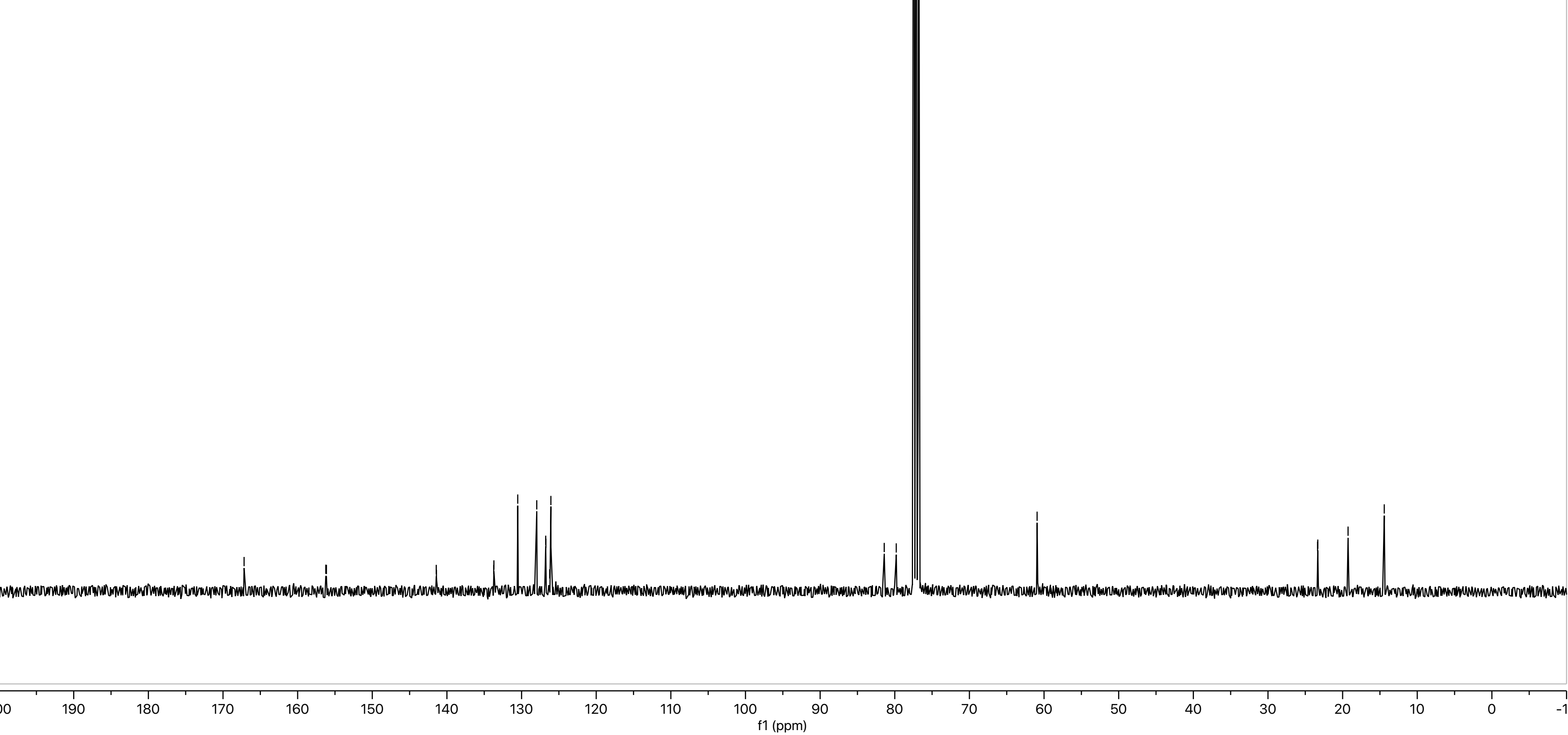

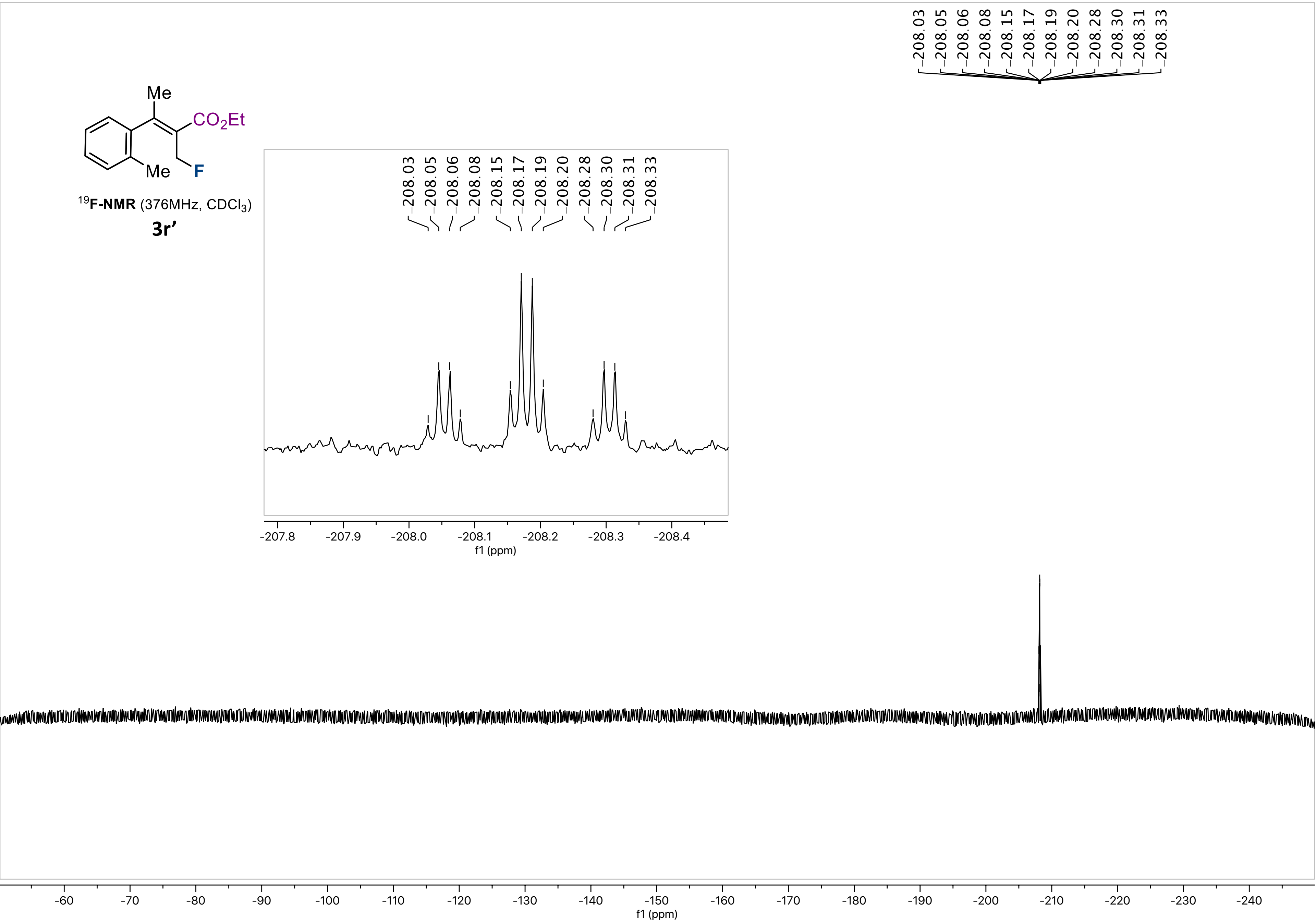
Me
CO_2Et
Me
F
^{19}F-NMR (376MHz, $CDCl_3$)
3r'
-208.03
-208.05
-208.06
-208.08
-208.15
-208.17
-208.19
-208.20
-208.28
-208.30
-208.31
-208.33
-207.8
-207.9
-208.0
-208.1
-208.2
-208.3
-208.4
f1 (ppm)
-60
-70
-80
-90
-100
-110
-120
-130
-140
-150
-160
-170
-180
-190
-200
-210
-220
-230
-240
f1 (ppm)

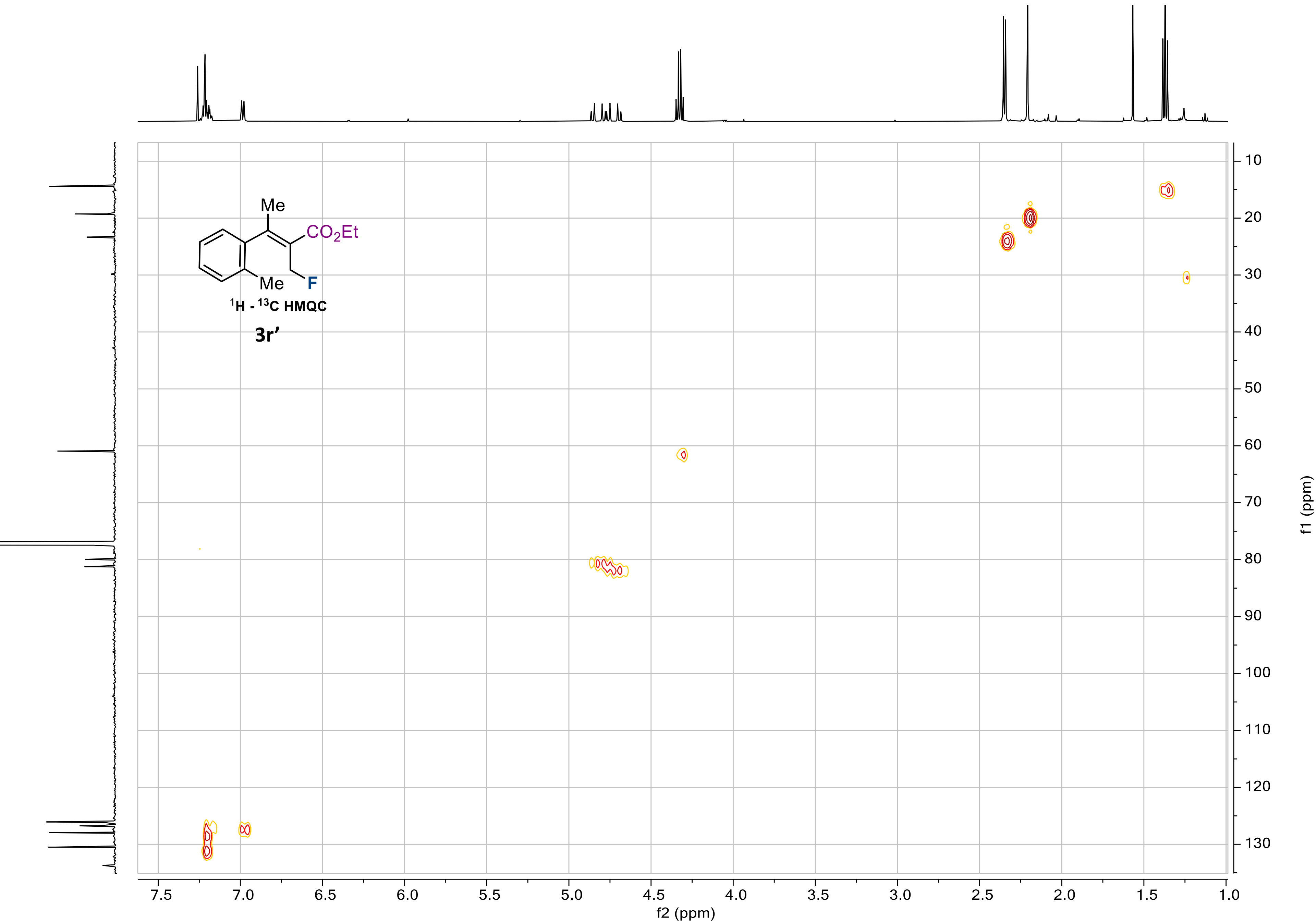
Me
CO2Et
Me
F
1H - 13C HMQC
3r'
f2 (ppm)
f1 (ppm)

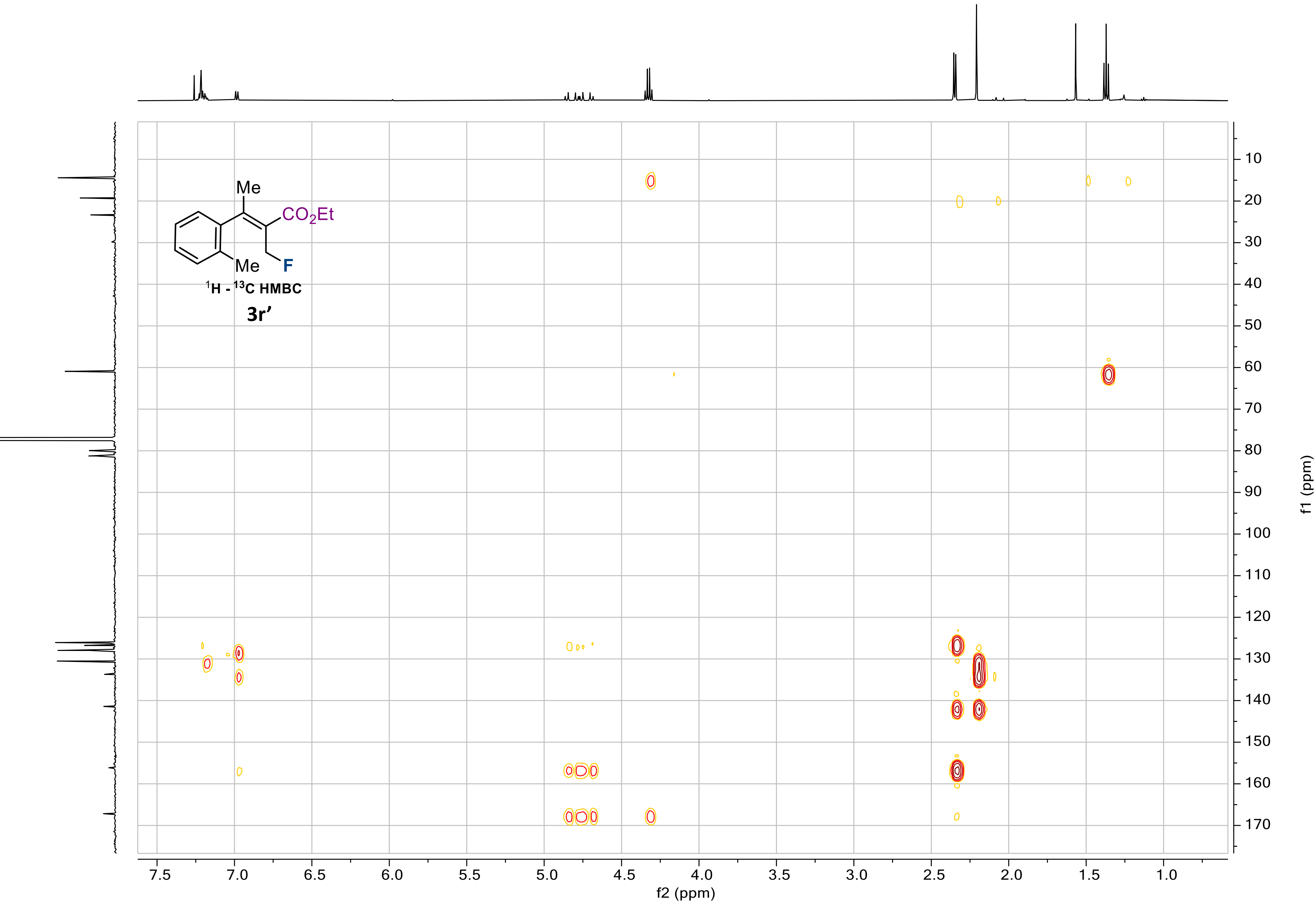
Me
CO_2Et
Me
F
^1H - ^{13}C HMBC
3r'
f1 (ppm)
f2 (ppm)

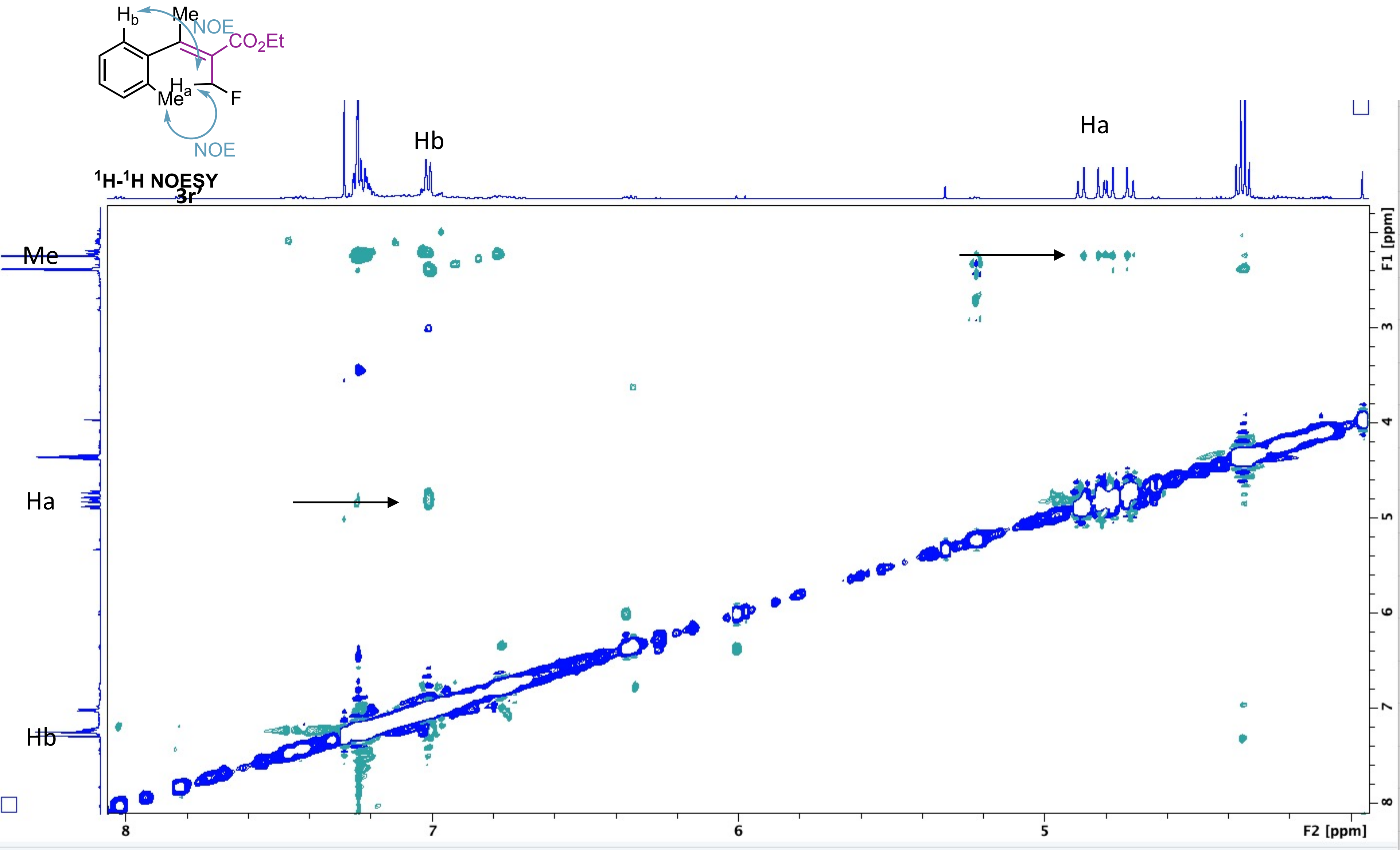

Hb
Me
NOE
CO2Et
Ha
Mea
F
NOE
1H-1H NOESY
3r'
Hb
Ha
Me
Ha
Hb
F1 [ppm]
F2 [ppm]

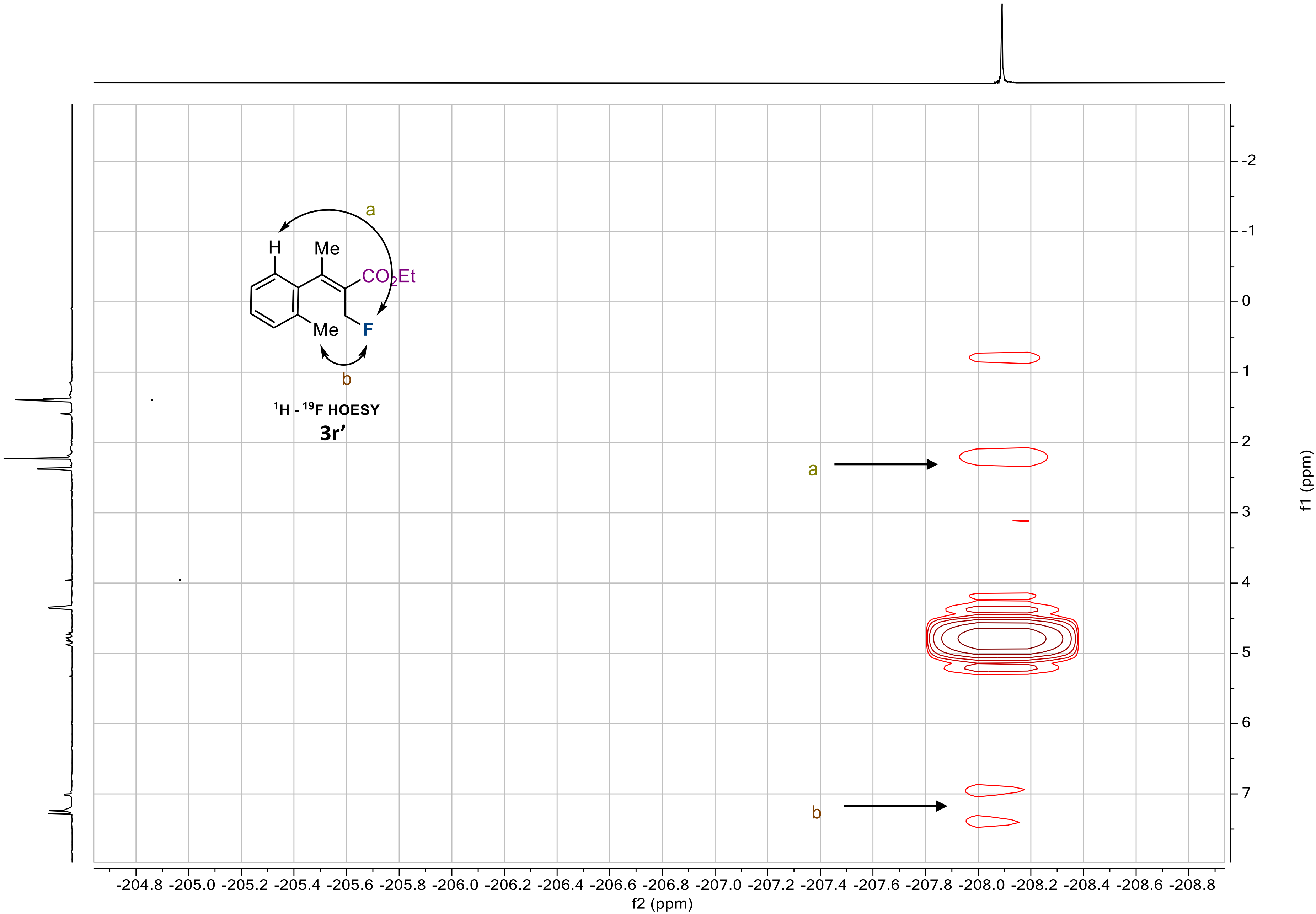

a
H
Me
CO_2Et
Me
F
b
^1H - ^{19}F HOESY
3r’
a
b
-204.8 -205.0 -205.2 -205.4 -205.6 -205.8 -206.0 -206.2 -206.4 -206.6 -206.8 -207.0 -207.2 -207.4 -207.6 -207.8 -208.0 -208.2 -208.4 -208.6 -208.8
f2 (ppm)
-2
-1
0
1
2
3
4
5
6
7
f1 (ppm)

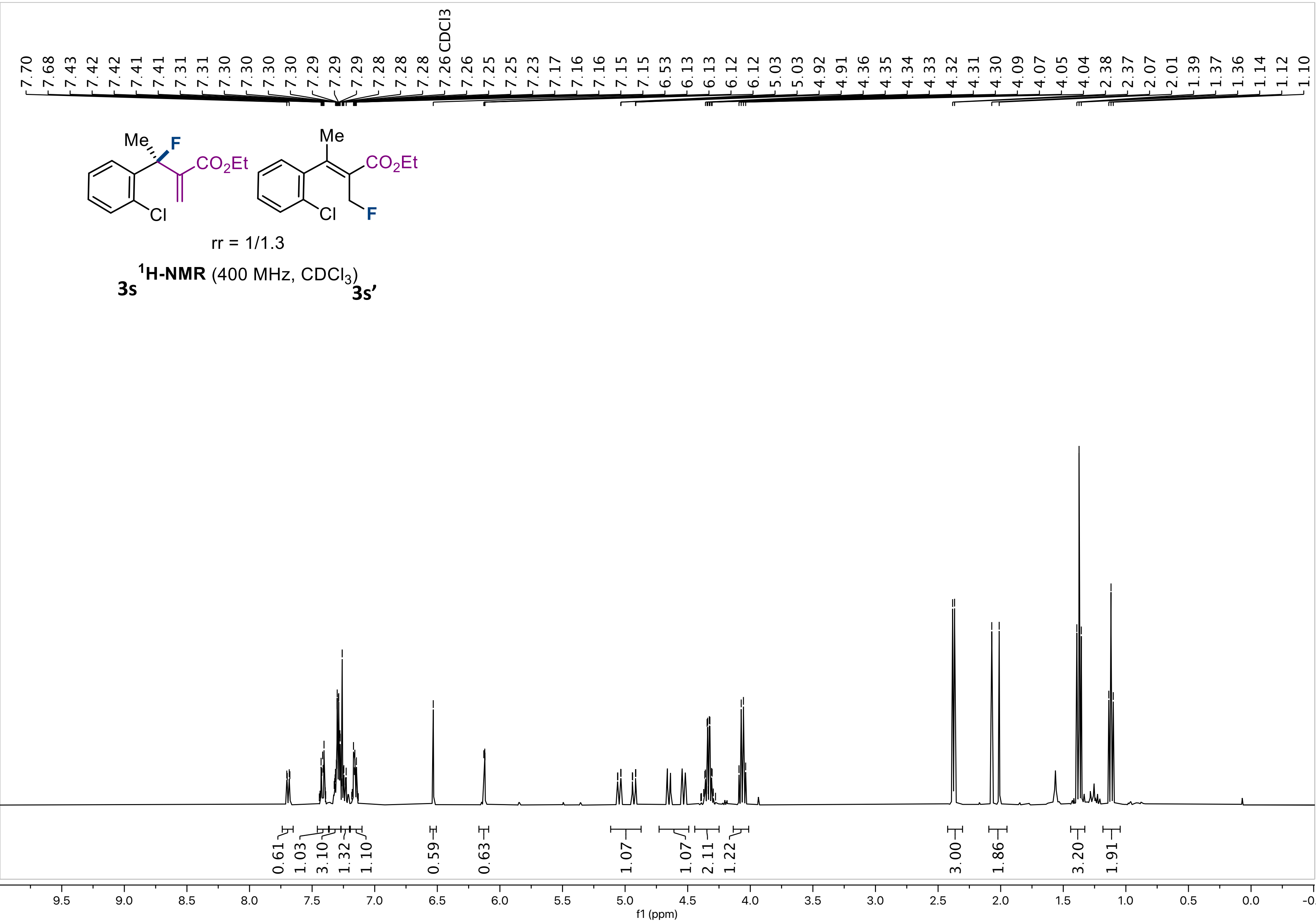
Me
F
CO2Et
Cl
Me
CO2Et
Cl
F
rr = 1/1.3
3s
1H-NMR (400 MHz, CDCl3)
3s'
CDCl3
f1 (ppm)

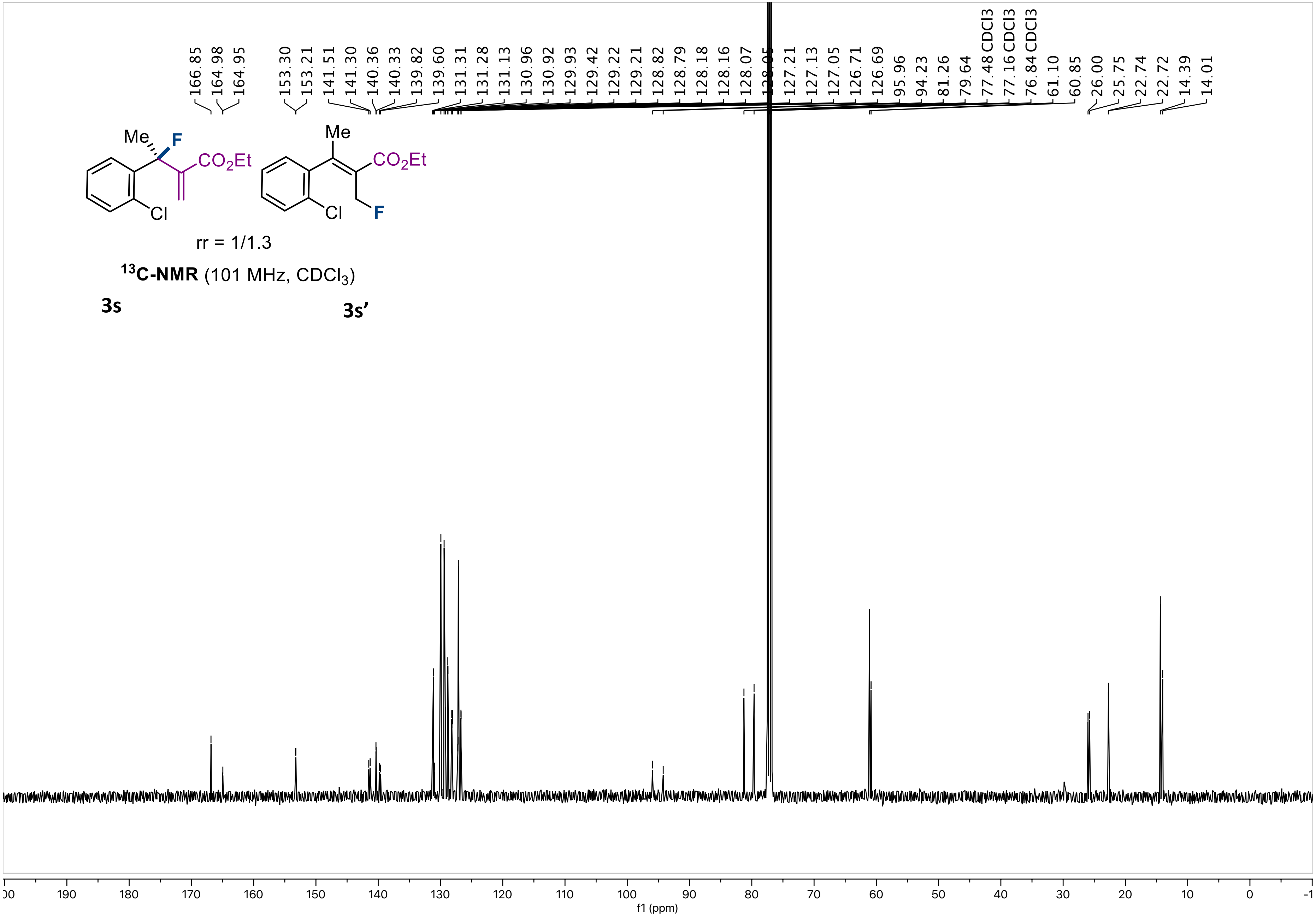

166.85
164.98
164.95
153.30
153.21
141.51
141.30
140.36
140.33
139.82
139.60
131.31
131.28
131.13
130.96
130.92
129.93
129.42
129.22
129.21
128.82
128.79
128.18
128.16
128.07
127.21
127.13
127.05
126.71
126.69
95.96
94.23
81.26
79.64
77.48 CDCl3
77.16 CDCl3
76.84 CDCl3
61.10
60.85
26.00
25.75
22.74
22.72
14.39
14.01
Me
F
CO2Et
Cl
Me
CO2Et
Cl
F
rr = 1/1.3
13C-NMR (101 MHz, CDCl3)
3s
3s'
f1 (ppm)

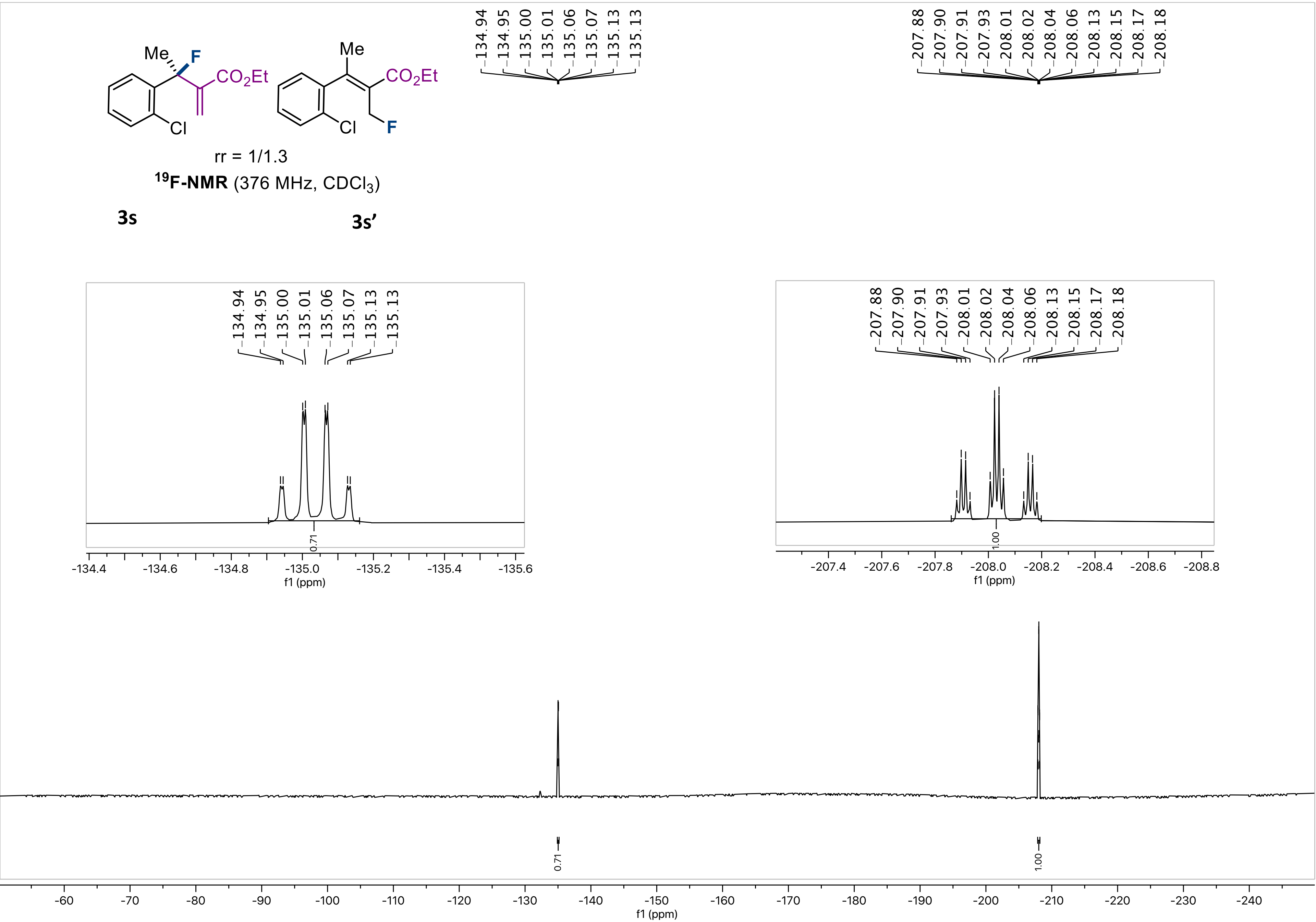

Me
F
CO_2Et
Cl
Me
CO_2Et
Cl
F
rr = 1/1.3
^{19}F-NMR (376 MHz, $CDCl_3$)
3s
3s'
-134.94
-134.95
-135.00
-135.01
-135.06
-135.07
-135.13
-135.13
-207.88
-207.90
-207.91
-207.93
-208.01
-208.02
-208.04
-208.06
-208.13
-208.15
-208.17
-208.18
0.71
1.00
f1 (ppm)

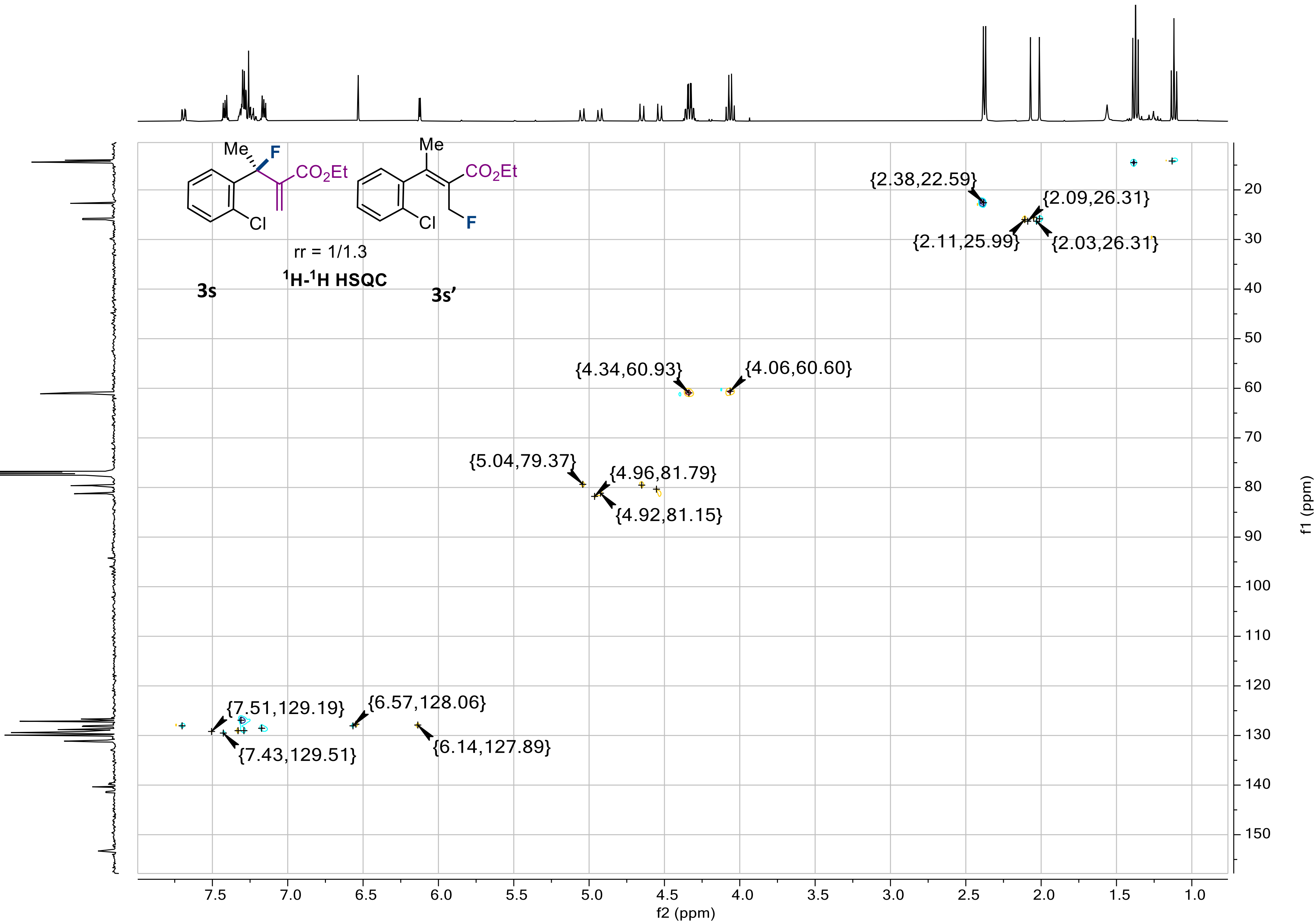

Me
F
CO2Et
Cl
Me
CO2Et
Cl
F
rr = 1/1.3
1H-1H HSQC
3s
3s'
{2.38,22.59}
{2.09,26.31}
{2.11,25.99}
{2.03,26.31}
{4.34,60.93}
{4.06,60.60}
{5.04,79.37}
{4.96,81.79}
{4.92,81.15}
{7.51,129.19}
{6.57,128.06}
{7.43,129.51}
{6.14,127.89}
f1 (ppm)
f2 (ppm)

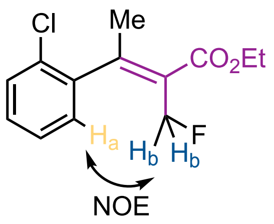

3s' ^{1}H-^{1}H NOESY

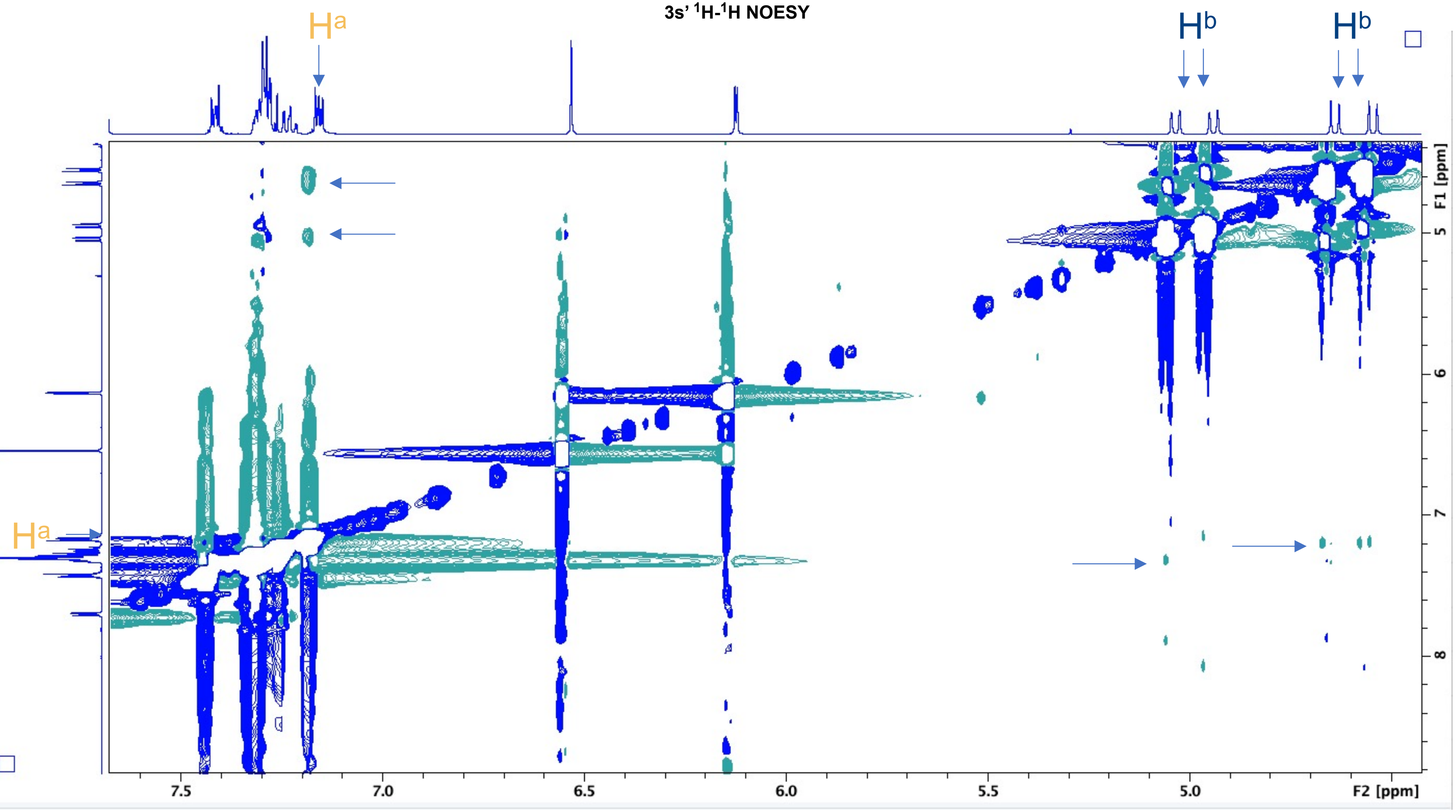

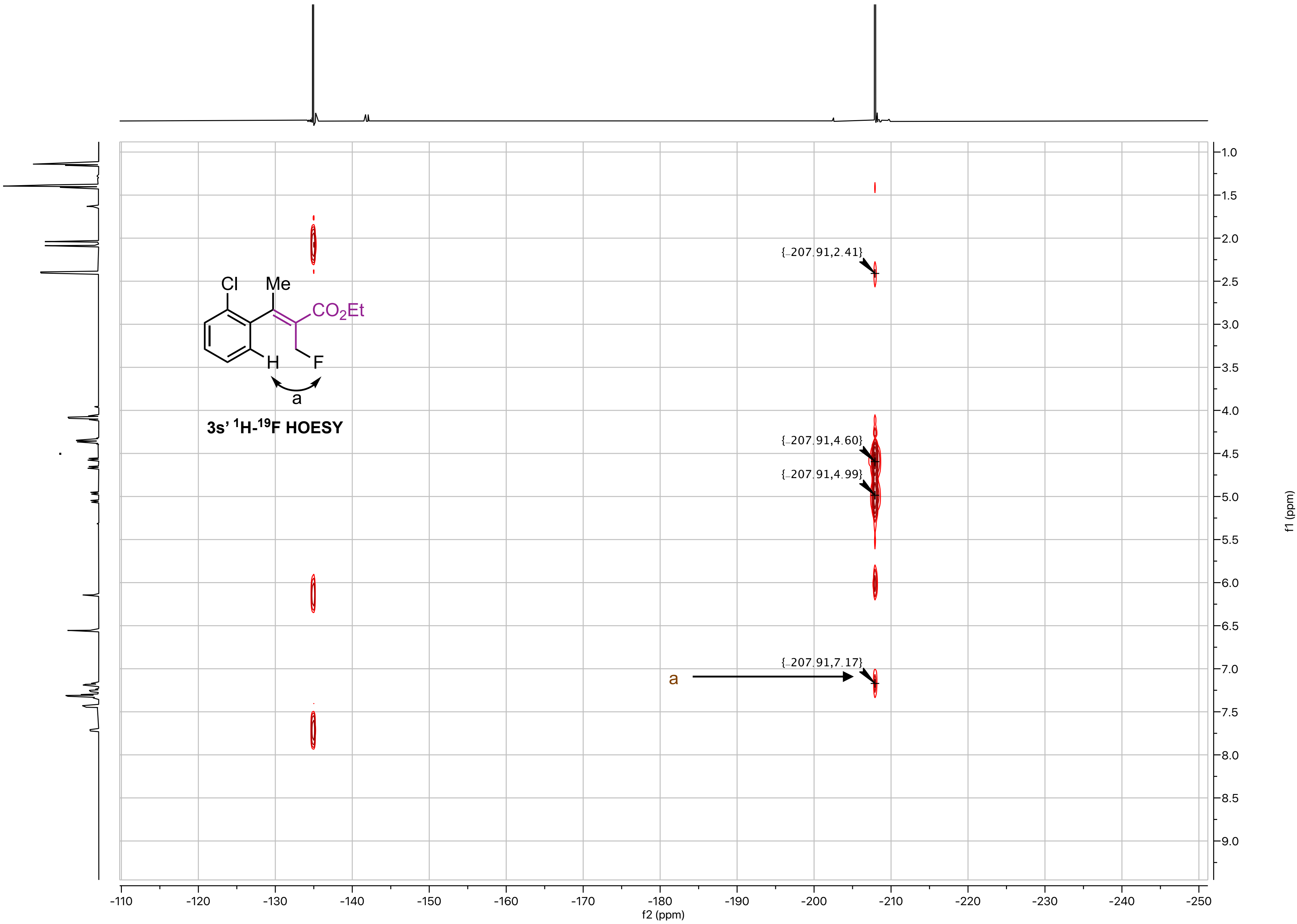

Cl
Me
CO2Et
H
F
a
3s' 1H-19F HOESY
{-207.91,2.41}
{-207.91,4.60}
{-207.91,4.99}
{-207.91,7.17}
a
f2 (ppm)
f1 (ppm)

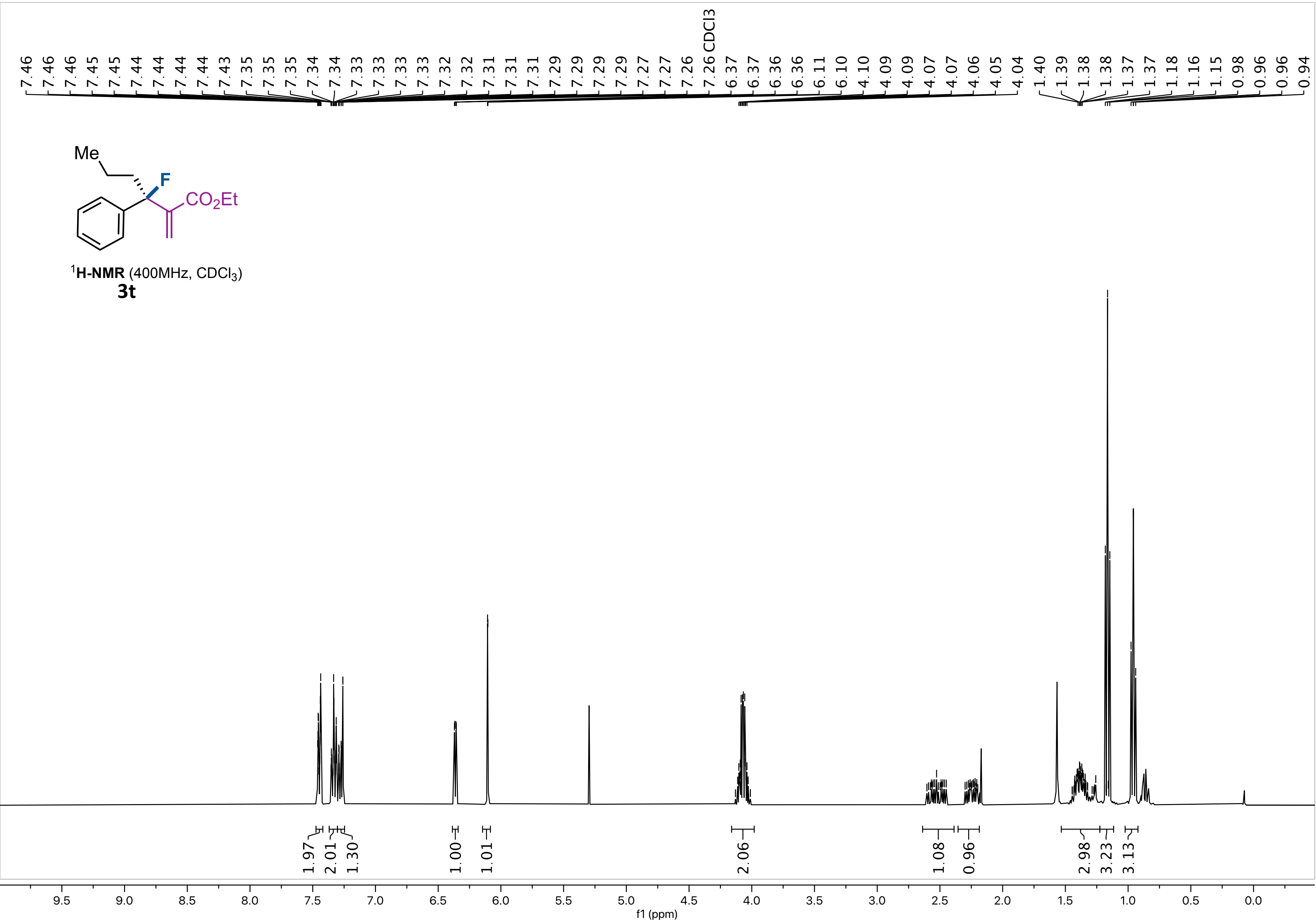

7.46 7.46 7.46 7.45 7.45 7.44 7.44 7.44 7.44 7.43 7.35 7.35 7.35 7.34 7.34 7.33 7.33 7.33 7.33 7.32 7.32 7.31 7.31 7.31 7.29 7.29 7.29 7.29 7.27 7.27 7.26 7.26 CDCl3 6.37 6.37 6.36 6.36 6.11 6.10 4.10 4.09 4.09 4.07 4.07 4.06 4.05 4.04 1.40 1.39 1.38 1.38 1.37 1.37 1.18 1.16 1.15 0.98 0.96 0.96 0.94
Me
F
CO2Et
1H-NMR (400MHz, CDCl3)
3t
1.97 2.01 1.30 1.00 1.01 2.06 1.08 0.96 2.98 3.23 3.13
9.5 9.0 8.5 8.0 7.5 7.0 6.5 6.0 5.5 5.0 4.5 4.0 3.5 3.0 2.5 2.0 1.5 1.0 0.5 0.0
f1 (ppm)

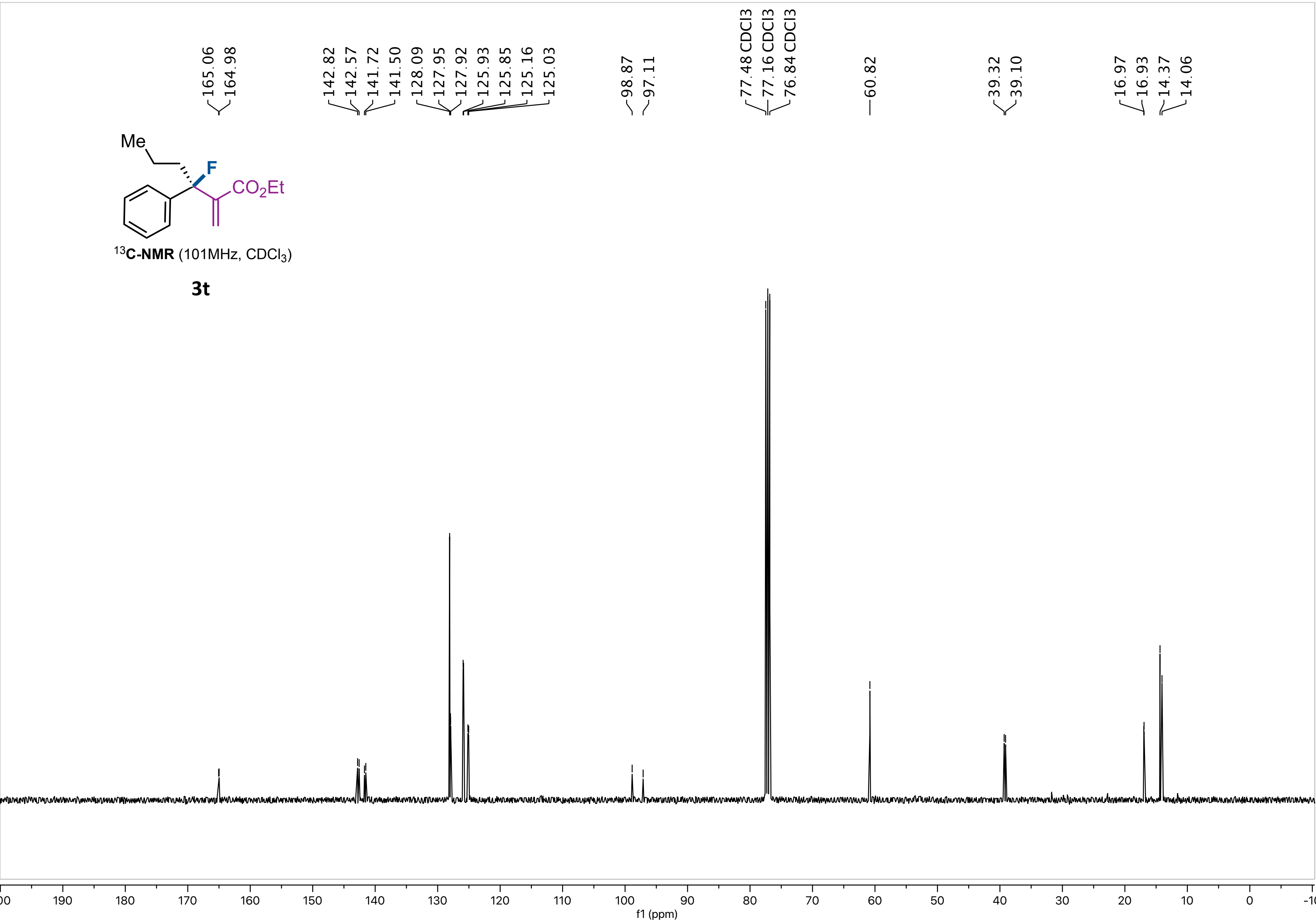
165.06
164.98
142.82
142.57
141.72
141.50
128.09
127.95
127.92
125.93
125.85
125.16
125.03
98.87
97.11
77.48 CDCl3
77.16 CDCl3
76.84 CDCl3
60.82
39.32
39.10
16.97
16.93
14.37
14.06
Me
F
CO2Et
13C-NMR (101MHz, CDCl3)
3t
f1 (ppm)

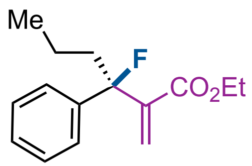

^{19}**F-NMR** (376MHz, $CDCl_3$)

3t

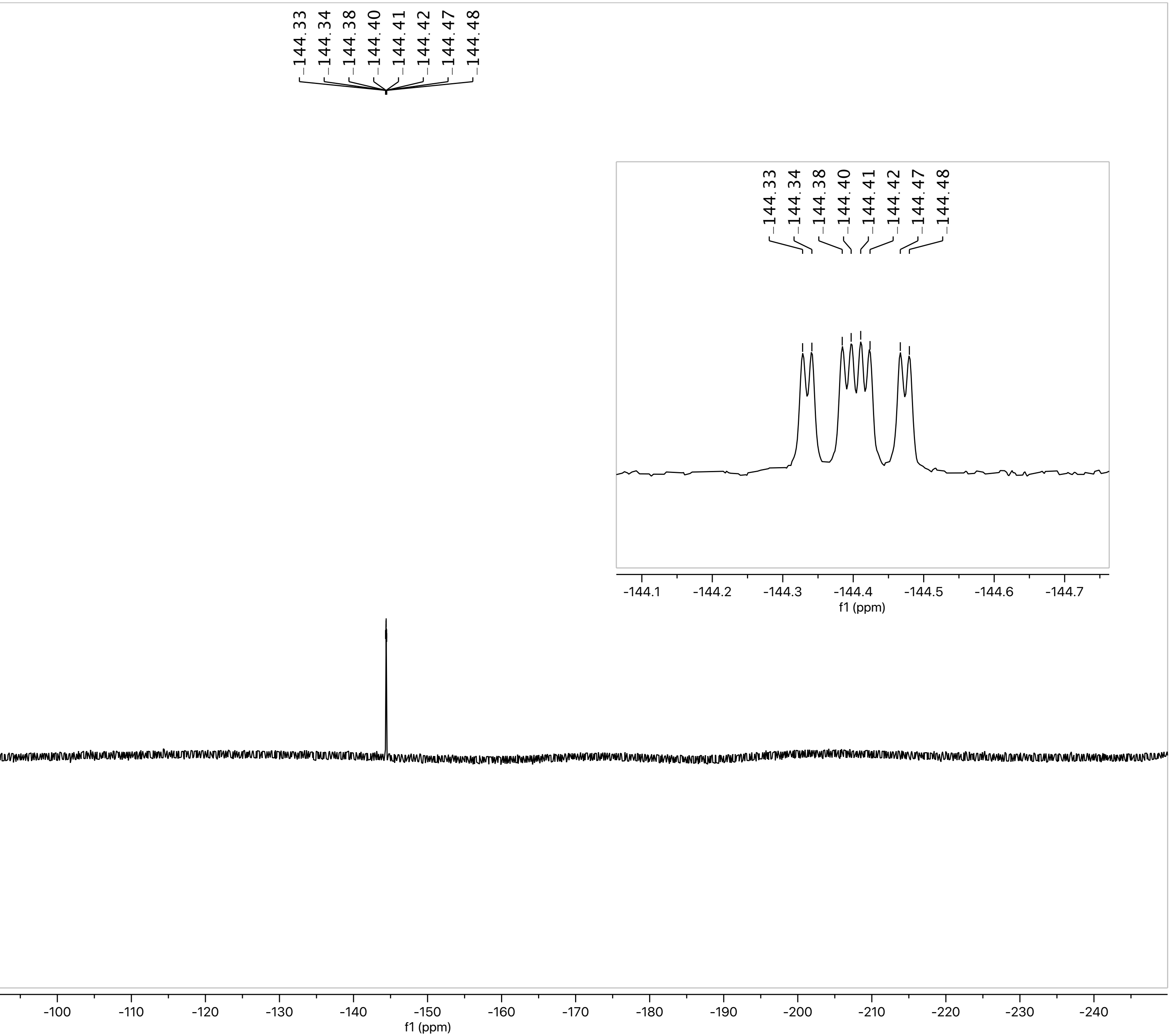

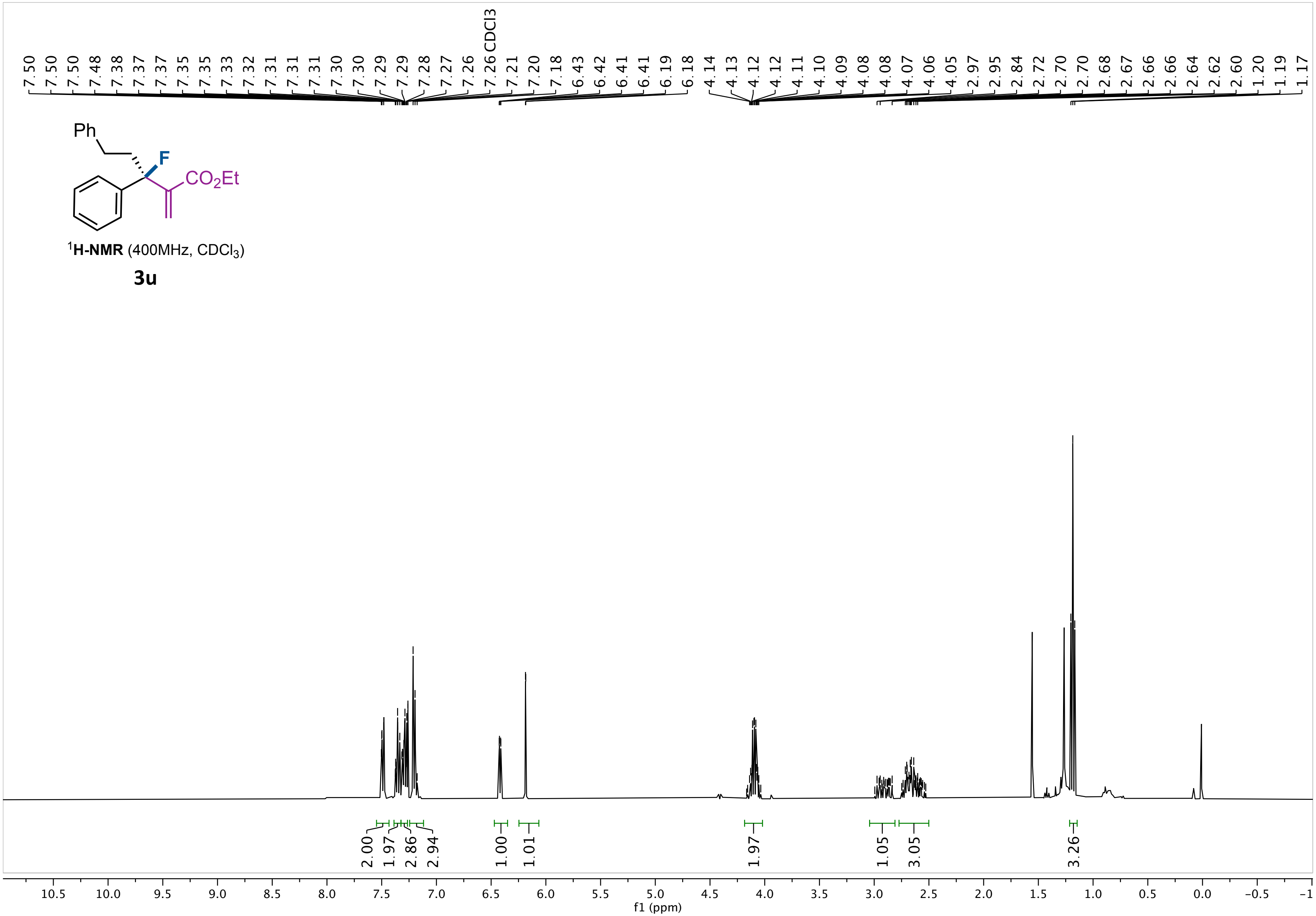

Ph
F
CO_2Et
^{1}H-NMR (400MHz, $CDCl_3$)
3u
CDCl3
f1 (ppm)

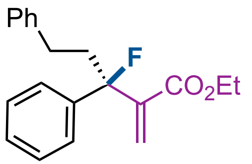

[1]**H-NMR** (400MHz, $CDCl_3$)
3u

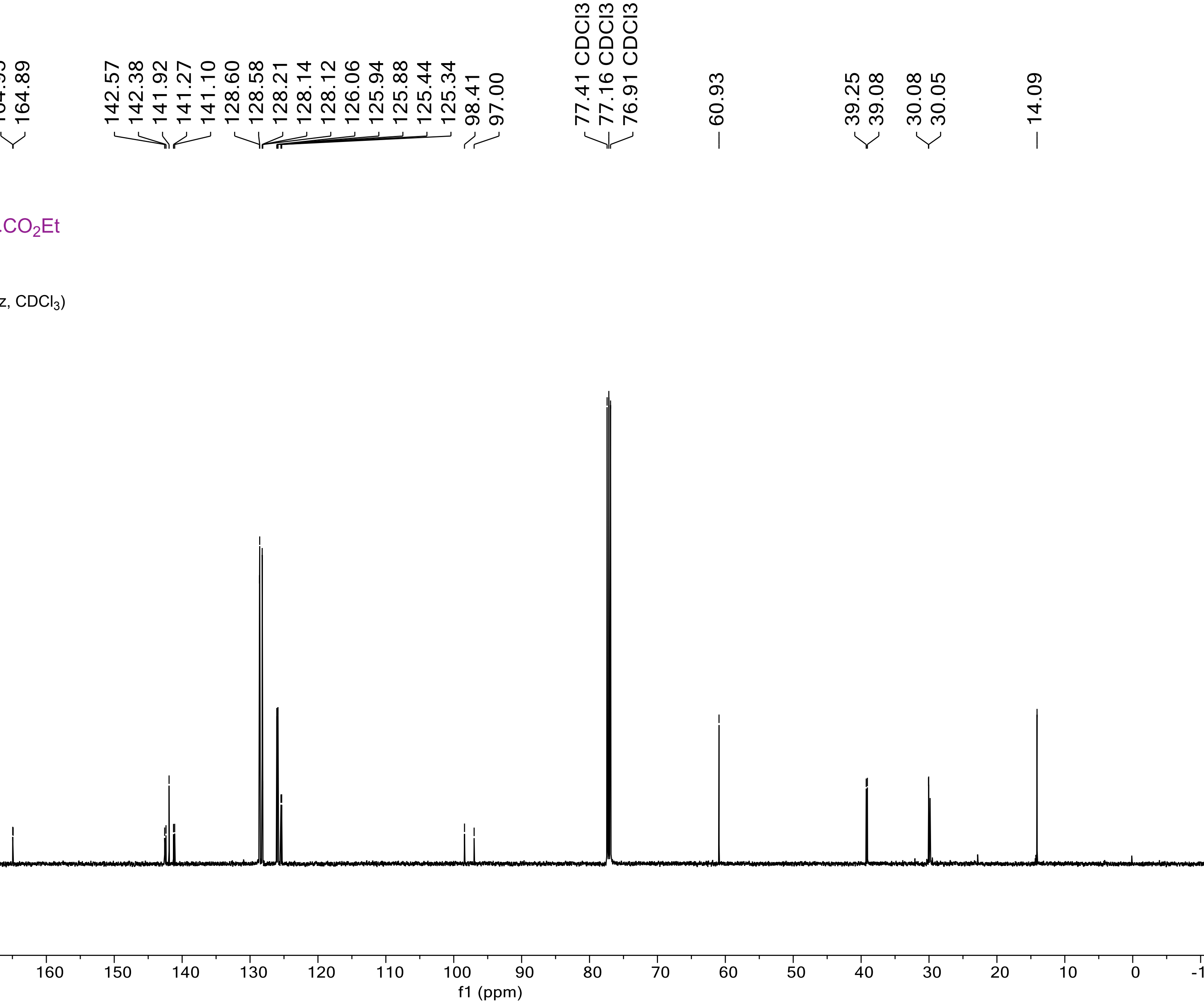

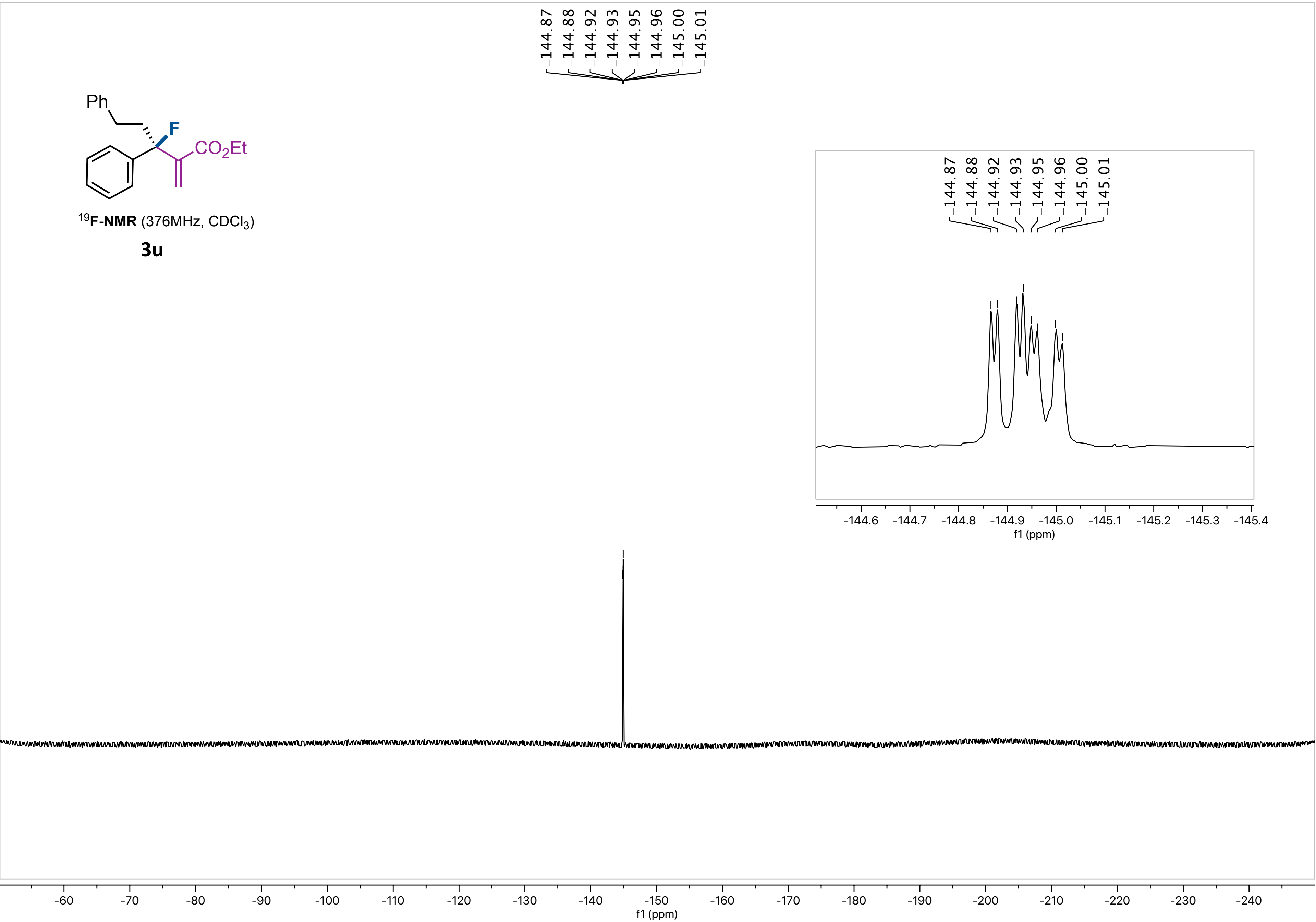
-144.87
-144.88
-144.92
-144.93
-144.95
-144.96
-145.00
-145.01
Ph
F
CO_2Et
^{19}F-NMR (376MHz, $CDCl_3$)
3u
-144.6 -144.7 -144.8 -144.9 -145.0 -145.1 -145.2 -145.3 -145.4
f1 (ppm)
-60 -70 -80 -90 -100 -110 -120 -130 -140 -150 -160 -170 -180 -190 -200 -210 -220 -230 -240
f1 (ppm)

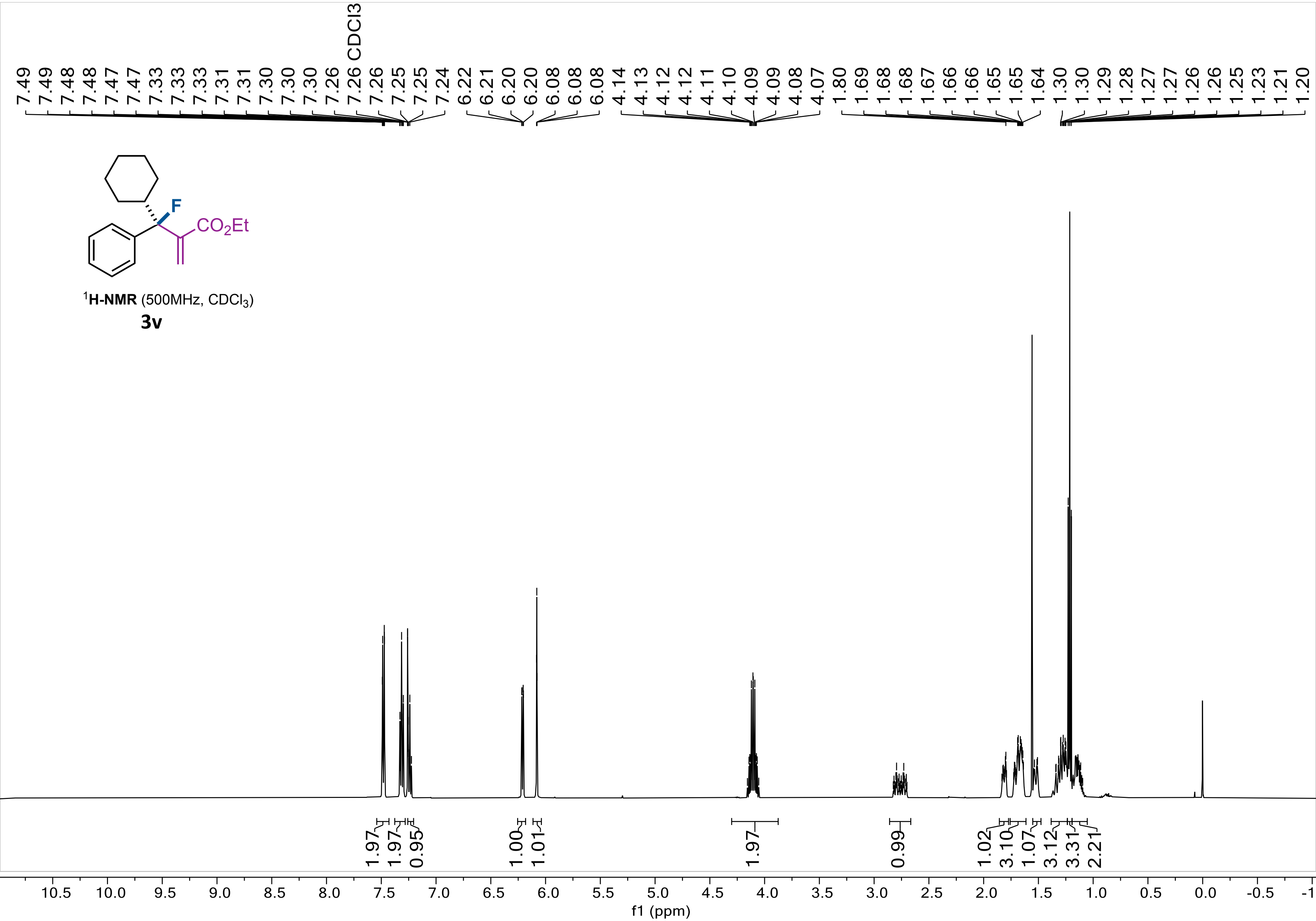
7.49 7.49 7.48 7.48 7.47 7.47 7.33 7.33 7.33 7.31 7.31 7.30 7.30 7.30 7.26 7.26 7.26 7.25 7.25 7.24 6.22 6.21 6.20 6.20 6.08 6.08 6.08 4.14 4.13 4.12 4.12 4.11 4.10 4.09 4.09 4.08 4.07 1.80 1.69 1.68 1.68 1.67 1.66 1.66 1.65 1.65 1.64 1.30 1.30 1.29 1.28 1.27 1.27 1.26 1.26 1.25 1.23 1.21 1.20
CDCl3
F
CO2Et
1H-NMR (500MHz, CDCl3)
3v
1.97 1.97 0.95 1.00 1.01 1.97 0.99 1.02 3.10 1.07 3.12 3.31 2.21
10.5 10.0 9.5 9.0 8.5 8.0 7.5 7.0 6.5 6.0 5.5 5.0 4.5 4.0 3.5 3.0 2.5 2.0 1.5 1.0 0.5 0.0 -0.5 -1
f1 (ppm)

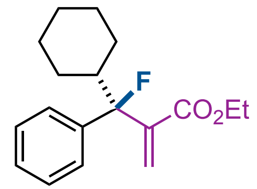

^{13}**C-NMR** (126MHz, $CDCl_3$)

3v

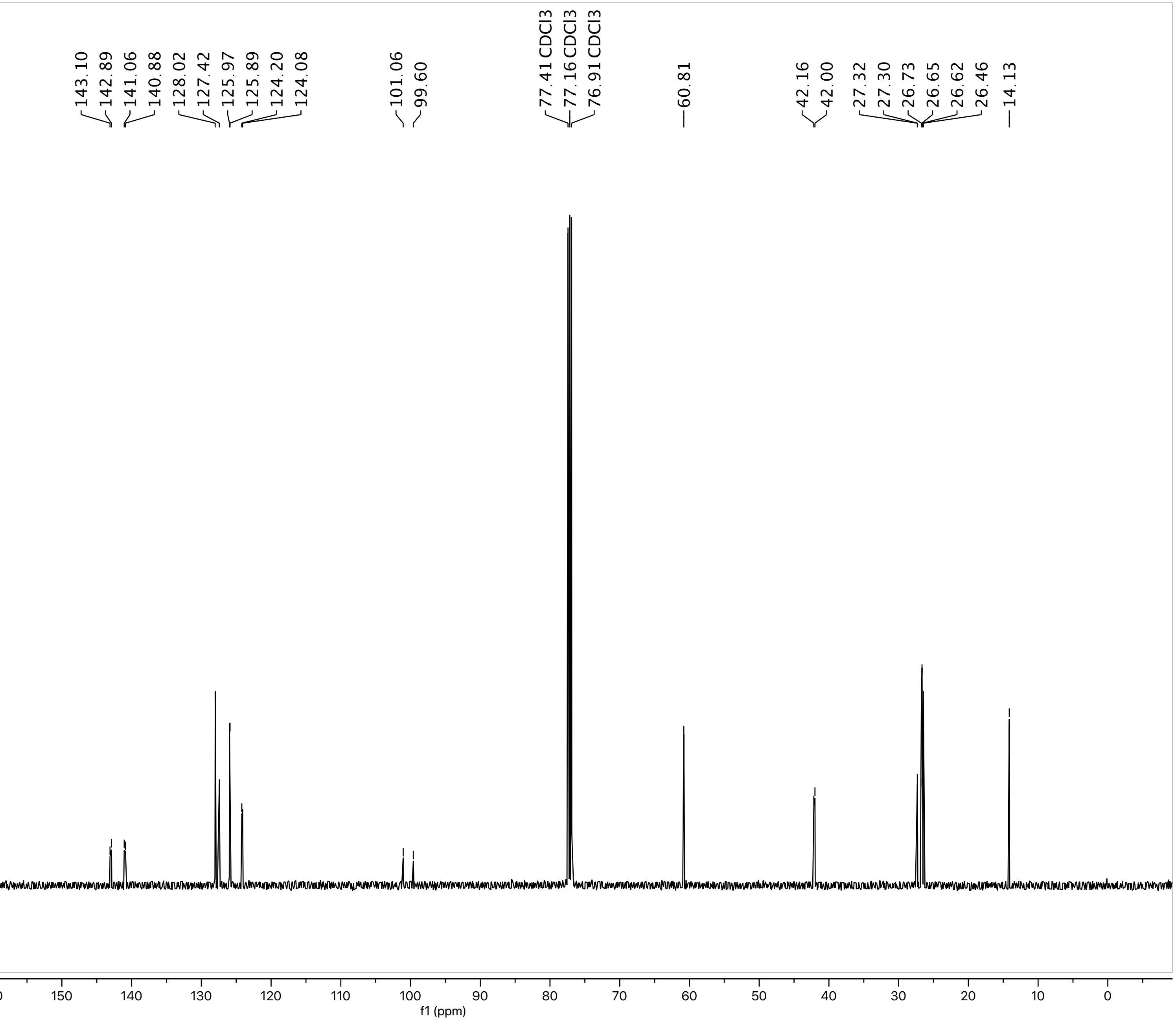

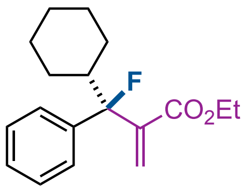

^{19}**F-NMR** (376MHz, $CDCl_3$)

3v

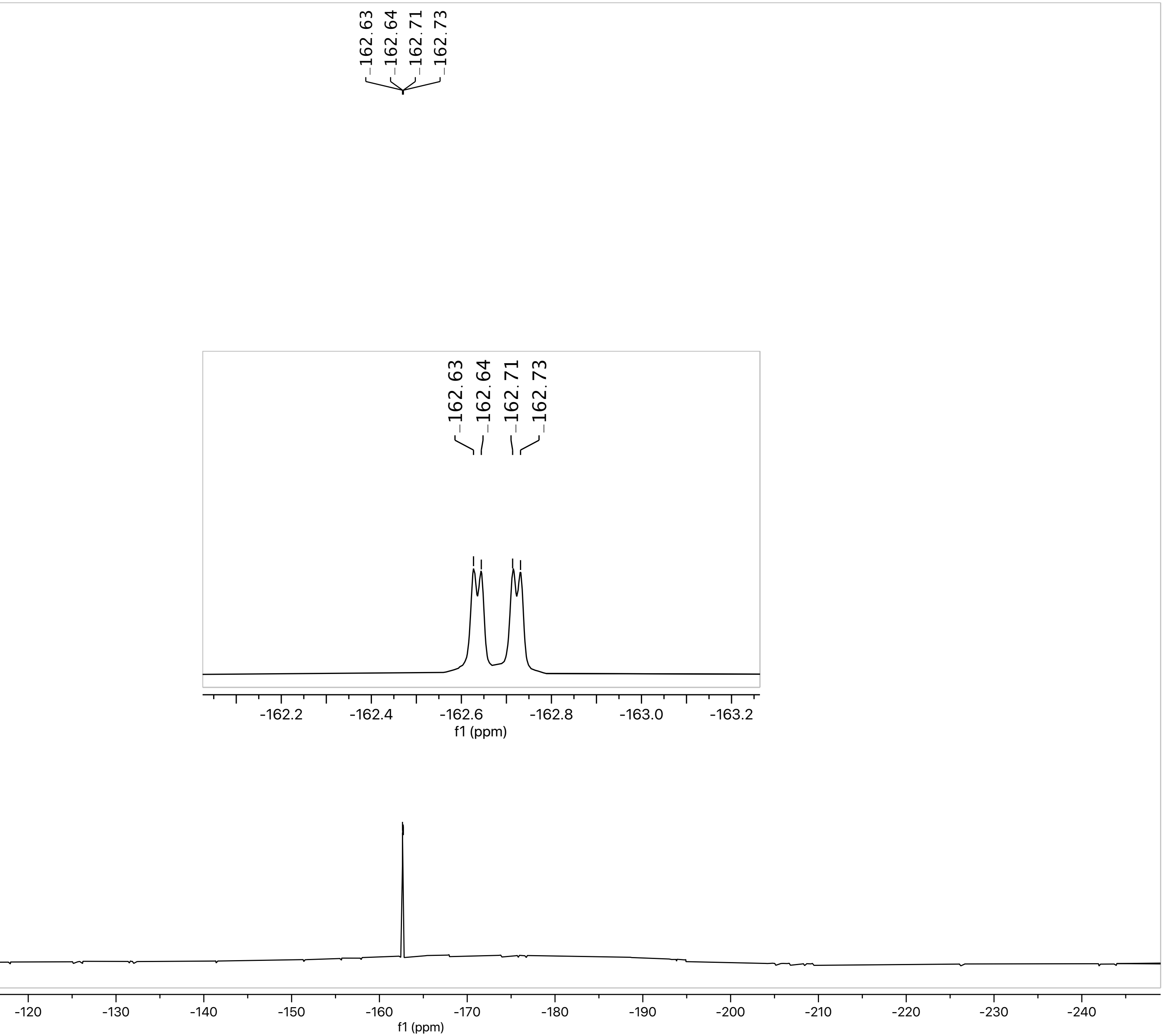

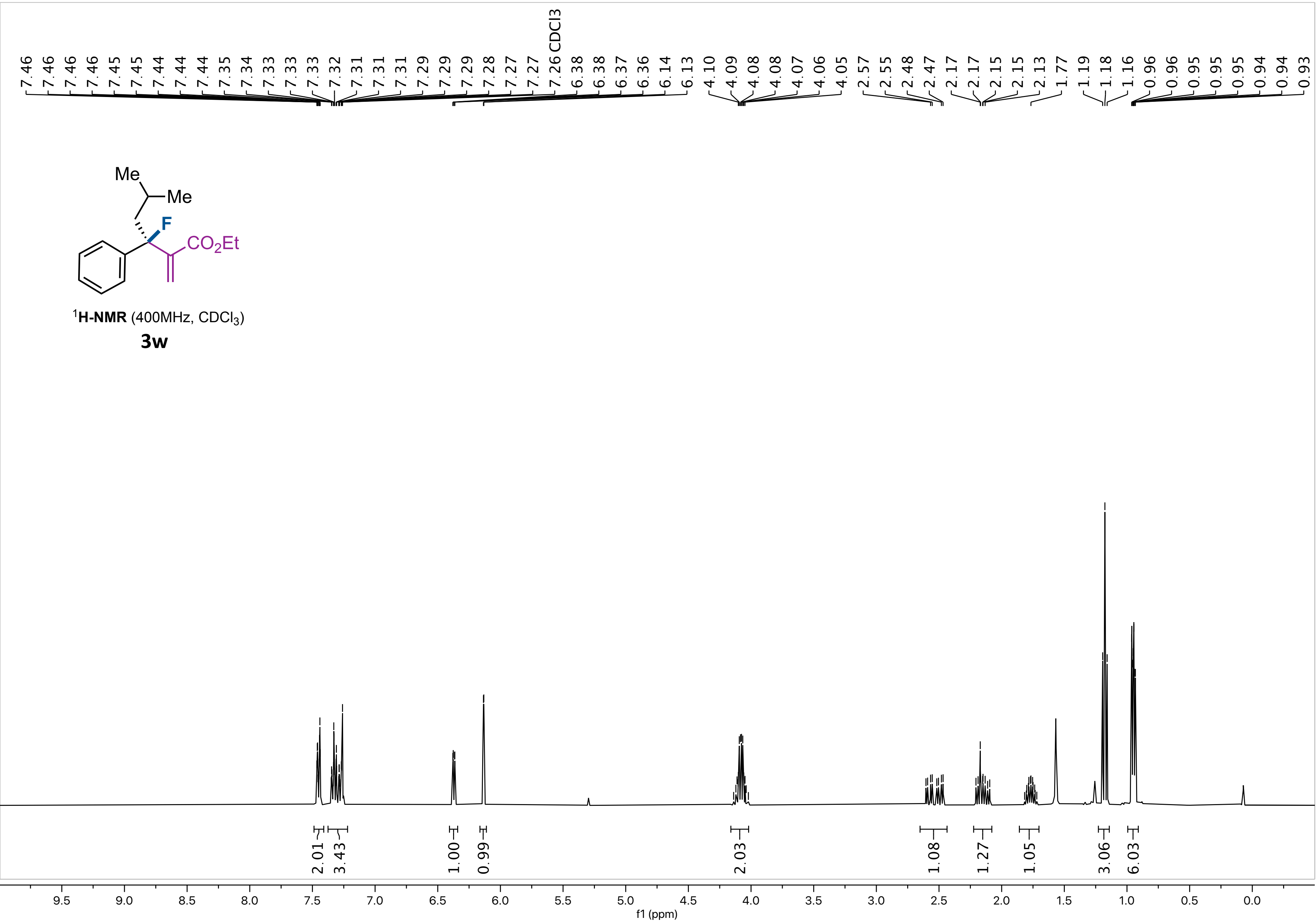
7.46 7.46 7.46 7.46 7.45 7.45 7.44 7.44 7.44 7.35 7.34 7.33 7.33 7.33 7.32 7.31 7.31 7.31 7.29 7.29 7.29 7.28 7.27 7.27 7.26 CDCl3 6.38 6.38 6.37 6.36 6.14 6.13 4.10 4.09 4.08 4.08 4.07 4.06 4.05 2.57 2.55 2.48 2.47 2.17 2.17 2.15 2.15 2.13 1.77 1.19 1.18 1.16 0.96 0.96 0.95 0.95 0.95 0.94 0.94 0.93
Me
Me
F
CO_2Et
^{1}H-NMR (400MHz, $CDCl_3$)
3w
2.01 3.43 1.00 0.99 2.03 1.08 1.27 1.05 3.06 6.03
9.5 9.0 8.5 8.0 7.5 7.0 6.5 6.0 5.5 5.0 4.5 4.0 3.5 3.0 2.5 2.0 1.5 1.0 0.5 0.0
f1 (ppm)

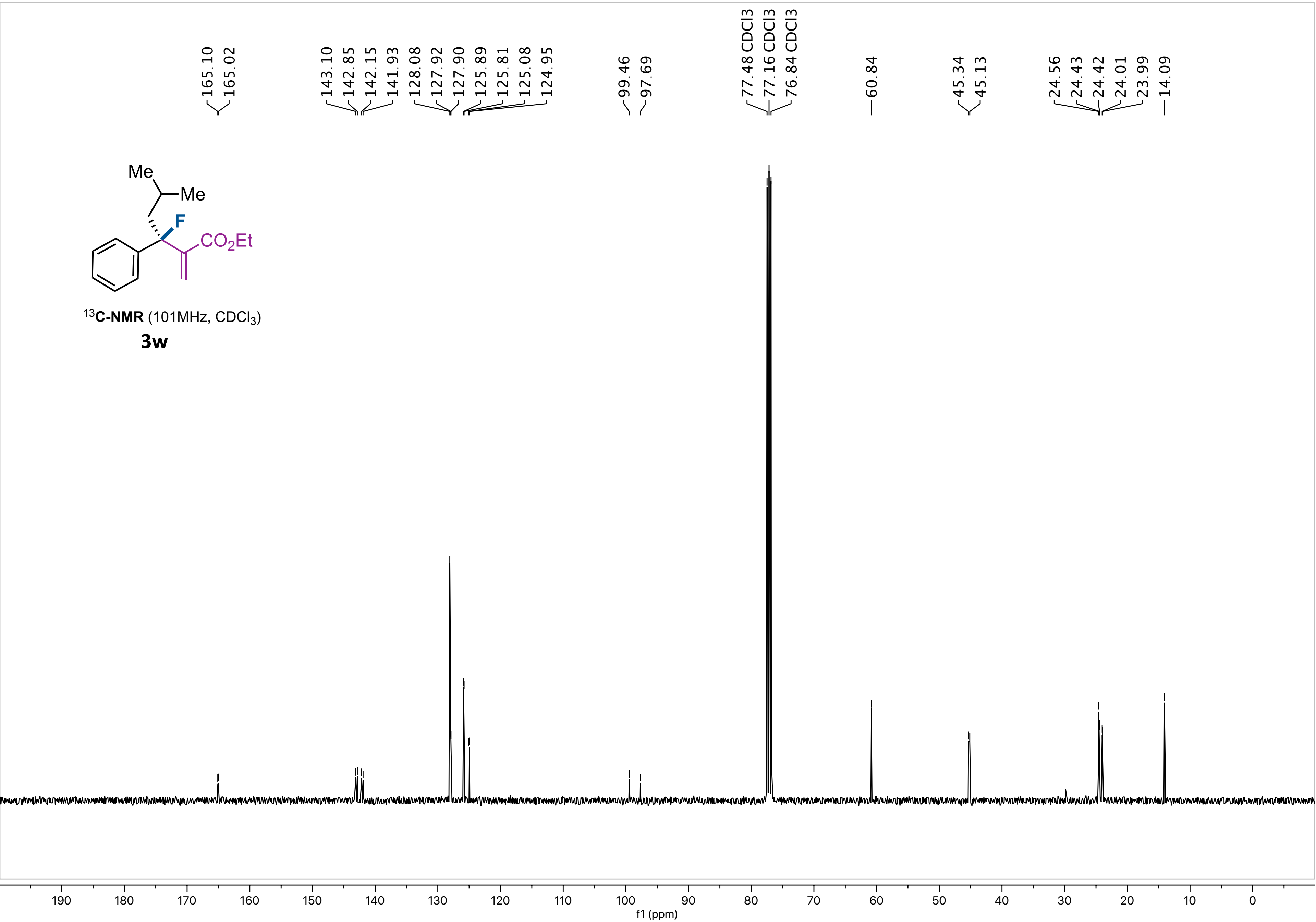
165.10
165.02
143.10
142.85
142.15
141.93
128.08
127.92
127.90
125.89
125.81
125.08
124.95
99.46
97.69
77.48 CDCl3
77.16 CDCl3
76.84 CDCl3
60.84
45.34
45.13
24.56
24.43
24.42
24.01
23.99
14.09
Me
Me
F
CO2Et
13C-NMR (101MHz, CDCl3)
3w
190
180
170
160
150
140
130
120
110
100
90
80
70
60
50
40
30
20
10
0
f1 (ppm)

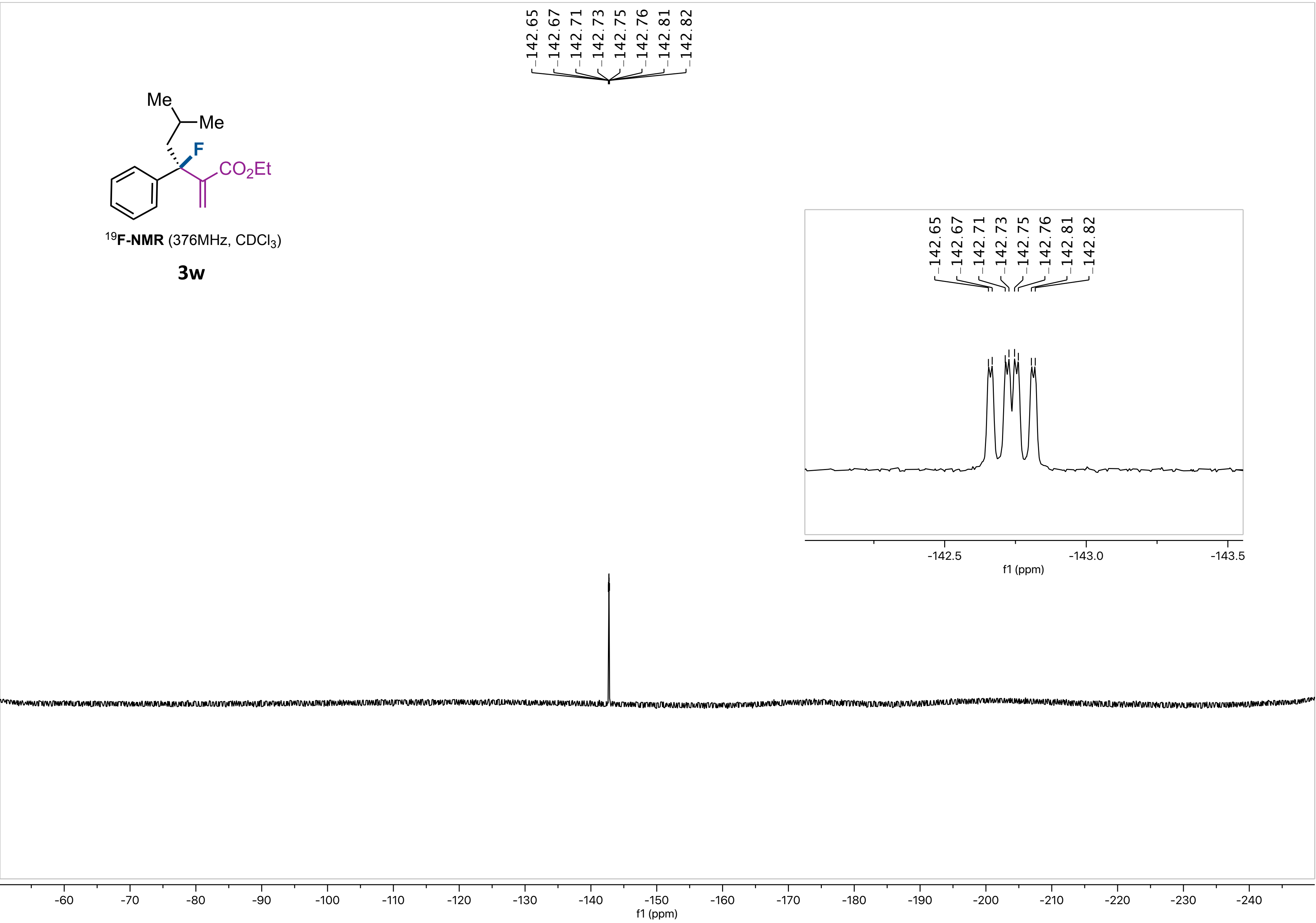

-142.65
-142.67
-142.71
-142.73
-142.75
-142.76
-142.81
-142.82
Me
Me
F
CO2Et
19F-NMR (376MHz, CDCl3)
3w
-142.5
-143.0
-143.5
f1 (ppm)
-60
-70
-80
-90
-100
-110
-120
-130
-140
-150
-160
-170
-180
-190
-200
-210
-220
-230
-240
f1 (ppm)

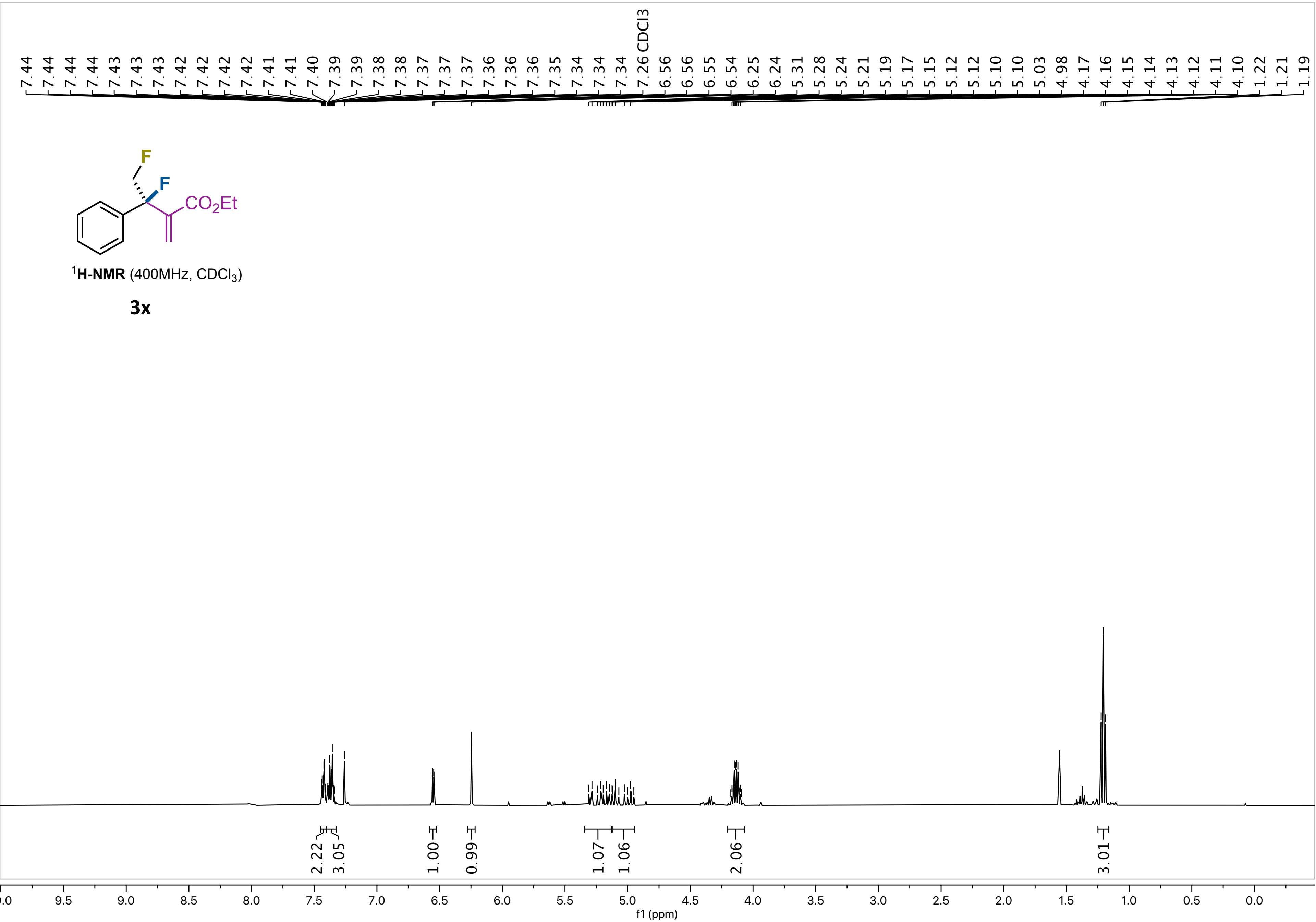

^{1}**H-NMR** (400MHz, $CDCl_3$)

3x

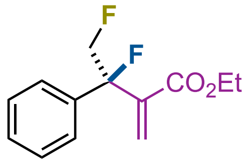

^{13}**C-NMR** (101MHz, $CDCl_3$)

3x

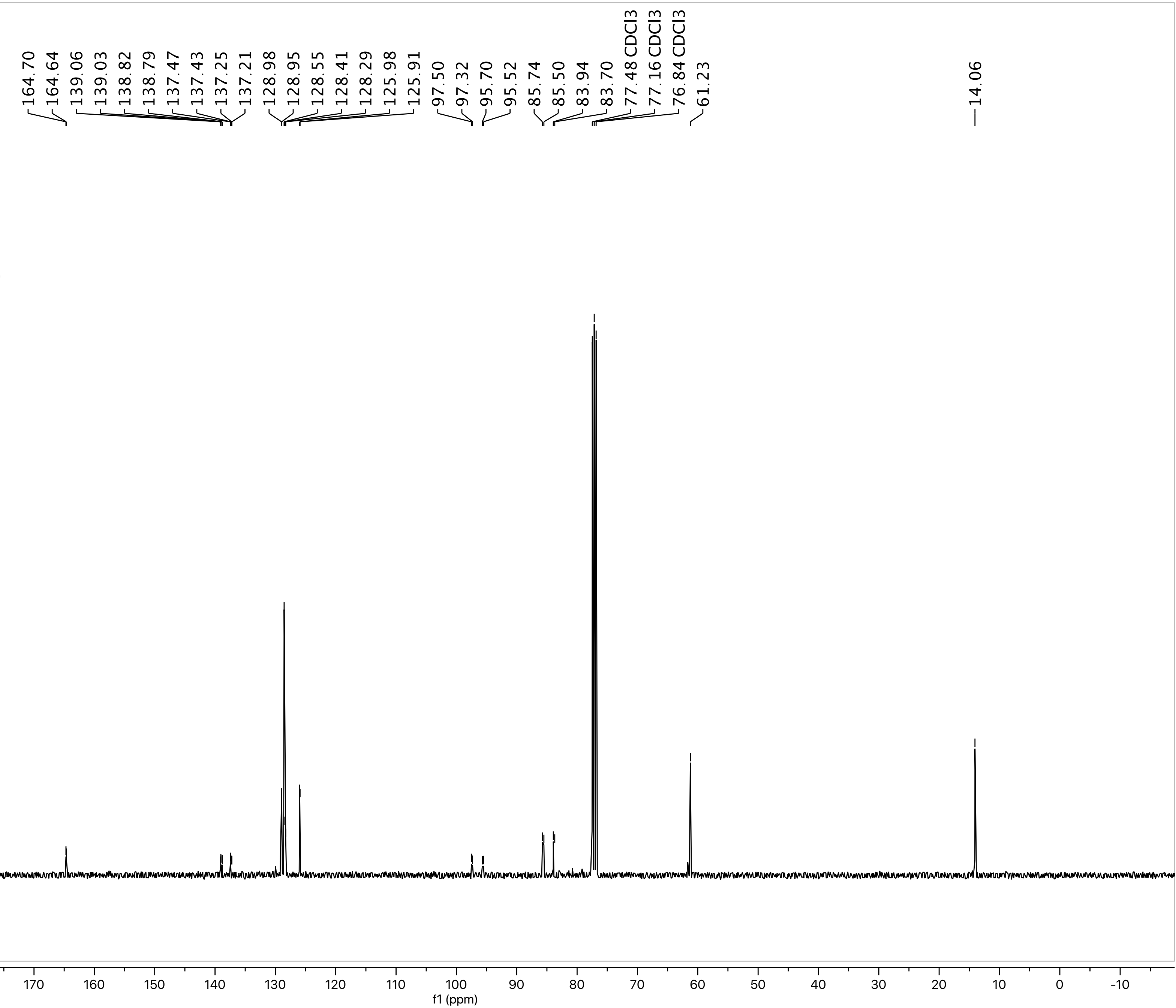

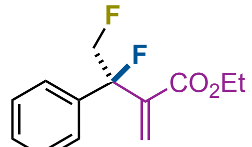

^{19}**F-NMR** (376MHz, $CDCl_3$)

3x

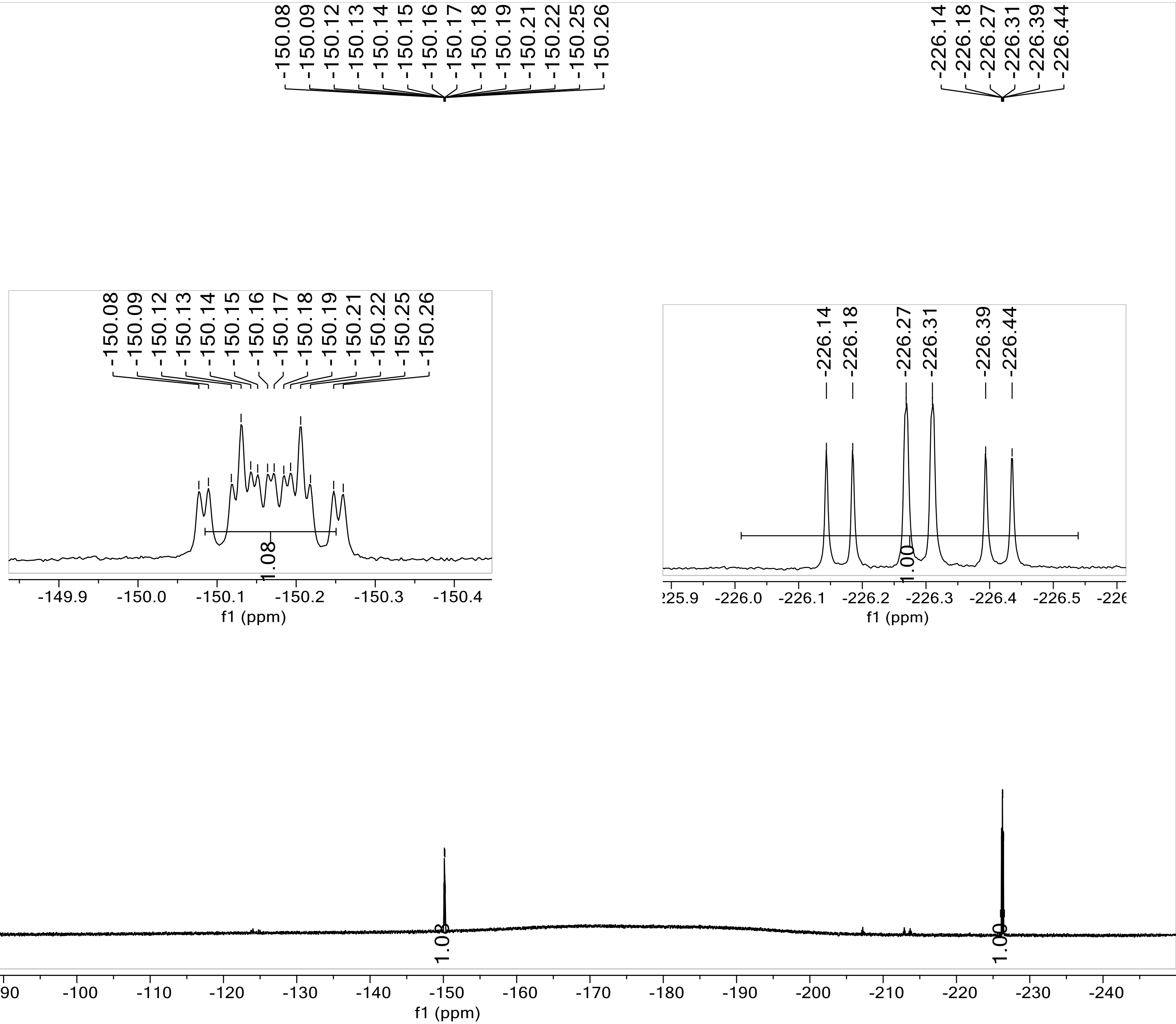

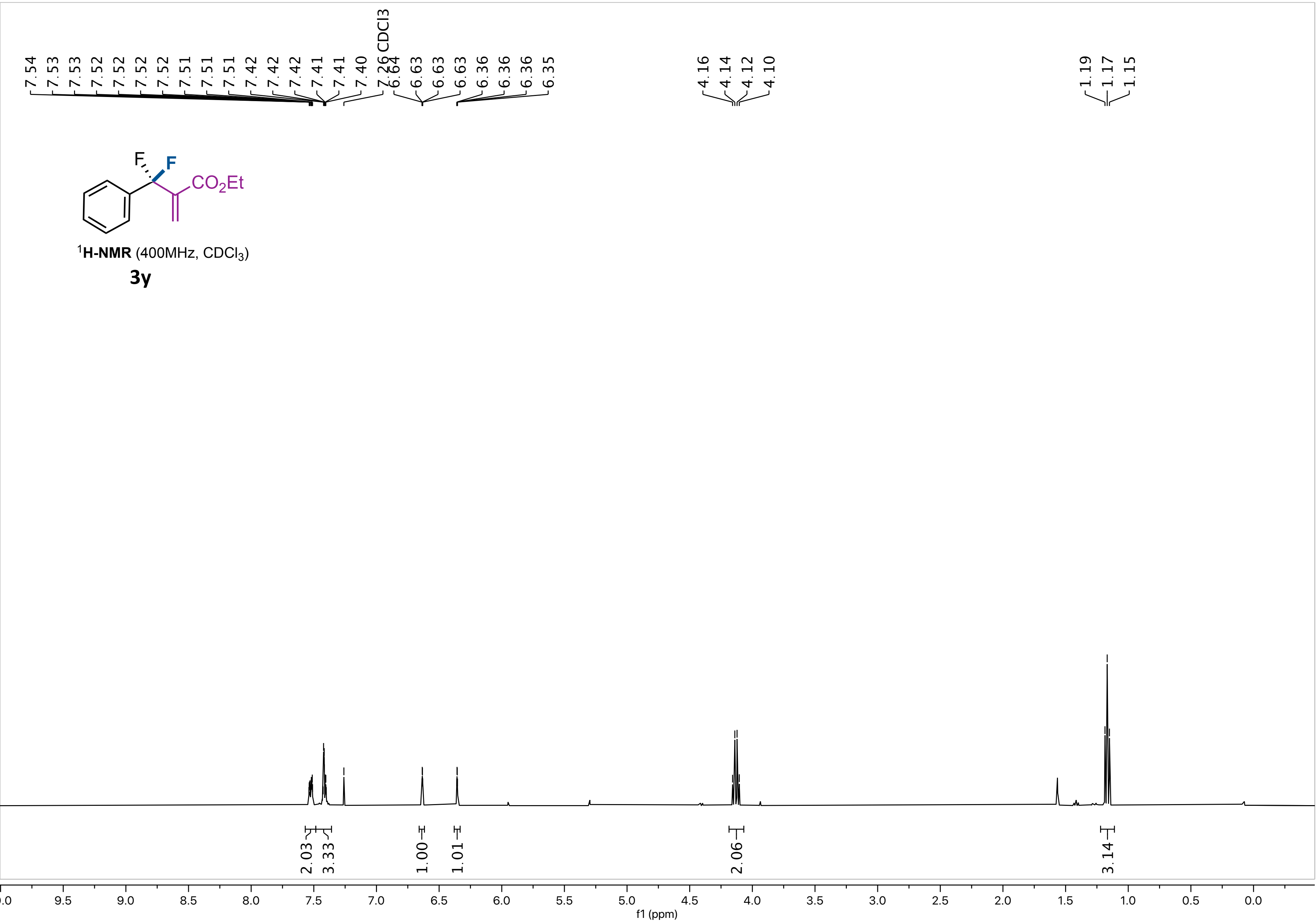
7.54
7.53
7.53
7.52
7.52
7.52
7.52
7.51
7.51
7.51
7.42
7.42
7.42
7.41
7.41
7.40
7.26 CDCl3
6.64
6.63
6.63
6.63
6.36
6.36
6.36
6.35
4.16
4.14
4.12
4.10
1.19
1.17
1.15
F
F
CO2Et
1H-NMR (400MHz, CDCl3)
3y
2.03
3.33
1.00
1.01
2.06
3.14
9.5
9.0
8.5
8.0
7.5
7.0
6.5
6.0
5.5
5.0
4.5
4.0
3.5
3.0
2.5
2.0
1.5
1.0
0.5
0.0
f1 (ppm)

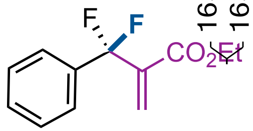

^{13}**C-NMR** (101MHz, $CDCl_3$)

3y

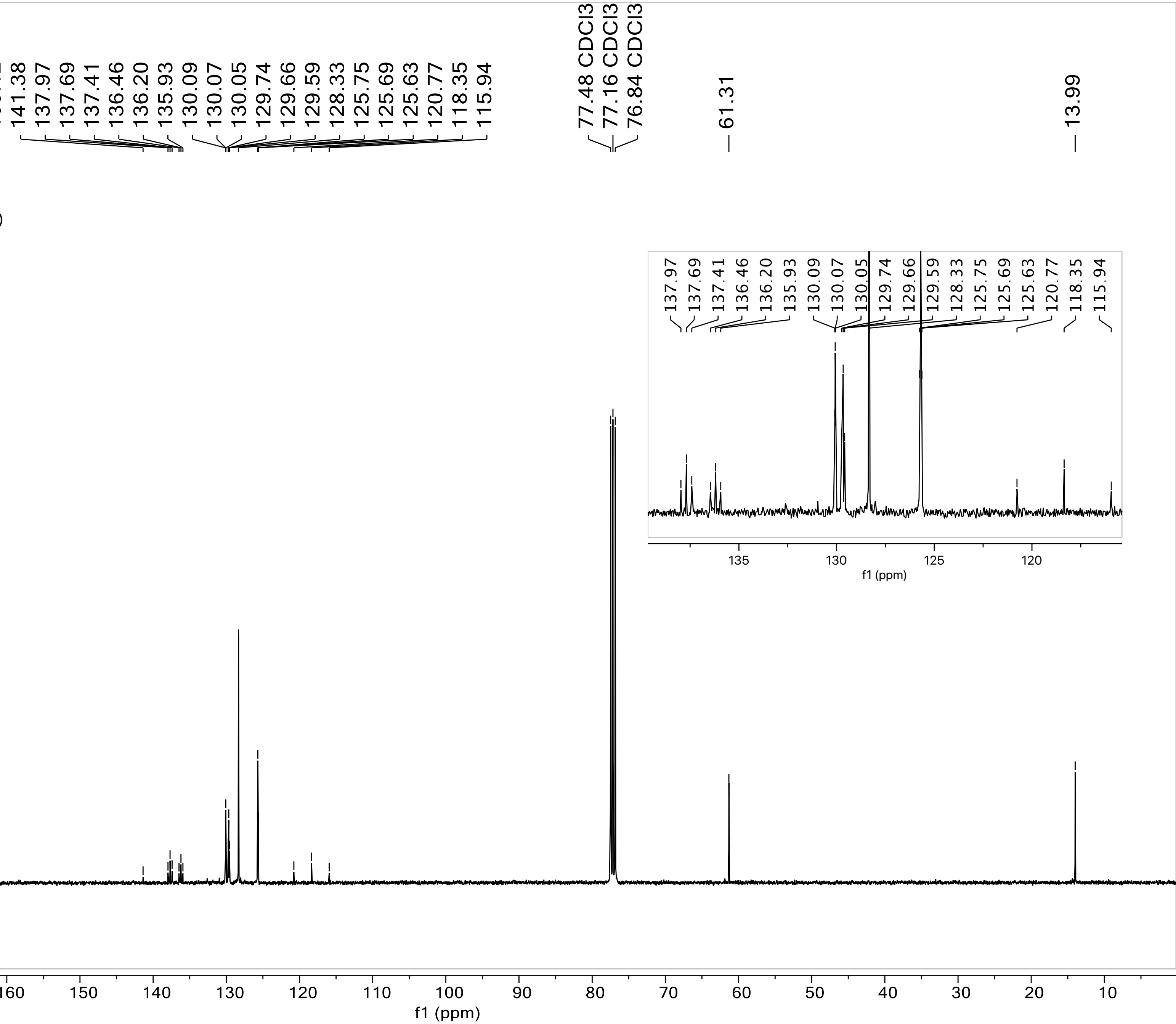

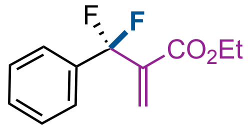

^{19}**F-NMR** (376MHz, $CDCl_3$)

3y

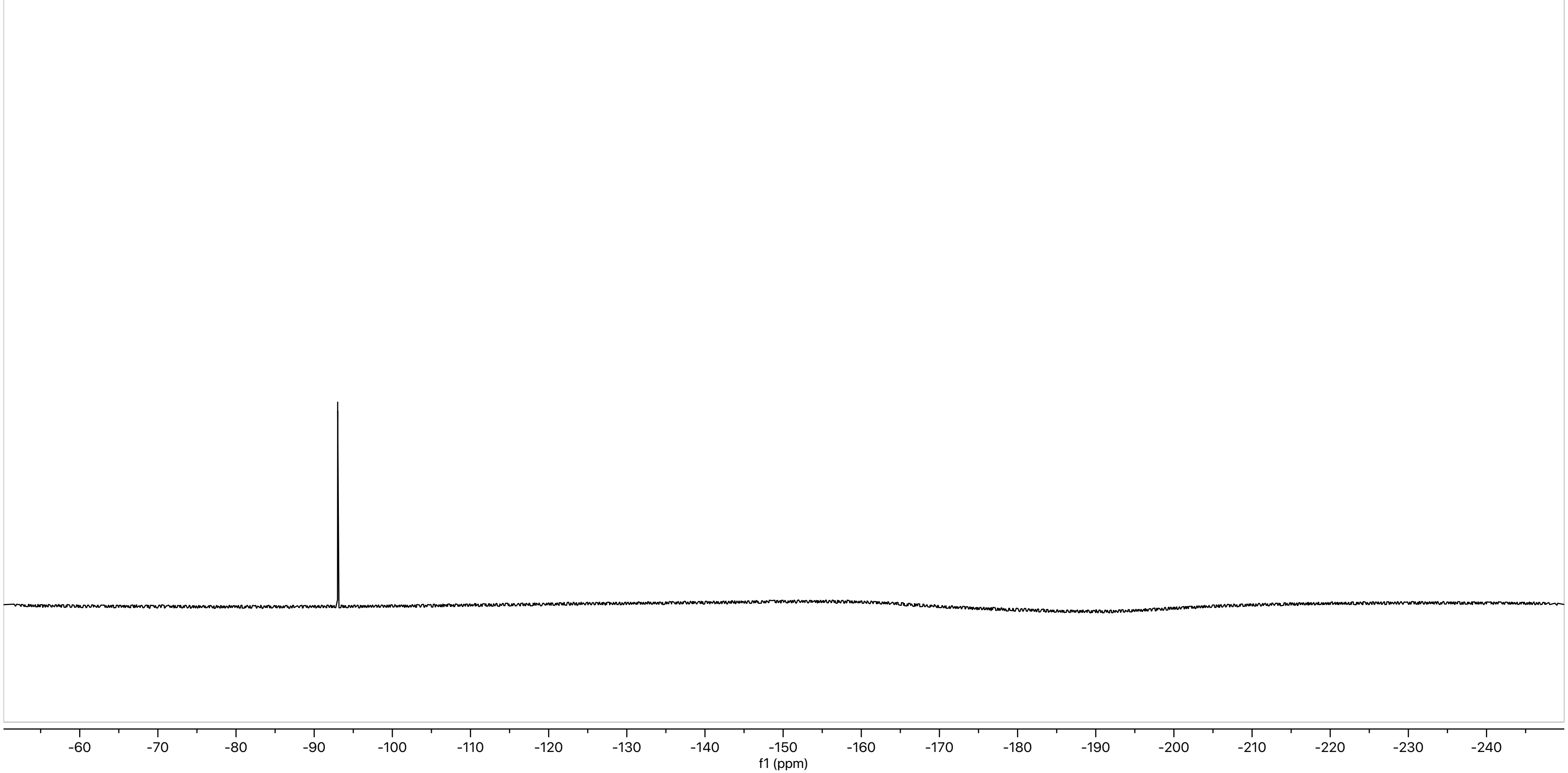

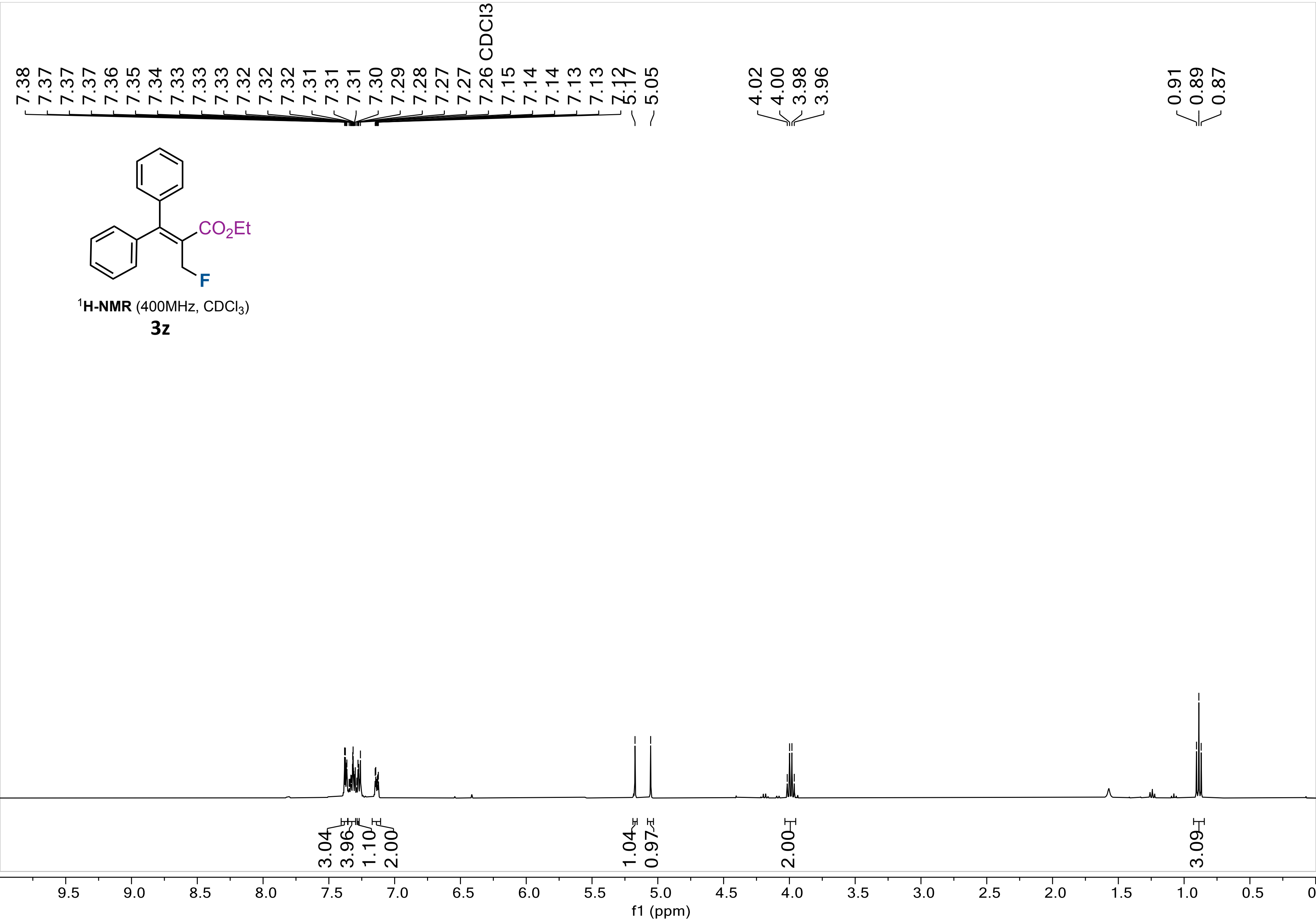
7.38
7.37
7.37
7.37
7.36
7.35
7.34
7.33
7.33
7.33
7.32
7.32
7.32
7.31
7.31
7.31
7.30
7.29
7.28
7.27
7.27
7.26 CDCl3
7.15
7.14
7.14
7.13
7.13
7.12
5.17
5.05
4.02
4.00
3.98
3.96
0.91
0.89
0.87
CO2Et
F
1H-NMR (400MHz, CDCl3)
3z
3.04
3.96
1.10
2.00
1.04
0.97
2.00
3.09
9.5
9.0
8.5
8.0
7.5
7.0
6.5
6.0
5.5
5.0
4.5
4.0
3.5
3.0
2.5
2.0
1.5
1.0
0.5
0
f1 (ppm)

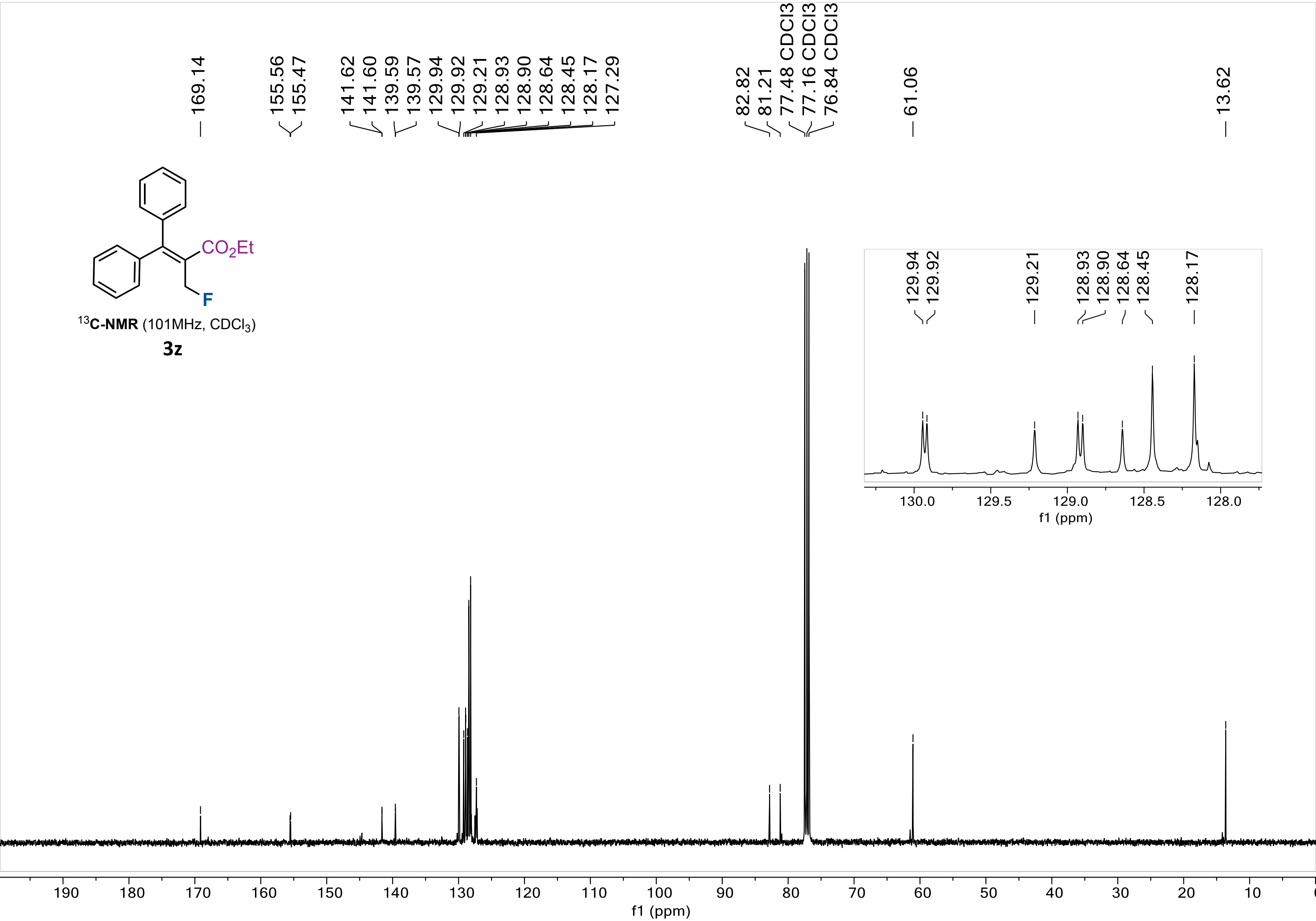

^{13}**C-NMR** (101MHz, $CDCl_3$)

3z

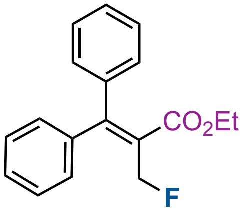

^{19}**F-NMR** (376MHz, $CDCl_3$)

3z

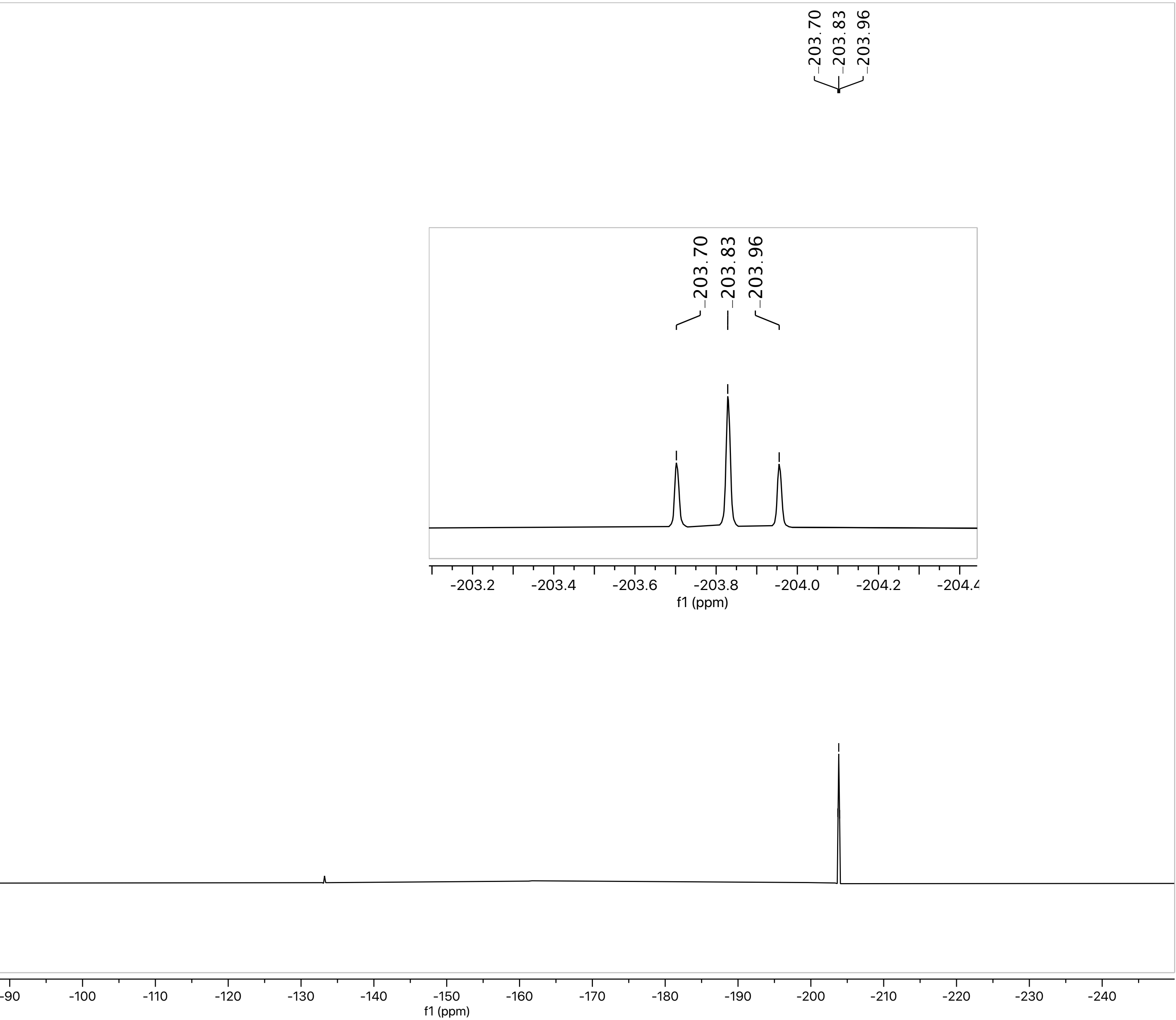

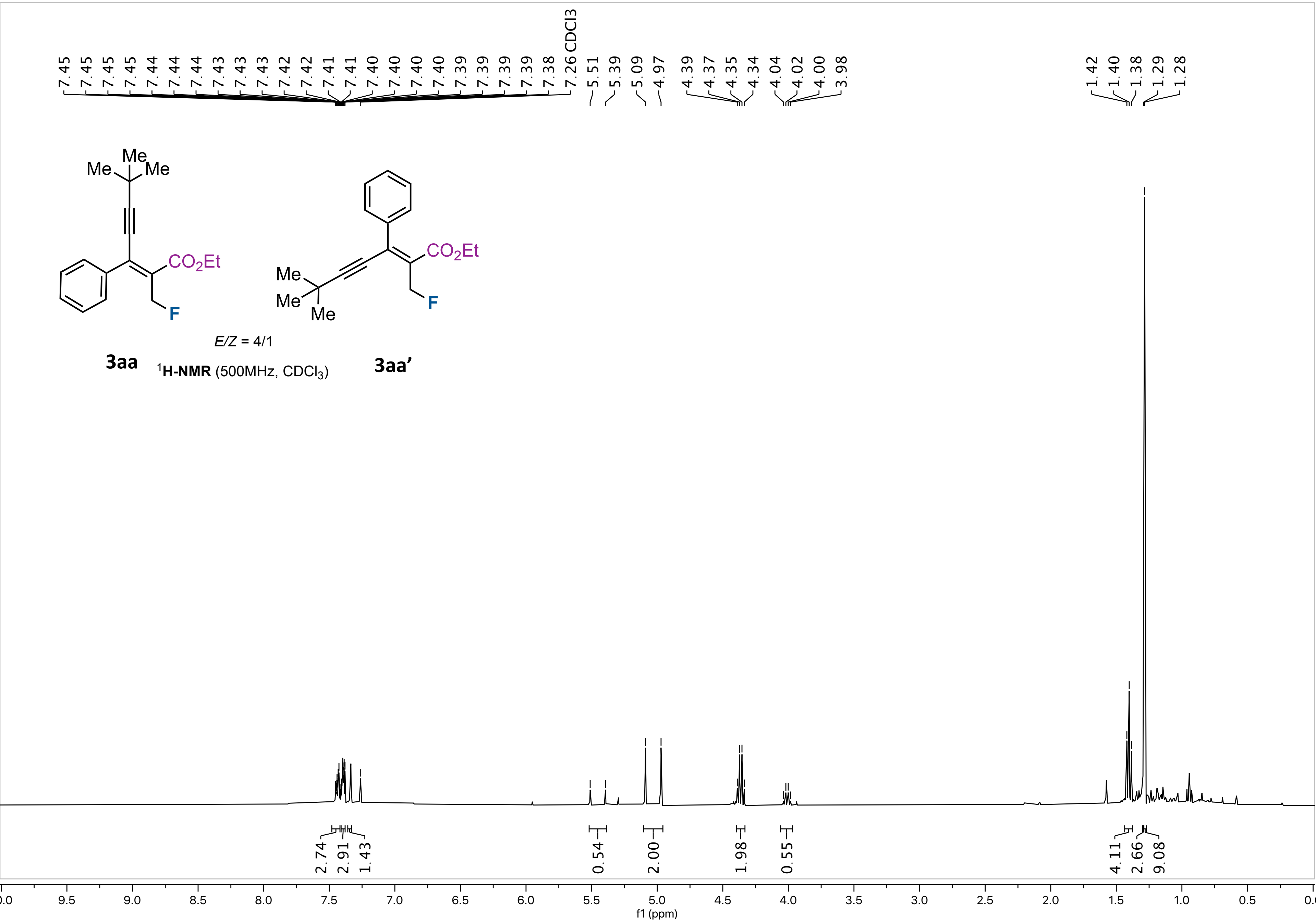

7.45 7.45 7.45 7.45 7.44 7.44 7.44 7.43 7.43 7.43 7.42 7.42 7.41 7.41 7.40 7.40 7.40 7.40 7.39 7.39 7.39 7.39 7.38 7.26 CDCl3 5.51 5.39 5.09 4.97 4.39 4.37 4.35 4.34 4.04 4.02 4.00 3.98 1.42 1.40 1.38 1.29 1.28
Me
Me
Me
CO2Et
F
Me
Me
Me
CO2Et
F
E/Z = 4/1
3aa
1H-NMR (500MHz, CDCl3)
3aa'
2.74 2.91 1.43 0.54 2.00 1.98 0.55 4.11 2.66 9.08
10.0 9.5 9.0 8.5 8.0 7.5 7.0 6.5 6.0 5.5 5.0 4.5 4.0 3.5 3.0 2.5 2.0 1.5 1.0 0.5 0.0
f1 (ppm)

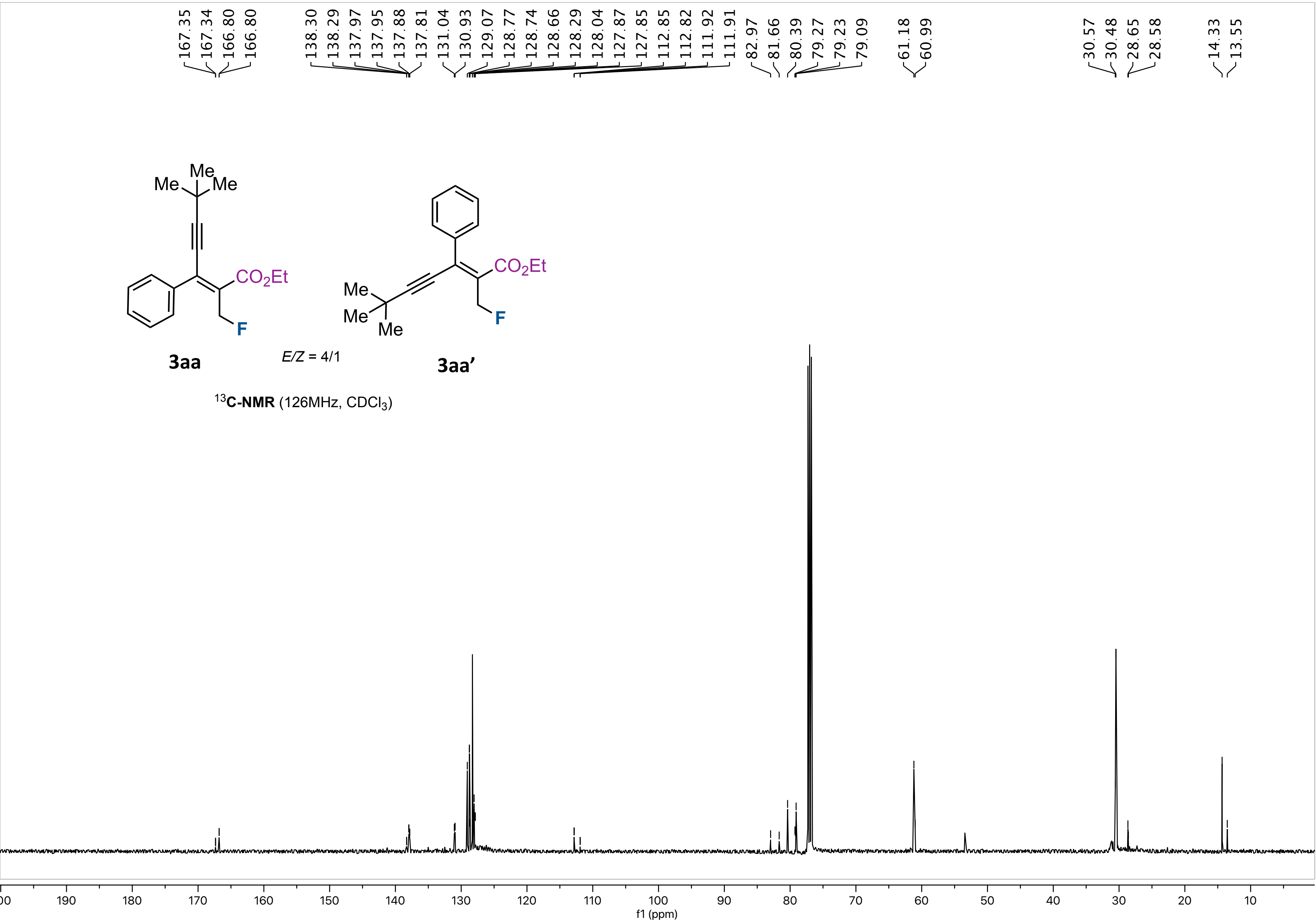
167.35
167.34
166.80
166.80
138.30
138.29
137.97
137.95
137.88
137.81
131.04
130.93
129.07
128.77
128.74
128.66
128.29
128.04
127.87
127.85
112.85
112.82
111.92
111.91
82.97
81.66
80.39
79.27
79.23
79.09
61.18
60.99
30.57
30.48
28.65
28.58
14.33
13.55
Me
Me
Me
CO2Et
F
3aa
E/Z = 4/1
3aa'
13C-NMR (126MHz, CDCl3)
f1 (ppm)

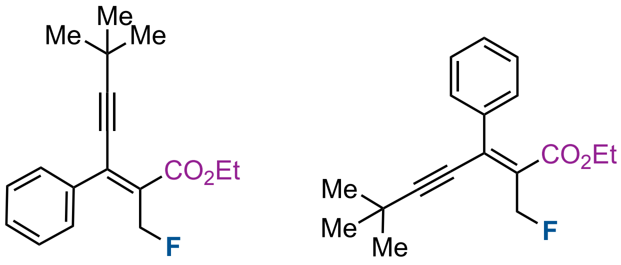

3aa *E/Z* = 4/1 **3aa'**

[19]**F-NMR** (376MHz, $CDCl_3$)

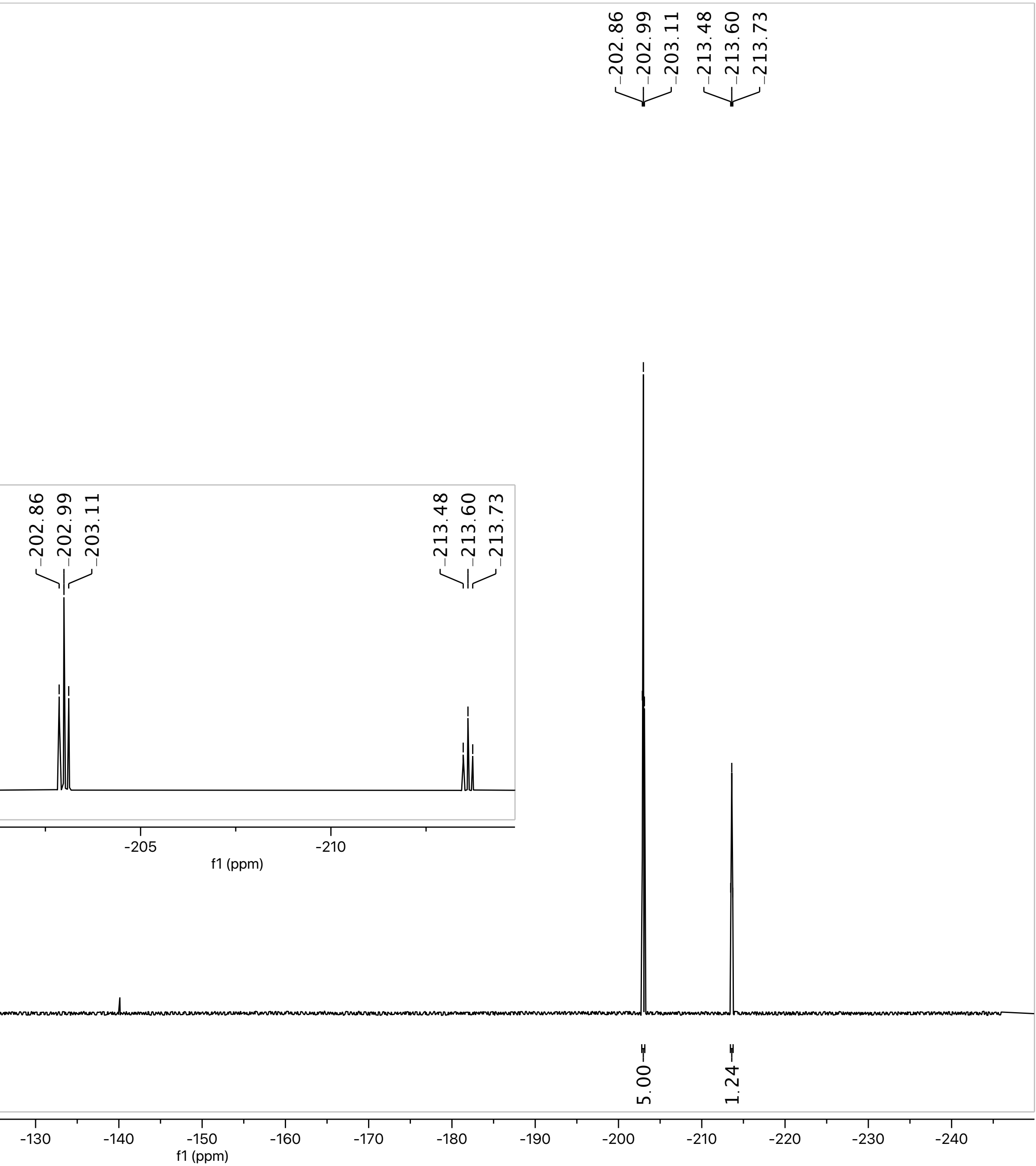

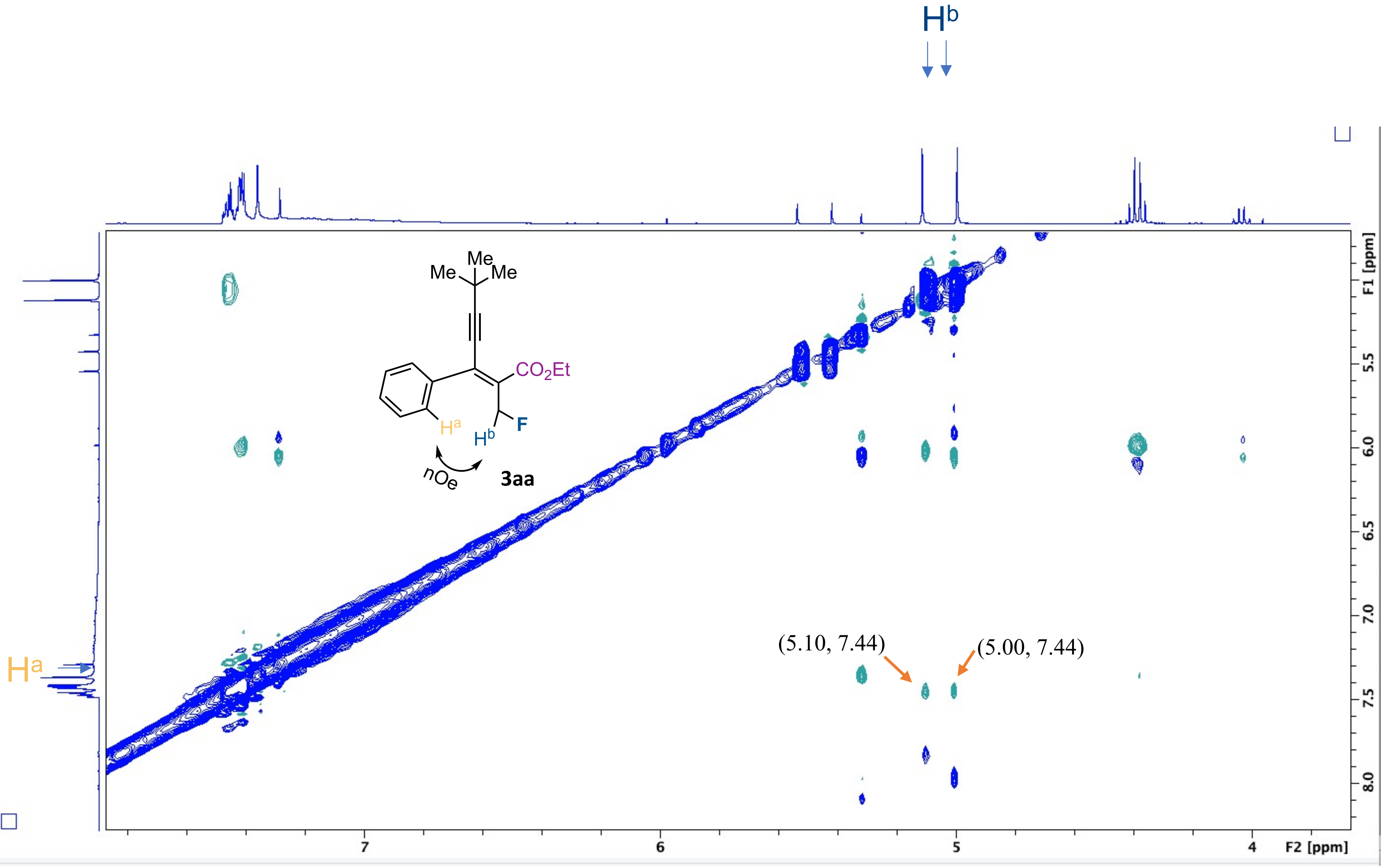
Hb
Ha
Me
Me
Me
CO2Et
F
nOe
3aa
(5.10, 7.44)
(5.00, 7.44)
F1 [ppm]
F2 [ppm]
7
6
5
4
5.5
6.0
6.5
7.0
7.5
8.0

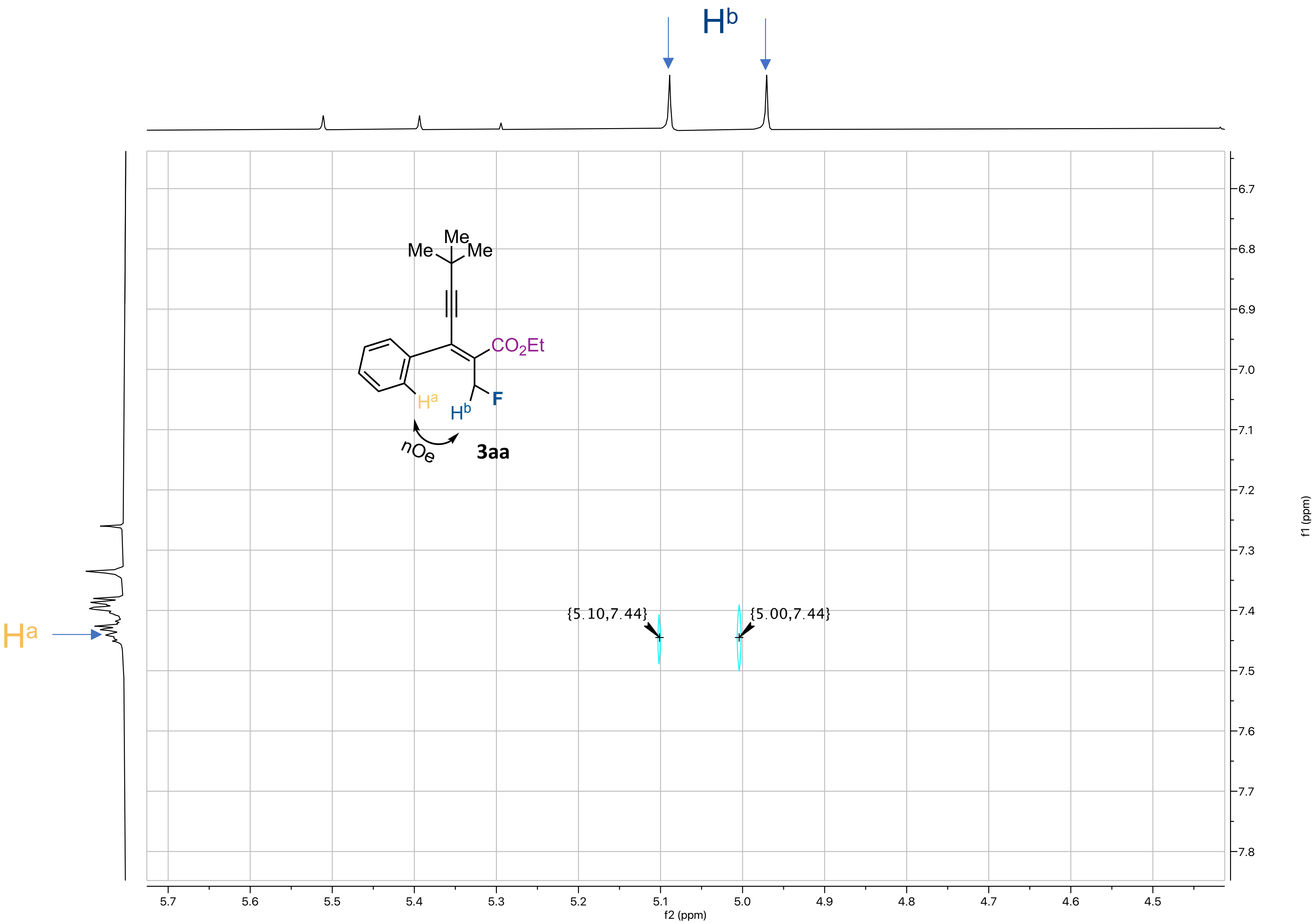

Hb
Ha
Me
Me
Me
CO2Et
F
nOe
3aa
{5.10,7.44}
{5.00,7.44}
f2 (ppm)
f1 (ppm)

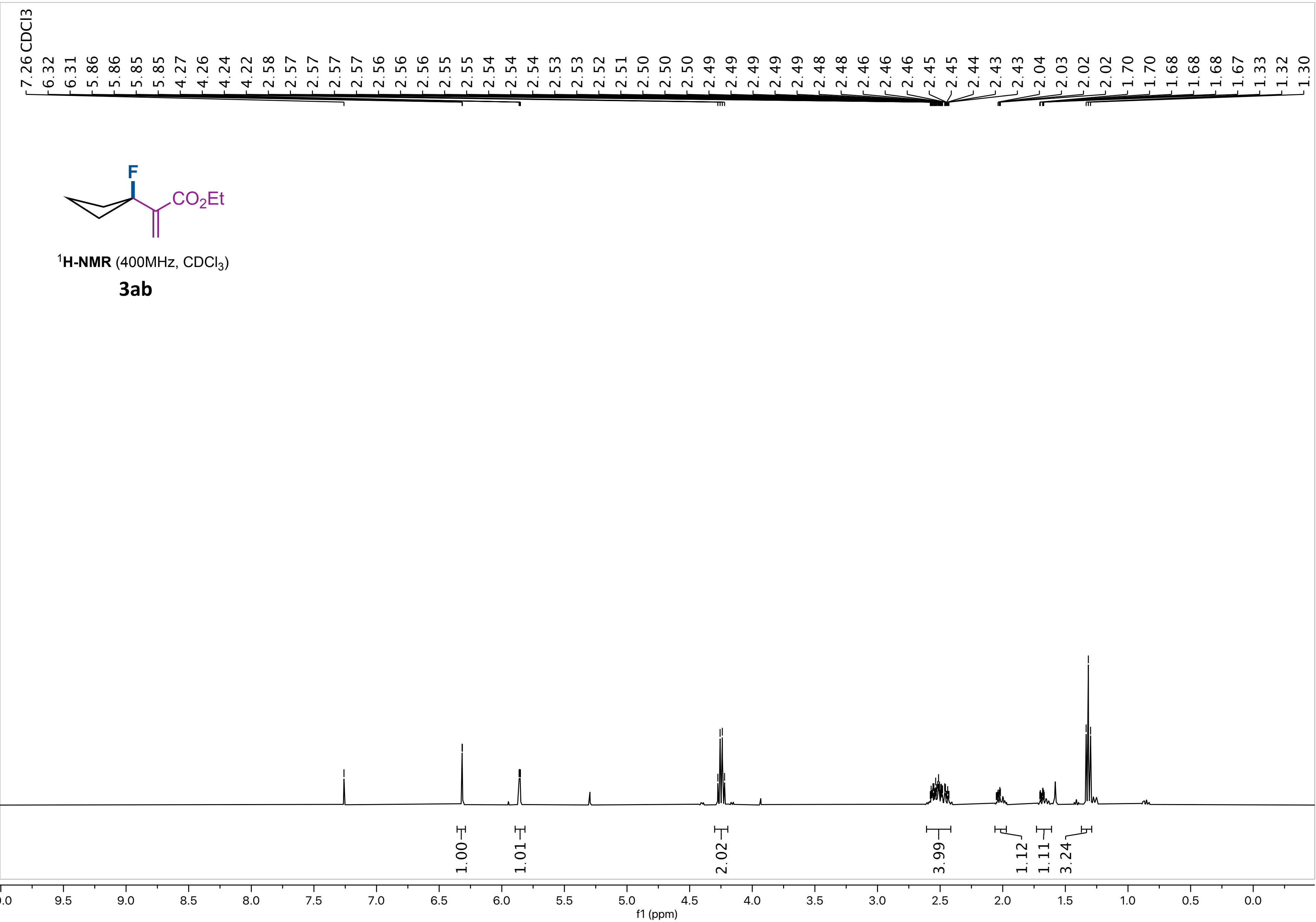
7.26 CDCl3
6.32
6.31
5.86
5.86
5.85
5.85
4.27
4.26
4.24
4.22
2.58
2.57
2.57
2.57
2.57
2.56
2.56
2.56
2.55
2.55
2.54
2.54
2.54
2.53
2.53
2.52
2.51
2.50
2.50
2.50
2.49
2.49
2.49
2.49
2.49
2.48
2.48
2.46
2.46
2.46
2.45
2.45
2.44
2.43
2.43
2.04
2.03
2.02
2.02
1.70
1.70
1.68
1.68
1.68
1.67
1.33
1.32
1.30
F
CO2Et
1H-NMR (400MHz, CDCl3)
3ab
1.00
1.01
2.02
3.99
1.12
1.11
3.24
f1 (ppm)

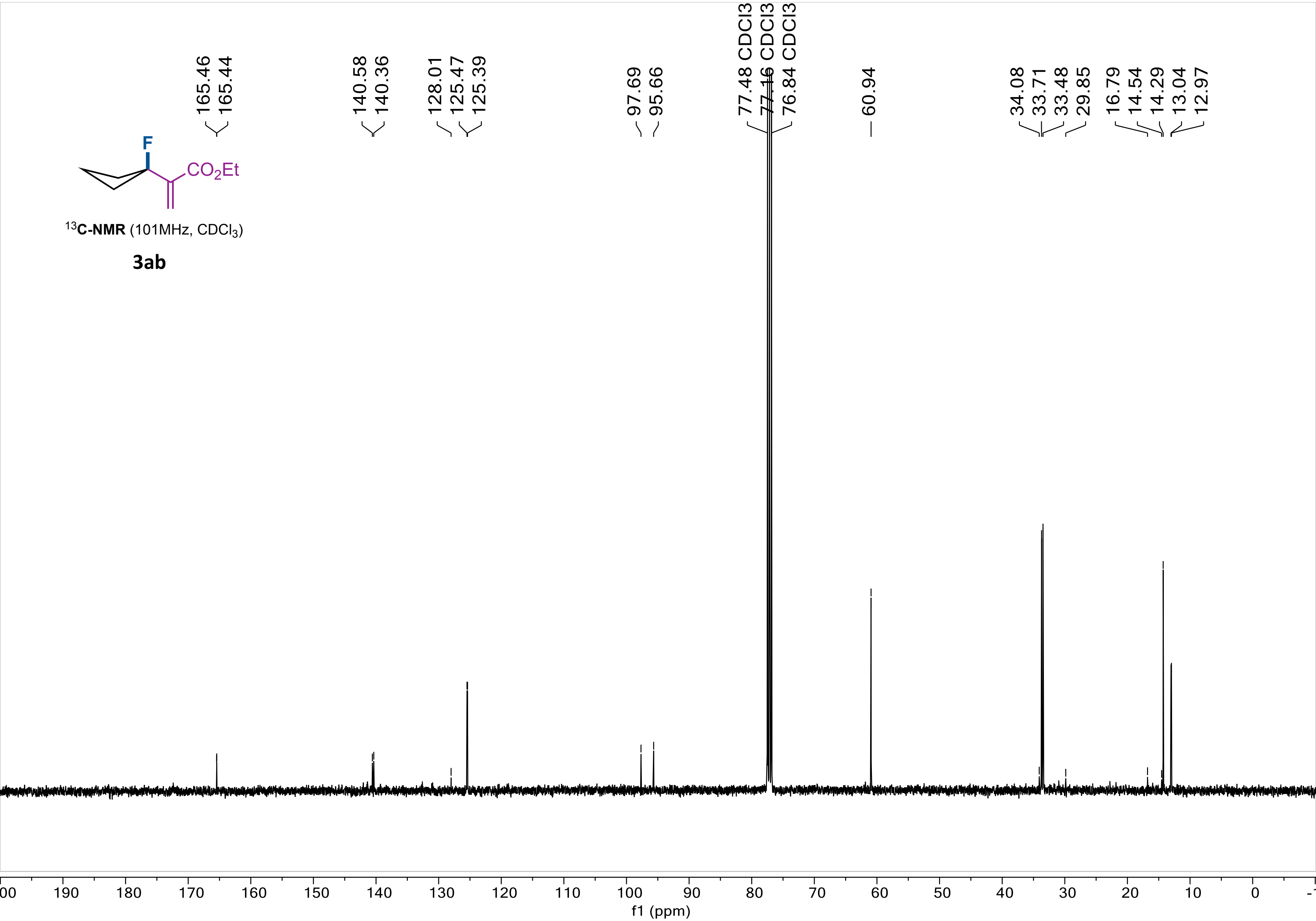

165.46
165.44
140.58
140.36
128.01
125.47
125.39
97.69
95.66
77.48 CDCl3
77.16 CDCl3
76.84 CDCl3
60.94
34.08
33.71
33.48
29.85
16.79
14.54
14.29
13.04
12.97
F
CO2Et
13C-NMR (101MHz, CDCl3)
f1 (ppm)

3ab

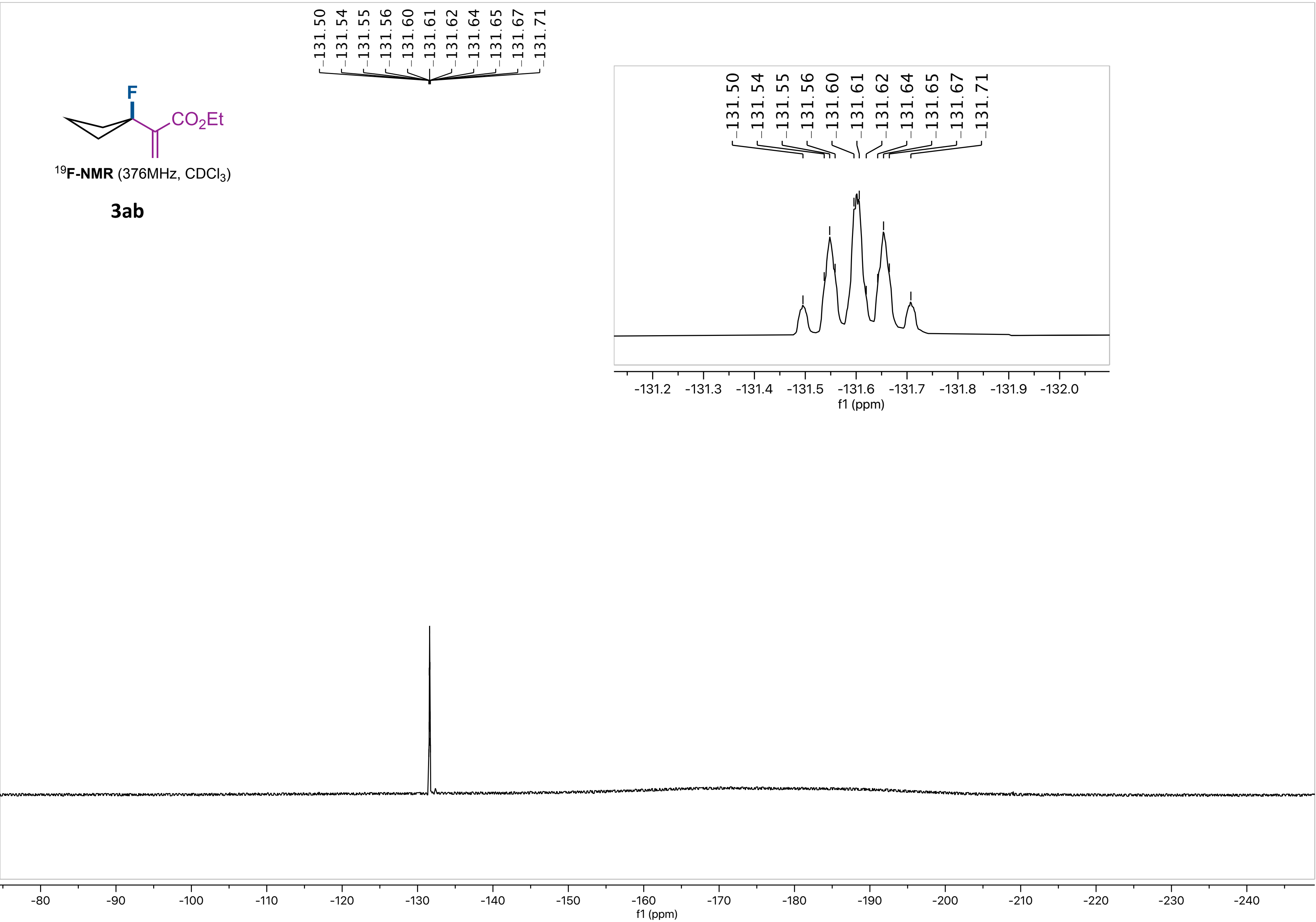
F
CO2Et
19F-NMR (376MHz, CDCl3)
3ab
-131.50
-131.54
-131.55
-131.56
-131.60
-131.61
-131.62
-131.64
-131.65
-131.67
-131.71
-131.2 -131.3 -131.4 -131.5 -131.6 -131.7 -131.8 -131.9 -132.0
f1 (ppm)
-80 -90 -100 -110 -120 -130 -140 -150 -160 -170 -180 -190 -200 -210 -220 -230 -240
f1 (ppm)

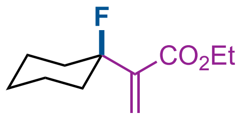

[1]**H-NMR** (400MHz, $CDCl_3$)

3ac

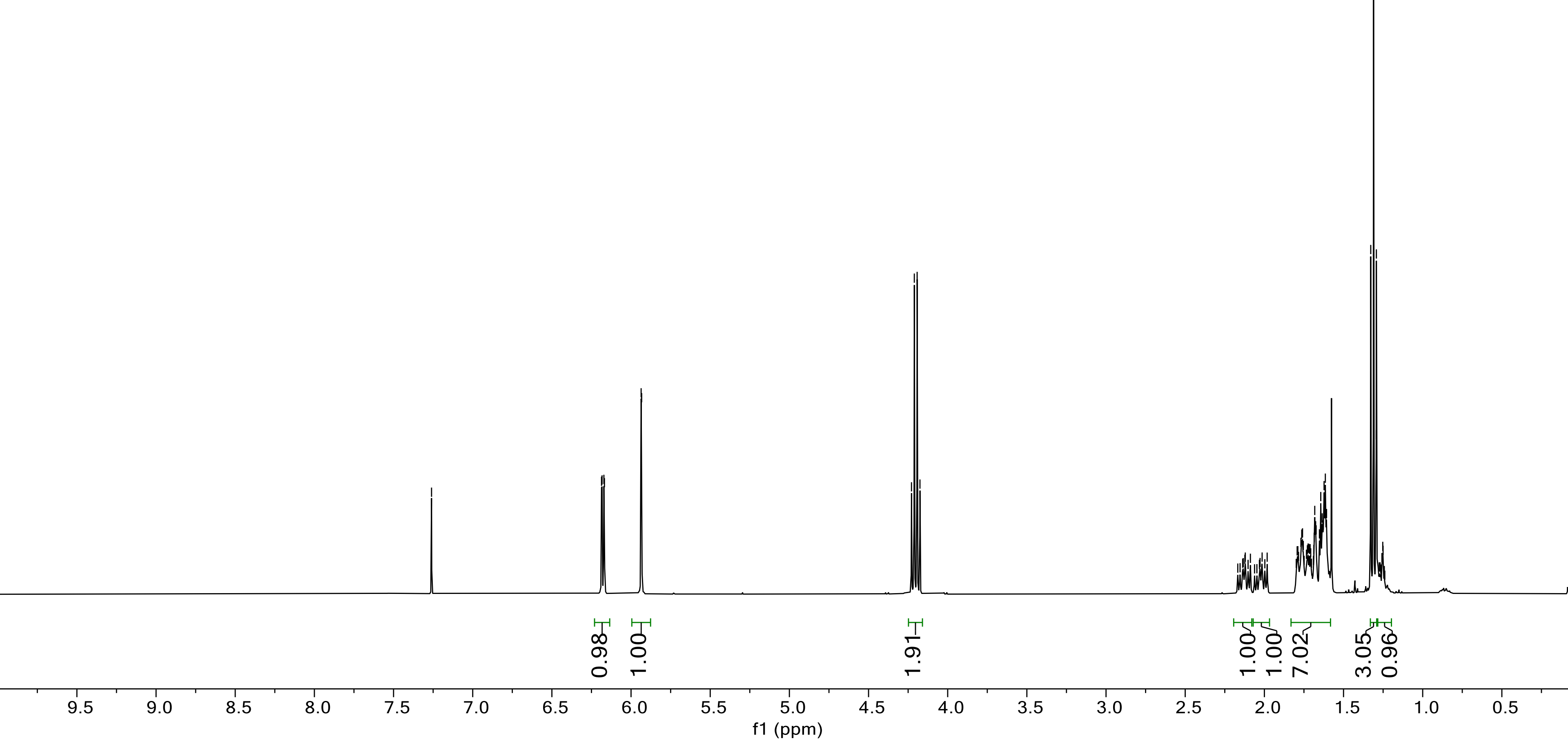

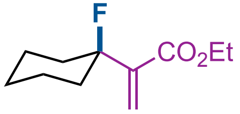

^{13}**C-NMR** (101MHz, $CDCl_3$)

3ac

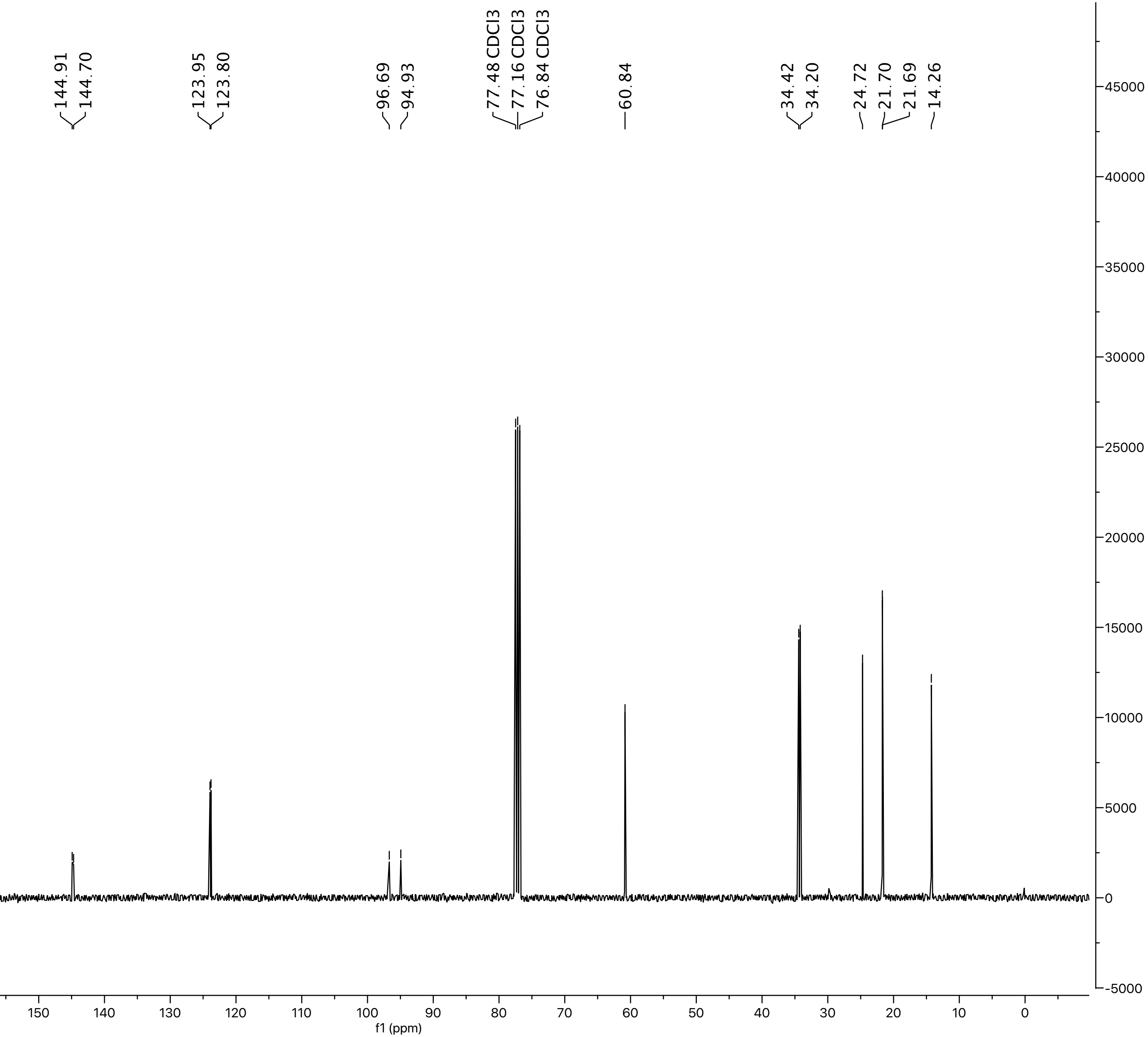

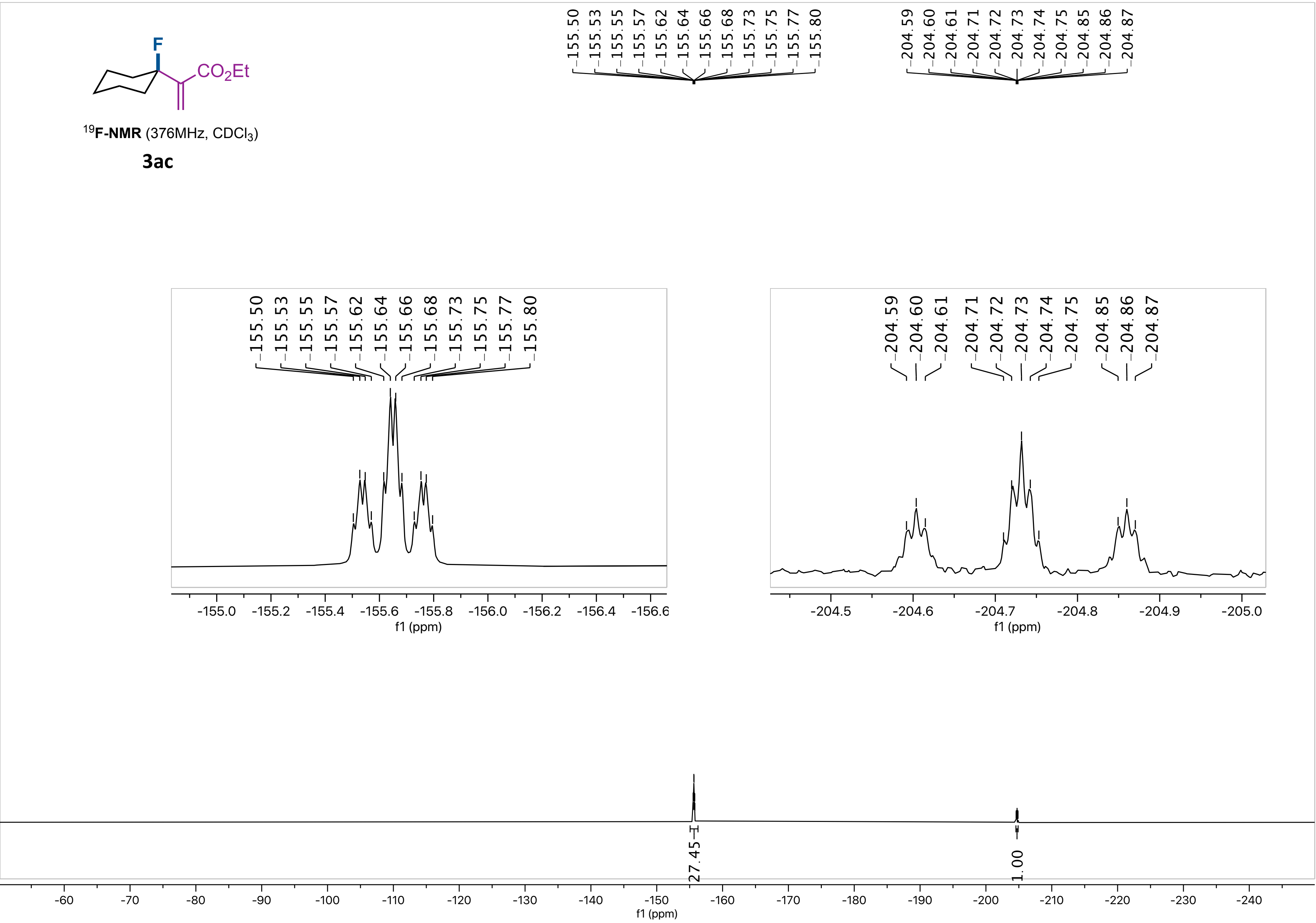
F
CO2Et
19F-NMR (376MHz, CDCl3)
3ac
-155.50 -155.53 -155.55 -155.57 -155.62 -155.64 -155.66 -155.68 -155.73 -155.75 -155.77 -155.80
-204.59 -204.60 -204.61 -204.71 -204.72 -204.73 -204.74 -204.75 -204.85 -204.86 -204.87
27.45
1.00
f1 (ppm)

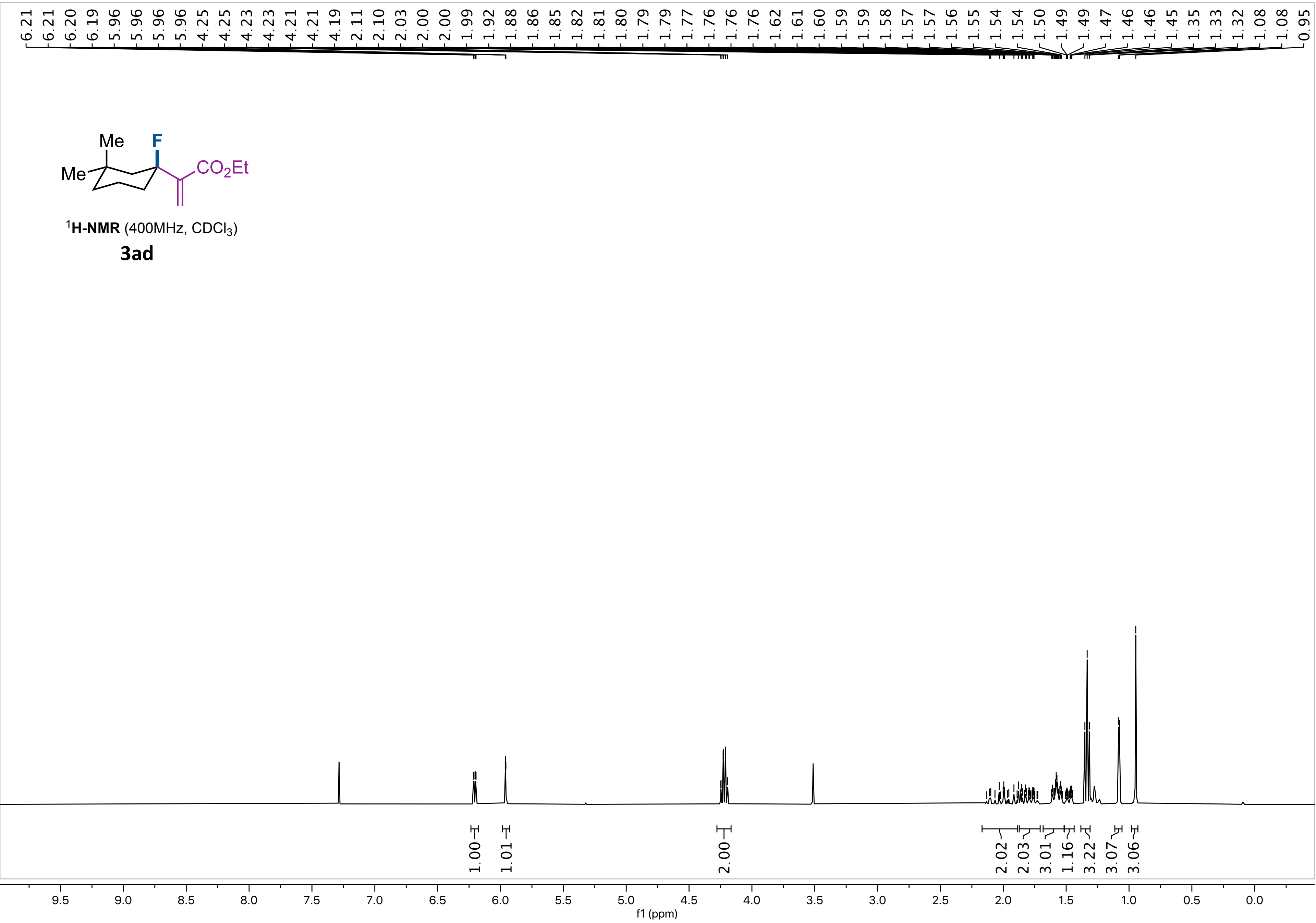
6.21 6.21 6.20 6.19 5.96 5.96 5.96 5.96 4.25 4.25 4.23 4.23 4.21 4.21 4.19 2.11 2.10 2.03 2.00 2.00 1.99 1.92 1.88 1.86 1.85 1.82 1.81 1.80 1.79 1.79 1.77 1.76 1.76 1.76 1.62 1.61 1.60 1.59 1.59 1.58 1.57 1.57 1.56 1.55 1.54 1.54 1.50 1.49 1.49 1.47 1.46 1.46 1.45 1.35 1.33 1.32 1.08 1.08 0.95
Me
Me
F
CO2Et
1H-NMR (400MHz, CDCl3)
3ad
1.00
1.01
2.00
2.02
2.03
3.01
1.16
3.22
3.07
3.06
9.5 9.0 8.5 8.0 7.5 7.0 6.5 6.0 5.5 5.0 4.5 4.0 3.5 3.0 2.5 2.0 1.5 1.0 0.5 0.0
f1 (ppm)

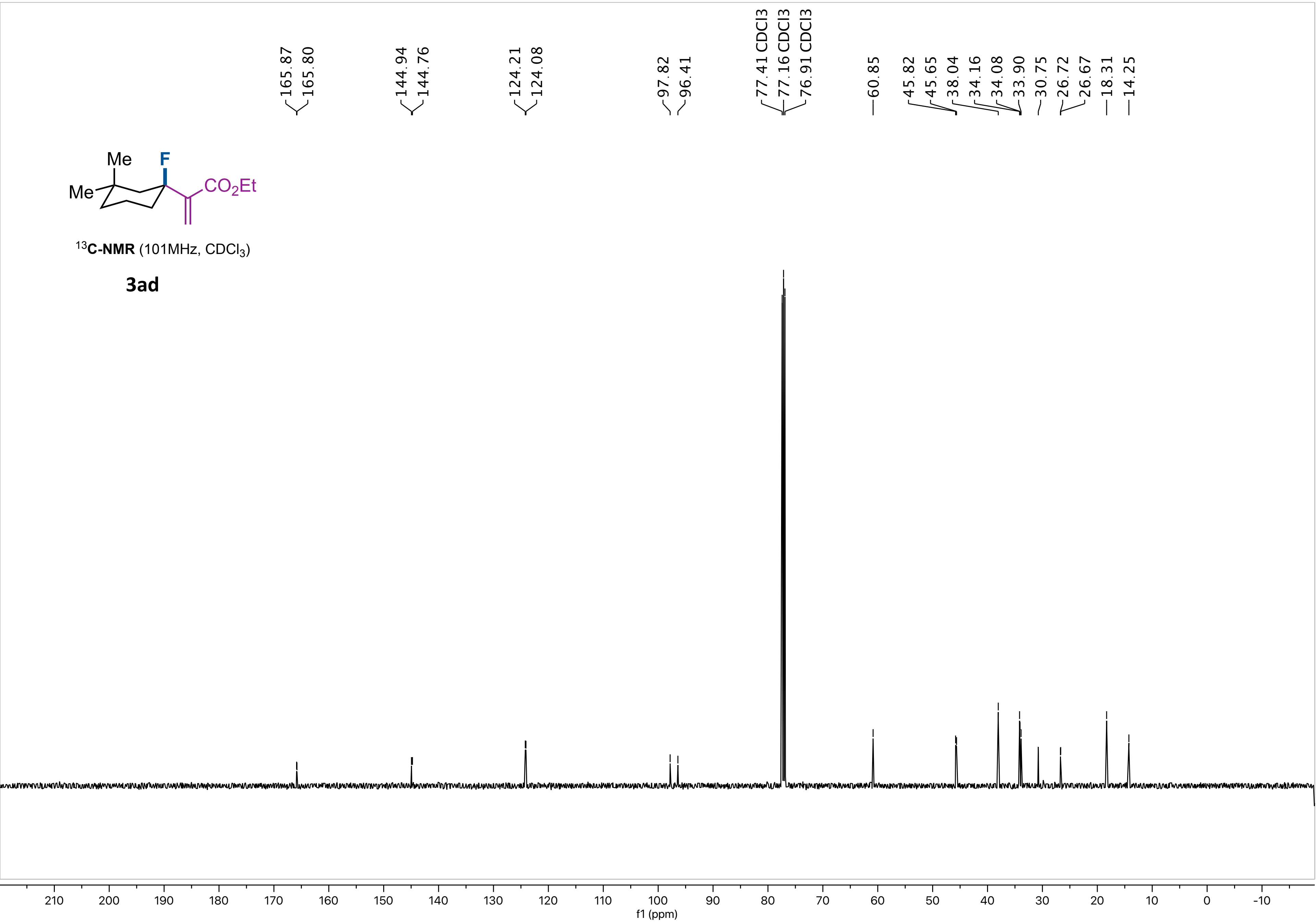
Me
F
Me
CO2Et
13C-NMR (101MHz, CDCl3)
3ad
165.87
165.80
144.94
144.76
124.21
124.08
97.82
96.41
77.41 CDCl3
77.16 CDCl3
76.91 CDCl3
60.85
45.82
45.65
38.04
34.16
34.08
33.90
30.75
26.72
26.67
18.31
14.25
210
200
190
180
170
160
150
140
130
120
110
100
90
80
70
60
50
40
30
20
10
0
-10
f1 (ppm)

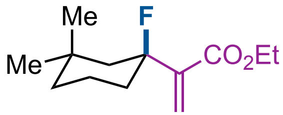

^{19}**F-NMR** (376MHz, $CDCl_3$)

3ad

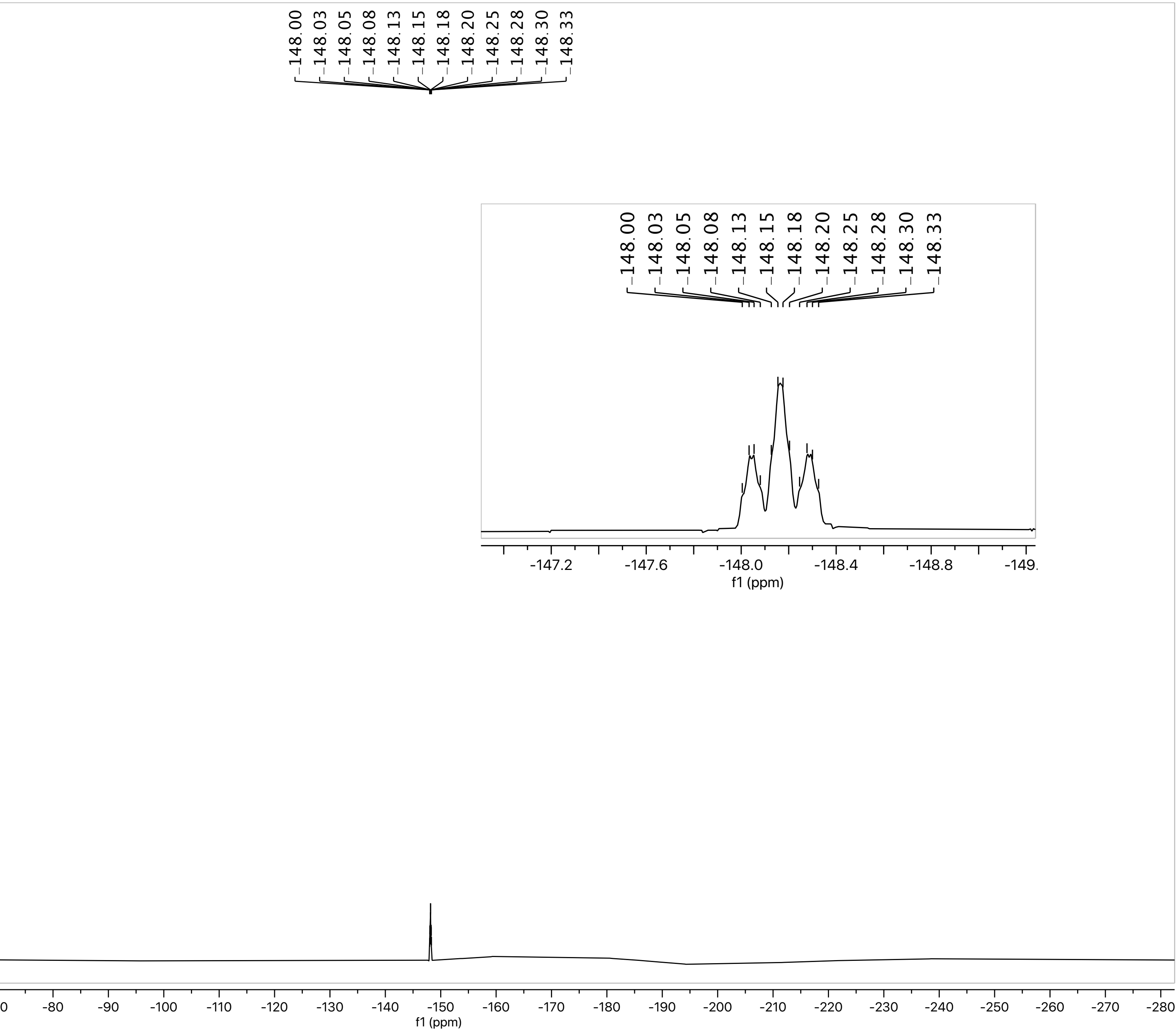

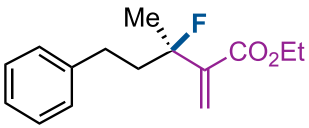

^{1}**H-NMR** (400MHz, $CDCl_3$)

3ae

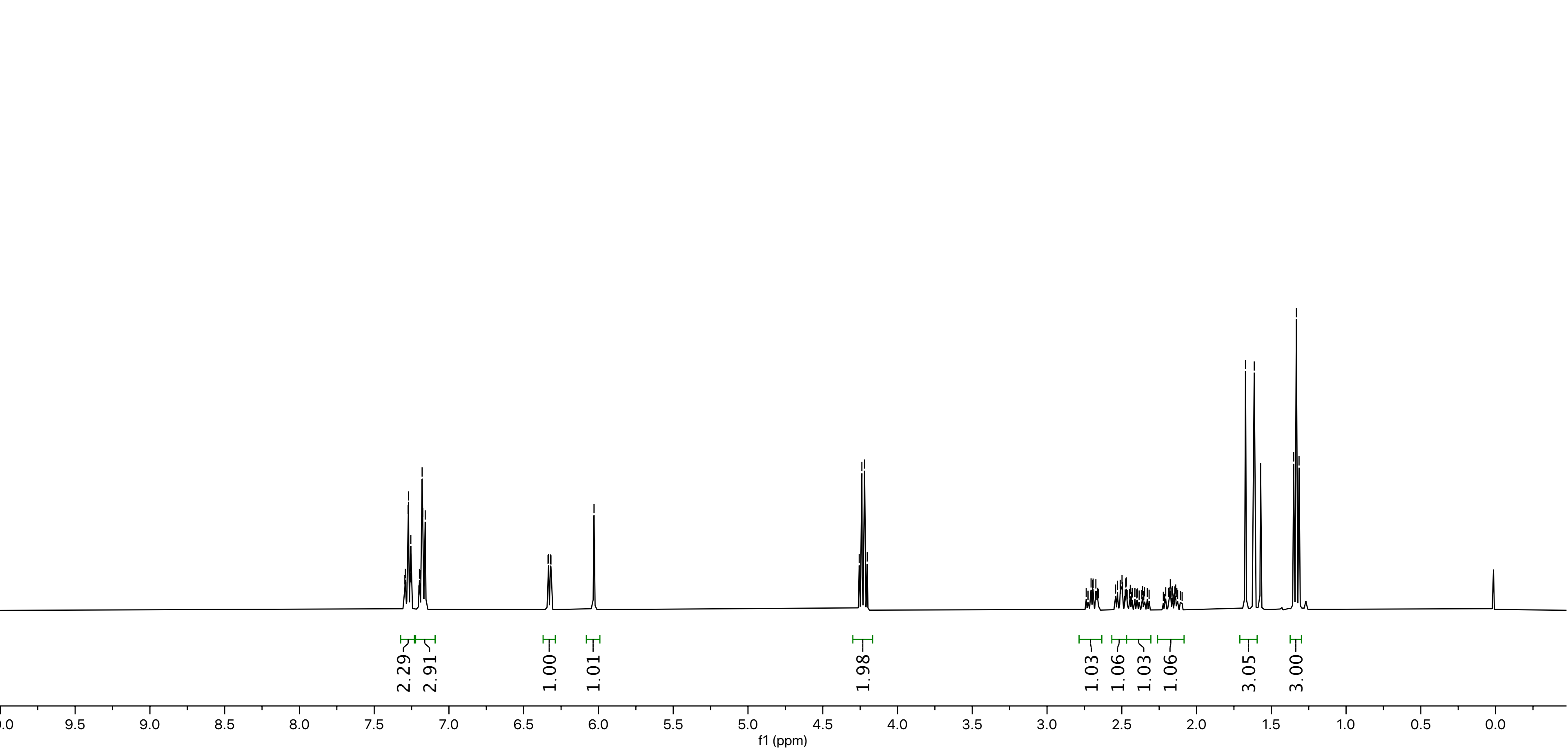

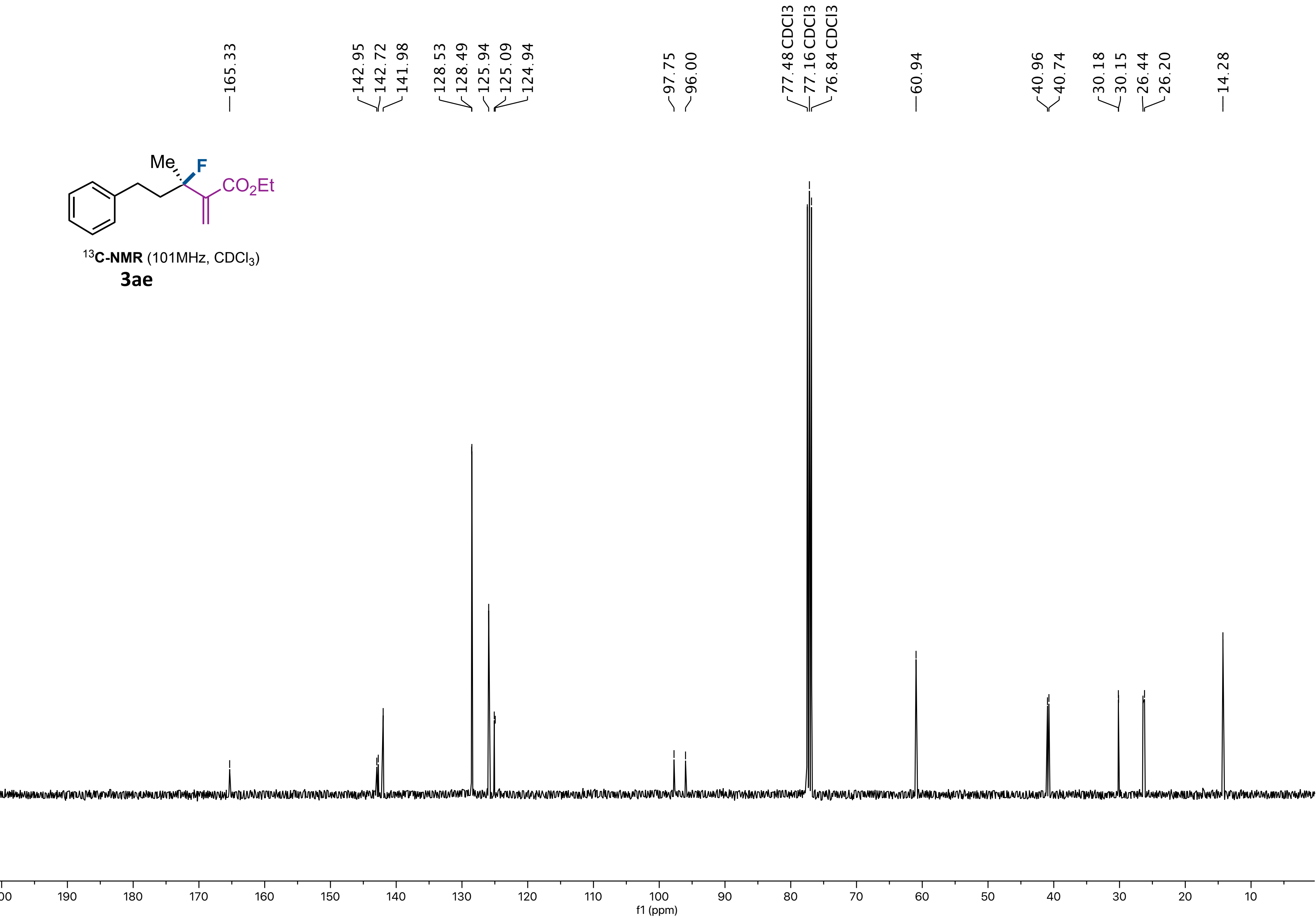

^{13}**C-NMR** (101MHz, $CDCl_3$)

3ae

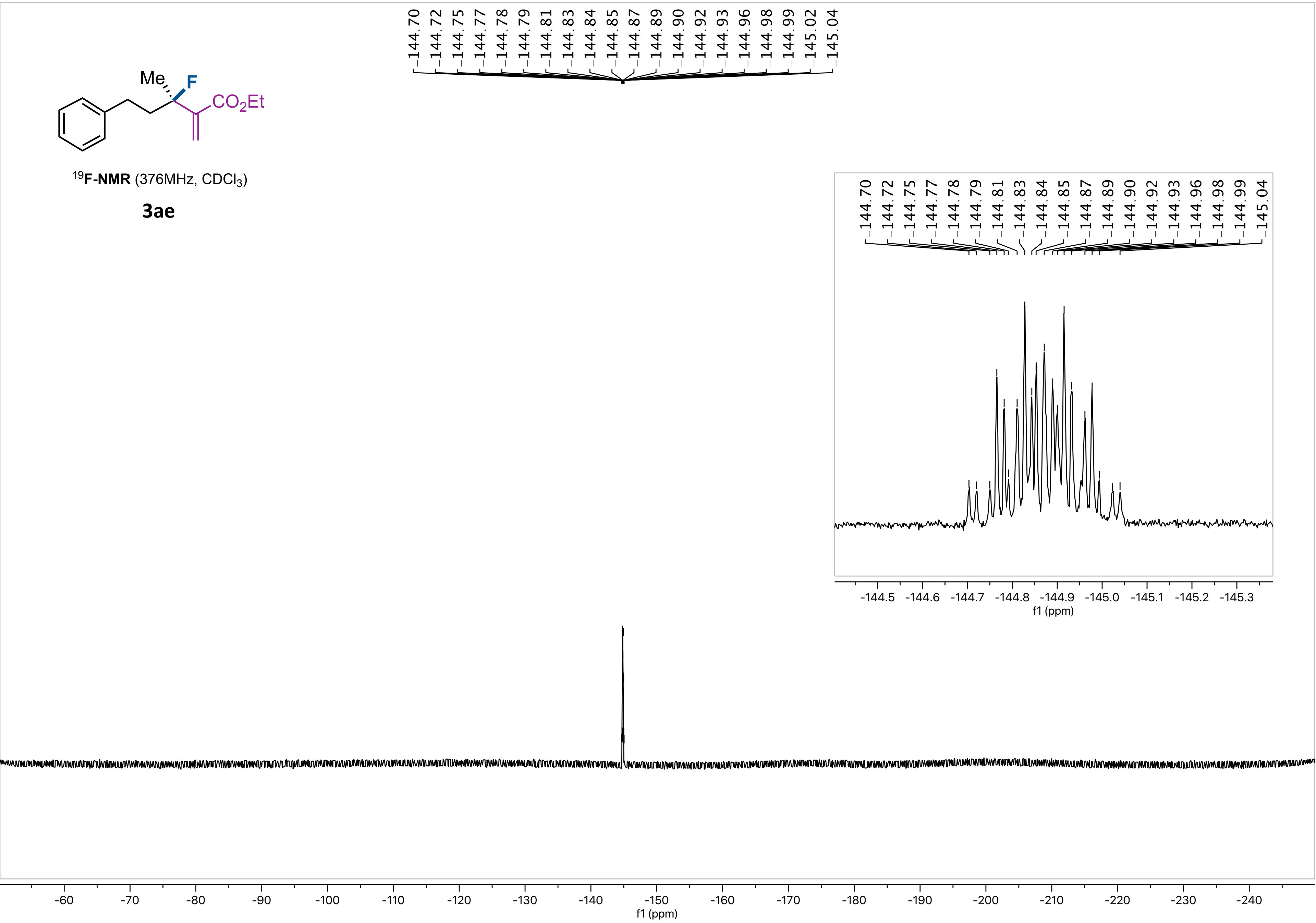
Me
F
CO_2Et
^{19}F-NMR (376MHz, $CDCl_3$)
3ae
-144.70
-144.72
-144.75
-144.77
-144.78
-144.79
-144.81
-144.83
-144.84
-144.85
-144.87
-144.89
-144.90
-144.92
-144.93
-144.96
-144.98
-144.99
-145.02
-145.04
-144.5 -144.6 -144.7 -144.8 -144.9 -145.0 -145.1 -145.2 -145.3
f1 (ppm)
-60 -70 -80 -90 -100 -110 -120 -130 -140 -150 -160 -170 -180 -190 -200 -210 -220 -230 -240
f1 (ppm)

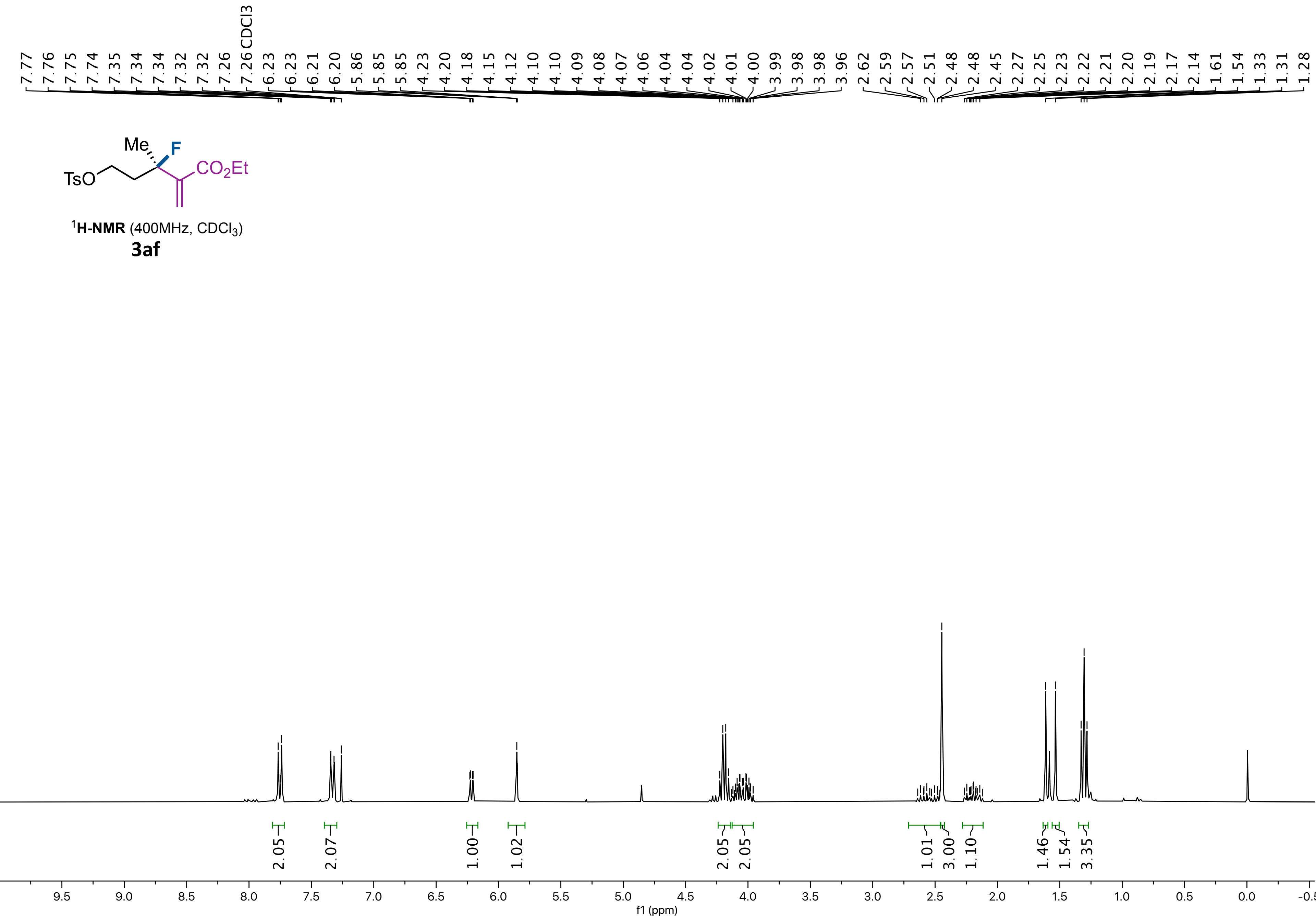

^{1}**H-NMR** (400MHz, $CDCl_3$)
3af

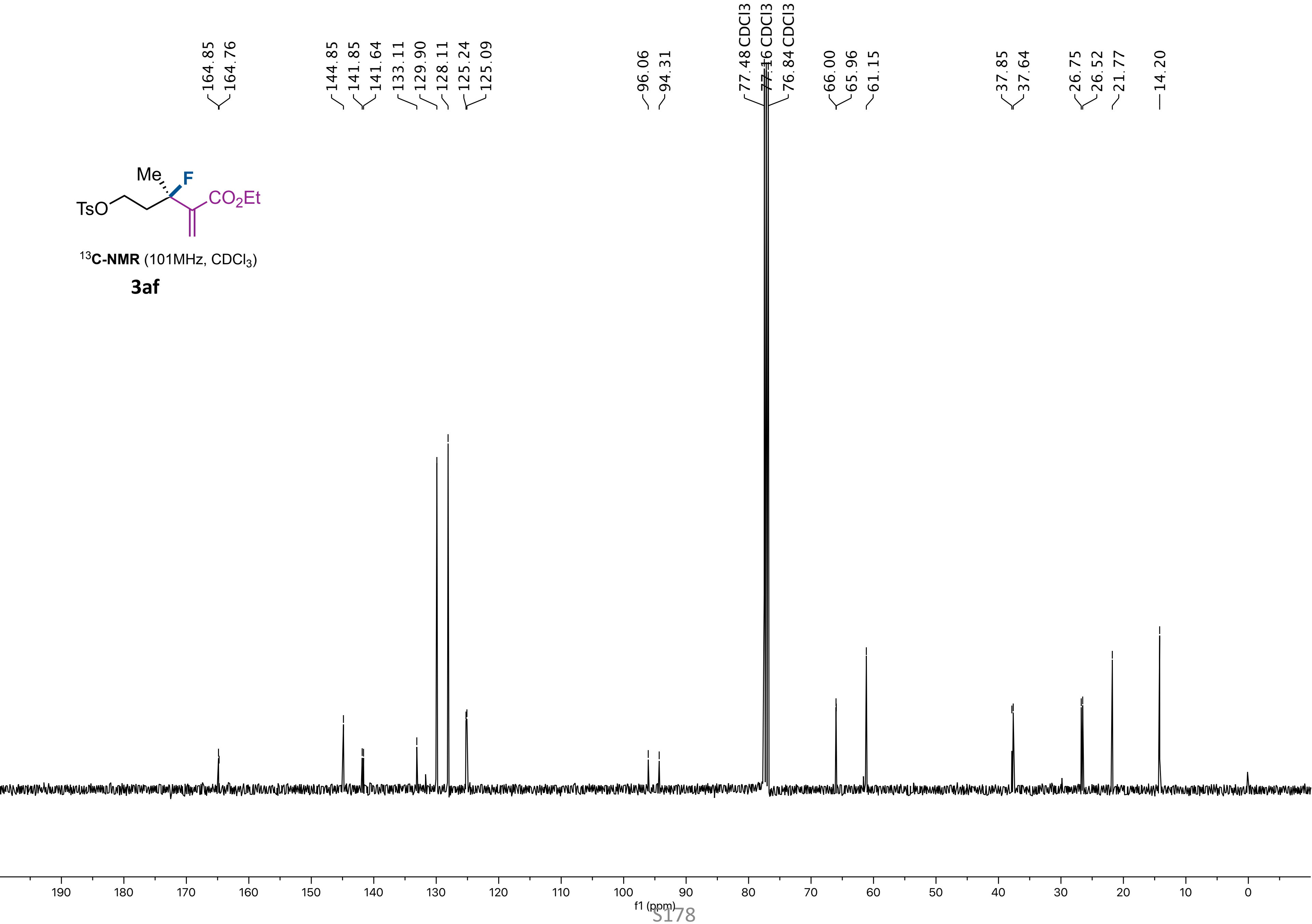
164.85
164.76
144.85
141.85
141.64
133.11
129.90
128.11
125.24
125.09
96.06
94.31
77.48 CDCl3
77.16 CDCl3
76.84 CDCl3
66.00
65.96
61.15
37.85
37.64
26.75
26.52
21.77
14.20
Me
F
TsO
CO2Et
13C-NMR (101MHz, CDCl3)
3af
190
180
170
160
150
140
130
120
110
100
90
80
70
60
50
40
30
20
10
0
f1 (ppm)

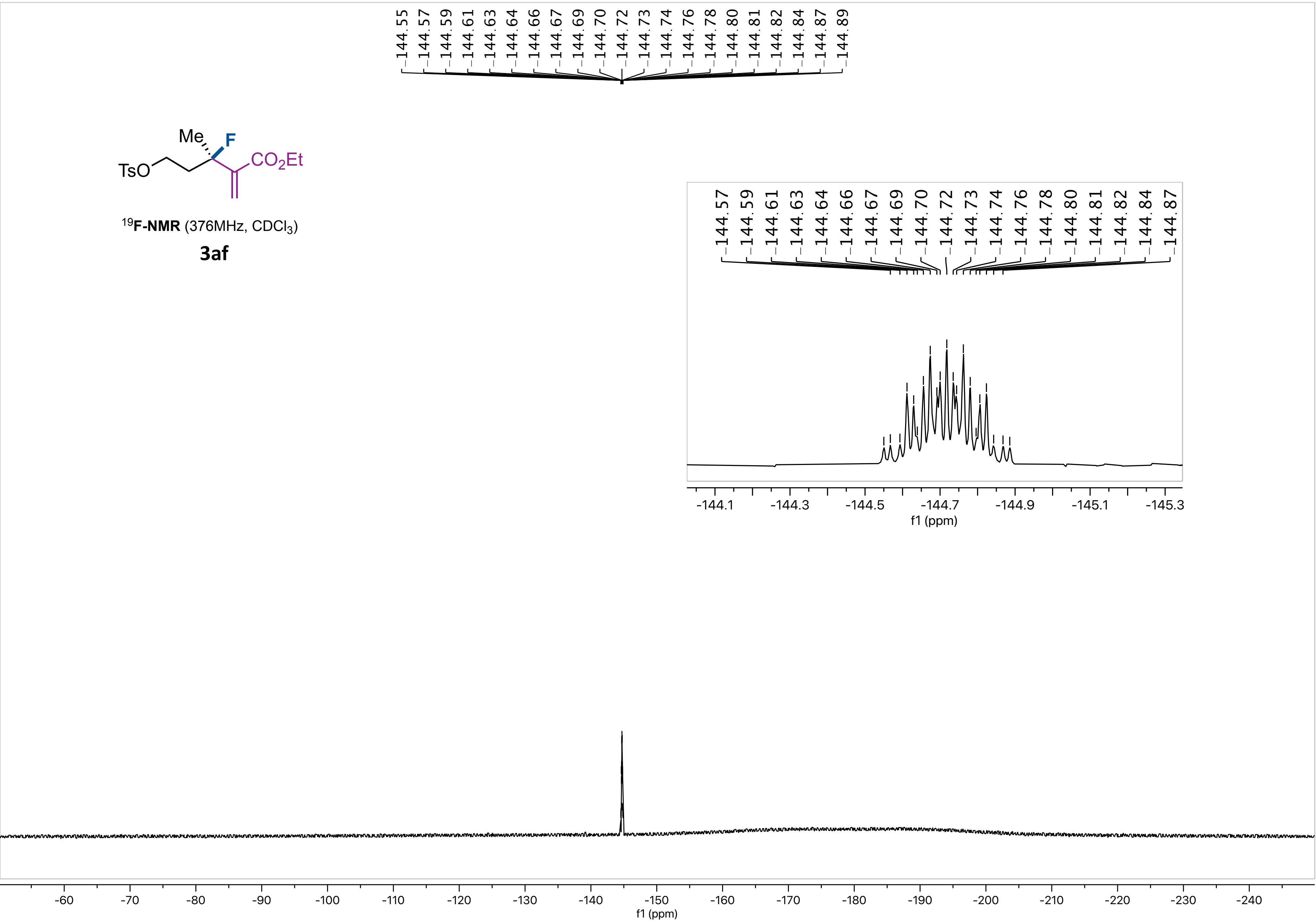

Me
F
TsO
CO_2Et
^{19}F-NMR (376MHz, $CDCl_3$)
3af
-144.55
-144.57
-144.59
-144.61
-144.63
-144.64
-144.66
-144.67
-144.69
-144.70
-144.72
-144.73
-144.74
-144.76
-144.78
-144.80
-144.81
-144.82
-144.84
-144.87
-144.89
-144.1
-144.3
-144.5
-144.7
-144.9
-145.1
-145.3
f1 (ppm)
-60
-70
-80
-90
-100
-110
-120
-130
-140
-150
-160
-170
-180
-190
-200
-210
-220
-230
-240
f1 (ppm)

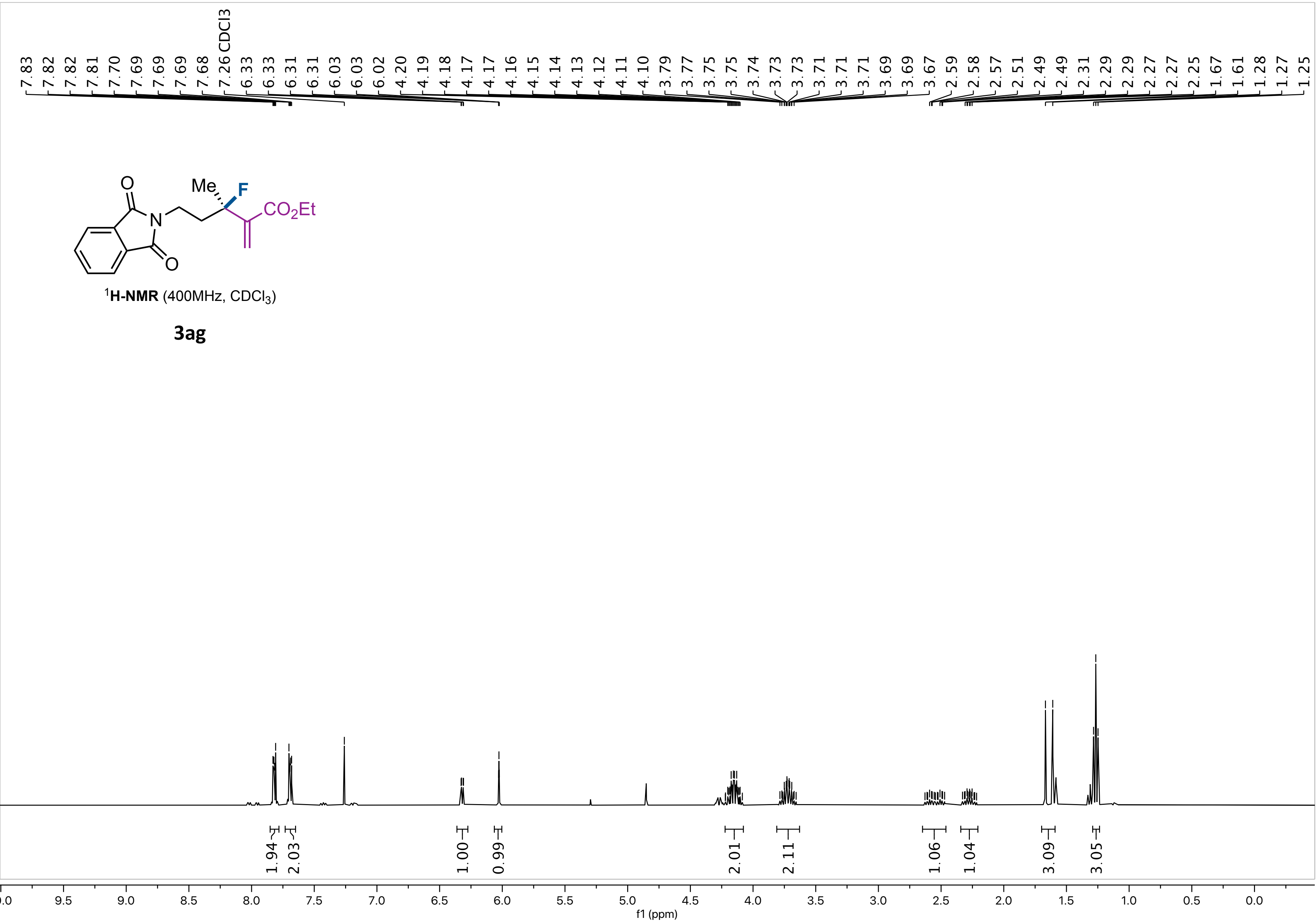

^{1}**H-NMR** (400MHz, $CDCl_3$)

3ag

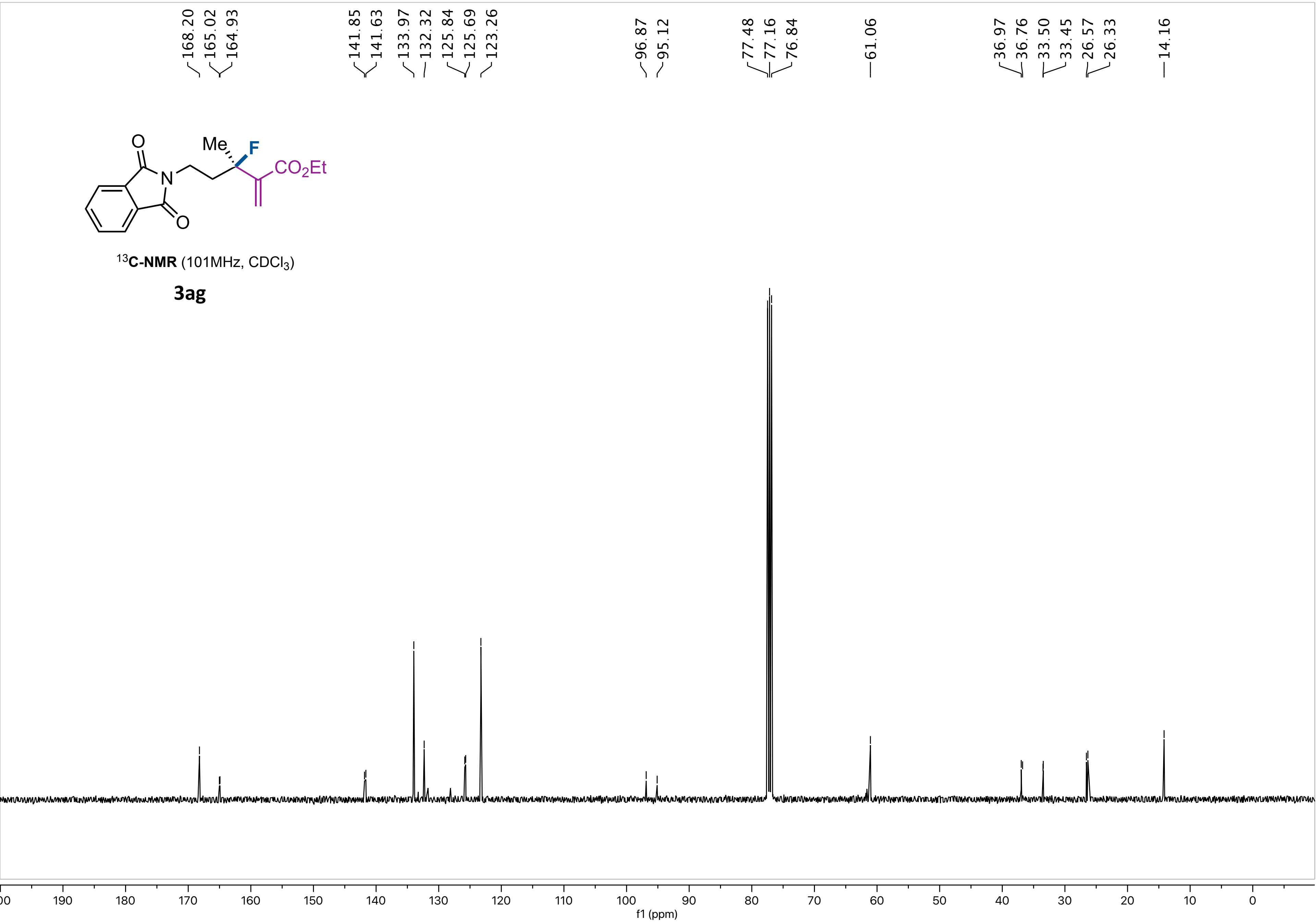

^{13}**C-NMR** (101MHz, $CDCl_3$)

3ag

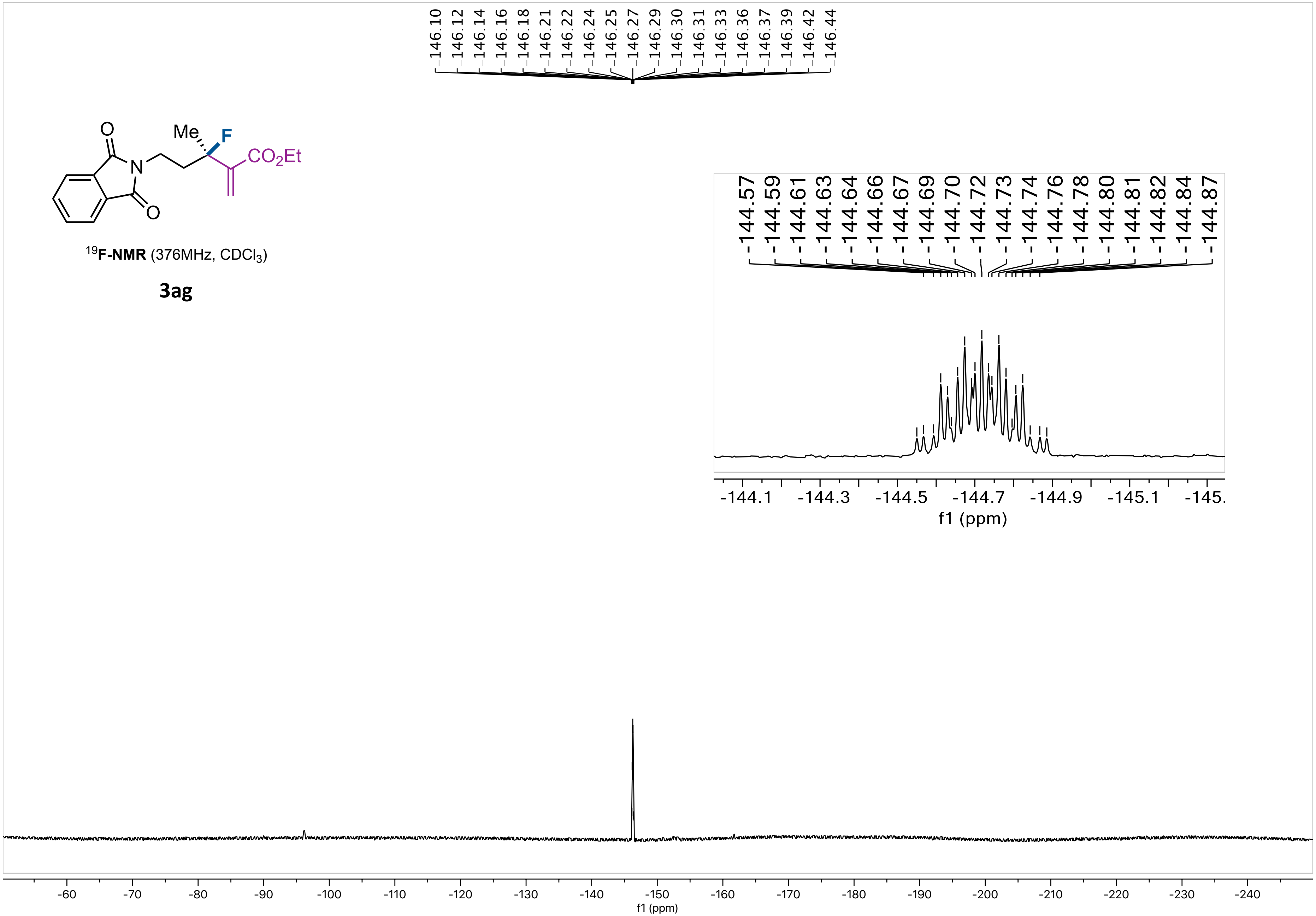

Me
F
CO2Et
O
N
O
19F-NMR (376MHz, CDCl3)
3ag
-144.1
-144.3
-144.5
-144.7
-144.9
-145.1
f1 (ppm)
-60
-70
-80
-90
-100
-110
-120
-130
-140
-150
-160
-170
-180
-190
-200
-210
-220
-230
-240
f1 (ppm)

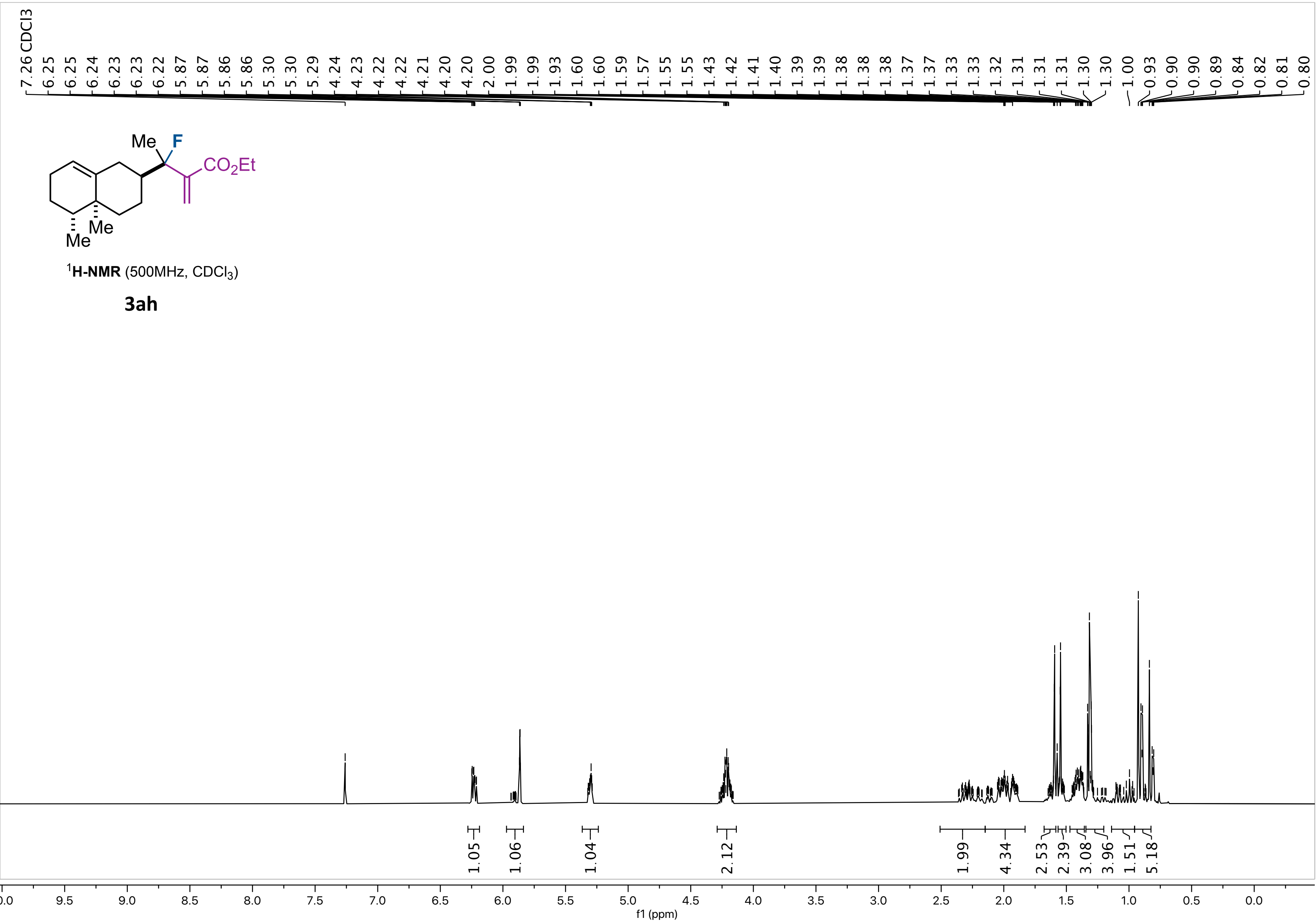
7.26 CDCl3
6.25 6.25 6.24 6.23 6.23 6.22 5.87 5.87 5.86 5.86 5.30 5.30 5.29 4.24 4.23 4.22 4.22 4.21 4.20 4.20 2.00 1.99 1.99 1.93 1.60 1.60 1.59 1.57 1.55 1.55 1.43 1.42 1.41 1.40 1.39 1.39 1.38 1.38 1.38 1.37 1.37 1.33 1.33 1.32 1.31 1.31 1.31 1.30 1.30 1.00 0.93 0.90 0.90 0.89 0.84 0.82 0.81 0.80
Me
F
CO2Et
Me
Me
1H-NMR (500MHz, CDCl3)
1.05
1.06
1.04
2.12
1.99
4.34
2.53
2.39
3.08
3.96
1.51
5.18
.0 9.5 9.0 8.5 8.0 7.5 7.0 6.5 6.0 5.5 5.0 4.5 4.0 3.5 3.0 2.5 2.0 1.5 1.0 0.5 0.0
f1 (ppm)

3ah

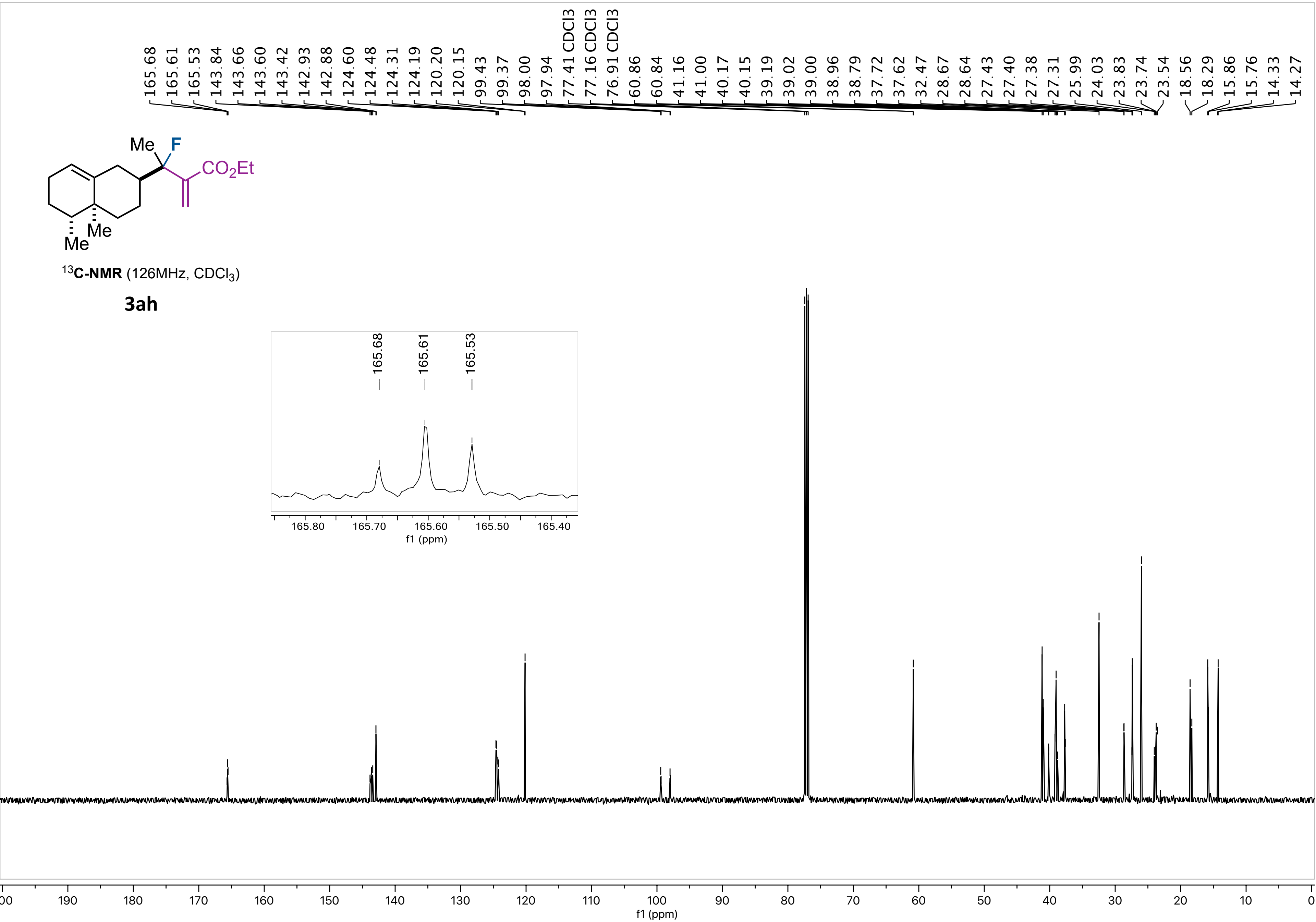

^{13}**C-NMR** (126MHz, $CDCl_3$)

3ah

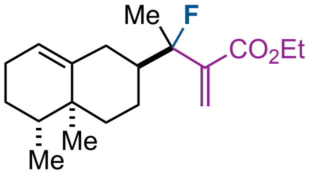

^{19}**F-NMR** (376MHz, $CDCl_3$)

3ah

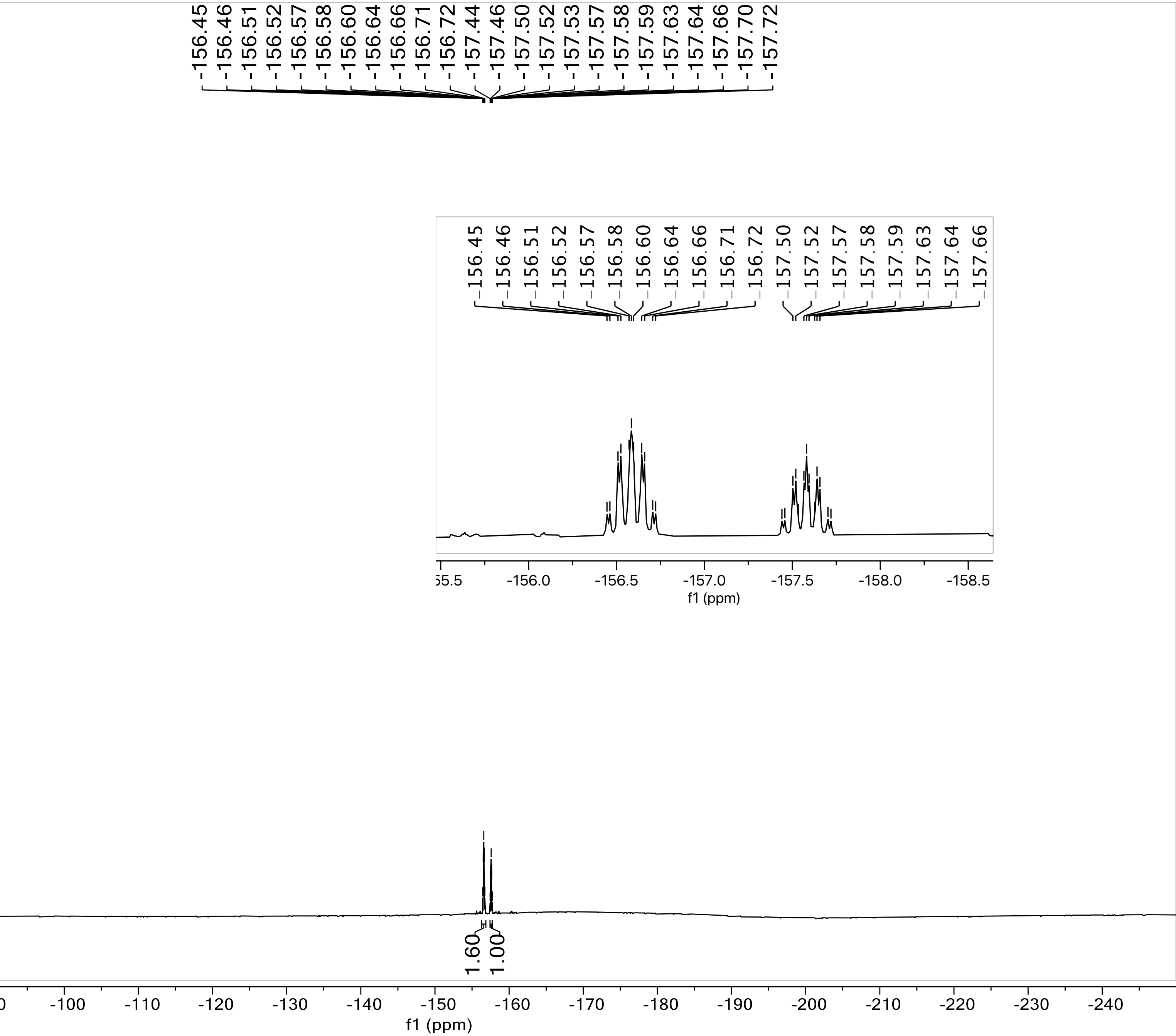

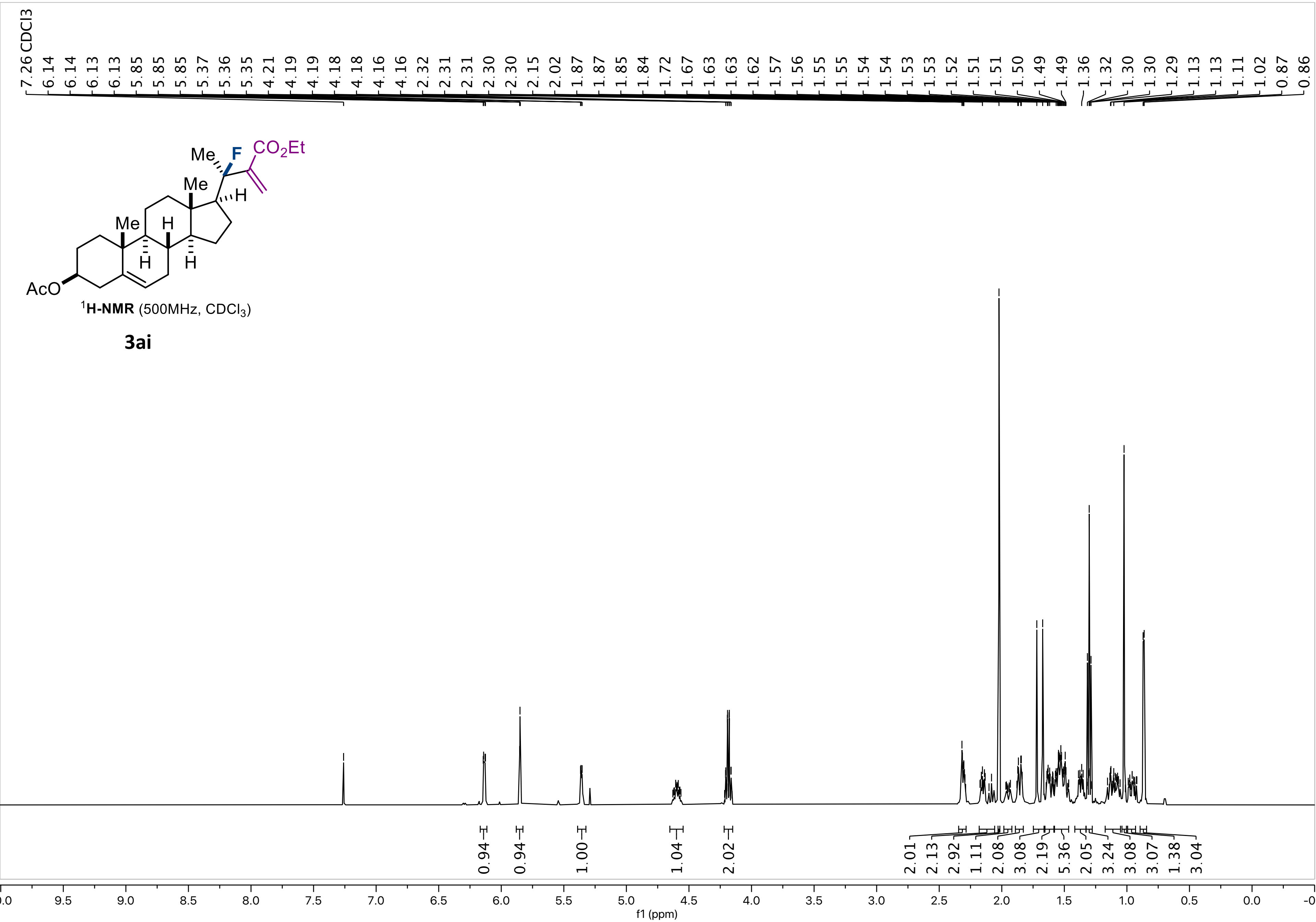

Me
F
CO2Et
Me
H
Me
H
H
H
AcO
1H-NMR (500MHz, CDCl3)
3ai
f1 (ppm)

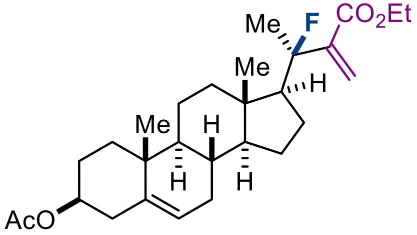

^{13}**C-NMR** (126MHz, $CDCl_3$)

3ai

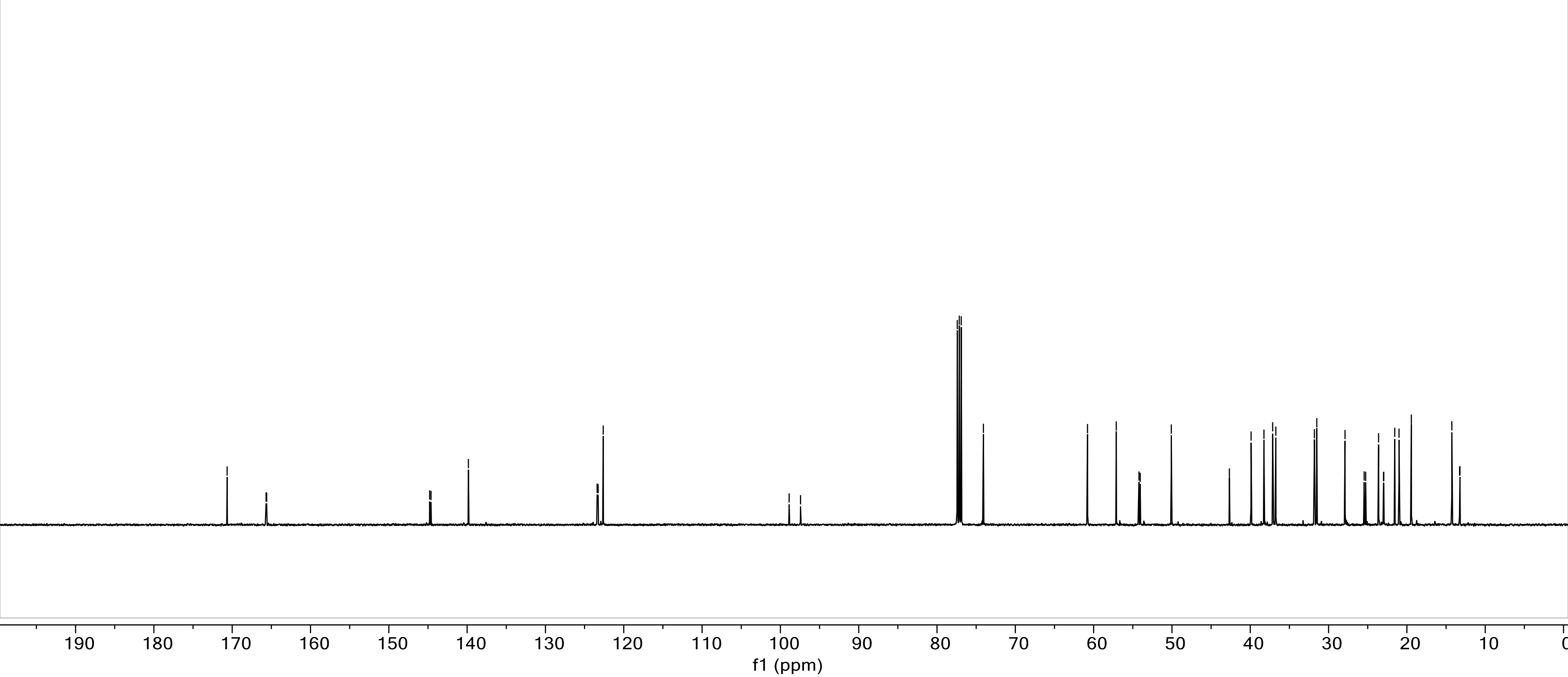

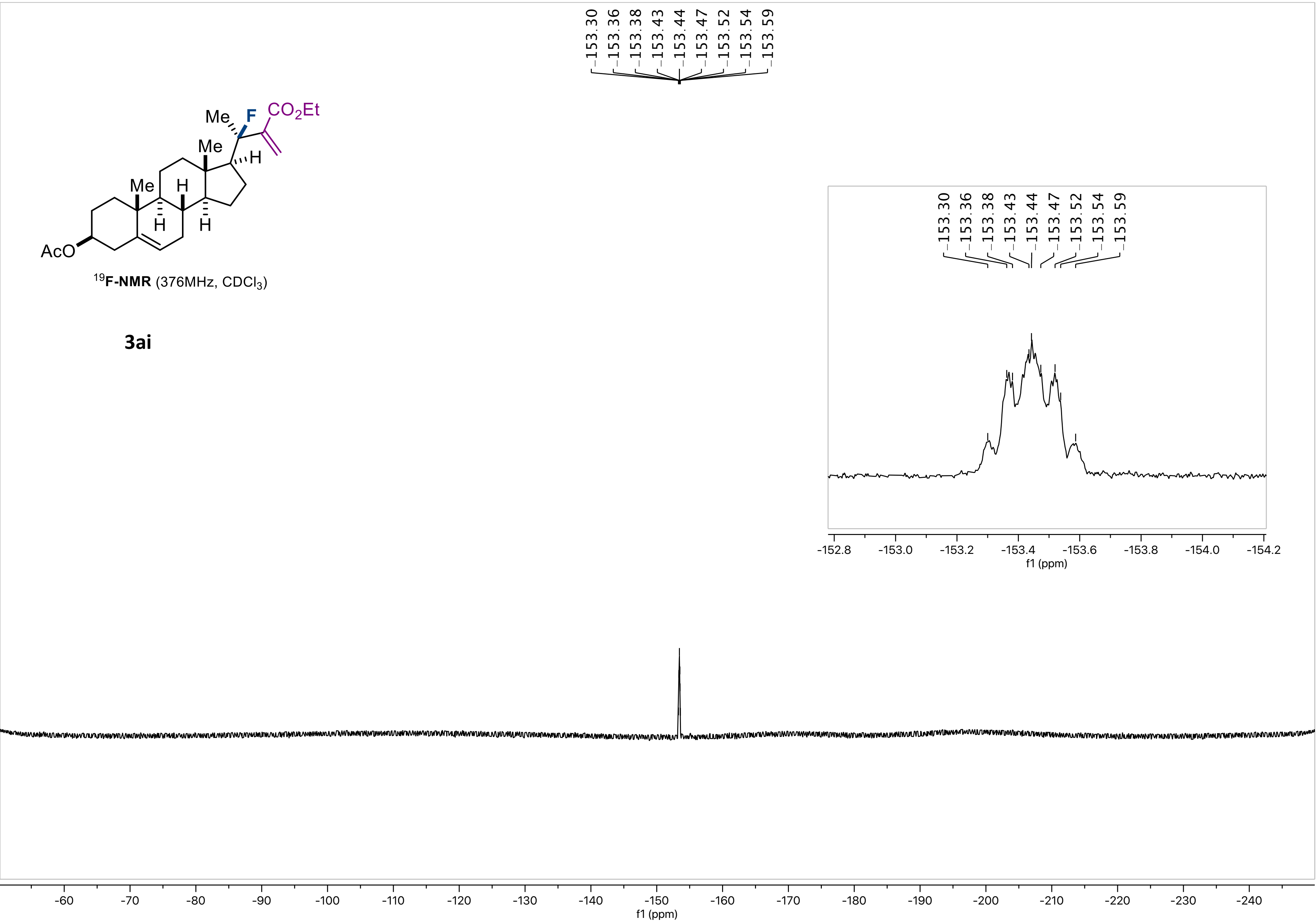

Me
F
CO2Et
Me
H
Me
H
H
H
AcO
19F-NMR (376MHz, CDCl3)
3ai
-153.30
-153.36
-153.38
-153.43
-153.44
-153.47
-153.52
-153.54
-153.59
-152.8
-153.0
-153.2
-153.4
-153.6
-153.8
-154.0
-154.2
f1 (ppm)
-60
-70
-80
-90
-100
-110
-120
-130
-140
-150
-160
-170
-180
-190
-200
-210
-220
-230
-240
f1 (ppm)

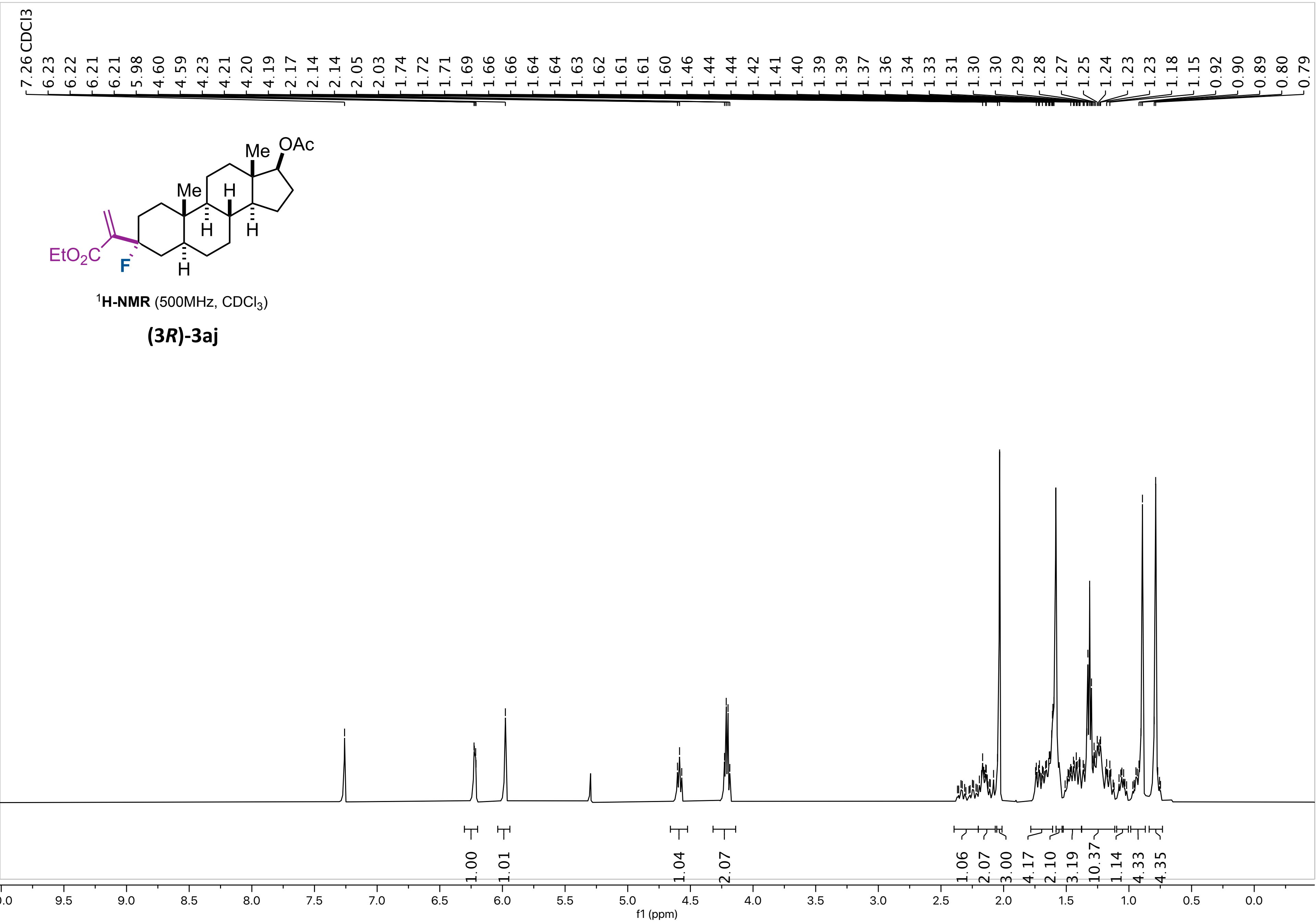

^{1}**H-NMR** (500MHz, $CDCl_3$)

(3*R*)-3aj

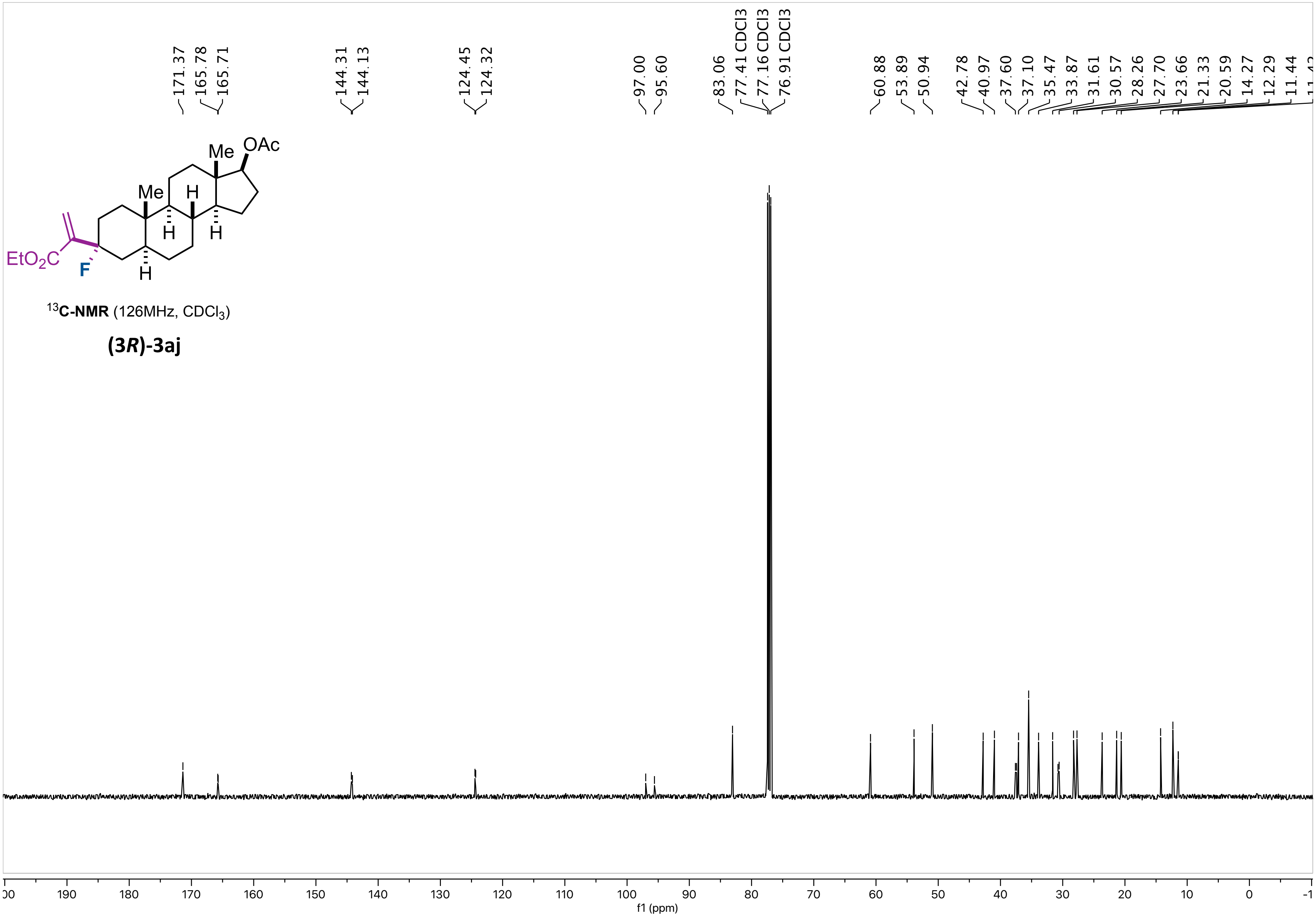

^{13}**C-NMR** (126MHz, $CDCl_3$)

(3*R*)-3aj

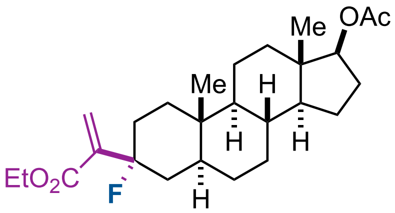

^{19}**F-NMR** (376MHz, $CDCl_3$)

(3*R*)-3aj

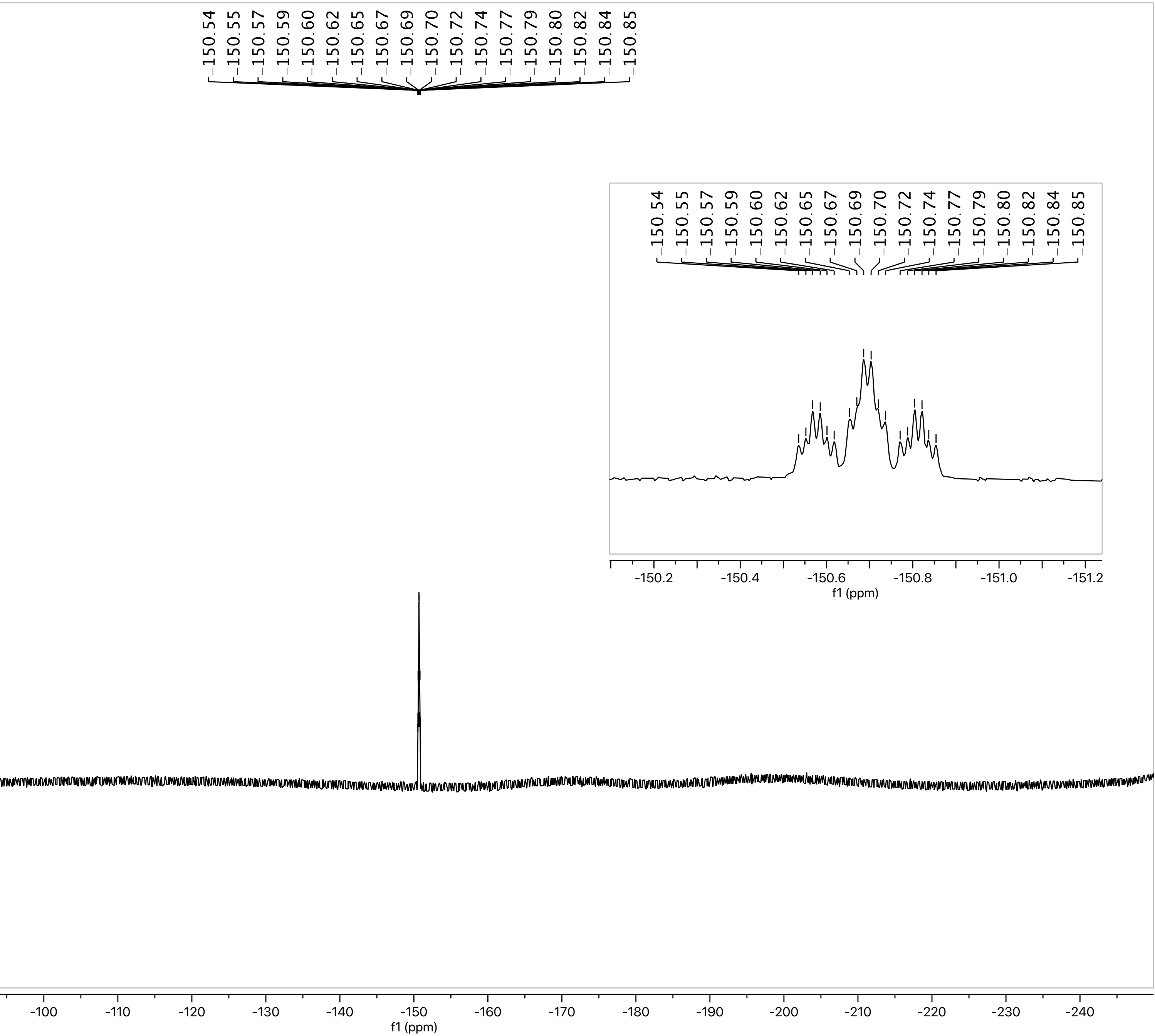

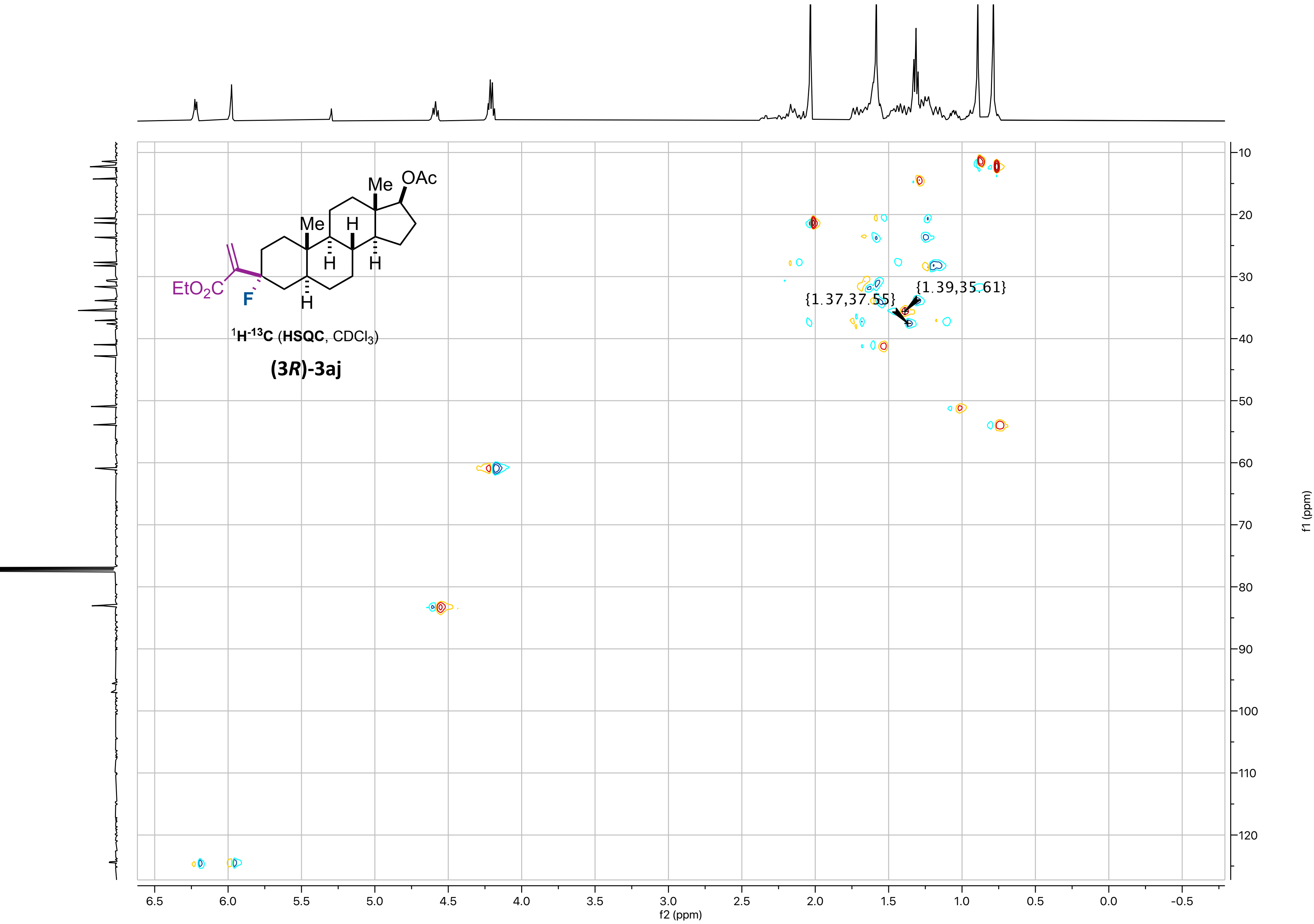
Me
OAc
Me
H
H
H
EtO2C
F
H
1H-13C (HSQC, CDCl3)
(3R)-3aj
{1.37,37.55}
{1.39,35.61}
f2 (ppm)
f1 (ppm)

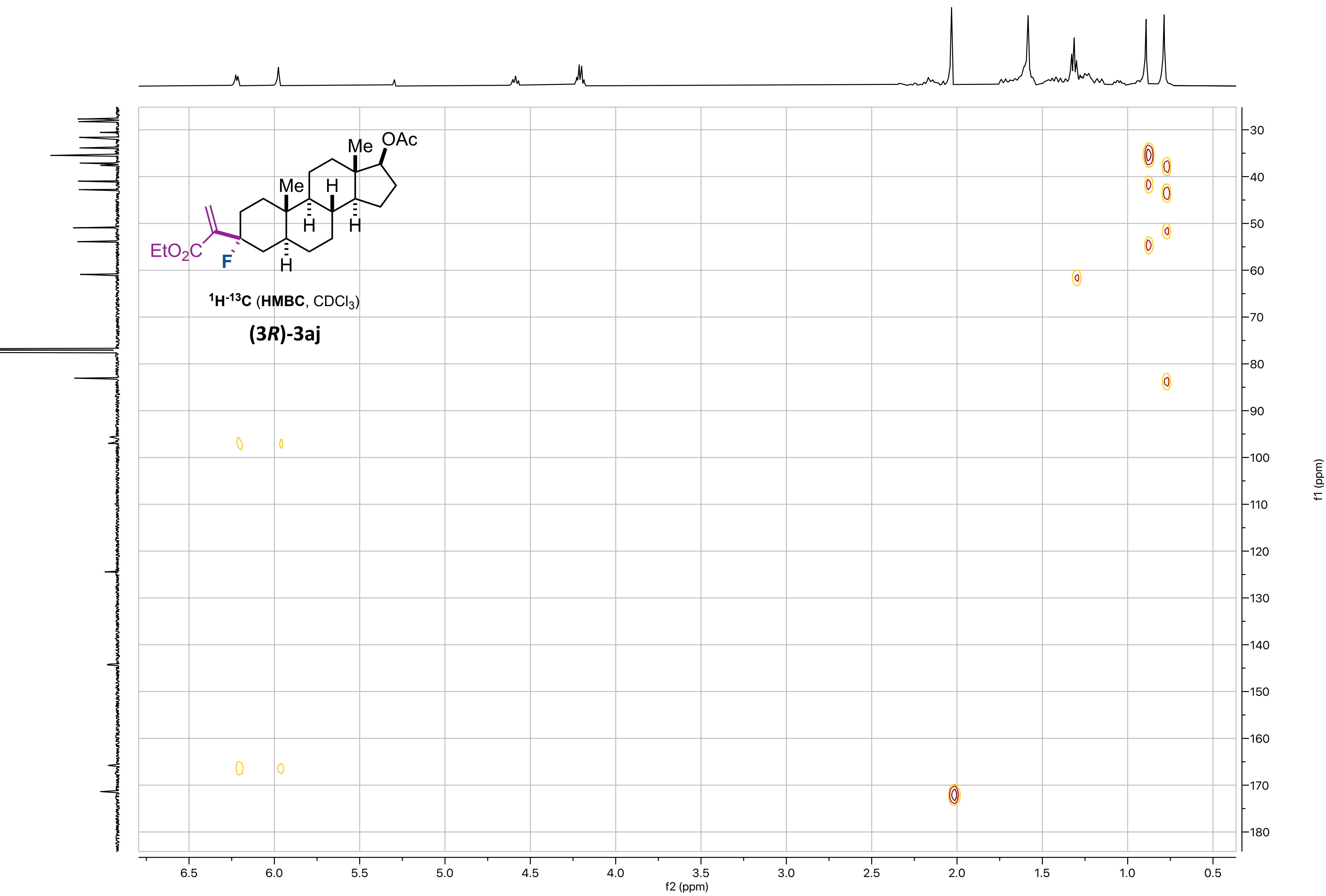
Me
OAc
Me
H
H
H
H
EtO2C
F
1H-13C (HMBC, CDCl3)
(3R)-3aj
f2 (ppm)
f1 (ppm)

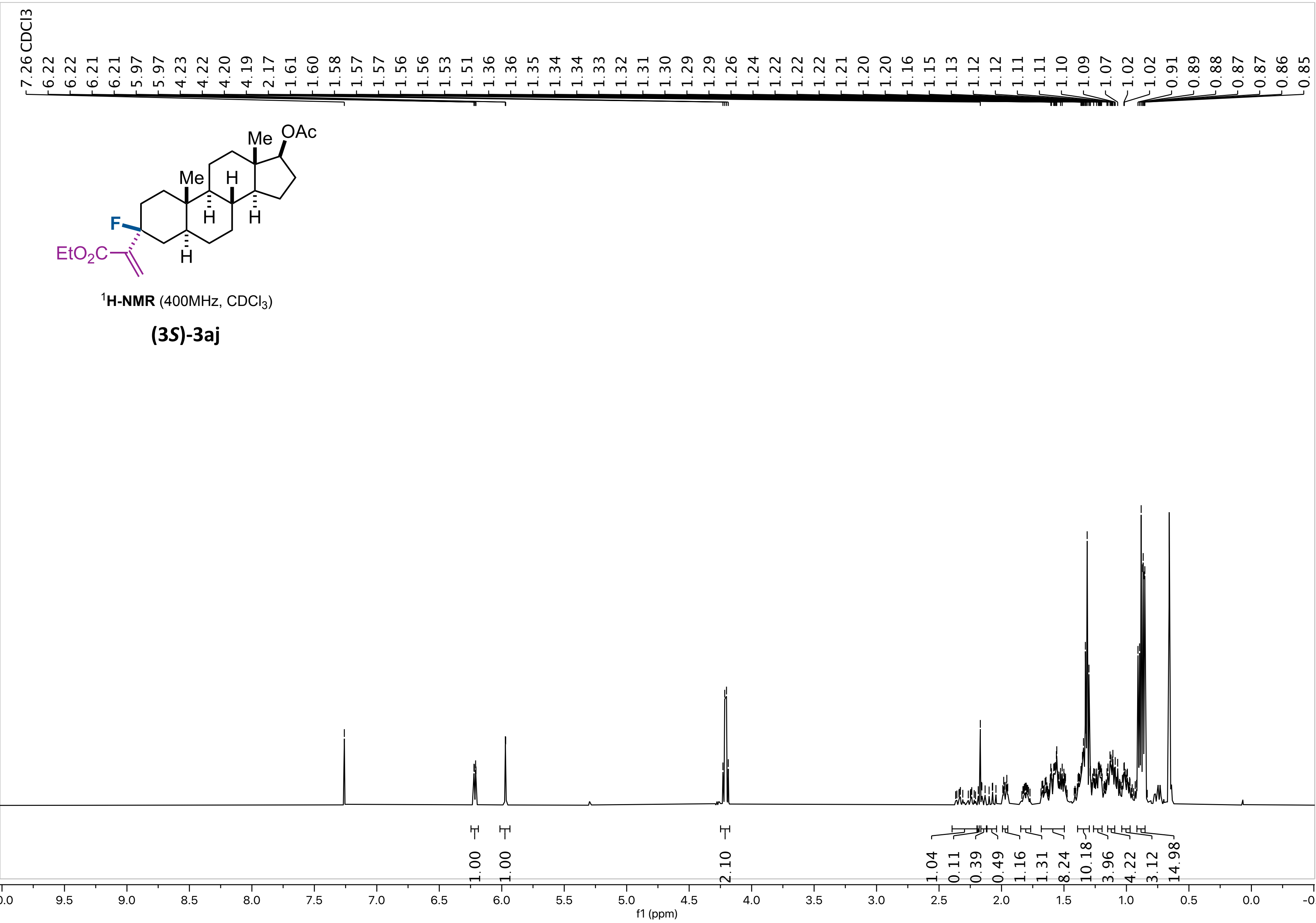
Me
OAc
Me
H
H
H
F
EtO2C
H
1H-NMR (400MHz, CDCl3)
(3S)-3aj
7.26 CDCl3
6.22
6.22
6.21
6.21
5.97
5.97
4.23
4.22
4.20
4.19
2.17
1.61
1.60
1.58
1.57
1.57
1.56
1.56
1.53
1.51
1.36
1.36
1.35
1.34
1.34
1.33
1.32
1.31
1.30
1.29
1.29
1.26
1.24
1.22
1.22
1.22
1.21
1.20
1.20
1.16
1.15
1.13
1.12
1.12
1.11
1.11
1.10
1.09
1.07
1.02
1.02
0.91
0.89
0.88
0.87
0.87
0.86
0.85
1.00
1.00
2.10
1.04
0.11
0.39
0.49
1.16
1.31
8.24
10.18
3.96
4.22
3.12
14.98
9.5
9.0
8.5
8.0
7.5
7.0
6.5
6.0
5.5
5.0
4.5
4.0
3.5
3.0
2.5
2.0
1.5
1.0
0.5
0.0
f1 (ppm)

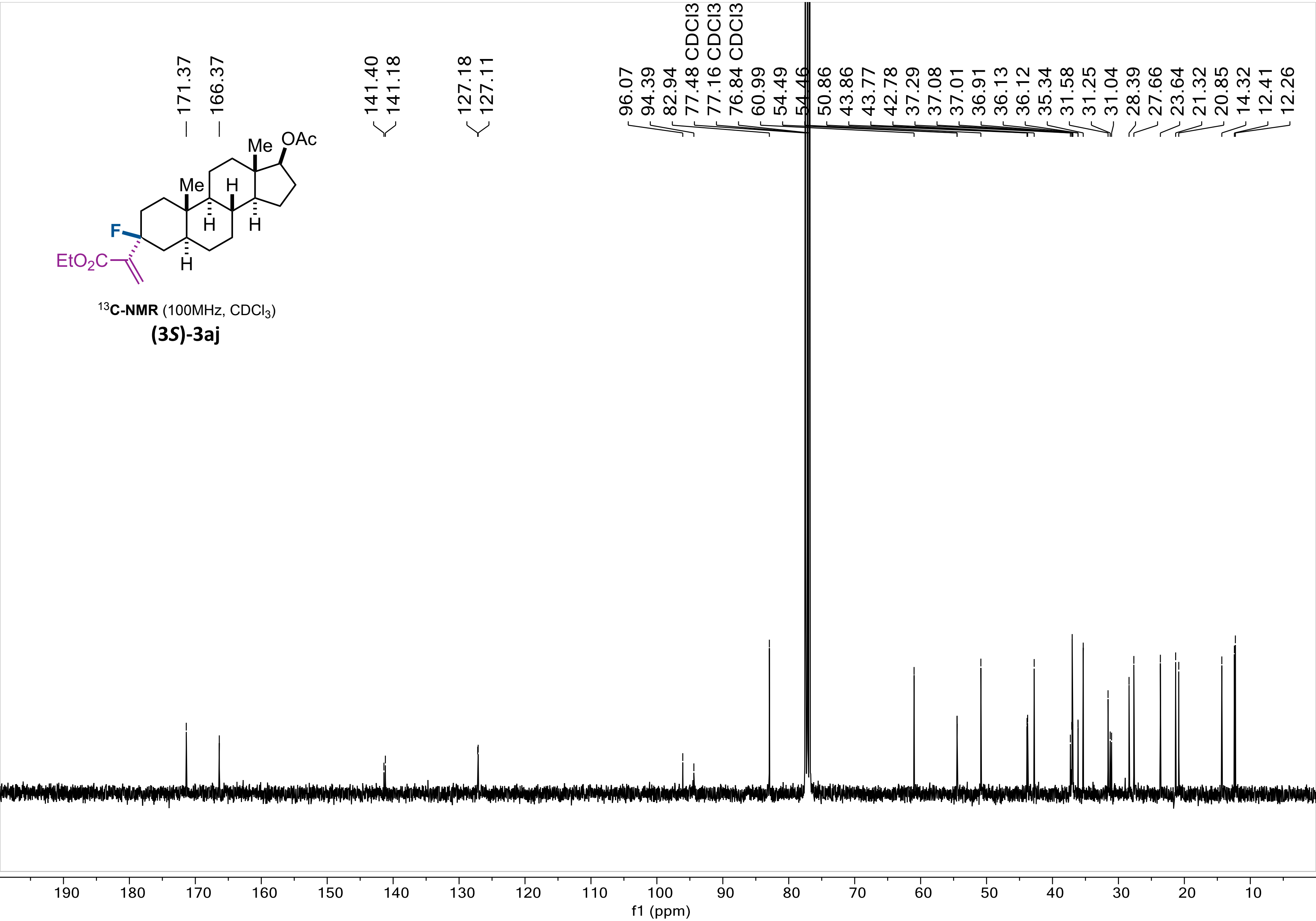
171.37
166.37
141.40
141.18
127.18
127.11
96.07
94.39
82.94
77.48 CDCl3
77.16 CDCl3
76.84 CDCl3
60.99
54.49
54.46
50.86
43.86
43.77
42.78
37.29
37.08
37.01
36.91
36.13
36.12
35.34
31.58
31.25
31.04
28.39
27.66
23.64
21.32
20.85
14.32
12.41
12.26
OAc
Me
Me
H
H
H
F
EtO2C
H
13C-NMR (100MHz, CDCl3)
(3S)-3aj
190
180
170
160
150
140
130
120
110
100
90
80
70
60
50
40
30
20
10
f1 (ppm)

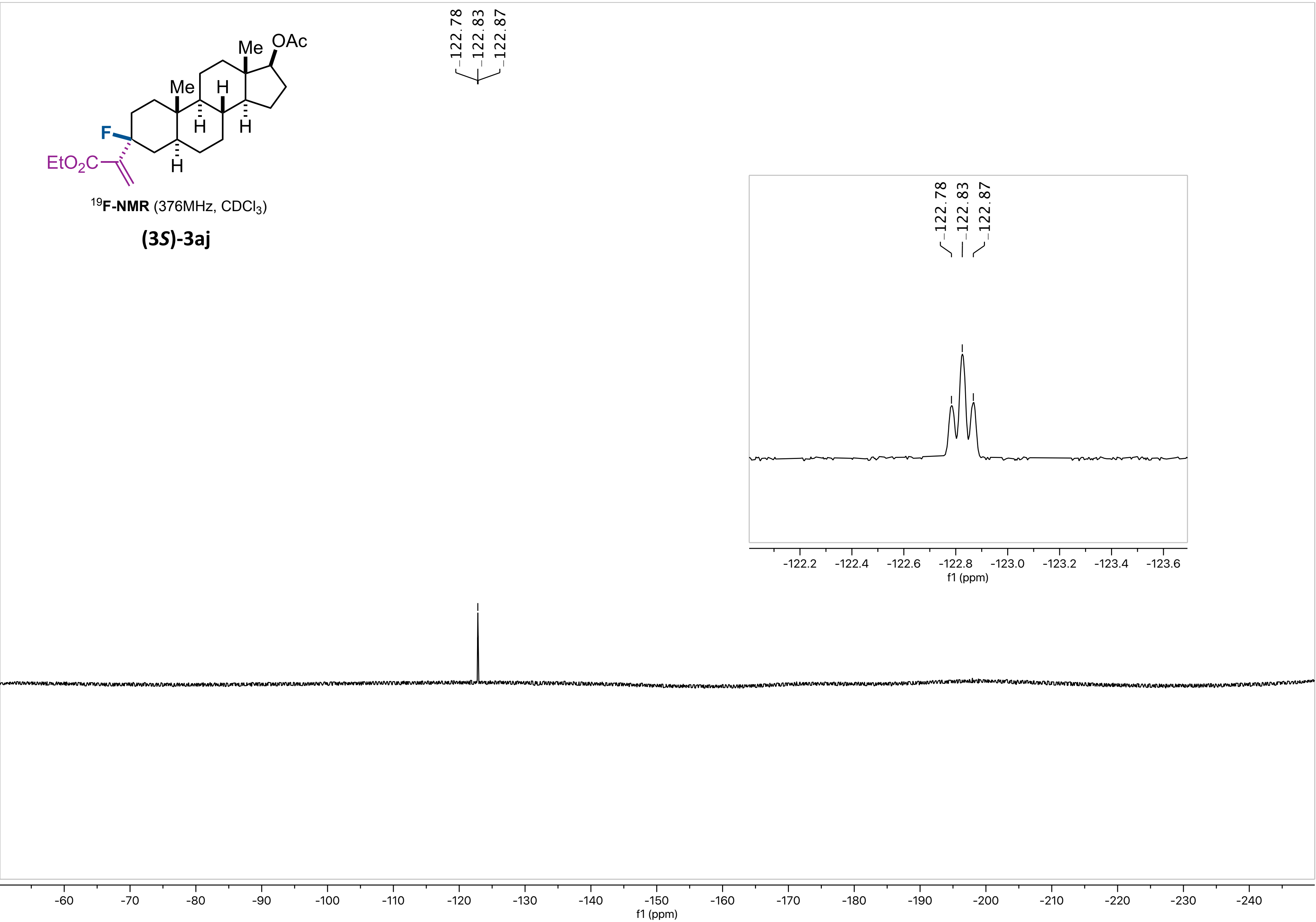

^{19}**F-NMR** (376MHz, $CDCl_3$)

(3*S*)-3aj

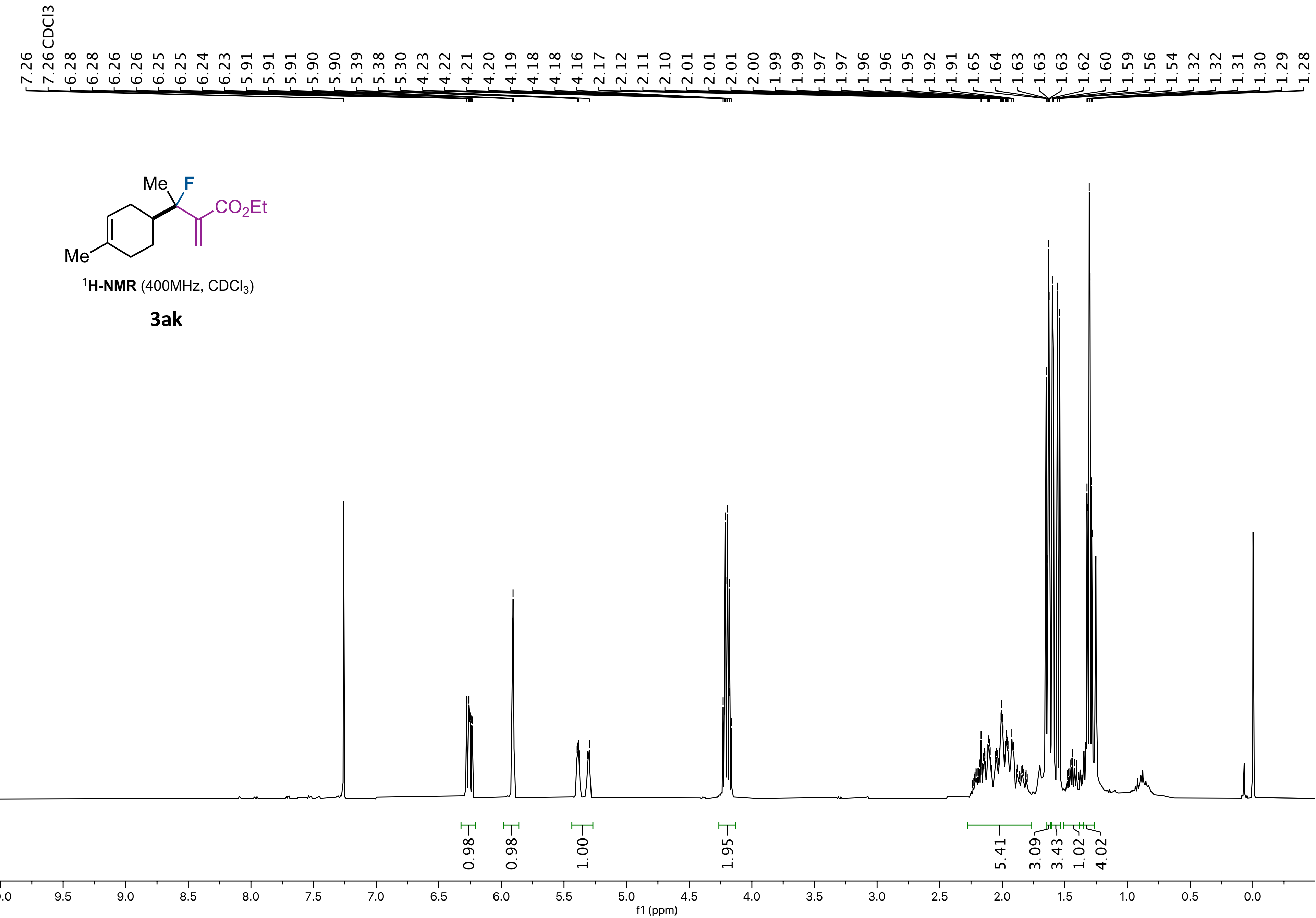

Me
F
CO2Et
Me
1H-NMR (400MHz, CDCl3)
3ak
7.26 CDCl3
7.26
6.28
6.28
6.26
6.26
6.25
6.25
6.24
6.23
5.91
5.91
5.91
5.90
5.90
5.39
5.38
5.30
4.23
4.22
4.21
4.20
4.19
4.18
4.18
4.16
2.17
2.12
2.11
2.10
2.01
2.01
2.01
2.00
1.99
1.99
1.97
1.97
1.96
1.96
1.95
1.92
1.91
1.65
1.64
1.63
1.63
1.63
1.62
1.60
1.59
1.56
1.54
1.32
1.32
1.31
1.30
1.29
1.28
0.98
0.98
1.00
1.95
5.41
3.09
3.43
1.02
4.02
9.5
9.0
8.5
8.0
7.5
7.0
6.5
6.0
5.5
5.0
4.5
4.0
3.5
3.0
2.5
2.0
1.5
1.0
0.5
0.0
f1 (ppm)

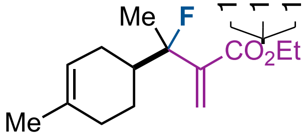

^{13}**C-NMR** (101MHz, $CDCl_3$)

3ak

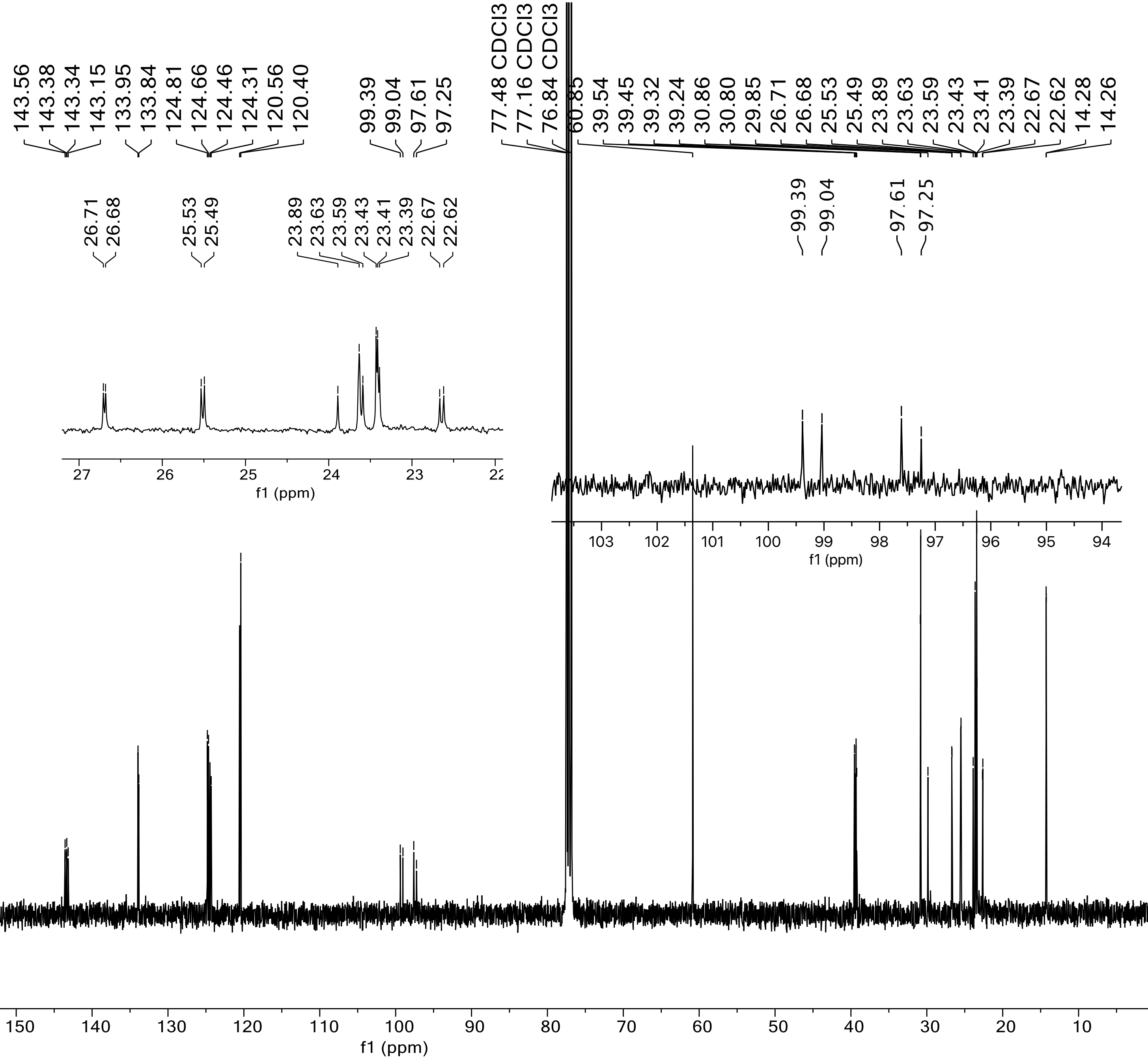

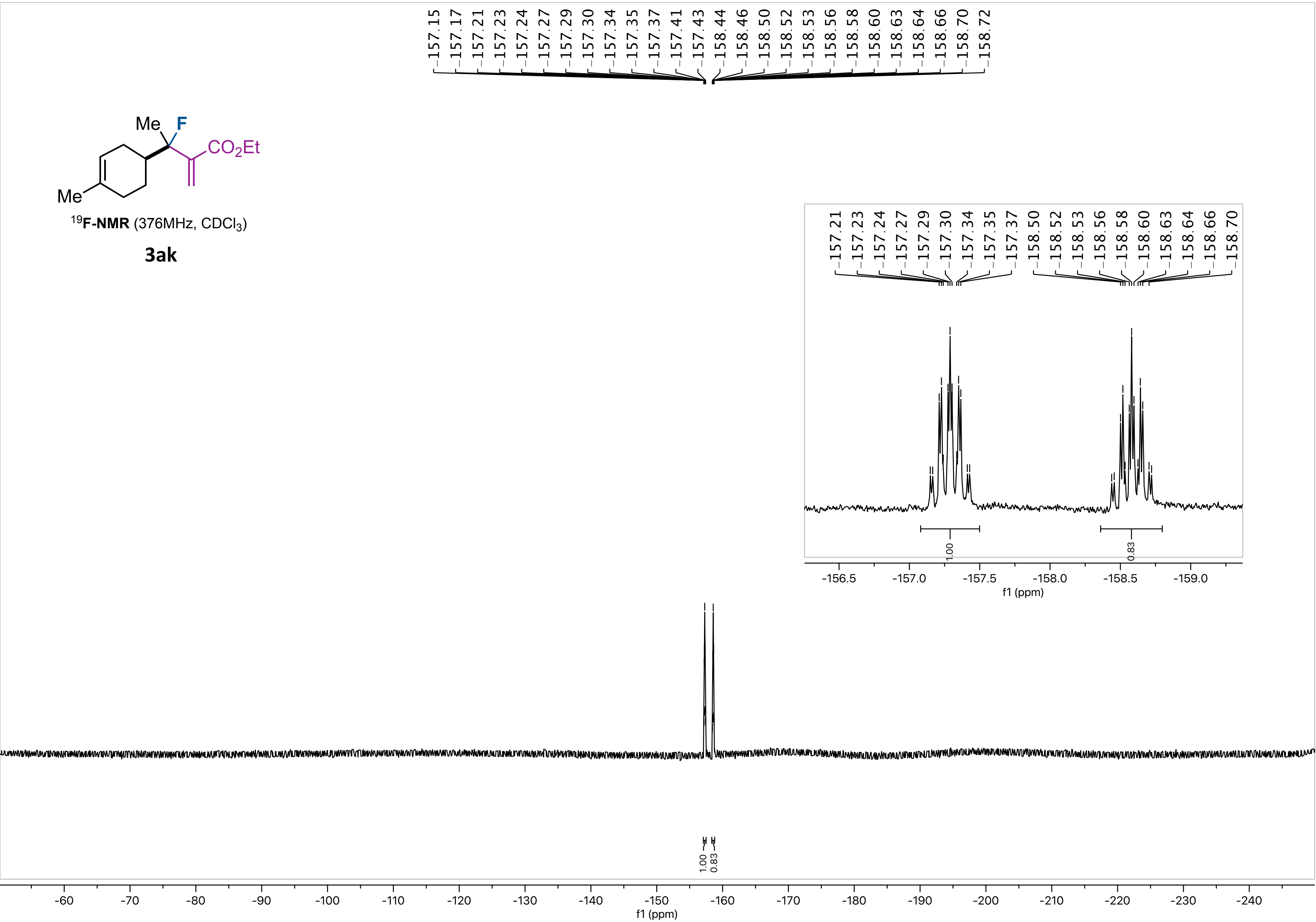

^{19}**F-NMR** (376MHz, $CDCl_3$)

3ak

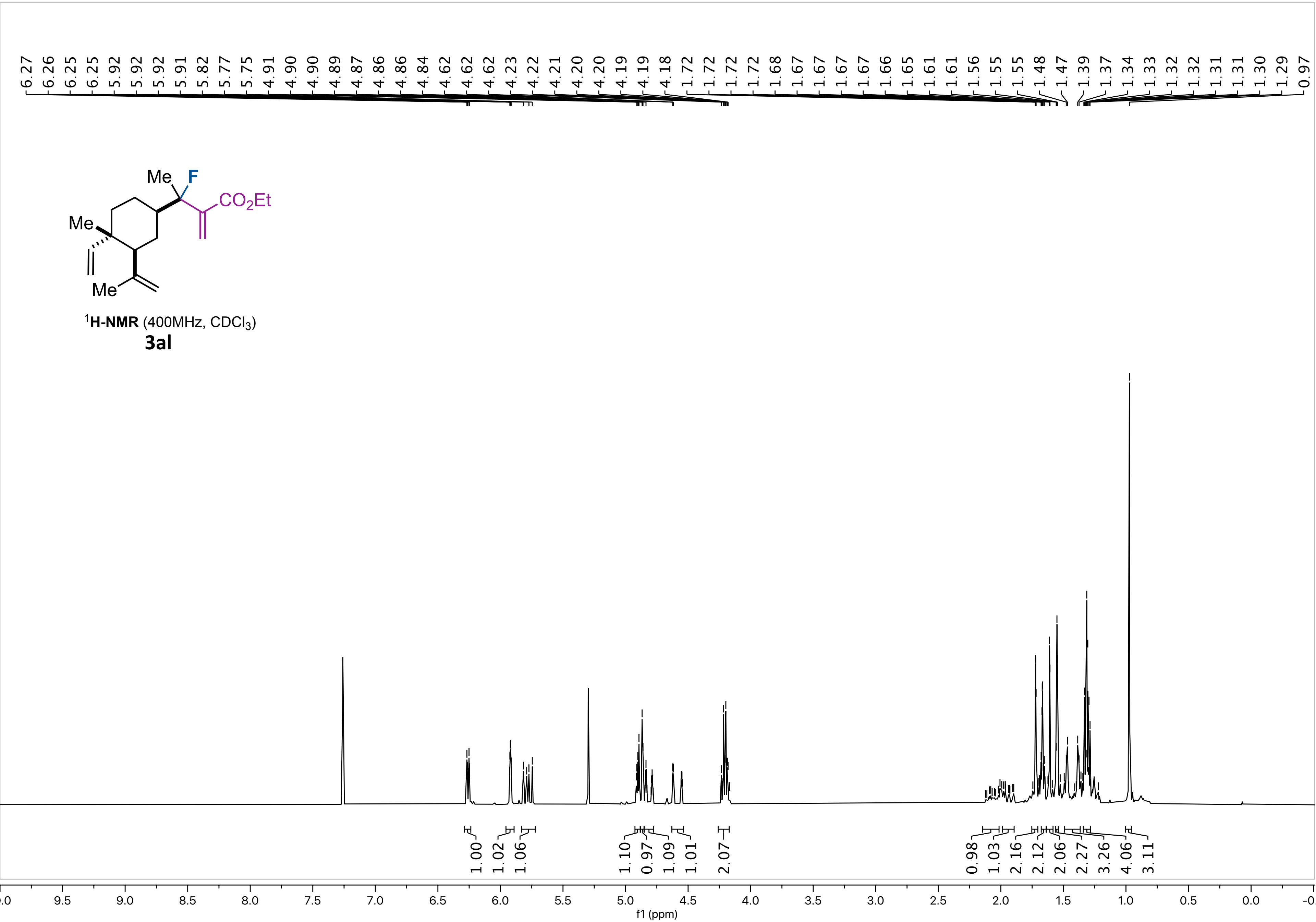
6.27 6.26 6.25 6.25 5.92 5.92 5.92 5.91 5.82 5.77 5.75 4.91 4.90 4.90 4.89 4.87 4.86 4.86 4.84 4.62 4.62 4.62 4.23 4.22 4.21 4.20 4.20 4.19 4.19 4.18 1.72 1.72 1.72 1.72 1.68 1.67 1.67 1.67 1.67 1.66 1.65 1.61 1.61 1.56 1.55 1.55 1.48 1.47 1.39 1.37 1.34 1.33 1.32 1.32 1.31 1.31 1.30 1.29 0.97
Me
F
CO_2Et
Me
Me
^{1}H-NMR (400MHz, $CDCl_3$)
3al
1.00 1.02 1.06 1.10 0.97 1.09 1.01 2.07 0.98 1.03 2.16 2.12 2.06 2.27 3.26 4.06 3.11
9.5 9.0 8.5 8.0 7.5 7.0 6.5 6.0 5.5 5.0 4.5 4.0 3.5 3.0 2.5 2.0 1.5 1.0 0.5 0.0
f1 (ppm)

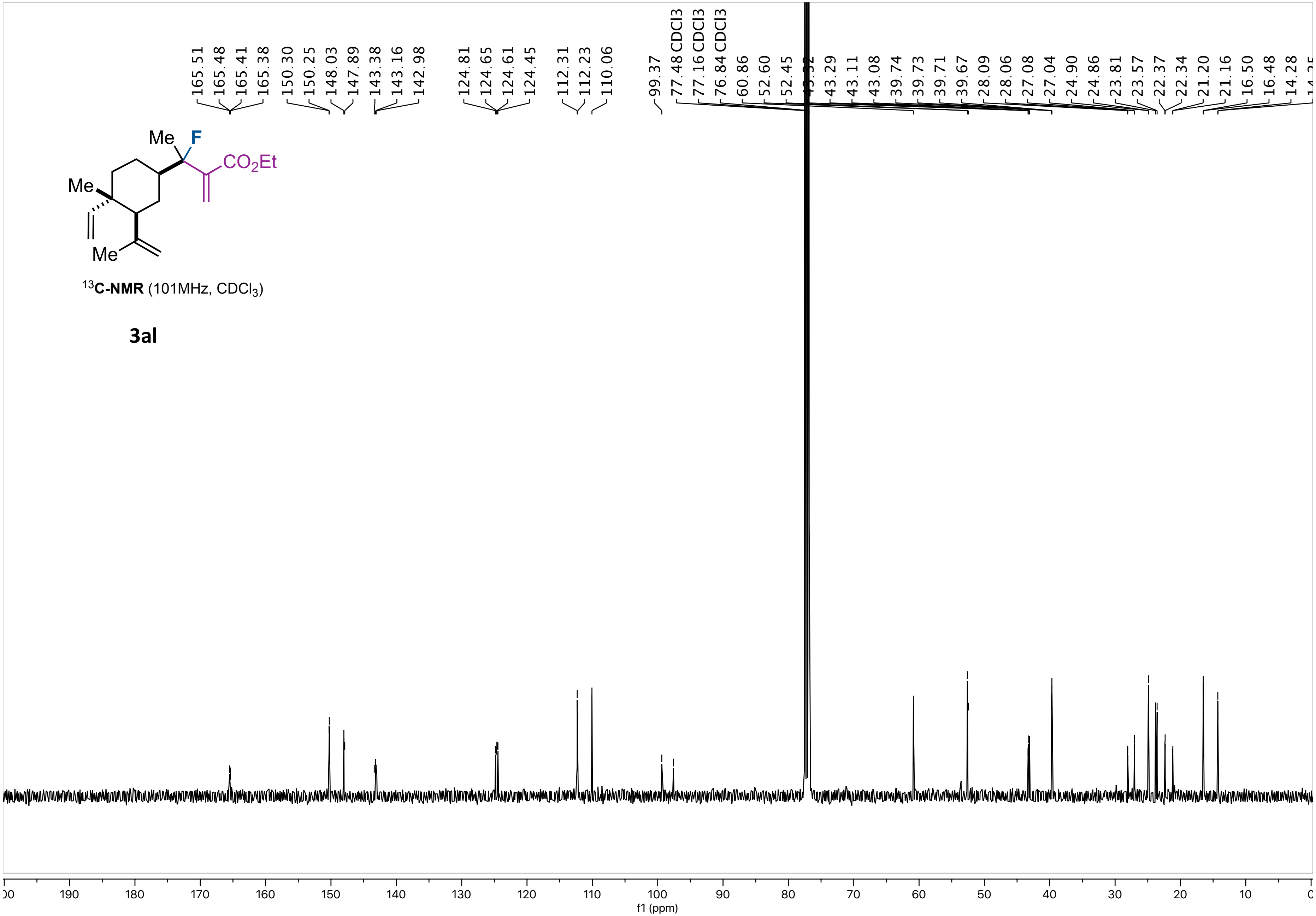

^{13}**C-NMR** (101MHz, $CDCl_3$)

3al

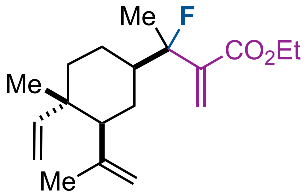

^{19}**F-NMR** (376MHz, $CDCl_3$)

3al

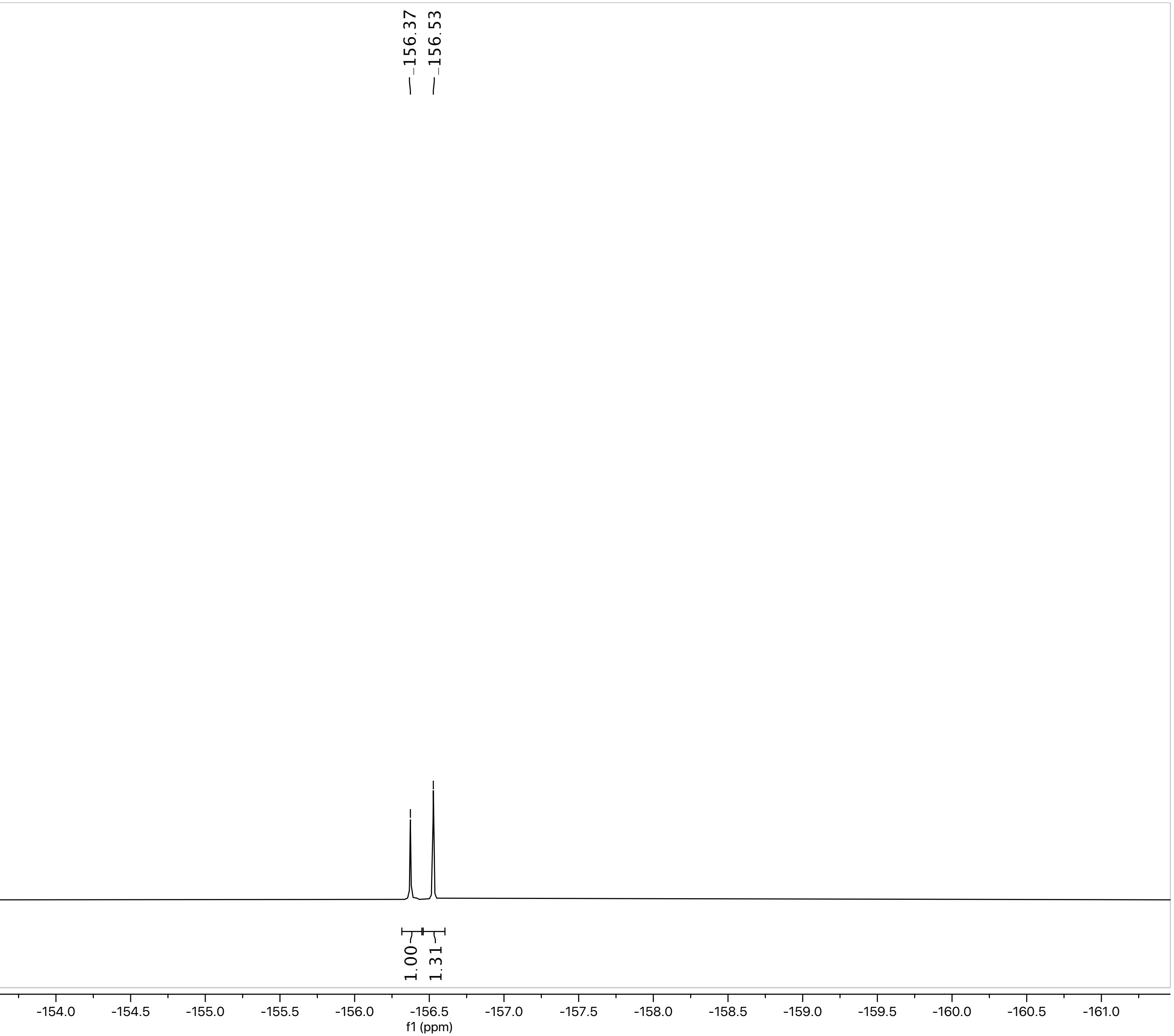

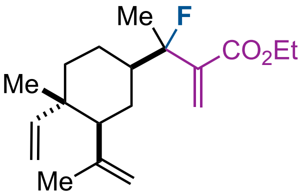

^{19}**F-NMR** (376MHz, $CDCl_3$)

3al

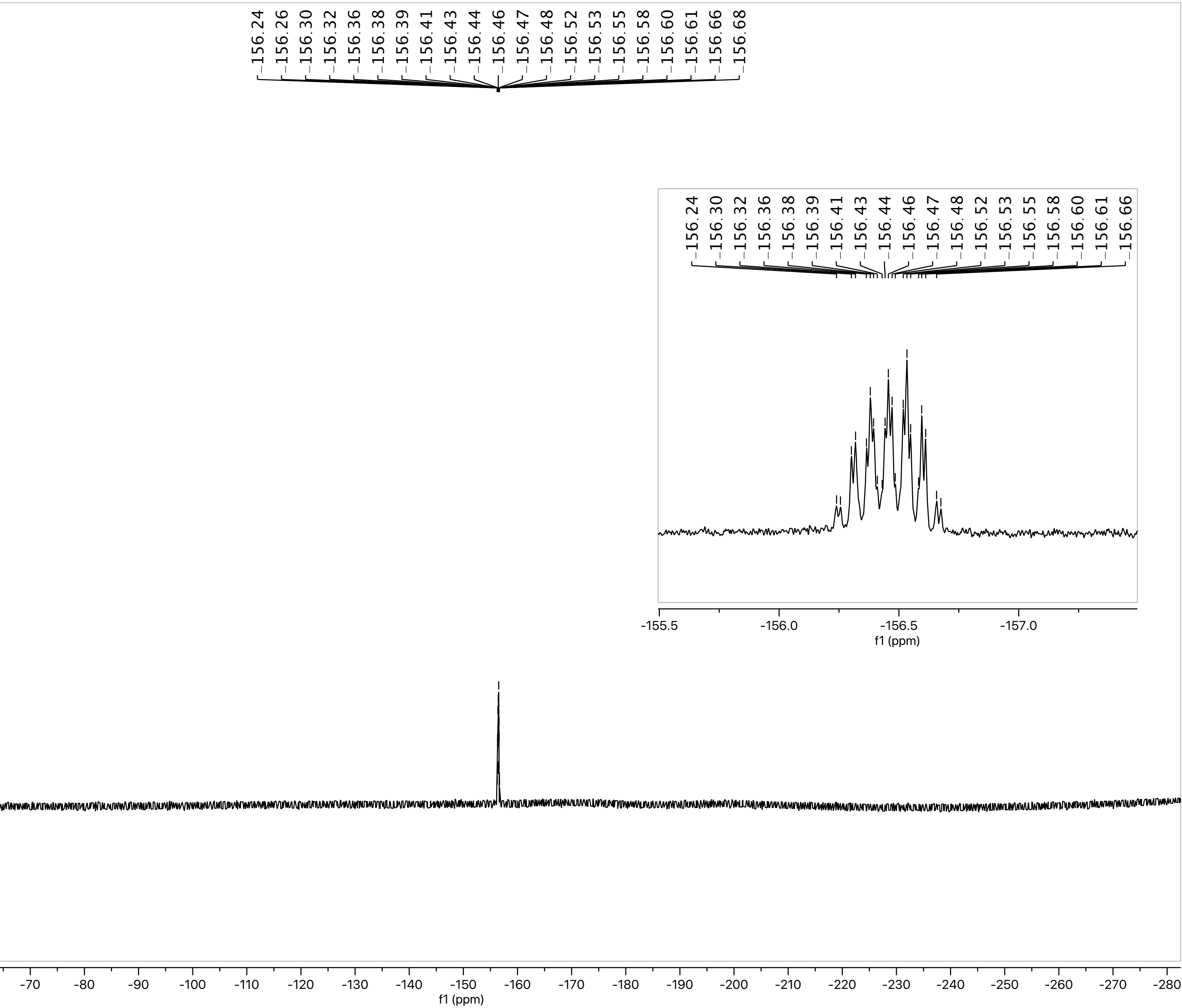

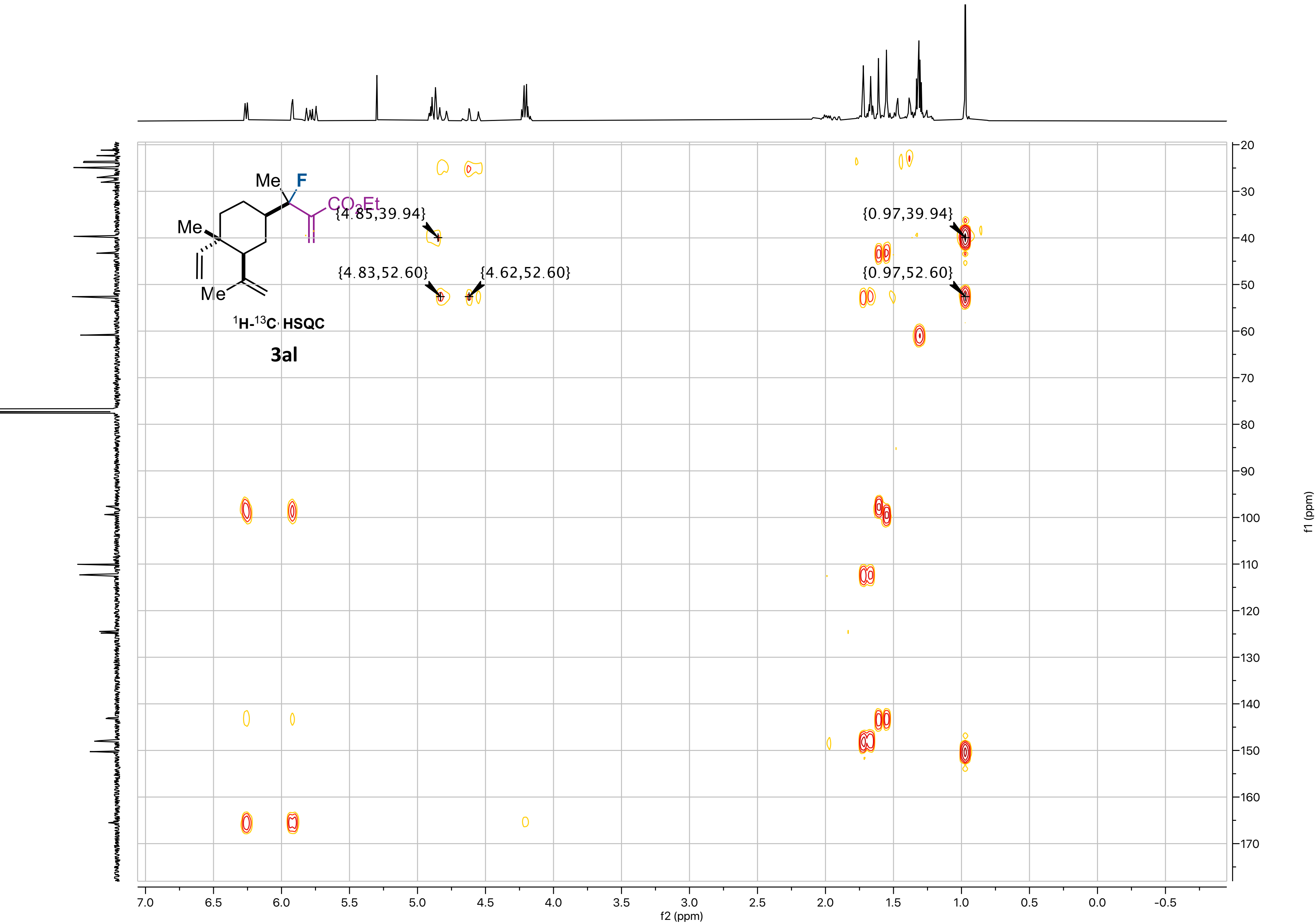

Me
F
CO2Et
Me
Me
1H-13C HSQC
3al
{4.85,39.94}
{0.97,39.94}
{4.83,52.60}
{4.62,52.60}
{0.97,52.60}
f2 (ppm)
f1 (ppm)

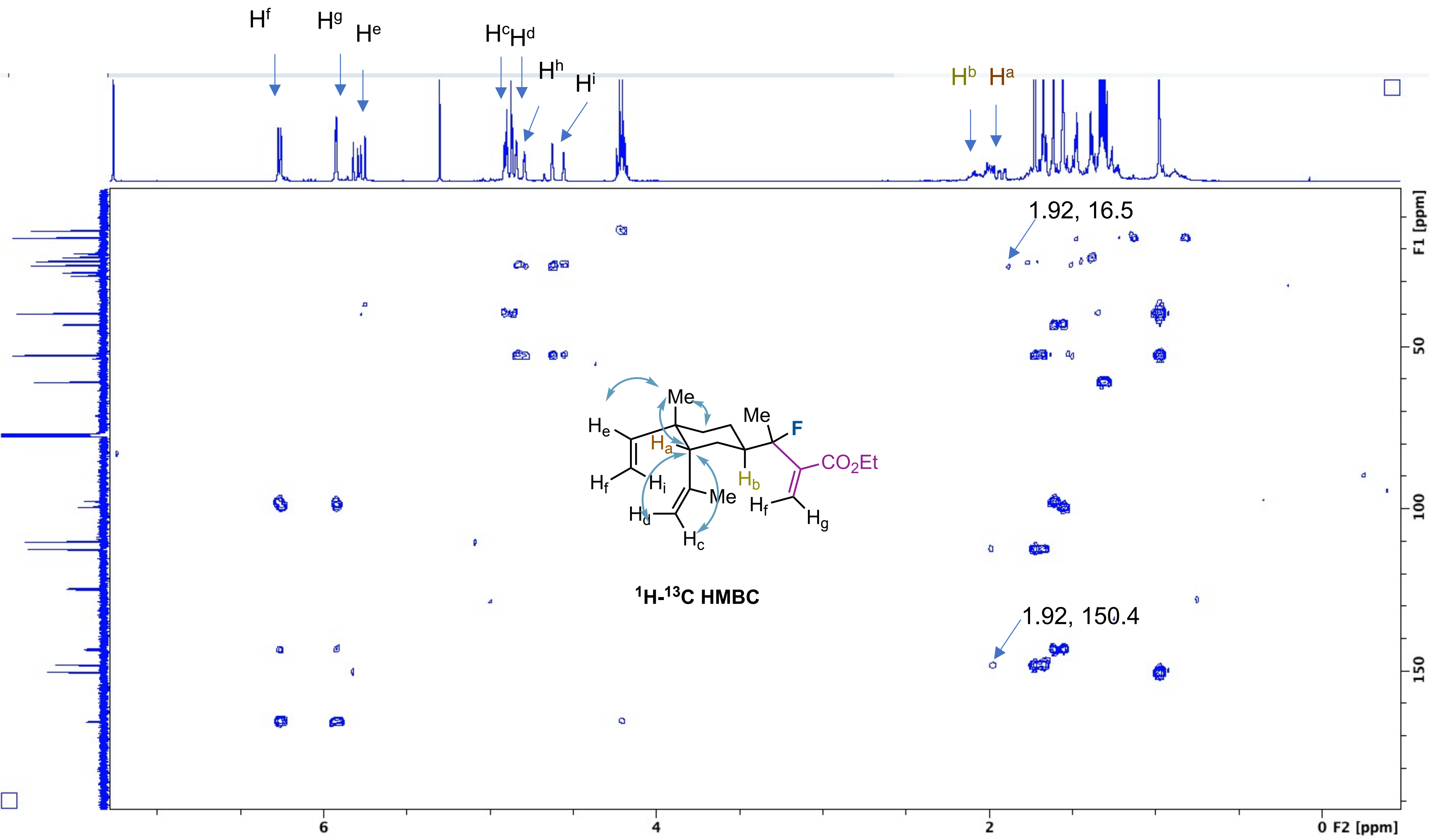
Hf
Hg
He
HcHd
Hh
Hi
Hb
Ha
1.92, 16.5
1.92, 150.4
Me
Me
F
CO2Et
Me
1H-13C HMBC
F1 [ppm]
F2 [ppm]

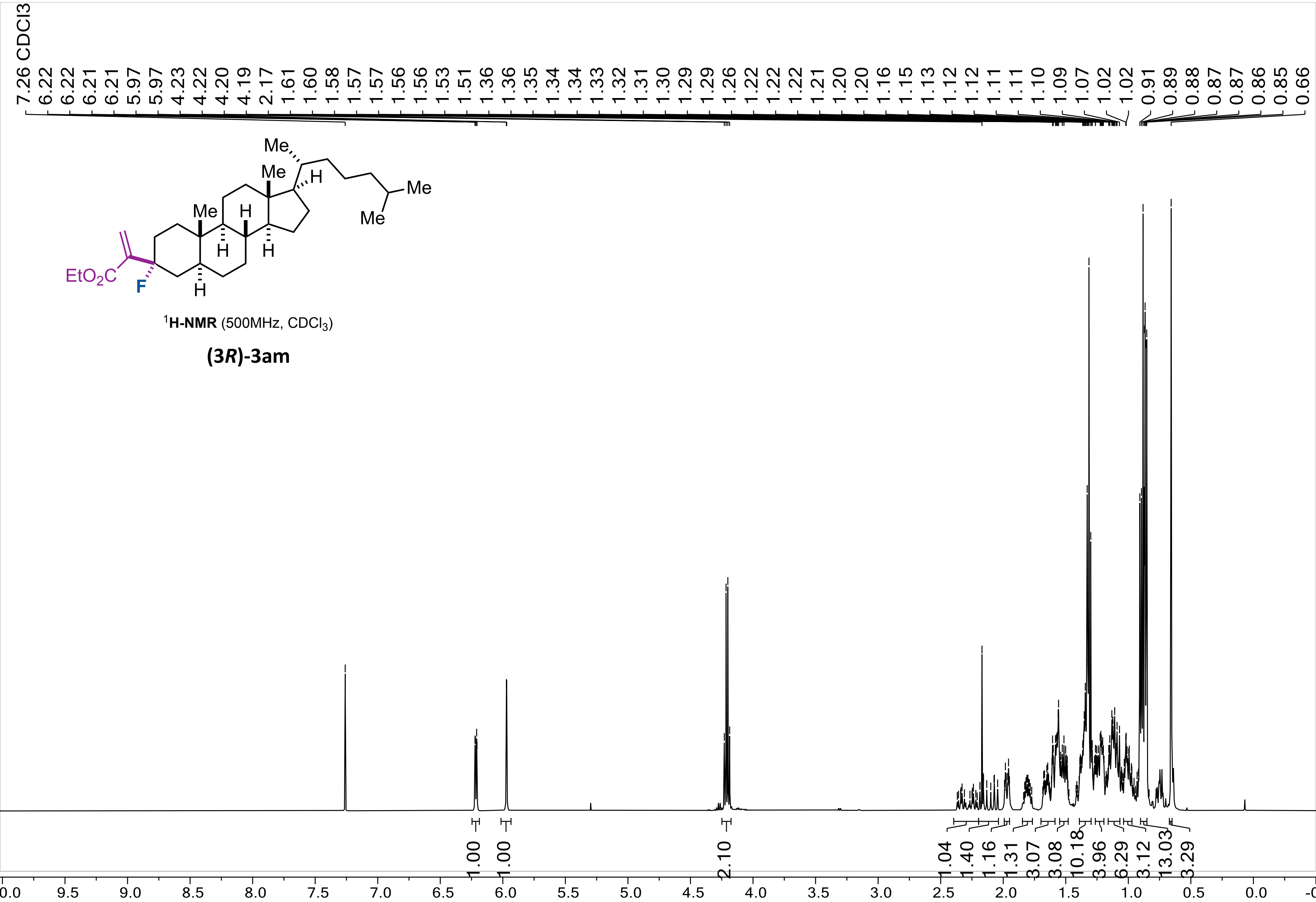

[1]**H-NMR** (500MHz, $CDCl_3$)

(3*R*)-3am

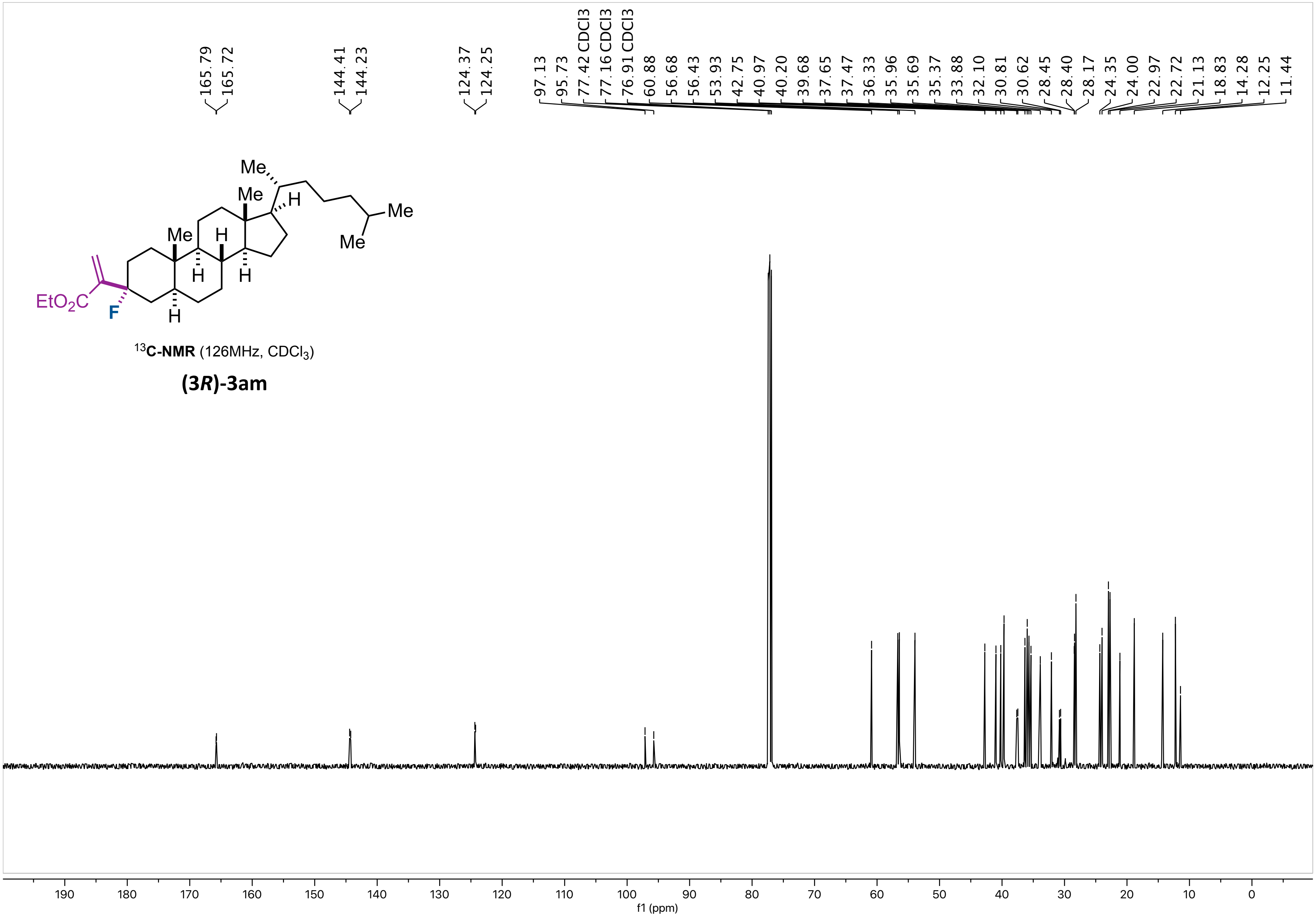
165.79
165.72
144.41
144.23
124.37
124.25
97.13
95.73
77.42 CDCl3
77.16 CDCl3
76.91 CDCl3
60.88
56.68
56.43
53.93
42.75
40.97
40.20
39.68
37.65
37.47
36.33
35.96
35.69
35.37
33.88
32.10
30.81
30.62
28.45
28.40
28.17
24.35
24.00
22.97
22.72
21.13
18.83
14.28
12.25
11.44
Me
Me
H
Me
Me
Me
H
H
H
EtO2C
F
H
^{13}C-NMR (126MHz, CDCl$_3$)
(3R)-3am
f1 (ppm)

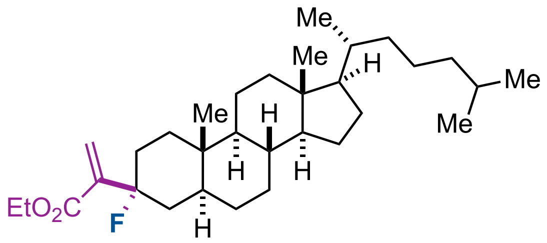

^{19}**F-NMR** (376MHz, $CDCl_3$)

(3*R*)-3am

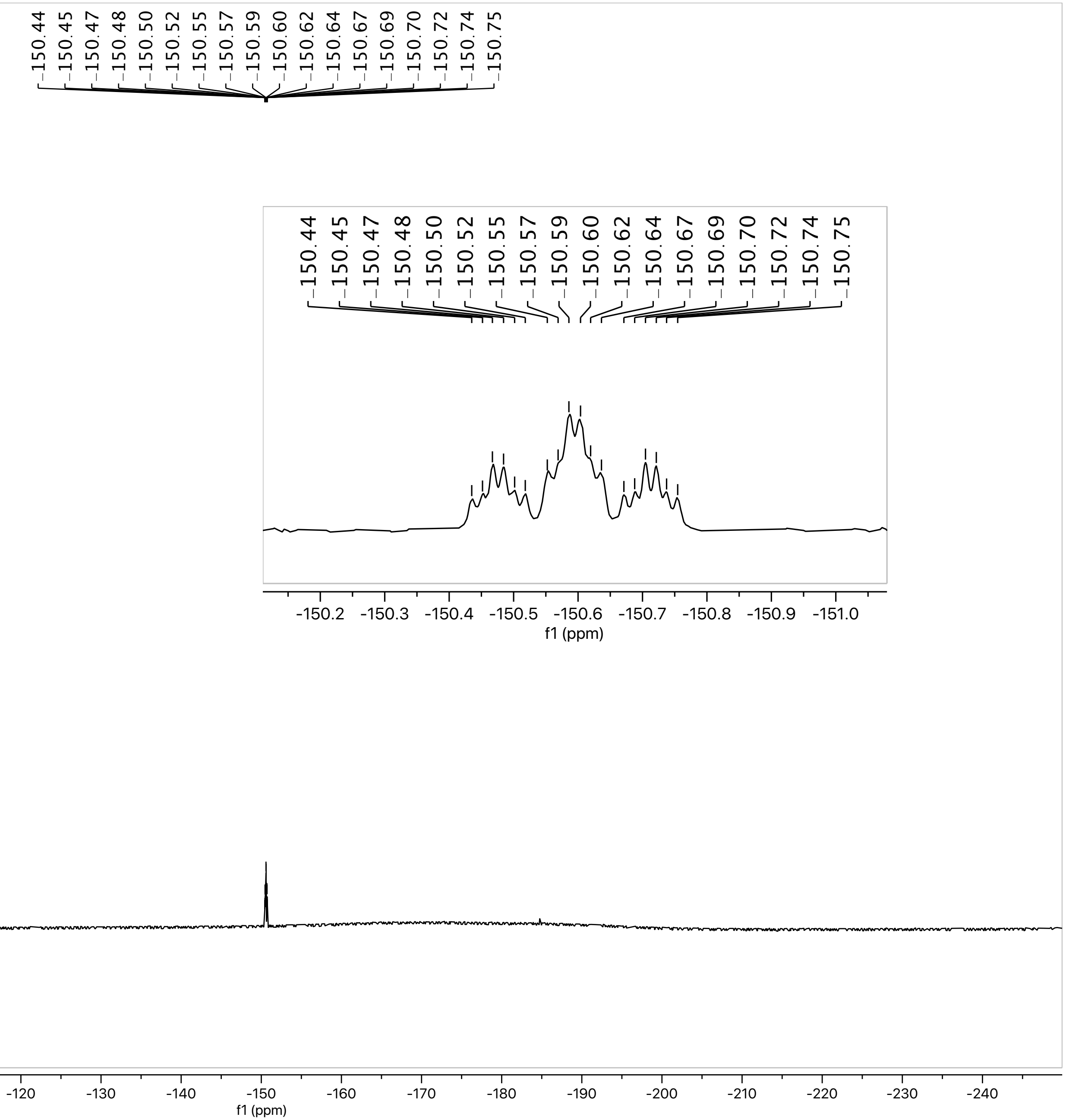

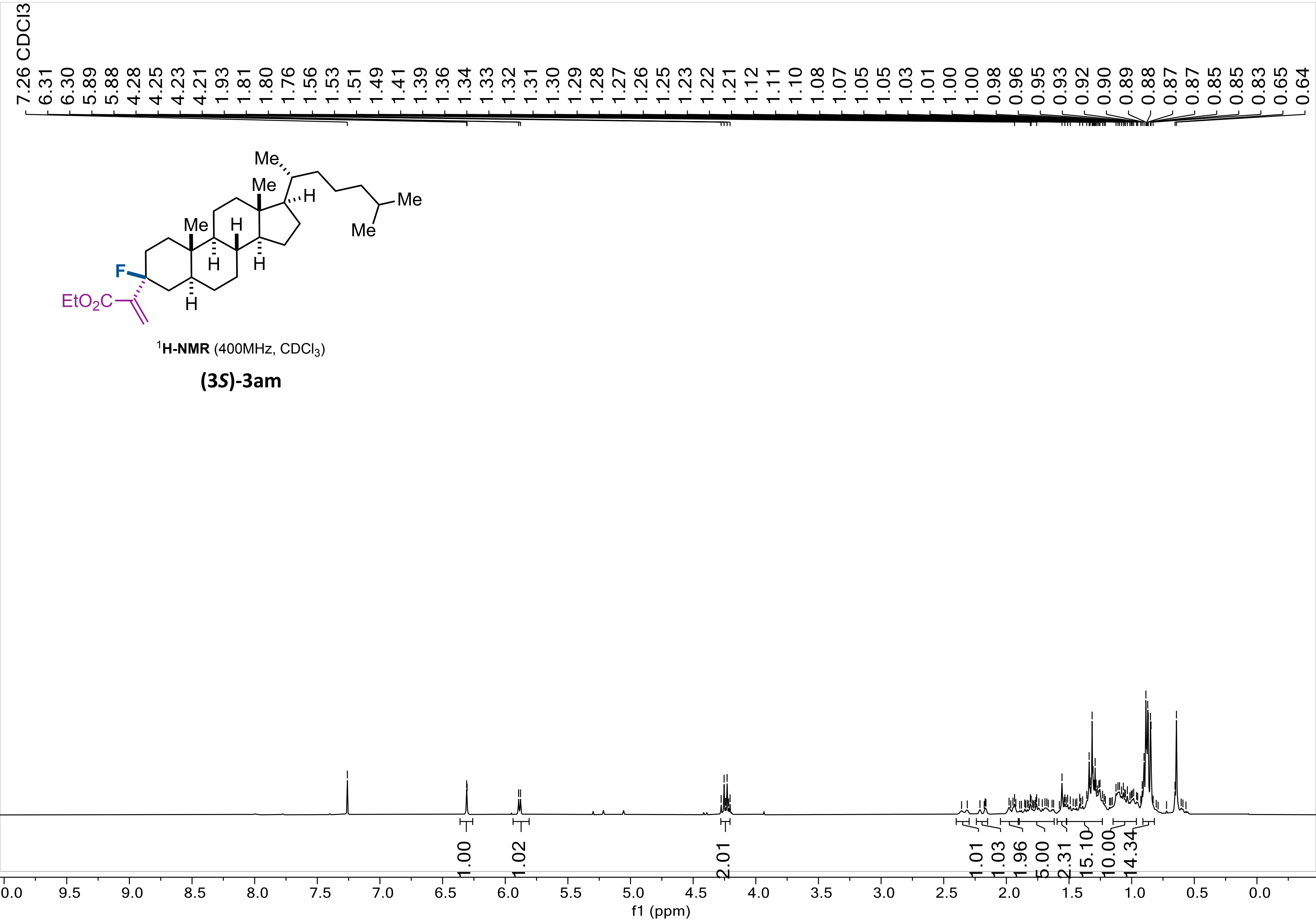

^{1}**H-NMR** (400MHz, $CDCl_3$)

(3*S*)-3am

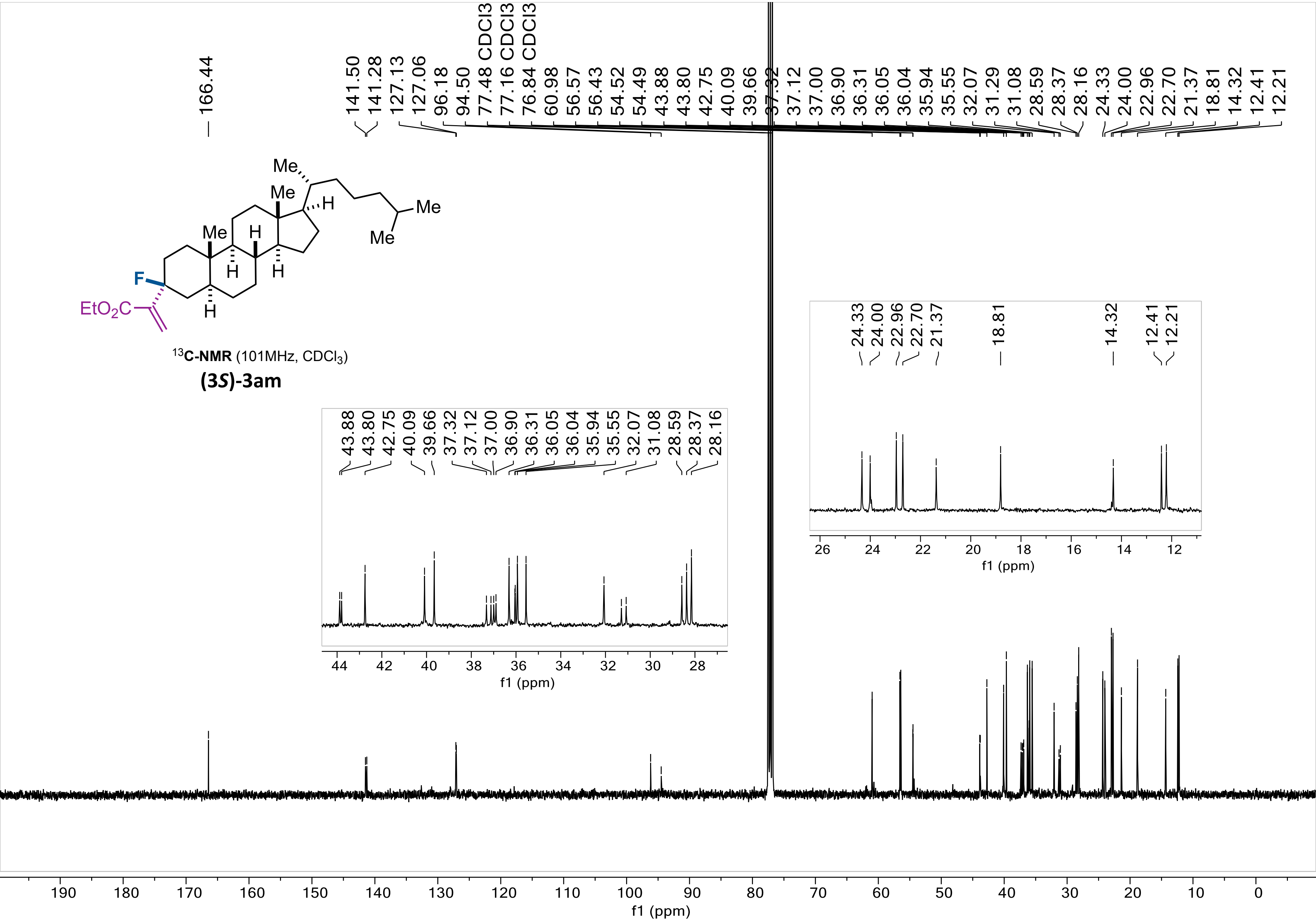

^{13}**C-NMR** (101MHz, $CDCl_3$)

(3*S*)-3am

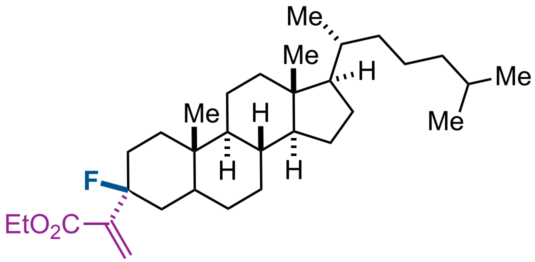

^{19}**F-NMR** (376MHz, $CDCl_3$)

(3*S*)-3am

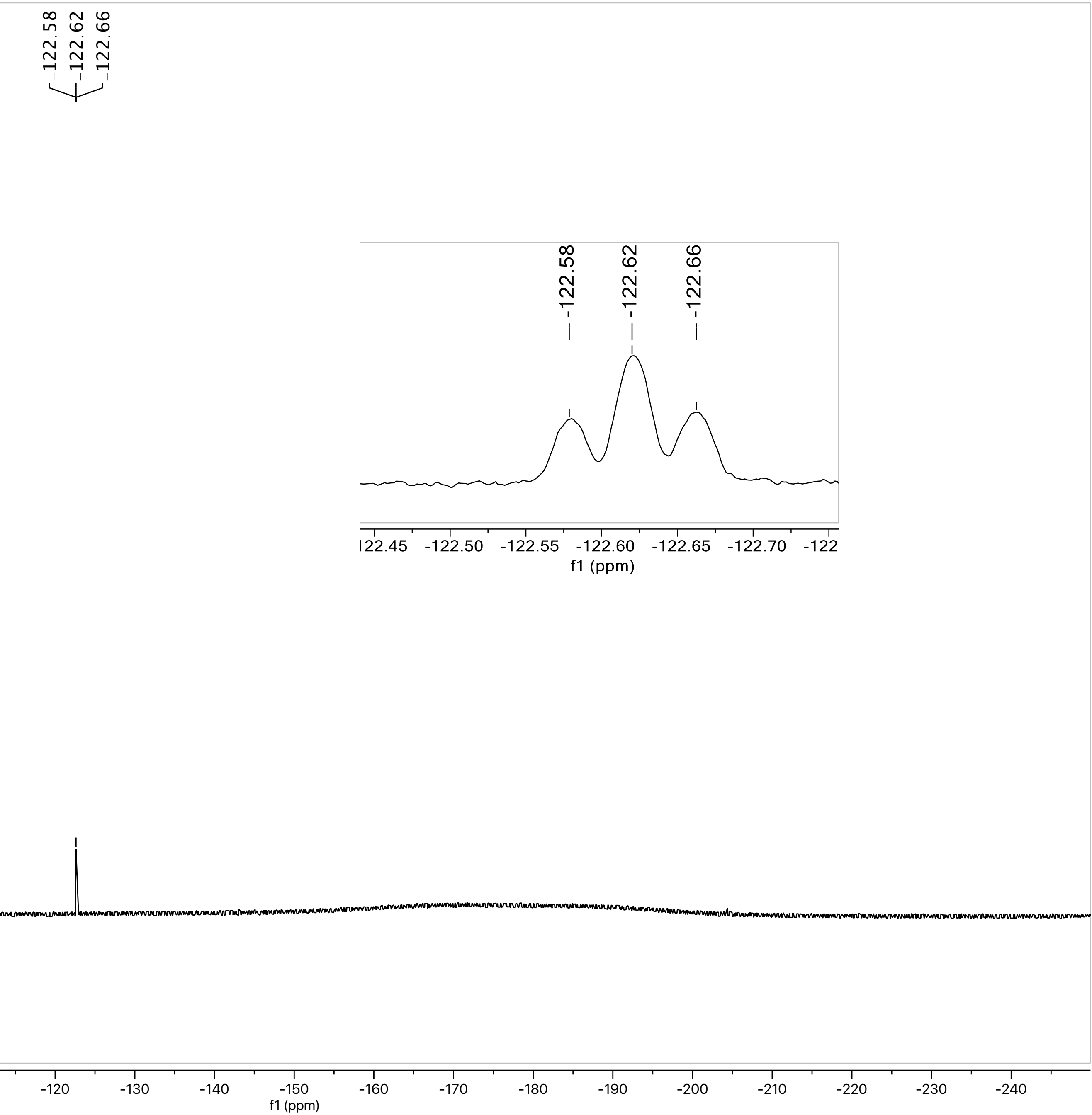

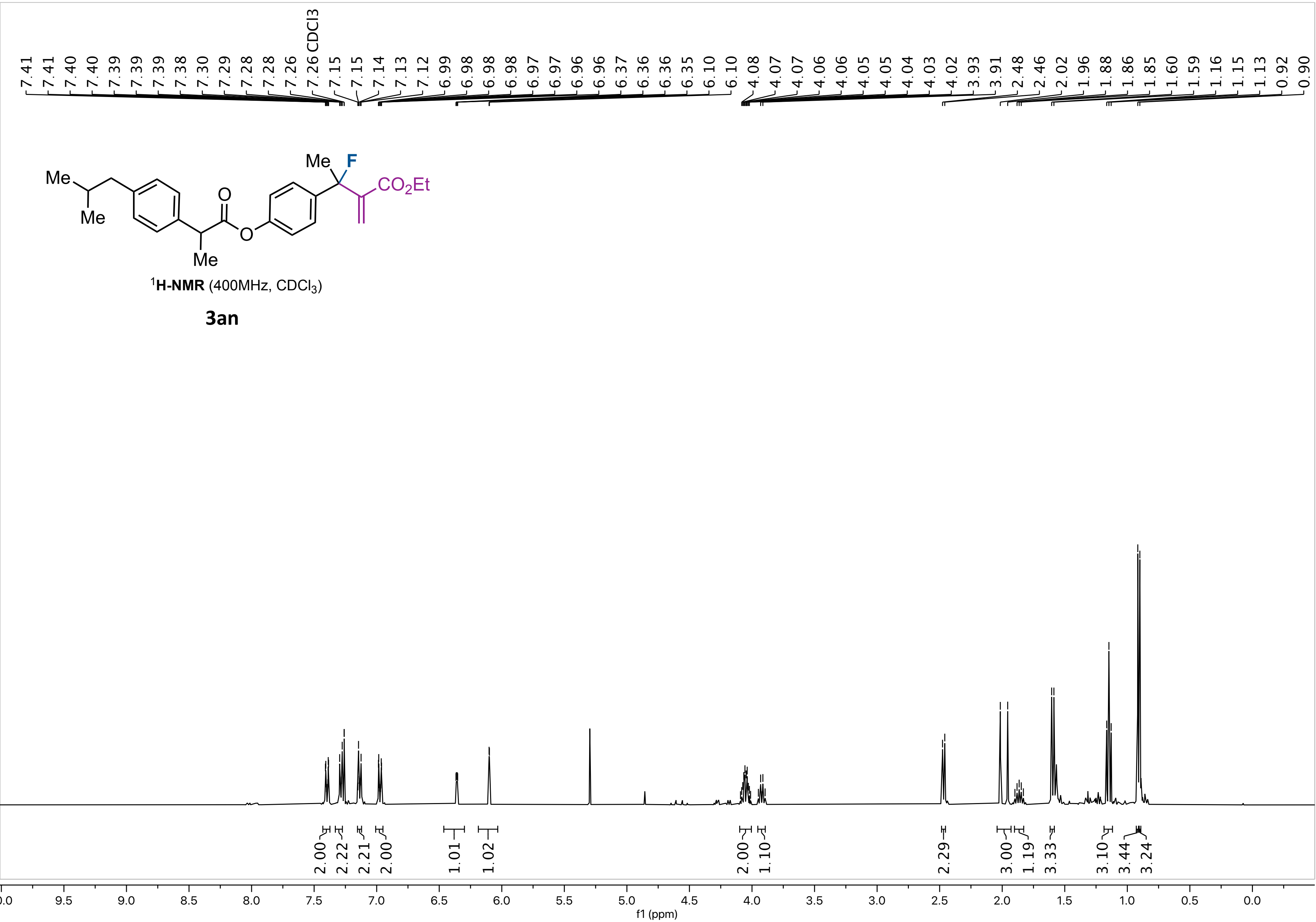
CDCl3
7.41 7.41 7.40 7.40 7.39 7.39 7.39 7.38 7.30 7.29 7.28 7.28 7.26 7.26 7.15 7.15 7.14 7.13 7.12 6.99 6.98 6.98 6.98 6.97 6.97 6.96 6.96 6.37 6.36 6.36 6.35 6.10 6.10 4.08 4.07 4.07 4.06 4.06 4.05 4.05 4.04 4.03 4.02 3.93 3.91 2.48 2.46 2.02 1.96 1.88 1.86 1.85 1.60 1.59 1.16 1.15 1.13 0.92 0.90
Me
F
CO2Et
Me
O
O
Me
Me
1H-NMR (400MHz, CDCl3)
3an
2.00 2.22 2.21 2.00 1.01 1.02 2.00 1.10 2.29 3.00 1.19 3.33 3.10 3.44 3.24
10.0 9.5 9.0 8.5 8.0 7.5 7.0 6.5 6.0 5.5 5.0 4.5 4.0 3.5 3.0 2.5 2.0 1.5 1.0 0.5 0.0
f1 (ppm)

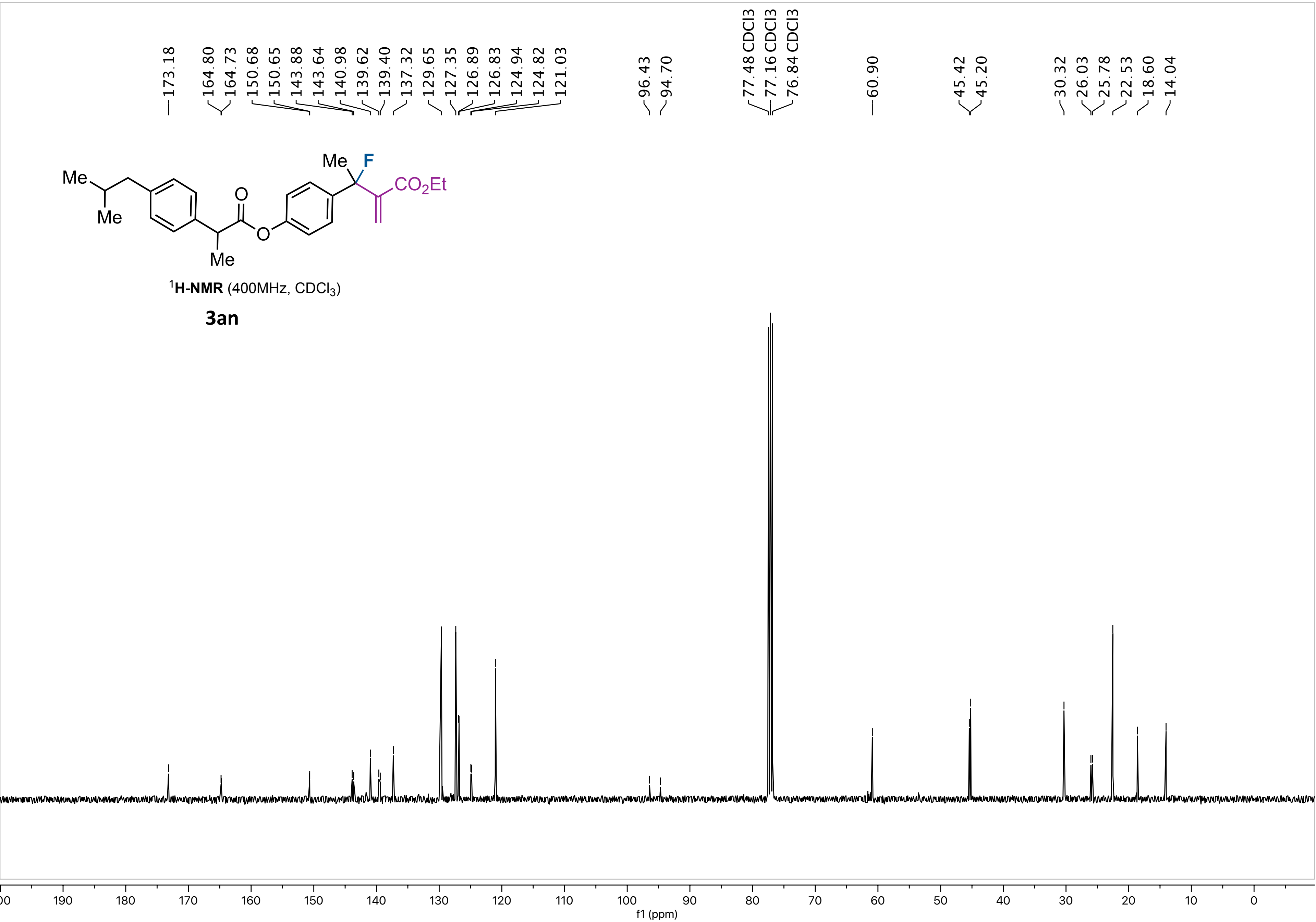

[1]**H-NMR** (400MHz, $CDCl_3$)

3an

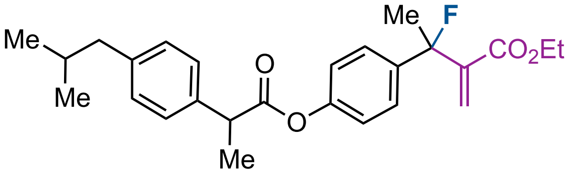

^{19}**F-NMR** (376MHz, $CDCl_3$)

3an

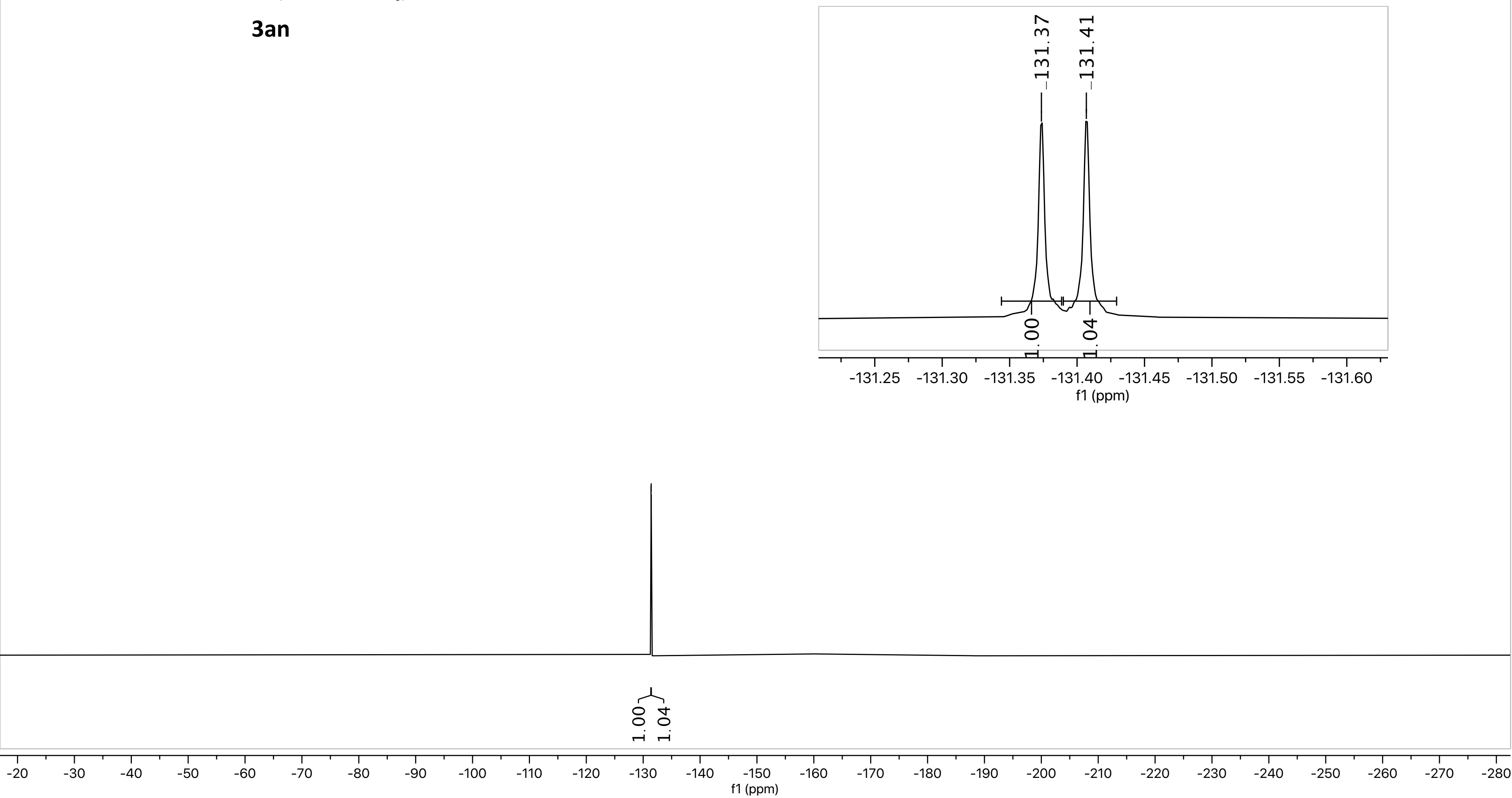

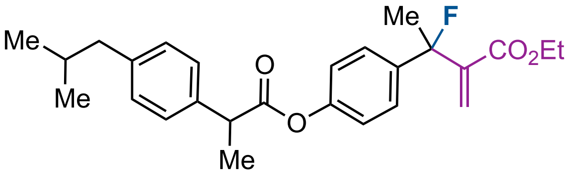

^{19}**F-NMR** (376MHz, $CDCl_3$)

3an

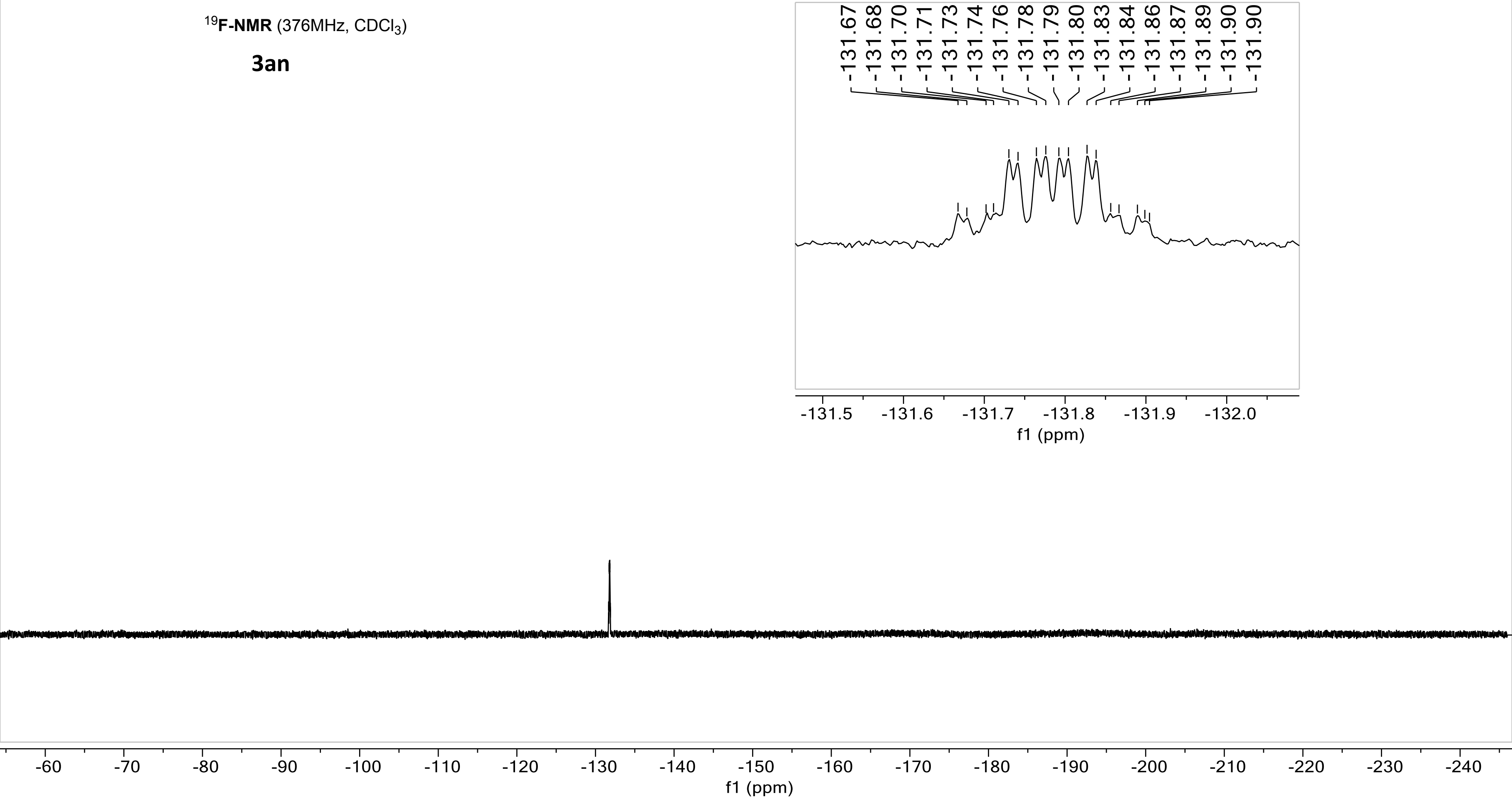

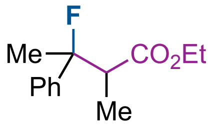

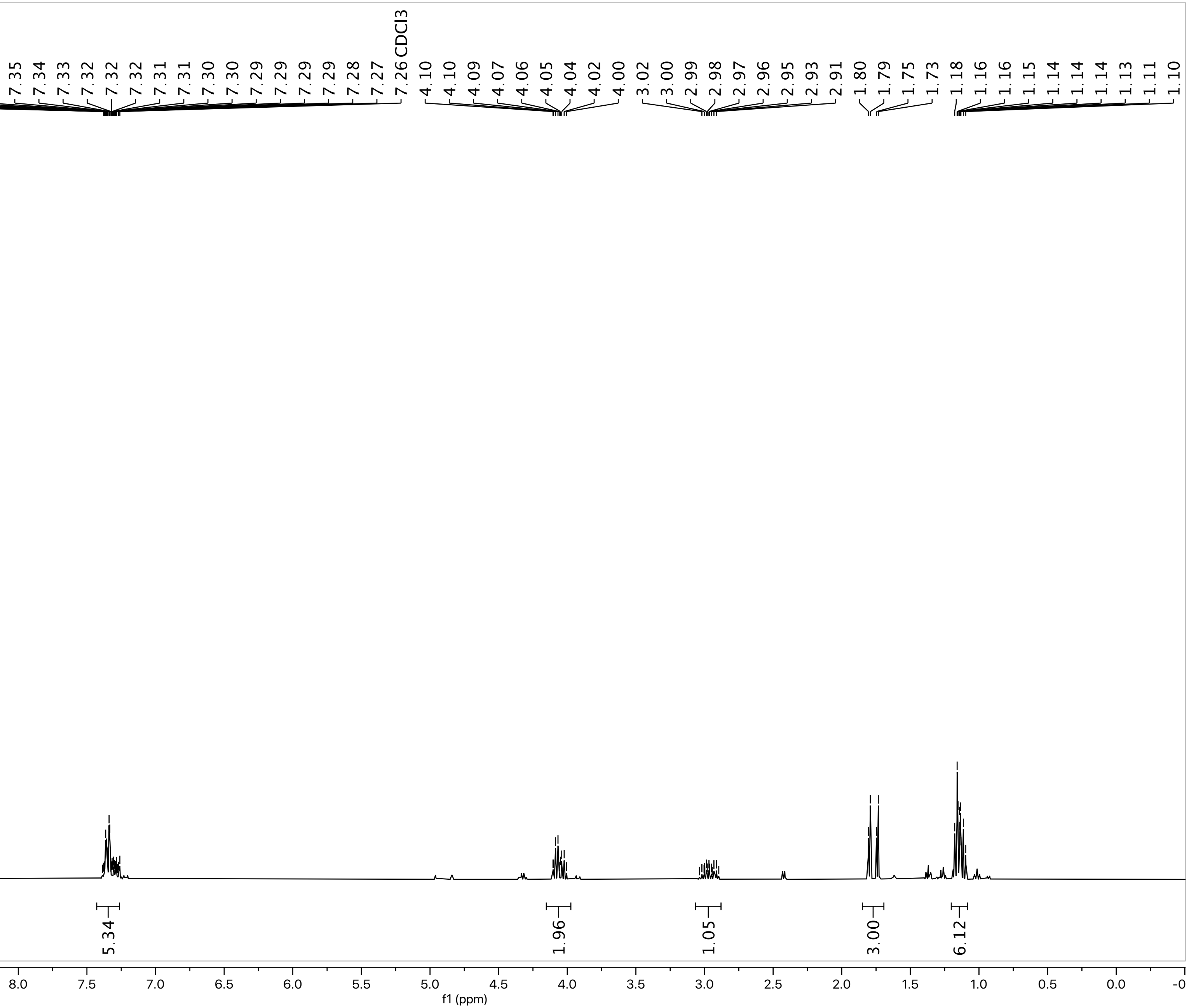

^{1}H-NMR (400MHz, $CDCl_3$)

6

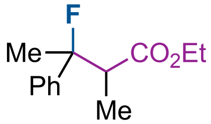

^{13}**C-NMR** (101MHz, $CDCl_3$)

6

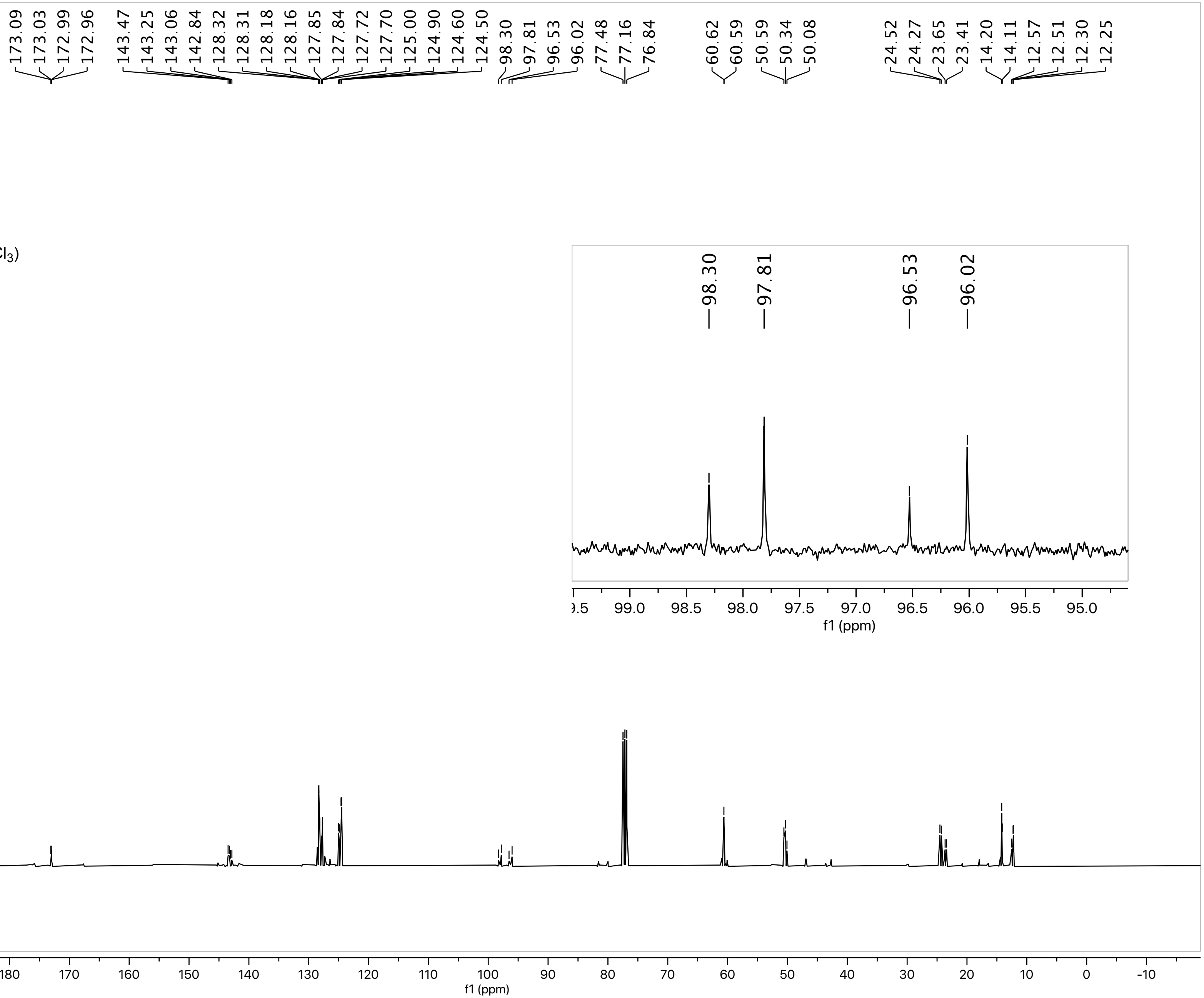

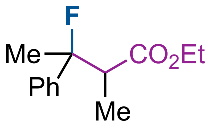

^{19}**F-NMR** (376MHz, $CDCl_3$)

6

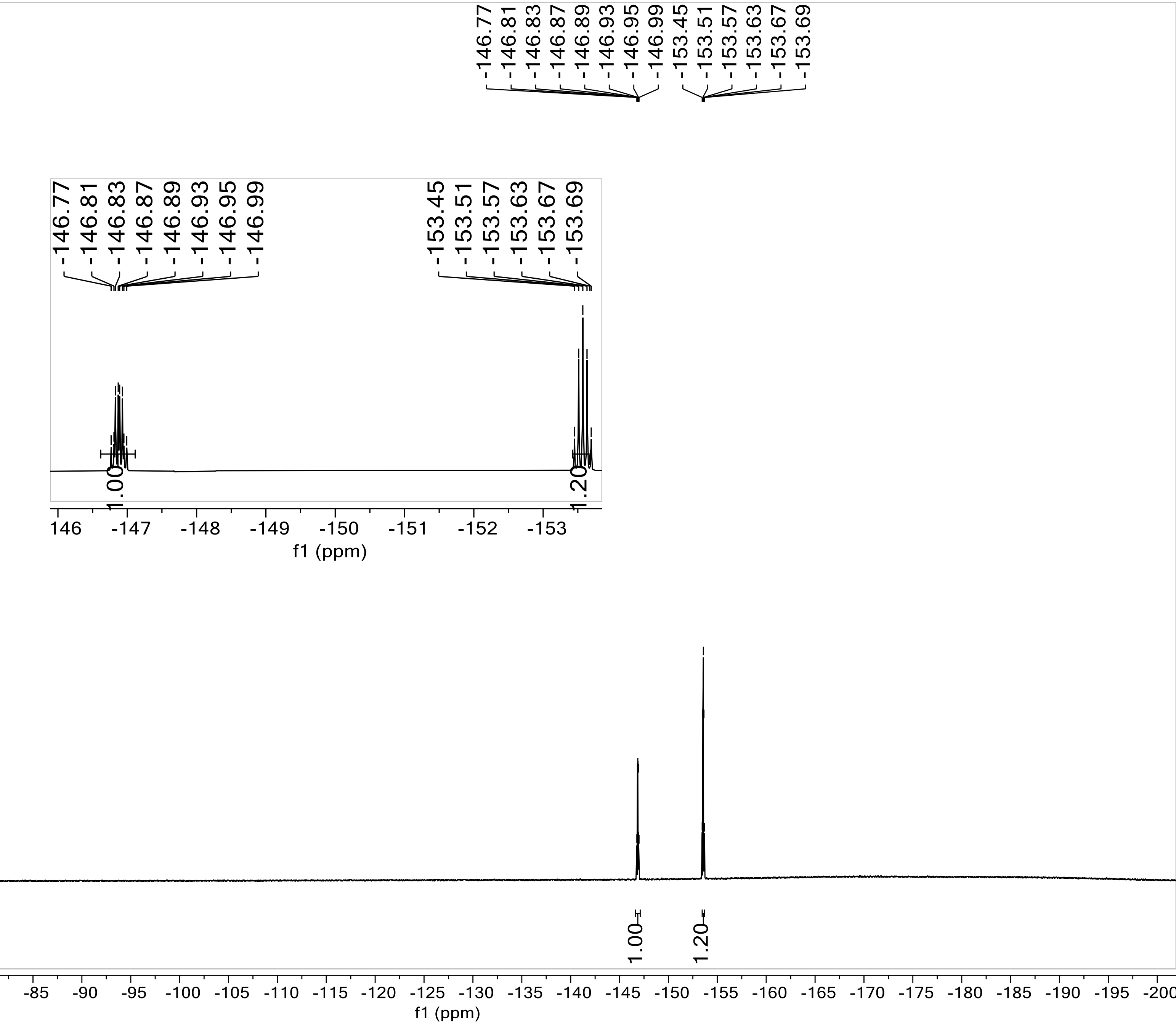

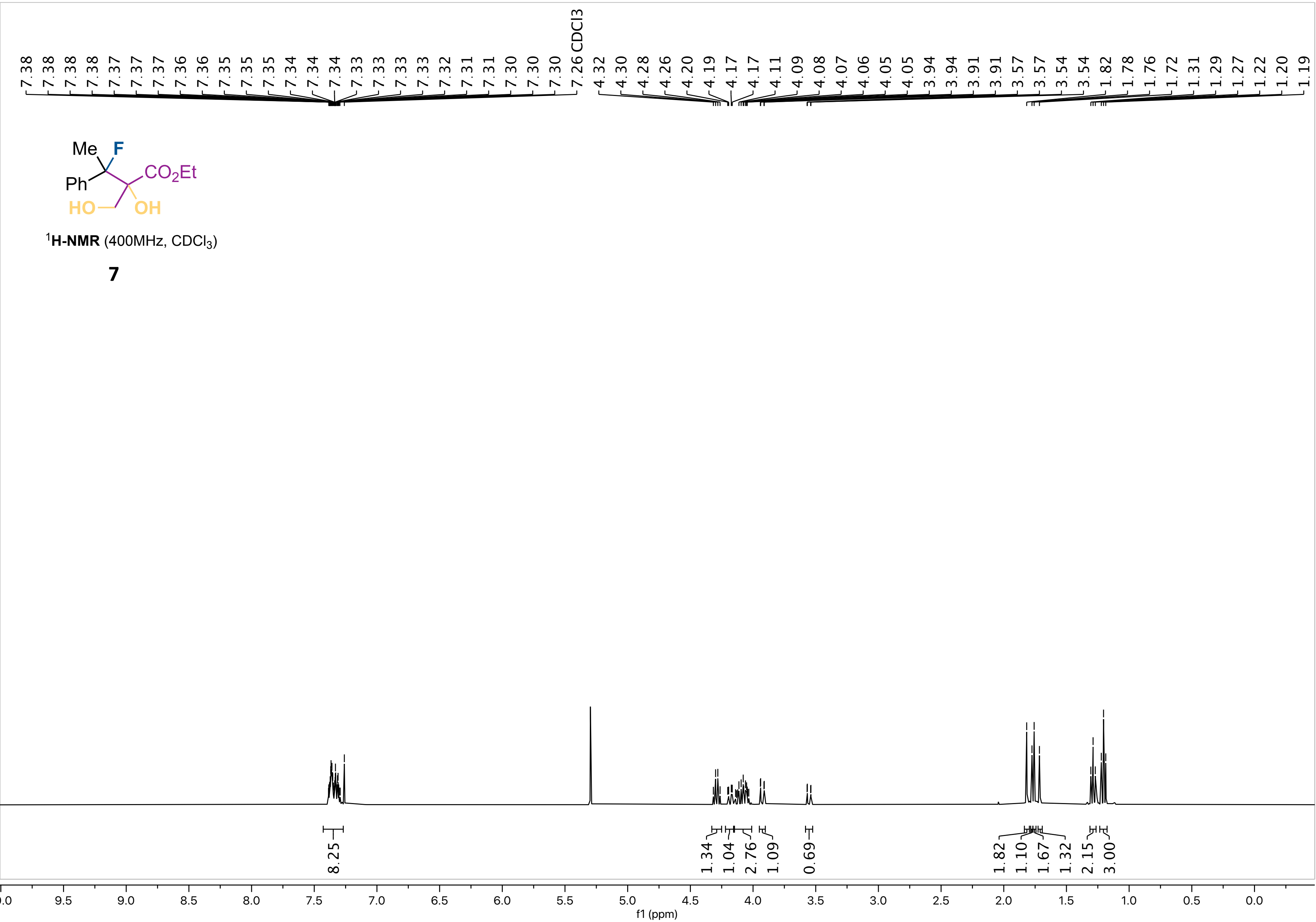
7.38 7.38 7.38 7.38 7.37 7.37 7.37 7.36 7.36 7.35 7.35 7.35 7.34 7.34 7.34 7.33 7.33 7.33 7.33 7.32 7.31 7.31 7.30 7.30 7.30 7.26 CDCl3
4.32 4.30 4.28 4.26 4.20 4.19 4.17 4.17 4.11 4.09 4.08 4.07 4.06 4.05 4.05 3.94 3.94 3.91 3.91 3.57 3.57 3.54 3.54 1.82 1.78 1.76 1.72 1.31 1.29 1.27 1.22 1.20 1.19
Me
F
Ph
CO2Et
HO
OH
1H-NMR (400MHz, CDCl3)
7
8.25
1.34 1.04 2.76 1.09
0.69
1.82 1.10 1.67 1.32 2.15 3.00
10.0 9.5 9.0 8.5 8.0 7.5 7.0 6.5 6.0 5.5 5.0 4.5 4.0 3.5 3.0 2.5 2.0 1.5 1.0 0.5 0.0
f1 (ppm)

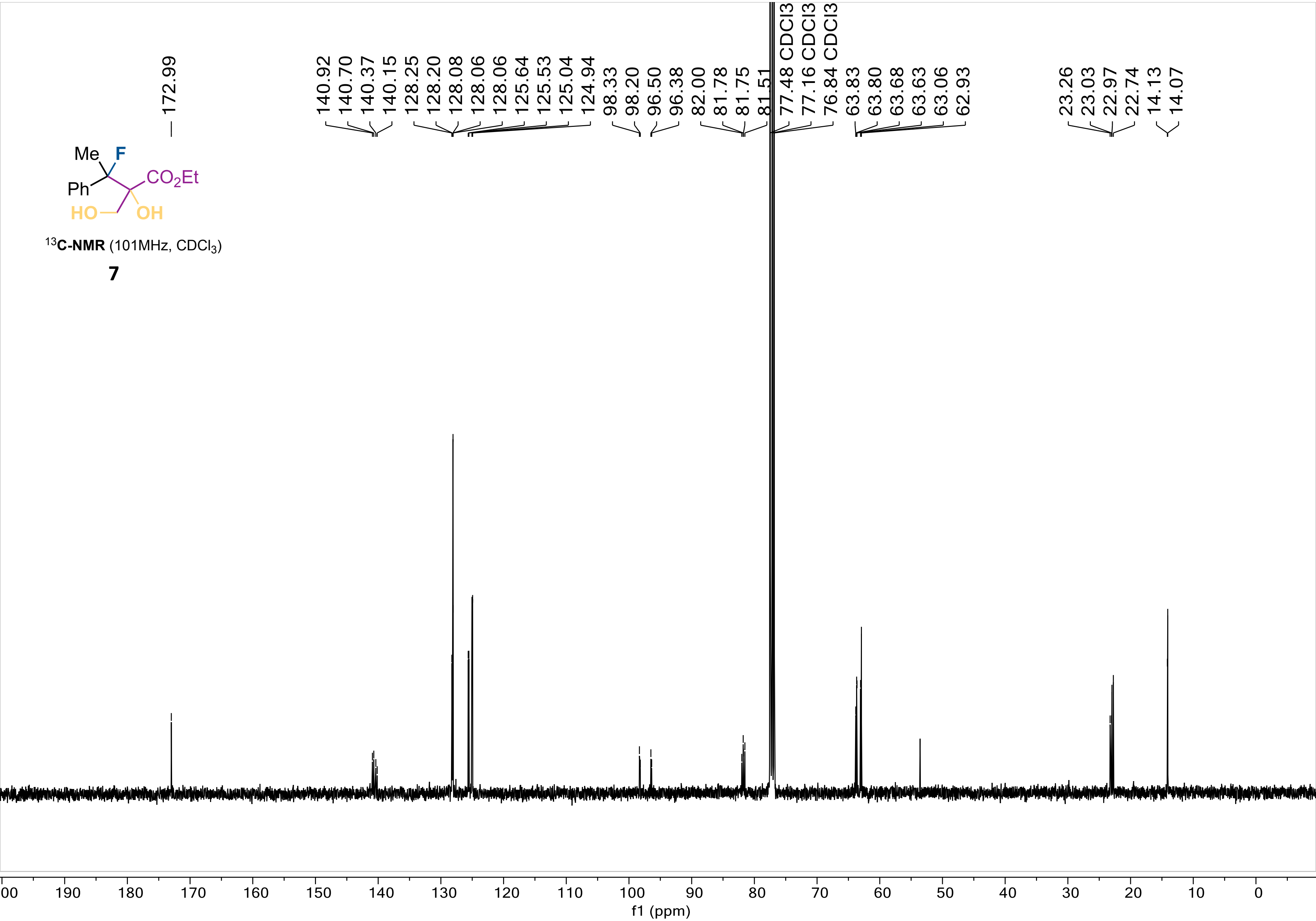

172.99
140.92
140.70
140.37
140.15
128.25
128.20
128.08
128.06
128.06
125.64
125.53
125.04
124.94
98.33
98.20
96.50
96.38
82.00
81.78
81.75
81.51
77.48 CDCl3
77.16 CDCl3
76.84 CDCl3
63.83
63.80
63.68
63.63
63.06
62.93
23.26
23.03
22.97
22.74
14.13
14.07
Me
F
Ph
CO2Et
HO
OH
13C-NMR (101MHz, CDCl3)
7
f1 (ppm)

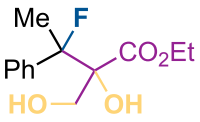

^{19}**F-NMR** (376MHz, $CDCl_3$)

7

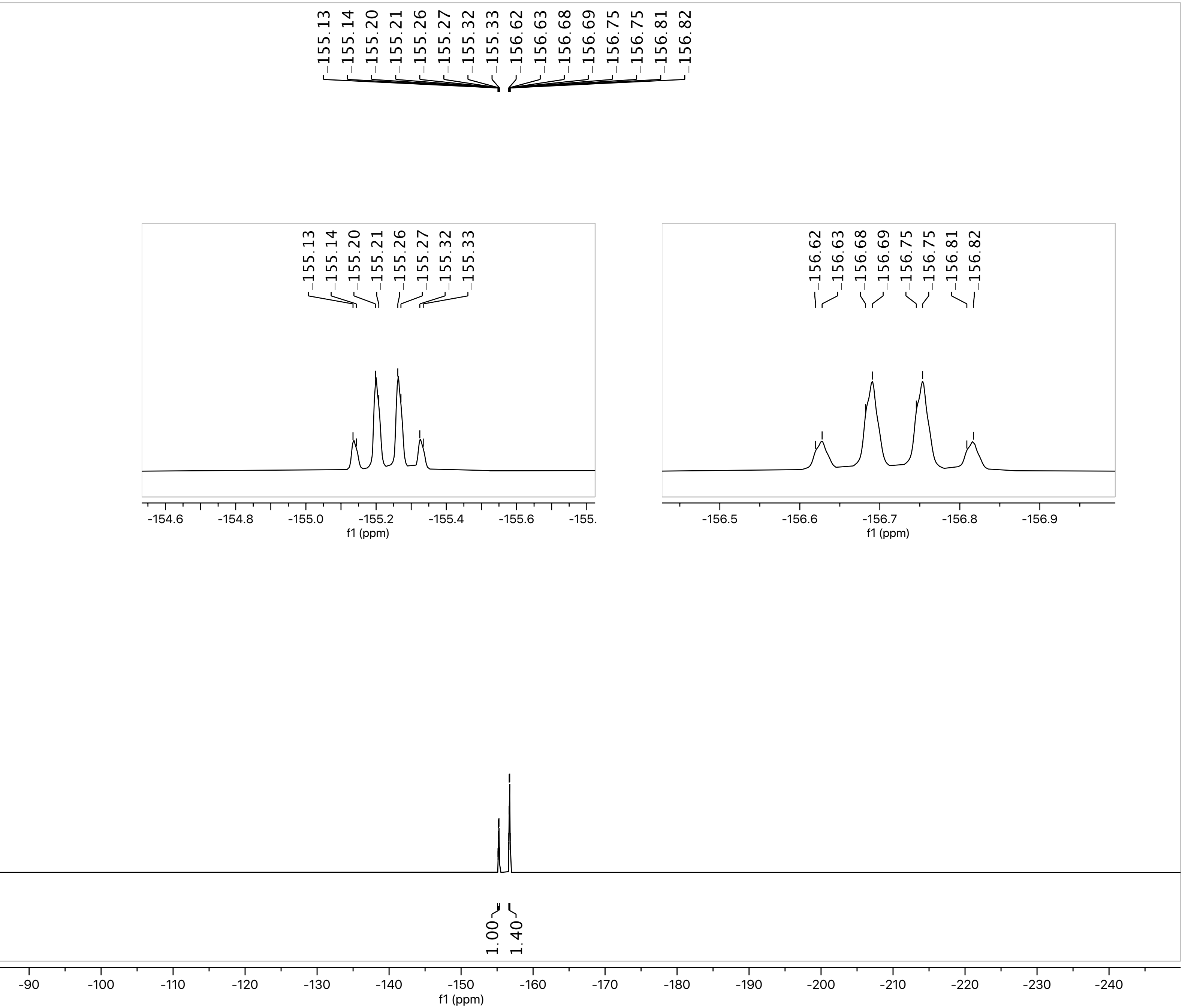

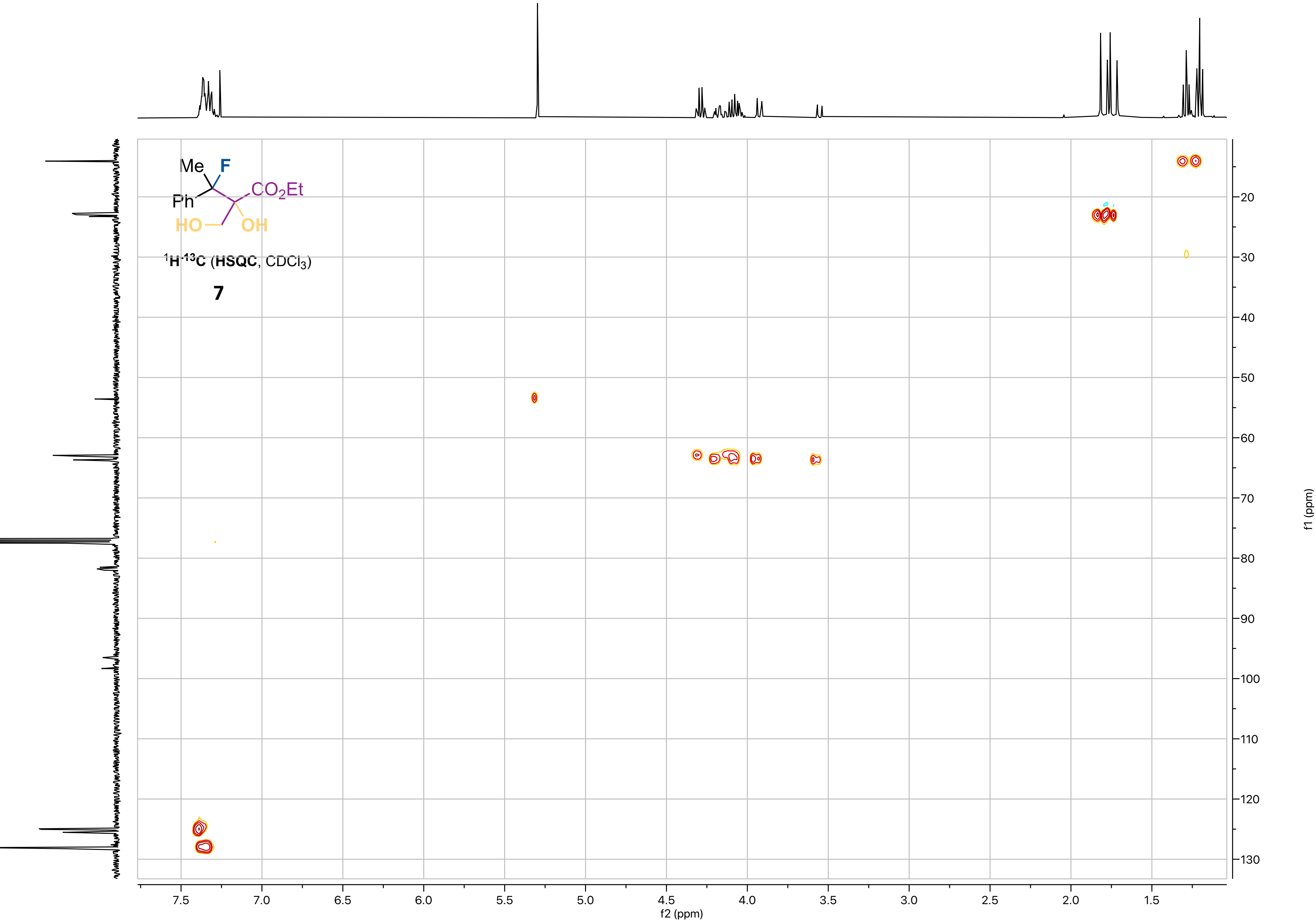

Me
F
Ph
CO2Et
HO
OH
1H-13C (HSQC, CDCl3)
7
f2 (ppm)
f1 (ppm)

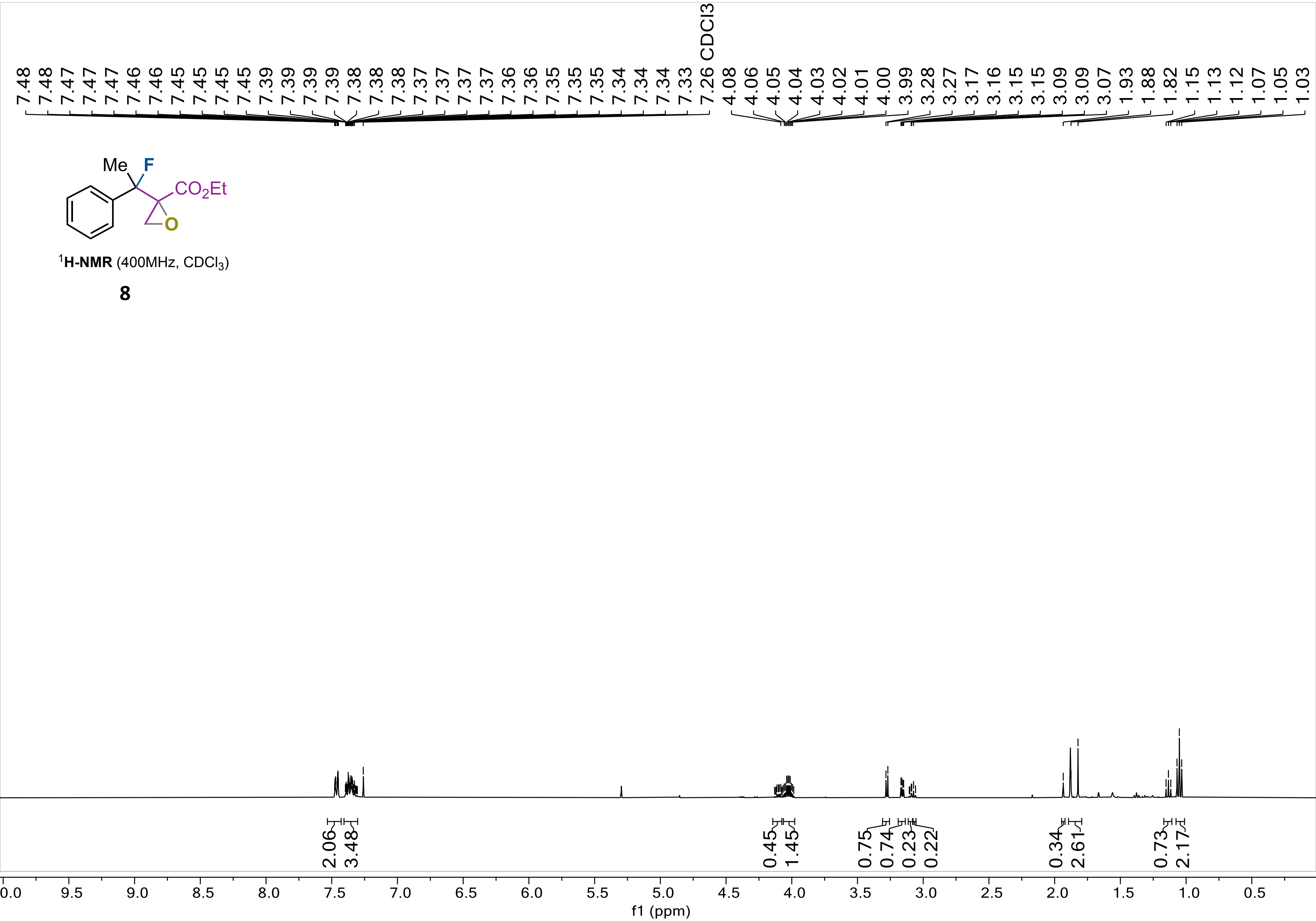
7.48
7.48
7.47
7.47
7.47
7.46
7.46
7.45
7.45
7.45
7.45
7.39
7.39
7.39
7.39
7.38
7.38
7.38
7.37
7.37
7.37
7.36
7.36
7.35
7.35
7.35
7.34
7.34
7.34
7.33
7.26 CDCl3
4.08
4.06
4.05
4.04
4.03
4.02
4.01
4.00
3.99
3.28
3.27
3.17
3.16
3.15
3.15
3.09
3.09
3.07
1.93
1.88
1.82
1.15
1.13
1.12
1.07
1.05
1.03
Me
F
CO2Et
O
1H-NMR (400MHz, CDCl3)
8
2.06
3.48
0.45
1.45
0.75
0.74
0.23
0.22
0.34
2.61
0.73
2.17
f1 (ppm)

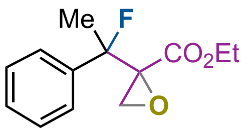

^{13}**C-NMR** (101MHz, $CDCl_3$)

8

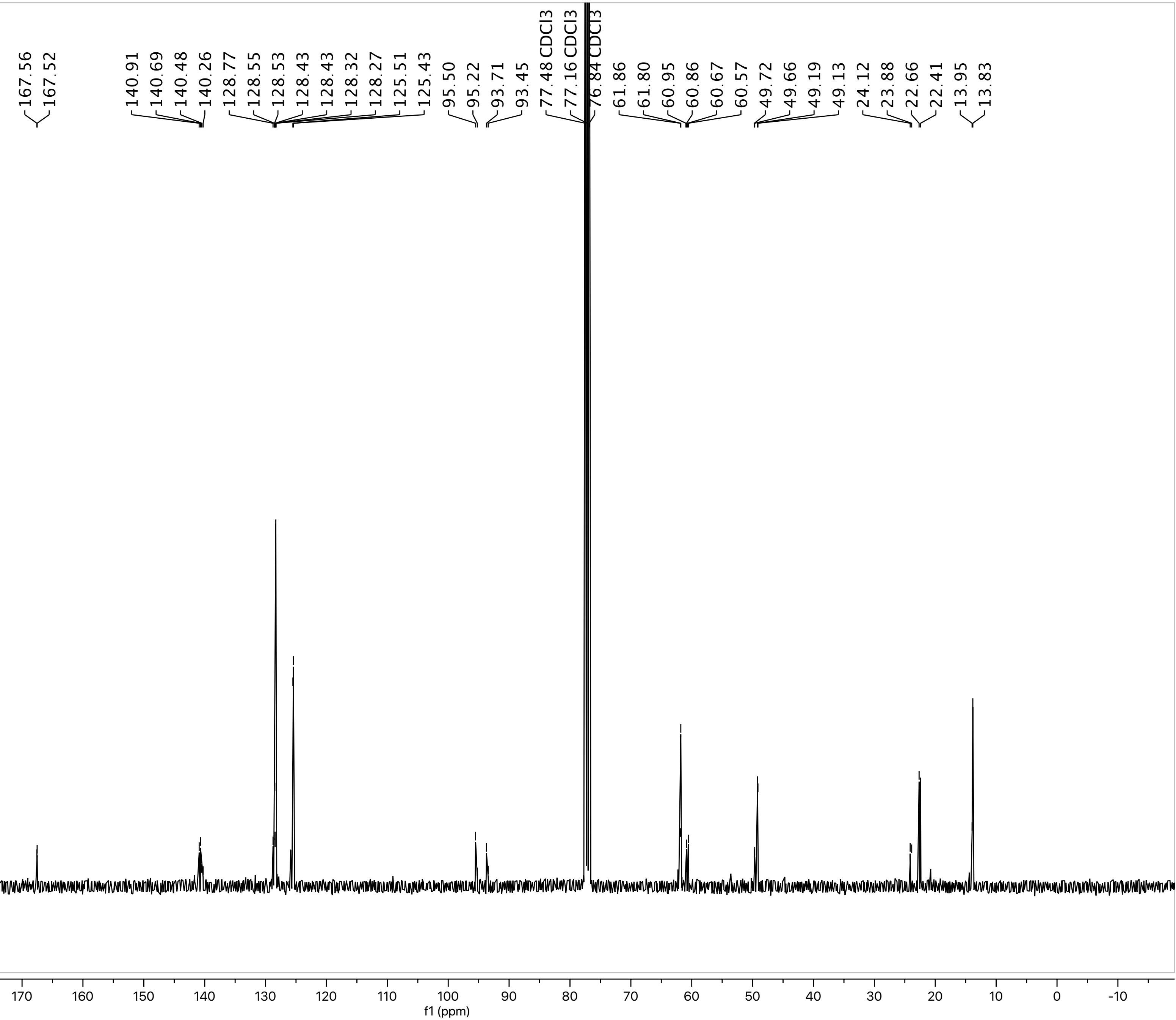

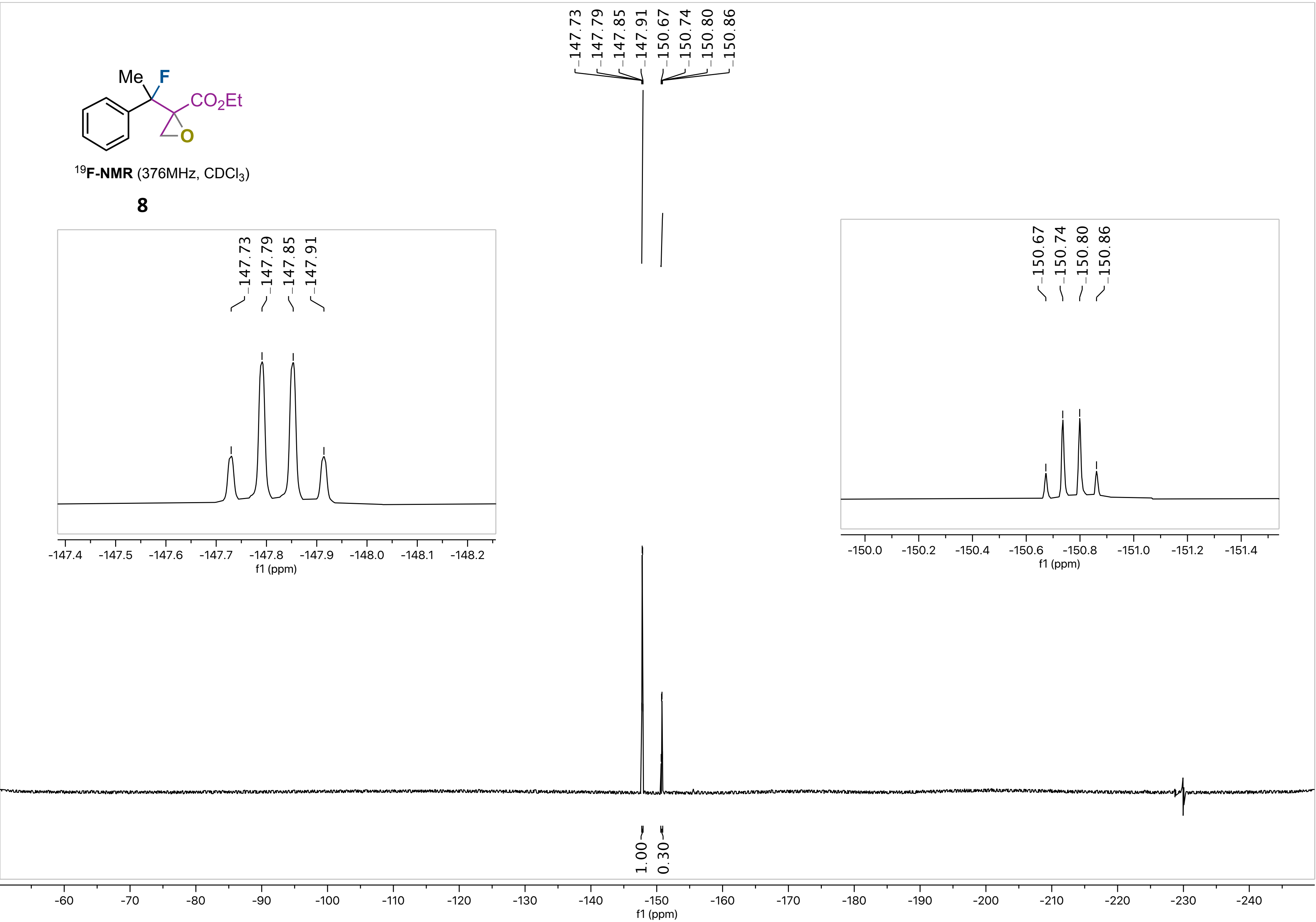

Me
F
CO2Et
O
19F-NMR (376MHz, CDCl3)
8
-147.73
-147.79
-147.85
-147.91
-150.67
-150.74
-150.80
-150.86
1.00
0.30
f1 (ppm)

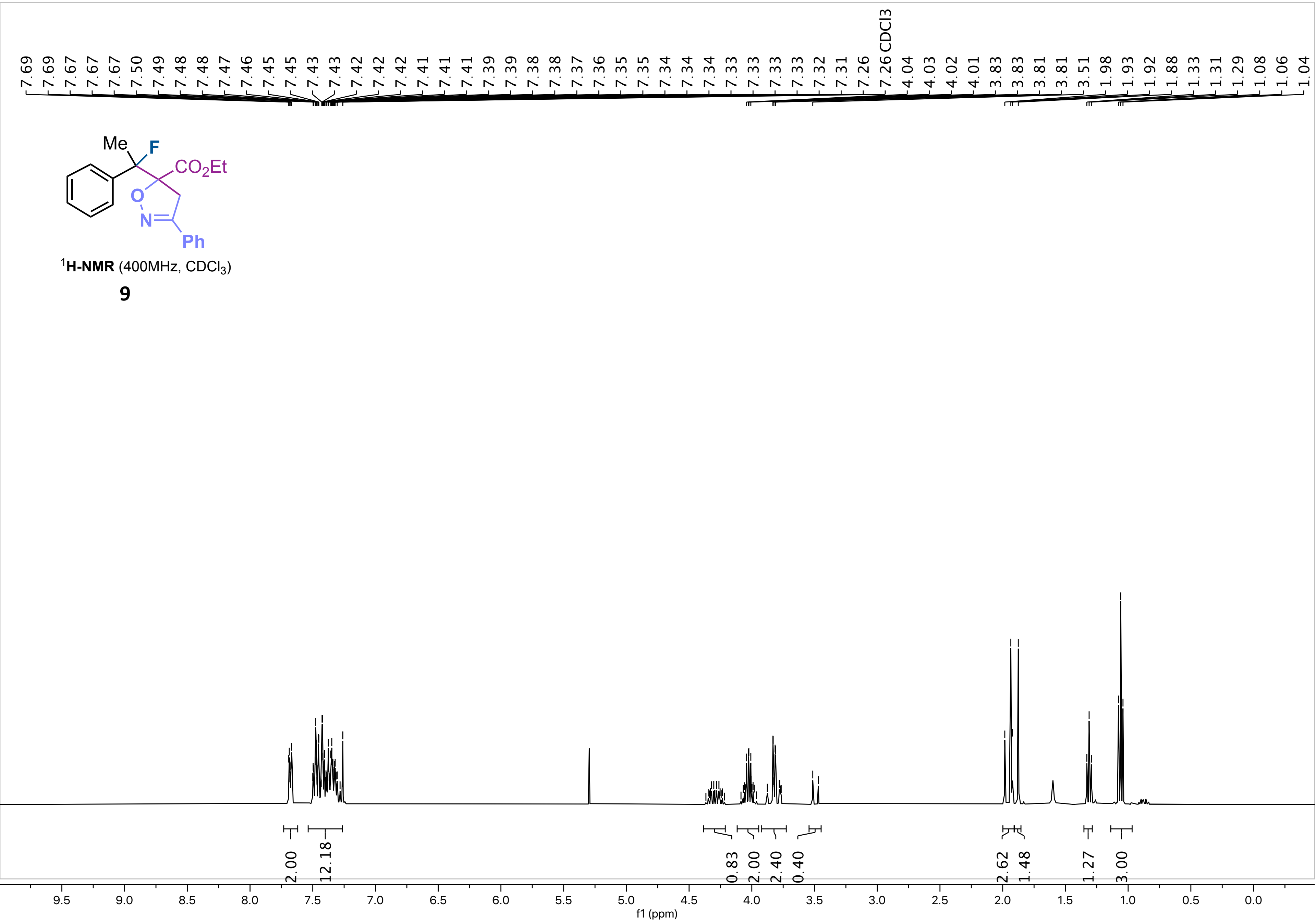

[1]**H-NMR** (400MHz, $CDCl_3$)

9

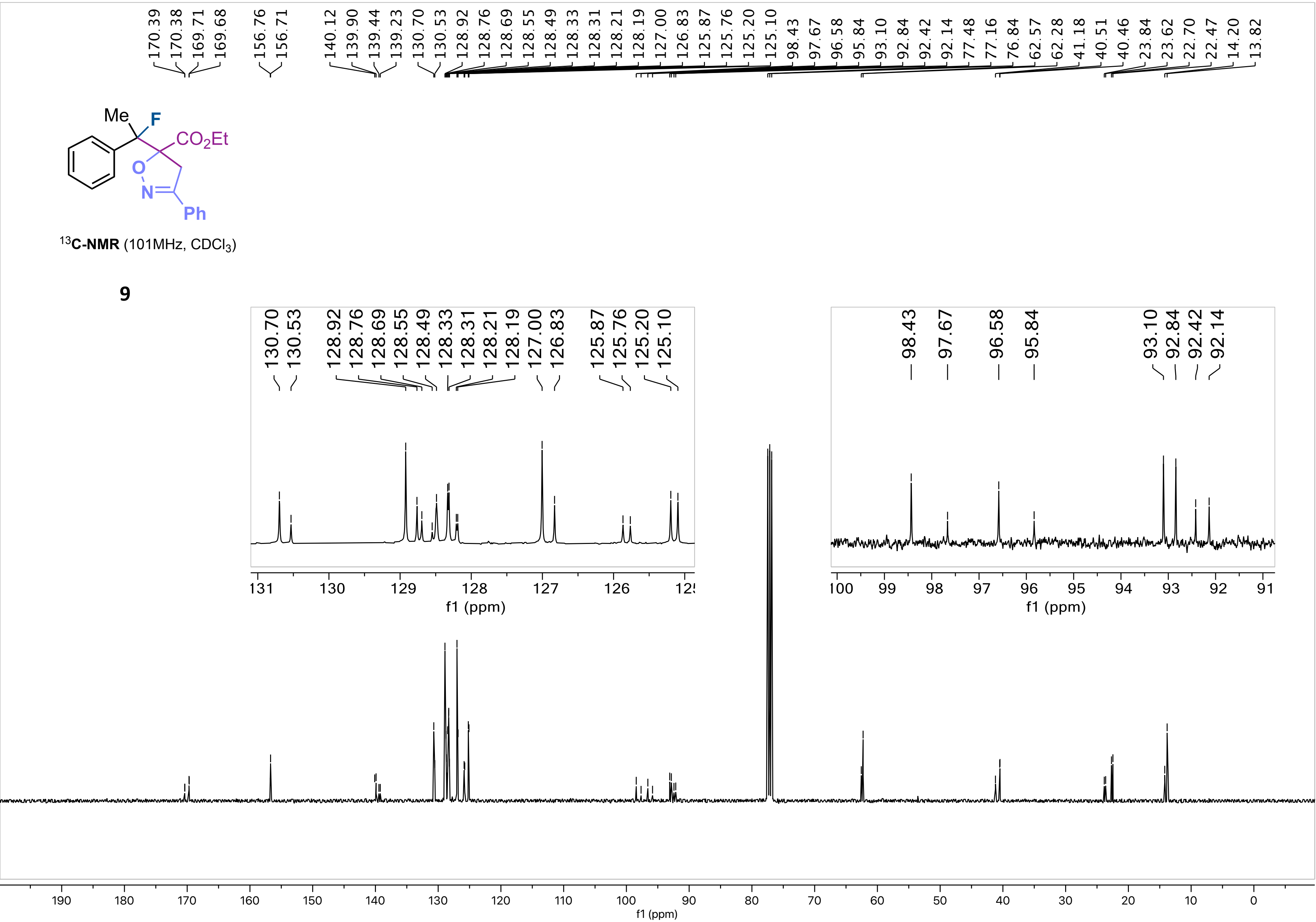

^{13}**C-NMR** (101MHz, $CDCl_3$)

9

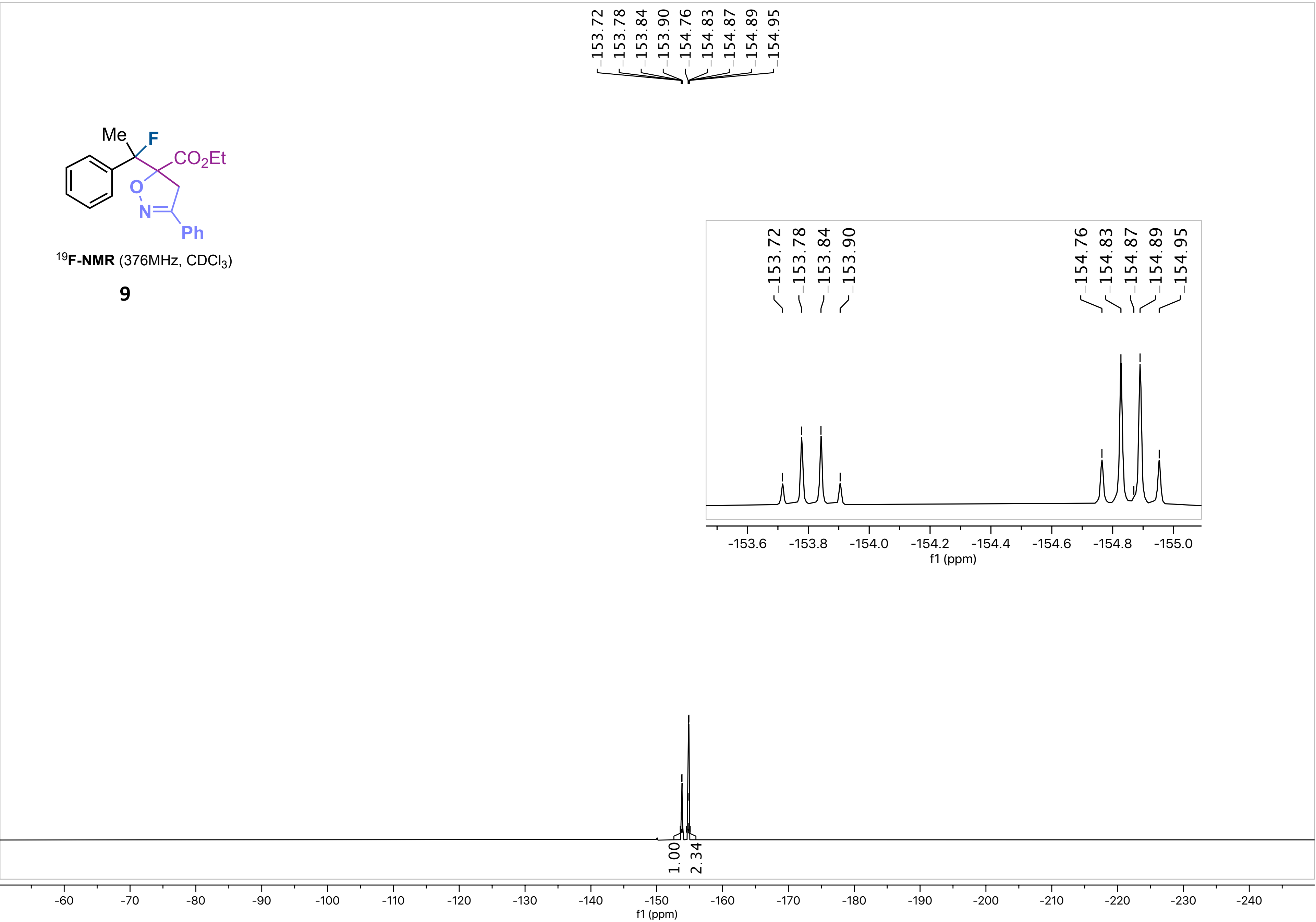

^{19}**F-NMR** (376MHz, $CDCl_3$)

9

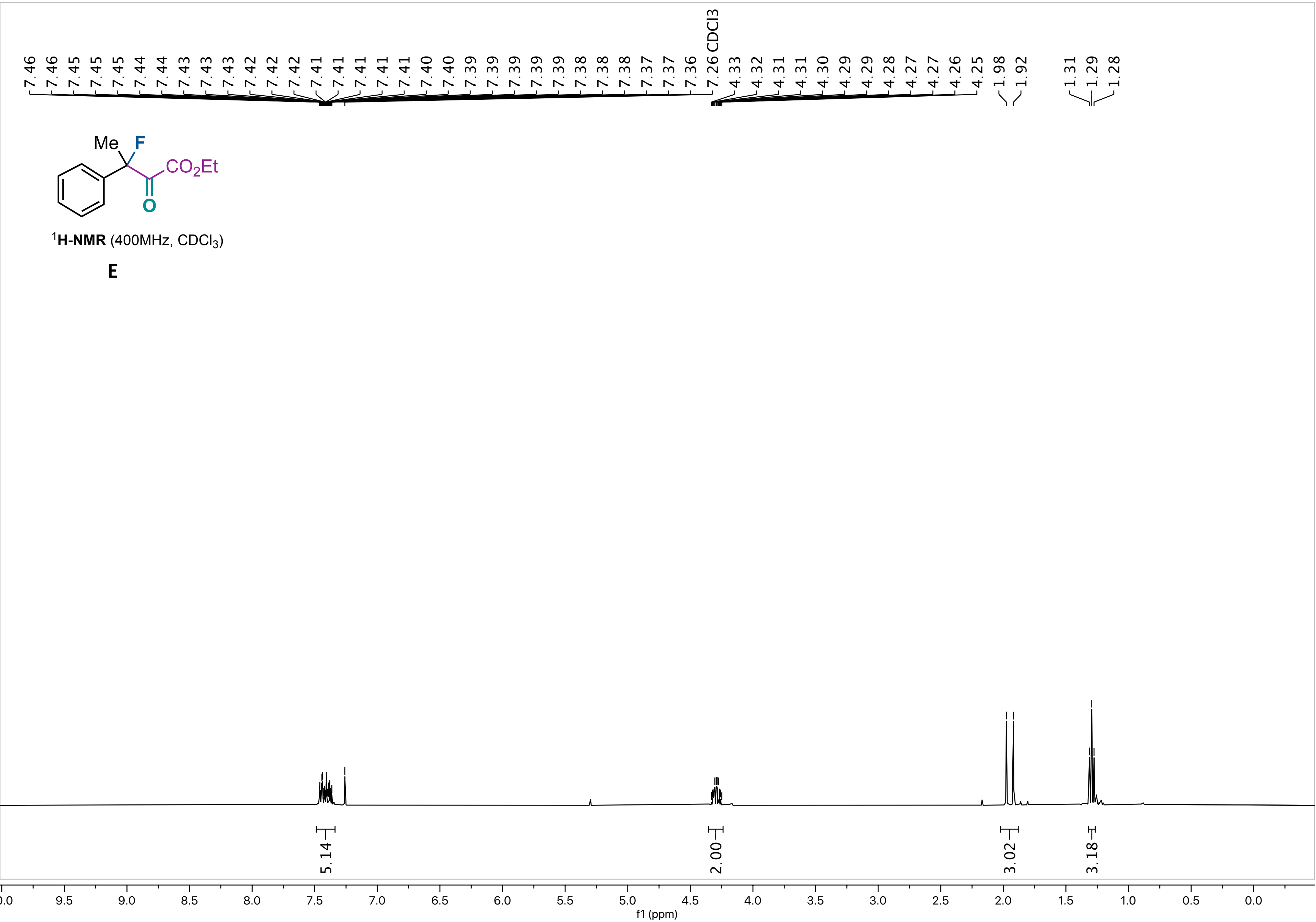
7.46 7.46 7.45 7.45 7.45 7.44 7.44 7.43 7.43 7.43 7.42 7.42 7.42 7.41 7.41 7.41 7.41 7.41 7.40 7.40 7.39 7.39 7.39 7.39 7.39 7.38 7.38 7.38 7.37 7.37 7.36 7.26 CDCl3 4.33 4.32 4.31 4.31 4.30 4.29 4.29 4.28 4.27 4.27 4.26 4.25 1.98 1.92 1.31 1.29 1.28
Me
F
CO2Et
O
1H-NMR (400MHz, CDCl3)
E
5.14
2.00
3.02
3.18
f1 (ppm)

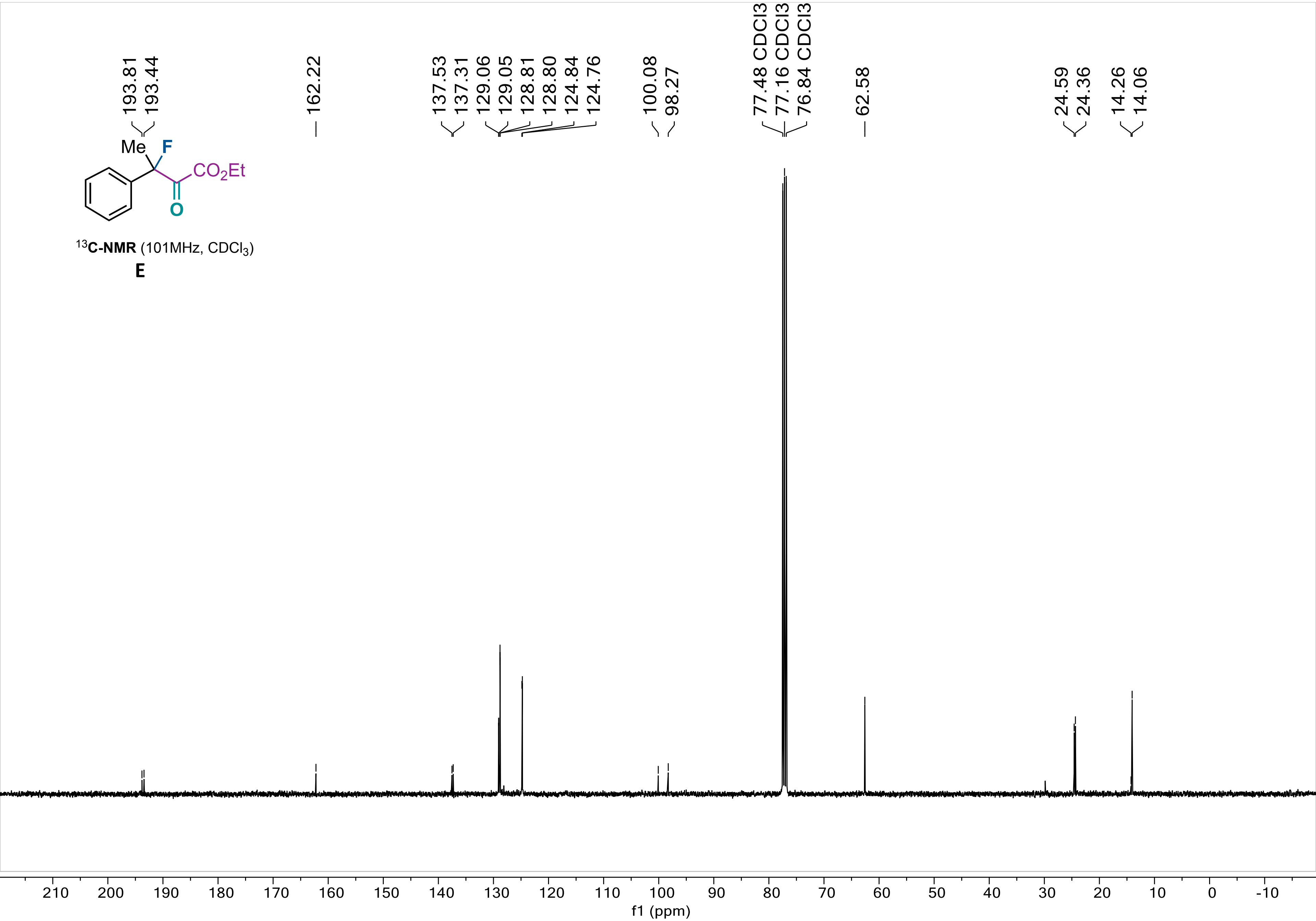
193.81
193.44
162.22
137.53
137.31
129.06
129.05
128.81
128.80
124.84
124.76
100.08
98.27
77.48 CDCl3
77.16 CDCl3
76.84 CDCl3
62.58
24.59
24.36
14.26
14.06
Me
F
CO_2Et
O
^{13}C-NMR (101MHz, $CDCl_3$)
E
210 200 190 180 170 160 150 140 130 120 110 100 90 80 70 60 50 40 30 20 10 0 -10
f1 (ppm)

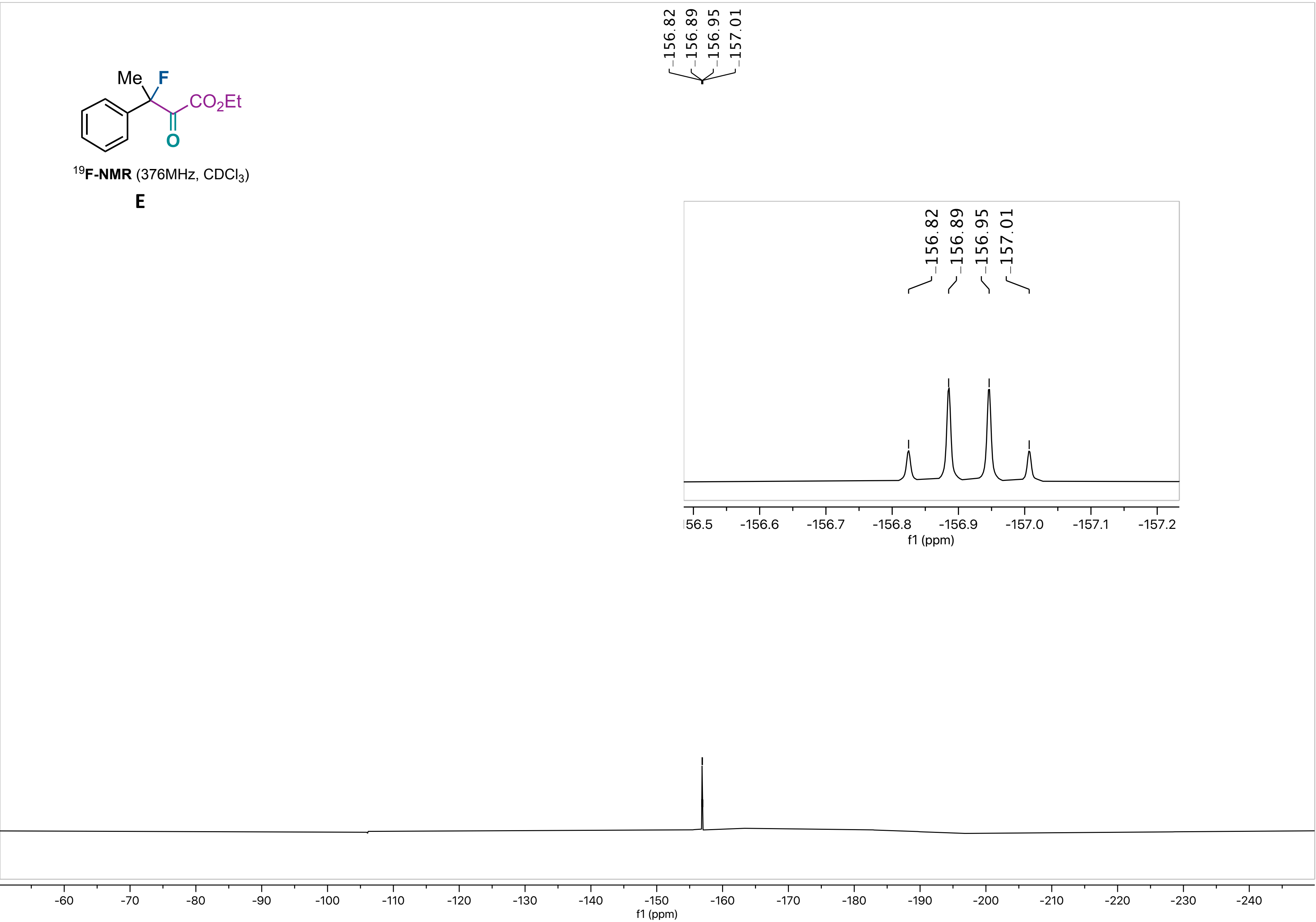

^{19}**F-NMR** (376MHz, $CDCl_3$)

E

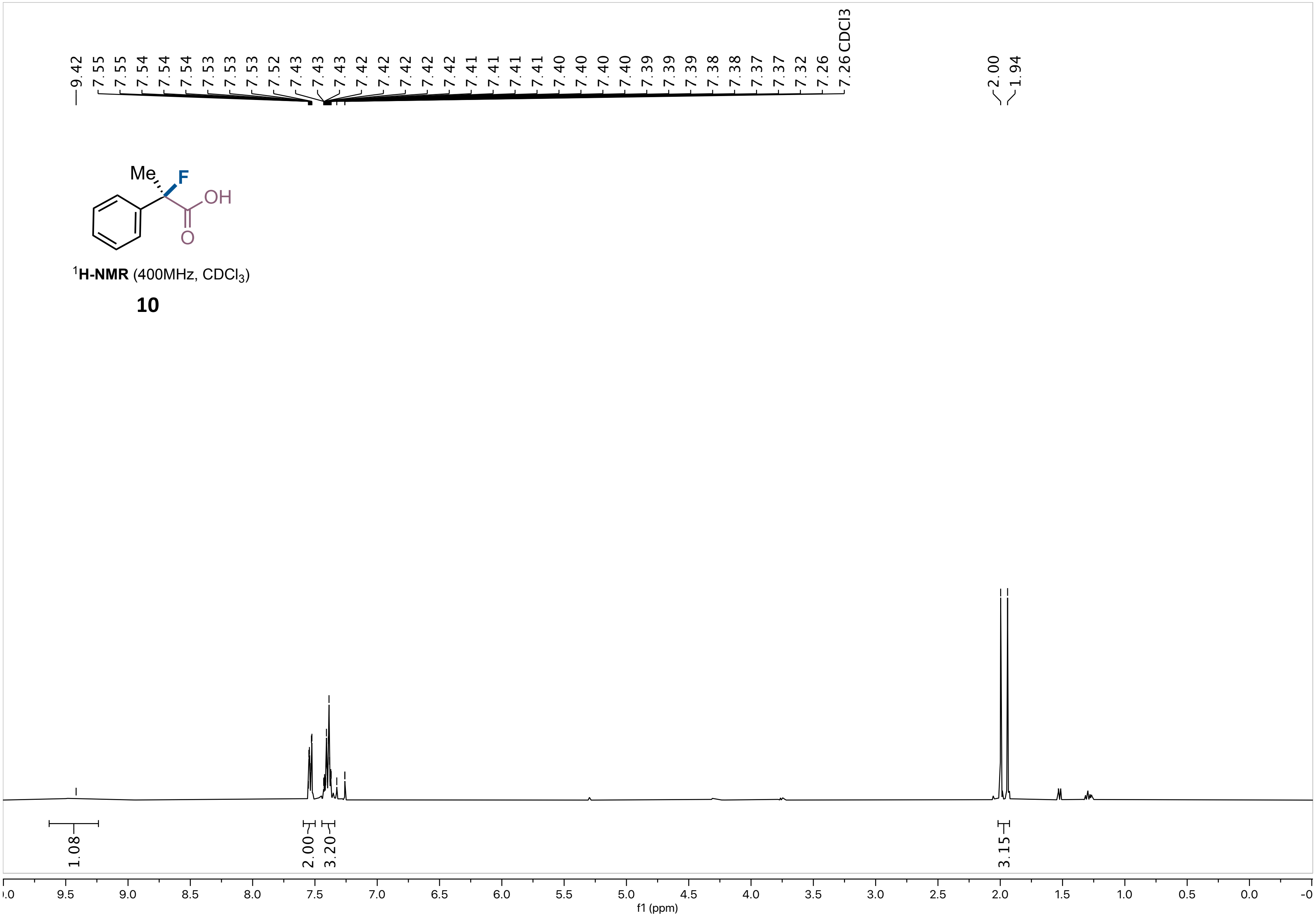

^{1}**H-NMR** (400MHz, $CDCl_3$)

10

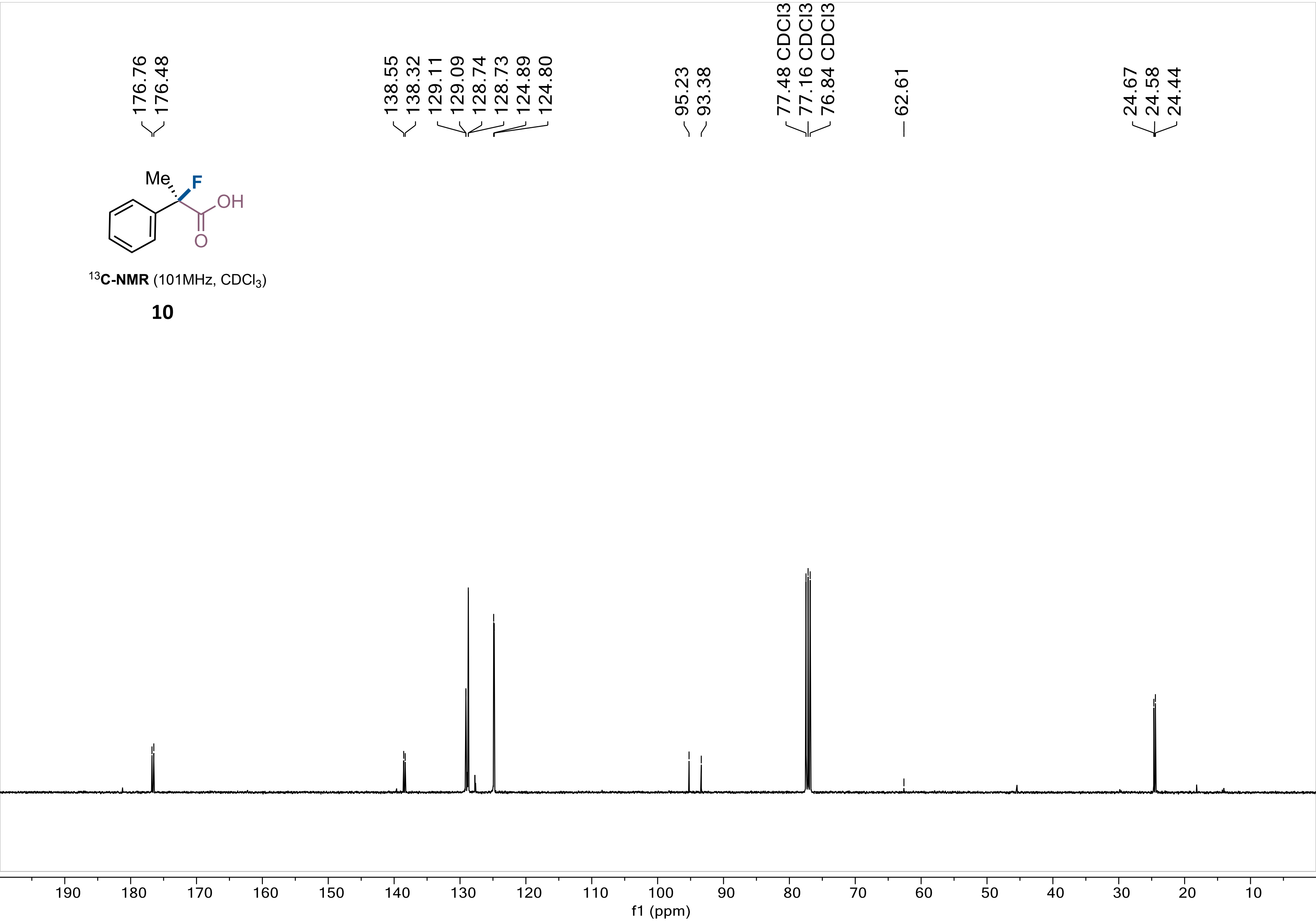

^{13}**C-NMR** (101MHz, $CDCl_3$)

10

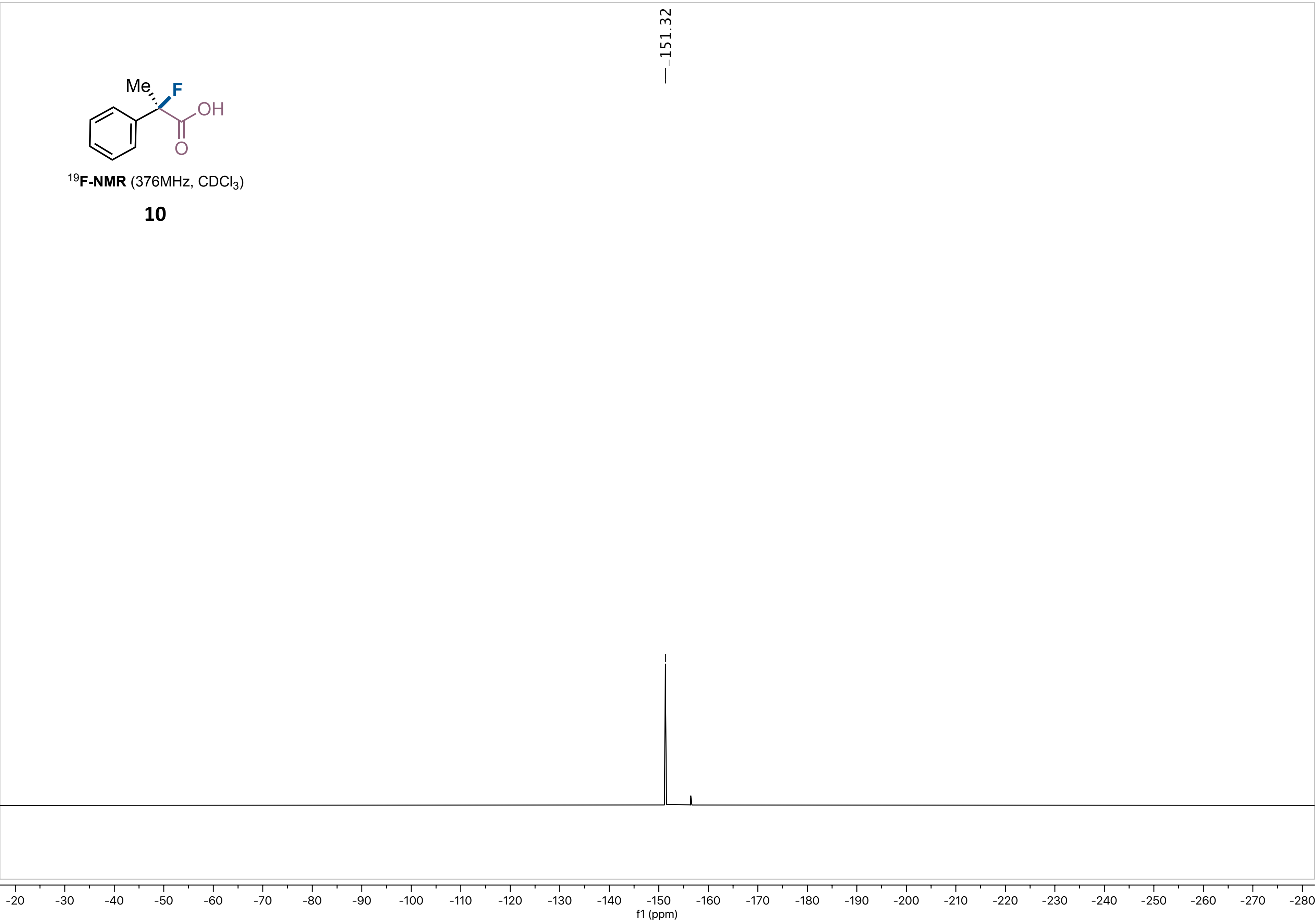
-151.32
Me
F
OH
O
19F-NMR (376MHz, CDCl3)
10
-20 -30 -40 -50 -60 -70 -80 -90 -100 -110 -120 -130 -140 -150 -160 -170 -180 -190 -200 -210 -220 -230 -240 -250 -260 -270 -280
f1 (ppm)

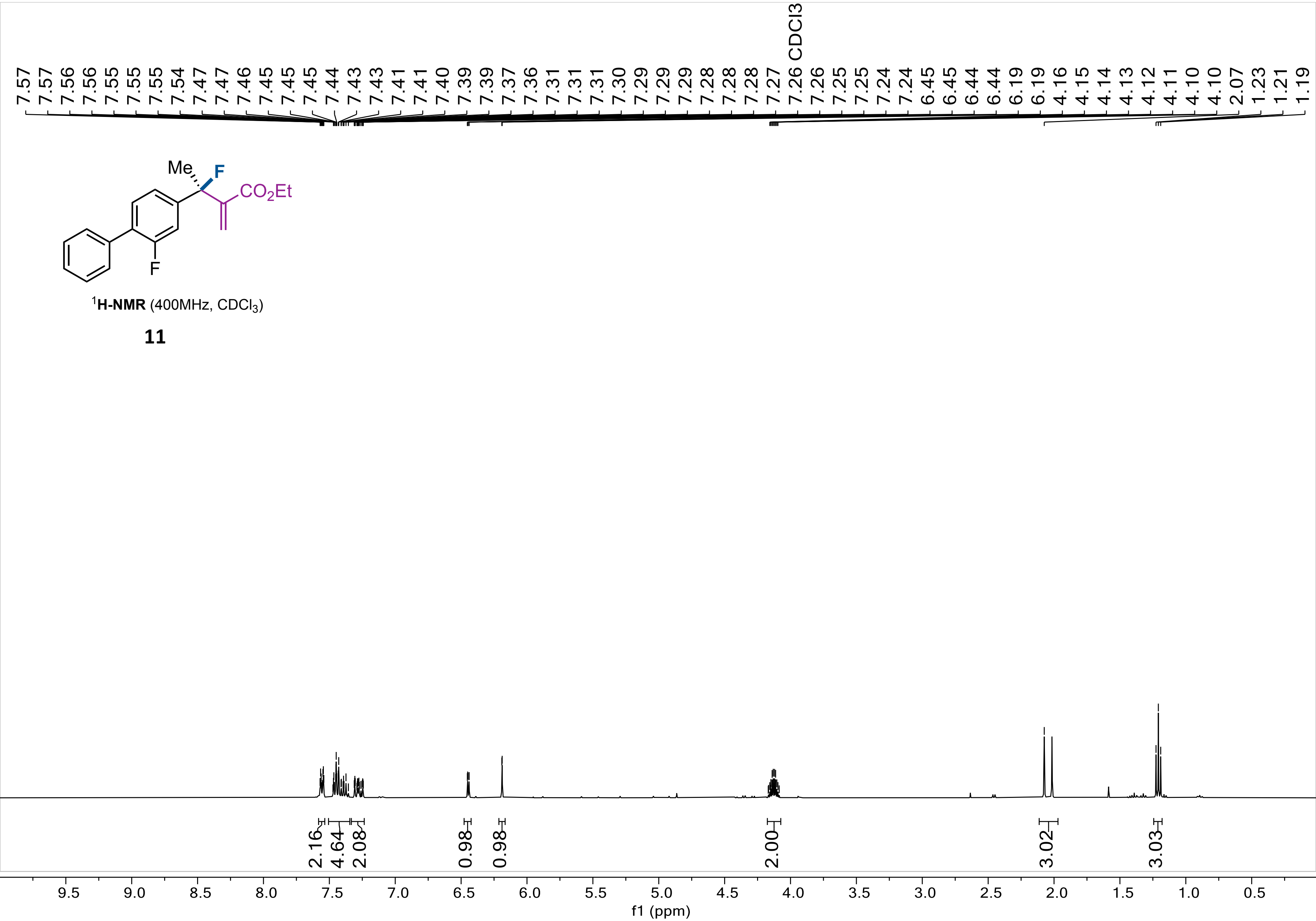

[1]**H-NMR** (400MHz, $CDCl_3$)

11

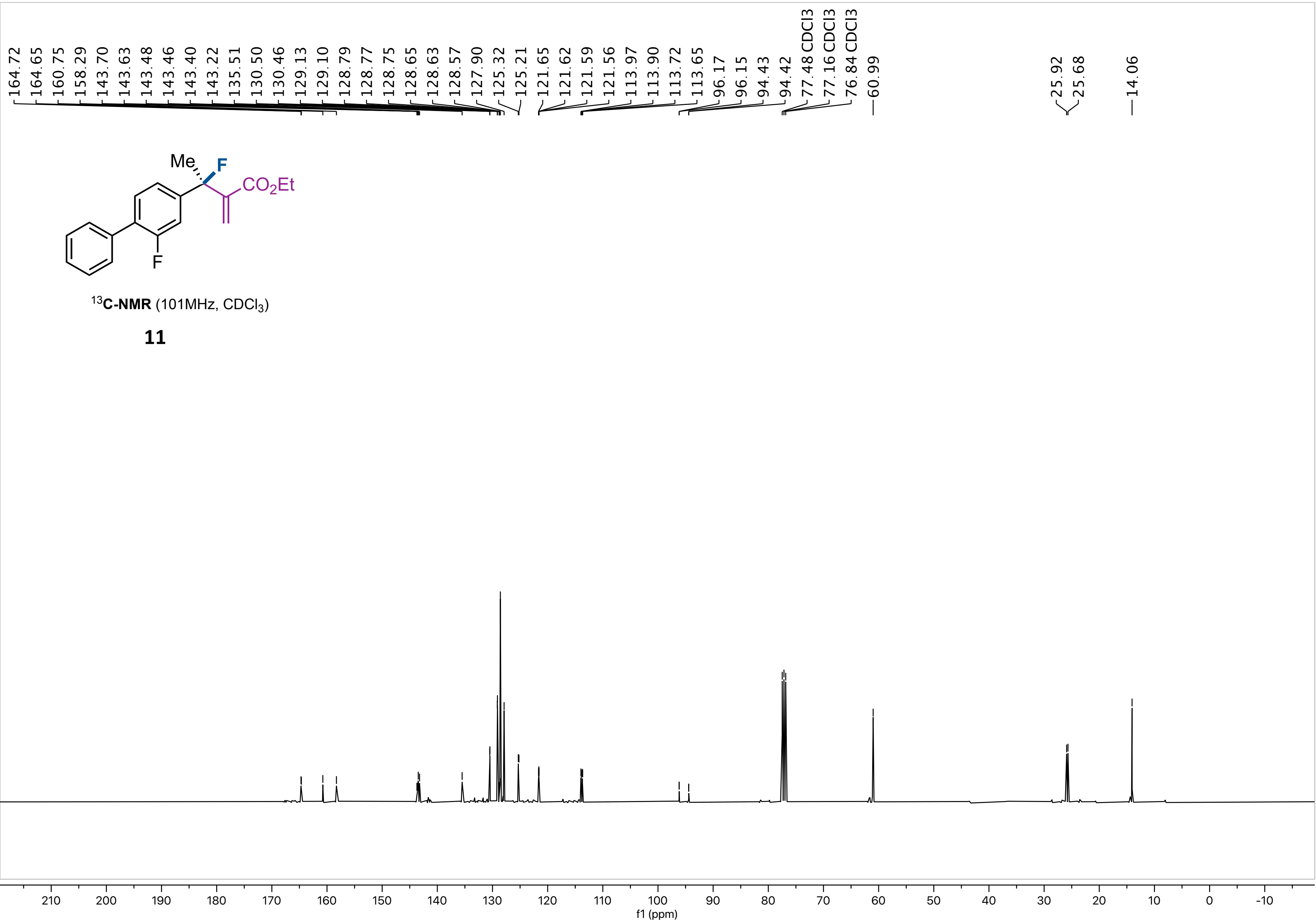

^{13}**C-NMR** (101MHz, $CDCl_3$)

11

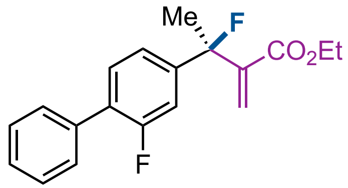

^{19}**F-NMR** (376MHz, $CDCl_3$)

11

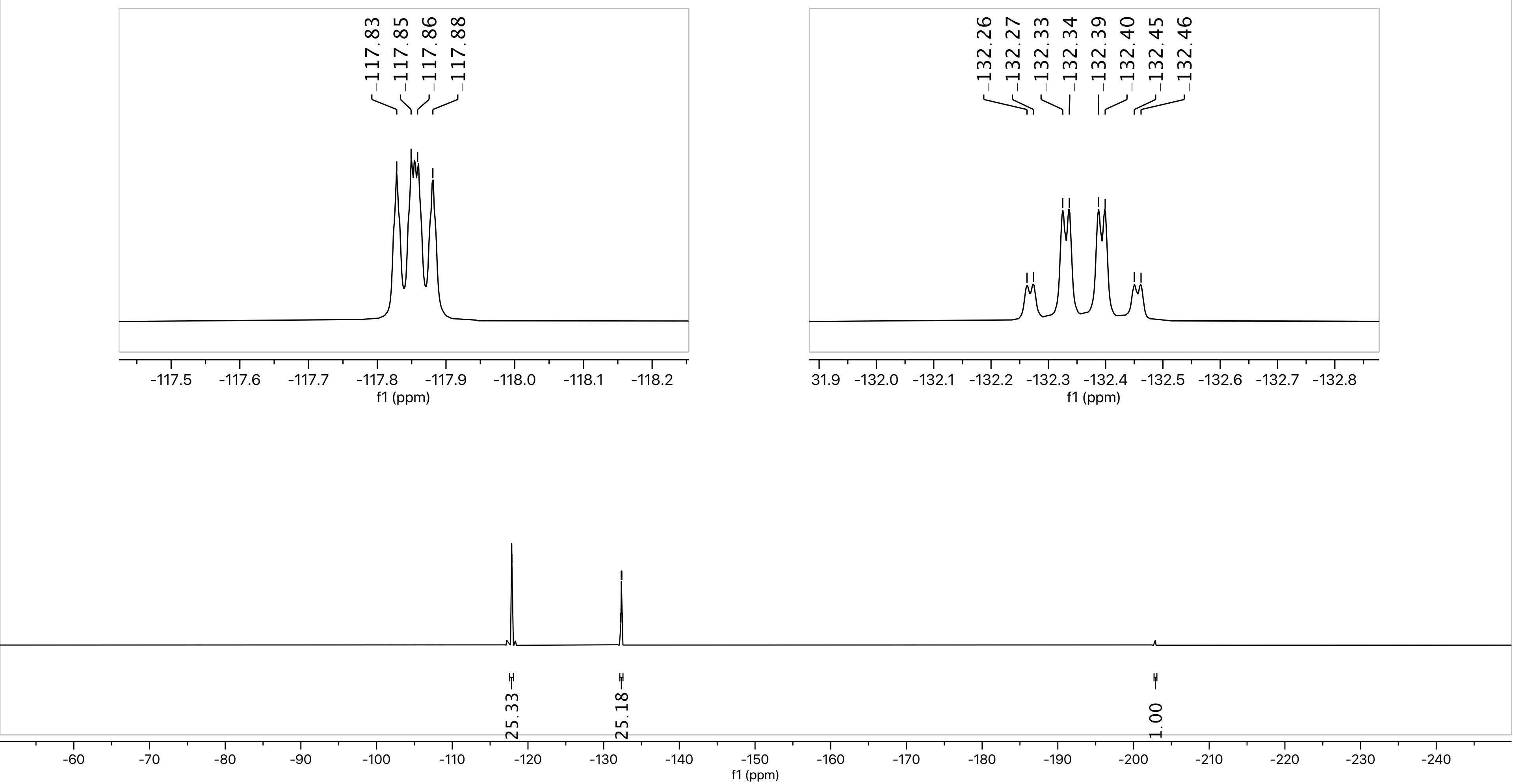

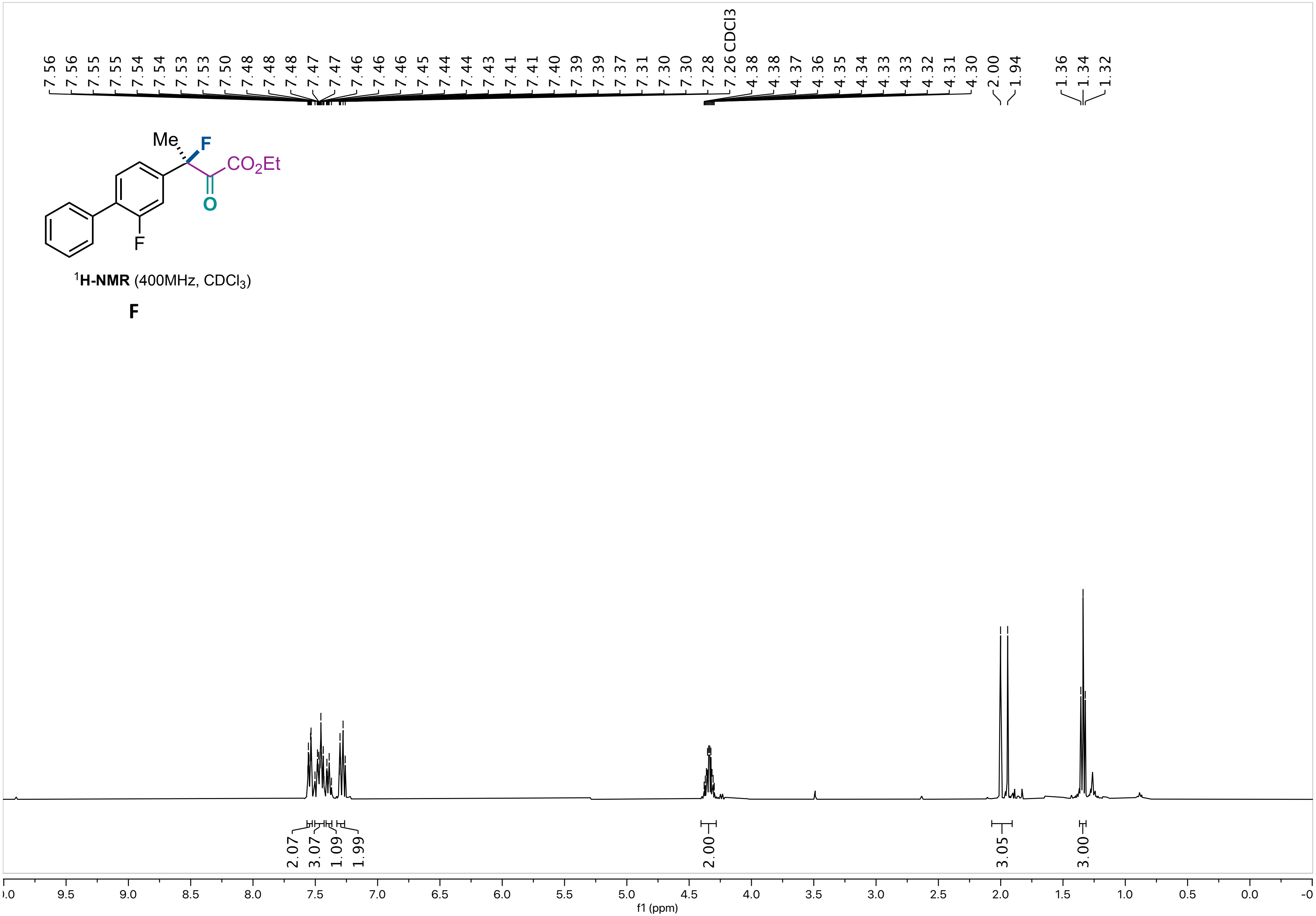
7.56 7.56 7.55 7.55 7.54 7.54 7.53 7.53 7.50 7.48 7.48 7.48 7.47 7.47 7.46 7.46 7.46 7.45 7.44 7.44 7.43 7.41 7.41 7.40 7.39 7.39 7.37 7.31 7.30 7.30 7.28 7.26 CDCl3 4.38 4.38 4.37 4.36 4.35 4.34 4.33 4.33 4.32 4.31 4.30 2.00 1.94 1.36 1.34 1.32
Me
F
CO2Et
O
F
1H-NMR (400MHz, CDCl3)
F
2.07
3.07
1.09
1.99
2.00
3.05
3.00
9.5
9.0
8.5
8.0
7.5
7.0
6.5
6.0
5.5
5.0
4.5
4.0
3.5
3.0
2.5
2.0
1.5
1.0
0.5
0.0
f1 (ppm)

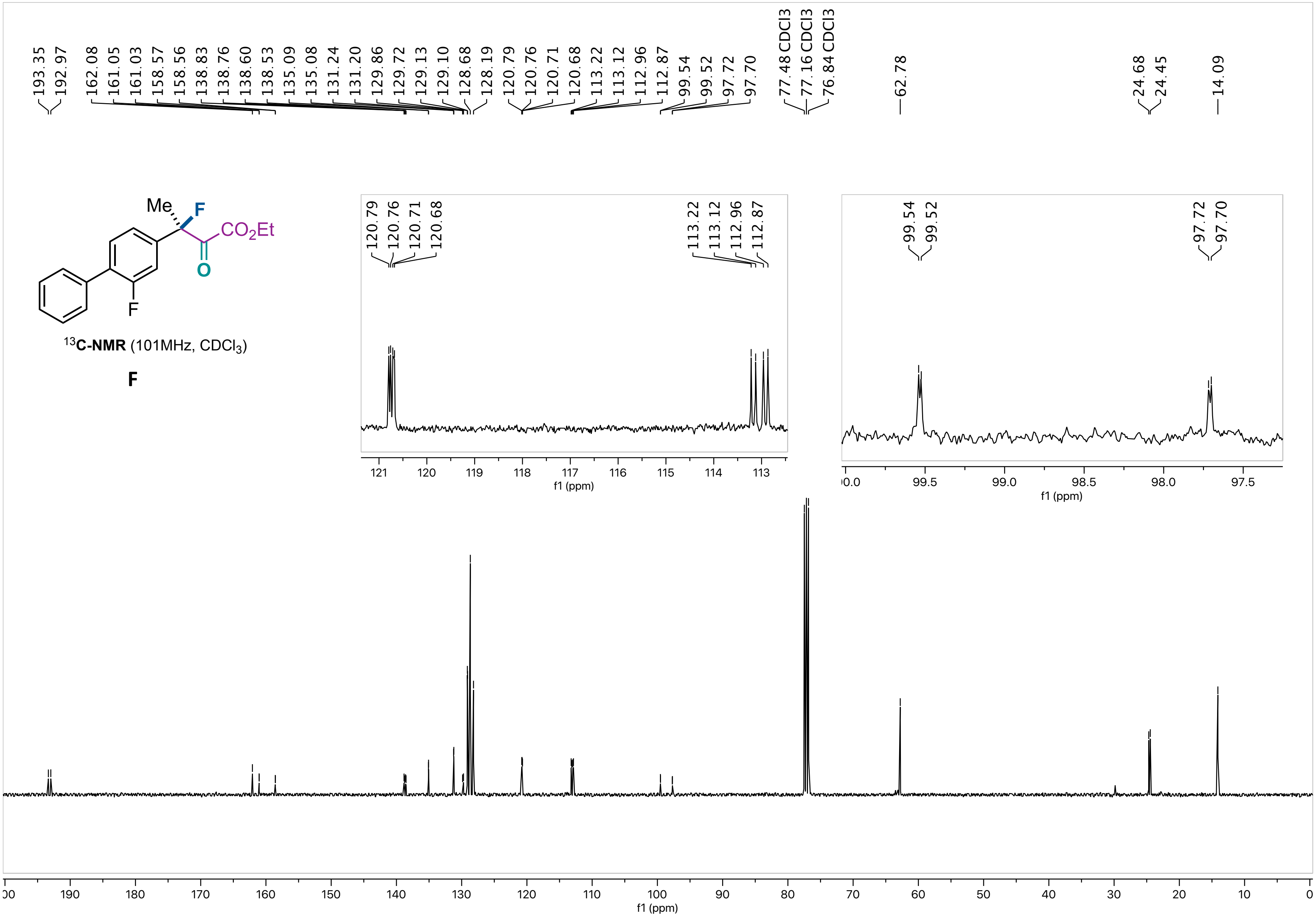
193.35
192.97
162.08
161.05
161.03
158.57
158.56
138.83
138.76
138.60
138.53
135.09
135.08
131.24
131.20
129.86
129.72
129.13
129.10
128.68
128.19
120.79
120.76
120.71
120.68
113.22
113.12
112.96
112.87
99.54
99.52
97.72
97.70
77.48 CDCl3
77.16 CDCl3
76.84 CDCl3
62.78
24.68
24.45
14.09
Me
F
CO2Et
O
F
13C-NMR (101MHz, CDCl3)
F
f1 (ppm)
f1 (ppm)
f1 (ppm)

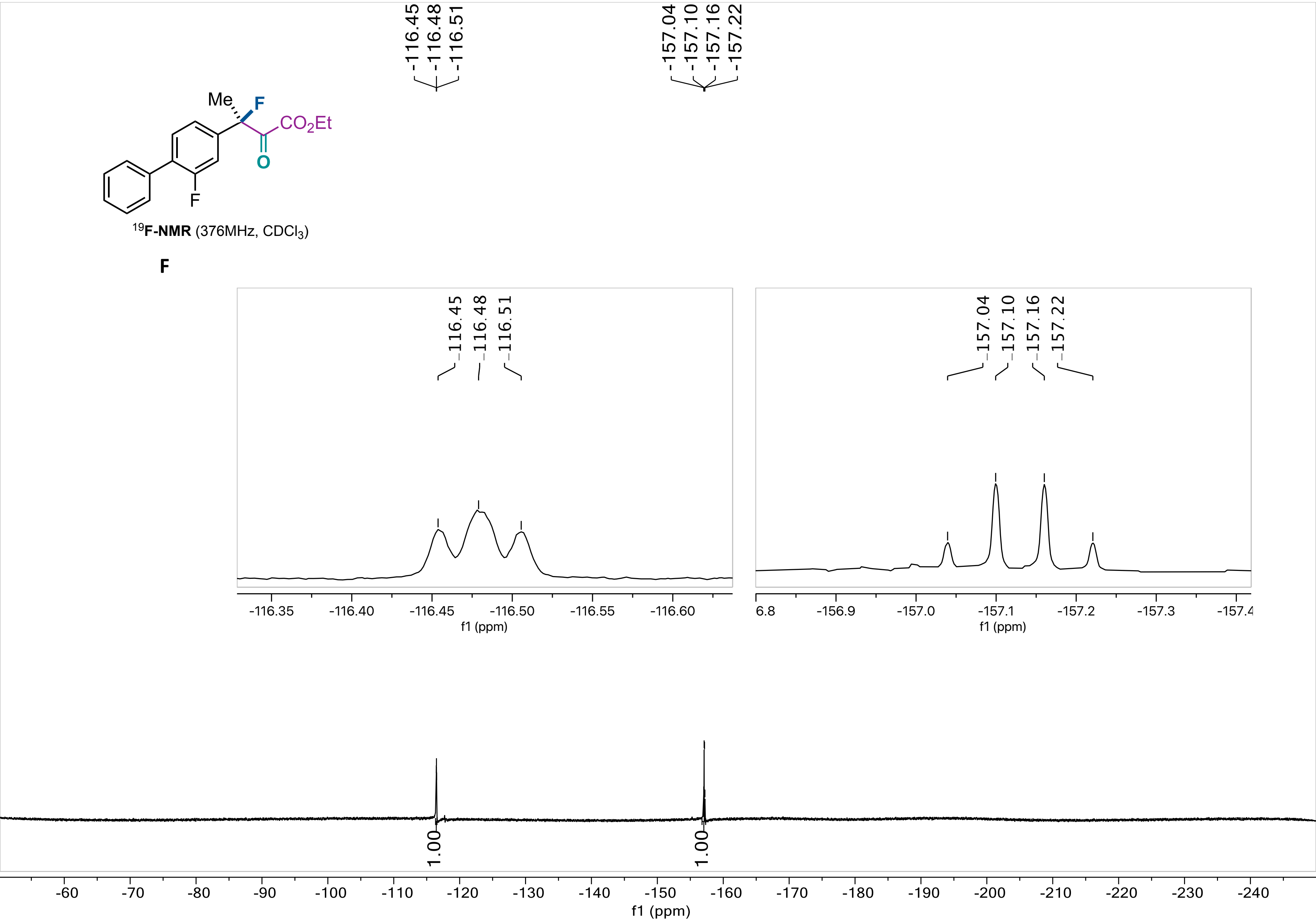
-116.45
-116.48
-116.51
-157.04
-157.10
-157.16
-157.22
Me
F
CO_2Et
O
F
^{19}F-NMR (376MHz, CDCl$_3$)
F
-116.35
-116.40
-116.45
-116.50
-116.55
-116.60
f1 (ppm)
-156.9
-157.0
-157.1
-157.2
-157.3
f1 (ppm)
1.00
1.00
-60
-70
-80
-90
-100
-110
-120
-130
-140
-150
-160
-170
-180
-190
-200
-210
-220
-230
-240
f1 (ppm)

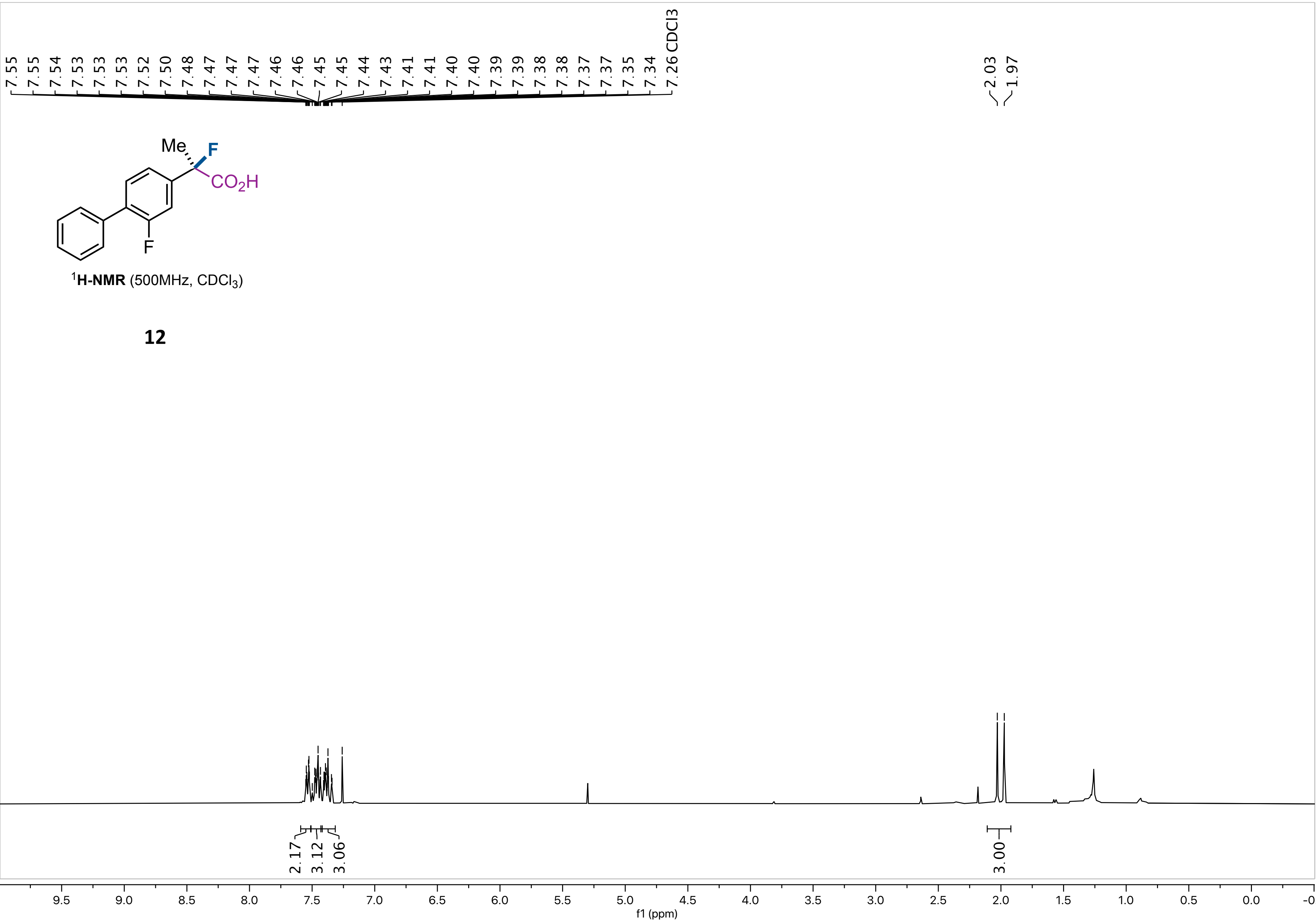
7.55
7.55
7.54
7.53
7.53
7.53
7.52
7.50
7.48
7.47
7.47
7.47
7.46
7.46
7.45
7.45
7.44
7.43
7.41
7.41
7.40
7.40
7.39
7.39
7.38
7.38
7.37
7.37
7.35
7.34
7.26 CDCl3
2.03
1.97
Me
F
CO_2H
F
^{1}**H-NMR** (500MHz, $CDCl_3$)
12
2.17
3.12
3.06
3.00
9.5
9.0
8.5
8.0
7.5
7.0
6.5
6.0
5.5
5.0
4.5
4.0
3.5
3.0
2.5
2.0
1.5
1.0
0.5
0.0
-0
f1 (ppm)

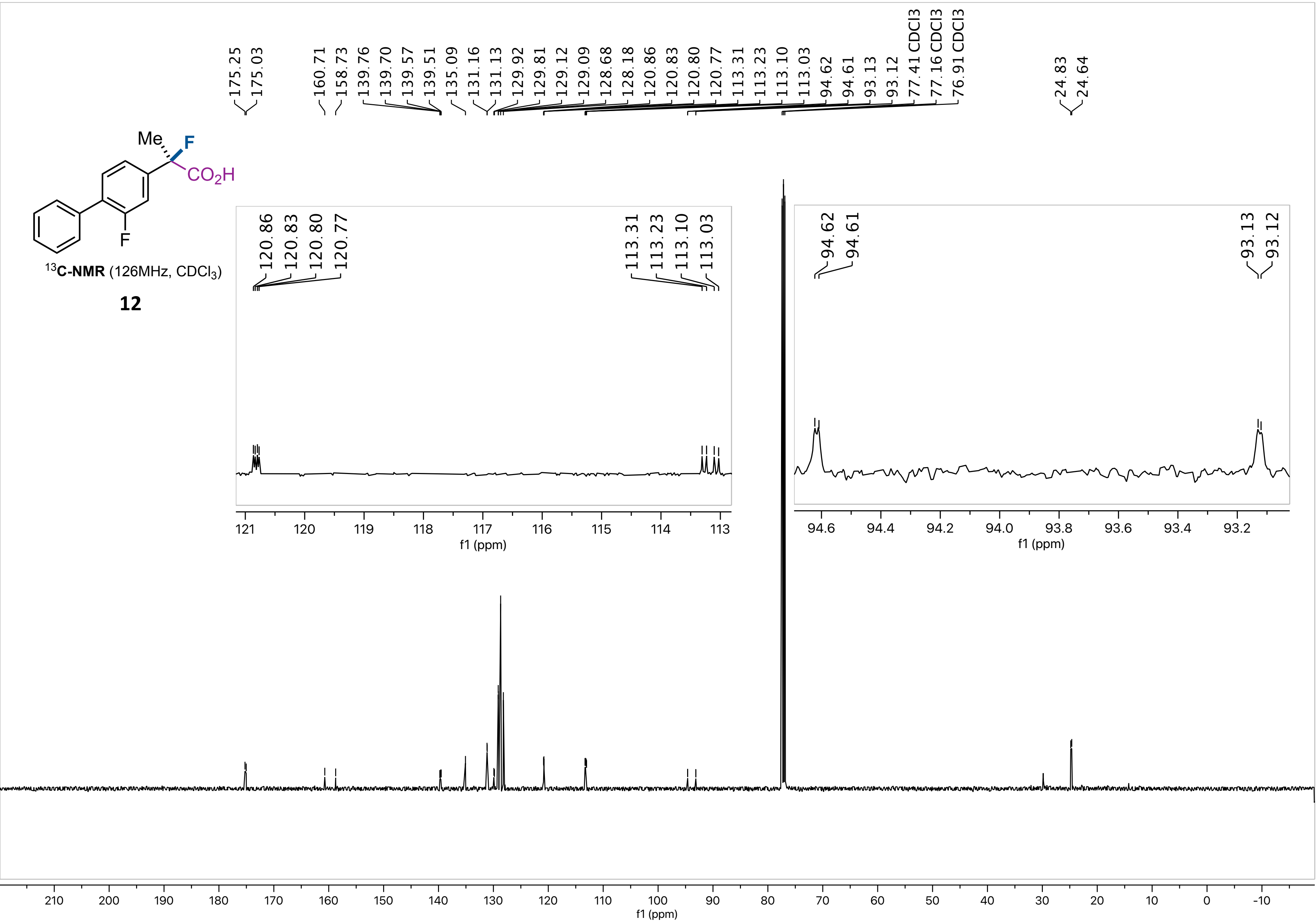

^{13}**C-NMR** (126MHz, $CDCl_3$)

12

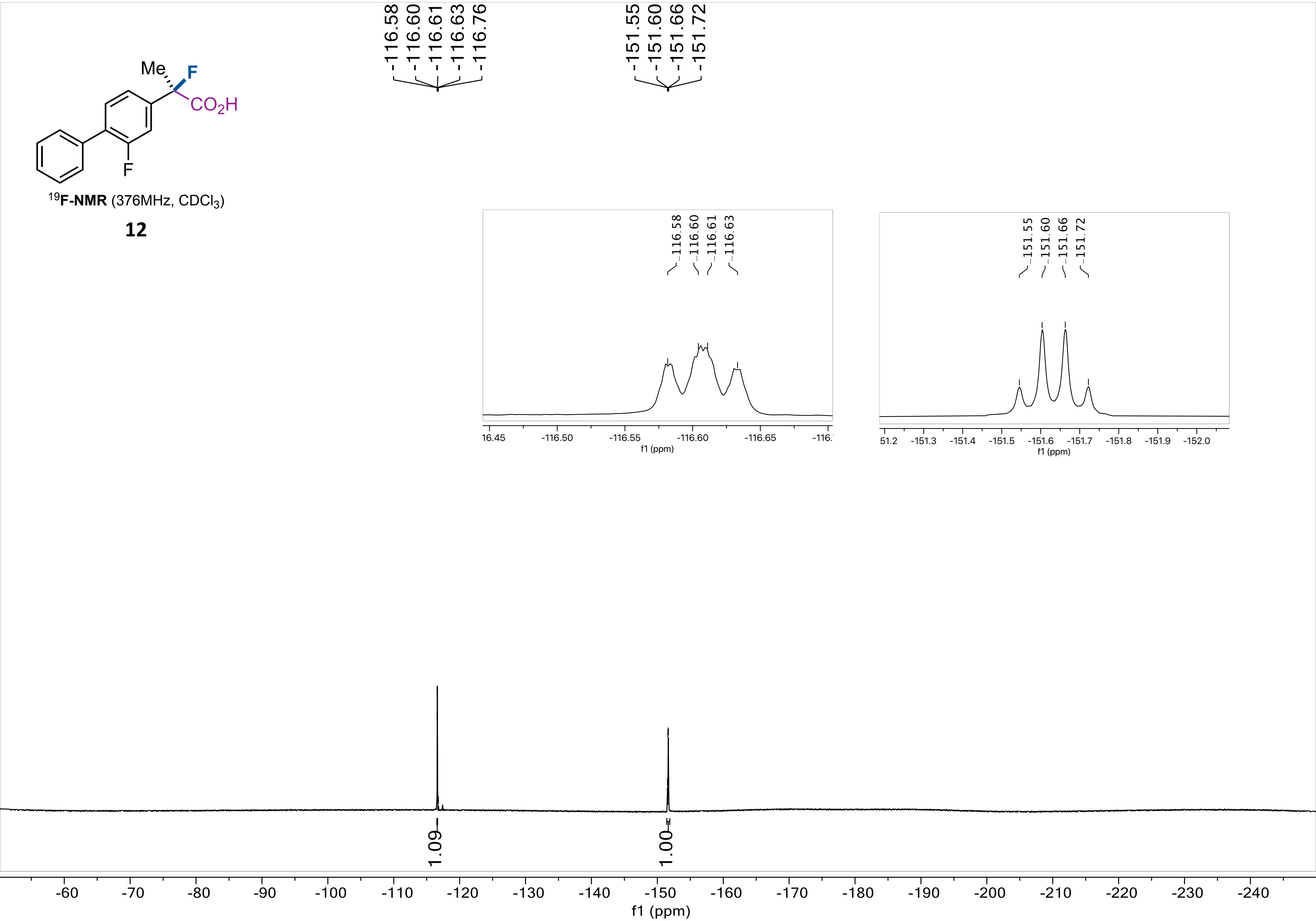
Me
F
CO2H
F
19F-NMR (376MHz, CDCl3)
12
-116.58
-116.60
-116.61
-116.63
-116.76
-151.55
-151.60
-151.66
-151.72
1.09
1.00
f1 (ppm)

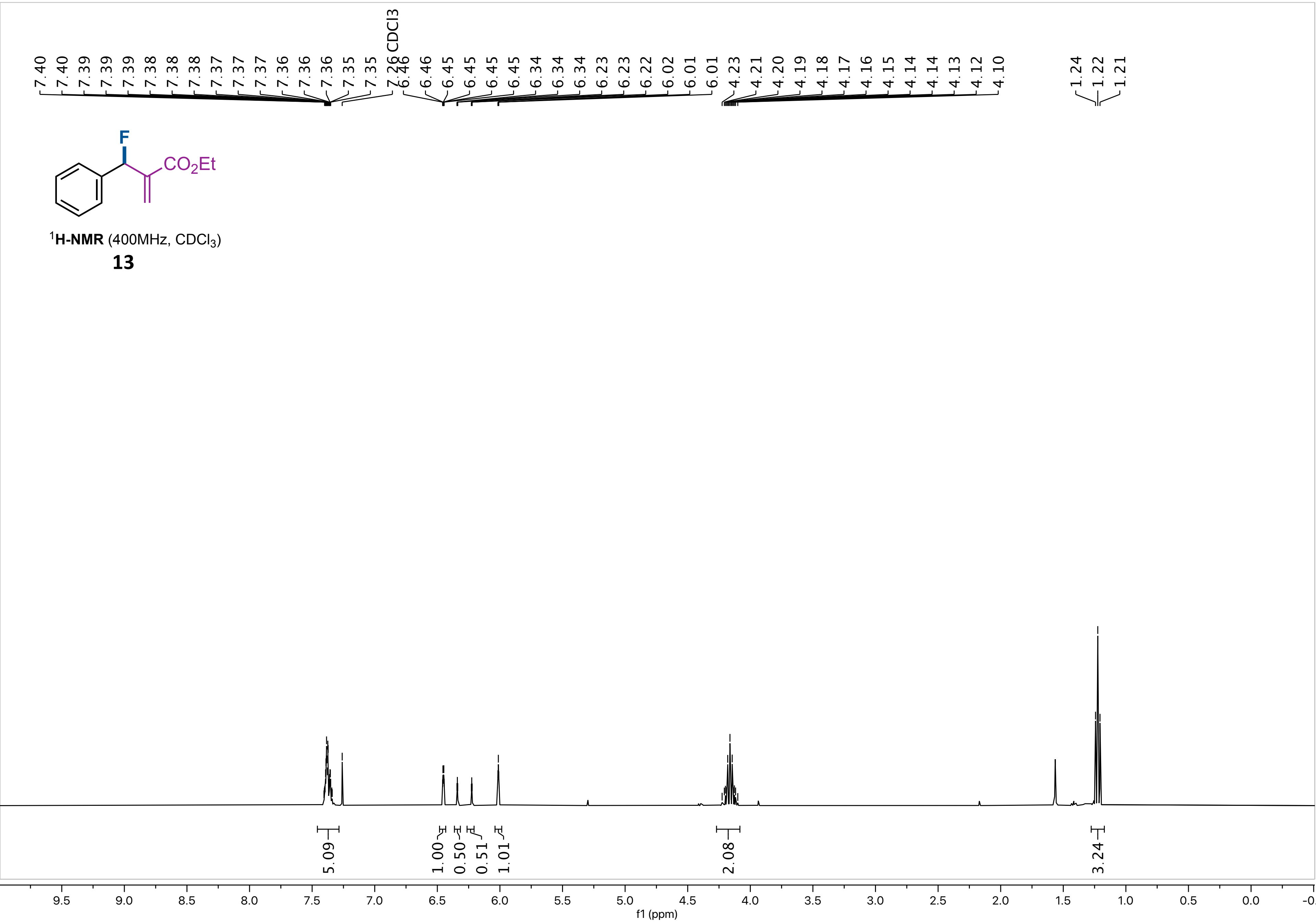
7.40 7.40 7.39 7.39 7.39 7.38 7.38 7.38 7.37 7.37 7.37 7.36 7.36 7.36 7.35 7.35 7.26 CDCl3 6.46 6.46 6.45 6.45 6.45 6.45 6.34 6.34 6.34 6.23 6.23 6.22 6.02 6.01 6.01 4.23 4.21 4.20 4.19 4.18 4.17 4.16 4.15 4.14 4.14 4.13 4.12 4.10
1.24 1.22 1.21
F
CO2Et
1H-NMR (400MHz, CDCl3)
13
5.09
1.00
0.50
0.51
1.01
2.08
3.24
9.5 9.0 8.5 8.0 7.5 7.0 6.5 6.0 5.5 5.0 4.5 4.0 3.5 3.0 2.5 2.0 1.5 1.0 0.5 0.0
f1 (ppm)

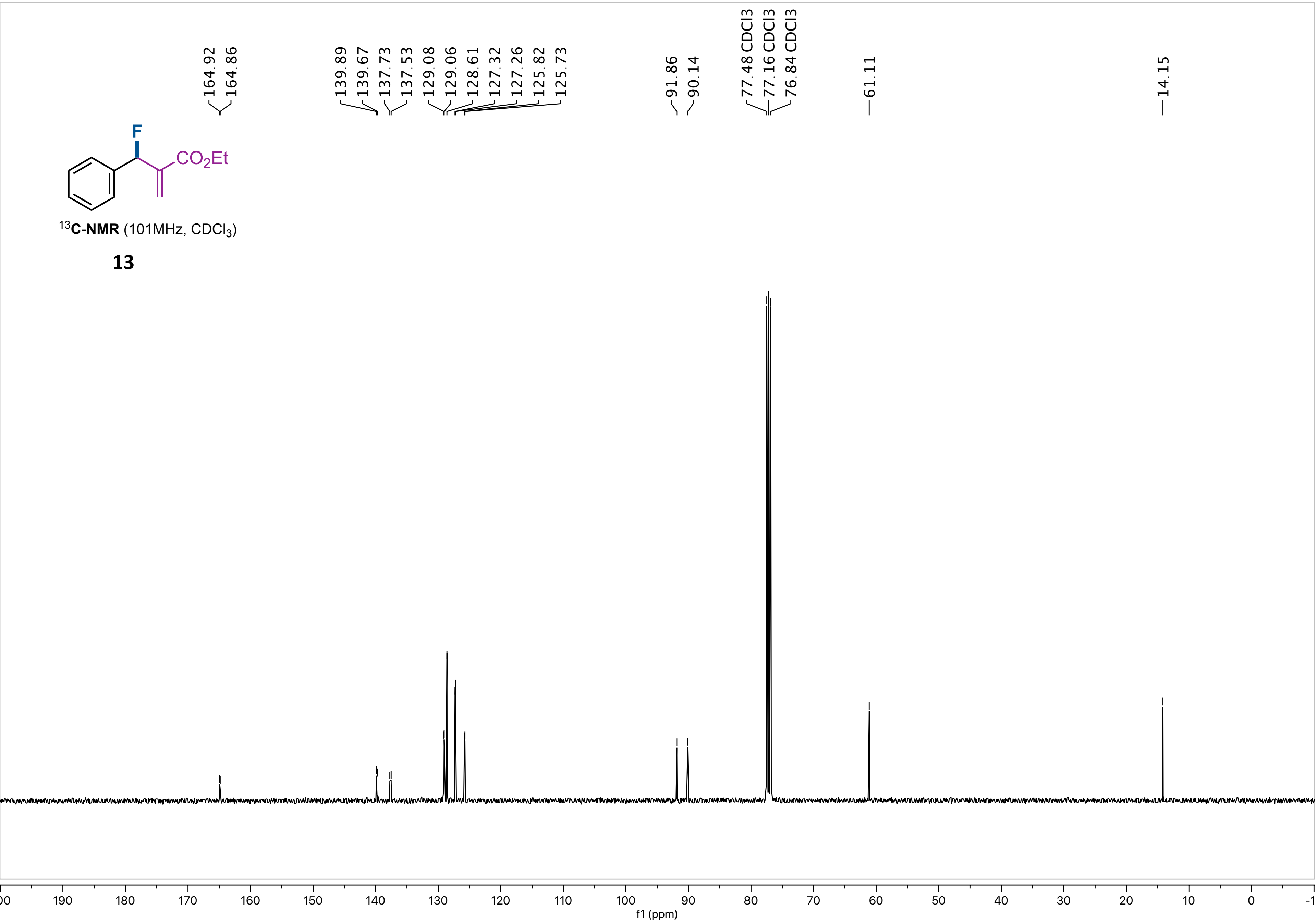

^{13}**C-NMR** (101MHz, $CDCl_3$)

13

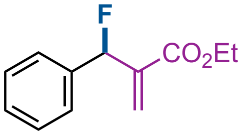

^{19}**F-NMR** (376MHz, $CDCl_3$)

13

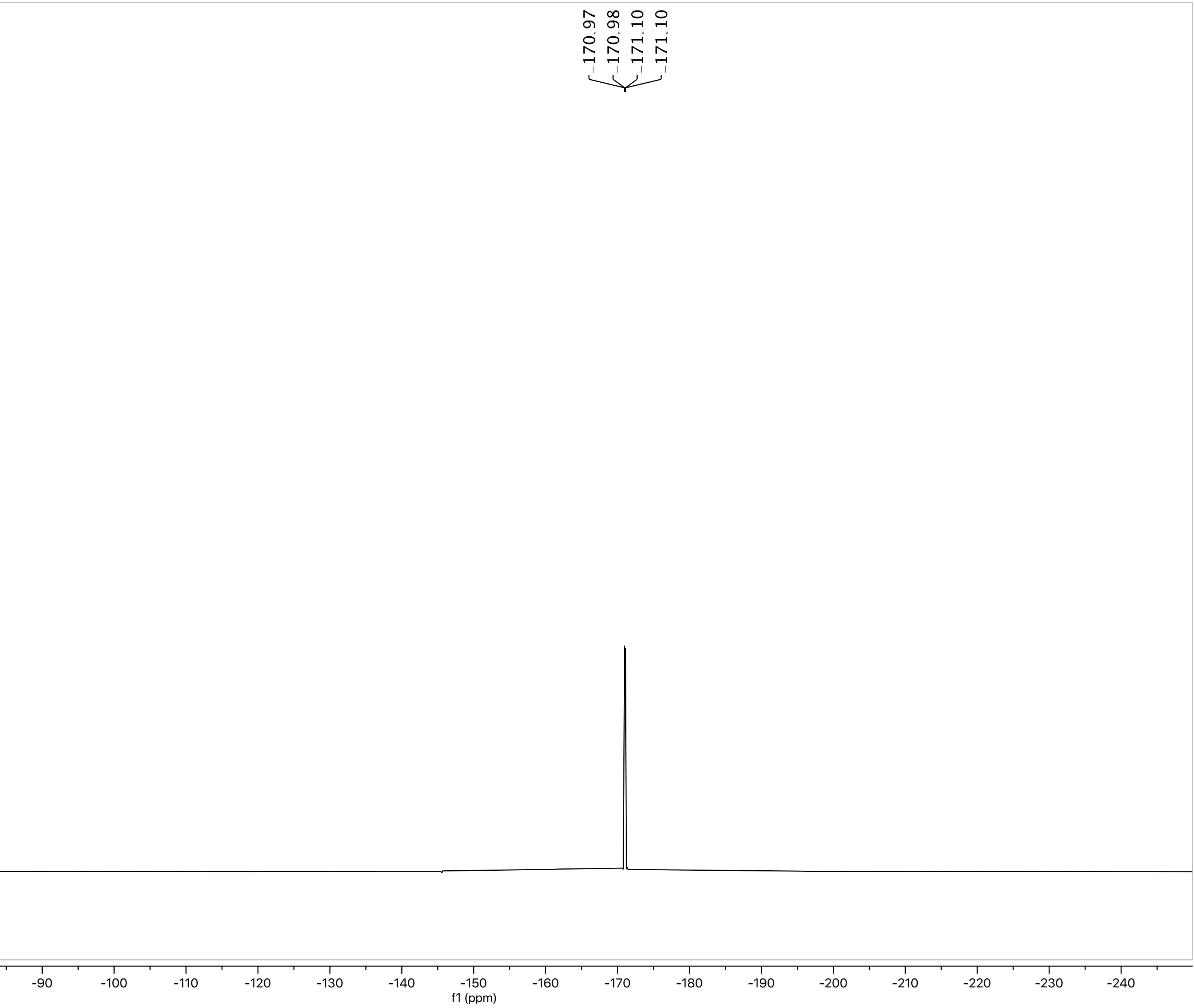

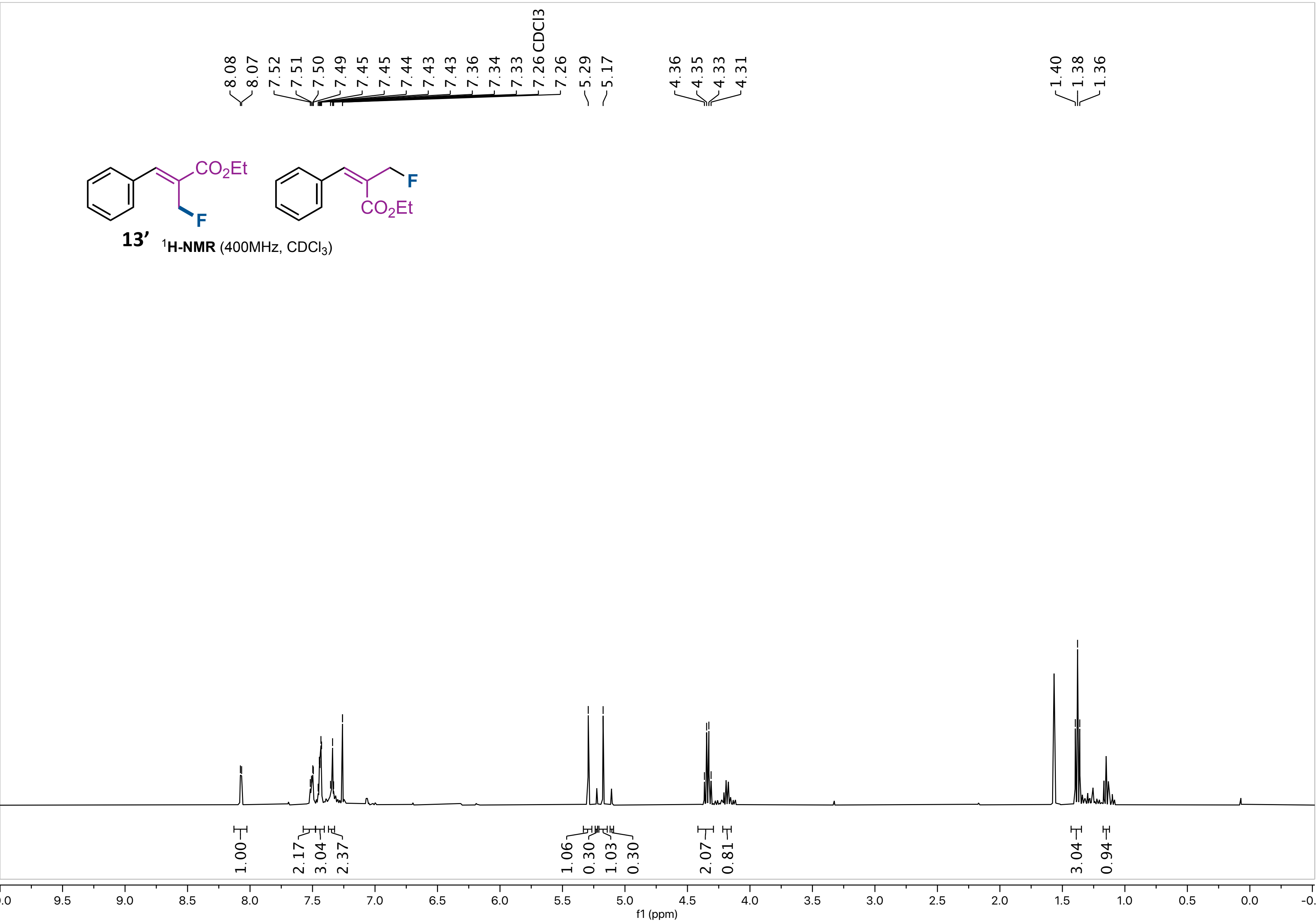
8.08
8.07
7.52
7.51
7.50
7.49
7.45
7.45
7.44
7.43
7.43
7.36
7.34
7.33
7.26 CDCl3
7.26
5.29
5.17
4.36
4.35
4.33
4.31
1.40
1.38
1.36
CO2Et
F
F
CO2Et
13'
1H-NMR (400MHz, CDCl3)
1.00
2.17
3.04
2.37
1.06
0.30
1.03
0.30
2.07
0.81
3.04
0.94
f1 (ppm)

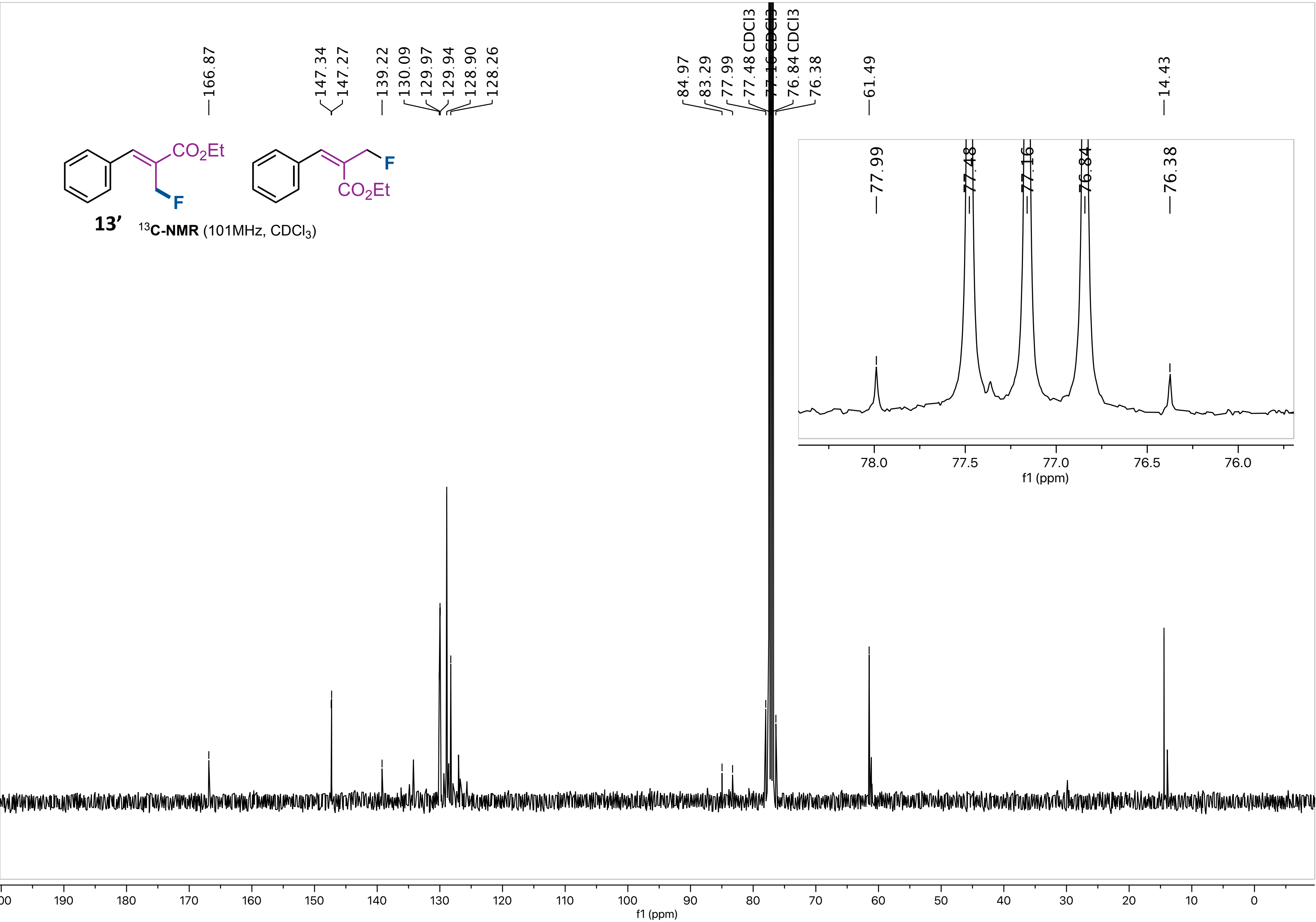

13’ ^{13}**C-NMR** (101MHz, $CDCl_3$)

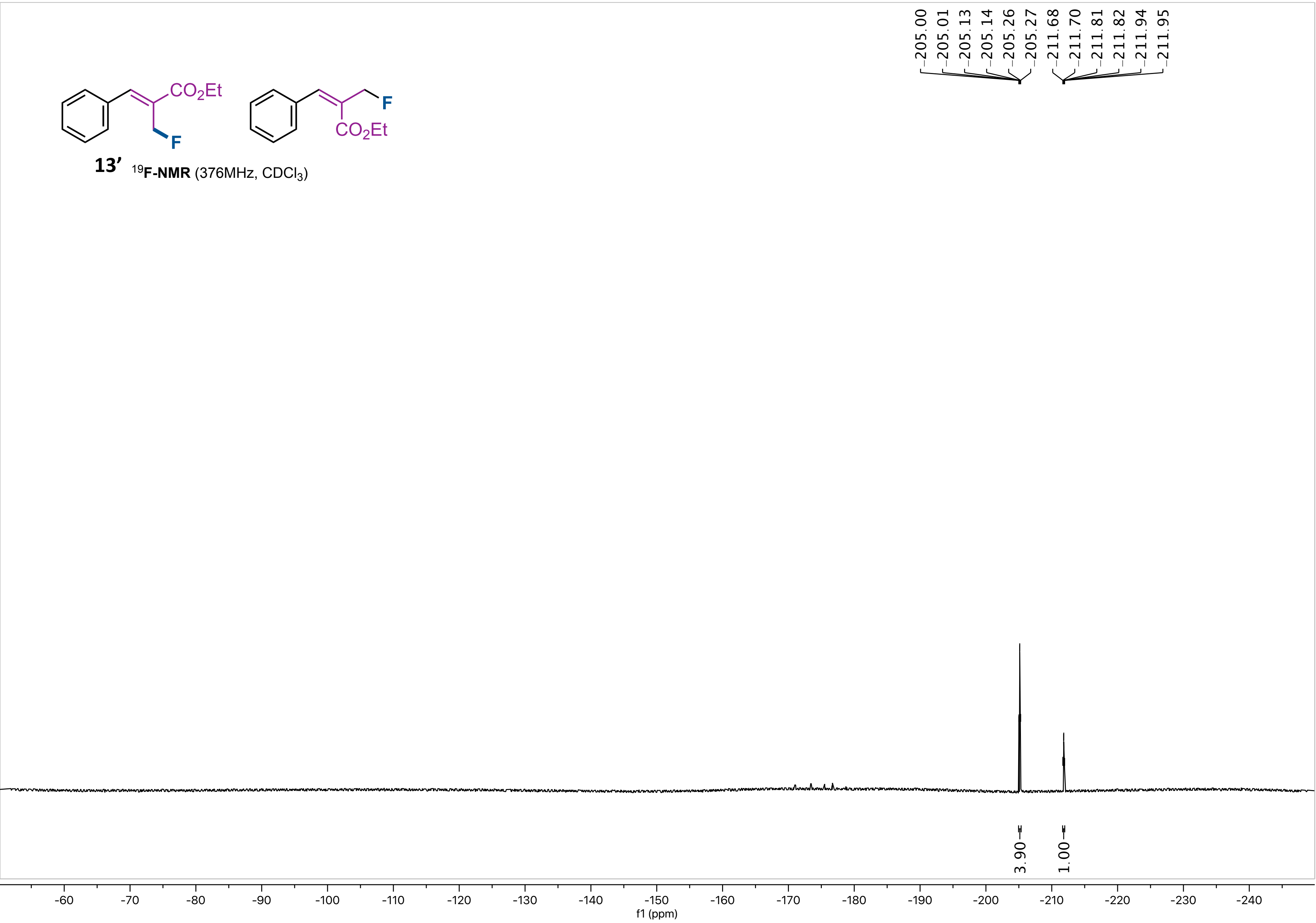
CO2Et
F
F
CO2Et
13'
19F-NMR (376MHz, CDCl3)
-205.00
-205.01
-205.13
-205.14
-205.26
-205.27
-211.68
-211.70
-211.81
-211.82
-211.94
-211.95
3.90
1.00
-60
-70
-80
-90
-100
-110
-120
-130
-140
-150
-160
-170
-180
-190
-200
-210
-220
-230
-240
f1 (ppm)

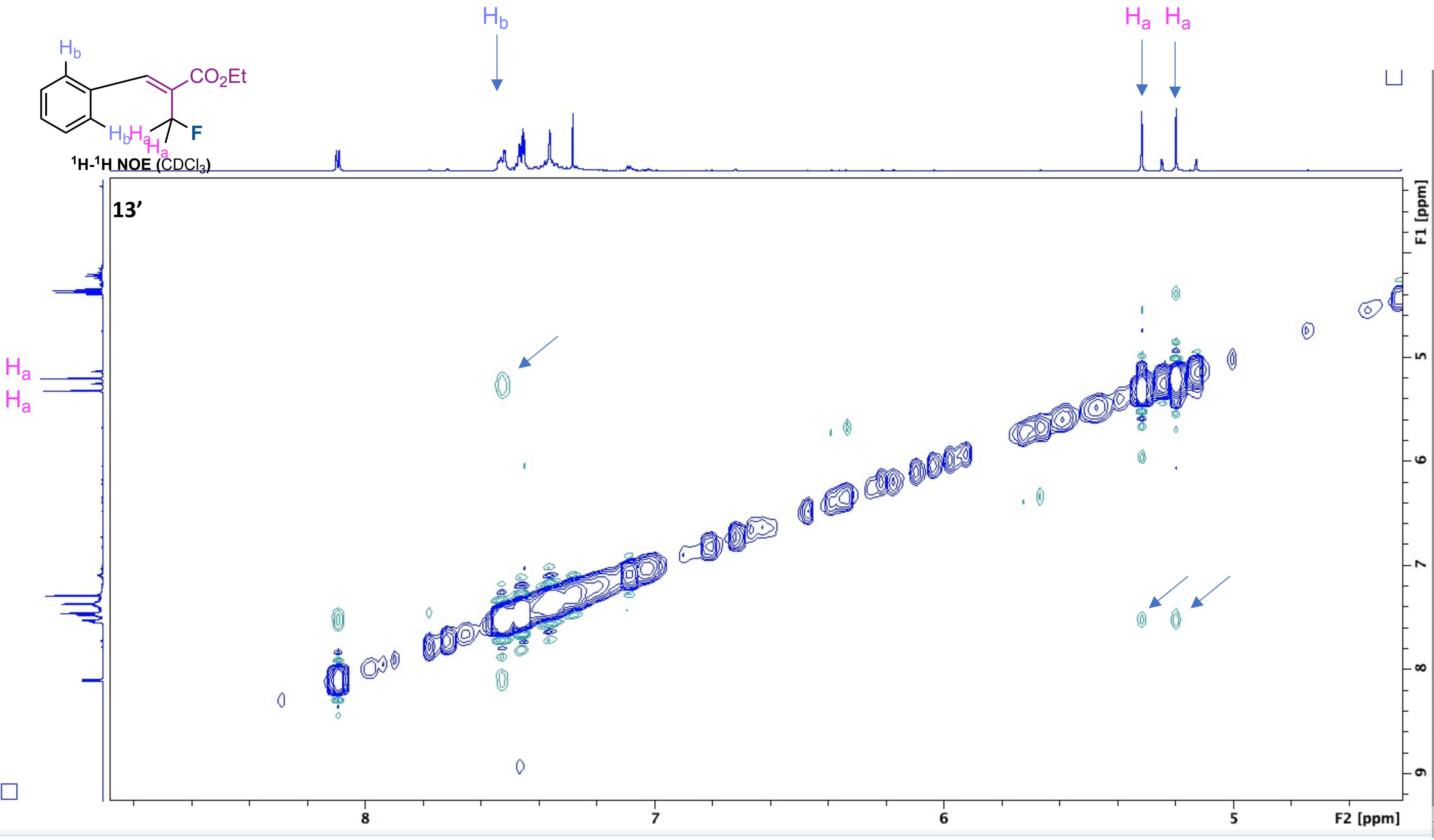
Hb
CO2Et
HbHa
Ha
F
1H-1H NOE (CDCl3)
13’
Hb
Ha
Ha
Ha
Ha
F1 [ppm]
F2 [ppm]
8
7
6
5
5
6
7
8
9

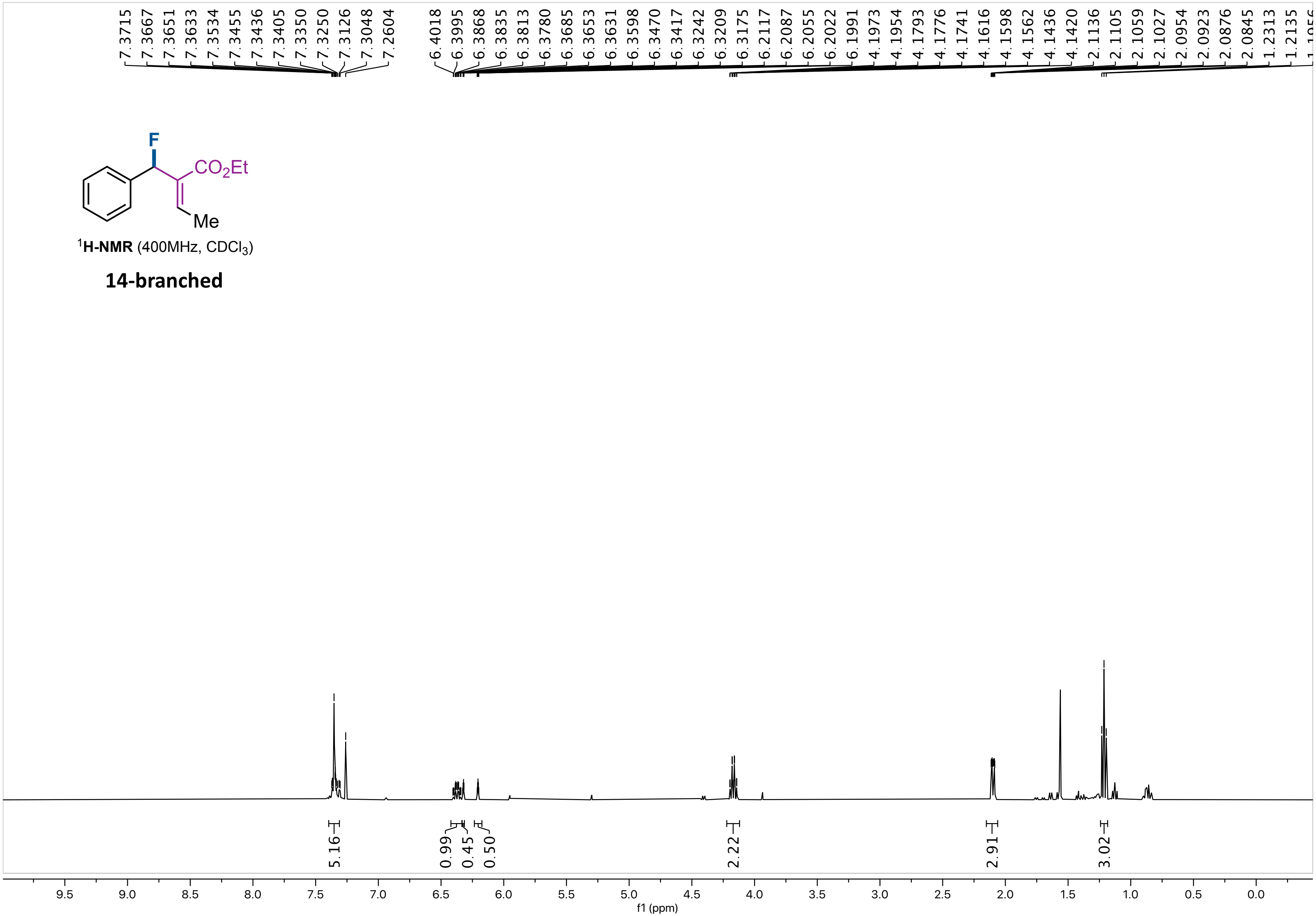
F
CO2Et
Me
1H-NMR (400MHz, CDCl3)
14-branched
7.3715
7.3667
7.3651
7.3633
7.3534
7.3455
7.3436
7.3405
7.3350
7.3250
7.3126
7.3048
7.2604
6.4018
6.3995
6.3868
6.3835
6.3813
6.3780
6.3685
6.3653
6.3631
6.3598
6.3470
6.3417
6.3242
6.3209
6.3175
6.2117
6.2087
6.2055
6.2022
6.1991
4.1973
4.1954
4.1793
4.1776
4.1741
4.1616
4.1598
4.1562
4.1436
4.1420
2.1136
2.1105
2.1059
2.1027
2.0954
2.0923
2.0876
2.0845
1.2313
1.2135
5.16
0.99
0.45
0.50
2.22
2.91
3.02
9.5
9.0
8.5
8.0
7.5
7.0
6.5
6.0
5.5
5.0
4.5
4.0
3.5
3.0
2.5
2.0
1.5
1.0
0.5
0.0
f1 (ppm)

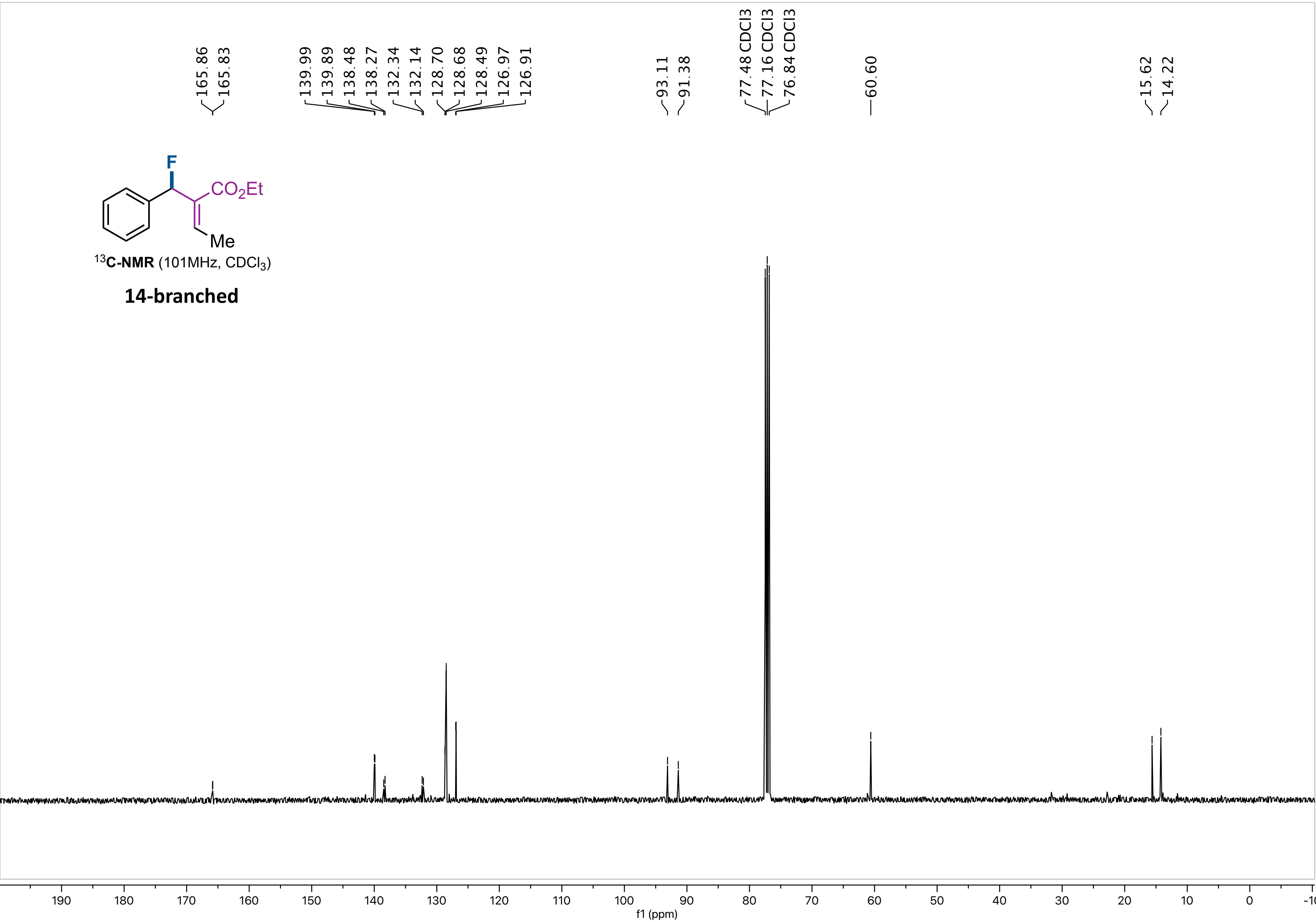
165.86
165.83
139.99
139.89
138.48
138.27
132.34
132.14
128.70
128.68
128.49
126.97
126.91
93.11
91.38
77.48 CDCl3
77.16 CDCl3
76.84 CDCl3
60.60
15.62
14.22
F
CO_2Et
Me
^{13}C-NMR (101MHz, $CDCl_3$)
14-branched
f1 (ppm)

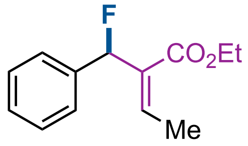

^{19}**F-NMR** (376MHz, $CDCl_3$)

14-branched

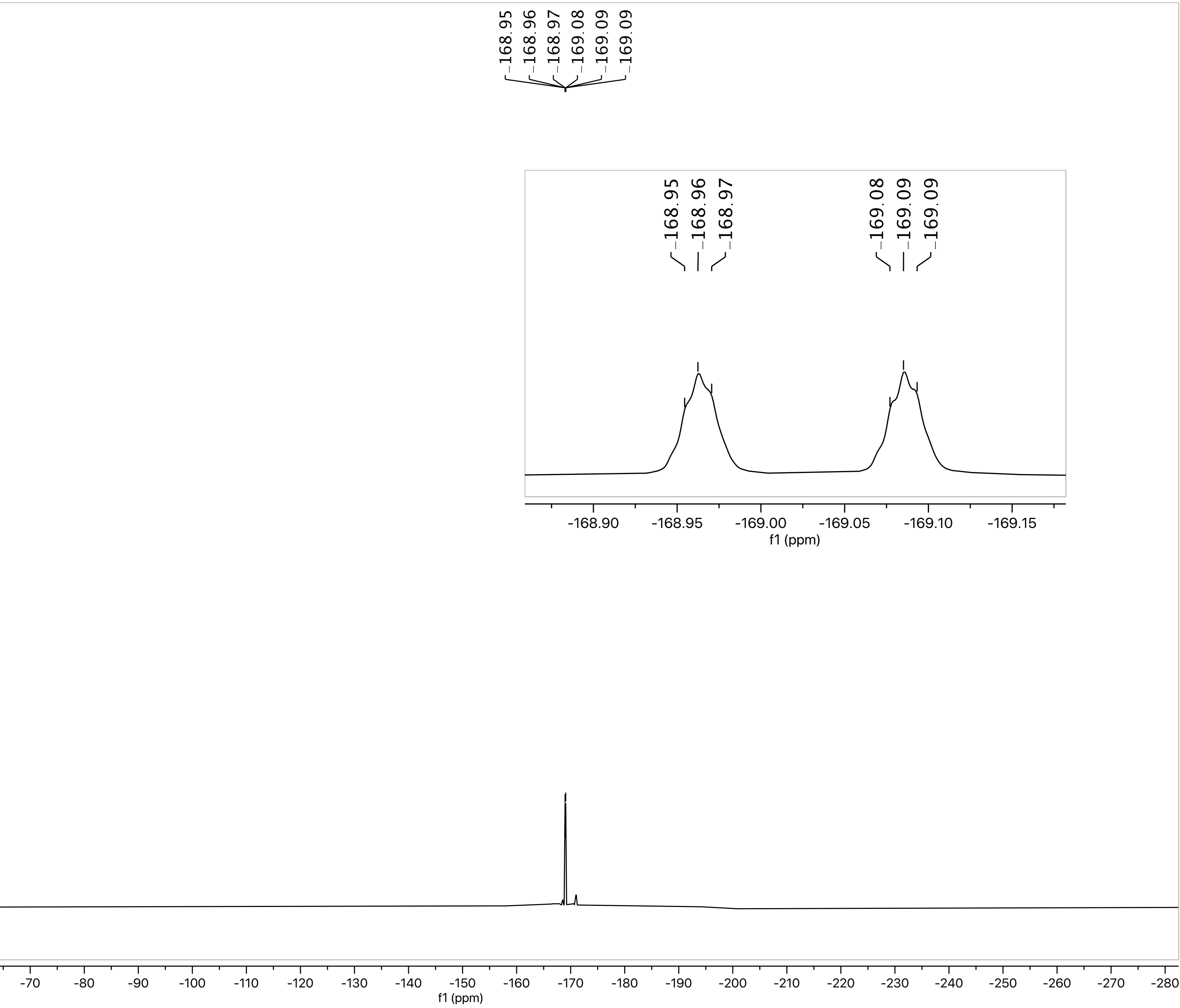

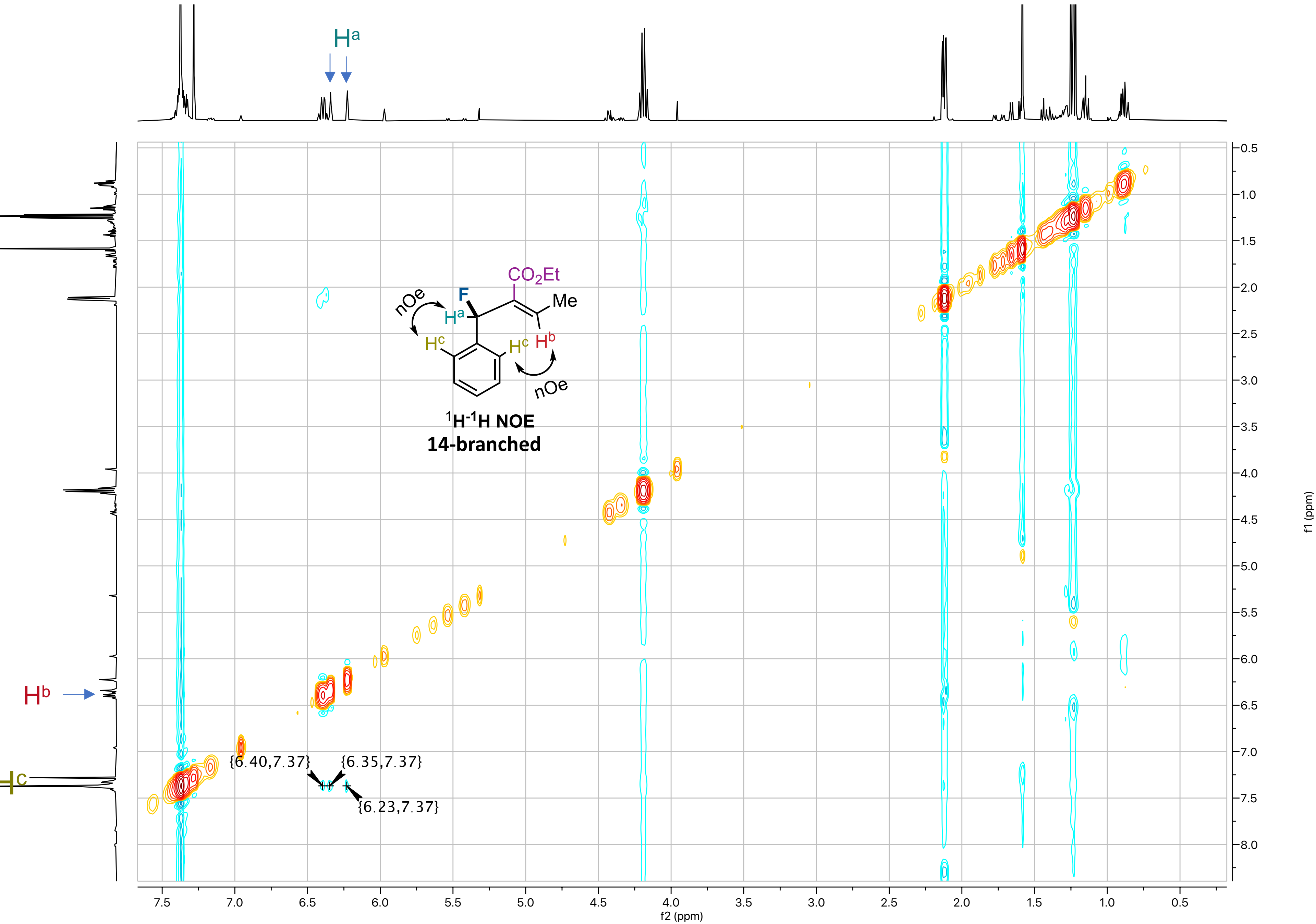
Ha
CO2Et
F
Me
nOe
Ha
Hc
Hc
Hb
nOe
1H-1H NOE
14-branched
Hb
Hc
{6.40,7.37}
{6.35,7.37}
{6.23,7.37}
f2 (ppm)
f1 (ppm)

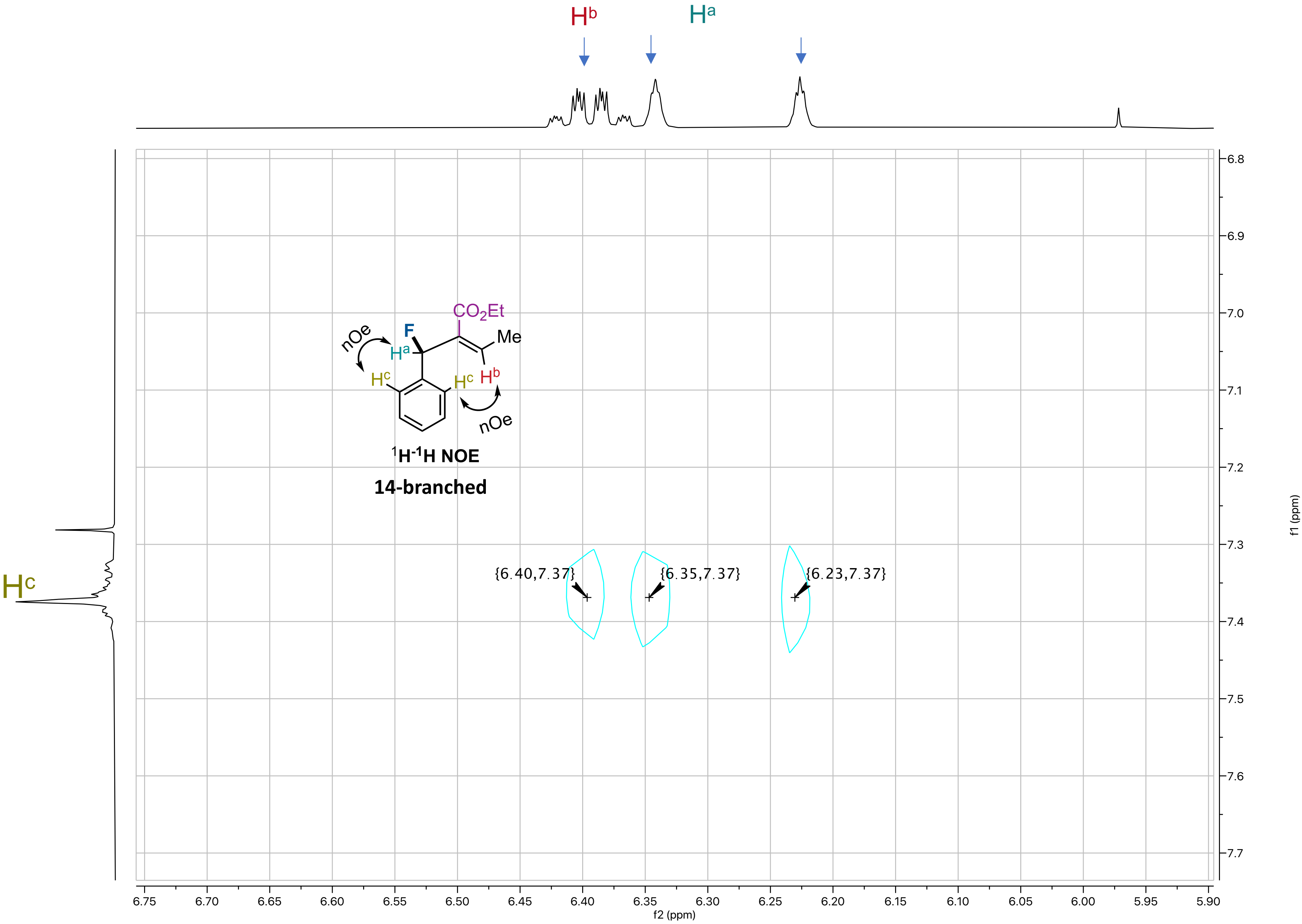
Hb
Ha
Hc
CO2Et
F
Me
Ha
Hc
Hc
Hb
nOe
nOe
1H-1H NOE
14-branched
{6.40,7.37}
{6.35,7.37}
{6.23,7.37}
f2 (ppm)
f1 (ppm)

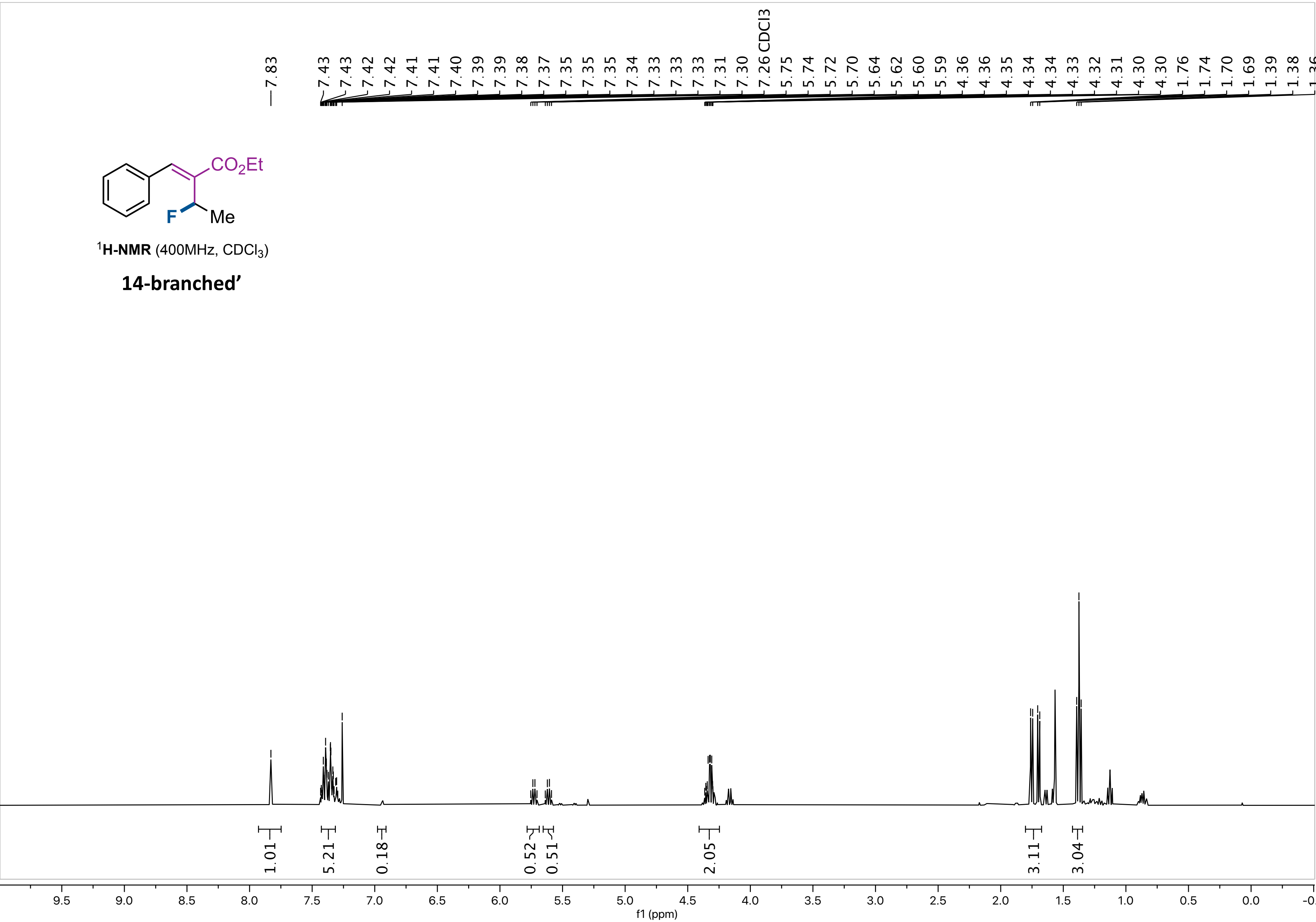

^{1}**H-NMR** (400MHz, $CDCl_3$)

14-branched’

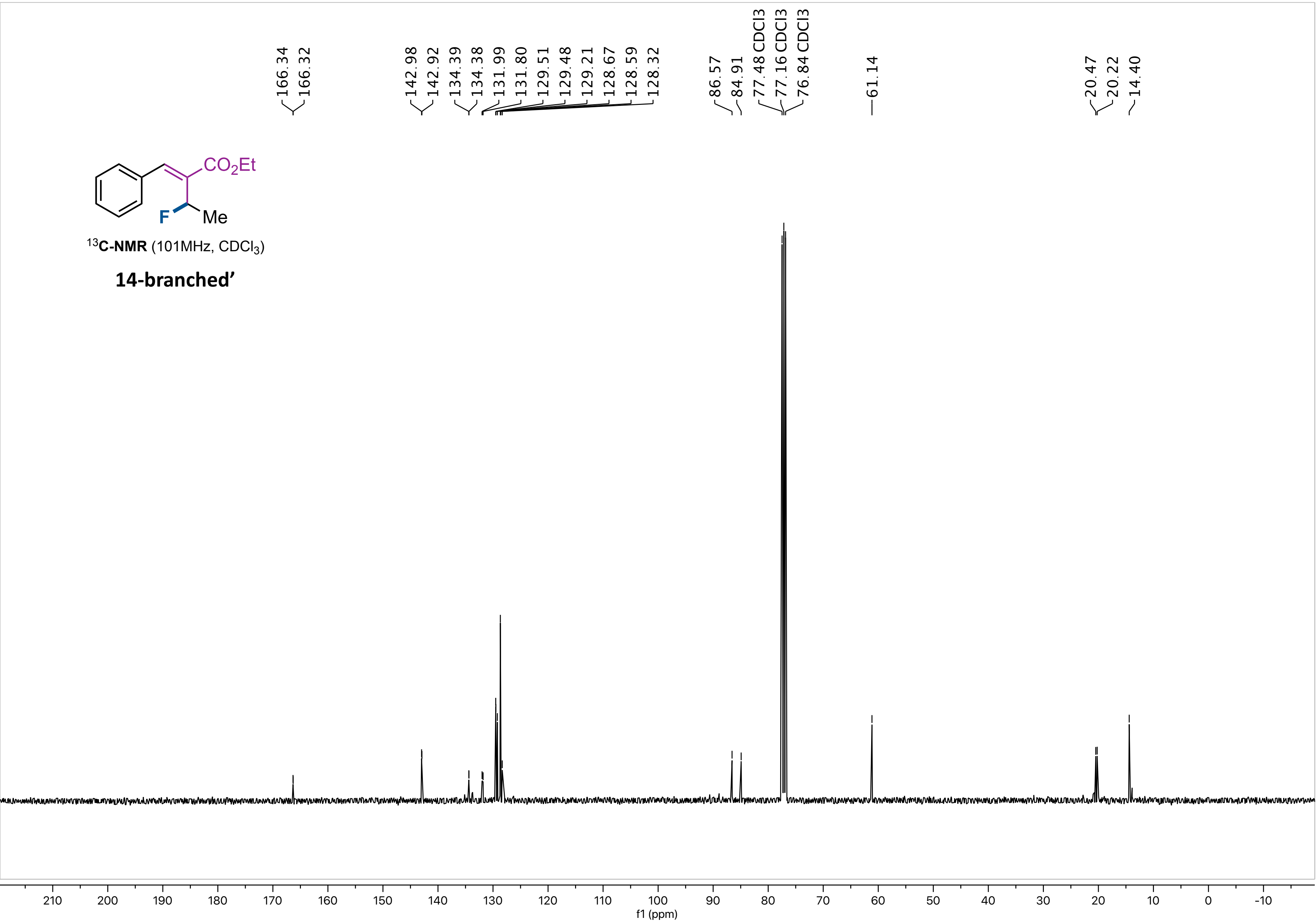
166.34
166.32
142.98
142.92
134.39
134.38
131.99
131.80
129.51
129.48
129.21
128.67
128.59
128.32
86.57
84.91
77.48 CDCl3
77.16 CDCl3
76.84 CDCl3
61.14
20.47
20.22
14.40
CO2Et
F
Me
13C-NMR (101MHz, CDCl3)
14-branched’
210
200
190
180
170
160
150
140
130
120
110
100
90
80
70
60
50
40
30
20
10
0
-10
f1 (ppm)

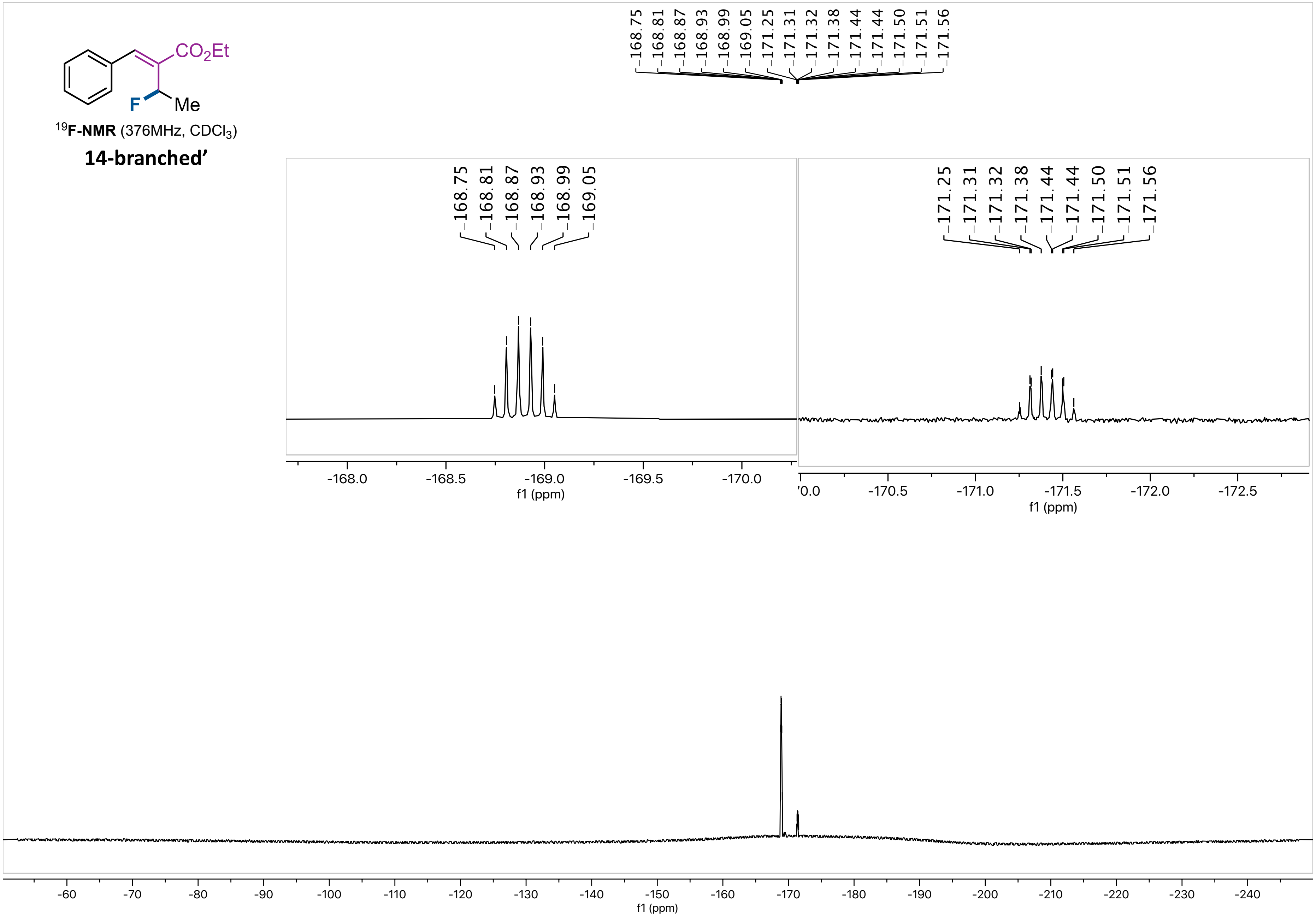
CO2Et
F
Me
19F-NMR (376MHz, CDCl3)
14-branched’
-168.75
-168.81
-168.87
-168.93
-168.99
-169.05
-171.25
-171.31
-171.32
-171.38
-171.44
-171.44
-171.50
-171.51
-171.56
f1 (ppm)

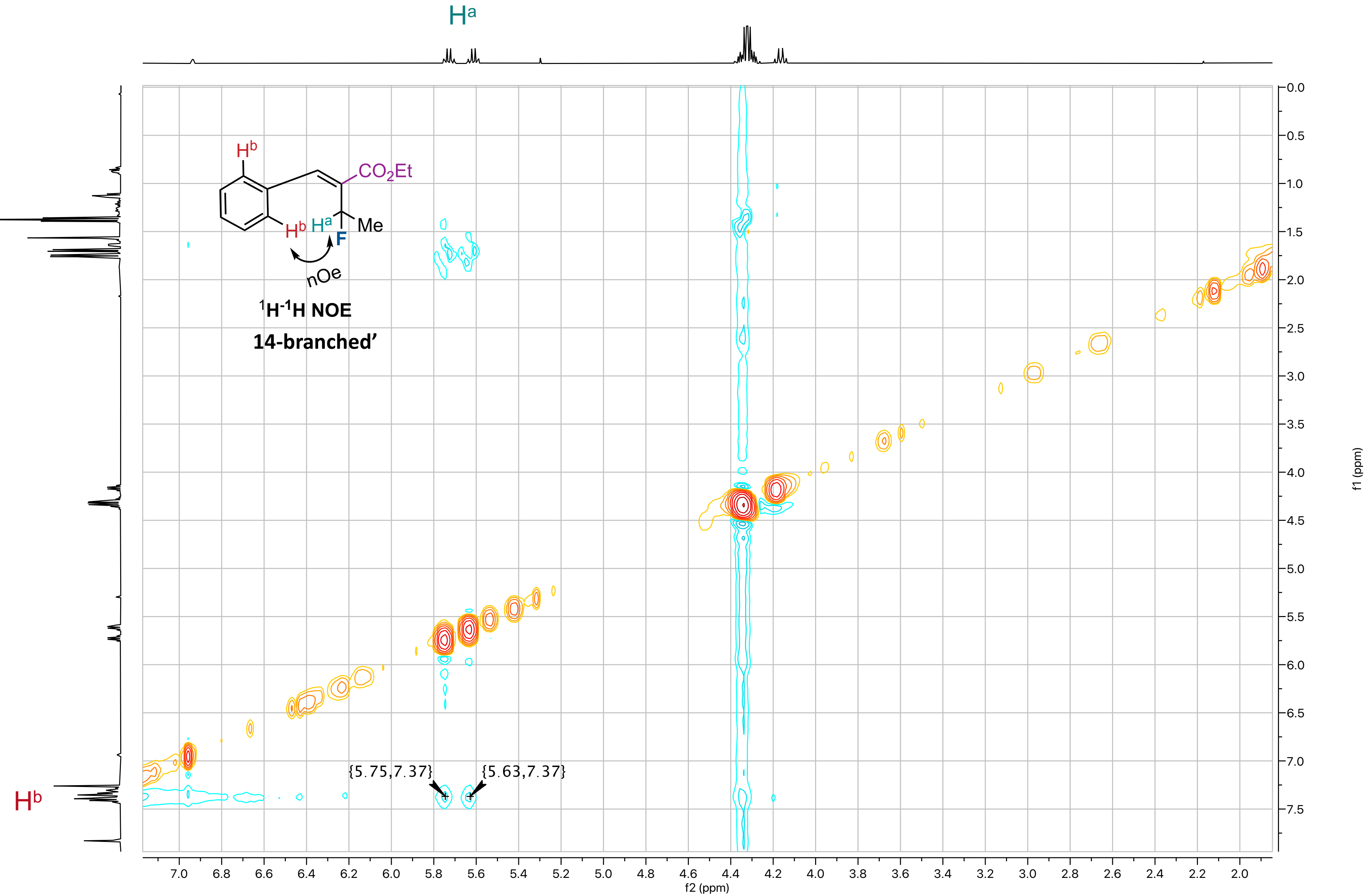

Hª
Hᵇ
CO₂Et
Hᵇ Hª
Me
F
nOe
¹H-¹H NOE
14-branched'
{5.75,7.37}
{5.63,7.37}
f2 (ppm)
f1 (ppm)
